# Supplementary material for: A rare gain of function mutation in a wheat tandem kinase confers resistance to powdery mildew
Source: Nat Commun. 2020 Feb 3;11:680. doi: 10.1038/s41467-020-14294-0 (PMC6997164; doi:10.1038/s41467-020-14294-0)
Supplement: Supplementary file 1 — Supplementary Information [file 41467_2020_14294_MOESM1_ESM.pdf]

Supplementary Information for “A rare gain of function mutation in a wheat tandem kinase confers resistance to powdery mildew” by Ping Lu et al.

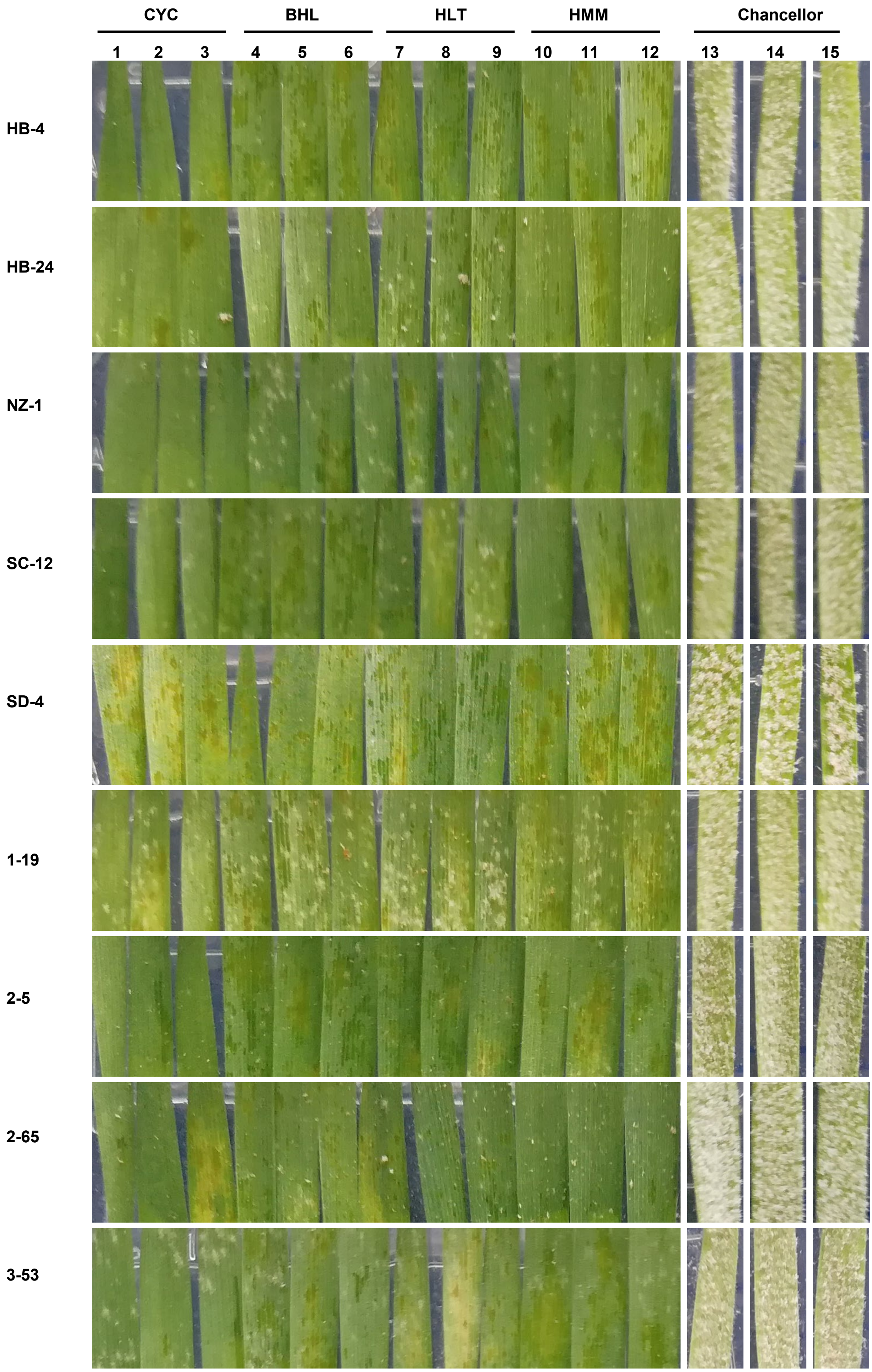
CYC
BHL
HLT
HMM
Chancellor
1
2
3
4
5
6
7
8
9
10
11
12
13
14
15
HB-4
HB-24
NZ-1
SC-12
SD-4
1-19
2-5
2-65
3-53

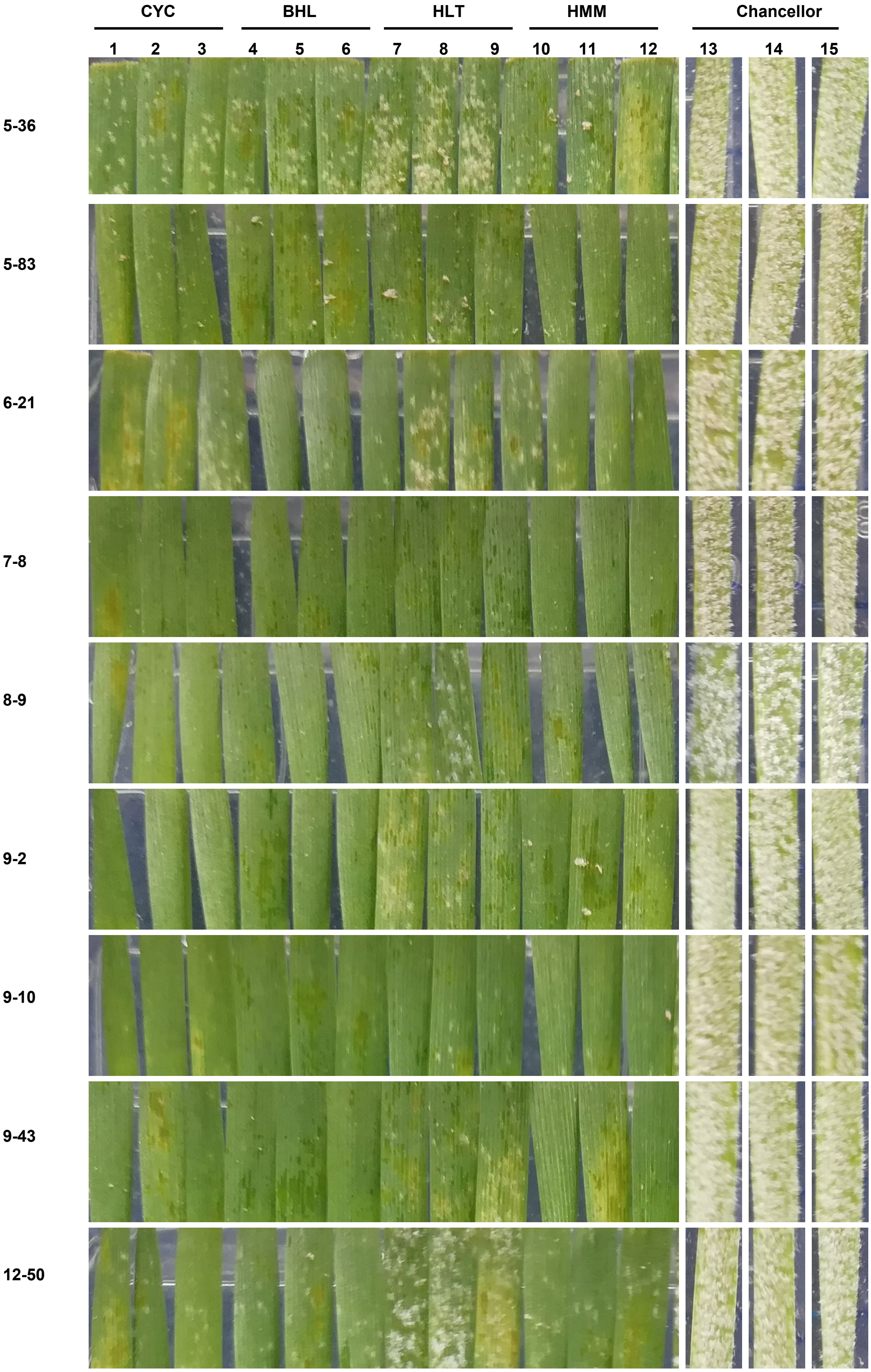
CYC
BHL
HLT
HMM
Chancellor
1
2
3
4
5
6
7
8
9
10
11
12
13
14
15
5-36
5-83
6-21
7-8
8-9
9-2
9-10
9-43
12-50

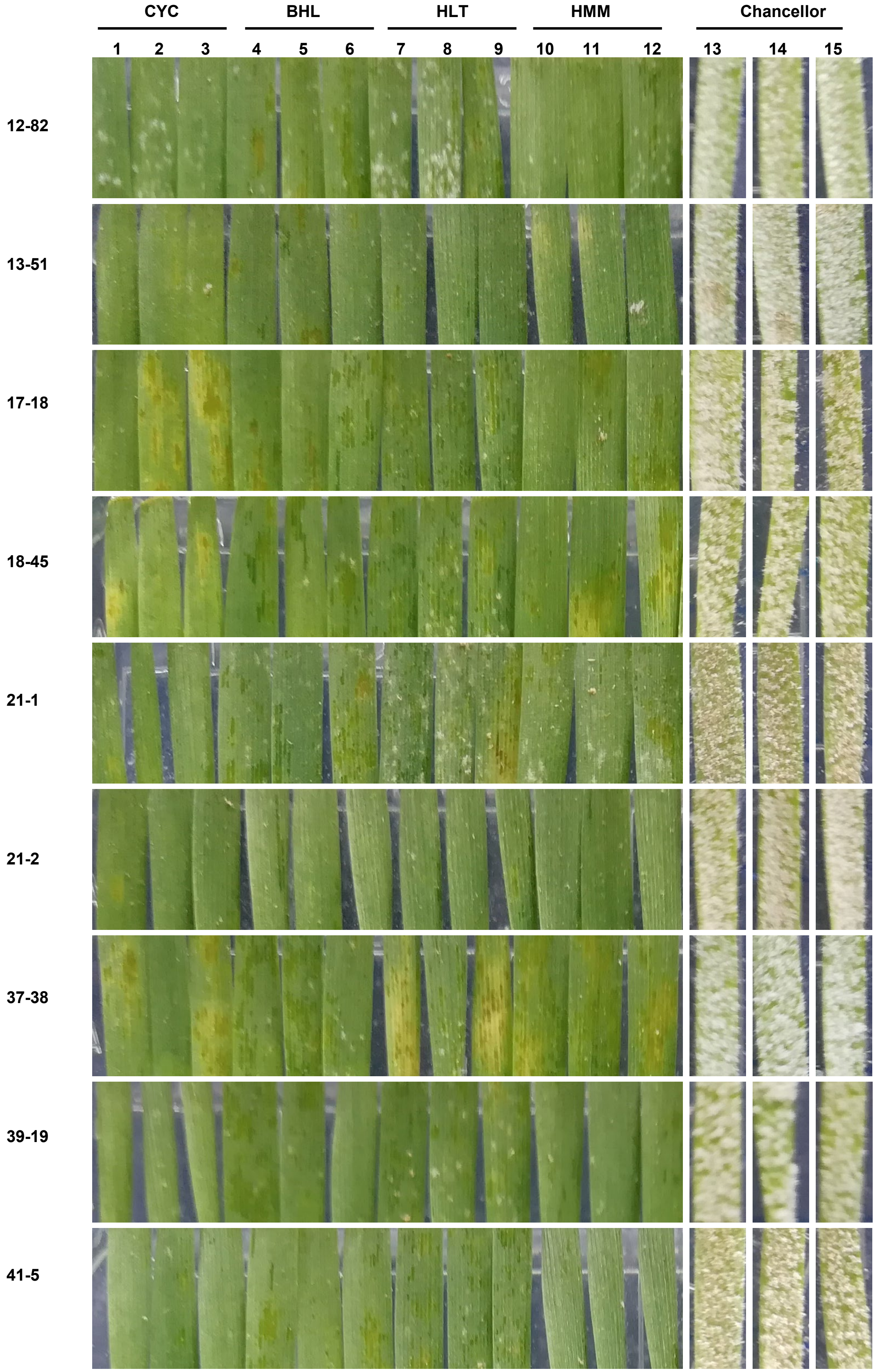

CYC
BHL
HLT
HMM
Chancellor
1
2
3
4
5
6
7
8
9
10
11
12
13
14
15
12-82
13-51
17-18
18-45
21-1
21-2
37-38
39-19
41-5

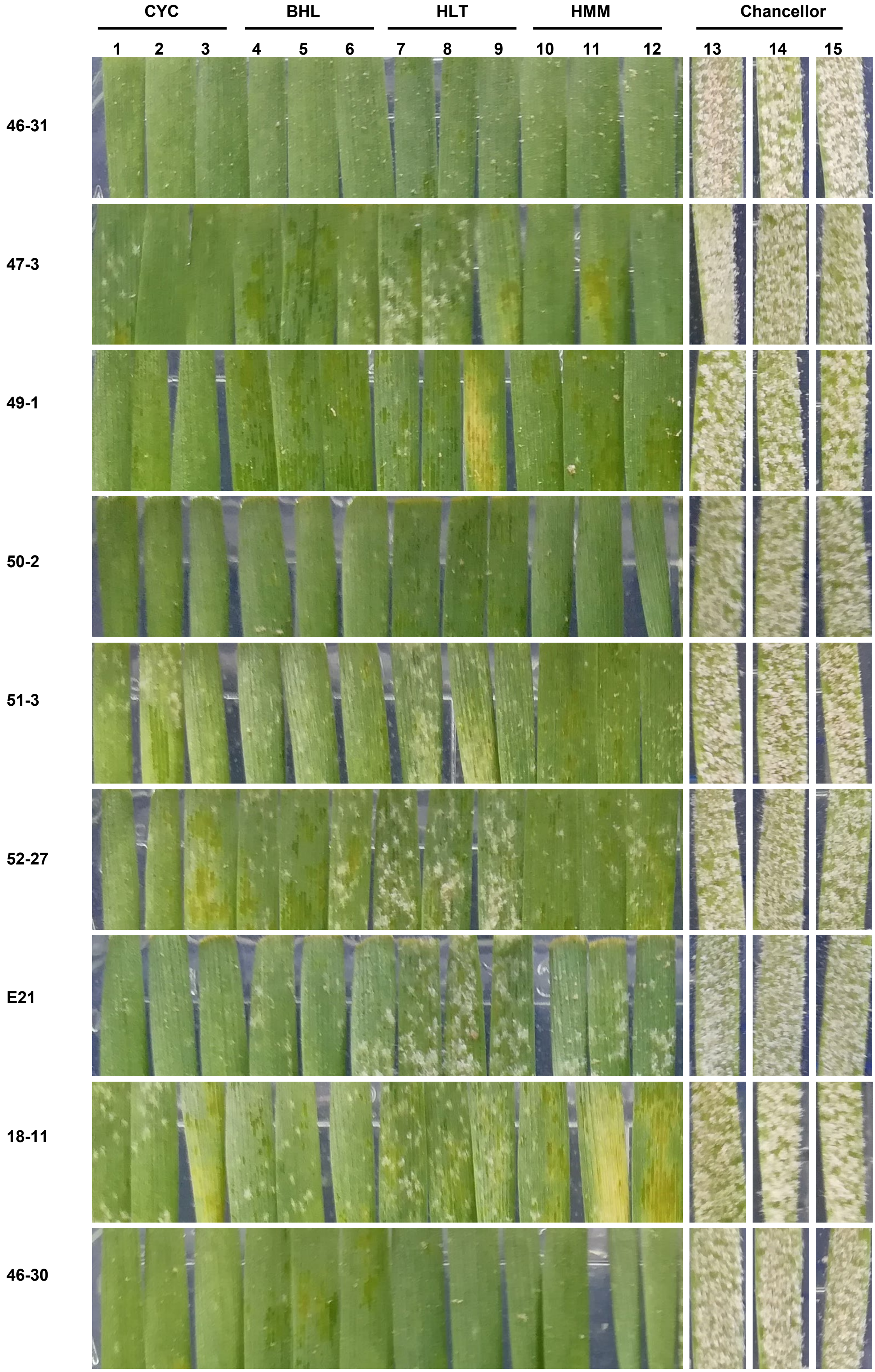

Supplementary Figure 1 Infection reactions of Chinese wheat landraces CYC, BHL, HLT and Hongmangmai (HMM) to 36 tested *Bgt* isolates. Common wheat cultivar Chancellor were used as the susceptible control. Three leaves from 3 independent plants (two-week-old) of CYC (1-3), BHL (4-6), HLT (7-9), HMM (10-12), and Chancellor (13-15) were inoculated with each *Bgt* isolate. Representative leaves were photographed at 10 dpi.

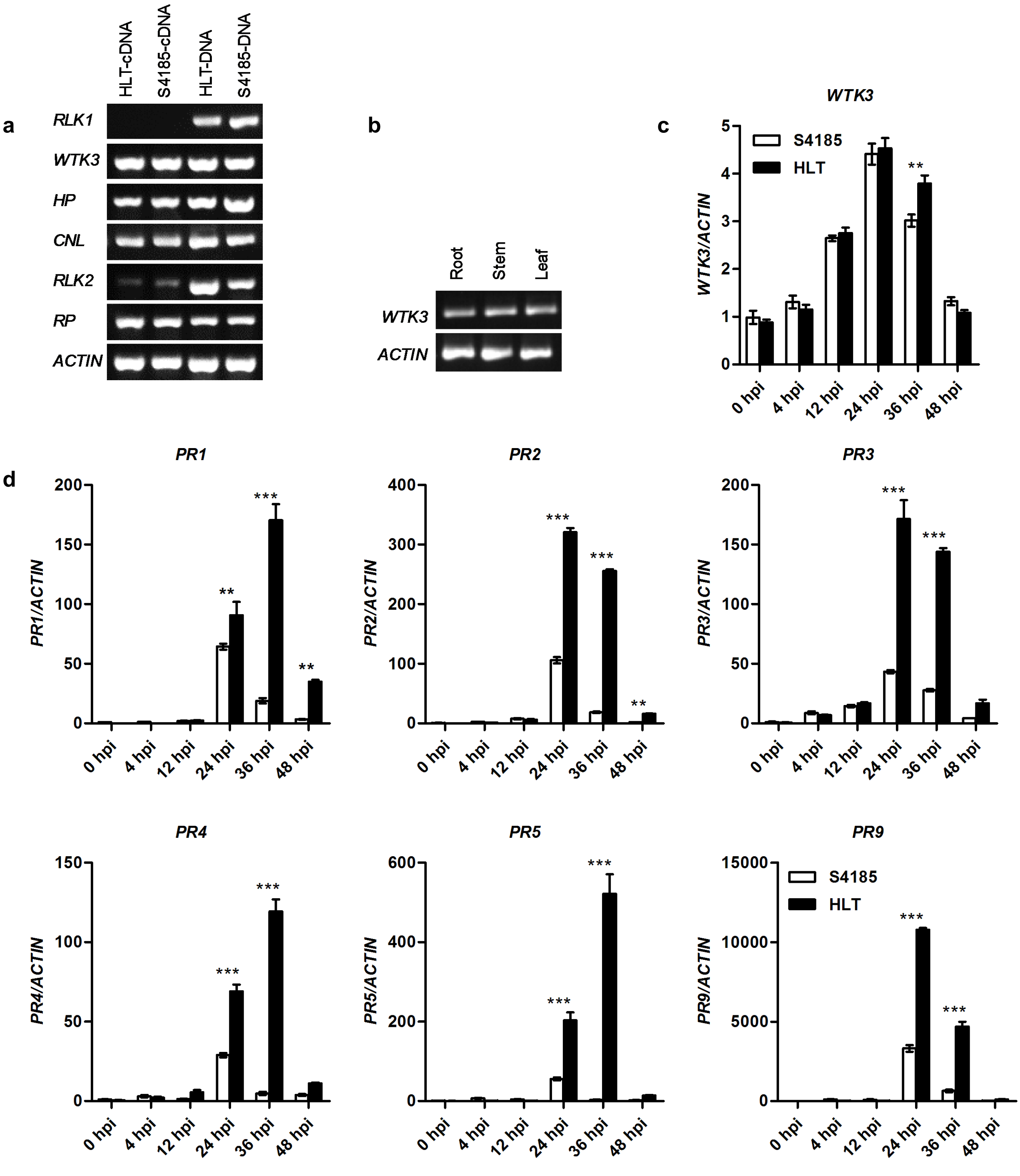

Supplementary Figure 2 Expression patterns of the *MlHLT* candidates, *WTK3 and PR* genes

a, E xpression analysis of *MlHLT* candidate genes in HLT and S4185. **b,** *WTK3* expression survey in root, stem, and leaf of HLT by RT-PCR. **c,** Relative expression level of $WTK3^{HLT}$ and $WTK3^{S4185}$ alleles in 2-week-old seeding plants at 0, 4, 12, 24, 36, and 48 hpi with *Bgt* isolate E09. **d,** Relative expression level of *PR* genes in 2-week-old HLT seeding plants at 0, 4, 12, 24, 36 and 48 hpi with *Bgt* isolate E09. Expression was survey by qPCR with *ACTIN* as an endogenous control and calculated using the $2^{-\Delta\Delta C_T}$ method. Error bars indicate SEMs. Statistical significance was determined using Student's t test. The asterisks represent significance of differences (**, P < 0.01, and ***, P < 0.001, n=3, Students t-test). Each sample was analyzed with three replicates.

a

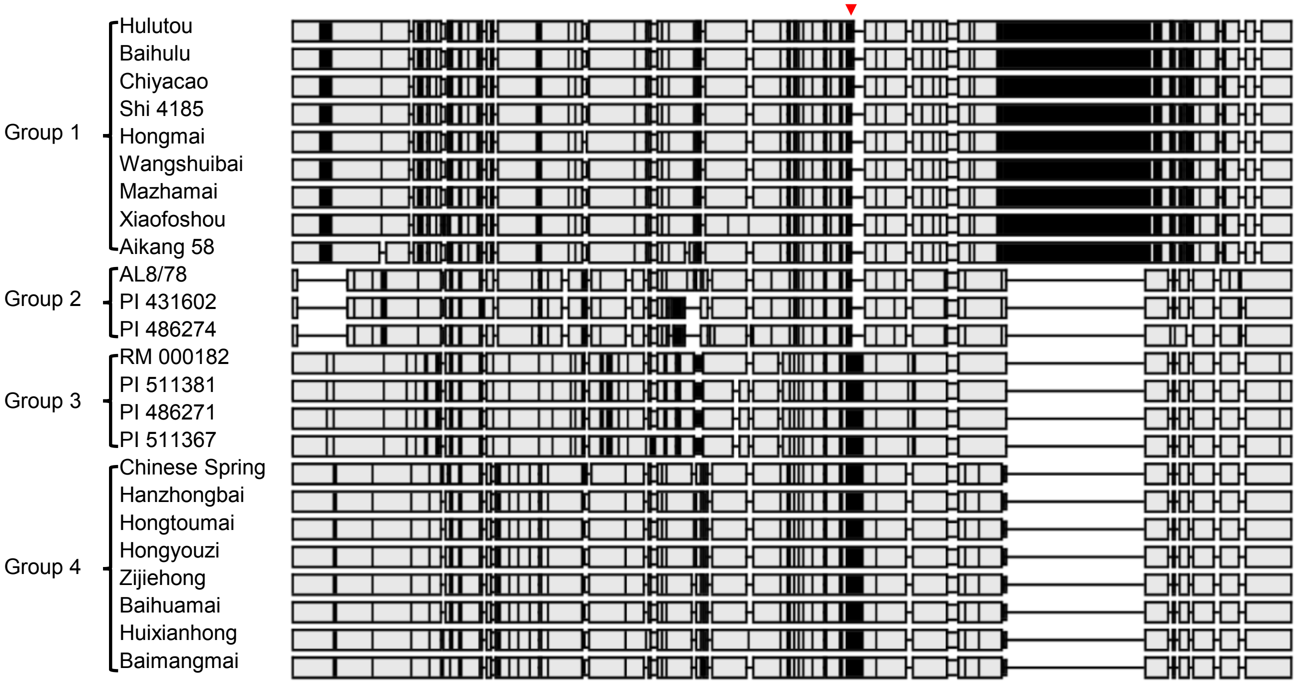

b

Entry \ Position	5610-5611	5635	5644	5652	5685	5781	5782	5784	5788	5790	5800	5802	5803	5804	5807	5808	5810	5812	5818	5861	5865	5873	5880-5881	5884	5906-5907	6028	6029	6057	NO.	Haplotype
HLT	G--A	C	G	C	T	C	G	A	A	G	T	T	T	G	C	T	A	A	G	G	G	T	G------T	T	TG	C	C	G	4	I
S4185	G--A	C	G	C	T	C	G	A	A	G	T	T	T	G	C	T	A	A	G	G	G	T	GAAAGGAT	T	TG	C	C	G	696	II
AL8/78	GATA	C	G	C	G	C	G	A	A	G	T	T	T	G	C	T	A	A	G	G	G	T	GAAAGGAT	T	TG	C	C	G	17	III
RM 000225	GATA	G	G	C	G	C	G	A	A	G	T	T	T	G	C	T	A	A	G	G	G	T	GAAAGGAT	T	TG	C	C	G	6	IV
DV 198	G--A	C	G	C	T	C	G	A	A	G	T	T	T	G	C	T	A	A	G	G	T	T	GAAAGGAT	T	TG	C	C	G	6	V
PI 554319	G--A	C	A	G	G	G	T	T	C	T	G	A	C	T	A	C	C	C	A	G	G	C	GAAAGGAT	A	T-114 bp -G	C	C	T	37	VI
Chinese Spring	G--A	C	A	G	G	G	T	T	C	T	G	A	C	T	A	C	C	C	A	A	G	C	GAAAGGAT	A	T-114 bp -G	C	C	T	243	VII
Richelle	G--A	C	A	G	G	G	T	G	C	T	G	A	C	T	A	C	C	C	A	A	G	C	GAAAGGAT	A	T-114 bp -G	C	C	T	33	VIII
Barbilla	G--A	C	A	G	G	G	T	T	C	T	G	A	C	T	A	C	C	C	A	A	G	C	GAAAGGAT	A	T-114 bp -G	C	A	T	25	IX
Trigo Obispado	G--A	C	A	G	G	G	T	T	C	T	G	A	C	T	A	C	C	C	A	A	G	C	GAAAGGAT	A	T-114 bp -G	T	A	T	2	X

Supplementary Figure 3 Haplotype characterization of *WTK3*

a, Sequence variants of the entire *WTK3* gene in 7 *Ae. tauschii* accessions 15 Chinese common wheat landraces, and two modern breeding cultivars S4185 and Aikang 58. Black lines and empty rectangle areas inside the rectangles indicate positions of SNPs and InDels, respectively. Red triangle indicates the 6 bp deletion located. **b,** Sequence alignment of InDel-WTK3 amplicons around the 6 bp critical deletion in the 1,069 wheat and *Ae. tauschii* accessions. The *WTK3* sequence of HLT was used as a reference.

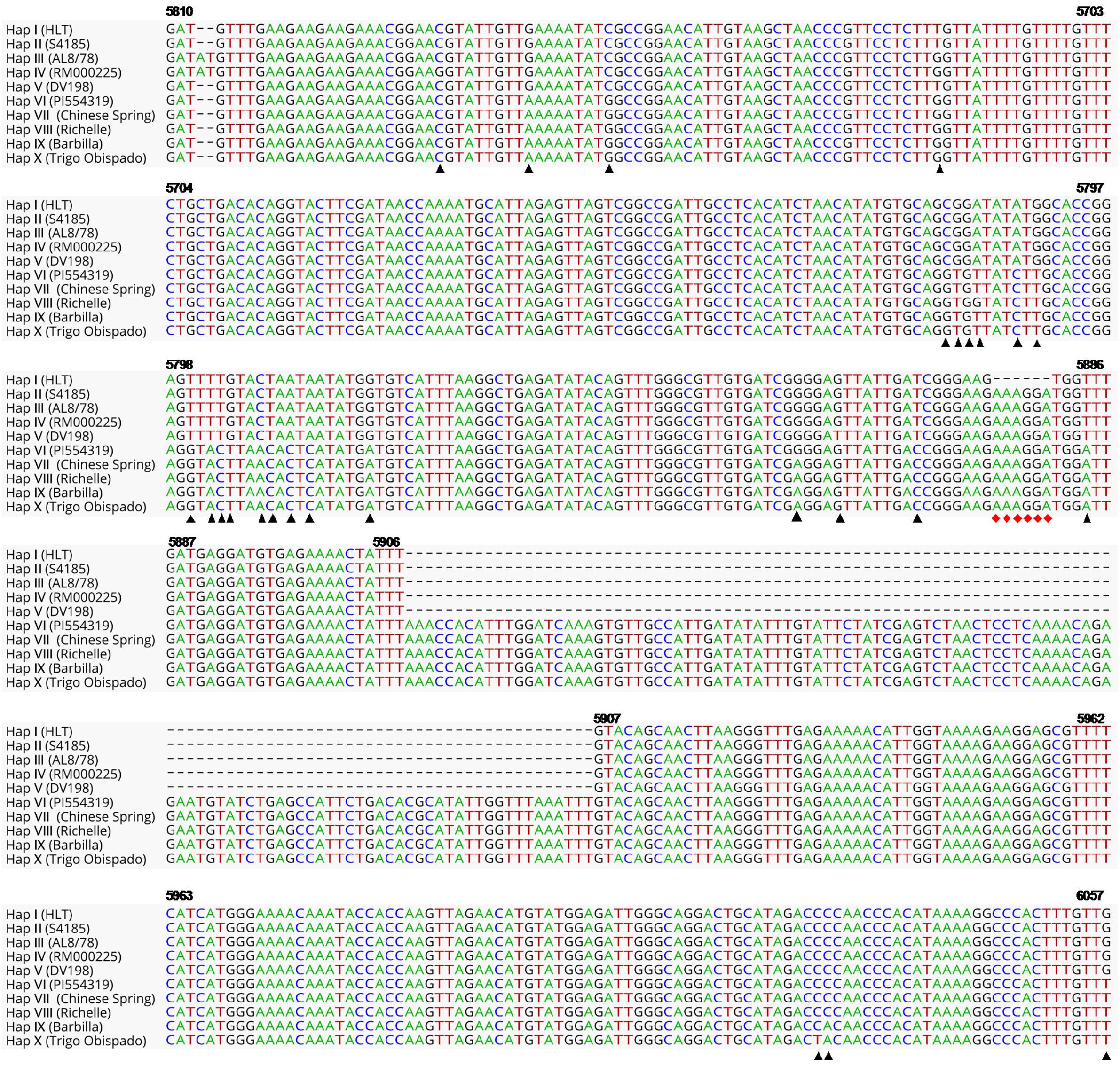

Supplementary Figure 4 Sequence variations in the 10 *WTK3* haplotypes of the 632 bp genomic region containing the 6-bp deletion in 1,069 common wheat and *Ae. tauschii* accessions. Labels: black triangle, SNP; red rhombus, 6-bp deletion.

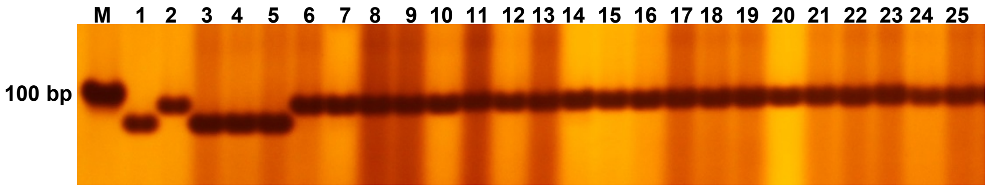

Supplementary Figure 5 Validation of sequence tagged site (STS) marker *STS-Pm24* for molecular marker-assisted selection (MAS) of *Pm24* in wheat.

M, 1 kb ladder; 1, HLT; 2, S4185; 3, CYC; 4, BHL; 5, Hongmangmai; 6, Chinese Spring; 7, Fuzhuang 30 (*Pm5e*); 8, Jiahongmai; 9, Youmangbaifu; 10, Mazhamai (*Pm5e*); 11, Xiaobaidong (*Pm5e*); 12, Hangzhongbai; 13, Zijiehong; 14, Baihuamai; 15, Hongyouzi; 16, Hongtoumai; 17, Pingyuan 50; 18, Wangshuibai; 19, Xiaofoshou; 20, Laozaomai (*Pm5e*); 21, Hongmai; 22, Baimangmai; 23, Huixianhong; 24, Sanyuehuang; 25, Huoliaomai.

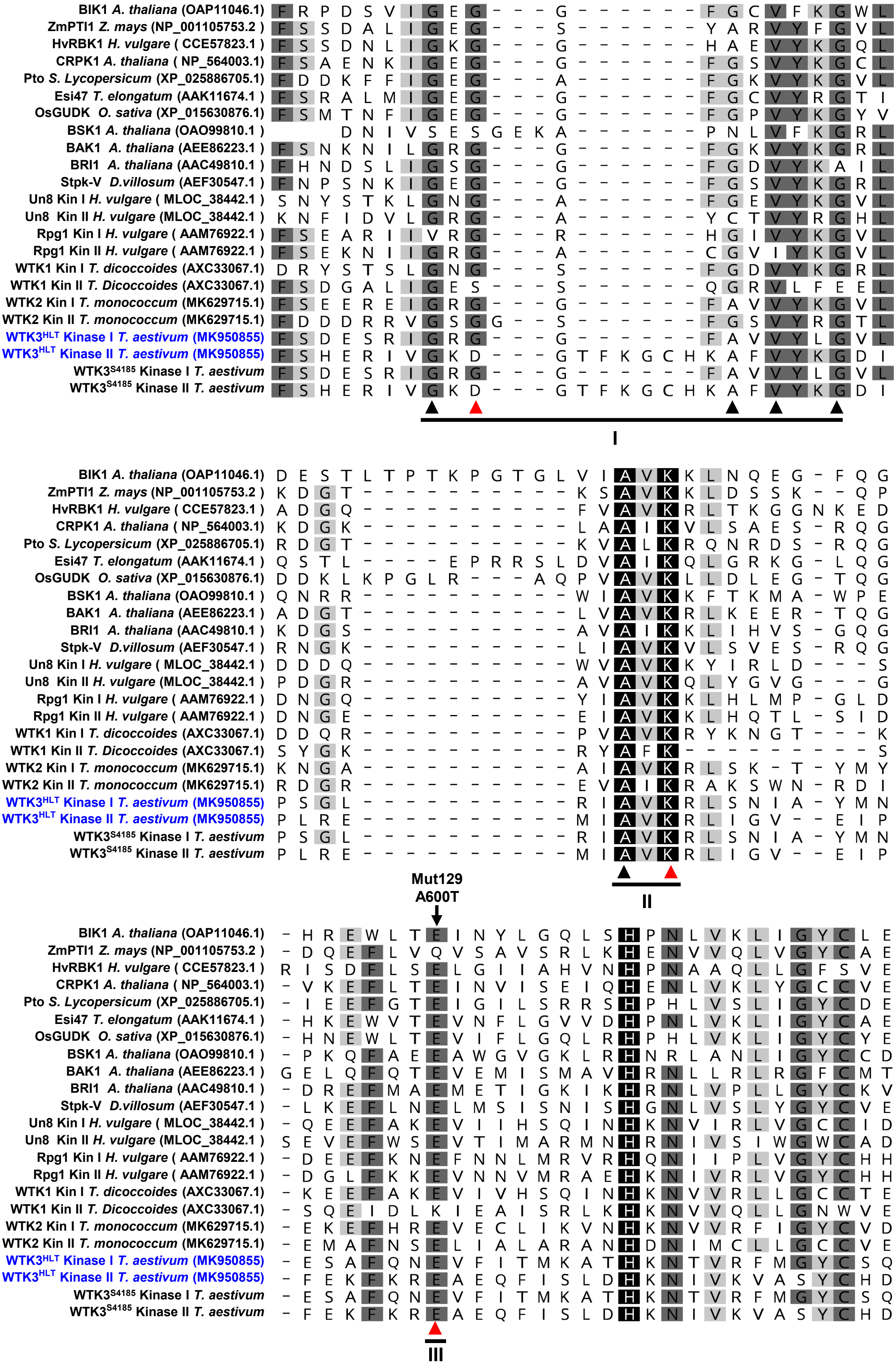
BIK1 A. thaliana (OAP11046.1)
ZmPTI1 Z. mays (NP_001105753.2)
HvRBK1 H. vulgare (CCE57823.1)
CRPK1 A. thaliana (NP_564003.1)
Pto S. Lycopersicum (XP_025886705.1)
Esi47 T. elongatum (AAK11674.1)
OsGUDK O. sativa (XP_015630876.1)
BSK1 A. thaliana (OAO99810.1)
BAK1 A. thaliana (AEE86223.1)
BRI1 A. thaliana (AAC49810.1)
Stpk-V D.villosum (AEF30547.1)
Un8 Kin I H. vulgare (MLOC_38442.1)
Un8 Kin II H. vulgare (MLOC_38442.1)
Rpg1 Kin I H. vulgare (AAM76922.1)
Rpg1 Kin II H. vulgare (AAM76922.1)
WTK1 Kin I T. dicoccoides (AXC33067.1)
WTK1 Kin II T. Dicoccoides (AXC33067.1)
WTK2 Kin I T. monococcum (MK629715.1)
WTK2 Kin II T. monococcum (MK629715.1)
WTK3HLT Kinase I T. aestivum (MK950855)
WTK3HLT Kinase II T. aestivum (MK950855)
WTK3S4185 Kinase I T. aestivum
WTK3S4185 Kinase II T. aestivum
I
II
Mut129
A600T
III

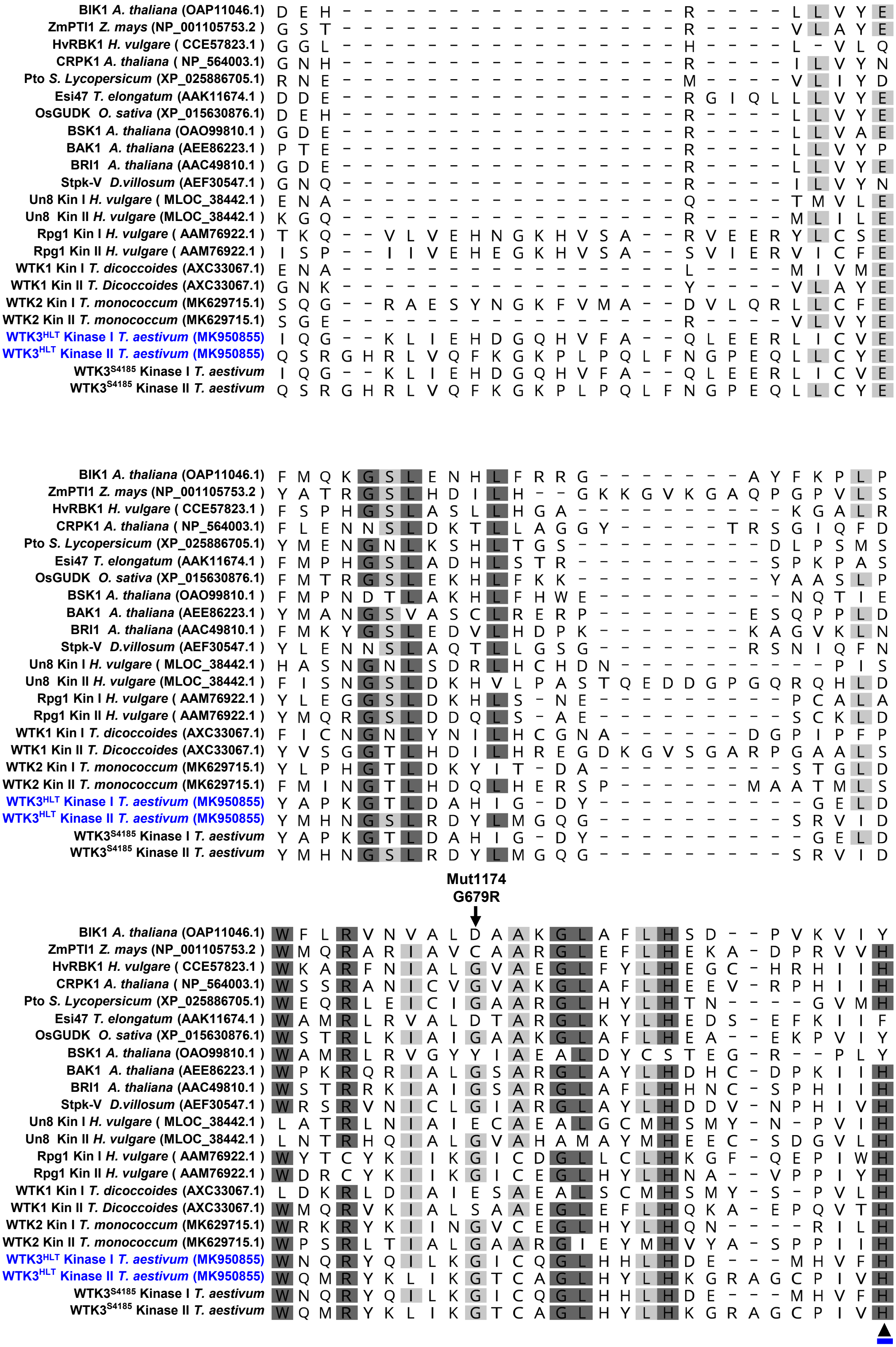
BIK1 A. thaliana (OAP11046.1) D E H - - - - - - - - - - - - - - - - R - - - - L L V Y E
ZmPTI1 Z. mays (NP_001105753.2) G S T - - - - - - - - - - - - - - - - R - - - - V L A Y E
HvRBK1 H. vulgare (CCE57823.1) G G L - - - - - - - - - - - - - - - - H - - - - L - V L Q
CRPK1 A. thaliana (NP_564003.1) G N H - - - - - - - - - - - - - - - - R - - - - I L V Y N
Pto S. Lycopersicum (XP_025886705.1) R N E - - - - - - - - - - - - - - - - M - - - - V L I Y D
Esi47 T. elongatum (AAK11674.1) D D E - - - - - - - - - - - - - - - - R G I Q L L L V Y E
OsGUDK O. sativa (XP_015630876.1) D E H - - - - - - - - - - - - - - - - R - - - - L L V Y E
BSK1 A. thaliana (OAO99810.1) G D E - - - - - - - - - - - - - - - - R - - - - L L V A E
BAK1 A. thaliana (AEE86223.1) P T E - - - - - - - - - - - - - - - - R - - - - L L V Y P
BRI1 A. thaliana (AAC49810.1) G D E - - - - - - - - - - - - - - - - R - - - - L L V Y E
Stpk-V D.villosum (AEF30547.1) G N Q - - - - - - - - - - - - - - - - R - - - - I L V Y N
Un8 Kin I H. vulgare (MLOC_38442.1) E N A - - - - - - - - - - - - - - - - Q - - - - T M V L E
Un8 Kin II H. vulgare (MLOC_38442.1) K G Q - - - - - - - - - - - - - - - - R - - - - M L I L E
Rpg1 Kin I H. vulgare (AAM76922.1) T K Q - - V L V E H N G K H V S A - - R V E E R Y L C S E
Rpg1 Kin II H. vulgare (AAM76922.1) I S P - - I I V E H E G K H V S A - - S V I E R V I C F E
WTK1 Kin I T. dicoccoides (AXC33067.1) E N A - - - - - - - - - - - - - - - - L - - - - M I V M E
WTK1 Kin II T. Dicoccoides (AXC33067.1) G N K - - - - - - - - - - - - - - - - Y - - - - V L A Y E
WTK2 Kin I T. monococcum (MK629715.1) S Q G - - R A E S Y N G K F V M A - - D V L Q R L L C F E
WTK2 Kin II T. monococcum (MK629715.1) S G E - - - - - - - - - - - - - - - - R - - - - V L V Y E
WTK3HLT Kinase I T. aestivum (MK950855) I Q G - - K L I E H D G Q H V F A - - Q L E E R L I C V E
WTK3HLT Kinase II T. aestivum (MK950855) Q S R G H R L V Q F K G K P L P Q L F N G P E Q L L C Y E
WTK3S4185 Kinase I T. aestivum I Q G - - K L I E H D G Q H V F A - - Q L E E R L I C V E
WTK3S4185 Kinase II T. aestivum Q S R G H R L V Q F K G K P L P Q L F N G P E Q L L C Y E
BIK1 A. thaliana (OAP11046.1) F M Q K G S L E N H L F R R G - - - - - - - A Y F K P L P
ZmPTI1 Z. mays (NP_001105753.2) Y A T R G S L H D I L H - - G K K G V K G A Q P G P V L S
HvRBK1 H. vulgare (CCE57823.1) F S P H G S L A S L L H G A - - - - - - - - - - K G A L R
CRPK1 A. thaliana (NP_564003.1) F L E N N S L D K T L L A G G Y - - - - - T R S G I Q F D
Pto S. Lycopersicum (XP_025886705.1) Y M E N G N L K S H L T G S - - - - - - - - - D L P S M S
Esi47 T. elongatum (AAK11674.1) F M P H G S L A D H L S T R - - - - - - - - - S P K P A S
OsGUDK O. sativa (XP_015630876.1) F M T R G S L E K H L F K K - - - - - - - - - Y A A S L P
BSK1 A. thaliana (OAO99810.1) F M P N D T L A K H L F H W E - - - - - - - - - N Q T I E
BAK1 A. thaliana (AEE86223.1) Y M A N G S V A S C L R E R P - - - - - - - E S Q P P L D
BRI1 A. thaliana (AAC49810.1) F M K Y G S L E D V L H D P K - - - - - - - K A G V K L N
Stpk-V D.villosum (AEF30547.1) Y L E N N S L A Q T L L G S G - - - - - - - R S N I Q F N
Un8 Kin I H. vulgare (MLOC_38442.1) H A S N G N L S D R L H C H D N - - - - - - - - - - P I S
Un8 Kin II H. vulgare (MLOC_38442.1) F I S N G S L D K H V L P A S T Q E D D G P G Q R Q H L D
Rpg1 Kin I H. vulgare (AAM76922.1) Y L E G G S L D K H L S - N E - - - - - - - - - P C A L A
Rpg1 Kin II H. vulgare (AAM76922.1) Y M Q R G S L D D Q L S - A E - - - - - - - - - S C K L D
WTK1 Kin I T. dicoccoides (AXC33067.1) F I C N G N L Y N I L H C G N A - - - - - - D G P I P F P
WTK1 Kin II T. Dicoccoides (AXC33067.1) Y V S G G T L H D I L H R E G D K G V S G A R P G A A L S
WTK2 Kin I T. monococcum (MK629715.1) Y L P H G T L D K Y I T - D A - - - - - - - - - S T G L D
WTK2 Kin II T. monococcum (MK629715.1) F M I N G T L H D Q L H E R S P - - - - - - M A A T M L S
WTK3HLT Kinase I T. aestivum (MK950855) Y A P K G T L D A H I G - D Y - - - - - - - - - - G E L D
WTK3HLT Kinase II T. aestivum (MK950855) Y M H N G S L R D Y L M G Q G - - - - - - - - - S R V I D
WTK3S4185 Kinase I T. aestivum Y A P K G T L D A H I G - D Y - - - - - - - - - - G E L D
WTK3S4185 Kinase II T. aestivum Y M H N G S L R D Y L M G Q G - - - - - - - - - S R V I D
Mut1174
G679R
BIK1 A. thaliana (OAP11046.1) W F L R V N V A L D A A K G L A F L H S D - - P V K V I Y
ZmPTI1 Z. mays (NP_001105753.2) W M Q R A R I A V C A A R G L E F L H E K A - D P R V V H
HvRBK1 H. vulgare (CCE57823.1) W K A R F N I A L G V A E G L F Y L H E G C - H R H I I H
CRPK1 A. thaliana (NP_564003.1) W S S R A N I C V G V A K G L A F L H E E V - R P H I I H
Pto S. Lycopersicum (XP_025886705.1) W E Q R L E I C I G A A R G L H Y L H T N - - - - G V M H
Esi47 T. elongatum (AAK11674.1) W A M R L R V A L D T A R G L K Y L H E D S - E F K I I F
OsGUDK O. sativa (XP_015630876.1) W S T R L K I A I G A A K G L A F L H E A - - E K P V I Y
BSK1 A. thaliana (OAO99810.1) W A M R L R V G Y Y I A E A L D Y C S T E G - R - - P L Y
BAK1 A. thaliana (AEE86223.1) W P K R Q R I A L G S A R G L A Y L H D H C - D P K I I H
BRI1 A. thaliana (AAC49810.1) W S T R R K I A I G S A R G L A F L H H N C - S P H I I H
Stpk-V D.villosum (AEF30547.1) W R S R V N I C L G I A R G L A Y L H D D V - N P H I V H
Un8 Kin I H. vulgare (MLOC_38442.1) L A T R L N I A I E C A E A L G C M H S M Y - N - P V I H
Un8 Kin II H. vulgare (MLOC_38442.1) L N T R H Q I A L G V A H A M A Y M H E E C - S D G V L H
Rpg1 Kin I H. vulgare (AAM76922.1) W Y T C Y K I I K G I C D G L L C L H K G F - Q E P I W H
Rpg1 Kin II H. vulgare (AAM76922.1) W D R C Y K I I K G I C E G L H Y L H N A - - V P P I Y H
WTK1 Kin I T. dicoccoides (AXC33067.1) L D K R L D I A I E S A E A L S C M H S M Y - S - P V L H
WTK1 Kin II T. Dicoccoides (AXC33067.1) W M Q R V K I A L S A A E G L E F L H Q K A - E P Q V T H
WTK2 Kin I T. monococcum (MK629715.1) W R K R Y K I I N G V C E G L H Y L H Q N - - - - R I L H
WTK2 Kin II T. monococcum (MK629715.1) W P S R L T I A L G A A R G I E Y M H V Y A - S P P I I H
WTK3HLT Kinase I T. aestivum (MK950855) W N Q R Y Q I L K G I C Q G L H H L H D E - - - M H V F H
WTK3HLT Kinase II T. aestivum (MK950855) W Q M R Y K L I K G T C A G L H Y L H K G R A G C P I V H
WTK3S4185 Kinase I T. aestivum W N Q R Y Q I L K G I C Q G L H H L H D E - - - M H V F H
WTK3S4185 Kinase II T. aestivum W Q M R Y K L I K G T C A G L H Y L H K G R A G C P I V H

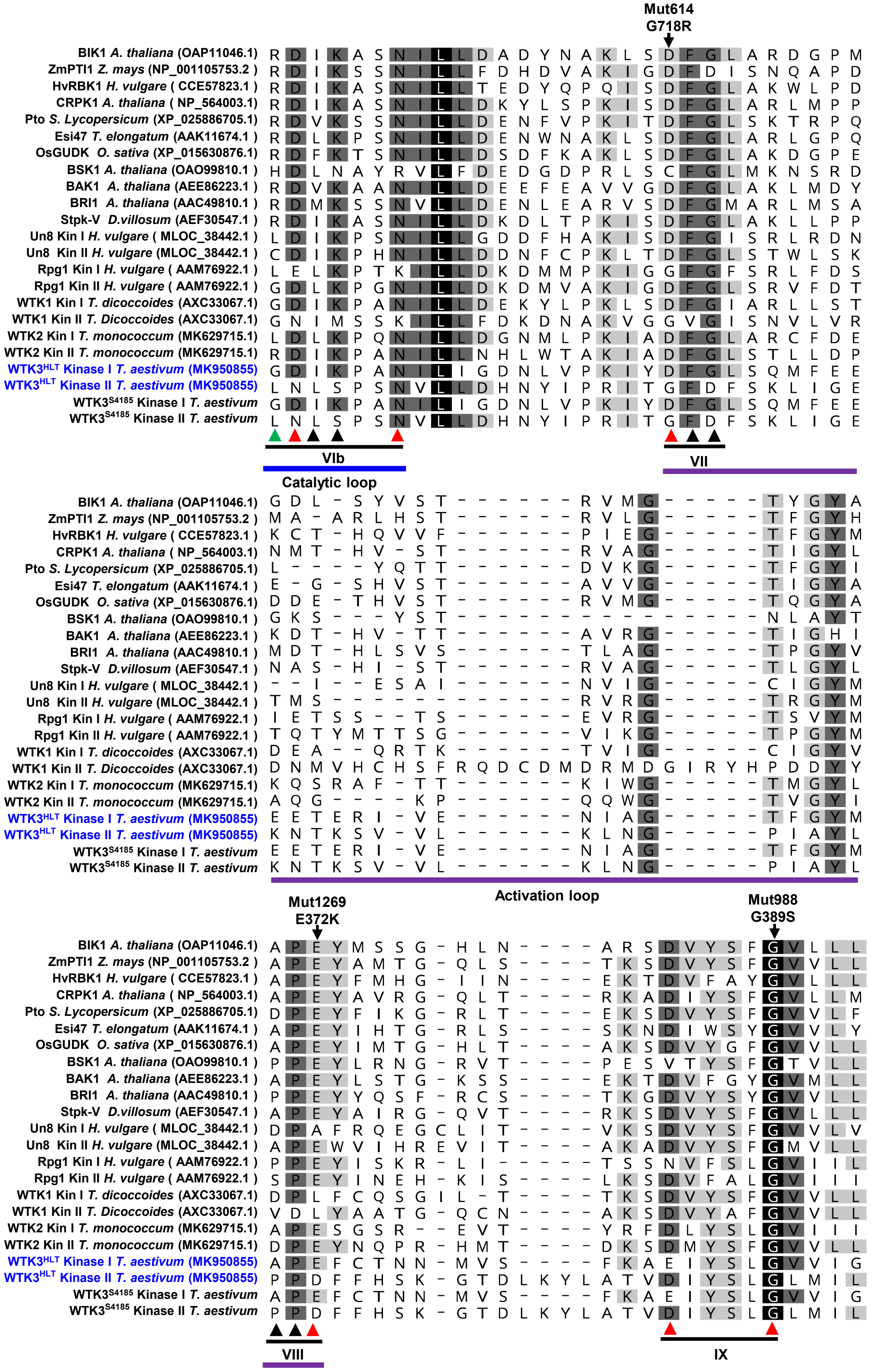
Mut614
G718R
BIK1 A. thaliana (OAP11046.1)
ZmPTI1 Z. mays (NP_001105753.2)
HvRBK1 H. vulgare (CCE57823.1)
CRPK1 A. thaliana (NP_564003.1)
Pto S. Lycopersicum (XP_025886705.1)
Esi47 T. elongatum (AAK11674.1)
OsGUDK O. sativa (XP_015630876.1)
BSK1 A. thaliana (OAO99810.1)
BAK1 A. thaliana (AEE86223.1)
BRI1 A. thaliana (AAC49810.1)
Stpk-V D.villosum (AEF30547.1)
Un8 Kin I H. vulgare (MLOC_38442.1)
Un8 Kin II H. vulgare (MLOC_38442.1)
Rpg1 Kin I H. vulgare (AAM76922.1)
Rpg1 Kin II H. vulgare (AAM76922.1)
WTK1 Kin I T. dicoccoides (AXC33067.1)
WTK1 Kin II T. Dicoccoides (AXC33067.1)
WTK2 Kin I T. monococcum (MK629715.1)
WTK2 Kin II T. monococcum (MK629715.1)
WTK3HLT Kinase I T. aestivum (MK950855)
WTK3HLT Kinase II T. aestivum (MK950855)
WTK3S4185 Kinase I T. aestivum
WTK3S4185 Kinase II T. aestivum
VIb
VII
Catalytic loop
Activation loop
Mut1269
E372K
Mut988
G389S
VIII
IX

**BIK1** ***A. thaliana*** **(OAP11046.1)**	EILSGKRALDHNRPAK-------------
**ZmPTI1** ***Z. mays*** **(NP_001105753.2)**	ELLTGRKPVDHTLPRG-------------
**HvRBK1** ***H. vulgare*** **(CCE57823.1)**	ELVTGRKAVDSSRQ---------------
**CRPK1** ***A. thaliana*** **(NP_564003.1)**	EIVSGRSNKN-TRLPTEYQYLL-------
**Pto** ***S. Lycopersicum*** **(XP_025886705.1)**	EVLCARSAMVQSLPRE-------------
**Esi47** ***T. elongatum*** **(AAK11674.1)**	ELLTGRRPLDRNRPRG-------------
**OsGUDK** ***O. sativa*** **(XP_015630876.1)**	ELLSGRKSVDKSRPAR-------------
**BSK1** ***A. thaliana*** **(OAO99810.1)**	DLLSGKHIPPSHAL---------------
**BAK1** ***A. thaliana*** **(AEE86223.1)**	ELITGQRAFDLARLANDDDVMLL------
**BRI1** ***A. thaliana*** **(AAC49810.1)**	ELLTGKRPTDSPDFG--------------
**Stpk-V** ***D.villosum*** **(AEF30547.1)**	EIVSGRSNTS-SRLPYEDQILLEKFPEVT
**Un8 Kin I** ***H. vulgare*** **(MLOC_38442.1)**	ELITKTKPTDETKRVI-------------
**Un8 Kin II** ***H. vulgare*** **(MLOC_38442.1)**	EVVSGRNYNFREESVGSEDWY--------
**Rpg1 Kin I** ***H. vulgare*** **(AAM76922.1)**	QIMAGKESYTKCVDIPPE--------EFT
**Rpg1 Kin II** ***H. vulgare*** **(AAM76922.1)**	KMMTGKEDYSNYAHTPRE--------EFI
**WTK1 Kin I** ***T. dicoccoides*** **(AXC33067.1)**	EMITRKKATDGATSLT-------------
**WTK1 Kin II** ***T. Dicoccoides*** **(AXC33067.1)**	ELLTGREAVDHALPKG-------------
**WTK2 Kin I** ***T. monococcum*** **(MK629715.1)**	EILTGKRGYEAVED---------------
**WTK2 Kin II** ***T. monococcum*** **(MK629715.1)**	ELLSGRRVVQQYPD------------SMT
**WTK3^{HLT} Kinase I** ***T. aestivum*** **(MK950855)**	ELLIGK--WFDEDV---------------
**WTK3^{HLT} Kinase II** ***T. aestivum*** **(MK950855)**	EIATQQEIKGIH-----G--------VLI
**WTK3^{S4185} Kinase I** ***T. aestivum***	ELLIGKKGWFDEDV---------------
**WTK3^{S4185} Kinase II** ***T. aestivum***	EIATQQEIKGIH-----G--------VLI

**BIK1** ***A. thaliana*** **(OAP11046.1)**	EENLVDWARPYL-----------------
**ZmPTI1** ***Z. mays*** **(NP_001105753.2)**	QQSLVTWATP-------------------
**HvRBK1** ***H. vulgare*** **(CCE57823.1)**	--SLVIWAKPLL-----------------
**CRPK1** ***A. thaliana*** **(NP_564003.1)**	-----ERAWELY-----------------
**Pto** ***S. Lycopersicum*** **(XP_025886705.1)**	MVNLAEWAVESH-----------------
**Esi47** ***T. elongatum*** **(AAK11674.1)**	EQNLVEWVKPYS-----------------
**OsGUDK** ***O. sativa*** **(XP_015630876.1)**	EQNLVEWARPYL-----------------
**BSK1** ***A. thaliana*** **(OAO99810.1)**	---------DM------------------
**BAK1** ***A. thaliana*** **(AEE86223.1)**	-----DWVKGLL-----------------
**BRI1** ***A. thaliana*** **(AAC49810.1)**	DNNLVGWVKQHA-----------------
**Stpk-V** ***D.villosum*** **(AEF30547.1)**	NGVLLLQTWMYY-----------------
**Un8 Kin I** ***H. vulgare*** **(MLOC_38442.1)**	----QRFGKAF------------------
**Un8 Kin II** ***H. vulgare*** **(MLOC_38442.1)**	---FPKWVYEKC-----------------
**Rpg1 Kin I** ***H. vulgare*** **(AAM76922.1)**	ERVYGFWVNRMP-----------------
**Rpg1 Kin II** ***H. vulgare*** **(AAM76922.1)**	EHVCKKWQVRLH-----------------
**WTK1 Kin I** ***T. dicoccoides*** **(AXC33067.1)**	----QCFAEAL------------------
**WTK1 Kin II** ***T. Dicoccoides*** **(AXC33067.1)**	KQSLVTWVYNHGEEKSPWRWENNIMEDNV
**WTK2 Kin I** ***T. monococcum*** **(MK629715.1)**	--ILQSWSNRL------------------
**WTK2 Kin II** ***T. monococcum*** **(MK629715.1)**	PKNVVDFAVPHI-----------------
**WTK3^{HLT} Kinase I** ***T. aestivum*** **(MK950855)**	---RKLFVQQLK-----------------
**WTK3^{HLT} Kinase II** ***T. aestivum*** **(MK950855)**	KSIEENWREESQ-----------------
**WTK3^{S4185} Kinase I** ***T. aestivum***	---RKLFVQQLK-----------------
**WTK3^{S4185} Kinase II** ***T. aestivum***	KSIEENWREESQ-----------------

Mut410
W429*

**BIK1** ***A. thaliana*** **(OAP11046.1)**	-----TSKRKVLLIVDNRLDTQY--LPEE
**ZmPTI1** ***Z. mays*** **(NP_001105753.2)**	----RLSEDKVRQCVDPRLGDEY--PPKA
**HvRBK1** ***H. vulgare*** **(CCE57823.1)**	------ESNNMKGLVDPSLDAGY--DLEE
**CRPK1** ***A. thaliana*** **(NP_564003.1)**	------ERNELVDLVDSGLNGVF--DAEE
**Pto** ***S. Lycopersicum*** **(XP_025886705.1)**	------NNGQLEQIVDPNLADKI--RPES
**Esi47** ***T. elongatum*** **(AAK11674.1)**	-----SDTKKFETIMDPRLEGNY--NLKS
**OsGUDK** ***O. sativa*** **(XP_015630876.1)**	-----TDARRLGRVMDRNLAGQY--PAKA
**BSK1** ***A. thaliana*** **(OAO99810.1)**	-----IRGKNIILLMDSHLEGKF--STEE
**BAK1** ***A. thaliana*** **(AEE86223.1)**	------KEKKLEALVDVDLQGNY--KDEE
**BRI1** ***A. thaliana*** **(AAC49810.1)**	------K-LRISDVFDPELMKEDPALEIE
**Stpk-V** ***D.villosum*** **(AEF30547.1)**	------EQGDLVKIIDSSVGDDL--DIEQ
**Un8 Kin I** ***H. vulgare*** **(MLOC_38442.1)**	-----TKGRPVRDLFDADIANKS--NTKV
**Un8 Kin II** ***H. vulgare*** **(MLOC_38442.1)**	-----YIHHKIEDILDPILQAEACQGTGI
**Rpg1 Kin I** ***H. vulgare*** **(AAM76922.1)**	-----GT--------------VSKHTSNE
**Rpg1 Kin II** ***H. vulgare*** **(AAM76922.1)**	-----AT--------------MRSHVFEE
**WTK1 Kin I** ***T. dicoccoides*** **(AXC33067.1)**	------GGKKVRQLFDVEIANDK-KKVKL
**WTK1 Kin II** ***T. Dicoccoides*** **(AXC33067.1)**	LTKTSFSEDMVQRCVDPRLKGYY--HRSA
**WTK2 Kin I** ***T. monococcum*** **(MK629715.1)**	---------------DES---QRDIQLEQ
**WTK2 Kin II** ***T. monococcum*** **(MK629715.1)**	------LADDVMRVLDPRLPTPTSHEAEA
**WTK3^{HLT} Kinase I** ***T. aestivum*** **(MK950855)**	-----GLRKTLVKEGAFS---SWENKYHQ
**WTK3^{HLT} Kinase II** ***T. aestivum*** **(MK950855)**	-----ITR------LYTS---LGADELRQ
**WTK3^{S4185} Kinase I** ***T. aestivum***	-----GLRKTLVKEGAFS---SWENKYHQ
**WTK3^{S4185} Kinase II** ***T. aestivum***	-----ITR------LYTS---LGADELRQ

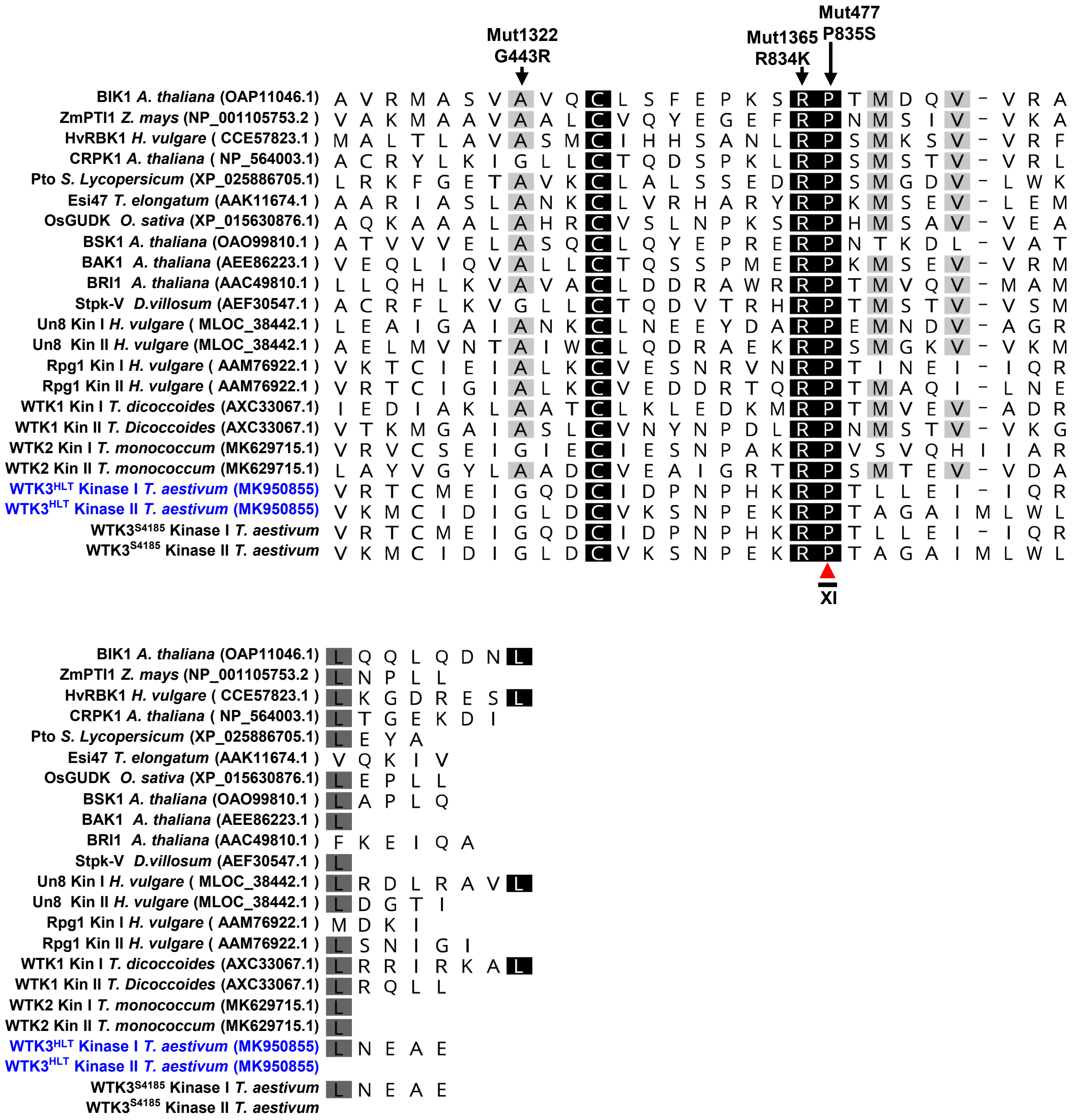

Supplementary Figure 6 Kinase domain comparison analysis of Pm24.

Multiple alignments of 15 RLCK[30-38] protein kinase domains were used to infer the architecture of WTK3. The sequence of WTK3 (Pm24) protein was similar to WTK1 (Yr15[35]). The WTK3 Kin I (row 20) is classified as a non-RD kinase and a serine/threonine protein kinase. The WTK3 Kin II (row 21) is a putative pseudokinase. Labels: black triangles, ATP binding site; red triangles, key conserved residues; green triangles positions within the catalytic loop that determine whether a kinase belongs to the RD or non-RD group. Conserved subdomains I, II, III, VIb, VII, VIII, IX, and XI are highlighted by black bars labeled with Roman numerals. The catalytic loop and the activation loop are highlighted by blue and purple bars, respectively. Blue rhombi highlight the rare 2 amino acid functional deletion in the WTK3^{S4185} Kin I domain that is associated with powdery mildew resistance of the WTK3^{HLT} (Pm24). EMS missense mutations resulted in loss of *Pm24* function are indicated by black arrows.

	1 10 20 30 40 50 60 70 80 90
WTK3^{S4185}	ATGGGCGGATACGAGTTCCAGAGGGCGGAGCTAGATGCACTGGAAGGCGTCGTACGCGATCCAACTGCGGAGCCAATGAGTCTGACGTTGCCGCTT
**WTK3**^{S4185}	M G G Y E F Q R A E L D A L E G V V R D P T A E P M S L T L P L
WTK3^{HLT}	ATGGGCGGATACGAGTTCCAGAGGGCGGAGCTAGATGCACTGGAAGGCGTCGTACGCGATCCAACTGCGGAGCCAATGAGTCTGACGTTGCCGCTT
**WTK3**^{HLT}	M G G Y E F Q R A E L D A L E G V V R D P T A E P M S L T L P L
WTK3#1	ATGGGCGGATACGAGTTCCAGAGGGCGGAGCTAGATGCACTGGAAGGCGTCGTACGCGATCCAACTGCGGAGCCAATGAGTCTGACGTTGCCGCTT
**WTK3#1**	M G G Y E F Q R A E L D A L E G V V R D P T A E P M S L T L P L
WTK3#2	ATGGGCGGATACGAGTTCCAGAGGGCGGAGCTAGATGCACTGGAAGGCGTCGTACGCGATCCAACTGCGGAGCCAATGAGTCTGACGTTGCCGCTT
**WTK3#2**	M G G Y E F Q R A E L D A L E G V V R D P T A E P M S L T L P L
WTK3#3	ATGGGCGGATACGAGTTCCAGAGGGCGGAGCTAGATGCACTGGAAGGCGTCGTACGCGATCCAACTGCGGAGCCAATGAGTCTGACGTTGCCGCTT
**WTK3#3**	M G G Y E F Q R A E L D A L E G V V R D P T A E P M S L T L P L
	100 110 120 130 140 150 160 170 180 190
WTK3^{S4185}	CTCAGGCACATAACAAATGATTTCTCCCCTGAATTTGAAATTAGTAAAGATGATTCTGCAGTGGTTTACCTGGGGGTGCTTCCAAGTGGGTTCCGT
**WTK3**^{S4185}	L R H I T N D F S P E F E I S K D D S A V V Y L G V L P S G F R
WTK3^{HLT}	CTCAGGCACATAACAAATGATTTCTCCCCTGAATTTGAAATTAGTAAAGATGATTCTGCAGTGGTTTACCTGGGGGTGCTTCCAAGTGGGTTCCGT
**WTK3**^{HLT}	L R H I T N D F S P E F E I S K D D S A V V Y L G V L P S G F R
WTK3#1	CTCAGGCACATAACAAATGATTTCTCCCCTGAATTTGAAATTAGTAAAGATGATTCTGCAGTGGTTTACCTGGGGGTGCTTCCAAGTGGGTTCCGT
**WTK3#1**	L R H I T N D F S P E F E I S K D D S A V V Y L G V L P S G F R
WTK3#2	CTCAGGCACATAACAAATGATTTCTCCCCTGAATTTGAAATTAGTAAAGATGATTCTGCAGTGGTTTACCTGGGGGTGCTTCCAAGTGGGTTCCGT
**WTK3#2**	L R H I T N D F S P E F E I S K D D S A V V Y L G V L P S G F R
WTK3#3	CTCAGGCACATAACAAATGATTTCTCCCCTGAATTTGAAATTAGTAAAGATGATTCTGCAGTGGTTTACCTGGGGGTGCTTCCAAGTGGGTTCCGT
**WTK3#3**	L R H I T N D F S P E F E I S K D D S A V V Y L G V L P S G F R
	200 210 220 230 240 250 260 270 280
WTK3^{S4185}	GTTGCTGTCAAGAAGTCTCACTTTCGTTTTTGCTTGGATGATGAAGATGCATTCACAAATGAAGTTTCTATTGCAATGAAGGCTGCTCATAAGAAC
**WTK3**^{S4185}	V A V K K S H F R F C L D D E D A F T N E V S I A M K A A H K N
WTK3^{HLT}	GTTGCTGTCAAGAAGTCTCACTTTCGTTTTTGCTTGGATGATGAAGATGCATTCACAAATGAAGTTTCTATTGCAATGAAGGCTGCTCATAAGAAC
**WTK3**^{HLT}	V A V K K S H F R F C L D D E D A F T N E V S I A M K A A H K N
WTK3#1	GTTGCTGTCAAGAAGTCTCACTTTCGTTTTTGCTTGGATGATGAAGATGCATTCACAAATGAAGTTTCTATTGCAATGAAGGCTGCTCATAAGAAC
**WTK3#1**	V A V K K S H F R F C L D D E D A F T N E V S I A M K A A H K N
WTK3#2	GTTGCTGTCAAGAAGTCTCACTTTCGTTTTTGCTTGGATGATGAAGATGCATTCACAAATGAAGTTTCTATTGCAATGAAGGCTGCTCATAAGAAC
**WTK3#2**	V A V K K S H F R F C L D D E D A F T N E V S I A M K A A H K N
WTK3#3	GTTGCTGTCAAGAAGTCTCACTTTCGTTTTTGCTTGGATGATGAAGATGCATTCACAAATGAAGTTTCTATTGCAATGAAGGCTGCTCATAAGAAC
**WTK3#3**	V A V K K S H F R F C L D D E D A F T N E V S I A M K A A H K N
	290 300 310 320 330 340 350 360 370 380
WTK3^{S4185}	ACAGTGCGAGTCATAGGCTACTGTCATCACACGCATGAGCAAATTGCCGAATACGAAGGAAAACAAGTTTTCGCAGAGGTCAGAGAAAGGTTGATC
**WTK3**^{S4185}	T V R V I G Y C H H T H E Q I A E Y E G K Q V F A E V R E R L I
WTK3^{HLT}	ACAGTGCGAGTCATAGGCTACTGTCATCACACGCATGAGCAAATTGCCGAATACGAAGGAAAACAAGTTTTCGCAGAGGTCAGAGAAAGGTTGATC
**WTK3**^{HLT}	T V R V I G Y C H H T H E Q I A E Y E G K Q V F A E V R E R L I
WTK3#1	ACAGTGCGAGTCATAGGCTACTGTCATCACACGCATGAGCAAATTGCCGAATACGAAGGAAAACAAGTTTTCGCAGAGGTCAGAGAAAGGTTGATC
**WTK3#1**	T V R V I G Y C H H T H E Q I A E Y E G K Q V F A E V R E R L I
WTK3#2	ACAGTGCGAGTCATAGGCTACTGTCATCACACGCATGAGCAAATTGCCGAATACGAAGGAAAACAAGTTTTCGCAGAGGTCAGAGAAAGGTTGATC
**WTK3#2**	T V R V I G Y C H H T H E Q I A E Y E G K Q V F A E V R E R L I
WTK3#3	ACAGTGCGAGTCATAGGCTACTGTCATCACACGCATGAGCAAATTGCCGAATACGAAGGAAAACAAGTTTTCGCAGAGGTCAGAGAAAGGTTGATC
**WTK3#3**	T V R V I G Y C H H T H E Q I A E Y E G K Q V F A E V R E R L I
	390 400 410 420 430 440 450 460 470 480
WTK3^{S4185}	TGTACCGAGTATGTGCCTAACGGACCCCTTAGTGGACATATCGAAGGTAAGATATGTGCGCAAATGGATGGATACGAGTTCCAGAGGGCAGAACTA
**WTK3**^{S4185}	C T E Y V P N G P L S G H I E G K I C A Q M D G Y E F Q R A E L
WTK3^{HLT}	TGTACCGAGTATGTGCCTAACGGACCCCTTAGTGGACATATCGAAGGTAAGATATGTGCGCAAATGGATGGATACGAGTTCCAGAGGGCAGAACTA
**WTK3**^{HLT}	C T E Y V P N G P L S G H I E G K I C A Q M D G Y E F Q R A E L
WTK3#1	TGTACCGAGTATGTGCCTAACGGACCCCTTAGTGGACATATCGAAGGTAAGATATGTGCGCAAATGGATGGATACGAGTTCCAGAGGGCAGAACTA
**WTK3#1**	C T E Y V P N G P L S G H I E G K I C A Q M D G Y E F Q R A E L
WTK3#2	TGTACCGAGTATGTGCCTAACGGACCCCTTAGTGGACATATCGAAGGTAAGATATGTGCGCAAATGGATGGATACGAGTTCCAGAGGGCAGAACTA
**WTK3#2**	C T E Y V P N G P L S G H I E G K I C A Q M D G Y E F Q R A E L
WTK3#3	TGTACCGAGTATGTGCCTAACGGACCCCTTAGTGGACATATCGAAGGTAAGATATGTGCGCAAATGGATGGATACGAGTTCCAGAGGGCAGAACTA
**WTK3#3**	C T E Y V P N G P L S G H I E G K I C A Q M D G Y E F Q R A E L
	490 500 510 520 530 540 550 560 570
WTK3^{S4185}	GATGCACTAGAACGCGTCGTACGCGATACAAGTGCGGAGCCAATGAGTCTGACGTTGCCGCTTCTCAGGCACATAACAAATGATTTCTCCGATGAA
**WTK3**^{S4185}	D A L E R V V R D T S A E P M S L T L P L L R H I T N D F S D E
WTK3^{HLT}	GATGCACTAGAACGCGTCGTACGCGATACAAGTGCGGAGCCAATGAGTCTGACGTTGCCGCTTCTCAGGCACATAACAAATGATTTCTCCGATGAA
**WTK3**^{HLT}	D A L E R V V R D T S A E P M S L T L P L L R H I T N D F S D E
WTK3#1	GATGCACTAGAACGCGTCGTACGCGATACAAGTGCGGAGCCAATGAGTCTGACGTTGCCGCTTCTCAGGCACATAACAAATGATTTCTCCGATGAA
**WTK3#1**	D A L E R V V R D T S A E P M S L T L P L L R H I T N D F S D E
WTK3#2	GATGCACTAGAACGCGTCGTACGCGATACAAGTGCGGAGCCAATGAGTCTGACGTTGCCGCTTCTCAGGCACATAACAAATGATTTCTCCGATGAA
**WTK3#2**	D A L E R V V R D T S A E P M S L T L P L L R H I T N D F S D E
WTK3#3	GATGCACTAGAACGCGTCGTACGCGATACAAGTGCGGAGCCAATGAGTCTGACGTTGCCGCTTCTCAGGCACATAACAAATGATTTCTCCGATGAA
**WTK3#3**	D A L E R V V R D T S A E P M S L T L P L L R H I T N D F S D E
	580 590 600 610 620 630 640 650 660 670
WTK3^{S4185}	TCTCGAATTGGCCGAGGTGGATTCGCAGTGGTTTACCTGGGGGTGCTTCCAAGTGGGTTACGTATTGCTGTTAAGAGGCTTAGCAATATTGCTTAT
**WTK3**^{S4185}	S R I G R G G F A V V Y L G V L P S G L R I A V K R L S N I A Y
WTK3^{HLT}	TCTCGAATTGGCCGAGGTGGATTCGCAGTGGTTTACCTGGGGGTGCTTCCAAGTGGGTTACGTATTGCTGTTAAGAGGCTTAGCAATATTGCTTAT
**WTK3**^{HLT}	S R I G R G G F A V V Y L G V L P S G L R I A V K R L S N I A Y
WTK3#1	TCTCGAATTGGCCGAGGTGGATTCGCAGTGGTTTACCTGGGGGTGCTTCCAAGTGGGTTACGTATTGCTGTTAAGAGGCTTAGCAATATTGCTTAT
**WTK3#1**	S R I G R G G F A V V Y L G V L P S G L R I A V K R L S N I A Y
WTK3#2	TCTCGAATTGGCCGAGGTGGATTCGCAGTGGTTTACCTGGGGGTGCTTCCAAGTGGGTTACGTATTGCTGTTAAGAGGCTTAGCAATATTGCTTAT
**WTK3#2**	S R I G R G G F A V V Y L G V L P S G L R I A V K R L S N I A Y
WTK3#3	TCTCGAATTGGCCGAGGTGGATTCGCAGTGGTTTACCTGGGGGTGCTTCCAAGTGGGTTACGTATTGCTGTTAAGAGGCTTAGCAATATTGCTTAT
**WTK3#3**	S R I G R G G F A V V Y L G V L P S G L R I A V K R L S N I A Y

```
              680       690       700       710       720       730       740       750       760
WTK3^S4185 ATGAACGAAAGTGCATTTCAAAATGAAGTGTTTATCACAATGAAGGCCACTCACAAGAACACAGTGCGATTCATGGGCTACTGTAGTCAAATACAA
WTK3^S4185  M  N  E  S  A  F  Q  N  E  V  F  I  T  M  K  A  T  H  K  N  T  V  R  F  M  G  Y  C  S  Q  I  Q
WTK3^HLT   ATGAACGAAAGTGCATTTCAAAATGAAGTGTTTATCACAATGAAGGCCACTCACAAGAACACAGTGCGATTCATGGGCTACTGTAGTCAAATACAA
WTK3^HLT    M  N  E  S  A  F  Q  N  E  V  F  I  T  M  K  A  T  H  K  N  T  V  R  F  M  G  Y  C  S  Q  I  Q
WTK3#1     ATGAACGAAAGTGCATTTCAAAATGAAGTGTTTATCACAATGAAGGCCACTCACAAGAACACAGTGCGATTCATGGGCTACTGTAGTCAAATACAA
WTK3#1      M  N  E  S  A  F  Q  N  E  V  F  I  T  M  K  A  T  H  K  N  T  V  R  F  M  G  Y  C  S  Q  I  Q
WTK3#2     ATGAACGAAAGTGCATTTCAAAATGAAGTGTTTATCACAATGAAGGCCACTCACAAGAACACAGTGCGATTCATGGGCTACTGTAGTCAAATACAA
WTK3#2      M  N  E  S  A  F  Q  N  E  V  F  I  T  M  K  A  T  H  K  N  T  V  R  F  M  G  Y  C  S  Q  I  Q
WTK3#3     ATGAACGAAAGTGCATTTCAAAATGAAGTGTTTATCACAATGAAGGCCACTCACAAGAACACAGTGCGATTCATGGGCTACTGTAGTCAAATACAA
WTK3#3      M  N  E  S  A  F  Q  N  E  V  F  I  T  M  K  A  T  H  K  N  T  V  R  F  M  G  Y  C  S  Q  I  Q

            770       780       790       800       810       820       830       840       850       860
WTK3^S4185 GGTAAACTCATCGAACACGACGGGCAACATGTTTTCGCACAGCTCGAGGAAAGGTTGATCTGTGTGGAATATGCGCCTAAAGGAACCCTTGATGCA
WTK3^S4185  G  K  L  I  E  H  D  G  Q  H  V  F  A  Q  L  E  E  R  L  I  C  V  E  Y  A  P  K  G  T  L  D  A
WTK3^HLT   GGTAAACTCATCGAACACGACGGGCAACATGTTTTCGCACAGCTCGAGGAAAGGTTGATCTGTGTGGAATATGCGCCTAAAGGAACCCTTGATGCA
WTK3^HLT    G  K  L  I  E  H  D  G  Q  H  V  F  A  Q  L  E  E  R  L  I  C  V  E  Y  A  P  K  G  T  L  D  A
WTK3#1     GGTAAACTCATCGAACACGACGGGCAACATGTTTTCGCACAGCTCGAGGAAAGGTTGATCTGTGTGGAATATGCGCCTAAAGGAACCCTTGATGCA
WTK3#1      G  K  L  I  E  H  D  G  Q  H  V  F  A  Q  L  E  E  R  L  I  C  V  E  Y  A  P  K  G  T  L  D  A
WTK3#2     GGTAAACTCATCGAACACGACGGGCAACATGTTTTCGCACAGCTCGAGGAAAGGTTGATCTGTGTGGAATATGCGCCTAAAGGAACCCTTGATGCA
WTK3#2      G  K  L  I  E  H  D  G  Q  H  V  F  A  Q  L  E  E  R  L  I  C  V  E  Y  A  P  K  G  T  L  D  A
WTK3#3     GGTAAACTCATCGAACACGACGGGCAACATGTTTTCGCACAGCTCGAGGAAAGGTTGATCTGTGTGGAATATGCGCCTAAAGGAACCCTTGATGCA
WTK3#3      G  K  L  I  E  H  D  G  Q  H  V  F  A  Q  L  E  E  R  L  I  C  V  E  Y  A  P  K  G  T  L  D  A

                870       880       890       900       910       920       930       940       950       960
WTK3^S4185 CATATCGGTGACTATGGTGAACTTGACTGGAACCAGCGTTATCAAATTCTAAAAGGAATTTGTCAAGGTTTGCATCATCTCCATGACGAAATGCAC
WTK3^S4185  H  I  G  D  Y  G  E  L  D  W  N  Q  R  Y  Q  I  L  K  G  I  C  Q  G  L  H  H  L  H  D  E  M  H
WTK3^HLT   CATATCGGTGACTATGGTGAACTTGACTGGAACCAGCGTTATCAAATTCTAAAAGGAATTTGTCAAGGTTTGCATCATCTCCATGACGAAATGCAC
WTK3^HLT    H  I  G  D  Y  G  E  L  D  W  N  Q  R  Y  Q  I  L  K  G  I  C  Q  G  L  H  H  L  H  D  E  M  H
WTK3#1     CATATCGGTGACTATGGTGAACTTGACTGGAACCAGCGTTATCAAATTCTAAAAGGAATTTGTCAAGGTTTGCATCATCTCCATGACGAAATGCAC
WTK3#1      H  I  G  D  Y  G  E  L  D  W  N  Q  R  Y  Q  I  L  K  G  I  C  Q  G  L  H  H  L  H  D  E  M  H
WTK3#2     CATATCGGTGACTATGGTGAACTTGACTGGAACCAGCGTTATCAAATTCTAAAAGGAATTTGTCAAGGTTTGCATCATCTCCATGACGAAATGCAC
WTK3#2      H  I  G  D  Y  G  E  L  D  W  N  Q  R  Y  Q  I  L  K  G  I  C  Q  G  L  H  H  L  H  D  E  M  H
WTK3#3     CATATCGGTGACTATGGTGAACTTGACTGGAACCAGCGTTATCAAATTCTAAAAGGAATTTGTCAAGGTTTGCATCATCTCCATGACGAAATGCAC
WTK3#3      H  I  G  D  Y  G  E  L  D  W  N  Q  R  Y  Q  I  L  K  G  I  C  Q  G  L  H  H  L  H  D  E  M  H

                    970       980       990     1,000     1,010     1,020     1,030     1,040     1,050
WTK3^S4185 GTTTTTCATGGAGATATCAAACCAGCCAATATATTAATAGGGGATAACCTTGTGCCTAAAATCTATGACTTCGGTCTCTCCCAGATGTTTGAAGAA
WTK3^S4185  V  F  H  G  D  I  K  P  A  N  I  L  I  G  D  N  L  V  P  K  I  Y  D  F  G  L  S  Q  M  F  E  E
WTK3^HLT   GTTTTTCATGGAGATATCAAACCAGCCAATATATTAATAGGGGATAACCTTGTGCCTAAAATCTATGACTTCGGTCTCTCCCAGATGTTTGAAGAA
WTK3^HLT    V  F  H  G  D  I  K  P  A  N  I  L  I  G  D  N  L  V  P  K  I  Y  D  F  G  L  S  Q  M  F  E  E
WTK3#1     GTTTTTCATGGAGATATCAAACCAGCCAATATATTAATAGGGGATAACCTTGTGCCTAAAATCTATGACTTCGGTCTCTCCCAGATGTTTGAAGAA
WTK3#1      V  F  H  G  D  I  K  P  A  N  I  L  I  G  D  N  L  V  P  K  I  Y  D  F  G  L  S  Q  M  F  E  E
WTK3#2     GTTTTTCATGGAGATATCAAACCAGCCAATATATTAATAGGGGATAACCTTGTGCCTAAAATCTATGACTTCGGTCTCTCCCAGATGTTTGAAGAA
WTK3#2      V  F  H  G  D  I  K  P  A  N  I  L  I  G  D  N  L  V  P  K  I  Y  D  F  G  L  S  Q  M  F  E  E
WTK3#3     GTTTTTCATGGAGATATCAAACCAGCCAATATATTAATAGGGGATAACCTTGTGCCTAAAATCTATGACTTCGGTCTCTCCCAGATGTTTGAAGAA
WTK3#3      V  F  H  G  D  I  K  P  A  N  I  L  I  G  D  N  L  V  P  K  I  Y  D  F  G  L  S  Q  M  F  E  E

             1,060     1,070     1,080     1,090     1,100     1,110     1,120     1,130     1,140     1,150
WTK3^S4185 GAAGAAACGGAACGTATTGTTGAAAATATCGCCGGAACATTCGGATATATGGCACCGGAGTTTTGTACTAATAATATGGTGTCATTTAAGGCTGAG
WTK3^S4185  E  E  T  E  R  I  V  E  N  I  A  G  T  F  G  Y  M  A  P  E  F  C  T  N  N  M  V  S  F  K  A  E
WTK3^HLT   GAAGAAACGGAACGTATTGTTGAAAATATCGCCGGAACATTCGGATATATGGCACCGGAGTTTTGTACTAATAATATGGTGTCATTTAAGGCTGAG
WTK3^HLT    E  E  T  E  R  I  V  E  N  I  A  G  T  F  G  Y  M  A  P  E  F  C  T  N  N  M  V  S  F  K  A  E
WTK3#1     GAAGAAACGGAACGTATTGTTGAAAATATCGCCGGAACATTCGGATATATGGCACCGGAGTTTTGTACTAATAATATGGTGTCATTTAAGGCTGAG
WTK3#1      E  E  T  E  R  I  V  E  N  I  A  G  T  F  G  Y  M  A  P  E  F  C  T  N  N  M  V  S  F  K  A  E
WTK3#2     GAAGAAACGGAACGTATTGTTGAAAATATCGCCGGAACATTCGGATATATGGCACCGGAGTTTTGTACTAATAATATGGTGTCATTTAAGGCTGAG
WTK3#2      E  E  T  E  R  I  V  E  N  I  A  G  T  F  G  Y  M  A  P  E  F  C  T  N  N  M  V  S  F  K  A  E
WTK3#3     GAAGAAACGGAACGTATTGTTGAAAATATCGCCGGAACATTCGGATATATGGCACCGGAGTTTTGTACTAATAATATGGTGTCATTTAAGGCTGAG
WTK3#3      E  E  T  E  R  I  V  E  N  I  A  G  T  F  G  Y  M  A  P  E  F  C  T  N  N  M  V  S  F  K  A  E

                 1,160     1,170     1,180     1,190     1,200     1,210     1,220     1,230     1,240
WTK3^S4185 ATATACAGTTTGGGCGTTGTGATCGGGGAGTTATTGATCGGGAAGAAAGGATGGTTTGATGAGGATGTGAGAAAACTATTTGTACAGCAACTTAAG
WTK3^S4185  I  Y  S  L  G  V  V  I  G  E  L  L  I  G  K  K  G  W  F  D  E  D  V  R  K  L  F  V  Q  Q  L  K
WTK3^HLT   ATATACAGTTTGGGCGTTGTGATCGGGGAGTTATTGATCGGGAAG------TGGTTTGATGAGGATGTGAGAAAACTATTTGTACAGCAACTTAAG
WTK3^HLT    I  Y  S  L  G  V  V  I  G  E  L  L  I  G  K  ------  W  F  D  E  D  V  R  K  L  F  V  Q  Q  L  K
WTK3#1     ATATACAGTTTGGGCGTTGTGATCGGGGAGTTATTGATCGGGAAG---GGATGGTTTGATGAGGATGTGAGAAAACTATTTGTACAGCAACTTAAG
WTK3#1      I  Y  S  L  G  V  V  I  G  E  L  L  I  G  K  ---  G  W  F  D  E  D  V  R  K  L  F  V  Q  Q  L  K
WTK3#2     ATATACAGTTTGGGCGTTGTGATCGGGGAGTTATTGATCGGGAAGAAA---TGGTTTGATGAGGATGTGAGAAAACTATTTGTACAGCAACTTAAG
WTK3#2      I  Y  S  L  G  V  V  I  G  E  L  L  I  G  K  K  ---  W  F  D  E  D  V  R  K  L  F  V  Q  Q  L  K
WTK3#3     ATATACAGTTTGGGCGTTGTGATCGGG------------TTTGATGAGGATGTGAGAAAACTATTTGTACAGCAACTTAAG
WTK3#3      I  Y  S  L  G  V  V  I  G  E  L  L  I  G  ------------  F  D  E  D  V  R  K  L  F  V  Q  Q  L  K

           1,250     1,260     1,270     1,280     1,290     1,300     1,310     1,320     1,330     1,340
WTK3^S4185 GGTTTGAGAAAAACATTGGTAAAAGAAGGAGCGTTTTCATCATGGGAAAACAAATACCACCAAGTTAGAACATGTATGGAGATTGGGCAGGACTGC
WTK3^S4185  G  L  R  K  T  L  V  K  E  G  A  F  S  S  W  E  N  K  Y  H  Q  V  R  T  C  M  E  I  G  Q  D  C
WTK3^HLT   GGTTTGAGAAAAACATTGGTAAAAGAAGGAGCGTTTTCATCATGGGAAAACAAATACCACCAAGTTAGAACATGTATGGAGATTGGGCAGGACTGC
WTK3^HLT    G  L  R  K  T  L  V  K  E  G  A  F  S  S  W  E  N  K  Y  H  Q  V  R  T  C  M  E  I  G  Q  D  C
WTK3#1     GGTTTGAGAAAAACATTGGTAAAAGAAGGAGCGTTTTCATCATGGGAAAACAAATACCACCAAGTTAGAACATGTATGGAGATTGGGCAGGACTGC
WTK3#1      G  L  R  K  T  L  V  K  E  G  A  F  S  S  W  E  N  K  Y  H  Q  V  R  T  C  M  E  I  G  Q  D  C
WTK3#2     GGTTTGAGAAAAACATTGGTAAAAGAAGGAGCGTTTTCATCATGGGAAAACAAATACCACCAAGTTAGAACATGTATGGAGATTGGGCAGGACTGC
WTK3#2      G  L  R  K  T  L  V  K  E  G  A  F  S  S  W  E  N  K  Y  H  Q  V  R  T  C  M  E  I  G  Q  D  C
WTK3#3     GGTTTGAGAAAAACATTGGTAAAAGAAGGAGCGTTTTCATCATGGGAAAACAAATACCACCAAGTTAGAACATGTATGGAGATTGGGCAGGACTGC
WTK3#3      G  L  R  K  T  L  V  K  E  G  A  F  S  S  W  E  N  K  Y  H  Q  V  R  T  C  M  E  I  G  Q  D  C
```

1,350 1,360 1,370 1,380 1,390 1,400 1,410 1,420 1,430 1,440

WTK3^{S4185} ATAGACCCCAACCCACATAAAAGGCCCACTTTGTTGGAGATTATCCAGCGGCTTAATGAAGCGGAAGATATGAACTATTCTGCAGCATCACTTTGG
WTK3^{S4185} I D P N P H K R P T L L E I I Q R L N E A E D M N Y S A A S L W
WTK3^{HLT} ATAGACCCCAACCCACATAAAAGGCCCACTTTGTTGGAGATTATCCAGCGGCTTAATGAAGCGGAAGATATGAACTATTCTGCAGCATCACTTTGG
WTK3^{HLT} I D P N P H K R P T L L E I I Q R L N E A E D M N Y S A A S L W
WTK3#1 ATAGACCCCAACCCACATAAAAGGCCCACTTTGTTGGAGATTATCCAGCGGCTTAATGAAGCGGAAGATATGAACTATTCTGCAGCATCACTTTGG
WTK3#1 I D P N P H K R P T L L E I I Q R L N E A E D M N Y S A A S L W
WTK3#2 ATAGACCCCAACCCACATAAAAGGCCCACTTTGTTGGAGATTATCCAGCGGCTTAATGAAGCGGAAGATATGAACTATTCTGCAGCATCACTTTGG
WTK3#2 I D P N P H K R P T L L E I I Q R L N E A E D M N Y S A A S L W
WTK3#3 ATAGACCCCAACCCACATAAAAGGCCCACTTTGTTGGAGATTATCCAGCGGCTTAATGAAGCGGAAGATATGAACTATTCTGCAGCATCACTTTGG
WTK3#3 I D P N P H K R P T L L E I I Q R L N E A E D M N Y S A A S L W

1,450 1,460 1,470 1,480 1,490 1,500 1,510 1,520 1,530

WTK3^{S4185} CAGTCAGGAGACGAGGAATCCGATTTATCGGATACAGAAGCTTTGGAGACAGAGACAACATCCGAGTTTCTTCCAAGTGACGAAGAACCCGCCTCT
WTK3^{S4185} Q S G D E E S D L S D T E A L E T E T T S E F L P S D E E P A S
WTK3^{HLT} CAGTCAGGAGACGAGGAATCCGATTTATCGGATACAGAAGCTTTGGAGACAGAGACAACATCCGAGTTTCTTCCAAGTGACGAAGAACCCGCCTCT
WTK3^{HLT} Q S G D E E S D L S D T E A L E T E T T S E F L P S D E E P A S
WTK3#1 CAGTCAGGAGACGAGGAATCCGATTTATCGGATACAGAAGCTTTGGAGACAGAGACAACATCCGAGTTTCTTCCAAGTGACGAAGAACCCGCCTCT
WTK3#1 Q S G D E E S D L S D T E A L E T E T T S E F L P S D E E P A S
WTK3#2 CAGTCAGGAGACGAGGAATCCGATTTATCGGATACAGAAGCTTTGGAGACAGAGACAACATCCGAGTTTCTTCCAAGTGACGAAGAACCCGCCTCT
WTK3#2 Q S G D E E S D L S D T E A L E T E T T S E F L P S D E E P A S
WTK3#3 CAGTCAGGAGACGAGGAATCCGATTTATCGGATACAGAAGCTTTGGAGACAGAGACAACATCCGAGTTTCTTCCAAGTGACGAAGAACCCGCCTCT
WTK3#3 Q S G D E E S D L S D T E A L E T E T T S E F L P S D E E P A S

1,540 1,550 1,560 1,570 1,580 1,590 1,600 1,610 1,620 1,630

WTK3^{S4185} GTGGGCAAGACCGGAGAAACAAGCACACAGGAGCCTGATAAACCGGACCTAATAAGTAAGTTGCCAGCATCGGTGGACCTGTCTGACCTAAAAGTC
WTK3^{S4185} V G K T G E T S T Q E P D K P D L I S K L P A S V D L S D L K V
WTK3^{HLT} GTGGGCAAGACCGGAGAAACAAGCACACAGGAGCCTGATAAACCGGACCTAATAAGTAAGTTGCCAGCATCGGTGGACCTGTCTGACCTAAAAGTC
WTK3^{HLT} V G K T G E T S T Q E P D K P D L I S K L P A S V D L S D L K V
WTK3#1 GTGGGCAAGACCGGAGAAACAAGCACACAGGAGCCTGATAAACCGGACCTAATAAGTAAGTTGCCAGCATCGGTGGACCTGTCTGACCTAAAAGTC
WTK3#1 V G K T G E T S T Q E P D K P D L I S K L P A S V D L S D L K V
WTK3#2 GTGGGCAAGACCGGAGAAACAAGCACACAGGAGCCTGATAAACCGGACCTAATAAGTAAGTTGCCAGCATCGGTGGACCTGTCTGACCTAAAAGTC
WTK3#2 V G K T G E T S T Q E P D K P D L I S K L P A S V D L S D L K V
WTK3#3 GTGGGCAAGACCGGAGAAACAAGCACACAGGAGCCTGATAAACCGGACCTAATAAGTAAGTTGCCAGCATCGGTGGACCTGTCTGACCTAAAAGTC
WTK3#3 V G K T G E T S T Q E P D K P D L I S K L P A S V D L S D L K V

1,640 1,650 1,660 1,670 1,680 1,690 1,700 1,710 1,720

WTK3^{S4185} CTGGAGAAAATCACAGATGATTTTTCACACGAAAGAATAGTTGGGAAGGACGGTACATTCAAAGGTTGTCATAAGGCATTTGTTTATAAGGGTGAC
WTK3^{S4185} L E K I T D D F S H E R I V G K D G T F K G C H K A F V Y K G D
WTK3^{HLT} CTGGAGAAAATCACAGATGATTTTTCACACGAAAGAATAGTTGGGAAGGACGGTACATTCAAAGGTTGTCATAAGGCATTTGTTTATAAGGGTGAC
WTK3^{HLT} L E K I T D D F S H E R I V G K D G T F K G C H K A F V Y K G D
WTK3#1 CTGGAGAAAATCACAGATGATTTTTCACACGAAAGAATAGTTGGGAAGGACGGTACATTCAAAGGTTGTCATAAGGCATTTGTTTATAAGGGTGAC
WTK3#1 L E K I T D D F S H E R I V G K D G T F K G C H K A F V Y K G D
WTK3#2 CTGGAGAAAATCACAGATGATTTTTCACACGAAAGAATAGTTGGGAAGGACGGTACATTCAAAGGTTGTCATAAGGCATTTGTTTATAAGGGTGAC
WTK3#2 L E K I T D D F S H E R I V G K D G T F K G C H K A F V Y K G D
WTK3#3 CTGGAGAAAATCACAGATGATTTTTCACACGAAAGAATAGTTGGGAAGGACGGTACATTCAAAGGTTGTCATAAGGCATTTGTTTATAAGGGTGAC
WTK3#3 L E K I T D D F S H E R I V G K D G T F K G C H K A F V Y K G D

1,730 1,740 1,750 1,760 1,770 1,780 1,790 1,800 1,810 1,820

WTK3^{S4185} ATTCCACTTAGAGAAATGATAGCCGTGAAGAGGTTAATTGGAGTGGAGATTCCATTTGAAAAGTTTAAGAGGGAAGCAGAACAGTTCATTAGTCTC
WTK3^{S4185} I P L R E M I A V K R L I G V E I P F E K F K R E A E Q F I S L
WTK3^{HLT} ATTCCACTTAGAGAAATGATAGCCGTGAAGAGGTTAATTGGAGTGGAGATTCCATTTGAAAAGTTTAAGAGGGAAGCAGAACAGTTCATTAGTCTC
WTK3^{HLT} I P L R E M I A V K R L I G V E I P F E K F K R E A E Q F I S L
WTK3#1 ATTCCACTTAGAGAAATGATAGCCGTGAAGAGGTTAATTGGAGTGGAGATTCCATTTGAAAAGTTTAAGAGGGAAGCAGAACAGTTCATTAGTCTC
WTK3#1 I P L R E M I A V K R L I G V E I P F E K F K R E A E Q F I S L
WTK3#2 ATTCCACTTAGAGAAATGATAGCCGTGAAGAGGTTAATTGGAGTGGAGATTCCATTTGAAAAGTTTAAGAGGGAAGCAGAACAGTTCATTAGTCTC
WTK3#2 I P L R E M I A V K R L I G V E I P F E K F K R E A E Q F I S L
WTK3#3 ATTCCACTTAGAGAAATGATAGCCGTGAAGAGGTTAATTGGAGTGGAGATTCCATTTGAAAAGTTTAAGAGGGAAGCAGAACAGTTCATTAGTCTC
WTK3#3 I P L R E M I A V K R L I G V E I P F E K F K R E A E Q F I S L

1,830 1,840 1,850 1,860 1,870 1,880 1,890 1,900 1,910 1,920

WTK3^{S4185} GATCATAAGAATATAGTAAAGGTTGCCAGCTACTGCCACGACCAGTCTAGAGGACATAGACTGGTACAGTTCAAAGGAAAACCGCTACCACAACTC
WTK3^{S4185} D H K N I V K V A S Y C H D Q S R G H R L V Q F K G K P L P Q L
WTK3^{HLT} GATCATAAGAATATAGTAAAGGTTGCCAGCTACTGCCACGACCAGTCTAGAGGACATAGACTGGTACAGTTCAAAGGAAAACCGCTACCACAACTC
WTK3^{HLT} D H K N I V K V A S Y C H D Q S R G H R L V Q F K G K P L P Q L
WTK3#1 GATCATAAGAATATAGTAAAGGTTGCCAGCTACTGCCACGACCAGTCTAGAGGACATAGACTGGTACAGTTCAAAGGAAAACCGCTACCACAACTC
WTK3#1 D H K N I V K V A S Y C H D Q S R G H R L V Q F K G K P L P Q L
WTK3#2 GATCATAAGAATATAGTAAAGGTTGCCAGCTACTGCCACGACCAGTCTAGAGGACATAGACTGGTACAGTTCAAAGGAAAACCGCTACCACAACTC
WTK3#2 D H K N I V K V A S Y C H D Q S R G H R L V Q F K G K P L P Q L
WTK3#3 GATCATAAGAATATAGTAAAGGTTGCCAGCTACTGCCACGACCAGTCTAGAGGACATAGACTGGTACAGTTCAAAGGAAAACCGCTACCACAACTC
WTK3#3 D H K N I V K V A S Y C H D Q S R G H R L V Q F K G K P L P Q L

1,930 1,940 1,950 1,960 1,970 1,980 1,990 2,000 2,010

WTK3^{S4185} TTTAACGGTCCCGAACAACTGCTCTGCTATGAATATATGCACAACGGAAGCCTTCGCGACTATCTTATGGGTCAAGGATCTCGTGTAATTGATTGG
WTK3^{S4185} F N G P E Q L L C Y E Y M H N G S L R D Y L M G Q G S R V I D W
WTK3^{HLT} TTTAACGGTCCCGAACAACTGCTCTGCTATGAATATATGCACAACGGAAGCCTTCGCGACTATCTTATGGGTCAAGGATCTCGTGTAATTGATTGG
WTK3^{HLT} F N G P E Q L L C Y E Y M H N G S L R D Y L M G Q G S R V I D W
WTK3#1 TTTAACGGTCCCGAACAACTGCTCTGCTATGAATATATGCACAACGGAAGCCTTCGCGACTATCTTATGGGTCAAGGATCTCGTGTAATTGATTGG
WTK3#1 F N G P E Q L L C Y E Y M H N G S L R D Y L M G Q G S R V I D W
WTK3#2 TTTAACGGTCCCGAACAACTGCTCTGCTATGAATATATGCACAACGGAAGCCTTCGCGACTATCTTATGGGTCAAGGATCTCGTGTAATTGATTGG
WTK3#2 F N G P E Q L L C Y E Y M H N G S L R D Y L M G Q G S R V I D W
WTK3#3 TTTAACGGTCCCGAACAACTGCTCTGCTATGAATATATGCACAACGGAAGCCTTCGCGACTATCTTATGGGTCAAGGATCTCGTGTAATTGATTGG
WTK3#3 F N G P E Q L L C Y E Y M H N G S L R D Y L M G Q G S R V I D W

```
             2,020     2,030     2,040     2,050     2,060     2,070     2,080     2,090     2,100     2,110
WTK3S4185 CAAATGCGCTACAAATTGATCAAAGGGACTTGCGCAGGCTTACATTACCTTCACAAGGGCCGTGCAGGTTGTCCAATTGTTCATTTGAATTTAAGC
WTK3S4185  Q  M  R  Y  K  L  I  K  G  T  C  A  G  L  H  Y  L  H  K  G  R  A  G  C  P  I  V  H  L  N  L  S
WTK3HLT   CAAATGCGCTACAAATTGATCAAAGGGACTTGCGCAGGCTTACATTACCTTCACAAGGGCCGTGCAGGTTGTCCAATTGTTCATTTGAATTTAAGC
WTK3HLT    Q  M  R  Y  K  L  I  K  G  T  C  A  G  L  H  Y  L  H  K  G  R  A  G  C  P  I  V  H  L  N  L  S
WTK3#1    CAAATGCGCTACAAATTGATCAAAGGGACTTGCGCAGGCTTACATTACCTTCACAAGGGCCGTGCAGGTTGTCCAATTGTTCATTTGAATTTAAGC
WTK3#1     Q  M  R  Y  K  L  I  K  G  T  C  A  G  L  H  Y  L  H  K  G  R  A  G  C  P  I  V  H  L  N  L  S
WTK3#2    CAAATGCGCTACAAATTGATCAAAGGGACTTGCGCAGGCTTACATTACCTTCACAAGGGCCGTGCAGGTTGTCCAATTGTTCATTTGAATTTAAGC
WTK3#2     Q  M  R  Y  K  L  I  K  G  T  C  A  G  L  H  Y  L  H  K  G  R  A  G  C  P  I  V  H  L  N  L  S
WTK3#3    CAAATGCGCTACAAATTGATCAAAGGGACTTGCGCAGGCTTACATTACCTTCACAAGGGCCGTGCAGGTTGTCCAATTGTTCATTTGAATTTAAGC
WTK3#3     Q  M  R  Y  K  L  I  K  G  T  C  A  G  L  H  Y  L  H  K  G  R  A  G  C  P  I  V  H  L  N  L  S

                 2,120     2,130     2,140     2,150     2,160     2,170     2,180     2,190     2,200
WTK3S4185 CCGTCAAATGTATTGCTGGACCACAACTACATACCACGCATCACAGGGTTCGATTTTTCGAAGCTCATTGGTGAAAAGAACACCAAATCAGTGGTA
WTK3S4185  P  S  N  V  L  L  D  H  N  Y  I  P  R  I  T  G  F  D  F  S  K  L  I  G  E  K  N  T  K  S  V  V
WTK3HLT   CCGTCAAATGTATTGCTGGACCACAACTACATACCACGCATCACAGGGTTCGATTTTTCGAAGCTCATTGGTGAAAAGAACACCAAATCAGTGGTA
WTK3HLT    P  S  N  V  L  L  D  H  N  Y  I  P  R  I  T  G  F  D  F  S  K  L  I  G  E  K  N  T  K  S  V  V
WTK3#1    CCGTCAAATGTATTGCTGGACCACAACTACATACCACGCATCACAGGGTTCGATTTTTCGAAGCTCATTGGTGAAAAGAACACCAAATCAGTGGTA
WTK3#1     P  S  N  V  L  L  D  H  N  Y  I  P  R  I  T  G  F  D  F  S  K  L  I  G  E  K  N  T  K  S  V  V
WTK3#2    CCGTCAAATGTATTGCTGGACCACAACTACATACCACGCATCACAGGGTTCGATTTTTCGAAGCTCATTGGTGAAAAGAACACCAAATCAGTGGTA
WTK3#2     P  S  N  V  L  L  D  H  N  Y  I  P  R  I  T  G  F  D  F  S  K  L  I  G  E  K  N  T  K  S  V  V
WTK3#3    CCGTCAAATGTATTGCTGGACCACAACTACATACCACGCATCACAGGGTTCGATTTTTCGAAGCTCATTGGTGAAAAGAACACCAAATCAGTGGTA
WTK3#3     P  S  N  V  L  L  D  H  N  Y  I  P  R  I  T  G  F  D  F  S  K  L  I  G  E  K  N  T  K  S  V  V

          2,210     2,220     2,230     2,240     2,250     2,260     2,270     2,280     2,290     2,300
WTK3S4185 CTTAAGCTGAATGGACCCATAGCGTACCTGCCACCGGATTTCTTCCATTCGAAGGGTACTGATCTTAAATATCTTGCTACGGTAGATATATACAGC
WTK3S4185  L  K  L  N  G  P  I  A  Y  L  P  P  D  F  F  H  S  K  G  T  D  L  K  Y  L  A  T  V  D  I  Y  S
WTK3HLT   CTTAAGCTGAATGGACCCATAGCGTACCTGCCACCGGATTTCTTCCATTCGAAGGGTACTGATCTTAAATATCTTGCTACGGTAGATATATACAGC
WTK3HLT    L  K  L  N  G  P  I  A  Y  L  P  P  D  F  F  H  S  K  G  T  D  L  K  Y  L  A  T  V  D  I  Y  S
WTK3#1    CTTAAGCTGAATGGACCCATAGCGTACCTGCCACCGGATTTCTTCCATTCGAAGGGTACTGATCTTAAATATCTTGCTACGGTAGATATATACAGC
WTK3#1     L  K  L  N  G  P  I  A  Y  L  P  P  D  F  F  H  S  K  G  T  D  L  K  Y  L  A  T  V  D  I  Y  S
WTK3#2    CTTAAGCTGAATGGACCCATAGCGTACCTGCCACCGGATTTCTTCCATTCGAAGGGTACTGATCTTAAATATCTTGCTACGGTAGATATATACAGC
WTK3#2     L  K  L  N  G  P  I  A  Y  L  P  P  D  F  F  H  S  K  G  T  D  L  K  Y  L  A  T  V  D  I  Y  S
WTK3#3    CTTAAGCTGAATGGACCCATAGCGTACCTGCCACCGGATTTCTTCCATTCGAAGGGTACTGATCTTAAATATCTTGCTACGGTAGATATATACAGC
WTK3#3     L  K  L  N  G  P  I  A  Y  L  P  P  D  F  F  H  S  K  G  T  D  L  K  Y  L  A  T  V  D  I  Y  S

               2,310     2,320     2,330     2,340     2,350     2,360     2,370     2,380     2,390     2,400
WTK3S4185 TTGGGTCTTATGATTTTAGAAATCGCAACACAACAAGAGATCAAAGGCATCCATGGAGTGCTTATTAAGAGTATAGAGGAAAACTGGAGGGAGGAG
WTK3S4185  L  G  L  M  I  L  E  I  A  T  Q  Q  E  I  K  G  I  H  G  V  L  I  K  S  I  E  E  N  W  R  E  E
WTK3HLT   TTGGGTCTTATGATTTTAGAAATCGCAACACAACAAGAGATCAAAGGCATCCATGGAGTGCTTATTAAGAGTATAGAGGAAAACTGGAGGGAGGAG
WTK3HLT    L  G  L  M  I  L  E  I  A  T  Q  Q  E  I  K  G  I  H  G  V  L  I  K  S  I  E  E  N  W  R  E  E
WTK3#1    TTGGGTCTTATGATTTTAGAAATCGCAACACAACAAGAGATCAAAGGCATCCATGGAGTGCTTATTAAGAGTATAGAGGAAAACTGGAGGGAGGAG
WTK3#1     L  G  L  M  I  L  E  I  A  T  Q  Q  E  I  K  G  I  H  G  V  L  I  K  S  I  E  E  N  W  R  E  E
WTK3#2    TTGGGTCTTATGATTTTAGAAATCGCAACACAACAAGAGATCAAAGGCATCCATGGAGTGCTTATTAAGAGTATAGAGGAAAACTGGAGGGAGGAG
WTK3#2     L  G  L  M  I  L  E  I  A  T  Q  Q  E  I  K  G  I  H  G  V  L  I  K  S  I  E  E  N  W  R  E  E
WTK3#3    TTGGGTCTTATGATTTTAGAAATCGCAACACAACAAGAGATCAAAGGCATCCATGGAGTGCTTATTAAGAGTATAGAGGAAAACTGGAGGGAGGAG
WTK3#3     L  G  L  M  I  L  E  I  A  T  Q  Q  E  I  K  G  I  H  G  V  L  I  K  S  I  E  E  N  W  R  E  E

                 2,410     2,420     2,430     2,440     2,450     2,460     2,470     2,480     2,490
WTK3S4185 TCACAAATAACACGGCTGTATACCTCACTAGGGGCCGACGAGCTGCGGCAAGTAAAAATGTGCATTGATATTGGCCTAGACTGTGTCAAGTCAAAC
WTK3S4185  S  Q  I  T  R  L  Y  T  S  L  G  A  D  E  L  R  Q  V  K  M  C  I  D  I  G  L  D  C  V  K  S  N
WTK3HLT   TCACAAATAACACGGCTGTATACCTCACTAGGGGCCGACGAGCTGCGGCAAGTAAAAATGTGCATTGATATTGGCCTAGACTGTGTCAAGTCAAAC
WTK3HLT    S  Q  I  T  R  L  Y  T  S  L  G  A  D  E  L  R  Q  V  K  M  C  I  D  I  G  L  D  C  V  K  S  N
WTK3#1    TCACAAATAACACGGCTGTATACCTCACTAGGGGCCGACGAGCTGCGGCAAGTAAAAATGTGCATTGATATTGGCCTAGACTGTGTCAAGTCAAAC
WTK3#1     S  Q  I  T  R  L  Y  T  S  L  G  A  D  E  L  R  Q  V  K  M  C  I  D  I  G  L  D  C  V  K  S  N
WTK3#2    TCACAAATAACACGGCTGTATACCTCACTAGGGGCCGACGAGCTGCGGCAAGTAAAAATGTGCATTGATATTGGCCTAGACTGTGTCAAGTCAAAC
WTK3#2     S  Q  I  T  R  L  Y  T  S  L  G  A  D  E  L  R  Q  V  K  M  C  I  D  I  G  L  D  C  V  K  S  N
WTK3#3    TCACAAATAACACGGCTGTATACCTCACTAGGGGCCGACGAGCTGCGGCAAGTAAAAATGTGCATTGATATTGGCCTAGACTGTGTCAAGTCAAAC
WTK3#3     S  Q  I  T  R  L  Y  T  S  L  G  A  D  E  L  R  Q  V  K  M  C  I  D  I  G  L  D  C  V  K  S  N

             2,500     2,510     2,520     2,530     2,540     2,550     2,560     2,570     2,580     2,590
WTK3S4185 CCTGAAAAGAGACCTACAGCTGGGGCCATCATGCTCTGGCTTGACAAAGAGAGCAAACCGGTCCCAGTTTCAAGGGCAGGTGCAGGAGTGCTGCCA
WTK3S4185  P  E  K  R  P  T  A  G  A  I  M  L  W  L  D  K  E  S  K  P  V  P  V  S  R  A  G  A  G  V  L  P
WTK3HLT   CCTGAAAAGAGACCTACAGCTGGGGCCATCATGCTCTGGCTTGACAAAGAGAGCAAACCGGTCCCAGTTTCAAGGGCAGGTGCAGGAGTGCTGCCA
WTK3HLT    P  E  K  R  P  T  A  G  A  I  M  L  W  L  D  K  E  S  K  P  V  P  V  S  R  A  G  A  G  V  L  P
WTK3#1    CCTGAAAAGAGACCTACAGCTGGGGCCATCATGCTCTGGCTTGACAAAGAGAGCAAACCGGTCCCAGTTTCAAGGGCAGGTGCAGGAGTGCTGCCA
WTK3#1     P  E  K  R  P  T  A  G  A  I  M  L  W  L  D  K  E  S  K  P  V  P  V  S  R  A  G  A  G  V  L  P
WTK3#2    CCTGAAAAGAGACCTACAGCTGGGGCCATCATGCTCTGGCTTGACAAAGAGAGCAAACCGGTCCCAGTTTCAAGGGCAGGTGCAGGAGTGCTGCCA
WTK3#2     P  E  K  R  P  T  A  G  A  I  M  L  W  L  D  K  E  S  K  P  V  P  V  S  R  A  G  A  G  V  L  P
WTK3#3    CCTGAAAAGAGACCTACAGCTGGGGCCATCATGCTCTGGCTTGACAAAGAGAGCAAACCGGTCCCAGTTTCAAGGGCAGGTGCAGGAGTGCTGCCA
WTK3#3     P  E  K  R  P  T  A  G  A  I  M  L  W  L  D  K  E  S  K  P  V  P  V  S  R  A  G  A  G  V  L  P

                 2,600     2,610     2,620     2,630     2,640     2,650     2,660     2,670     2,680  2,688
WTK3S4185 AGACCTCCGGTCCCTACTAATATCAACCATGCAGGTCGCATCCAAGAAAAGGAGAAGGCGGGATTCCTGAAACGACACTTCGGATGGAAGAAGTAA
WTK3S4185  R  P  P  V  P  T  N  I  N  H  A  G  R  I  Q  E  K  E  K  A  G  F  L  K  R  H  F  G  W  K  K  *
WTK3HLT   AGACCTCCGGTCCCTACTAATATCAACCATGCAGGTCGCATCCAAGAAAAGGAGAAGGCGGGATTCCTGAAACGACACTTCGGATGGAAGAAGTAA
WTK3HLT    R  P  P  V  P  T  N  I  N  H  A  G  R  I  Q  E  K  E  K  A  G  F  L  K  R  H  F  G  W  K  K  *
WTK3#1    AGACCTCCGGTCCCTACTAATATCAACCATGCAGGTCGCATCCAAGAAAAGGAGAAGGCGGGATTCCTGAAACGACACTTCGGATGGAAGAAGTAA
WTK3#1     R  P  P  V  P  T  N  I  N  H  A  G  R  I  Q  E  K  E  K  A  G  F  L  K  R  H  F  G  W  K  K  *
WTK3#2    AGACCTCCGGTCCCTACTAATATCAACCATGCAGGTCGCATCCAAGAAAAGGAGAAGGCGGGATTCCTGAAACGACACTTCGGATGGAAGAAGTAA
WTK3#2     R  P  P  V  P  T  N  I  N  H  A  G  R  I  Q  E  K  E  K  A  G  F  L  K  R  H  F  G  W  K  K  *
WTK3#3    AGACCTCCGGTCCCTACTAATATCAACCATGCAGGTCGCATCCAAGAAAAGGAGAAGGCGGGATTCCTGAAACGACACTTCGGATGGAAGAAGTAA
WTK3#3     R  P  P  V  P  T  N  I  N  H  A  G  R  I  Q  E  K  E  K  A  G  F  L  K  R  H  F  G  W  K  K  *
```

Supplementary Figure 7 Comparison of CDS and amino acid sequences of the WTK3 chimeric variants. The sequences in the red rectangle indicated the difference between the *WTK3*^{S4185}, *WTK3*^{HLT}, *WTK3#1*, *WTK3#2* and *WTK3#3*.

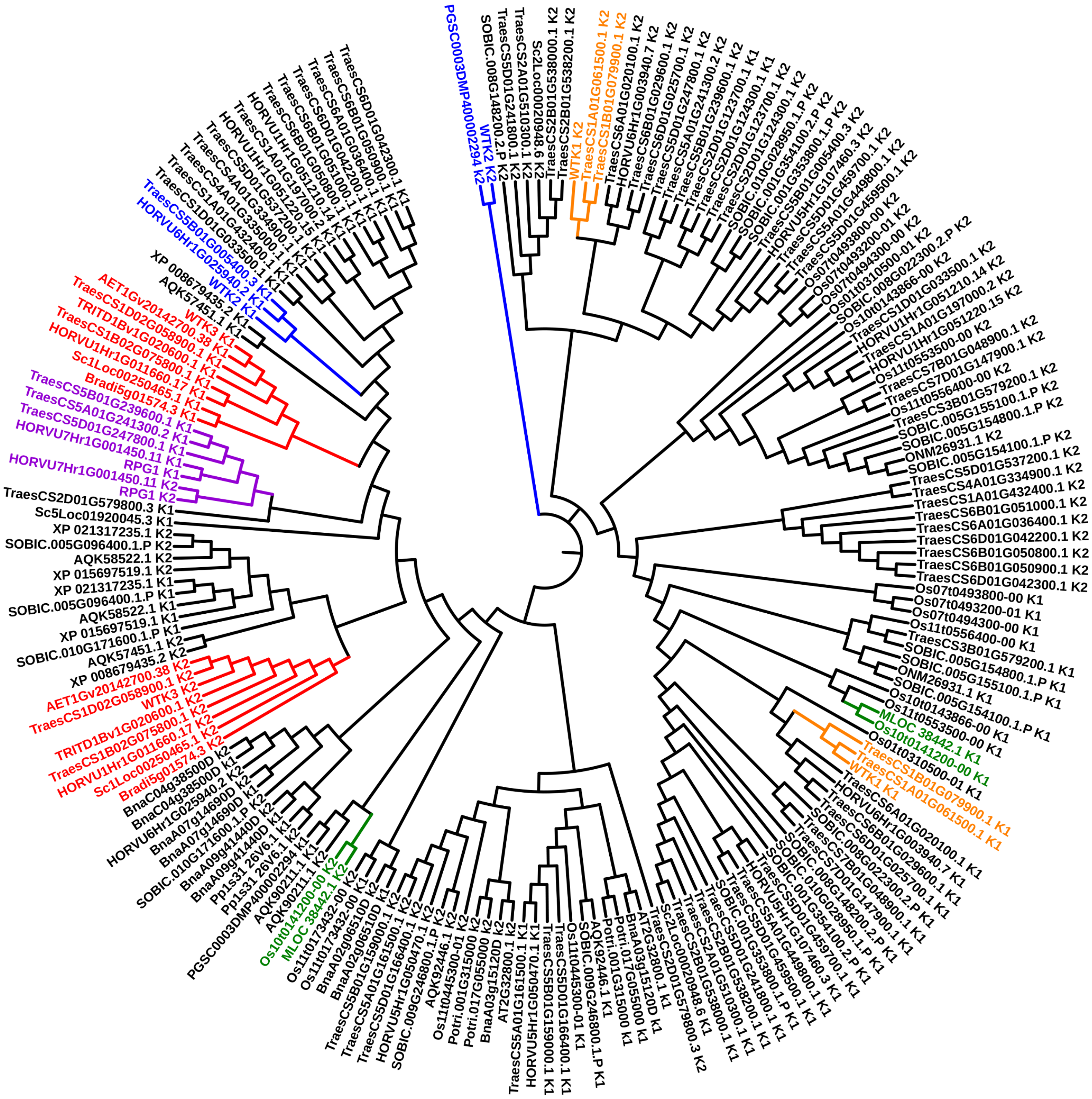

Supplementary Figure 8 Phylogenetic analysis of plant protein kinase domains. The closest homologs and branches of Kin I (K1) and Kin II (K2) domains of WTK1, WTK2, WTK3, Rpg1 and Un8 are indicated in orange, blue, red, purple and green, respectively.

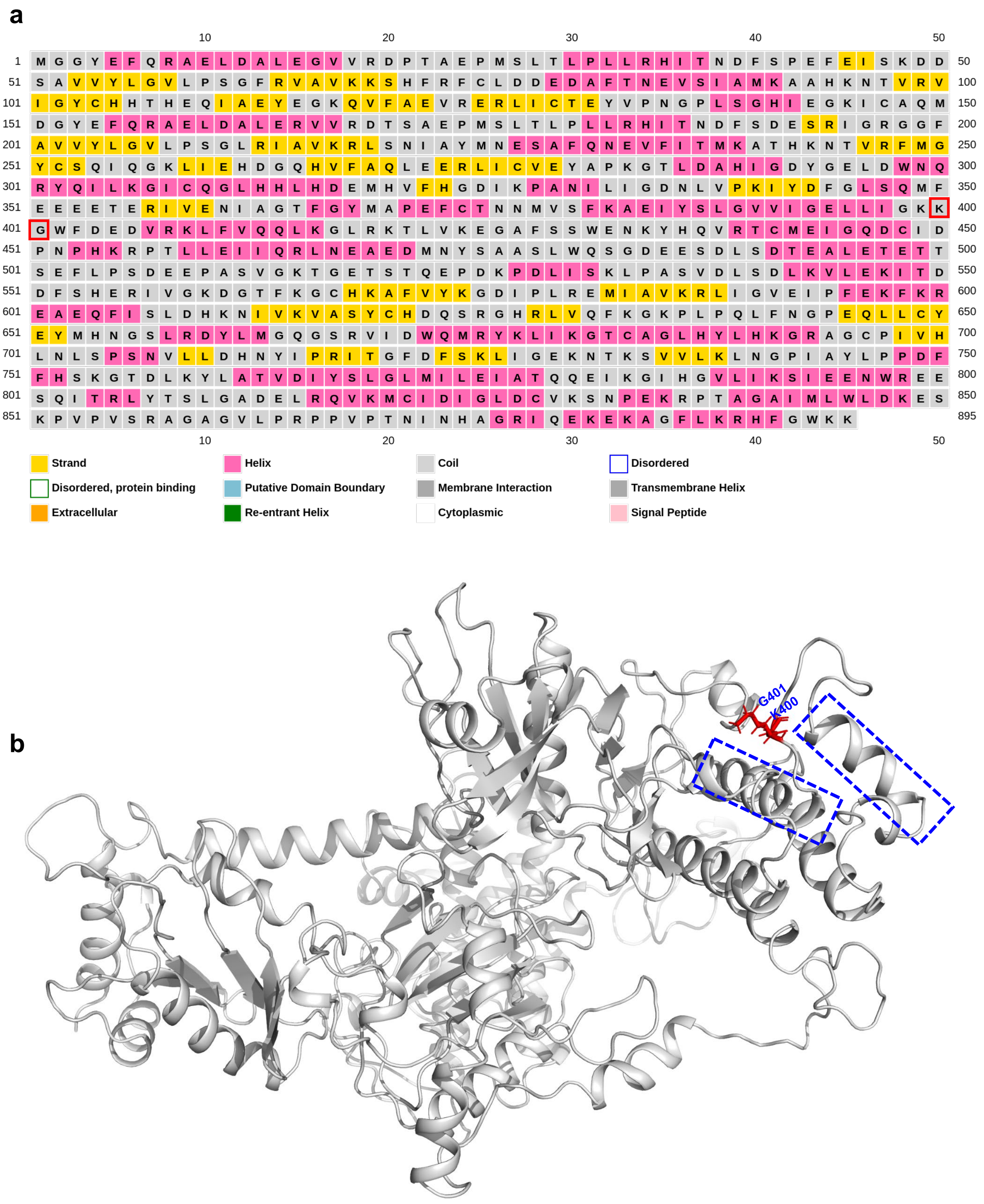

Supplementary Figure 9 The predicted secondary and tertiary structures of WTK3.

a, Predicted secondary structure of WTK3. The amino acid residues in the red rectangle were the key K400G401 deletion. **b,** Predicted tertiary structure of WTK3. The K400G401 residues lie in a loop region (red region) between two α-helixes (in the blue rectangle). The secondary structure of WTK3 was predicted by PSIPRED (http://bioinf.cs.ucl.ac.uk/psipred/), and the RosettaCM method was used to build the model of WTK3 tertiary structure.

Supplementary Table 1 Infection type of HLT, CYC, BHL, Hongmangmai and Chancellor to divergent *Bgt* isolates

Bgt	Collection site	Chiyacao (CYC)	Baihulu (BHL)	Hulutou (HLT)	Hongmangmai (HMM)	Chancellor
E09	Beijing	0	0	0	-	4
E21	Beijing	0;	1	2	1	4
Bg68-1	Beijing	0	0	0	-	4
Bg68-2	Beijing	0	0	0	-	4
Bg68-3	Beijing	0	0	0	-	4
Bg87	Beijing	0	0	0	-	4
Bg69-1	Cixian, Hebei	0	0	0	-	4
Bg69-2	Cixian, Hebei	0	0	0	-	4
Bg69-3	Cixian, Hebei	0	0	0	-	4
Bg70-2	Gucheng, Hebei	0	0	0	-	4
Bg70-1	Gucheng, Hebei	0	0	0	-	4
Bg70-3	Gucheng, Hebei	0	0	0	-	4
39-19	Handan, Hebei	0	0	0;	0	4
Bg71-2	Shahe, Hebei	2	0	0	-	4
Bg71-3	Shahe, Hebei	0	0	0	-	4
Bg72	Shijiazhuang, Hebei	0	0	0	-	4
37-38	Shijiazhuang, Hebei	0;	0;	0;	0;	4
Bg57-3	Xingtai, Hebei	0	0	0	-	4
Bg57-4	Xingtai, Hebei	0	0	0	-	4
Bg57-5	Xingtai, Hebei	0	0	0	-	4
Bg73-2	Yuansi, Hebei	0	0	0	-	4
Bg73-3	Yuansi, Hebei	0	0	0	-	4
Bg74-1	Zhuozhou, Hebei	0	0	0	-	4
Bg74-2	Zhuozhou, Hebei	0	0	0	-	4
Bg74-3	Zhuozhou, Hebei	0	0	0	-	4
Bg76-1	Lankao, Henan	0	0	0	-	4
Bg76-3	Lankao, Henan	0	0	0	-	4
18-11	Shangqiu, Henan	1	1	1	0;	4
18-45	Shangqiu, Henan	0;	0	1	0;	4
Bg77-1	Xihua, Henan	0	0	0	-	4
Bg77-3	Xihua, Henan	0	0	0	-	4
Bg78-1	Xinxiang, Henan	1	2	1	-	4
Bg78-2	Xinxiang, Henan	0	0	1	-	4
Bg78-3	Xinxiang, Henan	0	0	1	-	4
Bg75-1	Xunxian, Henan	0	0	0	-	4

Bg75-2	Xunxian, Henan	0	0	0	-	4
Bg75-3	Xunxian, Henan	1	0	1	-	4
HB-24	Gucheng, Hubei	0;	0;	0;	0;	4
NZ-1	Nanzhang, Hubei	0	0;	0;	0;	4
HB-4	Xiangyang, Hubei	0	0;	0;	0;	4
5-36	Meixian, Shaanxi	1	1	2	1	4
5-83	Meixian, Shaanxi	0;	0	0	0	4
6-21	Qishan, Shaanxi	0;	0	1	0;	4
7-8	Zhouzhi, Shaanxi	0	0	0	0	4
46-31	Gangu, Gansu	0	0	0	0	4
46-30	Gangu, Gansu	0	0	0	0	4
47-3	Wenxian, Gansu	0;	0;	1	0;	4
SD-4	Taian, Shandong	0;	0;	0;	0;	4
Bg88-2	Haniyang, Shandong	1	1	1	-	4
Bg88-3	Haiyang, Shandong	0	0	0	-	4
Bg79-1	Jining, Shandong	0	0	0	-	4
Bg79-2	Jining, Shandong	2	0	2	-	4
Bg79-3	Jining, Shandong	1	0	1	-	4
Bg80-3	Lvxian, Shandong	0	0	0	-	4
Bg81-2	Pingyi, Shandong	0	1	0	-	4
Bg81-3	Pingyi, Shandong	0	0	0	-	4
Bg82-2	Pingyi, Shandong	0	0	0	-	4
41-5	Qufu, Shandong	0	0;	0;	0	4
Bg82-1	Shouguang, Shandong	0	0	0	-	4
Bg82-3	Shouguang, Shandong	0	0	0	-	4
Bg83-1	Wendeng, Shandong	0	0	0	-	4
Bg83-2	Wendeng, Shandong	0	0	0	-	4
Bg84-1	Yuncheng, Shandong	0	0	0	-	4
Bg84-2	Yuncheng, Shandong	0	0	0	-	4
Bg84-3	Yuncheng, Shandong	0	0	0	-	4
Bg44-5	Zhanhua, Shandong	0	0	0	-	4
Bg85-1	Zhaoyuan, Shandong	0	0	0	-	4
Bg85-2	Zhaoyuan, Shandong	0	0	0	-	4
Bg85-3	Zhaoyuan, Shandong	1	2	1	-	4
17-18	Fuyang, Jiangsu	0;	0;	0;	0;	4
21-1	Yancheng, Jiangsu	0	0;	1	1	4
21-2	Yancheng, Jiangsu	0	0	0	0	4
Bg86-3	Yangzhou, Jiangsu	0	0	2	-	4

Bg86-2	Yangzhou, Jiangsu	0	0	0	-	4
Bg86-1	Yangzhou, Jiangsu	0	0	0	-	4
SC-12	Chengdu, Sichuan	0	0;	0;	0;	4
1-19	Mianyang, Sichuan	1	1	1	1	4
3-53	Yanting, Sichuan	1	0;	0;	0;	4
2-5	Zitong, Sichuan	0	0	0	0	4
2-65	Zitong, Sichuan	0;	0	1	1	4
8-9	Dehongmang, Yunnan	0	0	1	0	4
9-2	Dehongmang, Yunnan	0	0	1	0	4
9-10	Dehongmang, Yunnan	0	0	0	0	4
9-43	Dehongmang, Yunnan	0;	0	0;	0;	4
13-51	Anshun, Guizhou	0	0	0	0;	4
12-50	Yixing, Guizhou	0;	1	2	1	4
12-82	Yixing, Guizhou	1	0	1	1	4
49-1	Yining, Xinjiang	0	0	0;	0	4
50-2	Yining, Xinjiang	0	0	0	0	4
51-3	Nalati, Xinjiang	1	0;	1	0;	4
52-27	Fukang, Xinjiang	0;	0;	1	0;	4
Bg44-4	Unknown	2	0	1	-	4
Bg44-6	Unknown	0	0	0	-	4

Supplementary Table 2 List of PCR primers used for fine genetic mapping, 5′ RACE and 3′ RACE, CDS amplification, marker-assisted selection (MAS), allelic (haplotype) variation analysis, transgenic vectors construction, validation of the presence of transgene, candidate genes expression analysis, qPCR for *WTK3* and *PR* genes.

Marker	Primer sequence 5'-3' (Forward)	Primer sequence 5'-3' (Reverse)	Function
WGGB240	TAGTAAGTTCGTGCAATGAGC	CATCAGATTAACGGCTCCTAA	Fine mapping, SSR marker
WGGB241	ACTAATGAACTGTTCTGCTTCC	GTTTATAAGGTAGGCATGGTTG	Fine mapping, SSR marker
WGGB242	TACAGTTTGGGCGTTGTGAT	ATGCAGTCCTGCCCAATCTC	Fine mapping, STS marker
WGGB243	TCGTCACCAAGACCTTTG	TTGTCAGAAGTGCCTACTGC	Fine mapping, dCAPS (Hpa Ⅰ) marker
WGGB244	TCTCGACTGGGGACAATACC	TTCTTCCACGAACCGACTCT	Fine mapping, SNP marker
WGGB245	CAAGAACGAGGATAACATTG	CCGTAAGCTTCTGTTCTCAT	Fine mapping, SSR marker
WTK3-RACE5'		CAATGTTTTTCTCAAACCCTTAAGTTG	5' RACE
WTK3-RACE3'	GAGTATAGAGGAAAACTGGAGGGAG		3' RACE
CNL-RACE5'	GTCAACTTGGCCCAGCCACGACTATG		5' RACE
CNL-RACE3'		CATAAACCGGAGGAACCACAAAGAGC	3' RACE
WTK3-CDS	ATCTACAAGACGAGAAGCAAG	ACGACACATAACAGTTCAGATC	Full CDS
CNL-CDS	ACACACGGGACAGGATAAGG	GGAACACATATAGGCAGTCG	Full CDS
WTK3-AT-F1/R1	GTGCACACCGTTCATTTTAT	CCACTTGTAGCCAAGTTTTG	Haplotype analysis of WTK3 in Aegilops tauschii
WTK3-AT-F2/R2	TCCAAGTGGGTTACGTATTG	CTTGATATCGAAAGCACCAG	Haplotype analysis of WTK3 in Aegilops tauschii
WTK3-AT-F3/R3	CTGGAGTGCCAAGTCTATTG	TAACTTGGGTGTTCATCGTC	Haplotype analysis of WTK3 in Aegilops tauschii
WTK3-AT-F4/R4	CTCAAGCACGATAGATTTGG	CTGTGAGGATGTAAGCTTGG	Haplotype analysis of WTK3 in Aegilops tauschii
WTK3-AT-F5/R5	TGTATGGAGATTGGGCAGGA	TGTAGGTCTCTTTTCAGGGTTTG	Haplotype analysis of WTK3 in Aegilops tauschii
WTK3-AT-F6/R6	TGCGCAGGCTTAGATTACCT	AGAGCAAGATGGCGCATAGT	Haplotype analysis of WTK3 in Aegilops tauschii
WTK3-HW-F1/R1	TGGTTCAATTTCAAGCTCCA	GACCTTGCATTTCCTAAGACCC	Haplotype analysis of WTK3 in Hexaploid wheat and mutants analysis
WTK3-HW-F2/R2	AGCCTATTAATTCCCTACTCACC	CTCGGTACAGATCAACCTTTCTCT	Haplotype analysis of WTK3 in Hexaploid wheat and mutants analysis
WTK3-HW-F3/R3	TCAAGAAGTCTCACTTTCGTTT	GAAAACATGTTGCCCGTC	Haplotype analysis of WTK3 in Hexaploid wheat and mutants analysis
WTK3-HW-F4/R4	ATGAATCTCGAATTGGCCG	ATGAGCAATGAGCTCACACG	Haplotype analysis of WTK3 in Hexaploid wheat and mutants analysis
WTK3-HW-F5/R5	AACCGGCGGATCATGCATAG	ATTTTAACTCCCATGTGCTCCACT	Haplotype analysis of WTK3 in Hexaploid wheat and mutants analysis
WTK3-HW-F6/R6	TGTGTTCTGCACAATCTCAAGGT	TCTGGGAGAGACCGAAGTCATAG	Haplotype analysis of WTK3 in Hexaploid wheat and mutants analysis
WTK3-HW-F7/R7	AGCCTTTCGAGATGACAAGC	GCTTCCGTTGTGCATATATTCA	Haplotype analysis of WTK3 in Hexaploid wheat and mutants analysis
WTK3-HW-F8/R8	CCGAACAACTGCTCTGCTAT	CCCTGTGATGCGTGGTAT	Haplotype analysis of WTK3 in Hexaploid wheat and mutants analysis
WTK3-HW-F9/R9	TCTTGCTGTTGTATGACTTTGT	CGGTCAATAGACTTGCTAAACG	Haplotype analysis of WTK3 in Hexaploid wheat and mutants analysis
WTK3-HW-F10/R10	TCAAATGTATTGCTGGACCAC	ACGAGCTTTGTCGAACCATC	Haplotype analysis of WTK3 in Hexaploid wheat and mutants analysis
CNL-F1/R1	CCTTCTCTCTCACAACCCTAACAT	ACCATACCCTAACTTGCAGTCA	Analysis of CNL in mutants
CNL-F2/R2	GCCAACTCAATTCCGACAGAT	CTTTGACGTTGAACATGAGTTTC	Analysis of CNL in mutants
CNL-F3/R3	AGAAGAGGACGAGCTTATGGAG	CCAGGAATGGCTTCCAGGGT	Analysis of CNL in mutants
CNL-F4/R4	CTGAGCGTGATGTCAACGTG	CACTGGAGGATAAATTCCAAGC	Analysis of CNL in mutants
InDel-WTK3	GTCAAGGTTTGCATCATCTC	TACCTGCCAAAGTGATGCTG	Haplotype analysis, STS marker
STS-Pm24	TATGGTGTCATTTAAGGCTGAG	TTTCTCACATCCTCATCAAACC	MAS/Transgenic identify, STS marker
CNL-COMTrans	CGAATTCGAGCTCGGTACCCGGGGCAATTGTTGCTGTAATGTGGTA	AAACGACGGCCAGTGCCAAGCTCTTCCACTCCAAGGGTACTGACCT	*ProCNL:CNL construction*
WTK3-COMTransF1/R1	AATTCGAGCTCGGTACCCGGGTTGTTGGCCAATCGTTATCA	AGACCCCTAGTTTCCTGATGATA	*ProWTK3:WTK3 construction*
WTK3-COMTransF2/R2	TATCATCAGGAAACTAGGGGTCT	GCATATATTCATAGCAGAGCAGTTG	*ProWTK3:WTK3 construction*
WTK3-COMTransF3/R3	CAACTGCTCTGCTATGAATATATGC	CTGTGATGCGTGGTATGTAGTTG	*ProWTK3:WTK3 construction*
WTK3-COMTransF4/R4	CAACTACATACCACGCATCACAG	TGCAGGTCGACTCTAGAGGATCGTCCTTCATTGATGTGTTCC	*ProWTK3:WTK3 construction*
WTK3-OETrans	TCTAGAGGATCCCCGGGTACCATGGGCGGATACGAGTTCCA	TTCGAGCTCTCTAGAACTAGTTTACTTCTTCCATCCGAAGTGT	*ProUb1:WTK3 construction*
WTK3#1-OETransF1/R1	TCTAGAGGATCCCCGGGTACCATGGGCGGATACGAGTTCCA	CATCAAACCATCCCTTCCCGATC	*ProUb1:WTK3#1 construction*
WTK3#1-OETransF2/R2	GATCGGGAAGGGATGGTTTGATG	TTCGAGCTCTCTAGAACTAGTTTACTTCTTCCATCCGAAGTGT	*ProUb1:WTK3#1 construction*
WTK3#2-OETransF1/R1	TCTAGAGGATCCCCGGGTACCATGGGCGGATACGAGTTCCA	CATCAAACCATTTCTTCCCGATC	*ProUb1:WTK3#2 construction*
WTK3#2-OETransF2/R2	GATCGGGAAGAAATGGTTTGATG	TTCGAGCTCTCTAGAACTAGTTTACTTCTTCCATCCGAAGTGT	*ProUb1:WTK3#2 construction*
WTK3#3-OETransF1/R1	TCTAGAGGATCCCCGGGTACCATGGGCGGATACGAGTTCCA	CACATCCTCATCAAACCCGATCAATAACTC	*ProUb1:WTK3#3 construction*
WTK3#3-OETransF2/R2	GAGTTATTGATCGGGTTTGATGAGGATGTG	TTCGAGCTCTCTAGAACTAGTTTACTTCTTCCATCCGAAGTGT	*ProUb1:WTK3#3 construction*
RLK1-Exp	AGCACATGTTCTTCACGGAT	ACGCTCCTTCTTTTACCAACT	Expression analysis
WTK3-Exp	TTATGGGTCAAGGATCTCGTGT	TGTGATGCGTGGTATGTAGTTG	Expression analysis
HP-Exp	TGTTTGCAAAATGAAGGACC	ACAGCTCTAAGAGAAGGAACC	Expression analysis

CNL-Exp	TTCACACCTGACCACTCTGT	GGAAACCTCATCAATCTGCT	Expression analysis
RLK2-Exp	CCAATGAGCTTTGAAAAATCG	TTGGCACATGTGCTACAAAC	Expression analysis
RP-Exp	CACCAGGTCAGTATACAAAATA	CATTGCTCTGATGTATCATCTT	Expression analysis
PR1-Exp	CTGGAGCACGAAGCTGCAG	CGAGTGCTGGAGCTTGCAGT	Expression analysis
PR2-Exp	CTCGACATCGGTAACGACCAG	GCGGCGATGTACTTGATGTTC	Expression analysis
PR3-Exp	AGAGATAAGCAAGGCCACGTC	GGTTGCTCACCAGGTCCTTC	Expression analysis
PR4-Exp	CGAGGATCGTGGACCAGTG	GTCGACGAACTGGTAGTTGACG	Expression analysis
PR5-Exp	ACAGCTACGCCAAGGACGAC	CGCGTCCTAATCTAAGGGCAG	Expression analysis
PR9-Exp	GAGATTCCACAGATGCAAACGAG	GGAGGCCCTTGTTTCTGAATG	Expression analysis
ACTIN	GTTGGTGATGAGGCCCAATC	GTGCTACACGGAGCTCATTG	Expression analysis
COM-CNLV	TCGTCACCAAGACCTTTG	TTGTCAGAAGTGCCTACTGC	Transgenic identify

Supplementary Table 3 Powdery mildew reactions of the T1 transgenic plants to *Bgt* isolate E09

		Trangenic positive plants			Trangenic negative plants	
Construct	T1 transgenic family	No. of positive plants	No. of resistant plants	No. of susceptible plants	No. of negative plants	No. of susceptible plants
ProWTK3:WTK3	WTK3-COM1	16	16	0	4	4
	WTK3-COM2	11	11	0	9	9
	WTK3-COM3	14	14	0	2	2
	WTK3-COM4	17	17	0	0	0
	WTK3-COM5	9	9	0	5	5
	WTK3-COM6	16	16	0	2	2
	WTK3-COM7	16	16	0	2	2
ProCNL:CNL	CNL-COM1	12	0	12	5	5
	CNL-COM2	9	0	9	3	3
	CNL-COM3	12	0	12	2	2
	CNL-COM4	13	0	13	4	4
	CNL-COM5	11	0	11	5	5
	CNL-COM6	14	0	14	4	4
	CNL-COM7	8	0	8	3	3
	CNL-COM8	15	0	15	2	2
	CNL-COM9	12	0	12	6	6
	CNL-COM10	14	0	14	5	5
	CNL-COM11	11	0	11	3	3
	CNL-COM12	12	0	12	4	4
	CNL-COM13	9	0	9	3	3
	CNL-COM14	13	0	13	5	5
ProUbi:WTK3	WTK3-OE1	17	17	0	5	5
	WTK3-OE2	13	13	0	3	3
	WTK3-OE3	14	14	0	2	2
	WTK3-OE4	10	10	0	3	3
	WTK3-OE5	9	9	0	6	6
	WTK3-OE6	10	10	0	5	5
	WTK3-OE7	6	6	0	5	5
	WTK3-OE8	9	9	0	4	4
	WTK3-OE9	12	12	0	0	0
	WTK3-OE10	12	12	0	3	3
	WTK3-OE11	11	11	0	5	5
	WTK3-OE12	16	16	0	0	0
ProUbi:WTK3#1	WTK3#1-OE1	11	0	11	4	4
	WTK3#1-OE2	14	0	14	4	4
	WTK3#1-OE3	16	0	16	3	3
	WTK3#1-OE4	9	0	9	4	4
	WTK3#1-OE5	12	0	12	5	5
	WTK3#1-OE6	11	0	11	3	3
	WTK3#1-OE7	15	0	15	4	4
	WTK3#1-OE8	13	0	13	2	2
	WTK3#1-OE9	12	0	12	3	3
	WTK3#1-OE10	16	0	16	3	3
	WTK3#1-OE11	14	0	14	5	5
	WTK3#1-OE12	17	0	17	3	3
	WTK3#1-OE13	11	0	11	4	4
ProUbi:WTK3#2	WTK3#2-OE1	14	0	14	3	3
	WTK3#2-OE2	15	0	15	5	5
	WTK3#2-OE3	14	0	14	4	4
	WTK3#2-OE4	16	0	16	4	4
	WTK3#2-OE5	17	0	17	2	2
	WTK3#2-OE6	11	0	11	4	4
	WTK3#2-OE7	14	0	14	3	3
	WTK3#2-OE8	12	0	12	6	6
	WTK3#2-OE9	13	0	13	3	3
	WTK3#2-OE10	12	0	12	5	5
	WTK3#2-OE11	13	0	13	3	3
	WTK3#3-OE1	12	0	12	3	3
	WTK3#3-OE2	13	0	13	4	4

ProUbi:WTK3#3	WTK3#3-OE3	12	0	12	3	3
	WTK3#3-OE4	15	0	15	3	3
	WTK3#3-OE5	12	0	12	4	4

Supplementary Table 4 Molecular characterization of the *WTK3* EMS mutants.

Mutant	Mutation type	Position	Exon	Codon change	Amino acid change	Infection Type
Mut129	Missense	7073	7	GCA→ACA	A600T	3
Mut410	Nonsense	5970	5	TGG→TGA	W429* stop codon	4
Mut477	Missense	9848	10	CCT→TCT	P835S	4
Mut614	Missense	9192	8	GGG→AGG	G718R	4
Mut988	Missense	5848	5	GGC→AGC	G389S	4
Mut1091	Nonsense	6117	5	TGG→TGA	W478* stop codon	4
Mut1106	Splice site	5780	Intron 4/Exon 5	G→A	Frameshift	4
Mut1174	Missense	9075	8	GGG→AGG	G679R	4
Mut1269	Missense	5797	5	GAG→AAG	E372K	4
Mut1322	Missense	6010	5	GGG→AGG	G443R	3
Mut1365	Missense	9846	10	AGA→AAA	R834K	3
