# Supplementary material for: A rare gain of function mutation in a wheat tandem kinase confers resistance to powdery mildew
Source: Nat Commun. 2020 Feb 3;11:680. doi: 10.1038/s41467-020-14294-0 (PMC6997164; doi:10.1038/s41467-020-14294-0)
Supplement: Supplementary file 4 — Description of Additional Supplementary Files [file 41467_2020_14294_MOESM4_ESM.pdf]

Descriptions of Additional Supplementary Files

File name: Supplementary Data 1
Description: *WTK3* alternative transcript variants

File name: Supplementary Data 2
Description: Genomic DNA and CDS alignments of *WTK3* gene in Chinese wheat landraces CYC, BHL, HLT, and HMM

File name: Supplementary Data 3
Description: Genomic DNA alignments of *WTK3* gene in 25 common wheat and *Ae. tauschii* accessions

File name: Supplementary Data 4
Description: Sequence variations in the 632 bp genomic region containing the 6-bp deletion amplified by InDel-WTK3 in 1,069 *Aegilops tauschii* and hexaploid wheat accessions
