# Supplementary material for: A rare gain of function mutation in a wheat tandem kinase confers resistance to powdery mildew
Source: Nat Commun. 2020 Feb 3;11:680. doi: 10.1038/s41467-020-14294-0 (PMC6997164; doi:10.1038/s41467-020-14294-0)
Supplement: Supplementary file 5 — Supplementary Data 1 [file 41467_2020_14294_MOESM5_ESM.pdf]

a

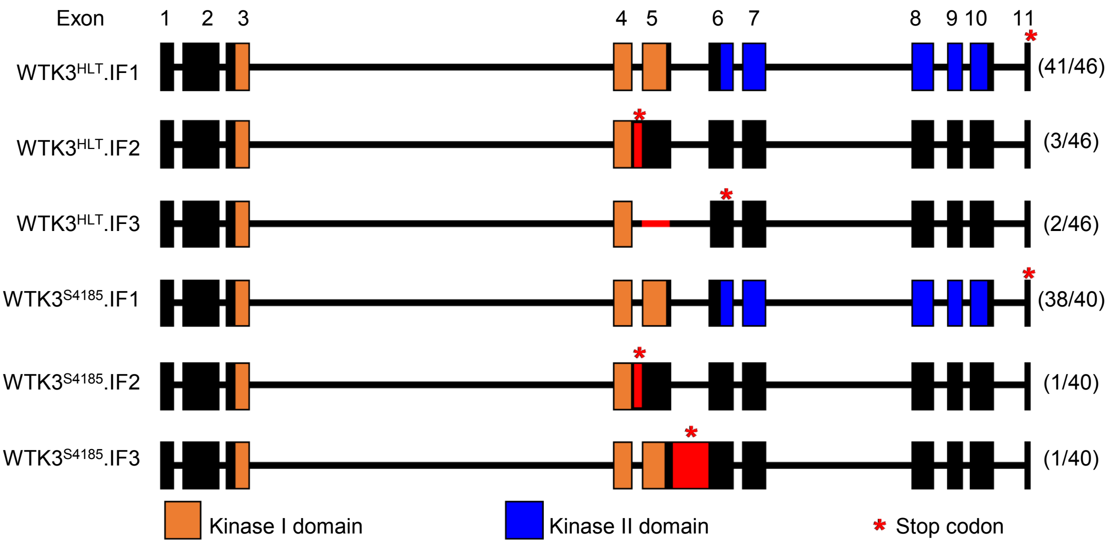
Exon
1
2
3
4
5
6
7
8
9
10
11
WTK3HLT.IF1
(41/46)
WTK3HLT.IF2
(3/46)
WTK3HLT.IF3
(2/46)
WTK3S4185.IF1
(38/40)
WTK3S4185.IF2
(1/40)
WTK3S4185.IF3
(1/40)
Kinase I domain
Kinase II domain
Stop codon

b

```
                1        10        20        30        40        50        60
WTK3HLT.IF1     ATGGGCGGATACGAGTTCCAGAGGGCGGAGCTAGATGCACTGGAAGGCGTCGTACGCGAT
Frame 1          M  G  G  Y  E  F  Q  R  A  E  L  D  A  L  E  G  V  V  R  D
WTK3HLT.IF2     ATGGGCGGATACGAGTTCCAGAGGGCGGAGCTAGATGCACTGGAAGGCGTCGTACGCGAT
Frame 1          M  G  G  Y  E  F  Q  R  A  E  L  D  A  L  E  G  V  V  R  D
WTK3HLT.IF3     ATGGGCGGATACGAGTTCCAGAGGGCGGAGCTAGATGCACTGGAAGGCGTCGTACGCGAT
Frame 1          M  G  G  Y  E  F  Q  R  A  E  L  D  A  L  E  G  V  V  R  D
WTK3S4185.IF1   ATGGGCGGATACGAGTTCCAGAGGGCGGAGCTAGATGCACTGGAAGGCGTCGTACGCGAT
Frame 1          M  G  G  Y  E  F  Q  R  A  E  L  D  A  L  E  G  V  V  R  D
WTK3S4185.IF2   ATGGGCGGATACGAGTTCCAGAGGGCGGAGCTAGATGCACTGGAAGGCGTCGTACGCGAT
Frame 1          M  G  G  Y  E  F  Q  R  A  E  L  D  A  L  E  G  V  V  R  D
WTK3S4185.IF3   ATGGGCGGATACGAGTTCCAGAGGGCGGAGCTAGATGCACTGGAAGGCGTCGTACGCGAT
Frame 1          M  G  G  Y  E  F  Q  R  A  E  L  D  A  L  E  G  V  V  R  D

                         70        80        90       100       110       120
WTK3HLT.IF1     CCAACTGCGGAGCCAATGAGTCTGACGTTGCCGCTTCTCAGGCACATAACAAATGATTTC
Frame 1          P  T  A  E  P  M  S  L  T  L  P  L  L  R  H  I  T  N  D  F
WTK3HLT.IF2     CCAACTGCGGAGCCAATGAGTCTGACGTTGCCGCTTCTCAGGCACATAACAAATGATTTC
Frame 1          P  T  A  E  P  M  S  L  T  L  P  L  L  R  H  I  T  N  D  F
WTK3HLT.IF3     CCAACTGCGGAGCCAATGAGTCTGACGTTGCCGCTTCTCAGGCACATAACAAATGATTTC
Frame 1          P  T  A  E  P  M  S  L  T  L  P  L  L  R  H  I  T  N  D  F
WTK3S4185.IF1   CCAACTGCGGAGCCAATGAGTCTGACGTTGCCGCTTCTCAGGCACATAACAAATGATTTC
Frame 1          P  T  A  E  P  M  S  L  T  L  P  L  L  R  H  I  T  N  D  F
WTK3S4185.IF2   CCAACTGCGGAGCCAATGAGTCTGACGTTGCCGCTTCTCAGGCACATAACAAATGATTTC
Frame 1          P  T  A  E  P  M  S  L  T  L  P  L  L  R  H  I  T  N  D  F
WTK3S4185.IF3   CCAACTGCGGAGCCAATGAGTCTGACGTTGCCGCTTCTCAGGCACATAACAAATGATTTC
Frame 1          P  T  A  E  P  M  S  L  T  L  P  L  L  R  H  I  T  N  D  F

                        130       140       150       160       170       180
WTK3HLT.IF1     TCCCCTGAATTTGAAATTAGTAAAGATGATTCTGCAGTGGTTTACCTGGGGGTGCTTCCA
Frame 1          S  P  E  F  E  I  S  K  D  D  S  A  V  V  Y  L  G  V  L  P
WTK3HLT.IF2     TCCCCTGAATTTGAAATTAGTAAAGATGATTCTGCAGTGGTTTACCTGGGGGTGCTTCCA
Frame 1          S  P  E  F  E  I  S  K  D  D  S  A  V  V  Y  L  G  V  L  P
WTK3HLT.IF3     TCCCCTGAATTTGAAATTAGTAAAGATGATTCTGCAGTGGTTTACCTGGGGGTGCTTCCA
Frame 1          S  P  E  F  E  I  S  K  D  D  S  A  V  V  Y  L  G  V  L  P
WTK3S4185.IF1   TCCCCTGAATTTGAAATTAGTAAAGATGATTCTGCAGTGGTTTACCTGGGGGTGCTTCCA
Frame 1          S  P  E  F  E  I  S  K  D  D  S  A  V  V  Y  L  G  V  L  P
WTK3S4185.IF2   TCCCCTGAATTTGAAATTAGTAAAGATGATTCTGCAGTGGTTTACCTGGGGGTGCTTCCA
Frame 1          S  P  E  F  E  I  S  K  D  D  S  A  V  V  Y  L  G  V  L  P
WTK3S4185.IF3   TCCCCTGAATTTGAAATTAGTAAAGATGATTCTGCAGTGGTTTACCTGGGGGTGCTTCCA
Frame 1          S  P  E  F  E  I  S  K  D  D  S  A  V  V  Y  L  G  V  L  P

                        190       200       210       220       230       240
WTK3HLT.IF1     AGTGGGTTCCGTGTTGCTGTCAAGAAGTCTCACTTTCGTTTTTGCTTGGATGATGAAGAT
Frame 1          S  G  F  R  V  A  V  K  K  S  H  F  R  F  C  L  D  D  E  D
WTK3HLT.IF2     AGTGGGTTCCGTGTTGCTGTCAAGAAGTCTCACTTTCGTTTTTGCTTGGATGATGAAGAT
Frame 1          S  G  F  R  V  A  V  K  K  S  H  F  R  F  C  L  D  D  E  D
WTK3HLT.IF3     AGTGGGTTCCGTGTTGCTGTCAAGAAGTCTCACTTTCGTTTTTGCTTGGATGATGAAGAT
Frame 1          S  G  F  R  V  A  V  K  K  S  H  F  R  F  C  L  D  D  E  D
WTK3S4185.IF1   AGTGGGTTCCGTGTTGCTGTCAAGAAGTCTCACTTTCGTTTTTGCTTGGATGATGAAGAT
Frame 1          S  G  F  R  V  A  V  K  K  S  H  F  R  F  C  L  D  D  E  D
WTK3S4185.IF2   AGTGGGTTCCGTGTTGCTGTCAAGAAGTCTCACTTTCGTTTTTGCTTGGATGATGAAGAT
Frame 1          S  G  F  R  V  A  V  K  K  S  H  F  R  F  C  L  D  D  E  D
WTK3S4185.IF3   AGTGGGTTCCGTGTTGCTGTCAAGAAGTCTCACTTTCGTTTTTGCTTGGATGATGAAGAT
Frame 1          S  G  F  R  V  A  V  K  K  S  H  F  R  F  C  L  D  D  E  D
```

```
                          250       260       270       280       290       300
WTK3HLT.IF1      GCATTCACAAATGAAGTTTCTATTGCAATGAAGGCTGCTCATAAGAACACAGTGCGAGTC
 Frame 1          A  F  T  N  E  V  S  I  A  M  K  A  A  H  K  N  T  V  R  V
WTK3HLT.IF2      GCATTCACAAATGAAGTTTCTATTGCAATGAAGGCTGCTCATAAGAACACAGTGCGAGTC
 Frame 1          A  F  T  N  E  V  S  I  A  M  K  A  A  H  K  N  T  V  R  V
WTK3HLT.IF3      GCATTCACAAATGAAGTTTCTATTGCAATGAAGGCTGCTCATAAGAACACAGTGCGAGTC
 Frame 1          A  F  T  N  E  V  S  I  A  M  K  A  A  H  K  N  T  V  R  V
WTK3S4185.IF1    GCATTCACAAATGAAGTTTCTATTGCAATGAAGGCTGCTCATAAGAACACAGTGCGAGTC
 Frame 1          A  F  T  N  E  V  S  I  A  M  K  A  A  H  K  N  T  V  R  V
WTK3S4185.IF2    GCATTCACAAATGAAGTTTCTATTGCAATGAAGGCTGCTCATAAGAACACAGTGCGAGTC
 Frame 1          A  F  T  N  E  V  S  I  A  M  K  A  A  H  K  N  T  V  R  V
WTK3S4185.IF3    GCATTCACAAATGAAGTTTCTATTGCAATGAAGGCTGCTCATAAGAACACAGTGCGAGTC
 Frame 1          A  F  T  N  E  V  S  I  A  M  K  A  A  H  K  N  T  V  R  V

                          310       320       330       340       350       360
WTK3HLT.IF1      ATAGGCTACTGTCATCACACGCATGAGCAAATTGCCGAATACGAAGGAAAACAAGTTTTC
 Frame 1          I  G  Y  C  H  H  T  H  E  Q  I  A  E  Y  E  G  K  Q  V  F
WTK3HLT.IF2      ATAGGCTACTGTCATCACACGCATGAGCAAATTGCCGAATACGAAGGAAAACAAGTTTTC
 Frame 1          I  G  Y  C  H  H  T  H  E  Q  I  A  E  Y  E  G  K  Q  V  F
WTK3HLT.IF3      ATAGGCTACTGTCATCACACGCATGAGCAAATTGCCGAATACGAAGGAAAACAAGTTTTC
 Frame 1          I  G  Y  C  H  H  T  H  E  Q  I  A  E  Y  E  G  K  Q  V  F
WTK3S4185.IF1    ATAGGCTACTGTCATCACACGCATGAGCAAATTGCCGAATACGAAGGAAAACAAGTTTTC
 Frame 1          I  G  Y  C  H  H  T  H  E  Q  I  A  E  Y  E  G  K  Q  V  F
WTK3S4185.IF2    ATAGGCTACTGTCATCACACGCATGAGCAAATTGCCGAATACGAAGGAAAACAAGTTTTC
 Frame 1          I  G  Y  C  H  H  T  H  E  Q  I  A  E  Y  E  G  K  Q  V  F
WTK3S4185.IF3    ATAGGCTACTGTCATCACACGCATGAGCAAATTGCCGAATACGAAGGAAAACAAGTTTTC
 Frame 1          I  G  Y  C  H  H  T  H  E  Q  I  A  E  Y  E  G  K  Q  V  F

                          370       380       390       400       410       420
WTK3HLT.IF1      GCAGAGGTCAGAGAAAGGTTGATCTGTACCGAGTATGTGCCTAACGGACCCCTTAGTGGA
 Frame 1          A  E  V  R  E  R  L  I  C  T  E  Y  V  P  N  G  P  L  S  G
WTK3HLT.IF2      GCAGAGGTCAGAGAAAGGTTGATCTGTACCGAGTATGTGCCTAACGGACCCCTTAGTGGA
 Frame 1          A  E  V  R  E  R  L  I  C  T  E  Y  V  P  N  G  P  L  S  G
WTK3HLT.IF3      GCAGAGGTCAGAGAAAGGTTGATCTGTACCGAGTATGTGCCTAACGGACCCCTTAGTGGA
 Frame 1          A  E  V  R  E  R  L  I  C  T  E  Y  V  P  N  G  P  L  S  G
WTK3S4185.IF1    GCAGAGGTCAGAGAAAGGTTGATCTGTACCGAGTATGTGCCTAACGGACCCCTTAGTGGA
 Frame 1          A  E  V  R  E  R  L  I  C  T  E  Y  V  P  N  G  P  L  S  G
WTK3S4185.IF2    GCAGAGGTCAGAGAAAGGTTGATCTGTACCGAGTATGTGCCTAACGGACCCCTTAGTGGA
 Frame 1          A  E  V  R  E  R  L  I  C  T  E  Y  V  P  N  G  P  L  S  G
WTK3S4185.IF3    GCAGAGGTCAGAGAAAGGTTGATCTGTACCGAGTATGTGCCTAACGGACCCCTTAGTGGA
 Frame 1          A  E  V  R  E  R  L  I  C  T  E  Y  V  P  N  G  P  L  S  G

                          430       440       450       460       470       480
WTK3HLT.IF1      CATATCGAAGGTAAGATATGTGCGCAAATGGATGGATACGAGTTCCAGAGGGCAGAACTA
 Frame 1          H  I  E  G  K  I  C  A  Q  M  D  G  Y  E  F  Q  R  A  E  L
WTK3HLT.IF2      CATATCGAAGGTAAGATATGTGCGCAAATGGATGGATACGAGTTCCAGAGGGCAGAACTA
 Frame 1          H  I  E  G  K  I  C  A  Q  M  D  G  Y  E  F  Q  R  A  E  L
WTK3HLT.IF3      CATATCGAAGGTAAGATATGTGCGCAAATGGATGGATACGAGTTCCAGAGGGCAGAACTA
 Frame 1          H  I  E  G  K  I  C  A  Q  M  D  G  Y  E  F  Q  R  A  E  L
WTK3S4185.IF1    CATATCGAAGGTAAGATATGTGCGCAAATGGATGGATACGAGTTCCAGAGGGCAGAACTA
 Frame 1          H  I  E  G  K  I  C  A  Q  M  D  G  Y  E  F  Q  R  A  E  L
WTK3S4185.IF2    CATATCGAAGGTAAGATATGTGCGCAAATGGATGGATACGAGTTCCAGAGGGCAGAACTA
 Frame 1          H  I  E  G  K  I  C  A  Q  M  D  G  Y  E  F  Q  R  A  E  L
WTK3S4185.IF3    CATATCGAAGGTAAGATATGTGCGCAAATGGATGGATACGAGTTCCAGAGGGCAGAACTA
 Frame 1          H  I  E  G  K  I  C  A  Q  M  D  G  Y  E  F  Q  R  A  E  L
```

```
                        490       500       510       520       530       540
WTK3HLT.IF1     GATGCACTAGAACGCGTCGTACGCGATACAAGTGCGGAGCCAATGAGTCTGACGTTGCCG
Frame 1          D  A  L  E  R  V  V  R  D  T  S  A  E  P  M  S  L  T  L  P
WTK3HLT.IF2     GATGCACTAGAACGCGTCGTACGCGATACAAGTGCGGAGCCAATGAGTCTGACGTTGCCG
Frame 1          D  A  L  E  R  V  V  R  D  T  S  A  E  P  M  S  L  T  L  P
WTK3HLT.IF3     GATGCACTAGAACGCGTCGTACGCGATACAAGTGCGGAGCCAATGAGTCTGACGTTGCCG
Frame 1          D  A  L  E  R  V  V  R  D  T  S  A  E  P  M  S  L  T  L  P
WTK3S4185.IF1   GATGCACTAGAACGCGTCGTACGCGATACAAGTGCGGAGCCAATGAGTCTGACGTTGCCG
Frame 1          D  A  L  E  R  V  V  R  D  T  S  A  E  P  M  S  L  T  L  P
WTK3S4185.IF2   GATGCACTAGAACGCGTCGTACGCGATACAAGTGCGGAGCCAATGAGTCTGACGTTGCCG
Frame 1          D  A  L  E  R  V  V  R  D  T  S  A  E  P  M  S  L  T  L  P
WTK3S4185.IF3   GATGCACTAGAACGCGTCGTACGCGATACAAGTGCGGAGCCAATGAGTCTGACGTTGCCG
Frame 1          D  A  L  E  R  V  V  R  D  T  S  A  E  P  M  S  L  T  L  P

                        550       560       570       580       590       600
WTK3HLT.IF1     CTTCTCAGGCACATAACAAATGATTTCTCCGATGAATCTCGAATTGGCCGAGGTGGATTC
Frame 1          L  L  R  H  I  T  N  D  F  S  D  E  S  R  I  G  R  G  G  F
WTK3HLT.IF2     CTTCTCAGGCACATAACAAATGATTTCTCCGATGAATCTCGAATTGGCCGAGGTGGATTC
Frame 1          L  L  R  H  I  T  N  D  F  S  D  E  S  R  I  G  R  G  G  F
WTK3HLT.IF3     CTTCTCAGGCACATAACAAATGATTTCTCCGATGAATCTCGAATTGGCCGAGGTGGATTC
Frame 1          L  L  R  H  I  T  N  D  F  S  D  E  S  R  I  G  R  G  G  F
WTK3S4185.IF1   CTTCTCAGGCACATAACAAATGATTTCTCCGATGAATCTCGAATTGGCCGAGGTGGATTC
Frame 1          L  L  R  H  I  T  N  D  F  S  D  E  S  R  I  G  R  G  G  F
WTK3S4185.IF2   CTTCTCAGGCACATAACAAATGATTTCTCCGATGAATCTCGAATTGGCCGAGGTGGATTC
Frame 1          L  L  R  H  I  T  N  D  F  S  D  E  S  R  I  G  R  G  G  F
WTK3S4185.IF3   CTTCTCAGGCACATAACAAATGATTTCTCCGATGAATCTCGAATTGGCCGAGGTGGATTC
Frame 1          L  L  R  H  I  T  N  D  F  S  D  E  S  R  I  G  R  G  G  F

                        610       620       630       640       650       660
WTK3HLT.IF1     GCAGTGGTTTACCTGGGGGTGCTTCCAAGTGGGTTACGTATTGCTGTTAAGAGGCTTAGC
Frame 1          A  V  V  Y  L  G  V  L  P  S  G  L  R  I  A  V  K  R  L  S
WTK3HLT.IF2     GCAGTGGTTTACCTGGGGGTGCTTCCAAGTGGGTTACGTATTGCTGTTAAGAGGCTTAGC
Frame 1          A  V  V  Y  L  G  V  L  P  S  G  L  R  I  A  V  K  R  L  S
WTK3HLT.IF3     GCAGTGGTTTACCTGGGGGTGCTTCCAAGTGGGTTACGTATTGCTGTTAAGAGGCTTAGC
Frame 1          A  V  V  Y  L  G  V  L  P  S  G  L  R  I  A  V  K  R  L  S
WTK3S4185.IF1   GCAGTGGTTTACCTGGGGGTGCTTCCAAGTGGGTTACGTATTGCTGTTAAGAGGCTTAGC
Frame 1          A  V  V  Y  L  G  V  L  P  S  G  L  R  I  A  V  K  R  L  S
WTK3S4185.IF2   GCAGTGGTTTACCTGGGGGTGCTTCCAAGTGGGTTACGTATTGCTGTTAAGAGGCTTAGC
Frame 1          A  V  V  Y  L  G  V  L  P  S  G  L  R  I  A  V  K  R  L  S
WTK3S4185.IF3   GCAGTGGTTTACCTGGGGGTGCTTCCAAGTGGGTTACGTATTGCTGTTAAGAGGCTTAGC
Frame 1          A  V  V  Y  L  G  V  L  P  S  G  L  R  I  A  V  K  R  L  S

                        670       680       690       700       710       720
WTK3HLT.IF1     AATATTGCTTATATGAACGAAAGTGCATTTCAAAATGAAGTGTTTATCACAATGAAGGCC
Frame 1          N  I  A  Y  M  N  E  S  A  F  Q  N  E  V  F  I  T  M  K  A
WTK3HLT.IF2     AATATTGCTTATATGAACGAAAGTGCATTTCAAAATGAAGTGTTTATCACAATGAAGGCC
Frame 1          N  I  A  Y  M  N  E  S  A  F  Q  N  E  V  F  I  T  M  K  A
WTK3HLT.IF3     AATATTGCTTATATGAACGAAAGTGCATTTCAAAATGAAGTGTTTATCACAATGAAGGCC
Frame 1          N  I  A  Y  M  N  E  S  A  F  Q  N  E  V  F  I  T  M  K  A
WTK3S4185.IF1   AATATTGCTTATATGAACGAAAGTGCATTTCAAAATGAAGTGTTTATCACAATGAAGGCC
Frame 1          N  I  A  Y  M  N  E  S  A  F  Q  N  E  V  F  I  T  M  K  A
WTK3S4185.IF2   AATATTGCTTATATGAACGAAAGTGCATTTCAAAATGAAGTGTTTATCACAATGAAGGCC
Frame 1          N  I  A  Y  M  N  E  S  A  F  Q  N  E  V  F  I  T  M  K  A
WTK3S4185.IF3   AATATTGCTTATATGAACGAAAGTGCATTTCAAAATGAAGTGTTTATCACAATGAAGGCC
Frame 1          N  I  A  Y  M  N  E  S  A  F  Q  N  E  V  F  I  T  M  K  A
```

```
                          730       740       750       760       770       780
WTK3HLT.IF1      ACTCACAAGAACACAGTGCGATTCATGGGCTACTGTAGTCAAATACAAGGTAAACTCATC
Frame 1           T  H  K  N  T  V  R  F  M  G  Y  C  S  Q  I  Q  G  K  L  I
WTK3HLT.IF2      ACTCACAAGAACACAGTGCGATTCATGGGCTACTGTAGTCAAATACAAGGTAAACTCATC
Frame 1           T  H  K  N  T  V  R  F  M  G  Y  C  S  Q  I  Q  G  K  L  I
WTK3HLT.IF3      ACTCACAAGAACACAGTGCGATTCATGGGCTACTGTAGTCAAATACAAGGTAAACTCATC
Frame 1           T  H  K  N  T  V  R  F  M  G  Y  C  S  Q  I  Q  G  K  L  I
WTK3S4185.IF1    ACTCACAAGAACACAGTGCGATTCATGGGCTACTGTAGTCAAATACAAGGTAAACTCATC
Frame 1           T  H  K  N  T  V  R  F  M  G  Y  C  S  Q  I  Q  G  K  L  I
WTK3S4185.IF2    ACTCACAAGAACACAGTGCGATTCATGGGCTACTGTAGTCAAATACAAGGTAAACTCATC
Frame 1           T  H  K  N  T  V  R  F  M  G  Y  C  S  Q  I  Q  G  K  L  I
WTK3S4185.IF3    ACTCACAAGAACACAGTGCGATTCATGGGCTACTGTAGTCAAATACAAGGTAAACTCATC
Frame 1           T  H  K  N  T  V  R  F  M  G  Y  C  S  Q  I  Q  G  K  L  I

                          790       800       810       820       830       840
WTK3HLT.IF1      GAACACGACGGGCAACATGTTTTCGCACAGCTCGAGGAAAGGTTGATCTGTGTGGAATAT
Frame 1           E  H  D  G  Q  H  V  F  A  Q  L  E  E  R  L  I  C  V  E  Y
WTK3HLT.IF2      GAACACGACGGGCAACATGTTTTCGCACAGCTCGAGGAAAGGTTGATCTGTGTGGAATAT
Frame 1           E  H  D  G  Q  H  V  F  A  Q  L  E  E  R  L  I  C  V  E  Y
WTK3HLT.IF3      GAACACGACGGGCAACATGTTTTCGCACAGCTCGAGGAAAGGTTGATCTGTGTGGAATAT
Frame 1           E  H  D  G  Q  H  V  F  A  Q  L  E  E  R  L  I  C  V  E  Y
WTK3S4185.IF1    GAACACGACGGGCAACATGTTTTCGCACAGCTCGAGGAAAGGTTGATCTGTGTGGAATAT
Frame 1           E  H  D  G  Q  H  V  F  A  Q  L  E  E  R  L  I  C  V  E  Y
WTK3S4185.IF2    GAACACGACGGGCAACATGTTTTCGCACAGCTCGAGGAAAGGTTGATCTGTGTGGAATAT
Frame 1           E  H  D  G  Q  H  V  F  A  Q  L  E  E  R  L  I  C  V  E  Y
WTK3S4185.IF3    GAACACGACGGGCAACATGTTTTCGCACAGCTCGAGGAAAGGTTGATCTGTGTGGAATAT
Frame 1           E  H  D  G  Q  H  V  F  A  Q  L  E  E  R  L  I  C  V  E  Y

                          850       860       870       880       890       900
WTK3HLT.IF1      GCGCCTAAAGGAACCCTTGATGCACATATCGGTGACTATGGTGAACTTGACTGGAACCAG
Frame 1           A  P  K  G  T  L  D  A  H  I  G  D  Y  G  E  L  D  W  N  Q
WTK3HLT.IF2      GCGCCTAAAGGAACCCTTGATGCACATATCGGTGACTATGGTGAACTTGACTGGAACCAG
Frame 1           A  P  K  G  T  L  D  A  H  I  G  D  Y  G  E  L  D  W  N  Q
WTK3HLT.IF3      GCGCCTAAAGGAACCCTTGATGCACATATCGGTGACTATGGTGAACTTGACTGGAACCAG
Frame 1           A  P  K  G  T  L  D  A  H  I  G  D  Y  G  E  L  D  W  N  Q
WTK3S4185.IF1    GCGCCTAAAGGAACCCTTGATGCACATATCGGTGACTATGGTGAACTTGACTGGAACCAG
Frame 1           A  P  K  G  T  L  D  A  H  I  G  D  Y  G  E  L  D  W  N  Q
WTK3S4185.IF2    GCGCCTAAAGGAACCCTTGATGCACATATCGGTGACTATGGTGAACTTGACTGGAACCAG
Frame 1           A  P  K  G  T  L  D  A  H  I  G  D  Y  G  E  L  D  W  N  Q
WTK3S4185.IF3    GCGCCTAAAGGAACCCTTGATGCACATATCGGTGACTATGGTGAACTTGACTGGAACCAG
Frame 1           A  P  K  G  T  L  D  A  H  I  G  D  Y  G  E  L  D  W  N  Q

                          910       920       930       940       950       960
WTK3HLT.IF1      CGTTATCAAATTCTAAAAGGAATTTGTCAAGGTTTGCATCATCTCCATGACGAAATGCAC
Frame 1           R  Y  Q  I  L  K  G  I  C  Q  G  L  H  H  L  H  D  E  M  H
WTK3HLT.IF2      CGTTATCAAATTCTAAAAGGAATTTGTCAAGGTTTGCATCATCTCCATGACGAAATGCAC
Frame 1           R  Y  Q  I  L  K  G  I  C  Q  G  L  H  H  L  H  D  E  M  H
WTK3HLT.IF3      CGTTATCAAATTCTAAAAGGAATTTGTCAAGGTTTGCATCATCTCCATGACGAAATGCAC
Frame 1           R  Y  Q  I  L  K  G  I  C  Q  G  L  H  H  L  H  D  E  M  H
WTK3S4185.IF1    CGTTATCAAATTCTAAAAGGAATTTGTCAAGGTTTGCATCATCTCCATGACGAAATGCAC
Frame 1           R  Y  Q  I  L  K  G  I  C  Q  G  L  H  H  L  H  D  E  M  H
WTK3S4185.IF2    CGTTATCAAATTCTAAAAGGAATTTGTCAAGGTTTGCATCATCTCCATGACGAAATGCAC
Frame 1           R  Y  Q  I  L  K  G  I  C  Q  G  L  H  H  L  H  D  E  M  H
WTK3S4185.IF3    CGTTATCAAATTCTAAAAGGAATTTGTCAAGGTTTGCATCATCTCCATGACGAAATGCAC
Frame 1           R  Y  Q  I  L  K  G  I  C  Q  G  L  H  H  L  H  D  E  M  H
```

```
                    970       980       990       1,000     1,010     1,020
WTK3HLT.IF1     GTTTTTCATGGAGATATCAAACCAGCCAATATATTAATAGGGGATAACCTTGTGCCTAAA
 Frame 1         V  F  H  G  D  I  K  P  A  N  I  L  I  G  D  N  L  V  P  K
WTK3HLT.IF2     GTTTTTCATGGAGATATCAAACCAGCCAATATATTAATAGGGGATAACCTTGTGCCTAAA
 Frame 1         V  F  H  G  D  I  K  P  A  N  I  L  I  G  D  N  L  V  P  K
WTK3HLT.IF3     GTTTTTCATGGAGATATCAAACCAGCCAATATATTAATAGGGGATAACCTTGTGCCTAAA
 Frame 1         V  F  H  G  D  I  K  P  A  N  I  L  I  G  D  N  L  V  P  K
WTK3S4185.IF1   GTTTTTCATGGAGATATCAAACCAGCCAATATATTAATAGGGGATAACCTTGTGCCTAAA
 Frame 1         V  F  H  G  D  I  K  P  A  N  I  L  I  G  D  N  L  V  P  K
WTK3S4185.IF2   GTTTTTCATGGAGATATCAAACCAGCCAATATATTAATAGGGGATAACCTTGTGCCTAAA
 Frame 1         V  F  H  G  D  I  K  P  A  N  I  L  I  G  D  N  L  V  P  K
WTK3S4185.IF3   GTTTTTCATGGAGATATCAAACCAGCCAATATATTAATAGGGGATAACCTTGTGCCTAAA
 Frame 1         V  F  H  G  D  I  K  P  A  N  I  L  I  G  D  N  L  V  P  K

                    1,030     1,040     1,050     1,060     1,070     1,080
WTK3HLT.IF1     ATCTATGACTTCGGTCTCTCCCAGATGTTTGAAGAAGAAGAAACGGAACGTATTGTTGAA
 Frame 1         I  Y  D  F  G  L  S  Q  M  F  E  E  E  E  T  E  R  I  V  E
WTK3HLT.IF2     ATCTATGACTTCGGTCTCTCCCAGATGTTTGAAGAAGAAGAAACGGAACGTATTGTTGAA
 Frame 1         I  Y  D  F  G  L  S  Q  M  F  E  E  E  E  T  E  R  I  V  E
WTK3HLT.IF3     ATCTATGACTTCGGTCTCTCCCAGATGTTTGAAGAAGAAGAAACGGAACGTATTGTTGAA
 Frame 1         I  Y  D  F  G  L  S  Q  M  F  E  E  E  E  T  E  R  I  V  E
WTK3S4185.IF1   ATCTATGACTTCGGTCTCTCCCAGATGTTTGAAGAAGAAGAAACGGAACGTATTGTTGAA
 Frame 1         I  Y  D  F  G  L  S  Q  M  F  E  E  E  E  T  E  R  I  V  E
WTK3S4185.IF2   ATCTATGACTTCGGTCTCTCCCAGATGTTTGAAGAAGAAGAAACGGAACGTATTGTTGAA
 Frame 1         I  Y  D  F  G  L  S  Q  M  F  E  E  E  E  T  E  R  I  V  E
WTK3S4185.IF3   ATCTATGACTTCGGTCTCTCCCAGATGTTTGAAGAAGAAGAAACGGAACGTATTGTTGAA
 Frame 1         I  Y  D  F  G  L  S  Q  M  F  E  E  E  E  T  E  R  I  V  E

                    1,090     1,100     1,110     1,120     1,130     1,140
WTK3HLT.IF1     AATATCGCCGGAACATT-------------------------------------------
 Frame 1         N  I  A  G  T  F-------------------------------------------
WTK3HLT.IF2     AATATCGCCGGAACATTGTAAGCTAACCCGTTCCTCTTTGTTATTTTGTTTTGTTTCTGC
 Frame 1         N  I  A  G  T  L  *  A  N  P  F  L  F  V  I  L  F  C  F  C
WTK3HLT.IF3     AATATCGCCGGAACAT--------------------------------------------
 Frame 1         N  I  A  G  T  --------------------------------------------
WTK3S4185.IF1   AATATCGCCGGAACATT-------------------------------------------
 Frame 1         N  I  A  G  T  F-------------------------------------------
WTK3S4185.IF2   AATATCGCCGGAACATTGTAAGCTAACCCGTTCCTCTTTGTTATTTTGTTTTGTTTCTGC
 Frame 1         N  I  A  G  T  L  *  A  N  P  F  L  F  V  I  L  F  C  F  C
WTK3S4185.IF3   AATATCGCCGGAACATT-------------------------------------------
 Frame 1         N  I  A  G  T  F-------------------------------------------

                    1,150     1,160     1,170     1,180     1,190     1,200
WTK3HLT.IF1     ------------------------------------------------------------
 Frame 1        ------------------------------------------------------------
WTK3HLT.IF2     TGACACAGGTACTTCGATAACCAAAATGCATTAGAGTTAGTCGGCCGATTGCCTCACATC
 Frame 1         *  H  R  Y  F  D  N  Q  N  A  L  E  L  V  G  R  L  P  H  I
WTK3HLT.IF3     ------------------------------------------------------------
 Frame 1        ------------------------------------------------------------
WTK3S4185.IF1   ------------------------------------------------------------
 Frame 1        ------------------------------------------------------------
WTK3S4185.IF2   TGACACAGGTACTTCGATAACCAAAATGCATTAGAGTTAGTCGGCCGATTGCCTCACATC
 Frame 1         *  H  R  Y  F  D  N  Q  N  A  L  E  L  V  G  R  L  P  H  I
WTK3S4185.IF3   ------------------------------------------------------------
 Frame 1        ------------------------------------------------------------
```

```
                          1,210     1,220     1,230     1,240     1,250     1,260
WTK3HLT.IF1     --------------CGGATATATGGCACCGGAGTTTTGTACTAATAATATGGTGTCATTT
 Frame 1        --------------  G  Y  M  A  P  E  F  C  T  N  N  M  V  S  F
WTK3HLT.IF2     TAACATATGTGCAGCGGATATATGGCACCGGAGTTTTGTACTAATAATATGGTGTCATTT
 Frame 1         *  H  M  C  S  G  Y  M  A  P  E  F  C  T  N  N  M  V  S  F
WTK3HLT.IF3     ------------------------------------------------------------
 Frame 1        ------------------------------------------------------------
WTK3S4185.IF1   --------------CGGATATATGGCACCGGAGTTTTGTACTAATAATATGGTGTCATTT
 Frame 1        --------------  G  Y  M  A  P  E  F  C  T  N  N  M  V  S  F
WTK3S4185.IF2   TAACATATGTGCAGCGGATATATGGCACCGGAGTTTTGTACTAATAATATGGTGTCATTT
 Frame 1         *  H  M  C  S  G  Y  M  A  P  E  F  C  T  N  N  M  V  S  F
WTK3S4185.IF3   --------------CGGATATATGGCACCGGAGTTTTGTACTAATAATATGGTGTCATTT
 Frame 1        --------------  G  Y  M  A  P  E  F  C  T  N  N  M  V  S  F

                          1,270     1,280     1,290     1,300     1,310     1,320
WTK3HLT.IF1     AAGGCTGAGATATACAGTTTGGGCGTTGTGATCGGGGAGTTATTGATCGGGAAG------
 Frame 1         K  A  E  I  Y  S  L  G  V  V  I  G  E  L  L  I  G  K ------
WTK3HLT.IF2     AAGGCTGAGATATACAGTTTGGGCGTTGTGATCGGGGAGTTATTGATCGGGAAG------
 Frame 1         K  A  E  I  Y  S  L  G  V  V  I  G  E  L  L  I  G  K ------
WTK3HLT.IF3     ------------------------------------------------------------
 Frame 1        ------------------------------------------------------------
WTK3S4185.IF1   AAGGCTGAGATATACAGTTTGGGCGTTGTGATCGGGGAGTTATTGATCGGGAAGAAAGGA
 Frame 1         K  A  E  I  Y  S  L  G  V  V  I  G  E  L  L  I  G  K  K  G
WTK3S4185.IF2   AAGGCTGAGATATACAGTTTGGGCGTTGTGATCGGGGAGTTATTGATCGGGAAGAAAGGA
 Frame 1         K  A  E  I  Y  S  L  G  V  V  I  G  E  L  L  I  G  K  K  G
WTK3S4185.IF3   AAGGCTGAGATATACAGTTTGGGCGTTGTGATCGGGGAGTTATTGATCGGGAAGAAAGGA
 Frame 1         K  A  E  I  Y  S  L  G  V  V  I  G  E  L  L  I  G  K  K  G

                          1,330     1,340     1,350     1,360     1,370     1,380
WTK3HLT.IF1     TGGTTTGATGAGGATGTGAGAAAACTATTTGTACAGCAACTTAAGGGTTTGAGAAAAACA
 Frame 1         W  F  D  E  D  V  R  K  L  F  V  Q  Q  L  K  G  L  R  K  T
WTK3HLT.IF2     TGGTTTGATGAGGATGTGAGAAAACTATTTGTACAGCAACTTAAGGGTTTGAGAAAAACA
 Frame 1         W  F  D  E  D  V  R  K  L  F  V  Q  Q  L  K  G  L  R  K  T
WTK3HLT.IF3     ------------------------------------------------------------
 Frame 1        ------------------------------------------------------------
WTK3S4185.IF1   TGGTTTGATGAGGATGTGAGAAAACTATTTGTACAGCAACTTAAGGGTTTGAGAAAAACA
 Frame 1         W  F  D  E  D  V  R  K  L  F  V  Q  Q  L  K  G  L  R  K  T
WTK3S4185.IF2   TGGTTTGATGAGGATGTGAGAAAACTATTTGTACAGCAACTTAAGGGTTTGAGAAAAACA
 Frame 1         W  F  D  E  D  V  R  K  L  F  V  Q  Q  L  K  G  L  R  K  T
WTK3S4185.IF3   TGGTTTGATGAGGATGTGAGAAAACTATTTGTACAGCAACTTAAGGGTTTGAGAAAAACA
 Frame 1         W  F  D  E  D  V  R  K  L  F  V  Q  Q  L  K  G  L  R  K  T

                          1,390     1,400     1,410     1,420     1,430     1,440
WTK3HLT.IF1     TTGGTAAAAGAAGGAGCGTTTTCATCATGGGAAAACAAATACCACCAAGTTAGAACATGT
 Frame 1         L  V  K  E  G  A  F  S  S  W  E  N  K  Y  H  Q  V  R  T  C
WTK3HLT.IF2     TTGGTAAAAGAAGGAGCGTTTTCATCATGGGAAAACAAATACCACCAAGTTAGAACATGT
 Frame 1         L  V  K  E  G  A  F  S  S  W  E  N  K  Y  H  Q  V  R  T  C
WTK3HLT.IF3     ------------------------------------------------------------
 Frame 1        ------------------------------------------------------------
WTK3S4185.IF1   TTGGTAAAAGAAGGAGCGTTTTCATCATGGGAAAACAAATACCACCAAGTTAGAACATGT
 Frame 1         L  V  K  E  G  A  F  S  S  W  E  N  K  Y  H  Q  V  R  T  C
WTK3S4185.IF2   TTGGTAAAAGAAGGAGCGTTTTCATCATGGGAAAACAAATACCACCAAGTTAGAACATGT
 Frame 1         L  V  K  E  G  A  F  S  S  W  E  N  K  Y  H  Q  V  R  T  C
WTK3S4185.IF3   TTGGTAAAAGAAGGAGCGTTTTCATCATGGGAAAACAAATACCACCAAGTTAGAACATGT
 Frame 1         L  V  K  E  G  A  F  S  S  W  E  N  K  Y  H  Q  V  R  T  C
```

```
                     1,450     1,460     1,470     1,480     1,490     1,500
WTK3HLT.IF1     ATGGAGATTGGGCAGGACTGCATAGACCCCAACCCACATAAAAGGCCCACTTTGTTGGAG
Frame 1          M  E  I  G  Q  D  C  I  D  P  N  P  H  K  R  P  T  L  L  E 
WTK3HLT.IF2     ATGGAGATTGGGCAGGACTGCATAGACCCCAACCCACATAAAAGGCCCACTTTGTTGGAG
Frame 1          M  E  I  G  Q  D  C  I  D  P  N  P  H  K  R  P  T  L  L  E 
WTK3HLT.IF3     ------------------------------------------------------------
Frame 1         ------------------------------------------------------------
WTK3S4185.IF1   ATGGAGATTGGGCAGGACTGCATAGACCCCAACCCACATAAAAGGCCCACTTTGTTGGAG
Frame 1          M  E  I  G  Q  D  C  I  D  P  N  P  H  K  R  P  T  L  L  E 
WTK3S4185.IF2   ATGGAGATTGGGCAGGACTGCATAGACCCCAACCCACATAAAAGGCCCACTTTGTTGGAG
Frame 1          M  E  I  G  Q  D  C  I  D  P  N  P  H  K  R  P  T  L  L  E 
WTK3S4185.IF3   ATGGAGATTGGGCAGGACTGCATAGACCCCAACCCACATAAAAGGCCCACTTTGTTGGAG
Frame 1          M  E  I  G  Q  D  C  I  D  P  N  P  H  K  R  P  T  L  L  E 

                     1,510     1,520     1,530     1,540     1,550     1,560
WTK3HLT.IF1     ATTATCCAGCGGCTTAATGAAGCGGAAGATATGAACTATTCTGCAGCATCACTTTGG---
Frame 1          I  I  Q  R  L  N  E  A  E  D  M  N  Y  S  A  A  S  L  W ---
WTK3HLT.IF2     ATTATCCAGCGGCTTAATGAAGCGGAAGATATGAACTATTCTGCAGCATCACTTTGG---
Frame 1          I  I  Q  R  L  N  E  A  E  D  M  N  Y  S  A  A  S  L  W ---
WTK3HLT.IF3     ------------------------------------------------------------
Frame 1         ------------------------------------------------------------
WTK3S4185.IF1   ATTATCCAGCGGCTTAATGAAGCGGAAGATATGAACTATTCTGCAGCATCACTTTGG---
Frame 1          I  I  Q  R  L  N  E  A  E  D  M  N  Y  S  A  A  S  L  W ---
WTK3S4185.IF2   ATTATCCAGCGGCTTAATGAAGCGGAAGATATGAACTATTCTGCAGCATCACTTTGG---
Frame 1          I  I  Q  R  L  N  E  A  E  D  M  N  Y  S  A  A  S  L  W ---
WTK3S4185.IF3   ATTATCCAGCGGCTTAATGAAGCGGAAGATATGAACTATTCTGCAGCATCACTTTGGCAG
Frame 1          I  I  Q  R  L  N  E  A  E  D  M  N  Y  S  A  A  S  L  W  Q 

                     1,570     1,580     1,590     1,600     1,610     1,620
WTK3HLT.IF1     ------------------------------------------------------------
Frame 1         ------------------------------------------------------------
WTK3HLT.IF2     ------------------------------------------------------------
Frame 1         ------------------------------------------------------------
WTK3HLT.IF3     ------------------------------------------------------------
Frame 1         ------------------------------------------------------------
WTK3S4185.IF1   ------------------------------------------------------------
Frame 1         ------------------------------------------------------------
WTK3S4185.IF2   ------------------------------------------------------------
Frame 1         ------------------------------------------------------------
WTK3S4185.IF3   GTATGGAAAATCGAAAGCATAAGGAAACAACTGTTCTTTATCTACCATTTCACTTTAGCT
Frame 1          V  W  K  I  E  S  I  R  K  Q  L  F  F  I  Y  H  F  T  L  A 

                     1,630     1,640     1,650     1,660     1,670     1,680
WTK3HLT.IF1     ------------------------------------------------------------
Frame 1         ------------------------------------------------------------
WTK3HLT.IF2     ------------------------------------------------------------
Frame 1         ------------------------------------------------------------
WTK3HLT.IF3     ------------------------------------------------------------
Frame 1         ------------------------------------------------------------
WTK3S4185.IF1   ------------------------------------------------------------
Frame 1         ------------------------------------------------------------
WTK3S4185.IF2   ------------------------------------------------------------
Frame 1         ------------------------------------------------------------
WTK3S4185.IF3   AACGTACTGTTTGAGAAGAATCAGTCTTAGCCAAGCTTACATCCTCACAGACACAGTACA
Frame 1          N  V  L  F  E  K  N  Q  S  *  P  S  L  H  P  H  R  H  S  T 
```

```
                    1,690     1,700     1,710     1,720     1,730     1,740
WTK3HLT.IF1     ------------------------------------------------------------
Frame 1         ------------------------------------------------------------
WTK3HLT.IF2     ------------------------------------------------------------
Frame 1         ------------------------------------------------------------
WTK3HLT.IF3     ------------------------------------------------------------
Frame 1         ------------------------------------------------------------
WTK3S4185.IF1   ------------------------------------------------------------
Frame 1         ------------------------------------------------------------
WTK3S4185.IF2   ------------------------------------------------------------
Frame 1         ------------------------------------------------------------
WTK3S4185.IF3   GAAGTACATTTCTTTTCTTTAGCCATTACGATTAACATAAAGAGTTCACAAAGCCATCTC
Frame 1          E  V  H  F  F  S  L  A  I  T  I  N  I  K  S  S  Q  S  H  L

                    1,750     1,760     1,770     1,780     1,790     1,800
WTK3HLT.IF1     ------------------------------------------------------------
Frame 1         ------------------------------------------------------------
WTK3HLT.IF2     ------------------------------------------------------------
Frame 1         ------------------------------------------------------------
WTK3HLT.IF3     ------------------------------------------------------------
Frame 1         ------------------------------------------------------------
WTK3S4185.IF1   ------------------------------------------------------------
Frame 1         ------------------------------------------------------------
WTK3S4185.IF2   ------------------------------------------------------------
Frame 1         ------------------------------------------------------------
WTK3S4185.IF3   CAACGAAAGATATGTGCTAATTAAACTAAATTGGAAGAGAAAATACATAACAAAGCGAAG
Frame 1          Q  R  K  I  C  A  N  *  T  K  L  E  E  K  I  H  N  K  A  K

                    1,810     1,820     1,830     1,840     1,850     1,860
WTK3HLT.IF1     ------------------------------------------------------------
Frame 1         ------------------------------------------------------------
WTK3HLT.IF2     ------------------------------------------------------------
Frame 1         ------------------------------------------------------------
WTK3HLT.IF3     ------------------------------------------------------------
Frame 1         ------------------------------------------------------------
WTK3S4185.IF1   ------------------------------------------------------------
Frame 1         ------------------------------------------------------------
WTK3S4185.IF2   ------------------------------------------------------------
Frame 1         ------------------------------------------------------------
WTK3S4185.IF3   ACACCATGTAAATTAGAGAAGAAACAAACTCTTGGTACTCCTTCAGCGAGGGAAGACACC
Frame 1          T  P  C  K  L  E  K  K  Q  T  L  G  T  P  S  A  R  E  D  T

                    1,870     1,880     1,890     1,900     1,910     1,920
WTK3HLT.IF1     ------------------------------------------------------------
Frame 1         ------------------------------------------------------------
WTK3HLT.IF2     ------------------------------------------------------------
Frame 1         ------------------------------------------------------------
WTK3HLT.IF3     ------------------------------------------------------------
Frame 1         ------------------------------------------------------------
WTK3S4185.IF1   ------------------------------------------------------------
Frame 1         ------------------------------------------------------------
WTK3S4185.IF2   ------------------------------------------------------------
Frame 1         ------------------------------------------------------------
WTK3S4185.IF3   TTGTCAACTTAGCAGAAACAAACTCTTGGTACTCCTTCAGCGAGGGAAGACACCTTGTCA
Frame 1          L  S  T  *  Q  K  Q  T  L  G  T  P  S  A  R  E  D  T  L  S
```

```
                      1,930     1,940     1,950     1,960     1,970     1,980
WTK3HLT.IF1     ------------------------------------------------------------
Frame 1         ------------------------------------------------------------
WTK3HLT.IF2     ------------------------------------------------------------
Frame 1         ------------------------------------------------------------
WTK3HLT.IF3     ------------------------------------------------------------
Frame 1         ------------------------------------------------------------
WTK3S4185.IF1   ------------------------------------------------------------
Frame 1         ------------------------------------------------------------
WTK3S4185.IF2   ------------------------------------------------------------
Frame 1         ------------------------------------------------------------
WTK3S4185.IF3   ACTTAGCAGAAACCTTACGACTCTAGTCCTACATGACATGTCTAAGAGAATATTGATGTC
Frame 1         T  *  Q  K  P  Y  D  S  S  P  T  *  H  V  *  E  N  I  D  V

                      1,990     2,000     2,010     2,020     2,030     2,040
WTK3HLT.IF1     -----------------------------------------CAGTCAGGAGACGAGGAAT
Frame 1         ----------------------------------------- Q  S  G  D  E  E
WTK3HLT.IF2     -----------------------------------------CAGTCAGGAGACGAGGAAT
Frame 1         ----------------------------------------- Q  S  G  D  E  E
WTK3HLT.IF3     -------------------------------------------TTCAGGAGACGAGGAAT
Frame 1         -------------------------------------------F  Q  E  T  R  N
WTK3S4185.IF1   -----------------------------------------CAGTCAGGAGACGAGGAAT
Frame 1         ----------------------------------------- Q  S  G  D  E  E
WTK3S4185.IF2   -----------------------------------------CAGTCAGGAGACGAGGAAT
Frame 1         ----------------------------------------- Q  S  G  D  E  E
WTK3S4185.IF3   ATGCATTGCATAATGCTTACATGGTAAGTTATATATAAGTGCAGTCAGGAGACGAGGAAT
Frame 1         M  H  C  I  M  L  T  W  *  V  I  Y  K  C  S  Q  E  T  R  N

                      2,050     2,060     2,070     2,080     2,090     2,100
WTK3HLT.IF1     CCGATTTATCGGATACAGAAGCTTTGGAGACAGAGACAACATCCGAGTTTCTTCCAAGTG
Frame 1         S  D  L  S  D  T  E  A  L  E  T  E  T  T  S  E  F  L  P  S
WTK3HLT.IF2     CCGATTTATCGGATACAGAAGCTTTGGAGACAGAGACAACATCCGAGTTTCTTCCAAGTG
Frame 1         S  D  L  S  D  T  E  A  L  E  T  E  T  T  S  E  F  L  P  S
WTK3HLT.IF3     CCGATTTATCGGATACAGAAGCTTTGGAGACAGAGACAACATCCGAGTTTCTTCCAAGTG
Frame 1          P  I  Y  R  I  Q  K  L  W  R  Q  R  Q  H  P  S  F  F  Q  V
WTK3S4185.IF1   CCGATTTATCGGATACAGAAGCTTTGGAGACAGAGACAACATCCGAGTTTCTTCCAAGTG
Frame 1         S  D  L  S  D  T  E  A  L  E  T  E  T  T  S  E  F  L  P  S
WTK3S4185.IF2   CCGATTTATCGGATACAGAAGCTTTGGAGACAGAGACAACATCCGAGTTTCTTCCAAGTG
Frame 1         S  D  L  S  D  T  E  A  L  E  T  E  T  T  S  E  F  L  P  S
WTK3S4185.IF3   CCGATTTATCGGATACAGAAGCTTTGGAGACAGAGACAACATCCGAGTTTCTTCCAAGTG
Frame 1          P  I  Y  R  I  Q  K  L  W  R  Q  R  Q  H  P  S  F  F  Q  V

                      2,110     2,120     2,130     2,140     2,150     2,160
WTK3HLT.IF1     ACGAAGAACCCGCCTCTGTGGGCAAGACCGGAGAAACAAGCACACAGGAGCCTGATAAAC
Frame 1         D  E  E  P  A  S  V  G  K  T  G  E  T  S  T  Q  E  P  D  K
WTK3HLT.IF2     ACGAAGAACCCGCCTCTGTGGGCAAGACCGGAGAAACAAGCACACAGGAGCCTGATAAAC
Frame 1         D  E  E  P  A  S  V  G  K  T  G  E  T  S  T  Q  E  P  D  K
WTK3HLT.IF3     ACGAAGAACCCGCCTCTGTGGGCAAGACCGGAGAAACAAGCACACAGGAGCCTGATAAAC
Frame 1          T  K  N  P  P  L  W  A  R  P  E  K  Q  A  H  R  S  L  I  N
WTK3S4185.IF1   ACGAAGAACCCGCCTCTGTGGGCAAGACCGGAGAAACAAGCACACAGGAGCCTGATAAAC
Frame 1         D  E  E  P  A  S  V  G  K  T  G  E  T  S  T  Q  E  P  D  K
WTK3S4185.IF2   ACGAAGAACCCGCCTCTGTGGGCAAGACCGGAGAAACAAGCACACAGGAGCCTGATAAAC
Frame 1         D  E  E  P  A  S  V  G  K  T  G  E  T  S  T  Q  E  P  D  K
WTK3S4185.IF3   ACGAAGAACCCGCCTCTGTGGGCAAGACCGGAGAAACAAGCACACAGGAGCCTGATAAAC
Frame 1          T  K  N  P  P  L  W  A  R  P  E  K  Q  A  H  R  S  L  I  N
```

```
                    2,170     2,180     2,190     2,200     2,210     2,220
WTK3HLT.IF1     CGGACCTAATAAGTAAGTTGCCAGCATCGGTGGACCTGTCTGACCTAAAAGTCCTGGAGA
Frame 1         P  D  L  I  S  K  L  P  A  S  V  D  L  S  D  L  K  V  L  E
WTK3HLT.IF2     CGGACCTAATAAGTAAGTTGCCAGCATCGGTGGACCTGTCTGACCTAAAAGTCCTGGAGA
Frame 1         P  D  L  I  S  K  L  P  A  S  V  D  L  S  D  L  K  V  L  E
WTK3HLT.IF3     CGGACCTAATAAGTAAGTTGCCAGCATCGGTGGACCTGTCTGACCTAAAAGTCCTGGAGA
Frame 1          R  T  *  *  V  S  C  Q  H  R  W  T  C  L  T  *  K  S  W  R
WTK3S4185.IF1   CGGACCTAATAAGTAAGTTGCCAGCATCGGTGGACCTGTCTGACCTAAAAGTCCTGGAGA
Frame 1         P  D  L  I  S  K  L  P  A  S  V  D  L  S  D  L  K  V  L  E
WTK3S4185.IF2   CGGACCTAATAAGTAAGTTGCCAGCATCGGTGGACCTGTCTGACCTAAAAGTCCTGGAGA
Frame 1         P  D  L  I  S  K  L  P  A  S  V  D  L  S  D  L  K  V  L  E
WTK3S4185.IF3   CGGACCTAATAAGTAAGTTGCCAGCATCGGTGGACCTGTCTGACCTAAAAGTCCTGGAGA
Frame 1          R  T  *  *  V  S  C  Q  H  R  W  T  C  L  T  *  K  S  W  R
```

```
                    2,230     2,240     2,250     2,260     2,270     2,280
WTK3HLT.IF1     AAATCACAGATGATTTTTCACACGAAAGAATAGTTGGGAAGGACGGTACATTCAAAGGTT
Frame 1         K  I  T  D  D  F  S  H  E  R  I  V  G  K  D  G  T  F  K  G
WTK3HLT.IF2     AAATCACAGATGATTTTTCACACGAAAGAATAGTTGGGAAGGACGGTACATTCAAAGGTT
Frame 1         K  I  T  D  D  F  S  H  E  R  I  V  G  K  D  G  T  F  K  G
WTK3HLT.IF3     AAATCACAGATGATTTTTCACACGAAAGAATAGTTGGGAAGGACGGTACATTCAAAGGTT
Frame 1          K  S  Q  M  I  F  H  T  K  E  *  L  G  R  T  V  H  S  K  V
WTK3S4185.IF1   AAATCACAGATGATTTTTCACACGAAAGAATAGTTGGGAAGGACGGTACATTCAAAGGTT
Frame 1         K  I  T  D  D  F  S  H  E  R  I  V  G  K  D  G  T  F  K  G
WTK3S4185.IF2   AAATCACAGATGATTTTTCACACGAAAGAATAGTTGGGAAGGACGGTACATTCAAAGGTT
Frame 1         K  I  T  D  D  F  S  H  E  R  I  V  G  K  D  G  T  F  K  G
WTK3S4185.IF3   AAATCACAGATGATTTTTCACACGAAAGAATAGTTGGGAAGGACGGTACATTCAAAGGTT
Frame 1          K  S  Q  M  I  F  H  T  K  E  *  L  G  R  T  V  H  S  K  V
```

```
                    2,290     2,300     2,310     2,320     2,330     2,340
WTK3HLT.IF1     GTCATAAGGCATTTGTTTATAAGGGTGACATTCCACTTAGAGAAATGATAGCCGTGAAGA
Frame 1         C  H  K  A  F  V  Y  K  G  D  I  P  L  R  E  M  I  A  V  K
WTK3HLT.IF2     GTCATAAGGCATTTGTTTATAAGGGTGACATTCCACTTAGAGAAATGATAGCCGTGAAGA
Frame 1         C  H  K  A  F  V  Y  K  G  D  I  P  L  R  E  M  I  A  V  K
WTK3HLT.IF3     GTCATAAGGCATTTGTTTATAAGGGTGACATTCCACTTAGAGAAATGATAGCCGTGAAGA
Frame 1          V  I  R  H  L  F  I  R  V  T  F  H  L  E  K  *  *  P  *  R
WTK3S4185.IF1   GTCATAAGGCATTTGTTTATAAGGGTGACATTCCACTTAGAGAAATGATAGCCGTGAAGA
Frame 1         C  H  K  A  F  V  Y  K  G  D  I  P  L  R  E  M  I  A  V  K
WTK3S4185.IF2   GTCATAAGGCATTTGTTTATAAGGGTGACATTCCACTTAGAGAAATGATAGCCGTGAAGA
Frame 1         C  H  K  A  F  V  Y  K  G  D  I  P  L  R  E  M  I  A  V  K
WTK3S4185.IF3   GTCATAAGGCATTTGTTTATAAGGGTGACATTCCACTTAGAGAAATGATAGCCGTGAAGA
Frame 1          V  I  R  H  L  F  I  R  V  T  F  H  L  E  K  *  *  P  *  R
```

```
                    2,350     2,360     2,370     2,380     2,390     2,400
WTK3HLT.IF1     GGTTAATTGGAGTGGAGATTCCATTTGAAAAGTTTAAGAGGGAAGCAGAACAGTTCATTA
Frame 1         R  L  I  G  V  E  I  P  F  E  K  F  K  R  E  A  E  Q  F  I
WTK3HLT.IF2     GGTTAATTGGAGTGGAGATTCCATTTGAAAAGTTTAAGAGGGAAGCAGAACAGTTCATTA
Frame 1         R  L  I  G  V  E  I  P  F  E  K  F  K  R  E  A  E  Q  F  I
WTK3HLT.IF3     GGTTAATTGGAGTGGAGATTCCATTTGAAAAGTTTAAGAGGGAAGCAGAACAGTTCATTA
Frame 1          G  *  L  E  W  R  F  H  L  K  S  L  R  G  K  Q  N  S  S  L
WTK3S4185.IF1   GGTTAATTGGAGTGGAGATTCCATTTGAAAAGTTTAAGAGGGAAGCAGAACAGTTCATTA
Frame 1         R  L  I  G  V  E  I  P  F  E  K  F  K  R  E  A  E  Q  F  I
WTK3S4185.IF2   GGTTAATTGGAGTGGAGATTCCATTTGAAAAGTTTAAGAGGGAAGCAGAACAGTTCATTA
Frame 1         R  L  I  G  V  E  I  P  F  E  K  F  K  R  E  A  E  Q  F  I
WTK3S4185.IF3   GGTTAATTGGAGTGGAGATTCCATTTGAAAAGTTTAAGAGGGAAGCAGAACAGTTCATTA
Frame 1          G  *  L  E  W  R  F  H  L  K  S  L  R  G  K  Q  N  S  S  L
```

```
                 2,410     2,420     2,430     2,440     2,450     2,460
WTK3HLT.IF1      GTCTCGATCATAAGAATATAGTAAAGGTTGCCAGCTACTGCCACGACCAGTCTAGAGGAC
 Frame 1         S  L  D  H  K  N  I  V  K  V  A  S  Y  C  H  D  Q  S  R  G
WTK3HLT.IF2      GTCTCGATCATAAGAATATAGTAAAGGTTGCCAGCTACTGCCACGACCAGTCTAGAGGAC
 Frame 1         S  L  D  H  K  N  I  V  K  V  A  S  Y  C  H  D  Q  S  R  G
WTK3HLT.IF3      GTCTCGATCATAAGAATATAGTAAAGGTTGCCAGCTACTGCCACGACCAGTCTAGAGGAC
 Frame 1          V  S  I  I  R  I  *  *  R  L  P  A  T  A  T  T  S  L  E  D
WTK3S4185.IF1    GTCTCGATCATAAGAATATAGTAAAGGTTGCCAGCTACTGCCACGACCAGTCTAGAGGAC
 Frame 1         S  L  D  H  K  N  I  V  K  V  A  S  Y  C  H  D  Q  S  R  G
WTK3S4185.IF2    GTCTCGATCATAAGAATATAGTAAAGGTTGCCAGCTACTGCCACGACCAGTCTAGAGGAC
 Frame 1         S  L  D  H  K  N  I  V  K  V  A  S  Y  C  H  D  Q  S  R  G
WTK3S4185.IF3    GTCTCGATCATAAGAATATAGTAAAGGTTGCCAGCTACTGCCACGACCAGTCTAGAGGAC
 Frame 1          V  S  I  I  R  I  *  *  R  L  P  A  T  A  T  T  S  L  E  D
```

```
                 2,470     2,480     2,490     2,500     2,510     2,520
WTK3HLT.IF1      ATAGACTGGTACAGTTCAAAGGAAAACCGCTACCACAACTCTTTAACGGTCCCGAACAAC
 Frame 1         H  R  L  V  Q  F  K  G  K  P  L  P  Q  L  F  N  G  P  E  Q
WTK3HLT.IF2      ATAGACTGGTACAGTTCAAAGGAAAACCGCTACCACAACTCTTTAACGGTCCCGAACAAC
 Frame 1         H  R  L  V  Q  F  K  G  K  P  L  P  Q  L  F  N  G  P  E  Q
WTK3HLT.IF3      ATAGACTGGTACAGTTCAAAGGAAAACCGCTACCACAACTCTTTAACGGTCCCGAACAAC
 Frame 1          I  D  W  Y  S  S  K  E  N  R  Y  H  N  S  L  T  V  P  N  N
WTK3S4185.IF1    ATAGACTGGTACAGTTCAAAGGAAAACCGCTACCACAACTCTTTAACGGTCCCGAACAAC
 Frame 1         H  R  L  V  Q  F  K  G  K  P  L  P  Q  L  F  N  G  P  E  Q
WTK3S4185.IF2    ATAGACTGGTACAGTTCAAAGGAAAACCGCTACCACAACTCTTTAACGGTCCCGAACAAC
 Frame 1         H  R  L  V  Q  F  K  G  K  P  L  P  Q  L  F  N  G  P  E  Q
WTK3S4185.IF3    ATAGACTGGTACAGTTCAAAGGAAAACCGCTACCACAACTCTTTAACGGTCCCGAACAAC
 Frame 1          I  D  W  Y  S  S  K  E  N  R  Y  H  N  S  L  T  V  P  N  N
```

```
                 2,530     2,540     2,550     2,560     2,570     2,580
WTK3HLT.IF1      TGCTCTGCTATGAATATATGCACAACGGAAGCCTTCGCGACTATCTTATGGGTCAAGGAT
 Frame 1         L  L  C  Y  E  Y  M  H  N  G  S  L  R  D  Y  L  M  G  Q  G
WTK3HLT.IF2      TGCTCTGCTATGAATATATGCACAACGGAAGCCTTCGCGACTATCTTATGGGTCAAGGAT
 Frame 1         L  L  C  Y  E  Y  M  H  N  G  S  L  R  D  Y  L  M  G  Q  G
WTK3HLT.IF3      TGCTCTGCTATGAATATATGCACAACGGAAGCCTTCGCGACTATCTTATGGGTCAAGGAT
 Frame 1          C  S  A  M  N  I  C  T  T  E  A  F  A  T  I  L  W  V  K  D
WTK3S4185.IF1    TGCTCTGCTATGAATATATGCACAACGGAAGCCTTCGCGACTATCTTATGGGTCAAGGAT
 Frame 1         L  L  C  Y  E  Y  M  H  N  G  S  L  R  D  Y  L  M  G  Q  G
WTK3S4185.IF2    TGCTCTGCTATGAATATATGCACAACGGAAGCCTTCGCGACTATCTTATGGGTCAAGGAT
 Frame 1         L  L  C  Y  E  Y  M  H  N  G  S  L  R  D  Y  L  M  G  Q  G
WTK3S4185.IF3    TGCTCTGCTATGAATATATGCACAACGGAAGCCTTCGCGACTATCTTATGGGTCAAGGAT
 Frame 1          C  S  A  M  N  I  C  T  T  E  A  F  A  T  I  L  W  V  K  D
```

```
                 2,590     2,600     2,610     2,620     2,630     2,640
WTK3HLT.IF1      CTCGTGTAATTGATTGGCAAATGCGCTACAAATTGATCAAAGGGACTTGCGCAGGCTTAC
 Frame 1         S  R  V  I  D  W  Q  M  R  Y  K  L  I  K  G  T  C  A  G  L
WTK3HLT.IF2      CTCGTGTAATTGATTGGCAAATGCGCTACAAATTGATCAAAGGGACTTGCGCAGGCTTAC
 Frame 1         S  R  V  I  D  W  Q  M  R  Y  K  L  I  K  G  T  C  A  G  L
WTK3HLT.IF3      CTCGTGTAATTGATTGGCAAATGCGCTACAAATTGATCAAAGGGACTTGCGCAGGCTTAC
 Frame 1          L  V  *  L  I  G  K  C  A  T  N  *  S  K  G  L  A  Q  A  Y
WTK3S4185.IF1    CTCGTGTAATTGATTGGCAAATGCGCTACAAATTGATCAAAGGGACTTGCGCAGGCTTAC
 Frame 1         S  R  V  I  D  W  Q  M  R  Y  K  L  I  K  G  T  C  A  G  L
WTK3S4185.IF2    CTCGTGTAATTGATTGGCAAATGCGCTACAAATTGATCAAAGGGACTTGCGCAGGCTTAC
 Frame 1         S  R  V  I  D  W  Q  M  R  Y  K  L  I  K  G  T  C  A  G  L
WTK3S4185.IF3    CTCGTGTAATTGATTGGCAAATGCGCTACAAATTGATCAAAGGGACTTGCGCAGGCTTAC
 Frame 1          L  V  *  L  I  G  K  C  A  T  N  *  S  K  G  L  A  Q  A  Y
```

```
                    2,650     2,660     2,670     2,680     2,690     2,700
WTK3HLT.IF1     ATTACCTTCACAAGGGCCGTGCAGGTTGTCCAATTGTTCATTTGAATTTAAGCCCGTCAA
Frame 1         H Y L H K G R A G C P I V H L N L S P S
WTK3HLT.IF2     ATTACCTTCACAAGGGCCGTGCAGGTTGTCCAATTGTTCATTTGAATTTAAGCCCGTCAA
Frame 1         H Y L H K G R A G C P I V H L N L S P S
WTK3HLT.IF3     ATTACCTTCACAAGGGCCGTGCAGGTTGTCCAATTGTTCATTTGAATTTAAGCCCGTCAA
Frame 1         I T F T R A V Q V V Q L F I * I * A R Q
WTK3S4185.IF1   ATTACCTTCACAAGGGCCGTGCAGGTTGTCCAATTGTTCATTTGAATTTAAGCCCGTCAA
Frame 1         H Y L H K G R A G C P I V H L N L S P S
WTK3S4185.IF2   ATTACCTTCACAAGGGCCGTGCAGGTTGTCCAATTGTTCATTTGAATTTAAGCCCGTCAA
Frame 1         H Y L H K G R A G C P I V H L N L S P S
WTK3S4185.IF3   ATTACCTTCACAAGGGCCGTGCAGGTTGTCCAATTGTTCATTTGAATTTAAGCCCGTCAA
Frame 1         I T F T R A V Q V V Q L F I * I * A R Q
```

```
                    2,710     2,720     2,730     2,740     2,750     2,760
WTK3HLT.IF1     ATGTATTGCTGGACCACAACTACATACCACGCATCACAGGGTTCGATTTTTCGAAGCTCA
Frame 1         N V L L D H N Y I P R I T G F D F S K L
WTK3HLT.IF2     ATGTATTGCTGGACCACAACTACATACCACGCATCACAGGGTTCGATTTTTCGAAGCTCA
Frame 1         N V L L D H N Y I P R I T G F D F S K L
WTK3HLT.IF3     ATGTATTGCTGGACCACAACTACATACCACGCATCACAGGGTTCGATTTTTCGAAGCTCA
Frame 1         M Y C W T T T T Y H A S Q G S I F R S S
WTK3S4185.IF1   ATGTATTGCTGGACCACAACTACATACCACGCATCACAGGGTTCGATTTTTCGAAGCTCA
Frame 1         N V L L D H N Y I P R I T G F D F S K L
WTK3S4185.IF2   ATGTATTGCTGGACCACAACTACATACCACGCATCACAGGGTTCGATTTTTCGAAGCTCA
Frame 1         N V L L D H N Y I P R I T G F D F S K L
WTK3S4185.IF3   ATGTATTGCTGGACCACAACTACATACCACGCATCACAGGGTTCGATTTTTCGAAGCTCA
Frame 1         M Y C W T T T T Y H A S Q G S I F R S S
```

```
                    2,770     2,780     2,790     2,800     2,810     2,820
WTK3HLT.IF1     TTGGTGAAAAGAACACCAAATCAGTGGTACTTAAGCTGAATGGACCCATAGCGTACCTGC
Frame 1         I G E K N T K S V V L K L N G P I A Y L
WTK3HLT.IF2     TTGGTGAAAAGAACACCAAATCAGTGGTACTTAAGCTGAATGGACCCATAGCGTACCTGC
Frame 1         I G E K N T K S V V L K L N G P I A Y L
WTK3HLT.IF3     TTGGTGAAAAGAACACCAAATCAGTGGTACTTAAGCTGAATGGACCCATAGCGTACCTGC
Frame 1         L V K R T P N Q W Y L S * M D P * R T C
WTK3S4185.IF1   TTGGTGAAAAGAACACCAAATCAGTGGTACTTAAGCTGAATGGACCCATAGCGTACCTGC
Frame 1         I G E K N T K S V V L K L N G P I A Y L
WTK3S4185.IF2   TTGGTGAAAAGAACACCAAATCAGTGGTACTTAAGCTGAATGGACCCATAGCGTACCTGC
Frame 1         I G E K N T K S V V L K L N G P I A Y L
WTK3S4185.IF3   TTGGTGAAAAGAACACCAAATCAGTGGTACTTAAGCTGAATGGACCCATAGCGTACCTGC
Frame 1         L V K R T P N Q W Y L S * M D P * R T C
```

```
                    2,830     2,840     2,850     2,860     2,870     2,880
WTK3HLT.IF1     CACCGGATTTCTTCCATTCGAAGGGTACTGATCTTAAATATCTTGCTACGGTAGATATAT
Frame 1         P P D F F H S K G T D L K Y L A T V D I
WTK3HLT.IF2     CACCGGATTTCTTCCATTCGAAGGGTACTGATCTTAAATATCTTGCTACGGTAGATATAT
Frame 1         P P D F F H S K G T D L K Y L A T V D I
WTK3HLT.IF3     CACCGGATTTCTTCCATTCGAAGGGTACTGATCTTAAATATCTTGCTACGGTAGATATAT
Frame 1         H R I S S I R R V L I L N I L L R * I Y
WTK3S4185.IF1   CACCGGATTTCTTCCATTCGAAGGGTACTGATCTTAAATATCTTGCTACGGTAGATATAT
Frame 1         P P D F F H S K G T D L K Y L A T V D I
WTK3S4185.IF2   CACCGGATTTCTTCCATTCGAAGGGTACTGATCTTAAATATCTTGCTACGGTAGATATAT
Frame 1         P P D F F H S K G T D L K Y L A T V D I
WTK3S4185.IF3   CACCGGATTTCTTCCATTCGAAGGGTACTGATCTTAAATATCTTGCTACGGTAGATATAT
Frame 1         H R I S S I R R V L I L N I L L R * I Y
```

```
                2,890     2,900     2,910     2,920     2,930     2,940
WTK3HLT.IF1     ACAGCTTGGGTCTTATGATTTTAGAAATCGCAACACAACAAGAGATCAAAGGCATCCATG
Frame 1         Y  S  L  G  L  M  I  L  E  I  A  T  Q  Q  E  I  K  G  I  H
WTK3HLT.IF2     ACAGCTTGGGTCTTATGATTTTAGAAATCGCAACACAACAAGAGATCAAAGGCATCCATG
Frame 1         Y  S  L  G  L  M  I  L  E  I  A  T  Q  Q  E  I  K  G  I  H
WTK3HLT.IF3     ACAGCTTGGGTCTTATGATTTTAGAAATCGCAACACAACAAGAGATCAAAGGCATCCATG
Frame 1          T  A  W  V  L  *  F  *  K  S  Q  H  N  K  R  S  K  A  S  M
WTK3S4185.IF1   ACAGCTTGGGTCTTATGATTTTAGAAATCGCAACACAACAAGAGATCAAAGGCATCCATG
Frame 1         Y  S  L  G  L  M  I  L  E  I  A  T  Q  Q  E  I  K  G  I  H
WTK3S4185.IF2   ACAGCTTGGGTCTTATGATTTTAGAAATCGCAACACAACAAGAGATCAAAGGCATCCATG
Frame 1         Y  S  L  G  L  M  I  L  E  I  A  T  Q  Q  E  I  K  G  I  H
WTK3S4185.IF3   ACAGCTTGGGTCTTATGATTTTAGAAATCGCAACACAACAAGAGATCAAAGGCATCCATG
Frame 1          T  A  W  V  L  *  F  *  K  S  Q  H  N  K  R  S  K  A  S  M

                2,950     2,960     2,970     2,980     2,990     3,000
WTK3HLT.IF1     GAGTGCTTATTAAGAGTATAGAGGAAAACTGGAGGGAGGAGTCACAAATAACACGGCTGT
Frame 1         G  V  L  I  K  S  I  E  E  N  W  R  E  E  S  Q  I  T  R  L
WTK3HLT.IF2     GAGTGCTTATTAAGAGTATAGAGGAAAACTGGAGGGAGGAGTCACAAATAACACGGCTGT
Frame 1         G  V  L  I  K  S  I  E  E  N  W  R  E  E  S  Q  I  T  R  L
WTK3HLT.IF3     GAGTGCTTATTAAGAGTATAGAGGAAAACTGGAGGGAGGAGTCACAAATAACACGGCTGT
Frame 1          E  C  L  L  R  V  *  R  K  T  G  G  R  S  H  K  *  H  G  C
WTK3S4185.IF1   GAGTGCTTATTAAGAGTATAGAGGAAAACTGGAGGGAGGAGTCACAAATAACACGGCTGT
Frame 1         G  V  L  I  K  S  I  E  E  N  W  R  E  E  S  Q  I  T  R  L
WTK3S4185.IF2   GAGTGCTTATTAAGAGTATAGAGGAAAACTGGAGGGAGGAGTCACAAATAACACGGCTGT
Frame 1         G  V  L  I  K  S  I  E  E  N  W  R  E  E  S  Q  I  T  R  L
WTK3S4185.IF3   GAGTGCTTATTAAGAGTATAGAGGAAAACTGGAGGGAGGAGTCACAAATAACACGGCTGT
Frame 1          E  C  L  L  R  V  *  R  K  T  G  G  R  S  H  K  *  H  G  C

                3,010     3,020     3,030     3,040     3,050     3,060
WTK3HLT.IF1     ATACCTCACTAGGGGCCGACGAGCTGCGGCAAGTAAAAATGTGCATTGATATTGGCCTAG
Frame 1         Y  T  S  L  G  A  D  E  L  R  Q  V  K  M  C  I  D  I  G  L
WTK3HLT.IF2     ATACCTCACTAGGGGCCGACGAGCTGCGGCAAGTAAAAATGTGCATTGATATTGGCCTAG
Frame 1         Y  T  S  L  G  A  D  E  L  R  Q  V  K  M  C  I  D  I  G  L
WTK3HLT.IF3     ATACCTCACTAGGGGCCGACGAGCTGCGGCAAGTAAAAATGTGCATTGATATTGGCCTAG
Frame 1          I  P  H  *  G  P  T  S  C  G  K  *  K  C  A  L  I  L  A  *
WTK3S4185.IF1   ATACCTCACTAGGGGCCGACGAGCTGCGGCAAGTAAAAATGTGCATTGATATTGGCCTAG
Frame 1         Y  T  S  L  G  A  D  E  L  R  Q  V  K  M  C  I  D  I  G  L
WTK3S4185.IF2   ATACCTCACTAGGGGCCGACGAGCTGCGGCAAGTAAAAATGTGCATTGATATTGGCCTAG
Frame 1         Y  T  S  L  G  A  D  E  L  R  Q  V  K  M  C  I  D  I  G  L
WTK3S4185.IF3   ATACCTCACTAGGGGCCGACGAGCTGCGGCAAGTAAAAATGTGCATTGATATTGGCCTAG
Frame 1          I  P  H  *  G  P  T  S  C  G  K  *  K  C  A  L  I  L  A  *

                3,070     3,080     3,090     3,100     3,110     3,120
WTK3HLT.IF1     ACTGTGTCAAGTCAAACCCTGAAAAGAGACCTACAGCTGGGGCCATCATGCTCTGGCTTG
Frame 1         D  C  V  K  S  N  P  E  K  R  P  T  A  G  A  I  M  L  W  L
WTK3HLT.IF2     ACTGTGTCAAGTCAAACCCTGAAAAGAGACCTACAGCTGGGGCCATCATGCTCTGGCTTG
Frame 1         D  C  V  K  S  N  P  E  K  R  P  T  A  G  A  I  M  L  W  L
WTK3HLT.IF3     ACTGTGTCAAGTCAAACCCTGAAAAGAGACCTACAGCTGGGGCCATCATGCTCTGGCTTG
Frame 1          T  V  S  S  Q  T  L  K  R  D  L  Q  L  G  P  S  C  S  G  L
WTK3S4185.IF1   ACTGTGTCAAGTCAAACCCTGAAAAGAGACCTACAGCTGGGGCCATCATGCTCTGGCTTG
Frame 1         D  C  V  K  S  N  P  E  K  R  P  T  A  G  A  I  M  L  W  L
WTK3S4185.IF2   ACTGTGTCAAGTCAAACCCTGAAAAGAGACCTACAGCTGGGGCCATCATGCTCTGGCTTG
Frame 1         D  C  V  K  S  N  P  E  K  R  P  T  A  G  A  I  M  L  W  L
WTK3S4185.IF3   ACTGTGTCAAGTCAAACCCTGAAAAGAGACCTACAGCTGGGGCCATCATGCTCTGGCTTG
Frame 1          T  V  S  S  Q  T  L  K  R  D  L  Q  L  G  P  S  C  S  G  L
```

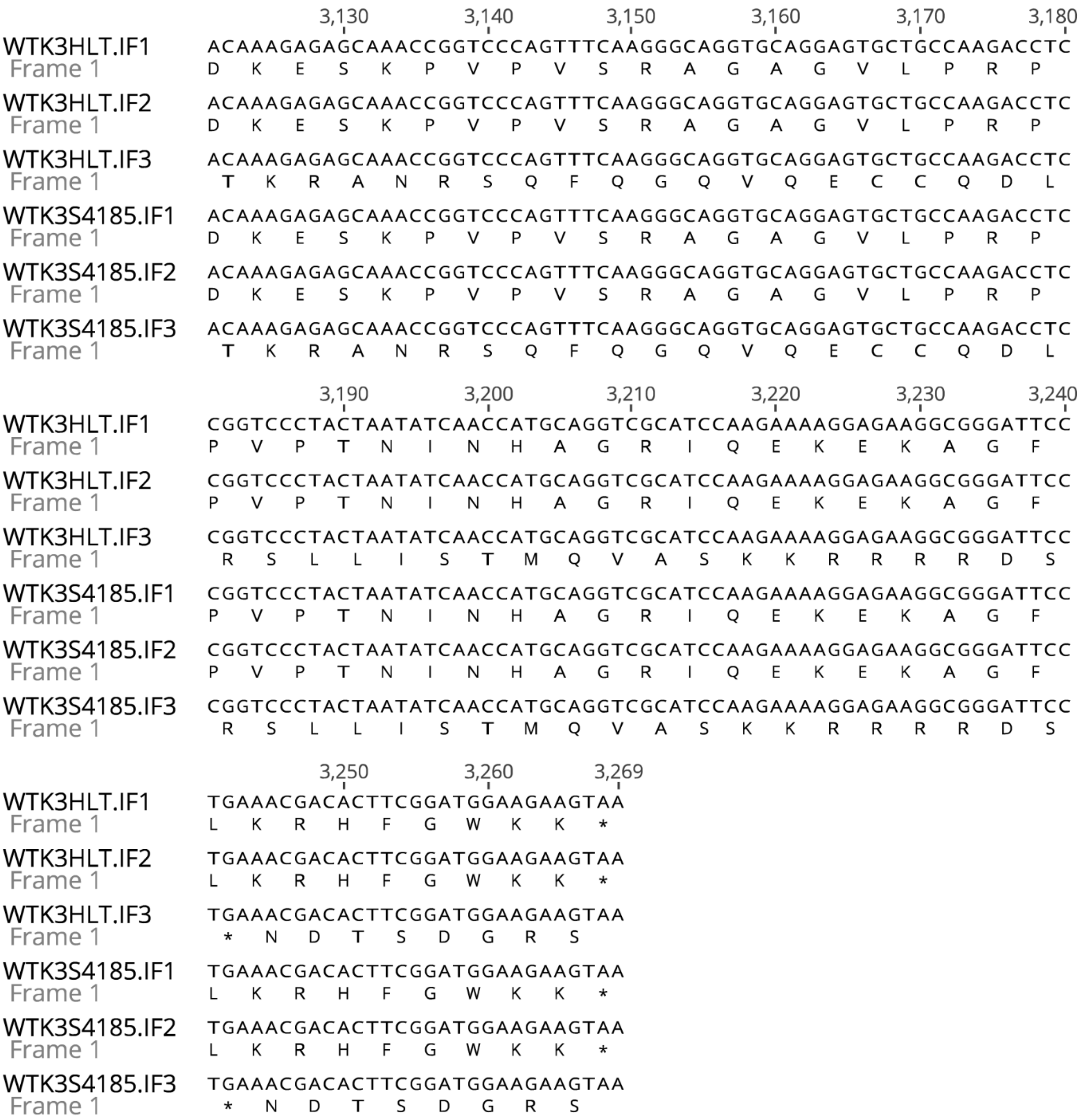

Supplementary Data 1 *WTK3* alternative transcript variants

a Cloning and sequencing of *WTK3* cDNA clones from HLT and S4185 revealed three transcript variants, designated WTK3^{HLT}.IF1.1 to WTK3^{HLT}.IF3 and WTK3^{S4185}.IF1 to WTK3^{S4185}.IF3 (colonies number of each variant was indicated in the brackets), respectively. WTK3^{HLT}.IF1 and WTK3^{S4185}.IF1 are the main variants with 11 exons encoding for complete kinase I and kinase II domains. WTK3^{HLT}.IF2 and WTK3^{S4185}.IF2 transcripts were derived from the mis-splicing of the 4th exon and 4th intron, resulting astop codon in intron 4 (marked in red). WTK3^{HLT}.IF3 isform spliced out the fifth exon and generated a premature stop codon in the 6th exon (marked in red). WTK3^{S4185}.IF3 isoform was derived from the mis-splicing of the 5th exon and 5th intron, resulting a stop codon in intron 5 (marked in red) . **b** HLT and S4185 variants sequences and predicted protein.
