# Supplementary material for: A rare gain of function mutation in a wheat tandem kinase confers resistance to powdery mildew
Source: Nat Commun. 2020 Feb 3;11:680. doi: 10.1038/s41467-020-14294-0 (PMC6997164; doi:10.1038/s41467-020-14294-0)
Supplement: Supplementary file 6 — Supplementary Data 2 [file 41467_2020_14294_MOESM6_ESM.pdf]

a

```
            1        10        20        30        40        50        60
Hulutou     TTGTTGGCCAATCGTTATCACCGGTACTACCAGGGGCGGAGACAGGGAGGGGCGAGCAGGGGTCTGACC
Baihulu     TTGTTGGCCAATCGTTATCACCGGTACTACCAGGGGCGGAGACAGGGAGGGGCGAGCAGGGGTCTGACC
Chiyacao    TTGTTGGCCAATCGTTATCACCGGTACTACCAGGGGCGGAGACAGGGAGGGGCGAGCAGGGGTCTGACC
Hongmangmai TTGTTGGCCAATCGTTATCACCGGTACTACCAGGGGCGGAGACAGGGAGGGGCGAGCAGGGGTCTGACC
            70        80        90        100       110       120       130
Hulutou     CCTGCCAATCGCTCGCCCCCCTCAACGATTTGTAGCAGGTAGTGCGTAACAGCCGCAGCTAATCACATA
Baihulu     CCTGCCAATCGCTCGCCCCCCTCAACGATTTGTAGCAGGTAGTGCGTAACAGCCGCAGCTAATCACATA
Chiyacao    CCTGCCAATCGCTCGCCCCCCTCAACGATTTGTAGCAGGTAGTGCGTAACAGCCGCAGCTAATCACATA
Hongmangmai CCTGCCAATCGCTCGCCCCCCTCAACGATTTGTAGCAGGTAGTGCGTAACAGCCGCAGCTAATCACATA
             140       150       160       170       180       190       200
Hulutou     ATTAACCCATTTCTTTTTGAGCGTAACATAAGCCCATATAGAAACAATTAACTGGACCAGCACCAAAGC
Baihulu     ATTAACCCATTTCTTTTTGAGCGTAACATAAGCCCATATAGAAACAATTAACTGGACCAGCACCAAAGC
Chiyacao    ATTAACCCATTTCTTTTTGAGCGTAACATAAGCCCATATAGAAACAATTAACTGGACCAGCACCAAAGC
Hongmangmai ATTAACCCATTTCTTTTTGAGCGTAACATAAGCCCATATAGAAACAATTAACTGGACCAGCACCAAAGC
              210       220       230       240       250       260       270
Hulutou     AGAGAAGCCCACCCAACAATCCATGTACGACGGTTGTGCCTAGCTATTTTTTAGCTCGGCCCAGTGCCT
Baihulu     AGAGAAGCCCACCCAACAATCCATGTACGACGGTTGTGCCTAGCTATTTTTTAGCTCGGCCCAGTGCCT
Chiyacao    AGAGAAGCCCACCCAACAATCCATGTACGACGGTTGTGCCTAGCTATTTTTTAGCTCGGCCCAGTGCCT
Hongmangmai AGAGAAGCCCACCCAACAATCCATGTACGACGGTTGTGCCTAGCTATTTTTTAGCTCGGCCCAGTGCCT
               280       290       300       310       320       330       340
Hulutou     AATCCGTCAGTACGTACATGCGTGTTCTGCACTTCTCCTAGGGCGTTCTTCGCCGCCGCCGCTTGCTTG
Baihulu     AATCCGTCAGTACGTACATGCGTGTTCTGCACTTCTCCTAGGGCGTTCTTCGCCGCCGCCGCTTGCTTG
Chiyacao    AATCCGTCAGTACGTACATGCGTGTTCTGCACTTCTCCTAGGGCGTTCTTCGCCGCCGCCGCTTGCTTG
Hongmangmai AATCCGTCAGTACGTACATGCGTGTTCTGCACTTCTCCTAGGGCGTTCTTCGCCGCCGCCGCTTGCTTG
                350       360       370       380       390       400       410
Hulutou     TACTGCTAGGGTCTAGGGTGTTTGCCATTGTCTCTGGCCGCTACTCGACACGACAACAACACCGTCCAC
Baihulu     TACTGCTAGGGTCTAGGGTGTTTGCCATTGTCTCTGGCCGCTACTCGACACGACAACAACACCGTCCAC
Chiyacao    TACTGCTAGGGTCTAGGGTGTTTGCCATTGTCTCTGGCCGCTACTCGACACGACAACAACACCGTCCAC
Hongmangmai TACTGCTAGGGTCTAGGGTGTTTGCCATTGTCTCTGGCCGCTACTCGACACGACAACAACACCGTCCAC
                 420       430       440       450       460       470       480
Hulutou     CACTGATTTGCAGTACGCAGTTGACGCACGGGCATGGGCGGACGGCTTGACGCTGGCGGATCTAGATCT
Baihulu     CACTGATTTGCAGTACGCAGTTGACGCACGGGCATGGGCGGACGGCTTGACGCTGGCGGATCTAGATCT
Chiyacao    CACTGATTTGCAGTACGCAGTTGACGCACGGGCATGGGCGGACGGCTTGACGCTGGCGGATCTAGATCT
Hongmangmai CACTGATTTGCAGTACGCAGTTGACGCACGGGCATGGGCGGACGGCTTGACGCTGGCGGATCTAGATCT
                  490       500       510       520       530       540       550
Hulutou     CGGGTGAGCAGGATAGATGCAGGTATATTACATTGATAGTAGTTATTACTCTCTATGTTCTAAAATAAT
Baihulu     CGGGTGAGCAGGATAGATGCAGGTATATTACATTGATAGTAGTTATTACTCTCTATGTTCTAAAATAAT
Chiyacao    CGGGTGAGCAGGATAGATGCAGGTATATTACATTGATAGTAGTTATTACTCTCTATGTTCTAAAATAAT
Hongmangmai CGGGTGAGCAGGATAGATGCAGGTATATTACATTGATAGTAGTTATTACTCTCTATGTTCTAAAATAAT
                   560       570       580       590       600       610       620
Hulutou     TGTAGCTCTACGTTTGTCCTAAGTAAAACTAATTTAAGTTTAATGATGTTTATAGAAAAATATGGTAAG
Baihulu     TGTAGCTCTACGTTTGTCCTAAGTAAAACTAATTTAAGTTTAATGATGTTTATAGAAAAATATGGTAAG
Chiyacao    TGTAGCTCTACGTTTGTCCTAAGTAAAACTAATTTAAGTTTAATGATGTTTATAGAAAAATATGGTAAG
Hongmangmai TGTAGCTCTACGTTTGTCCTAAGTAAAACTAATTTAAGTTTAATGATGTTTATAGAAAAATATGGTAAG
                    630       640       650       660       670       680       690
Hulutou     ATCTACAAAATTAAATATACATAGTGTGCATATTTTTTAAAGATTCTAATAAAGCTATGGTGTTTCAAA
Baihulu     ATCTACAAAATTAAATATACATAGTGTGCATATTTTTTAAAGATTCTAATAAAGCTATGGTGTTTCAAA
Chiyacao    ATCTACAAAATTAAATATACATAGTGTGCATATTTTTTAAAGATTCTAATAAAGCTATGGTGTTTCAAA
Hongmangmai ATCTACAAAATTAAATATACATAGTGTGCATATTTTTTAAAGATTCTAATAAAGCTATGGTGTTTCAAA
```

```
                  700         710         720         730         740         750
Hulutou      ATATTGATATATTTTTCTACAAACTGGTAAAACTTGAAGAAGTTTAACTTAGAATAAATTTAGAGCTTC
Baihulu      ATATTGATATATTTTTCTACAAACTGGTAAAACTTGAAGAAGTTTAACTTAGAATAAATTTAGAGCTTC
Chiyacao     ATATTGATATATTTTTCTACAAACTGGTAAAACTTGAAGAAGTTTAACTTAGAATAAATTTAGAGCTTC
Hongmangmai  ATATTGATATATTTTTCTACAAACTGGTAAAACTTGAAGAAGTTTAACTTAGAATAAATTTAGAGCTTC
             760       770       780       790       800       810       820
Hulutou      TATTATTTTAAAACAGAGGGAGTGAGACTTTTCTTTGGATAAAAAGACCTTACTATGCTTAATTAATGT
Baihulu      TATTATTTTAAAACAGAGGGAGTGAGACTTTTCTTTGGATAAAAAGACCTTACTATGCTTAATTAATGT
Chiyacao     TATTATTTTAAAACAGAGGGAGTGAGACTTTTCTTTGGATAAAAAGACCTTACTATGCTTAATTAATGT
Hongmangmai  TATTATTTTAAAACAGAGGGAGTGAGACTTTTCTTTGGATAAAAAGACCTTACTATGCTTAATTAATGT
             830       840       850       860       870       880       890
Hulutou      TTCATATAAAACAGTAAATTAGTCTAGTAATTATGTGTCTATTTGTACTAGTCAAAGATGAAGCGGAAG
Baihulu      TTCATATAAAACAGTAAATTAGTCTAGTAATTATGTGTCTATTTGTACTAGTCAAAGATGAAGCGGAAG
Chiyacao     TTCATATAAAACAGTAAATTAGTCTAGTAATTATGTGTCTATTTGTACTAGTCAAAGATGAAGCGGAAG
Hongmangmai  TTCATATAAAACAGTAAATTAGTCTAGTAATTATGTGTCTATTTGTACTAGTCAAAGATGAAGCGGAAG
              900       910       920       930       940       950       960
Hulutou      AATCAACAACAAATGGGGTAAATTCTTTTTCAATCCAATTGGTTCAATTTCAAGCTCCAACATTATTAC
Baihulu      AATCAACAACAAATGGGGTAAATTCTTTTTCAATCCAATTGGTTCAATTTCAAGCTCCAACATTATTAC
Chiyacao     AATCAACAACAAATGGGGTAAATTCTTTTTCAATCCAATTGGTTCAATTTCAAGCTCCAACATTATTAC
Hongmangmai  AATCAACAACAAATGGGGTAAATTCTTTTTCAATCCAATTGGTTCAATTTCAAGCTCCAACATTATTAC
               970       980       990      1,000     1,010     1,020     1,030
Hulutou      TACTAATAGTATATTTTTTATAGTTCCAAACAATAATTTTATGTATTATCATCGATGCTATCGTGAGAC
Baihulu      TACTAATAGTATATTTTTTATAGTTCCAAACAATAATTTTATGTATTATCATCGATGCTATCGTGAGAC
Chiyacao     TACTAATAGTATATTTTTTATAGTTCCAAACAATAATTTTATGTATTATCATCGATGCTATCGTGAGAC
Hongmangmai  TACTAATAGTATATTTTTTATAGTTCCAAACAATAATTTTATGTATTATCATCGATGCTATCGTGAGAC
              1,040     1,050     1,060     1,070     1,080     1,090     1,100
Hulutou      TTAATATATTGTGATATTCTGTCATTTGCGTCAACAATGGTGTCTCGCCCTCCTCATGCTGAAATCCTG
Baihulu      TTAATATATTGTGATATTCTGTCATTTGCGTCAACAATGGTGTCTCGCCCTCCTCATGCTGAAATCCTG
Chiyacao     TTAATATATTGTGATATTCTGTCATTTGCGTCAACAATGGTGTCTCGCCCTCCTCATGCTGAAATCCTG
Hongmangmai  TTAATATATTGTGATATTCTGTCATTTGCGTCAACAATGGTGTCTCGCCCTCCTCATGCTGAAATCCTG
               1,110     1,120     1,130     1,140     1,150     1,160     1,170
Hulutou      GCTCCCCCCCTGGGTACTACTACAATGAAATCATGACGCATGCAGAACCCGACGGACAACTGCTTCCCT
Baihulu      GCTCCCCCCCTGGGTACTACTACAATGAAATCATGACGCATGCAGAACCCGACGGACAACTGCTTCCCT
Chiyacao     GCTCCCCCCCTGGGTACTACTACAATGAAATCATGACGCATGCAGAACCCGACGGACAACTGCTTCCCT
Hongmangmai  GCTCCCCCCCTGGGTACTACTACAATGAAATCATGACGCATGCAGAACCCGACGGACAACTGCTTCCCT
                1,180     1,190     1,200     1,210     1,220     1,230     1,240
Hulutou      TAATCGAGAGTGGGACACTTGCAAACTCGCAACTTTACCCCTAGACCCACCAAATTACGACGCTTACAA
Baihulu      TAATCGAGAGTGGGACACTTGCAAACTCGCAACTTTACCCCTAGACCCACCAAATTACGACGCTTACAA
Chiyacao     TAATCGAGAGTGGGACACTTGCAAACTCGCAACTTTACCCCTAGACCCACCAAATTACGACGCTTACAA
Hongmangmai  TAATCGAGAGTGGGACACTTGCAAACTCGCAACTTTACCCCTAGACCCACCAAATTACGACGCTTACAA
                 1,250     1,260     1,270     1,280     1,290     1,300     1,310
Hulutou      ACACACGGGATTCAGAAACTTTTACTGAAGCAAAATGCTCTGATTTTGCATATTTTGAAGCCGATGCAC
Baihulu      ACACACGGGATTCAGAAACTTTTACTGAAGCAAAATGCTCTGATTTTGCATATTTTGAAGCCGATGCAC
Chiyacao     ACACACGGGATTCAGAAACTTTTACTGAAGCAAAATGCTCTGATTTTGCATATTTTGAAGCCGATGCAC
Hongmangmai  ACACACGGGATTCAGAAACTTTTACTGAAGCAAAATGCTCTGATTTTGCATATTTTGAAGCCGATGCAC
                  1,320     1,330     1,340     1,350     1,360     1,370     1,380
Hulutou      ATTTACCGTCCTAGCAATCCCATGCTCAGATTGCGTATACTGAAGCAAACAAGCTGTCGGGGGAGGAGG
Baihulu      ATTTACCGTCCTAGCAATCCCATGCTCAGATTGCGTATACTGAAGCAAACAAGCTGTCGGGGGAGGAGG
Chiyacao     ATTTACCGTCCTAGCAATCCCATGCTCAGATTGCGTATACTGAAGCAAACAAGCTGTCGGGGGAGGAGG
Hongmangmai  ATTTACCGTCCTAGCAATCCCATGCTCAGATTGCGTATACTGAAGCAAACAAGCTGTCGGGGGAGGAGG
```

```
              1,390    1,400    1,410    1,420    1,430    1,440
Hulutou       CAGCGAGAAGCTCCATAAAACACAAAAATAGAATCGCCCAAACGAGCAGAACACGCTGCTGGCAACAGT
Baihulu       CAGCGAGAAGCTCCATAAAACACAAAAATAGAATCGCCCAAACGAGCAGAACACGCTGCTGGCAACAGT
Chiyacao      CAGCGAGAAGCTCCATAAAACACAAAAATAGAATCGCCCAAACGAGCAGAACACGCTGCTGGCAACAGT
Hongmangmai   CAGCGAGAAGCTCCATAAAACACAAAAATAGAATCGCCCAAACGAGCAGAACACGCTGCTGGCAACAGT

              1,450    1,460    1,470    1,480    1,490    1,500    1,510
Hulutou       TTGGTTGGATAATGGTGAGGTGATCGGCAGCAGGAACTTGCAGTGGCCATGGAAAAAACTAAGTAAGAG
Baihulu       TTGGTTGGATAATGGTGAGGTGATCGGCAGCAGGAACTTGCAGTGGCCATGGAAAAAACTAAGTAAGAG
Chiyacao      TTGGTTGGATAATGGTGAGGTGATCGGCAGCAGGAACTTGCAGTGGCCATGGAAAAAACTAAGTAAGAG
Hongmangmai   TTGGTTGGATAATGGTGAGGTGATCGGCAGCAGGAACTTGCAGTGGCCATGGAAAAAACTAAGTAAGAG

              1,520    1,530    1,540    1,550    1,560    1,570    1,580
Hulutou       AGAAAAAGAAGAAGAAAAGTCAGTTAGTTCATACGGTGTTACAGCCTGGTAAGGATAAGGTCAGCAGGA
Baihulu       AGAAAAAGAAGAAGAAAAGTCAGTTAGTTCATACGGTGTTACAGCCTGGTAAGGATAAGGTCAGCAGGA
Chiyacao      AGAAAAAGAAGAAGAAAAGTCAGTTAGTTCATACGGTGTTACAGCCTGGTAAGGATAAGGTCAGCAGGA
Hongmangmai   AGAAAAAGAAGAAGAAAAGTCAGTTAGTTCATACGGTGTTACAGCCTGGTAAGGATAAGGTCAGCAGGA

              1,590    1,600    1,610    1,620    1,630    1,640    1,650
Hulutou       TGATAAGGCACATGTGGTGGTGGAGAAGTGGGCACTTGTTGCTTGGCTACGTACACACCTAGACGTACA
Baihulu       TGATAAGGCACATGTGGTGGTGGAGAAGTGGGCACTTGTTGCTTGGCTACGTACACACCTAGACGTACA
Chiyacao      TGATAAGGCACATGTGGTGGTGGAGAAGTGGGCACTTGTTGCTTGGCTACGTACACACCTAGACGTACA
Hongmangmai   TGATAAGGCACATGTGGTGGTGGAGAAGTGGGCACTTGTTGCTTGGCTACGTACACACCTAGACGTACA

              1,660    1,670    1,680    1,690    1,700    1,710    1,720
Hulutou       TGAGAAAGAACATCTCAAAAATAATCAAACAAACATGTGATGTATTAAATCCTGTTTCCTCAAGTGGGA
Baihulu       TGAGAAAGAACATCTCAAAAATAATCAAACAAACATGTGATGTATTAAATCCTGTTTCCTCAAGTGGGA
Chiyacao      TGAGAAAGAACATCTCAAAAATAATCAAACAAACATGTGATGTATTAAATCCTGTTTCCTCAAGTGGGA
Hongmangmai   TGAGAAAGAACATCTCAAAAATAATCAAACAAACATGTGATGTATTAAATCCTGTTTCCTCAAGTGGGA

              1,730    1,740    1,750    1,760    1,770    1,780    1,790
Hulutou       CAGAGGATTTGACCACATTGTTTGGATTTCTTGTGATAATTCAAGTCTTTTTTCTATTAGCCATGTTCA
Baihulu       CAGAGGATTTGACCACATTGTTTGGATTTCTTGTGATAATTCAAGTCTTTTTTCTATTAGCCATGTTCA
Chiyacao      CAGAGGATTTGACCACATTGTTTGGATTTCTTGTGATAATTCAAGTCTTTTTTCTATTAGCCATGTTCA
Hongmangmai   CAGAGGATTTGACCACATTGTTTGGATTTCTTGTGATAATTCAAGTCTTTTTTCTATTAGCCATGTTCA

              1,800    1,810    1,820    1,830    1,840    1,850    1,860
Hulutou       AGAATTCGCACGATAATTAACCAGTTAACTTGCGATTAATCCTTATCCGTAGGGTCACCAAGTAGTCAA
Baihulu       AGAATTCGCACGATAATTAACCAGTTAACTTGCGATTAATCCTTATCCGTAGGGTCACCAAGTAGTCAA
Chiyacao      AGAATTCGCACGATAATTAACCAGTTAACTTGCGATTAATCCTTATCCGTAGGGTCACCAAGTAGTCAA
Hongmangmai   AGAATTCGCACGATAATTAACCAGTTAACTTGCGATTAATCCTTATCCGTAGGGTCACCAAGTAGTCAA

              1,870    1,880    1,890    1,900    1,910    1,920    1,930
Hulutou       TAAACTGATAAATCATTTAATTAATTAATTAATTAAATGGCCATTAACTTACTGATTAGCCTATTAATT
Baihulu       TAAACTGATAAATCATTTAATTAATTAATTAATTAAATGGCCATTAACTTACTGATTAGCCTATTAATT
Chiyacao      TAAACTGATAAATCATTTAATTAATTAATTAATTAAATGGCCATTAACTTACTGATTAGCCTATTAATT
Hongmangmai   TAAACTGATAAATCATTTAATTAATTAATTAATTAAATGGCCATTAACTTACTGATTAGCCTATTAATT

              1,940    1,950    1,960    1,970    1,980    1,990    2,000
Hulutou       CCCTACTCACCAGCCAACCGAGCAGCCACTGGCGTACCAACGGGTGTGCCAGGTGTTGAAAAATATTTT
Baihulu       CCCTACTCACCAGCCAACCGAGCAGCCACTGGCGTACCAACGGGTGTGCCAGGTGTTGAAAAATATTTT
Chiyacao      CCCTACTCACCAGCCAACCGAGCAGCCACTGGCGTACCAACGGGTGTGCCAGGTGTTGAAAAATATTTT
Hongmangmai   CCCTACTCACCAGCCAACCGAGCAGCCACTGGCGTACCAACGGGTGTGCCAGGTGTTGAAAAATATTTT

              2,010    2,020    2,030    2,040    2,050    2,060    2,070
Hulutou       TGCACCATGTAAAAATTAGCTGATTTTTAAGGCCCATGCAAGGTAATTGAAATAGACTTCATTTCTTTT
Baihulu       TGCACCATGTAAAAATTAGCTGATTTTTAAGGCCCATGCAAGGTAATTGAAATAGACTTCATTTCTTTT
Chiyacao      TGCACCATGTAAAAATTAGCTGATTTTTAAGGCCCATGCAAGGTAATTGAAATAGACTTCATTTCTTTT
Hongmangmai   TGCACCATGTAAAAATTAGCTGATTTTTAAGGCCCATGCAAGGTAATTGAAATAGACTTCATTTCTTTT
```

2,080 2,090 2,100 2,110 2,120 2,130

Hulutou AAATGTGCACACCGTTCATTTTATTTCTAGATTCGCCATTGCGAGCAGCTACCAGTTAACGATTTCCTT
Baihulu AAATGTGCACACCGTTCATTTTATTTCTAGATTCGCCATTGCGAGCAGCTACCAGTTAACGATTTCCTT
Chiyacao AAATGTGCACACCGTTCATTTTATTTCTAGATTCGCCATTGCGAGCAGCTACCAGTTAACGATTTCCTT
Hongmangmai AAATGTGCACACCGTTCATTTTATTTCTAGATTCGCCATTGCGAGCAGCTACCAGTTAACGATTTCCTT

2,140 2,150 2,160 2,170 2,180 2,190 2,200

Hulutou AACAATGATGATTAGTATATGTGTTACTAAAGAGCCACAAAGTTGAGATGACTGTGCTGTGTGTGAAAA
Baihulu AACAATGATGATTAGTATATGTGTTACTAAAGAGCCACAAAGTTGAGATGACTGTGCTGTGTGTGAAAA
Chiyacao AACAATGATGATTAGTATATGTGTTACTAAAGAGCCACAAAGTTGAGATGACTGTGCTGTGTGTGAAAA
Hongmangmai AACAATGATGATTAGTATATGTGTTACTAAAGAGCCACAAAGTTGAGATGACTGTGCTGTGTGTGAAAA

2,210 2,220 2,230 2,240 2,250 2,260 2,270

Hulutou AAAAATATGAATCGGGTAAATTCGTGGCATGAAAAAGCCGCAATATTAAAATGCAAGATTGATCCTAAA
Baihulu AAAAATATGAATCGGGTAAATTCGTGGCATGAAAAAGCCGCAATATTAAAATGCAAGATTGATCCTAAA
Chiyacao AAAAATATGAATCGGGTAAATTCGTGGCATGAAAAAGCCGCAATATTAAAATGCAAGATTGATCCTAAA
Hongmangmai AAAAATATGAATCGGGTAAATTCGTGGCATGAAAAAGCCGCAATATTAAAATGCAAGATTGATCCTAAA

2,280 2,290 2,300 2,310 2,320 2,330 2,340

Hulutou ACCAATTAAGTGAATGGTTGTCTCAATACAACCTTAAATATATTAAAACAAGTTTCATTATCAAAATAT
Baihulu ACCAATTAAGTGAATGGTTGTCTCAATACAACCTTAAATATATTAAAACAAGTTTCATTATCAAAATAT
Chiyacao ACCAATTAAGTGAATGGTTGTCTCAATACAACCTTAAATATATTAAAACAAGTTTCATTATCAAAATAT
Hongmangmai ACCAATTAAGTGAATGGTTGTCTCAATACAACCTTAAATATATTAAAACAAGTTTCATTATCAAAATAT

2,350 2,360 2,370 2,380 2,390 2,400 2,410

Hulutou ATATTAAAAAGTTCCCTTTCTTCCTCAAATTTGAGCAGGCACCTGTGCTTCTCCAAAGATTATTTCGCC
Baihulu ATATTAAAAAGTTCCCTTTCTTCCTCAAATTTGAGCAGGCACCTGTGCTTCTCCAAAGATTATTTCGCC
Chiyacao ATATTAAAAAGTTCCCTTTCTTCCTCAAATTTGAGCAGGCACCTGTGCTTCTCCAAAGATTATTTCGCC
Hongmangmai ATATTAAAAAGTTCCCTTTCTTCCTCAAATTTGAGCAGGCACCTGTGCTTCTCCAAAGATTATTTCGCC

2,420 2,430 2,440 2,450 2,460 2,470 2,480

Hulutou TCTTCTCGCCCATGGCGTCTCCTCTTCCCACAGACGTTGTCGCAGCCCCATTCACCCCCTCAGTCATGC
Baihulu TCTTCTCGCCCATGGCGTCTCCTCTTCCCACAGACGTTGTCGCAGCCCCATTCACCCCCTCAGTCATGC
Chiyacao TCTTCTCGCCCATGGCGTCTCCTCTTCCCACAGACGTTGTCGCAGCCCCATTCACCCCCTCAGTCATGC
Hongmangmai TCTTCTCGCCCATGGCGTCTCCTCTTCCCACAGACGTTGTCGCAGCCCCATTCACCCCCTCAGTCATGC

2,490 2,500 2,510 2,520 2,530 2,540 2,550

Hulutou ACATGCGCCCCGTTGATGTCTGGCCGCCGAGTACATAAATTCATAAATAAATTCAACGAGACGGATCCG
Baihulu ACATGCGCCCCGTTGATGTCTGGCCGCCGAGTACATAAATTCATAAATAAATTCAACGAGACGGATCCG
Chiyacao ACATGCGCCCCGTTGATGTCTGGCCGCCGAGTACATAAATTCATAAATAAATTCAACGAGACGGATCCG
Hongmangmai ACATGCGCCCCGTTGATGTCTGGCCGCCGAGTACATAAATTCATAAATAAATTCAACGAGACGGATCCG

2,560 2,570 2,580 2,590 2,600 2,610 2,620

Hulutou ATGATCTCCTCAAGAGCGTGCTTTTAAAATGCAGGCTGCCGCGAACCAATCTGTGATTGGATAGTTAGA
Baihulu ATGATCTCCTCAAGAGCGTGCTTTTAAAATGCAGGCTGCCGCGAACCAATCTGTGATTGGATAGTTAGA
Chiyacao ATGATCTCCTCAAGAGCGTGCTTTTAAAATGCAGGCTGCCGCGAACCAATCTGTGATTGGATAGTTAGA
Hongmangmai ATGATCTCCTCAAGAGCGTGCTTTTAAAATGCAGGCTGCCGCGAACCAATCTGTGATTGGATAGTTAGA

2,630 2,640 2,650 2,660 2,670 2,680 2,690

Hulutou AGGTCTGCGGTAACCCCATCCAACCAGATTCATCCAGTCCCAAACTTAATATTGATGCTCACATTTTTC
Baihulu AGGTCTGCGGTAACCCCATCCAACCAGATTCATCCAGTCCCAAACTTAATATTGATGCTCACATTTTTC
Chiyacao AGGTCTGCGGTAACCCCATCCAACCAGATTCATCCAGTCCCAAACTTAATATTGATGCTCACATTTTTC
Hongmangmai AGGTCTGCGGTAACCCCATCCAACCAGATTCATCCAGTCCCAAACTTAATATTGATGCTCACATTTTTC

2,700 2,710 2,720 2,730 2,740 2,750 2,760

Hulutou TAGATTTATTTCAGGCTTTCGTCAATCTCAACATGCTATGTCGGCTCAGACTCTCGAAGGTATTTATAG
Baihulu TAGATTTATTTCAGGCTTTCGTCAATCTCAACATGCTATGTCGGCTCAGACTCTCGAAGGTATTTATAG
Chiyacao TAGATTTATTTCAGGCTTTCGTCAATCTCAACATGCTATGTCGGCTCAGACTCTCGAAGGTATTTATAG
Hongmangmai TAGATTTATTTCAGGCTTTCGTCAATCTCAACATGCTATGTCGGCTCAGACTCTCGAAGGTATTTATAG

```
                 2,770     2,780     2,790     2,800     2,810     2,820
Hulutou      GGTAGGGTATACGTGTATGTGTTCACAGAGGTGTGAGTGCTCGTTTATGTGAGCGACTTCGACTGTGTC
Baihulu      GGTAGGGTATACGTGTATGTGTTCACAGAGGTGTGAGTGCTCGTTTATGTGAGCGACTTCGACTGTGTC
Chiyacao     GGTAGGGTATACGTGTATGTGTTCACAGAGGTGTGAGTGCTCGTTTATGTGAGCGACTTCGACTGTGTC
Hongmangmai  GGTAGGGTATACGTGTATGTGTTCACAGAGGTGTGAGTGCTCGTTTATGTGAGCGACTTCGACTGTGTC
             2,830     2,840     2,850     2,860     2,870     2,880     2,890
Hulutou      GTGTTAAAAAAAATGCAAGCTGCCTCTGTCCCCGCCGTTTTTTTAATTATTAATCTGGCATGTGTATGG
Baihulu      GTGTTAAAAAAAATGCAAGCTGCCTCTGTCCCCGCCGTTTTTTTAATTATTAATCTGGCATGTGTATGG
Chiyacao     GTGTTAAAAAAAATGCAAGCTGCCTCTGTCCCCGCCGTTTTTTTAATTATTAATCTGGCATGTGTATGG
Hongmangmai  GTGTTAAAAAAAATGCAAGCTGCCTCTGTCCCCGCCGTTTTTTTAATTATTAATCTGGCATGTGTATGG
              2,900     2,910     2,920     2,930     2,940     2,950     2,960
Hulutou      TAATTCAACAAGTTGATCGGGTCTTAGGAAATGCAAGGTCCCTCGGTCACCAGGCCGACCGGTCTTTTT
Baihulu      TAATTCAACAAGTTGATCGGGTCTTAGGAAATGCAAGGTCCCTCGGTCACCAGGCCGACCGGTCTTTTT
Chiyacao     TAATTCAACAAGTTGATCGGGTCTTAGGAAATGCAAGGTCCCTCGGTCACCAGGCCGACCGGTCTTTTT
Hongmangmai  TAATTCAACAAGTTGATCGGGTCTTAGGAAATGCAAGGTCCCTCGGTCACCAGGCCGACCGGTCTTTTT
               2,970     2,980     2,990     3,000     3,010     3,020     3,030
Hulutou      TTTTTCAGACAAAGGGCCGACCGATCATTTATTAGTACTCTGCTTATCGGGCCATTGGCACAGCTCTAG
Baihulu      TTTTTCAGACAAAGGGCCGACCGATCATTTATTAGTACTCTGCTTATCGGGCCATTGGCACAGCTCTAG
Chiyacao     TTTTTCAGACAAAGGGCCGACCGATCATTTATTAGTACTCTGCTTATCGGGCCATTGGCACAGCTCTAG
Hongmangmai  TTTTTCAGACAAAGGGCCGACCGATCATTTATTAGTACTCTGCTTATCGGGCCATTGGCACAGCTCTAG
                3,040     3,050     3,060     3,070     3,080     3,090     3,100
Hulutou      AGCTCCGGATCTGGATCGACTTGTTATGCTCCCCCCGGTCAACGCCTTTGCCTGCTGAGCCTGGGAATG
Baihulu      AGCTCCGGATCTGGATCGACTTGTTATGCTCCCCCCGGTCAACGCCTTTGCCTGCTGAGCCTGGGAATG
Chiyacao     AGCTCCGGATCTGGATCGACTTGTTATGCTCCCCCCGGTCAACGCCTTTGCCTGCTGAGCCTGGGAATG
Hongmangmai  AGCTCCGGATCTGGATCGACTTGTTATGCTCCCCCCGGTCAACGCCTTTGCCTGCTGAGCCTGGGAATG
                 3,110     3,120     3,130     3,140     3,150     3,160     3,170
Hulutou      ATTGATCCGCTCATGATCTACAAGACGAGAAGCAAGCCCAGTTTTGCTCCTAGCTAGCTGCTTTCTCCT
Baihulu      ATTGATCCGCTCATGATCTACAAGACGAGAAGCAAGCCCAGTTTTGCTCCTAGCTAGCTGCTTTCTCCT
Chiyacao     ATTGATCCGCTCATGATCTACAAGACGAGAAGCAAGCCCAGTTTTGCTCCTAGCTAGCTGCTTTCTCCT
Hongmangmai  ATTGATCCGCTCATGATCTACAAGACGAGAAGCAAGCCCAGTTTTGCTCCTAGCTAGCTGCTTTCTCCT
                  3,180     3,190     3,200     3,210     3,220     3,230     3,240
Hulutou      CCTCCTCCAATCTCCAGGTGAGTAATTAAGATGCAACTAGGTAGCTGCTTTCTGGTAAGACTTTAGCTA
Baihulu      CCTCCTCCAATCTCCAGGTGAGTAATTAAGATGCAACTAGGTAGCTGCTTTCTGGTAAGACTTTAGCTA
Chiyacao     CCTCCTCCAATCTCCAGGTGAGTAATTAAGATGCAACTAGGTAGCTGCTTTCTGGTAAGACTTTAGCTA
Hongmangmai  CCTCCTCCAATCTCCAGGTGAGTAATTAAGATGCAACTAGGTAGCTGCTTTCTGGTAAGACTTTAGCTA
                   3,250     3,260     3,270     3,280     3,290     3,300     3,310
Hulutou      CCTACTTATCTTCTGCTTGATTTGCTTTGTGCAGCAACGAATGAAGGAGAAGCTGGCAACCATATTCAT
Baihulu      CCTACTTATCTTCTGCTTGATTTGCTTTGTGCAGCAACGAATGAAGGAGAAGCTGGCAACCATATTCAT
Chiyacao     CCTACTTATCTTCTGCTTGATTTGCTTTGTGCAGCAACGAATGAAGGAGAAGCTGGCAACCATATTCAT
Hongmangmai  CCTACTTATCTTCTGCTTGATTTGCTTTGTGCAGCAACGAATGAAGGAGAAGCTGGCAACCATATTCAT
                    3,320     3,330     3,340     3,350     3,360     3,370     3,380
Hulutou      ATCCATAATCCATATCTATATGCGAGCTATTGCTTGCTAGAAAGCTTCTTGCTCGCATACTCGGAAATG
Baihulu      ATCCATAATCCATATCTATATGCGAGCTATTGCTTGCTAGAAAGCTTCTTGCTCGCATACTCGGAAATG
Chiyacao     ATCCATAATCCATATCTATATGCGAGCTATTGCTTGCTAGAAAGCTTCTTGCTCGCATACTCGGAAATG
Hongmangmai  ATCCATAATCCATATCTATATGCGAGCTATTGCTTGCTAGAAAGCTTCTTGCTCGCATACTCGGAAATG
                     3,390     3,400     3,410     3,420     3,430     3,440     3,450
Hulutou      GGCGGATACGAGTTCCAGAGGGCGGAGCTAGATGCACTGGAAGGCGTCGTACGCGATCCAACTGCGGAG
Baihulu      GGCGGATACGAGTTCCAGAGGGCGGAGCTAGATGCACTGGAAGGCGTCGTACGCGATCCAACTGCGGAG
Chiyacao     GGCGGATACGAGTTCCAGAGGGCGGAGCTAGATGCACTGGAAGGCGTCGTACGCGATCCAACTGCGGAG
Hongmangmai  GGCGGATACGAGTTCCAGAGGGCGGAGCTAGATGCACTGGAAGGCGTCGTACGCGATCCAACTGCGGAG
```

```
                3,460      3,470      3,480      3,490      3,500      3,510
Hulutou      CCAATGAGTCTGACGTTGCCGCTTCTCAGGCACATAACAAATGATTTCTCCCCTGAATTTGAAATTAGT
Baihulu      CCAATGAGTCTGACGTTGCCGCTTCTCAGGCACATAACAAATGATTTCTCCCCTGAATTTGAAATTAGT
Chiyacao     CCAATGAGTCTGACGTTGCCGCTTCTCAGGCACATAACAAATGATTTCTCCCCTGAATTTGAAATTAGT
Hongmangmai  CCAATGAGTCTGACGTTGCCGCTTCTCAGGCACATAACAAATGATTTCTCCCCTGAATTTGAAATTAGT
            3,520      3,530      3,540      3,550      3,560      3,570      3,580
Hulutou      AAAGATGATTCTGCAGTGGTTTACCTGGTACGACTAAACTCATATCTCGTTTATTATTAATATAAGCAA
Baihulu      AAAGATGATTCTGCAGTGGTTTACCTGGTACGACTAAACTCATATCTCGTTTATTATTAATATAAGCAA
Chiyacao     AAAGATGATTCTGCAGTGGTTTACCTGGTACGACTAAACTCATATCTCGTTTATTATTAATATAAGCAA
Hongmangmai  AAAGATGATTCTGCAGTGGTTTACCTGGTACGACTAAACTCATATCTCGTTTATTATTAATATAAGCAA
             3,590      3,600      3,610      3,620      3,630      3,640      3,650
Hulutou      ATCCTATCTCGTTAATTAGTACTCTGCTTATGTGGTCGAATATATATGTAGGGGGTGCTTCCAAGTGGG
Baihulu      ATCCTATCTCGTTAATTAGTACTCTGCTTATGTGGTCGAATATATATGTAGGGGGTGCTTCCAAGTGGG
Chiyacao     ATCCTATCTCGTTAATTAGTACTCTGCTTATGTGGTCGAATATATATGTAGGGGGTGCTTCCAAGTGGG
Hongmangmai  ATCCTATCTCGTTAATTAGTACTCTGCTTATGTGGTCGAATATATATGTAGGGGGTGCTTCCAAGTGGG
              3,660      3,670      3,680      3,690      3,700      3,710      3,720
Hulutou      TTCCGTGTTGCTGTCAAGAAGTCTCACTTTCGTTTTTGCTTGGATGATGAAGATGCATTCACAAATGAA
Baihulu      TTCCGTGTTGCTGTCAAGAAGTCTCACTTTCGTTTTTGCTTGGATGATGAAGATGCATTCACAAATGAA
Chiyacao     TTCCGTGTTGCTGTCAAGAAGTCTCACTTTCGTTTTTGCTTGGATGATGAAGATGCATTCACAAATGAA
Hongmangmai  TTCCGTGTTGCTGTCAAGAAGTCTCACTTTCGTTTTTGCTTGGATGATGAAGATGCATTCACAAATGAA
               3,730      3,740      3,750      3,760      3,770      3,780      3,790
Hulutou      GTTTCTATTGCAATGAAGGCTGCTCATAAGAACACAGTGCGAGTCATAGGCTACTGTCATCACACGCAT
Baihulu      GTTTCTATTGCAATGAAGGCTGCTCATAAGAACACAGTGCGAGTCATAGGCTACTGTCATCACACGCAT
Chiyacao     GTTTCTATTGCAATGAAGGCTGCTCATAAGAACACAGTGCGAGTCATAGGCTACTGTCATCACACGCAT
Hongmangmai  GTTTCTATTGCAATGAAGGCTGCTCATAAGAACACAGTGCGAGTCATAGGCTACTGTCATCACACGCAT
                3,800      3,810      3,820      3,830      3,840      3,850      3,860
Hulutou      GAGCAAATTGCCGAATACGAAGGAAAACAAGTTTTCGCAGAGGTCAGAGAAAGGTTGATCTGTACCGAG
Baihulu      GAGCAAATTGCCGAATACGAAGGAAAACAAGTTTTCGCAGAGGTCAGAGAAAGGTTGATCTGTACCGAG
Chiyacao     GAGCAAATTGCCGAATACGAAGGAAAACAAGTTTTCGCAGAGGTCAGAGAAAGGTTGATCTGTACCGAG
Hongmangmai  GAGCAAATTGCCGAATACGAAGGAAAACAAGTTTTCGCAGAGGTCAGAGAAAGGTTGATCTGTACCGAG
                 3,870      3,880      3,890      3,900      3,910      3,920      3,930
Hulutou      TATGTGCCTAACGGACCCCTTAGTGGACATATCGAAGGTAAGATATGTGCGCAAATGGATGGATACGAG
Baihulu      TATGTGCCTAACGGACCCCTTAGTGGACATATCGAAGGTAAGATATGTGCGCAAATGGATGGATACGAG
Chiyacao     TATGTGCCTAACGGACCCCTTAGTGGACATATCGAAGGTAAGATATGTGCGCAAATGGATGGATACGAG
Hongmangmai  TATGTGCCTAACGGACCCCTTAGTGGACATATCGAAGGTAAGATATGTGCGCAAATGGATGGATACGAG
                  3,940      3,950      3,960      3,970      3,980      3,990      4,000
Hulutou      TTCCAGAGGGCAGAACTAGATGCACTAGAACGCGTCGTACGCGATACAAGTGCGGAGCCAATGAGTCTG
Baihulu      TTCCAGAGGGCAGAACTAGATGCACTAGAACGCGTCGTACGCGATACAAGTGCGGAGCCAATGAGTCTG
Chiyacao     TTCCAGAGGGCAGAACTAGATGCACTAGAACGCGTCGTACGCGATACAAGTGCGGAGCCAATGAGTCTG
Hongmangmai  TTCCAGAGGGCAGAACTAGATGCACTAGAACGCGTCGTACGCGATACAAGTGCGGAGCCAATGAGTCTG
                   4,010      4,020      4,030      4,040      4,050      4,060      4,070
Hulutou      ACGTTGCCGCTTCTCAGGCACATAACAAATGATTTCTCCGATGAATCTCGAATTGGCCGAGGTGGATTC
Baihulu      ACGTTGCCGCTTCTCAGGCACATAACAAATGATTTCTCCGATGAATCTCGAATTGGCCGAGGTGGATTC
Chiyacao     ACGTTGCCGCTTCTCAGGCACATAACAAATGATTTCTCCGATGAATCTCGAATTGGCCGAGGTGGATTC
Hongmangmai  ACGTTGCCGCTTCTCAGGCACATAACAAATGATTTCTCCGATGAATCTCGAATTGGCCGAGGTGGATTC
                    4,080      4,090      4,100      4,110      4,120      4,130      4,140
Hulutou      GCAGTGGTTTACCTGGTATGATTAAACTCATACAGTATCTCCTTAGATATTAAGCAATTCCTATCTTGT
Baihulu      GCAGTGGTTTACCTGGTATGATTAAACTCATACAGTATCTCCTTAGATATTAAGCAATTCCTATCTTGT
Chiyacao     GCAGTGGTTTACCTGGTATGATTAAACTCATACAGTATCTCCTTAGATATTAAGCAATTCCTATCTTGT
Hongmangmai  GCAGTGGTTTACCTGGTATGATTAAACTCATACAGTATCTCCTTAGATATTAAGCAATTCCTATCTTGT
```

```
             4,150   4,160   4,170   4,180   4,190   4,200
Hulutou      CAATTACTAACTTCTTATTGTGGTCAAATATACAGGGGGTGCTTCCAAGTGGGTTACGTATTGCTGTTA
Baihulu      CAATTACTAACTTCTTATTGTGGTCAAATATACAGGGGGTGCTTCCAAGTGGGTTACGTATTGCTGTTA
Chiyacao     CAATTACTAACTTCTTATTGTGGTCAAATATACAGGGGGTGCTTCCAAGTGGGTTACGTATTGCTGTTA
Hongmangmai  CAATTACTAACTTCTTATTGTGGTCAAATATACAGGGGGTGCTTCCAAGTGGGTTACGTATTGCTGTTA

             4,210   4,220   4,230   4,240   4,250   4,260   4,270
Hulutou      AGAGGCTTAGCAATATTGCTTATATGAACGAAAGTGCATTTCAAAATGAAGTGTTTATCACAATGAAGG
Baihulu      AGAGGCTTAGCAATATTGCTTATATGAACGAAAGTGCATTTCAAAATGAAGTGTTTATCACAATGAAGG
Chiyacao     AGAGGCTTAGCAATATTGCTTATATGAACGAAAGTGCATTTCAAAATGAAGTGTTTATCACAATGAAGG
Hongmangmai  AGAGGCTTAGCAATATTGCTTATATGAACGAAAGTGCATTTCAAAATGAAGTGTTTATCACAATGAAGG

             4,280   4,290   4,300   4,310   4,320   4,330   4,340
Hulutou      CCACTCACAAGAACACAGTGCGATTCATGGGCTACTGTAGTCAAATACAAGGTAAACTCATCGAACACG
Baihulu      CCACTCACAAGAACACAGTGCGATTCATGGGCTACTGTAGTCAAATACAAGGTAAACTCATCGAACACG
Chiyacao     CCACTCACAAGAACACAGTGCGATTCATGGGCTACTGTAGTCAAATACAAGGTAAACTCATCGAACACG
Hongmangmai  CCACTCACAAGAACACAGTGCGATTCATGGGCTACTGTAGTCAAATACAAGGTAAACTCATCGAACACG

             4,350   4,360   4,370   4,380   4,390   4,400   4,410
Hulutou      ACGGGCAACATGTTTTCGCACAGCTCGAGGAAAGGTTGATCTGTGTGGAATATGCGCCTAAAGGAACCC
Baihulu      ACGGGCAACATGTTTTCGCACAGCTCGAGGAAAGGTTGATCTGTGTGGAATATGCGCCTAAAGGAACCC
Chiyacao     ACGGGCAACATGTTTTCGCACAGCTCGAGGAAAGGTTGATCTGTGTGGAATATGCGCCTAAAGGAACCC
Hongmangmai  ACGGGCAACATGTTTTCGCACAGCTCGAGGAAAGGTTGATCTGTGTGGAATATGCGCCTAAAGGAACCC

             4,420   4,430   4,440   4,450   4,460   4,470   4,480
Hulutou      TTGATGCACATATCGGTAAGATCGATAACACGGCATGCCCGCCCTAGCAATAATTCCCTCTCTTTATTT
Baihulu      TTGATGCACATATCGGTAAGATCGATAACACGGCATGCCCGCCCTAGCAATAATTCCCTCTCTTTATTT
Chiyacao     TTGATGCACATATCGGTAAGATCGATAACACGGCATGCCCGCCCTAGCAATAATTCCCTCTCTTTATTT
Hongmangmai  TTGATGCACATATCGGTAAGATCGATAACACGGCATGCCCGCCCTAGCAATAATTCCCTCTCTTTATTT

             4,490   4,500   4,510   4,520   4,530   4,540   4,550
Hulutou      TGGTGTTTCTGTTTGTGTGGCTGTTTTTTACTTTGTGCTCACAGAACAAAGAATGAATACCAGTGGAAC
Baihulu      TGGTGTTTCTGTTTGTGTGGCTGTTTTTTACTTTGTGCTCACAGAACAAAGAATGAATACCAGTGGAAC
Chiyacao     TGGTGTTTCTGTTTGTGTGGCTGTTTTTTACTTTGTGCTCACAGAACAAAGAATGAATACCAGTGGAAC
Hongmangmai  TGGTGTTTCTGTTTGTGTGGCTGTTTTTTACTTTGTGCTCACAGAACAAAGAATGAATACCAGTGGAAC

             4,560   4,570   4,580   4,590   4,600   4,610   4,620
Hulutou      AAAAAAGTAGACTATAATTTTAGCGCAACTGAAACCAACATGACCAGTCGTAGAACACAAATTTAGACA
Baihulu      AAAAAAGTAGACTATAATTTTAGCGCAACTGAAACCAACATGACCAGTCGTAGAACACAAATTTAGACA
Chiyacao     AAAAAAGTAGACTATAATTTTAGCGCAACTGAAACCAACATGACCAGTCGTAGAACACAAATTTAGACA
Hongmangmai  AAAAAAGTAGACTATAATTTTAGCGCAACTGAAACCAACATGACCAGTCGTAGAACACAAATTTAGACA

             4,630   4,640   4,650   4,660   4,670   4,680   4,690
Hulutou      AAGTGGACCAAATCATAAGAAATATTAAAACATTAATTTTTTAGAACATAAGGCACAAAATTATAGTGC
Baihulu      AAGTGGACCAAATCATAAGAAATATTAAAACATTAATTTTTTAGAACATAAGGCACAAAATTATAGTGC
Chiyacao     AAGTGGACCAAATCATAAGAAATATTAAAACATTAATTTTTTAGAACATAAGGCACAAAATTATAGTGC
Hongmangmai  AAGTGGACCAAATCATAAGAAATATTAAAACATTAATTTTTTAGAACATAAGGCACAAAATTATAGTGC

             4,700   4,710   4,720   4,730   4,740   4,750   4,760
Hulutou      TCGTGAATCAAAACTTGGCTACAAGTGGAACACCTATTGTAATAAATGCAAGAAAACTATTAGAGGCTT
Baihulu      TCGTGAATCAAAACTTGGCTACAAGTGGAACACCTATTGTAATAAATGCAAGAAAACTATTAGAGGCTT
Chiyacao     TCGTGAATCAAAACTTGGCTACAAGTGGAACACCTATTGTAATAAATGCAAGAAAACTATTAGAGGCTT
Hongmangmai  TCGTGAATCAAAACTTGGCTACAAGTGGAACACCTATTGTAATAAATGCAAGAAAACTATTAGAGGCTT

             4,770   4,780   4,790   4,800   4,810   4,820   4,830
Hulutou      GTGCAATAAAATTTCGGTGCCCATGGAACAAATCAAAATTCTAGTGGAACAAAAGTTTTAACTAAGTGA
Baihulu      GTGCAATAAAATTTCGGTGCCCATGGAACAAATCAAAATTCTAGTGGAACAAAAGTTTTAACTAAGTGA
Chiyacao     GTGCAATAAAATTTCGGTGCCCATGGAACAAATCAAAATTCTAGTGGAACAAAAGTTTTAACTAAGTGA
Hongmangmai  GTGCAATAAAATTTCGGTGCCCATGGAACAAATCAAAATTCTAGTGGAACAAAAGTTTTAACTAAGTGA
```

4,840 4,850 4,860 4,870 4,880 4,890

Hulutou ATGAGCAAAGTATATCACTTGCAGGAACAAATTGAAACTACTACTGCTGCCACTTCACCACTTCTTGTG
Baihulu ATGAGCAAAGTATATCACTTGCAGGAACAAATTGAAACTACTACTGCTGCCACTTCACCACTTCTTGTG
Chiyacao ATGAGCAAAGTATATCACTTGCAGGAACAAATTGAAACTACTACTGCTGCCACTTCACCACTTCTTGTG
Hongmangmai ATGAGCAAAGTATATCACTTGCAGGAACAAATTGAAACTACTACTGCTGCCACTTCACCACTTCTTGTG

4,900 4,910 4,920 4,930 4,940 4,950 4,960

Hulutou CTGACTTTGCCATCCTCACGTGGTACCAATTTCCTTCAGTTTTCGTGTGTGCCAATGAATTCAGATTTC
Baihulu CTGACTTTGCCATCCTCACGTGGTACCAATTTCCTTCAGTTTTCGTGTGTGCCAATGAATTCAGATTTC
Chiyacao CTGACTTTGCCATCCTCACGTGGTACCAATTTCCTTCAGTTTTCGTGTGTGCCAATGAATTCAGATTTC
Hongmangmai CTGACTTTGCCATCCTCACGTGGTACCAATTTCCTTCAGTTTTCGTGTGTGCCAATGAATTCAGATTTC

4,970 4,980 4,990 5,000 5,010 5,020 5,030

Hulutou CAAACAAGTGGAGGTTTTTTCTTCCTTCCCCAATATCCCTCGTTCTACCCAACTATATAATGGGGCAGA
Baihulu CAAACAAGTGGAGGTTTTTTCTTCCTTCCCCAATATCCCTCGTTCTACCCAACTATATAATGGGGCAGA
Chiyacao CAAACAAGTGGAGGTTTTTTCTTCCTTCCCCAATATCCCTCGTTCTACCCAACTATATAATGGGGCAGA
Hongmangmai CAAACAAGTGGAGGTTTTTTCTTCCTTCCCCAATATCCCTCGTTCTACCCAACTATATAATGGGGCAGA

5,040 5,050 5,060 5,070 5,080 5,090 5,100

Hulutou GAAGGATAAGCAAAGTAGGCGGTAGAACAAAAAATTAATGTAATTGGGACAAAATTTAATCAGTAAATG
Baihulu GAAGGATAAGCAAAGTAGGCGGTAGAACAAAAAATTAATGTAATTGGGACAAAATTTAATCAGTAAATG
Chiyacao GAAGGATAAGCAAAGTAGGCGGTAGAACAAAAAATTAATGTAATTGGGACAAAATTTAATCAGTAAATG
Hongmangmai GAAGGATAAGCAAAGTAGGCGGTAGAACAAAAAATTAATGTAATTGGGACAAAATTTAATCAGTAAATG

5,110 5,120 5,130 5,140 5,150 5,160 5,170

Hulutou CTCACTAGTCTGAGGATAAACGAAAGCATGAAACAAGGAAAAGATGAGGATGAGCAACTATTGGAATAA
Baihulu CTCACTAGTCTGAGGATAAACGAAAGCATGAAACAAGGAAAAGATGAGGATGAGCAACTATTGGAATAA
Chiyacao CTCACTAGTCTGAGGATAAACGAAAGCATGAAACAAGGAAAAGATGAGGATGAGCAACTATTGGAATAA
Hongmangmai CTCACTAGTCTGAGGATAAACGAAAGCATGAAACAAGGAAAAGATGAGGATGAGCAACTATTGGAATAA

5,180 5,190 5,200 5,210 5,220 5,230 5,240

Hulutou AAGCAAAATTAAACTAGCAGAGCAAAGTAAAGTTGCAACCAAATCATCAGCGAGCACCACATCAGCGCG
Baihulu AAGCAAAATTAAACTAGCAGAGCAAAGTAAAGTTGCAACCAAATCATCAGCGAGCACCACATCAGCGCG
Chiyacao AAGCAAAATTAAACTAGCAGAGCAAAGTAAAGTTGCAACCAAATCATCAGCGAGCACCACATCAGCGCG
Hongmangmai AAGCAAAATTAAACTAGCAGAGCAAAGTAAAGTTGCAACCAAATCATCAGCGAGCACCACATCAGCGCG

5,250 5,260 5,270 5,280 5,290 5,300 5,310

Hulutou CTAGTCTCCCCTCCCGGTGGTAACCGCTATTGGTGGCTCACCGGCGTCTAAGGCCCCTAGGCGGATGAA
Baihulu CTAGTCTCCCCTCCCGGTGGTAACCGCTATTGGTGGCTCACCGGCGTCTAAGGCCCCTAGGCGGATGAA
Chiyacao CTAGTCTCCCCTCCCGGTGGTAACCGCTATTGGTGGCTCACCGGCGTCTAAGGCCCCTAGGCGGATGAA
Hongmangmai CTAGTCTCCCCTCCCGGTGGTAACCGCTATTGGTGGCTCACCGGCGTCTAAGGCCCCTAGGCGGATGAA

5,320 5,330 5,340 5,350 5,360 5,370 5,380

Hulutou GACTAGATGGCTTTCCTTGCCGATCTACATGACATCTGGCCCTTGGCTTATTGGTGGCGATTTCAACAT
Baihulu GACTAGATGGCTTTCCTTGCCGATCTACATGACATCTGGCCCTTGGCTTATTGGTGGCGATTTCAACAT
Chiyacao GACTAGATGGCTTTCCTTGCCGATCTACATGACATCTGGCCCTTGGCTTATTGGTGGCGATTTCAACAT
Hongmangmai GACTAGATGGCTTTCCTTGCCGATCTACATGACATCTGGCCCTTGGCTTATTGGTGGCGATTTCAACAT

5,390 5,400 5,410 5,420 5,430 5,440 5,450

Hulutou GATCGTCTCTGAGGCGGACAAGAATAACCCGCACGTGAACCGGCGGATCATGCATAGCTTTCGATGCTT
Baihulu GATCGTCTCTGAGGCGGACAAGAATAACCCGCACGTGAACCGGCGGATCATGCATAGCTTTCGATGCTT
Chiyacao GATCGTCTCTGAGGCGGACAAGAATAACCCGCACGTGAACCGGCGGATCATGCATAGCTTTCGATGCTT
Hongmangmai GATCGTCTCTGAGGCGGACAAGAATAACCCGCACGTGAACCGGCGGATCATGCATAGCTTTCGATGCTT

5,460 5,470 5,480 5,490 5,500 5,510 5,520

Hulutou CCTTGTCGACGAGGAACTTCATGACATTTGTATGCATGGTAGGTGCTACACATGGTCCAGCGAGCGCAA
Baihulu CCTTGTCGACGAGGAACTTCATGACATTTGTATGCATGGTAGGTGCTACACATGGTCCAGCGAGCGCAA
Chiyacao CCTTGTCGACGAGGAACTTCATGACATTTGTATGCATGGTAGGTGCTACACATGGTCCAGCGAGCGCAA
Hongmangmai CCTTGTCGACGAGGAACTTCATGACATTTGTATGCATGGTAGGTGCTACACATGGTCCAGCGAGCGCAA

```
              5,530     5,540     5,550     5,560     5,570     5,580
Hulutou       GATTCCTACTCTTGTCAAGAACAACCGCATCGTCTCCTCCTCCTCGAAGGATGCGCATCCCCACTGCCT
Baihulu       GATTCCTACTCTTGTCAAGAACAACCGCATCGTCTCCTCCTCCTCGAAGGATGCGCATCCCCACTGCCT
Chiyacao      GATTCCTACTCTTGTCAAGAACAACCGCATCGTCTCCTCCTCCTCGAAGGATGCGCATCCCCACTGCCT
Hongmangmai   GATTCCTACTCTTGTCAAGAACAACCGCATCGTCTCCTCCTCCTCGAAGGATGCGCATCCCCACTGCCT
              5,590     5,600     5,610     5,620     5,630     5,640     5,650
Hulutou       GCTGTAGTGCCTCTATTCAGCTGCTTCAGAGCACTGGCCGCTCCTTCTTGATTGTGTCCCGTGTACCCA
Baihulu       GCTGTAGTGCCTCTATTCAGCTGCTTCAGAGCACTGGCCGCTCCTTCTTGATTGTGTCCCGTGTACCCA
Chiyacao      GCTGTAGTGCCTCTATTCAGCTGCTTCAGAGCACTGGCCGCTCCTTCTTGATTGTGTCCCGTGTACCCA
Hongmangmai   GCTGTAGTGCCTCTATTCAGCTGCTTCAGAGCACTGGCCGCTCCTTCTTGATTGTGTCCCGTGTACCCA
              5,660     5,670     5,680     5,690     5,700     5,710     5,720
Hulutou       TGGGATCAAGCGGTTCCACTTCGAGTGATTTTGGCCGAAGCTTGATGATTTCCAACTAGTCGCCTCTGA
Baihulu       TGGGATCAAGCGGTTCCACTTCGAGTGATTTTGGCCGAAGCTTGATGATTTCCAACTAGTCGCCTCTGA
Chiyacao      TGGGATCAAGCGGTTCCACTTCGAGTGATTTTGGCCGAAGCTTGATGATTTCCAACTAGTCGCCTCTGA
Hongmangmai   TGGGATCAAGCGGTTCCACTTCGAGTGATTTTGGCCGAAGCTTGATGATTTCCAACTAGTCGCCTCTGA
              5,730     5,740     5,750     5,760     5,770     5,780     5,790
Hulutou       GGCTTGGAACTCCATTGAGCATGCCCCTGATGCATTTCGCCATATCGTCGTGCGCCTTAAGTCTACCAC
Baihulu       GGCTTGGAACTCCATTGAGCATGCCCCTGATGCATTTCGCCATATCGTCGTGCGCCTTAAGTCTACCAC
Chiyacao      GGCTTGGAACTCCATTGAGCATGCCCCTGATGCATTTCGCCATATCGTCGTGCGCCTTAAGTCTACCAC
Hongmangmai   GGCTTGGAACTCCATTGAGCATGCCCCTGATGCATTTCGCCATATCGTCGTGCGCCTTAAGTCTACCAC
              5,800     5,810     5,820     5,830     5,840     5,850     5,860
Hulutou       ACGTCGTCTCCAGAGCTGGAGTGCCAAGTCTATTGGCCAAATCTCGGTGCAGCTTCAGATCGCGTGTGA
Baihulu       ACGTCGTCTCCAGAGCTGGAGTGCCAAGTCTATTGGCCAAATCTCGGTGCAGCTTCAGATCGCGTGTGA
Chiyacao      ACGTCGTCTCCAGAGCTGGAGTGCCAAGTCTATTGGCCAAATCTCGGTGCAGCTTCAGATCGCGTGTGA
Hongmangmai   ACGTCGTCTCCAGAGCTGGAGTGCCAAGTCTATTGGCCAAATCTCGGTGCAGCTTCAGATCGCGTGTGA
              5,870     5,880     5,890     5,900     5,910     5,920     5,930
Hulutou       GCTCATTGCTCATTTGGACGCCGCTCAGCACTTTCGGCCGCTCTCGACCTCGGAGGCCTGGTTGCGACA
Baihulu       GCTCATTGCTCATTTGGACGCCGCTCAGCACTTTCGGCCGCTCTCGACCTCGGAGGCCTGGTTGCGACA
Chiyacao      GCTCATTGCTCATTTGGACGCCGCTCAGCACTTTCGGCCGCTCTCGACCTCGGAGGCCTGGTTGCGACA
Hongmangmai   GCTCATTGCTCATTTGGACGCCGCTCAGCACTTTCGGCCGCTCTCGACCTCGGAGGCCTGGTTGCGACA
              5,940     5,950     5,960     5,970     5,980     5,990     6,000
Hulutou       TAGGCTGAAGGCCGCCTACCTTGGCCTTGCCTCGCTCGAGTGTTCCATCATTCGGCAACGCACGAGGTT
Baihulu       TAGGCTGAAGGCCGCCTACCTTGGCCTTGCCTCGCTCGAGTGTTCCATCATTCGGCAACGCACGAGGTT
Chiyacao      TAGGCTGAAGGCCGCCTACCTTGGCCTTGCCTCGCTCGAGTGTTCCATCATTCGGCAACGCACGAGGTT
Hongmangmai   TAGGCTGAAGGCCGCCTACCTTGGCCTTGCCTCGCTCGAGTGTTCCATCATTCGGCAACGCACGAGGTT
              6,010     6,020     6,030     6,040     6,050     6,060     6,070
Hulutou       TGCCTACGAGGTTTGCCTGTCTCAGGGAGGCTGATGCTGGCCCTACCTTCTTCAACATGCATGCATGCC
Baihulu       TGCCTACGAGGTTTGCCTGTCTCAGGGAGGCTGATGCTGGCCCTACCTTCTTCAACATGCATGCATGCC
Chiyacao      TGCCTACGAGGTTTGCCTGTCTCAGGGAGGCTGATGCTGGCCCTACCTTCTTCAACATGCATGCATGCC
Hongmangmai   TGCCTACGAGGTTTGCCTGTCTCAGGGAGGCTGATGCTGGCCCTACCTTCTTCAACATGCATGCATGCC
              6,080     6,090     6,100     6,110     6,120     6,130     6,140
Hulutou       TCGTACCATAAGCAGAAAAACCGCATCTTCGAGCTCAAGGTGGGTGATAGAGTTGTCTCCACGCCGGAG
Baihulu       TCGTACCATAAGCAGAAAAACCGCATCTTCGAGCTCAAGGTGGGTGATAGAGTTGTCTCCACGCCGGAG
Chiyacao      TCGTACCATAAGCAGAAAAACCGCATCTTCGAGCTCAAGGTGGGTGATAGAGTTGTCTCCACGCCGGAG
Hongmangmai   TCGTACCATAAGCAGAAAAACCGCATCTTCGAGCTCAAGGTGGGTGATAGAGTTGTCTCCACGCCGGAG
              6,150     6,160     6,170     6,180     6,190     6,200     6,210
Hulutou       GCGTTTGCCACTGCGGGATTTTGATCACTTCACCGGTGCCCTTGAGACCTCCGAGGACCGCGACTTCTC
Baihulu       GCGTTTGCCACTGCGGGATTTTGATCACTTCACCGGTGCCCTTGAGACCTCCGAGGACCGCGACTTCTC
Chiyacao      GCGTTTGCCACTGCGGGATTTTGATCACTTCACCGGTGCCCTTGAGACCTCCGAGGACCGCGACTTCTC
Hongmangmai   GCGTTTGCCACTGCGGGATTTTGATCACTTCACCGGTGCCCTTGAGACCTCCGAGGACCGCGACTTCTC
```

```
                        6,220     6,230     6,240     6,250     6,260     6,270
Hulutou      CATCTCCCTTCGGGATCTGCATGCTGGTGCTTTCGATATCAAGGCCCTTGATGAGCCATTTTTTTAGGC
Baihulu      CATCTCCCTTCGGGATCTGCATGCTGGTGCTTTCGATATCAAGGCCCTTGATGAGCCATTTTTTTAGGC
Chiyacao     CATCTCCCTTCGGGATCTGCATGCTGGTGCTTTCGATATCAAGGCCCTTGATGAGCCATTTTTTTAGGC
Hongmangmai  CATCTCCCTTCGGGATCTGCATGCTGGTGCTTTCGATATCAAGGCCCTTGATGAGCCATTTTTTTAGGC

             6,280     6,290     6,300     6,310     6,320     6,330     6,340
Hulutou      CGAAATTTGGCGCGTTGTCAAGGCTCTACCCTCCAGTAAGACGCCGGGGCCATCCCTCAATGCGTGAGG
Baihulu      CGAAATTTGGCGCGTTGTCAAGGCTCTACCCTCCAGTAAGACGCCGGGGCCATCCCTCAATGCGTGAGG
Chiyacao     CGAAATTTGGCGCGTTGTCAAGGCTCTACCCTCCAGTAAGACGCCGGGGCCATCCCTCAATGCGTGAGG
Hongmangmai  CGAAATTTGGCGCGTTGTCAAGGCTCTACCCTCCAGTAAGACGCCGGGGCCATCCCTCAATGCGTGAGG

             6,350     6,360     6,370     6,380     6,390     6,400     6,410
Hulutou      CTTTCAAAAACTCAACGAGGCATACATTACCCTGCTCCCCAAACGGCATGCCGACGCAACCACACTCTT
Baihulu      CTTTCAAAAACTCAACGAGGCATACATTACCCTGCTCCCCAAACGGCATGCCGACGCAACCACACTCTT
Chiyacao     CTTTCAAAAACTCAACGAGGCATACATTACCCTGCTCCCCAAACGGCATGCCGACGCAACCACACTCTT
Hongmangmai  CTTTCAAAAACTCAACGAGGCATACATTACCCTGCTCCCCAAACGGCATGCCGACGCAACCACACTCTT

              6,420     6,430     6,440     6,450     6,460     6,470     6,480
Hulutou      CTACTTTTGGTCCATTAGCCTCATCCATCTGATGGCGAAGTTGTTCGCCAAGGCGCTCTCTCTCTCTCT
Baihulu      CTACTTTTGGTCCATTAGCCTCATCCATCTGATGGCGAAGTTGTTCGCCAAGGCGCTCTCTCTCTCTCT
Chiyacao     CTACTTTTGGTCCATTAGCCTCATCCATCTGATGGCGAAGTTGTTCGCCAAGGCGCTCTCTCTCTCTCT
Hongmangmai  CTACTTTTGGTCCATTAGCCTCATCCATCTGATGGCGAAGTTGTTCGCCAAGGCGCTCTCTCTCTCTCT

               6,490     6,500     6,510     6,520     6,530     6,540     6,550
Hulutou      CTCTCTCTCTCGCTCTCTCTCTCTCTCTGCAAACGGCGTGTGTTCTGCACAATCTCAAGGTTCCTCGGG
Baihulu      CTCTCTCTCTCGCTCTCTCTCTCTCTCTGCAAACGGCGTGTGTTCTGCACAATCTCAAGGTTCCTCGGG
Chiyacao     CTCTCTCTCTCGCTCTCTCTCTCTCTCTGCAAACGGCGTGTGTTCTGCACAATCTCAAGGTTCCTCGGG
Hongmangmai  CTCTCTCTCTCGCTCTCTCTCTCTCTCTGCAAACGGCGTGTGTTCTGCACAATCTCAAGGTTCCTCGGG

                6,560     6,570     6,580     6,590     6,600     6,610     6,620
Hulutou      TGATGCTGAAGCTTGACATCGTTAGGGCCTTCGACTCGGTGTCTTAGCCCTTTCTTCTGGCCCGAATGT
Baihulu      TGATGCTGAAGCTTGACATCGTTAGGGCCTTCGACTCGGTGTCTTAGCCCTTTCTTCTGGCCCGAATGT
Chiyacao     TGATGCTGAAGCTTGACATCGTTAGGGCCTTCGACTCGGTGTCTTAGCCCTTTCTTCTGGCCCGAATGT
Hongmangmai  TGATGCTGAAGCTTGACATCGTTAGGGCCTTCGACTCGGTGTCTTAGCCCTTTCTTCTGGCCCGAATGT

                 6,630     6,640     6,650     6,660     6,670     6,680     6,690
Hulutou      CCCTCTCGTCCGCAGAAAGGGCGTGTGATTCTTGGGCAATGTCAAGGCGAAGGATAACGAGAAGGGCTG
Baihulu      CCCTCTCGTCCGCAGAAAGGGCGTGTGATTCTTGGGCAATGTCAAGGCGAAGGATAACGAGAAGGGCTG
Chiyacao     CCCTCTCGTCCGCAGAAAGGGCGTGTGATTCTTGGGCAATGTCAAGGCGAAGGATAACGAGAAGGGCTG
Hongmangmai  CCCTCTCGTCCGCAGAAAGGGCGTGTGATTCTTGGGCAATGTCAAGGCGAAGGATAACGAGAAGGGCTG

                  6,700     6,710     6,720     6,730     6,740     6,750     6,760
Hulutou      CATGAAGATGCAACTTTGCCTTGGAGAAGAGGCCCTTACTCCATCTCGCCAAGTGCCACCCCGTTTTCC
Baihulu      CATGAAGATGCAACTTTGCCTTGGAGAAGAGGCCCTTACTCCATCTCGCCAAGTGCCACCCCGTTTTCC
Chiyacao     CATGAAGATGCAACTTTGCCTTGGAGAAGAGGCCCTTACTCCATCTCGCCAAGTGCCACCCCGTTTTCC
Hongmangmai  CATGAAGATGCAACTTTGCCTTGGAGAAGAGGCCCTTACTCCATCTCGCCAAGTGCCACCCCGTTTTCC

                   6,770     6,780     6,790     6,800     6,810     6,820     6,830
Hulutou      TGAGCTTATGGAAAAGCCGCTGGCAAGGCTCCGTGTGCTCGCATGGCTCATCCCAAGCCTTTGACACGA
Baihulu      TGAGCTTATGGAAAAGCCGCTGGCAAGGCTCCGTGTGCTCGCATGGCTCATCCCAAGCCTTTGACACGA
Chiyacao     TGAGCTTATGGAAAAGCCGCTGGCAAGGCTCCGTGTGCTCGCATGGCTCATCCCAAGCCTTTGACACGA
Hongmangmai  TGAGCTTATGGAAAAGCCGCTGGCAAGGCTCCGTGTGCTCGCATGGCTCATCCCAAGCCTTTGACACGA

                    6,840     6,850     6,860     6,870     6,880     6,890     6,900
Hulutou      TGTCAAAGAAGCCGGGGAGACAGGACAGAAAATTCTCAAATTTGAAACATCTTGGTCGCTTGGGCCCCT
Baihulu      TGTCAAAGAAGCCGGGGAGACAGGACAGAAAATTCTCAAATTTGAAACATCTTGGTCGCTTGGGCCCCT
Chiyacao     TGTCAAAGAAGCCGGGGAGACAGGACAGAAAATTCTCAAATTTGAAACATCTTGGTCGCTTGGGCCCCT
Hongmangmai  TGTCAAAGAAGCCGGGGAGACAGGACAGAAAATTCTCAAATTTGAAACATCTTGGTCGCTTGGGCCCCT
```

```
            6,910     6,920     6,930     6,940     6,950     6,960
Hulutou     TGTCATCAGCGAGGAGGAGAGGGCAGTGATCCGAAAGTGAATAGGAGATGGAGTGGAGCACATGGGAGT
Baihulu     TGTCATCAGCGAGGAGGAGAGGGCAGTGATCCGAAAGTGAATAGGAGATGGAGTGGAGCACATGGGAGT
Chiyacao    TGTCATCAGCGAGGAGGAGAGGGCAGTGATCCGAAAGTGAATAGGAGATGGAGTGGAGCACATGGGAGT
Hongmangmai TGTCATCAGCGAGGAGGAGAGGGCAGTGATCCGAAAGTGAATAGGAGATGGAGTGGAGCACATGGGAGT
            6,970     6,980     6,990     7,000     7,010     7,020     7,030
Hulutou     TAAAATGCATGTCCCAATCGGCATTTCGGAACATCAAGTCAAGCTTGCAAAGAGTTGGGTTTTGACGCT
Baihulu     TAAAATGCATGTCCCAATCGGCATTTCGGAACATCAAGTCAAGCTTGCAAAGAGTTGGGTTTTGACGCT
Chiyacao    TAAAATGCATGTCCCAATCGGCATTTCGGAACATCAAGTCAAGCTTGCAAAGAGTTGGGTTTTGACGCT
Hongmangmai TAAAATGCATGTCCCAATCGGCATTTCGGAACATCAAGTCAAGCTTGCAAAGAGTTGGGTTTTGACGCT
            7,040     7,050     7,060     7,070     7,080     7,090     7,100
Hulutou     CGTTGCTCCAAGTGAATCGTATGTTTTGCATTACCATGCCTTCTATTTTTATCTCAAGCACGATAGATT
Baihulu     CGTTGCTCCAAGTGAATCGTATGTTTTGCATTACCATGCCTTCTATTTTTATCTCAAGCACGATAGATT
Chiyacao    CGTTGCTCCAAGTGAATCGTATGTTTTGCATTACCATGCCTTCTATTTTTATCTCAAGCACGATAGATT
Hongmangmai CGTTGCTCCAAGTGAATCGTATGTTTTGCATTACCATGCCTTCTATTTTTATCTCAAGCACGATAGATT
            7,110     7,120     7,130     7,140     7,150     7,160     7,170
Hulutou     TGGTTGAAGTCGCCCATGACAATCCATTTAGTGCCATTGTTGGGTTTTTGTGAGATTAGCTCGTTAAAG
Baihulu     TGGTTGAAGTCGCCCATGACAATCCATTTAGTGCCATTGTTGGGTTTTTGTGAGATTAGCTCGTTAAAG
Chiyacao    TGGTTGAAGTCGCCCATGACAATCCATTTAGTGCCATTGTTGGGTTTTTGTGAGATTAGCTCGTTAAAG
Hongmangmai TGGTTGAAGTCGCCCATGACAATCCATTTAGTGCCATTGTTGGGTTTTTGTGAGATTAGCTCGTTAAAG
            7,180     7,190     7,200     7,210     7,220     7,230     7,240
Hulutou     AAGTCACCCTTTCTGTCGGAGGCCGTGGGAACATAGACACTAGTGATCATGAAGTAGGTATCGGTGTGA
Baihulu     AAGTCACCCTTTCTGTCGGAGGCCGTGGGAACATAGACACTAGTGATCATGAAGTAGGTATCGGTGTGA
Chiyacao    AAGTCACCCTTTCTGTCGGAGGCCGTGGGAACATAGACACTAGTGATCATGAAGTAGGTATCGGTGTGA
Hongmangmai AAGTCACCCTTTCTGTCGGAGGCCGTGGGAACATAGACACTAGTGATCATGAAGTAGGTATCGGTGTGA
            7,250     7,260     7,270     7,280     7,290     7,300     7,310
Hulutou     ATCACATTGACCATTGCCGAGAGGCAGAACTCAGTAAACTTGACGTCTGAAATACGGACGACACTATCG
Baihulu     ATCACATTGACCATTGCCGAGAGGCAGAACTCAGTAAACTTGACGTCTGAAATACGGACGACACTATCG
Chiyacao    ATCACATTGACCATTGCCGAGAGGCAGAACTCAGTAAACTTGACGTCTGAAATACGGACGACACTATCG
Hongmangmai ATCACATTGACCATTGCCGAGAGGCAGAACTCAGTAAACTTGACGTCTGAAATACGGACGACACTATCG
            7,320     7,330     7,340     7,350     7,360     7,370     7,380
Hulutou     TCCCAAAGCATGAGTAGGCCCCCTCTGGTTCCAATAGCCGGACGCTGACAAAACTCGAGAGCCTATTGC
Baihulu     TCCCAAAGCATGAGTAGGCCCCCTCTGGTTCCAATAGCCGGACGCTGACAAAACTCGAGAGCCTATTGC
Chiyacao    TCCCAAAGCATGAGTAGGCCCCCTCTGGTTCCAATAGCCGGACGCTGACAAAACTCGAGAGCCTATTGC
Hongmangmai TCCCAAAGCATGAGTAGGCCCCCTCTGGTTCCAATAGCCGGACGCTGACAAAACTCGAGAGCCTATTGC
            7,390     7,400     7,410     7,420     7,430     7,440     7,450
Hulutou     CGCCGAGGTAGGAGGCGATGAATCGGTCCACTGATTCCATCTTGGACTCCTGAAAGCAAACAATGTGGC
Baihulu     CGCCGAGGTAGGAGGCGATGAATCGGTCCACTGATTCCATCTTGGACTCCTGAAAGCAAACAATGTGGC
Chiyacao    CGCCGAGGTAGGAGGCGATGAATCGGTCCACTGATTCCATCTTGGACTCCTGAAAGCAAACAATGTGGC
Hongmangmai CGCCGAGGTAGGAGGCGATGAATCGGTCCACTGATTCCATCTTGGACTCCTGAAAGCAAACAATGTGGC
            7,460     7,470     7,480     7,490     7,500     7,510     7,520
Hulutou     AAGAGGAGGAGGCAATCGTCTCATGAACCGTCGCCCGTCTACCTGGGTTGCCAAATTTTGATAATTGGT
Baihulu     AAGAGGAGGAGGCAATCGTCTCATGAACCGTCGCCCGTCTACCTGGGTTGCCAAATTTTGATAATTGGT
Chiyacao    AAGAGGAGGAGGCAATCGTCTCATGAACCGTCGCCCGTCTACCTGGGTTGCCAAATTTTGATAATTGGT
Hongmangmai AAGAGGAGGAGGCAATCGTCTCATGAACCGTCGCCCGTCTACCTGGGTTGCCAAATTTTGATAATTGGT
            7,530     7,540     7,550     7,560     7,570     7,580     7,590
Hulutou     TTGATGCCAAAAATTGGCATGCCAAATTTTGGCAACTGCCAAATATTGGCTAGAGATTGATTGCTTGCC
Baihulu     TTGATGCCAAAAATTGGCATGCCAAATTTTGGCAACTGCCAAATATTGGCTAGAGATTGATTGCTTGCC
Chiyacao    TTGATGCCAAAAATTGGCATGCCAAATTTTGGCAACTGCCAAATATTGGCTAGAGATTGATTGCTTGCC
Hongmangmai TTGATGCCAAAAATTGGCATGCCAAATTTTGGCAACTGCCAAATATTGGCTAGAGATTGATTGCTTGCC
```

```
                 7,600     7,610     7,620     7,630     7,640     7,650
Hulutou      AATTTTCATTGCCAATCTCAGAAAAAGTTGTCAAATGTGAAGTTGCCAACATTTGACAATAAAAATTGG
Baihulu      AATTTTCATTGCCAATCTCAGAAAAAGTTGTCAAATGTGAAGTTGCCAACATTTGACAATAAAAATTGG
Chiyacao     AATTTTCATTGCCAATCTCAGAAAAAGTTGTCAAATGTGAAGTTGCCAACATTTGACAATAAAAATTGG
Hongmangmai  AATTTTCATTGCCAATCTCAGAAAAAGTTGTCAAATGTGAAGTTGCCAACATTTGACAATAAAAATTGG

             7,660     7,670     7,680     7,690     7,700     7,710     7,720
Hulutou      CAAGCAACCAATCTCTAGTTAATATTTGGCACTTGCCAAAATTTGGCATGCCAACTTTTAGCATCAAAC
Baihulu      CAAGCAACCAATCTCTAGTTAATATTTGGCACTTGCCAAAATTTGGCATGCCAACTTTTAGCATCAAAC
Chiyacao     CAAGCAACCAATCTCTAGTTAATATTTGGCACTTGCCAAAATTTGGCATGCCAACTTTTAGCATCAAAC
Hongmangmai  CAAGCAACCAATCTCTAGTTAATATTTGGCACTTGCCAAAATTTGGCATGCCAACTTTTAGCATCAAAC

             7,730     7,740     7,750     7,760     7,770     7,780     7,790
Hulutou      CAATTATGCTCTTAACCCTTGCAGATTTCAACTCAAAAAAAATTATGTTTTGTTCTATCATCAGGAAAC
Baihulu      CAATTATGCTCTTAACCCTTGCAGATTTCAACTCAAAAAAAATTATGTTTTGTTCTATCATCAGGAAAC
Chiyacao     CAATTATGCTCTTAACCCTTGCAGATTTCAACTCAAAAAAAATTATGTTTTGTTCTATCATCAGGAAAC
Hongmangmai  CAATTATGCTCTTAACCCTTGCAGATTTCAACTCAAAAAAAATTATGTTTTGTTCTATCATCAGGAAAC

             7,800     7,810     7,820     7,830     7,840     7,850     7,860
Hulutou      TAGGGGTCTCGTGGCACTCACAATTAGAGCCCGTCGGGGTTGCATGAAGTGTGCTTGCTGAACCAGCTC
Baihulu      TAGGGGTCTCGTGGCACTCACAATTAGAGCCCGTCGGGGTTGCATGAAGTGTGCTTGCTGAACCAGCTC
Chiyacao     TAGGGGTCTCGTGGCACTCACAATTAGAGCCCGTCGGGGTTGCATGAAGTGTGCTTGCTGAACCAGCTC
Hongmangmai  TAGGGGTCTCGTGGCACTCACAATTAGAGCCCGTCGGGGTTGCATGAAGTGTGCTTGCTGAACCAGCTC

             7,870     7,880     7,890     7,900     7,910     7,920     7,930
Hulutou      TTTGCACAACAAAGAGAGAAATAATCTGGAAGCTGCGTGGAGTTTGAGGTCAGGGTGGCTGCTGTCTAG
Baihulu      TTTGCACAACAAAGAGAGAAATAATCTGGAAGCTGCGTGGAGTTTGAGGTCAGGGTGGCTGCTGTCTAG
Chiyacao     TTTGCACAACAAAGAGAGAAATAATCTGGAAGCTGCGTGGAGTTTGAGGTCAGGGTGGCTGCTGTCTAG
Hongmangmai  TTTGCACAACAAAGAGAGAAATAATCTGGAAGCTGCGTGGAGTTTGAGGTCAGGGTGGCTGCTGTCTAG

             7,940     7,950     7,960     7,970     7,980     7,990     8,000
Hulutou      AGTAGAGGTTGCAACTTGCAAGCGATCTTTGCACAACCATGATCTTCATTAAGCACTGGCGACGATGAA
Baihulu      AGTAGAGGTTGCAACTTGCAAGCGATCTTTGCACAACCATGATCTTCATTAAGCACTGGCGACGATGAA
Chiyacao     AGTAGAGGTTGCAACTTGCAAGCGATCTTTGCACAACCATGATCTTCATTAAGCACTGGCGACGATGAA
Hongmangmai  AGTAGAGGTTGCAACTTGCAAGCGATCTTTGCACAACCATGATCTTCATTAAGCACTGGCGACGATGAA

             8,010     8,020     8,030     8,040     8,050     8,060     8,070
Hulutou      CACCCAAGTTACAACCAACAGGAACGTTACAGCAACTAGTTCCTCTTGGTTAAGGGATAACAGAGAGCT
Baihulu      CACCCAAGTTACAACCAACAGGAACGTTACAGCAACTAGTTCCTCTTGGTTAAGGGATAACAGAGAGCT
Chiyacao     CACCCAAGTTACAACCAACAGGAACGTTACAGCAACTAGTTCCTCTTGGTTAAGGGATAACAGAGAGCT
Hongmangmai  CACCCAAGTTACAACCAACAGGAACGTTACAGCAACTAGTTCCTCTTGGTTAAGGGATAACAGAGAGCT

             8,080     8,090     8,100     8,110     8,120     8,130     8,140
Hulutou      TTGCTAACATCAGGGCAACTAGTGCTTTGGCTCTGTGAAGTGGGGAGAAACAATGGAGGCACTTGCCCA
Baihulu      TTGCTAACATCAGGGCAACTAGTGCTTTGGCTCTGTGAAGTGGGGAGAAACAATGGAGGCACTTGCCCA
Chiyacao     TTGCTAACATCAGGGCAACTAGTGCTTTGGCTCTGTGAAGTGGGGAGAAACAATGGAGGCACTTGCCCA
Hongmangmai  TTGCTAACATCAGGGCAACTAGTGCTTTGGCTCTGTGAAGTGGGGAGAAACAATGGAGGCACTTGCCCA

             8,150     8,160     8,170     8,180     8,190     8,200     8,210
Hulutou      CCAATTGAGGGGGGATTTGAGGCCGCCTGTGAGGAGCTGGAGGGGGGGTTTGGCAGGCCAGGGGAGGGG
Baihulu      CCAATTGAGGGGGGATTTGAGGCCGCCTGTGAGGAGCTGGAGGGGGGGTTTGGCAGGCCAGGGGAGGGG
Chiyacao     CCAATTGAGGGGGGATTTGAGGCCGCCTGTGAGGAGCTGGAGGGGGGGTTTGGCAGGCCAGGGGAGGGG
Hongmangmai  CCAATTGAGGGGGGATTTGAGGCCGCCTGTGAGGAGCTGGAGGGGGGGTTTGGCAGGCCAGGGGAGGGG

             8,220     8,230     8,240     8,250     8,260     8,270     8,280
Hulutou      AGGGGAGGTTGTCGCTGCGTCCAGCCAGGCTCCAGGGGGCATCCTGTACTTCCTCTGCAAGGCGATGCG
Baihulu      AGGGGAGGTTGTCGCTGCGTCCAGCCAGGCTCCAGGGGGCATCCTGTACTTCCTCTGCAAGGCGATGCG
Chiyacao     AGGGGAGGTTGTCGCTGCGTCCAGCCAGGCTCCAGGGGGCATCCTGTACTTCCTCTGCAAGGCGATGCG
Hongmangmai  AGGGGAGGTTGTCGCTGCGTCCAGCCAGGCTCCAGGGGGCATCCTGTACTTCCTCTGCAAGGCGATGCG
```

```
            8,290     8,300     8,310     8,320     8,330     8,340
Hulutou     CGGGGGCGGCGTTTGCAGCGGAAGACCCGGGGCGGGGTTGCCGCCCGGGCATTGATGGCGGCCTCCTCT
Baihulu     CGGGGGCGGCGTTTGCAGCGGAAGACCCGGGGCGGGGTTGCCGCCCGGGCATTGATGGCGGCCTCCTCT
Chiyacao    CGGGGGCGGCGTTTGCAGCGGAAGACCCGGGGCGGGGTTGCCGCCCGGGCATTGATGGCGGCCTCCTCT
Hongmangmai CGGGGGCGGCGTTTGCAGCGGAAGACCCGGGGCGGGGTTGCCGCCCGGGCATTGATGGCGGCCTCCTCT
            8,350     8,360     8,370     8,380     8,390     8,400     8,410
Hulutou     TGAATGCGTTGCAGAGACGCGTCGGGGGCGACGATGATGGAAGCGACGCGCACGCCTCCCCCTCGAAGG
Baihulu     TGAATGCGTTGCAGAGACGCGTCGGGGGCGACGATGATGGAAGCGACGCGCACGCCTCCCCCTCGAAGG
Chiyacao    TGAATGCGTTGCAGAGACGCGTCGGGGGCGACGATGATGGAAGCGACGCGCACGCCTCCCCCTCGAAGG
Hongmangmai TGAATGCGTTGCAGAGACGCGTCGGGGGCGACGATGATGGAAGCGACGCGCACGCCTCCCCCTCGAAGG
            8,420     8,430     8,440     8,450     8,460     8,470     8,480
Hulutou     ACAGGCGGCGTCTCCAGAACCTCGTCAGCAAGCTCAGCTGCCTGGATCCGGAGACGTGCAGCGGCCGAT
Baihulu     ACAGGCGGCGTCTCCAGAACCTCGTCAGCAAGCTCAGCTGCCTGGATCCGGAGACGTGCAGCGGCCGAT
Chiyacao    ACAGGCGGCGTCTCCAGAACCTCGTCAGCAAGCTCAGCTGCCTGGATCCGGAGACGTGCAGCGGCCGAT
Hongmangmai ACAGGCGGCGTCTCCAGAACCTCGTCAGCAAGCTCAGCTGCCTGGATCCGGAGACGTGCAGCGGCCGAT
            8,490     8,500     8,510     8,520     8,530     8,540     8,550
Hulutou     CCAGACCGGGCACCAGTGGCACGACAGTGGACACAGGGATAACAACCGGGGAAAAAAAATTCTCCGGCA
Baihulu     CCAGACCGGGCACCAGTGGCACGACAGTGGACACAGGGATAACAACCGGGGAAAAAAAATTCTCCGGCA
Chiyacao    CCAGACCGGGCACCAGTGGCACGACAGTGGACACAGGGATAACAACCGGGGAAAAAAAATTCTCCGGCA
Hongmangmai CCAGACCGGGCACCAGTGGCACGACAGTGGACACAGGGATAACAACCGGGGAAAAAAAATTCTCCGGCA
            8,560     8,570     8,580     8,590     8,600     8,610     8,620
Hulutou     AGCGGGGCGGCGGCGGTGGCAGCTCATGTTGTCCGGCGAGCCCCGAATGGCACGCGTCGGGAGGGGCTG
Baihulu     AGCGGGGCGGCGGCGGTGGCAGCTCATGTTGTCCGGCGAGCCCCGAATGGCACGCGTCGGGAGGGGCTG
Chiyacao    AGCGGGGCGGCGGCGGTGGCAGCTCATGTTGTCCGGCGAGCCCCGAATGGCACGCGTCGGGAGGGGCTG
Hongmangmai AGCGGGGCGGCGGCGGTGGCAGCTCATGTTGTCCGGCGAGCCCCGAATGGCACGCGTCGGGAGGGGCTG
            8,630     8,640     8,650     8,660     8,670     8,680     8,690
Hulutou     GTGGGTGGGTCGCCCACTCATGTTGTCCGGTGATTTTTCCAGCTGTGCCAGCTAAGTTTTTCAAGTGTT
Baihulu     GTGGGTGGGTCGCCCACTCATGTTGTCCGGTGATTTTTCCAGCTGTGCCAGCTAAGTTTTTCAAGTGTT
Chiyacao    GTGGGTGGGTCGCCCACTCATGTTGTCCGGTGATTTTTCCAGCTGTGCCAGCTAAGTTTTTCAAGTGTT
Hongmangmai GTGGGTGGGTCGCCCACTCATGTTGTCCGGTGATTTTTCCAGCTGTGCCAGCTAAGTTTTTCAAGTGTT
            8,700     8,710     8,720     8,730     8,740     8,750     8,760
Hulutou     TGCGATTTAGGAATTTTGAGAGGATACATGCTTCGCTGTTTGATCTATTTCTATCTATCTCATGTAAGC
Baihulu     TGCGATTTAGGAATTTTGAGAGGATACATGCTTCGCTGTTTGATCTATTTCTATCTATCTCATGTAAGC
Chiyacao    TGCGATTTAGGAATTTTGAGAGGATACATGCTTCGCTGTTTGATCTATTTCTATCTATCTCATGTAAGC
Hongmangmai TGCGATTTAGGAATTTTGAGAGGATACATGCTTCGCTGTTTGATCTATTTCTATCTATCTCATGTAAGC
            8,770     8,780     8,790     8,800     8,810     8,820     8,830
Hulutou     CTTTCGAGATGACAAGCAGATCAACTTCATGACATTATCAATACTTCTGCAGGTGACTATGGTGAACTT
Baihulu     CTTTCGAGATGACAAGCAGATCAACTTCATGACATTATCAATACTTCTGCAGGTGACTATGGTGAACTT
Chiyacao    CTTTCGAGATGACAAGCAGATCAACTTCATGACATTATCAATACTTCTGCAGGTGACTATGGTGAACTT
Hongmangmai CTTTCGAGATGACAAGCAGATCAACTTCATGACATTATCAATACTTCTGCAGGTGACTATGGTGAACTT
            8,840     8,850     8,860     8,870     8,880     8,890     8,900
Hulutou     GACTGGAACCAGCGTTATCAAATTCTAAAAGGAATTTGTCAAGGTTTGCATCATCTCCATGACGAAATG
Baihulu     GACTGGAACCAGCGTTATCAAATTCTAAAAGGAATTTGTCAAGGTTTGCATCATCTCCATGACGAAATG
Chiyacao    GACTGGAACCAGCGTTATCAAATTCTAAAAGGAATTTGTCAAGGTTTGCATCATCTCCATGACGAAATG
Hongmangmai GACTGGAACCAGCGTTATCAAATTCTAAAAGGAATTTGTCAAGGTTTGCATCATCTCCATGACGAAATG
            8,910     8,920     8,930     8,940     8,950     8,960     8,970
Hulutou     CACGTTTTTCATGGAGATATCAAACCAGCCAATATATTAATAGGGGATAACCTTGTGCCTAAAATCTAT
Baihulu     CACGTTTTTCATGGAGATATCAAACCAGCCAATATATTAATAGGGGATAACCTTGTGCCTAAAATCTAT
Chiyacao    CACGTTTTTCATGGAGATATCAAACCAGCCAATATATTAATAGGGGATAACCTTGTGCCTAAAATCTAT
Hongmangmai CACGTTTTTCATGGAGATATCAAACCAGCCAATATATTAATAGGGGATAACCTTGTGCCTAAAATCTAT
```

8,980 8,990 9,000 9,010 9,020 9,030

Hulutou GACTTCGGTCTCTCCCAGATGTTTGAAGAAGAAGAAACGGAACGTATTGTTGAAAATATCGCCGGAACA
Baihulu GACTTCGGTCTCTCCCAGATGTTTGAAGAAGAAGAAACGGAACGTATTGTTGAAAATATCGCCGGAACA
Chiyacao GACTTCGGTCTCTCCCAGATGTTTGAAGAAGAAGAAACGGAACGTATTGTTGAAAATATCGCCGGAACA
Hongmangmai GACTTCGGTCTCTCCCAGATGTTTGAAGAAGAAGAAACGGAACGTATTGTTGAAAATATCGCCGGAACA

9,040 9,050 9,060 9,070 9,080 9,090 9,100

Hulutou TTGTAAGCTAACCCGTTCCTCTTTGTTATTTTGTTTTGTTTCTGCTGACACAGGTACTTCGATAACCAA
Baihulu TTGTAAGCTAACCCGTTCCTCTTTGTTATTTTGTTTTGTTTCTGCTGACACAGGTACTTCGATAACCAA
Chiyacao TTGTAAGCTAACCCGTTCCTCTTTGTTATTTTGTTTTGTTTCTGCTGACACAGGTACTTCGATAACCAA
Hongmangmai TTGTAAGCTAACCCGTTCCTCTTTGTTATTTTGTTTTGTTTCTGCTGACACAGGTACTTCGATAACCAA

9,110 9,120 9,130 9,140 9,150 9,160 9,170

Hulutou AATGCATTAGAGTTAGTCGGCCGATTGCCTCACATCTAACATATGTGCAGCGGATATATGGCACCGGAG
Baihulu AATGCATTAGAGTTAGTCGGCCGATTGCCTCACATCTAACATATGTGCAGCGGATATATGGCACCGGAG
Chiyacao AATGCATTAGAGTTAGTCGGCCGATTGCCTCACATCTAACATATGTGCAGCGGATATATGGCACCGGAG
Hongmangmai AATGCATTAGAGTTAGTCGGCCGATTGCCTCACATCTAACATATGTGCAGCGGATATATGGCACCGGAG

9,180 9,190 9,200 9,210 9,220 9,230 9,240

Hulutou TTTTGTACTAATAATATGGTGTCATTTAAGGCTGAGATATACAGTTTGGGCGTTGTGATCGGGGAGTTA
Baihulu TTTTGTACTAATAATATGGTGTCATTTAAGGCTGAGATATACAGTTTGGGCGTTGTGATCGGGGAGTTA
Chiyacao TTTTGTACTAATAATATGGTGTCATTTAAGGCTGAGATATACAGTTTGGGCGTTGTGATCGGGGAGTTA
Hongmangmai TTTTGTACTAATAATATGGTGTCATTTAAGGCTGAGATATACAGTTTGGGCGTTGTGATCGGGGAGTTA

9,250 9,260 9,270 9,280 9,290 9,300 9,310

Hulutou TTGATCGGGAAGTGGTTTGATGAGGATGTGAGAAAACTATTTGTACAGCAACTTAAGGGTTTGAGAAAA
Baihulu TTGATCGGGAAGTGGTTTGATGAGGATGTGAGAAAACTATTTGTACAGCAACTTAAGGGTTTGAGAAAA
Chiyacao TTGATCGGGAAGTGGTTTGATGAGGATGTGAGAAAACTATTTGTACAGCAACTTAAGGGTTTGAGAAAA
Hongmangmai TTGATCGGGAAGTGGTTTGATGAGGATGTGAGAAAACTATTTGTACAGCAACTTAAGGGTTTGAGAAAA

9,320 9,330 9,340 9,350 9,360 9,370 9,380

Hulutou ACATTGGTAAAAGAAGGAGCGTTTTCATCATGGGAAAACAAATACCACCAAGTTAGAACATGTATGGAG
Baihulu ACATTGGTAAAAGAAGGAGCGTTTTCATCATGGGAAAACAAATACCACCAAGTTAGAACATGTATGGAG
Chiyacao ACATTGGTAAAAGAAGGAGCGTTTTCATCATGGGAAAACAAATACCACCAAGTTAGAACATGTATGGAG
Hongmangmai ACATTGGTAAAAGAAGGAGCGTTTTCATCATGGGAAAACAAATACCACCAAGTTAGAACATGTATGGAG

9,390 9,400 9,410 9,420 9,430 9,440 9,450

Hulutou ATTGGGCAGGACTGCATAGACCCCAACCCACATAAAAGGCCCACTTTGTTGGAGATTATCCAGCGGCTT
Baihulu ATTGGGCAGGACTGCATAGACCCCAACCCACATAAAAGGCCCACTTTGTTGGAGATTATCCAGCGGCTT
Chiyacao ATTGGGCAGGACTGCATAGACCCCAACCCACATAAAAGGCCCACTTTGTTGGAGATTATCCAGCGGCTT
Hongmangmai ATTGGGCAGGACTGCATAGACCCCAACCCACATAAAAGGCCCACTTTGTTGGAGATTATCCAGCGGCTT

9,460 9,470 9,480 9,490 9,500 9,510 9,520

Hulutou AATGAAGCGGAAGATATGAACTATTCTGCAGCATCACTTTGGCAGGTATGGAAAATCGAAAGCATAAGG
Baihulu AATGAAGCGGAAGATATGAACTATTCTGCAGCATCACTTTGGCAGGTATGGAAAATCGAAAGCATAAGG
Chiyacao AATGAAGCGGAAGATATGAACTATTCTGCAGCATCACTTTGGCAGGTATGGAAAATCGAAAGCATAAGG
Hongmangmai AATGAAGCGGAAGATATGAACTATTCTGCAGCATCACTTTGGCAGGTATGGAAAATCGAAAGCATAAGG

9,530 9,540 9,550 9,560 9,570 9,580 9,590

Hulutou AAACAACTGTTCTTTATCTACCATTTCACTTTAGCTAACGTACTGTTTGAGAAGAATCAGTCTTAGCCA
Baihulu AAACAACTGTTCTTTATCTACCATTTCACTTTAGCTAACGTACTGTTTGAGAAGAATCAGTCTTAGCCA
Chiyacao AAACAACTGTTCTTTATCTACCATTTCACTTTAGCTAACGTACTGTTTGAGAAGAATCAGTCTTAGCCA
Hongmangmai AAACAACTGTTCTTTATCTACCATTTCACTTTAGCTAACGTACTGTTTGAGAAGAATCAGTCTTAGCCA

9,600 9,610 9,620 9,630 9,640 9,650 9,660

Hulutou AGCTTACATCCTCACAGACACAGTACAGAAGTACATTTCTTTTCTTTAGCCATTACGATTAACATAAAG
Baihulu AGCTTACATCCTCACAGACACAGTACAGAAGTACATTTCTTTTCTTTAGCCATTACGATTAACATAAAG
Chiyacao AGCTTACATCCTCACAGACACAGTACAGAAGTACATTTCTTTTCTTTAGCCATTACGATTAACATAAAG
Hongmangmai AGCTTACATCCTCACAGACACAGTACAGAAGTACATTTCTTTTCTTTAGCCATTACGATTAACATAAAG

```
                    9,670       9,680       9,690       9,700       9,710       9,720
Hulutou       AGTTCACAAAGCCATCTCCAACGAAAGATATGTGCTAATTAAACTAAATTGGAAGAGAAAATACATAAC
Baihulu       AGTTCACAAAGCCATCTCCAACGAAAGATATGTGCTAATTAAACTAAATTGGAAGAGAAAATACATAAC
Chiyacao      AGTTCACAAAGCCATCTCCAACGAAAGATATGTGCTAATTAAACTAAATTGGAAGAGAAAATACATAAC
Hongmangmai   AGTTCACAAAGCCATCTCCAACGAAAGATATGTGCTAATTAAACTAAATTGGAAGAGAAAATACATAAC
              9,730       9,740       9,750       9,760       9,770       9,780       9,790
Hulutou       AAAGCGAAGACACCATGTAAATTAGAGAAGAAACAAACTCTTGGTACTCCTTCAGCGAGGGAAGACACC
Baihulu       AAAGCGAAGACACCATGTAAATTAGAGAAGAAACAAACTCTTGGTACTCCTTCAGCGAGGGAAGACACC
Chiyacao      AAAGCGAAGACACCATGTAAATTAGAGAAGAAACAAACTCTTGGTACTCCTTCAGCGAGGGAAGACACC
Hongmangmai   AAAGCGAAGACACCATGTAAATTAGAGAAGAAACAAACTCTTGGTACTCCTTCAGCGAGGGAAGACACC
              9,800       9,810       9,820       9,830       9,840       9,850       9,860
Hulutou       TTGTCAACTTAGCAGAAACAAACTCTTGGTACTCCTTCAGCGAGGGAAGACACCTTGTCAACTTAGCAG
Baihulu       TTGTCAACTTAGCAGAAACAAACTCTTGGTACTCCTTCAGCGAGGGAAGACACCTTGTCAACTTAGCAG
Chiyacao      TTGTCAACTTAGCAGAAACAAACTCTTGGTACTCCTTCAGCGAGGGAAGACACCTTGTCAACTTAGCAG
Hongmangmai   TTGTCAACTTAGCAGAAACAAACTCTTGGTACTCCTTCAGCGAGGGAAGACACCTTGTCAACTTAGCAG
               9,870       9,880       9,890       9,900       9,910       9,920       9,930
Hulutou       AAACCTTACGACTCTAGTCCTACATGACATGTCTAAGAGAATATTGATGTCATGCATTGCATAATGCTT
Baihulu       AAACCTTACGACTCTAGTCCTACATGACATGTCTAAGAGAATATTGATGTCATGCATTGCATAATGCTT
Chiyacao      AAACCTTACGACTCTAGTCCTACATGACATGTCTAAGAGAATATTGATGTCATGCATTGCATAATGCTT
Hongmangmai   AAACCTTACGACTCTAGTCCTACATGACATGTCTAAGAGAATATTGATGTCATGCATTGCATAATGCTT
                9,940       9,950       9,960       9,970       9,980       9,990       10,000
Hulutou       ACATGGTAAGTTATATATAAGTGCAGTCAGGAGACGAGGAATCCGATTTATCGGATACAGAAGCTTTGG
Baihulu       ACATGGTAAGTTATATATAAGTGCAGTCAGGAGACGAGGAATCCGATTTATCGGATACAGAAGCTTTGG
Chiyacao      ACATGGTAAGTTATATATAAGTGCAGTCAGGAGACGAGGAATCCGATTTATCGGATACAGAAGCTTTGG
Hongmangmai   ACATGGTAAGTTATATATAAGTGCAGTCAGGAGACGAGGAATCCGATTTATCGGATACAGAAGCTTTGG
                 10,010      10,020      10,030      10,040      10,050      10,060      10,070
Hulutou       AGACAGAGACAACATCCGAGTTTCTTCCAAGTGACGAAGAACCCGCCTCTGTGGGCAAGACCGGAGAAA
Baihulu       AGACAGAGACAACATCCGAGTTTCTTCCAAGTGACGAAGAACCCGCCTCTGTGGGCAAGACCGGAGAAA
Chiyacao      AGACAGAGACAACATCCGAGTTTCTTCCAAGTGACGAAGAACCCGCCTCTGTGGGCAAGACCGGAGAAA
Hongmangmai   AGACAGAGACAACATCCGAGTTTCTTCCAAGTGACGAAGAACCCGCCTCTGTGGGCAAGACCGGAGAAA
                  10,080      10,090      10,100      10,110      10,120      10,130      10,140
Hulutou       CAAGCACACAGGAGCCTGATAAACCGGACCTAATAAGTAAGTTGCCAGCATCGGTGGACCTGTCTGACC
Baihulu       CAAGCACACAGGAGCCTGATAAACCGGACCTAATAAGTAAGTTGCCAGCATCGGTGGACCTGTCTGACC
Chiyacao      CAAGCACACAGGAGCCTGATAAACCGGACCTAATAAGTAAGTTGCCAGCATCGGTGGACCTGTCTGACC
Hongmangmai   CAAGCACACAGGAGCCTGATAAACCGGACCTAATAAGTAAGTTGCCAGCATCGGTGGACCTGTCTGACC
                   10,150      10,160      10,170      10,180      10,190      10,200      10,210
Hulutou       TAAAAGTCCTGGAGAAAATCACAGATGATTTTTCACACGAAAGAATAGTTGGGAAGGACGGTACATTCA
Baihulu       TAAAAGTCCTGGAGAAAATCACAGATGATTTTTCACACGAAAGAATAGTTGGGAAGGACGGTACATTCA
Chiyacao      TAAAAGTCCTGGAGAAAATCACAGATGATTTTTCACACGAAAGAATAGTTGGGAAGGACGGTACATTCA
Hongmangmai   TAAAAGTCCTGGAGAAAATCACAGATGATTTTTCACACGAAAGAATAGTTGGGAAGGACGGTACATTCA
                    10,220      10,230      10,240      10,250      10,260      10,270      10,280
Hulutou       AAGGTTGTCATAAGGCATTTGTTTATAAGGTACACATAGTTGTAAGTTACTATGCAGTGCTTTGCTTTG
Baihulu       AAGGTTGTCATAAGGCATTTGTTTATAAGGTACACATAGTTGTAAGTTACTATGCAGTGCTTTGCTTTG
Chiyacao      AAGGTTGTCATAAGGCATTTGTTTATAAGGTACACATAGTTGTAAGTTACTATGCAGTGCTTTGCTTTG
Hongmangmai   AAGGTTGTCATAAGGCATTTGTTTATAAGGTACACATAGTTGTAAGTTACTATGCAGTGCTTTGCTTTG
                     10,290      10,300      10,310      10,320      10,330      10,340    10,350
Hulutou       CTGATGTCCTTTGAGTTCAACATATTCTTCCATCTTAATTATACTCCTAACACACACACGCATACTAAA
Baihulu       CTGATGTCCTTTGAGTTCAACATATTCTTCCATCTTAATTATACTCCTAACACACACACGCATACTAAA
Chiyacao      CTGATGTCCTTTGAGTTCAACATATTCTTCCATCTTAATTATACTCCTAACACACACACGCATACTAAA
Hongmangmai   CTGATGTCCTTTGAGTTCAACATATTCTTCCATCTTAATTATACTCCTAACACACACACGCATACTAAA
```

```
              10,360    10,370    10,380    10,390    10,400    10,410
Hulutou       TGTACATTTATTTGAACAGGGTGACATTCCACTTAGAGAAATGATAGCCGTGAAGAGGTTAATTGGAGT
Baihulu       TGTACATTTATTTGAACAGGGTGACATTCCACTTAGAGAAATGATAGCCGTGAAGAGGTTAATTGGAGT
Chiyacao      TGTACATTTATTTGAACAGGGTGACATTCCACTTAGAGAAATGATAGCCGTGAAGAGGTTAATTGGAGT
Hongmangmai   TGTACATTTATTTGAACAGGGTGACATTCCACTTAGAGAAATGATAGCCGTGAAGAGGTTAATTGGAGT
              10,420    10,430    10,440    10,450    10,460    10,470    10,480
Hulutou       GGAGATTCCATTTGAAAAGTTTAAGAGGGAAGCAGAACAGTTCATTAGTCTCGATCATAAGAATATAGT
Baihulu       GGAGATTCCATTTGAAAAGTTTAAGAGGGAAGCAGAACAGTTCATTAGTCTCGATCATAAGAATATAGT
Chiyacao      GGAGATTCCATTTGAAAAGTTTAAGAGGGAAGCAGAACAGTTCATTAGTCTCGATCATAAGAATATAGT
Hongmangmai   GGAGATTCCATTTGAAAAGTTTAAGAGGGAAGCAGAACAGTTCATTAGTCTCGATCATAAGAATATAGT
              10,490    10,500    10,510    10,520    10,530    10,540    10,550
Hulutou       AAAGGTTGCCAGCTACTGCCACGACCAGTCTAGAGGACATAGACTGGTACAGTTCAAAGGAAAACCGCT
Baihulu       AAAGGTTGCCAGCTACTGCCACGACCAGTCTAGAGGACATAGACTGGTACAGTTCAAAGGAAAACCGCT
Chiyacao      AAAGGTTGCCAGCTACTGCCACGACCAGTCTAGAGGACATAGACTGGTACAGTTCAAAGGAAAACCGCT
Hongmangmai   AAAGGTTGCCAGCTACTGCCACGACCAGTCTAGAGGACATAGACTGGTACAGTTCAAAGGAAAACCGCT
              10,560    10,570    10,580    10,590    10,600    10,610    10,620
Hulutou       ACCACAACTCTTTAACGGTCCCGAACAACTGCTCTGCTATGAATATATGCACAACGGAAGCCTTCGCGA
Baihulu       ACCACAACTCTTTAACGGTCCCGAACAACTGCTCTGCTATGAATATATGCACAACGGAAGCCTTCGCGA
Chiyacao      ACCACAACTCTTTAACGGTCCCGAACAACTGCTCTGCTATGAATATATGCACAACGGAAGCCTTCGCGA
Hongmangmai   ACCACAACTCTTTAACGGTCCCGAACAACTGCTCTGCTATGAATATATGCACAACGGAAGCCTTCGCGA
              10,630    10,640    10,650    10,660    10,670    10,680    10,690
Hulutou       CTATCTTATGGGTAATGATACCGATCACTAATCAACTACTGTTTTCTTTAACATCCGTAAACAATTACT
Baihulu       CTATCTTATGGGTAATGATACCGATCACTAATCAACTACTGTTTTCTTTAACATCCGTAAACAATTACT
Chiyacao      CTATCTTATGGGTAATGATACCGATCACTAATCAACTACTGTTTTCTTTAACATCCGTAAACAATTACT
Hongmangmai   CTATCTTATGGGTAATGATACCGATCACTAATCAACTACTGTTTTCTTTAACATCCGTAAACAATTACT
              10,700    10,710    10,720    10,730    10,740    10,750    10,760
Hulutou       GTTTTCTTTAACATCTGTAATCAACTAGTGACAATTGCTTTCGATGGAAGAGAGCTGCACATCCAGTAC
Baihulu       GTTTTCTTTAACATCTGTAATCAACTAGTGACAATTGCTTTCGATGGAAGAGAGCTGCACATCCAGTAC
Chiyacao      GTTTTCTTTAACATCTGTAATCAACTAGTGACAATTGCTTTCGATGGAAGAGAGCTGCACATCCAGTAC
Hongmangmai   GTTTTCTTTAACATCTGTAATCAACTAGTGACAATTGCTTTCGATGGAAGAGAGCTGCACATCCAGTAC
              10,770    10,780    10,790    10,800    10,810    10,820    10,830
Hulutou       AAATCATATATATGGATGCAAATATTTAGGAACTGCTTGCAATGGAAGCCAGGTTAGAAGAACCCTAGC
Baihulu       AAATCATATATATGGATGCAAATATTTAGGAACTGCTTGCAATGGAAGCCAGGTTAGAAGAACCCTAGC
Chiyacao      AAATCATATATATGGATGCAAATATTTAGGAACTGCTTGCAATGGAAGCCAGGTTAGAAGAACCCTAGC
Hongmangmai   AAATCATATATATGGATGCAAATATTTAGGAACTGCTTGCAATGGAAGCCAGGTTAGAAGAACCCTAGC
              10,840    10,850    10,860    10,870    10,880    10,890    10,900
Hulutou       GCTTGGAAGGCAGCATCAAACCTCTCCCCTCCCCTGGGTCGCCCCCGCGCGGCGACCAGGGGGAACCCT
Baihulu       GCTTGGAAGGCAGCATCAAACCTCTCCCCTCCCCTGGGTCGCCCCCGCGCGGCGACCAGGGGGAACCCT
Chiyacao      GCTTGGAAGGCAGCATCAAACCTCTCCCCTCCCCTGGGTCGCCCCCGCGCGGCGACCAGGGGGAACCCT
Hongmangmai   GCTTGGAAGGCAGCATCAAACCTCTCCCCTCCCCTGGGTCGCCCCCGCGCGGCGACCAGGGGGAACCCT
              10,910    10,920    10,930    10,940    10,950    10,960    10,970
Hulutou       AGCGCCGCCAAGCCCTCGGCTCCCTCTCCCACCTCTCCTGCCCGCCGCCGCCGGGGCGCGCCGTCGGGC
Baihulu       AGCGCCGCCAAGCCCTCGGCTCCCTCTCCCACCTCTCCTGCCCGCCGCCGCCGGGGCGCGCCGTCGGGC
Chiyacao      AGCGCCGCCAAGCCCTCGGCTCCCTCTCCCACCTCTCCTGCCCGCCGCCGCCGGGGCGCGCCGTCGGGC
Hongmangmai   AGCGCCGCCAAGCCCTCGGCTCCCTCTCCCACCTCTCCTGCCCGCCGCCGCCGGGGCGCGCCGTCGGGC
              10,980    10,990    11,000    11,010    11,020    11,030    11,040
Hulutou       AAAGCCCGGTTGGCGTCGGCGGCGGCGGGATCTCTCCTCCCCGCAGCGGCCGGACCTGGCGCGGGTGGC
Baihulu       AAAGCCCGGTTGGCGTCGGCGGCGGCGGGATCTCTCCTCCCCGCAGCGGCCGGACCTGGCGCGGGTGGC
Chiyacao      AAAGCCCGGTTGGCGTCGGCGGCGGCGGGATCTCTCCTCCCCGCAGCGGCCGGACCTGGCGCGGGTGGC
Hongmangmai   AAAGCCCGGTTGGCGTCGGCGGCGGCGGGATCTCTCCTCCCCGCAGCGGCCGGACCTGGCGCGGGTGGC
```

```
            11,050      11,060      11,070      11,080      11,090      11,100
Hulutou     GTTCGGCGGCGGTTGGCGGCGCGTTGGTGACGGGGCGCGCCGGCGAGGGCTCAGGGGGTGACGGCAGGA
Baihulu     GTTCGGCGGCGGTTGGCGGCGCGTTGGTGACGGGGCGCGCCGGCGAGGGCTCAGGGGGTGACGGCAGGA
Chiyacao    GTTCGGCGGCGGTTGGCGGCGCGTTGGTGACGGGGCGCGCCGGCGAGGGCTCAGGGGGTGACGGCAGGA
Hongmangmai GTTCGGCGGCGGTTGGCGGCGCGTTGGTGACGGGGCGCGCCGGCGAGGGCTCAGGGGGTGACGGCAGGA
            11,110      11,120      11,130      11,140      11,150      11,160      11,170
Hulutou     GCTGCTTCCCCCGGTGGTCGCGTGGGGGGCCATTCGGGGCGCGCCTATGGTGGCCATGGTCGATCTGTT
Baihulu     GCTGCTTCCCCCGGTGGTCGCGTGGGGGGCCATTCGGGGCGCGCCTATGGTGGCCATGGTCGATCTGTT
Chiyacao    GCTGCTTCCCCCGGTGGTCGCGTGGGGGGCCATTCGGGGCGCGCCTATGGTGGCCATGGTCGATCTGTT
Hongmangmai GCTGCTTCCCCCGGTGGTCGCGTGGGGGGCCATTCGGGGCGCGCCTATGGTGGCCATGGTCGATCTGTT
            11,180      11,190      11,200      11,210      11,220      11,230      11,240
Hulutou     TTAGATCTGACCGCCGGTGCCTCGGGCGGCGCGGGGATCTCAGGGCGGCGACCTCGATCGATGGCGCGC
Baihulu     TTAGATCTGACCGCCGGTGCCTCGGGCGGCGCGGGGATCTCAGGGCGGCGACCTCGATCGATGGCGCGC
Chiyacao    TTAGATCTGACCGCCGGTGCCTCGGGCGGCGCGGGGATCTCAGGGCGGCGACCTCGATCGATGGCGCGC
Hongmangmai TTAGATCTGACCGCCGGTGCCTCGGGCGGCGCGGGGATCTCAGGGCGGCGACCTCGATCGATGGCGCGC
            11,250      11,260      11,270      11,280      11,290      11,300      11,310
Hulutou     CGTGCGGCCTGCCTGGCGGGCGGCACCCGCGGGTGCAGATGGGGGGCCTTCCACGGCTGTGTGGGCGGT
Baihulu     CGTGCGGCCTGCCTGGCGGGCGGCACCCGCGGGTGCAGATGGGGGGCCTTCCACGGCTGTGTGGGCGGT
Chiyacao    CGTGCGGCCTGCCTGGCGGGCGGCACCCGCGGGTGCAGATGGGGGGCCTTCCACGGCTGTGTGGGCGGT
Hongmangmai CGTGCGGCCTGCCTGGCGGGCGGCACCCGCGGGTGCAGATGGGGGGCCTTCCACGGCTGTGTGGGCGGT
            11,320      11,330      11,340      11,350      11,360      11,370      11,380
Hulutou     GTGGAGGTGGCGGCGCTACGACGGCTCCTCGACGGCCGCCCGGAGTCCCATGGGAGGTGTCGTCTCAGA
Baihulu     GTGGAGGTGGCGGCGCTACGACGGCTCCTCGACGGCCGCCCGGAGTCCCATGGGAGGTGTCGTCTCAGA
Chiyacao    GTGGAGGTGGCGGCGCTACGACGGCTCCTCGACGGCCGCCCGGAGTCCCATGGGAGGTGTCGTCTCAGA
Hongmangmai GTGGAGGTGGCGGCGCTACGACGGCTCCTCGACGGCCGCCCGGAGTCCCATGGGAGGTGTCGTCTCAGA
            11,390      11,400      11,410      11,420      11,430      11,440      11,450
Hulutou     CTCGATCTACTCCGGTTCGTGCTGGCTACAGTAGCGACTACGGTCGACACCAATACTGCCTGGACGGAT
Baihulu     CTCGATCTACTCCGGTTCGTGCTGGCTACAGTAGCGACTACGGTCGACACCAATACTGCCTGGACGGAT
Chiyacao    CTCGATCTACTCCGGTTCGTGCTGGCTACAGTAGCGACTACGGTCGACACCAATACTGCCTGGACGGAT
Hongmangmai CTCGATCTACTCCGGTTCGTGCTGGCTACAGTAGCGACTACGGTCGACACCAATACTGCCTGGACGGAT
            11,460      11,470      11,480      11,490      11,500      11,510      11,520
Hulutou     CTGGCGGGTGGGCATGGATCCGGGGGAAACTCCAGGCCGGCGTGGCGGCCGCACCAAAGTCAACGCCCT
Baihulu     CTGGCGGGTGGGCATGGATCCGGGGGAAACTCCAGGCCGGCGTGGCGGCCGCACCAAAGTCAACGCCCT
Chiyacao    CTGGCGGGTGGGCATGGATCCGGGGGAAACTCCAGGCCGGCGTGGCGGCCGCACCAAAGTCAACGCCCT
Hongmangmai CTGGCGGGTGGGCATGGATCCGGGGGAAACTCCAGGCCGGCGTGGCGGCCGCACCAAAGTCAACGCCCT
            11,530      11,540      11,550      11,560      11,570      11,580      11,590
Hulutou     TGGCGCCGATTACCTTCCTGGAGGCTTCGGTGTGGATCATACGCCCCCCACCTCTACCATGAGCGCAAG
Baihulu     TGGCGCCGATTACCTTCCTGGAGGCTTCGGTGTGGATCATACGCCCCCCACCTCTACCATGAGCGCAAG
Chiyacao    TGGCGCCGATTACCTTCCTGGAGGCTTCGGTGTGGATCATACGCCCCCCACCTCTACCATGAGCGCAAG
Hongmangmai TGGCGCCGATTACCTTCCTGGAGGCTTCGGTGTGGATCATACGCCCCCCACCTCTACCATGAGCGCAAG
            11,600      11,610      11,620      11,630      11,640      11,650      11,660
Hulutou     CGAAAGCCTCTGTTCCTCTGTTTGGGCGACGATGACACTTTGGTGTCATGTTCCTTCCTGAAGGCATCG
Baihulu     CGAAAGCCTCTGTTCCTCTGTTTGGGCGACGATGACACTTTGGTGTCATGTTCCTTCCTGAAGGCATCG
Chiyacao    CGAAAGCCTCTGTTCCTCTGTTTGGGCGACGATGACACTTTGGTGTCATGTTCCTTCCTGAAGGCATCG
Hongmangmai CGAAAGCCTCTGTTCCTCTGTTTGGGCGACGATGACACTTTGGTGTCATGTTCCTTCCTGAAGGCATCG
            11,670      11,680      11,690      11,700      11,710      11,720      11,730
Hulutou     GCCGAGGACCGCTCAGGGTGGTGGAGTTGCTGGTAGTTCGGAGTCGACACGAGATGGCATGTAGGTGGA
Baihulu     GCCGAGGACCGCTCAGGGTGGTGGAGTTGCTGGTAGTTCGGAGTCGACACGAGATGGCATGTAGGTGGA
Chiyacao    GCCGAGGACCGCTCAGGGTGGTGGAGTTGCTGGTAGTTCGGAGTCGACACGAGATGGCATGTAGGTGGA
Hongmangmai GCCGAGGACCGCTCAGGGTGGTGGAGTTGCTGGTAGTTCGGAGTCGACACGAGATGGCATGTAGGTGGA
```

```
             11,740      11,750      11,760      11,770      11,780      11,790
Hulutou      GCAGTGTGCCATCAACAGTATCATCGACGGTGGGTCTCGGCGGCATGGCGCAGTGGAGTCTCGGCGTCC
Baihulu      GCAGTGTGCCATCAACAGTATCATCGACGGTGGGTCTCGGCGGCATGGCGCAGTGGAGTCTCGGCGTCC
Chiyacao     GCAGTGTGCCATCAACAGTATCATCGACGGTGGGTCTCGGCGGCATGGCGCAGTGGAGTCTCGGCGTCC
Hongmangmai  GCAGTGTGCCATCAACAGTATCATCGACGGTGGGTCTCGGCGGCATGGCGCAGTGGAGTCTCGGCGTCC

             11,800      11,810      11,820      11,830      11,840      11,850      11,860
Hulutou      GATGCGCGGAGATGGACTCGCGCAGGAGGAGGTAGCTGTCTGGCGTCGTGGTGGCGTCGACGGTAGCTA
Baihulu      GATGCGCGGAGATGGACTCGCGCAGGAGGAGGTAGCTGTCTGGCGTCGTGGTGGCGTCGACGGTAGCTA
Chiyacao     GATGCGCGGAGATGGACTCGCGCAGGAGGAGGTAGCTGTCTGGCGTCGTGGTGGCGTCGACGGTAGCTA
Hongmangmai  GATGCGCGGAGATGGACTCGCGCAGGAGGAGGTAGCTGTCTGGCGTCGTGGTGGCGTCGACGGTAGCTA

             11,870      11,880      11,890      11,900      11,910      11,920      11,930
Hulutou      GACCGTGCAAGGGAGATGCAACAGTACAACTCTGAAGATGGATTGGTGGCAGGTGGCTGCGGCAGCCTC
Baihulu      GACCGTGCAAGGGAGATGCAACAGTACAACTCTGAAGATGGATTGGTGGCAGGTGGCTGCGGCAGCCTC
Chiyacao     GACCGTGCAAGGGAGATGCAACAGTACAACTCTGAAGATGGATTGGTGGCAGGTGGCTGCGGCAGCCTC
Hongmangmai  GACCGTGCAAGGGAGATGCAACAGTACAACTCTGAAGATGGATTGGTGGCAGGTGGCTGCGGCAGCCTC

             11,940      11,950      11,960      11,970      11,980      11,990      12,000
Hulutou      ATACCCGGCAGGCGTCCTGGTTGAGGAGTGCGCTGGACTGGTAGCAGGTGCCCCATACCCGGCAGGCGT
Baihulu      ATACCCGGCAGGCGTCCTGGTTGAGGAGTGCGCTGGACTGGTAGCAGGTGCCCCATACCCGGCAGGCGT
Chiyacao     ATACCCGGCAGGCGTCCTGGTTGAGGAGTGCGCTGGACTGGTAGCAGGTGCCCCATACCCGGCAGGCGT
Hongmangmai  ATACCCGGCAGGCGTCCTGGTTGAGGAGTGCGCTGGACTGGTAGCAGGTGCCCCATACCCGGCAGGCGT

             12,010      12,020      12,030      12,040      12,050      12,060      12,070
Hulutou      CCTGGTTGAGGAGTGCGCTGGACTGGTAGCAGGTGCCCCATACCCGGCAGGCGTCCTGGTTGGGACCTC
Baihulu      CCTGGTTGAGGAGTGCGCTGGACTGGTAGCAGGTGCCCCATACCCGGCAGGCGTCCTGGTTGGGACCTC
Chiyacao     CCTGGTTGAGGAGTGCGCTGGACTGGTAGCAGGTGCCCCATACCCGGCAGGCGTCCTGGTTGGGACCTC
Hongmangmai  CCTGGTTGAGGAGTGCGCTGGACTGGTAGCAGGTGCCCCATACCCGGCAGGCGTCCTGGTTGGGACCTC

             12,080      12,090      12,100      12,110      12,120      12,130      12,140
Hulutou      AGGTCTTAGATGTTTAGGTTTGGCTGCGATGTCTGTTTGGTATTAGGCCCAGACTATCAGTGCCCCTTC
Baihulu      AGGTCTTAGATGTTTAGGTTTGGCTGCGATGTCTGTTTGGTATTAGGCCCAGACTATCAGTGCCCCTTC
Chiyacao     AGGTCTTAGATGTTTAGGTTTGGCTGCGATGTCTGTTTGGTATTAGGCCCAGACTATCAGTGCCCCTTC
Hongmangmai  AGGTCTTAGATGTTTAGGTTTGGCTGCGATGTCTGTTTGGTATTAGGCCCAGACTATCAGTGCCCCTTC

             12,150      12,160      12,170      12,180      12,190      12,200      12,210
Hulutou      ATCAATTGGATAGGTGTAGCGACAATTATTGCTTAGACGGTGGCTTTAGTCTTGCTGTTGTATGACTTT
Baihulu      ATCAATTGGATAGGTGTAGCGACAATTATTGCTTAGACGGTGGCTTTAGTCTTGCTGTTGTATGACTTT
Chiyacao     ATCAATTGGATAGGTGTAGCGACAATTATTGCTTAGACGGTGGCTTTAGTCTTGCTGTTGTATGACTTT
Hongmangmai  ATCAATTGGATAGGTGTAGCGACAATTATTGCTTAGACGGTGGCTTTAGTCTTGCTGTTGTATGACTTT

             12,220      12,230      12,240      12,250      12,260      12,270      12,280
Hulutou      GTAAGGTCTTATGAGAATAATTAATAAAGTGGCCGTATGCATCGCCCAGATACAGAGGCCGGGGGTCAT
Baihulu      GTAAGGTCTTATGAGAATAATTAATAAAGTGGCCGTATGCATCGCCCAGATACAGAGGCCGGGGGTCAT
Chiyacao     GTAAGGTCTTATGAGAATAATTAATAAAGTGGCCGTATGCATCGCCCAGATACAGAGGCCGGGGGTCAT
Hongmangmai  GTAAGGTCTTATGAGAATAATTAATAAAGTGGCCGTATGCATCGCCCAGATACAGAGGCCGGGGGTCAT

             12,290      12,300      12,310      12,320      12,330      12,340      12,350
Hulutou      CCTCCTTTTCTTAAAAAAAGGAACTGCTTGCAAAAATAAAAATAAAATTGAACAATTTAAAGTTCAATA
Baihulu      CCTCCTTTTCTTAAAAAAAGGAACTGCTTGCAAAAATAAAAATAAAATTGAACAATTTAAAGTTCAATA
Chiyacao     CCTCCTTTTCTTAAAAAAAGGAACTGCTTGCAAAAATAAAAATAAAATTGAACAATTTAAAGTTCAATA
Hongmangmai  CCTCCTTTTCTTAAAAAAAGGAACTGCTTGCAAAAATAAAAATAAAATTGAACAATTTAAAGTTCAATA

             12,360      12,370      12,380      12,390      12,400      12,410      12,420
Hulutou      AGCTAGCTCCTTGCTCATTGATCTCTCCATGTCTACTTGTTGTGTTACTAGGTCAAGGATCTCGTGTAA
Baihulu      AGCTAGCTCCTTGCTCATTGATCTCTCCATGTCTACTTGTTGTGTTACTAGGTCAAGGATCTCGTGTAA
Chiyacao     AGCTAGCTCCTTGCTCATTGATCTCTCCATGTCTACTTGTTGTGTTACTAGGTCAAGGATCTCGTGTAA
Hongmangmai  AGCTAGCTCCTTGCTCATTGATCTCTCCATGTCTACTTGTTGTGTTACTAGGTCAAGGATCTCGTGTAA
```

```
            12,430  12,440  12,450  12,460  12,470  12,480
Hulutou     TTGATTGGCAAATGCGCTACAAATTGATCAAAGGGACTTGCGCAGGCTTACATTACCTTCACAAGGGCC
Baihulu     TTGATTGGCAAATGCGCTACAAATTGATCAAAGGGACTTGCGCAGGCTTACATTACCTTCACAAGGGCC
Chiyacao    TTGATTGGCAAATGCGCTACAAATTGATCAAAGGGACTTGCGCAGGCTTACATTACCTTCACAAGGGCC
Hongmangmai TTGATTGGCAAATGCGCTACAAATTGATCAAAGGGACTTGCGCAGGCTTACATTACCTTCACAAGGGCC
            12,490  12,500  12,510  12,520  12,530  12,540  12,550
Hulutou     GTGCAGGTTGTCCAATTGTTCATTTGAATTTAAGCCCGTCAAATGTATTGCTGGACCACAACTACATAC
Baihulu     GTGCAGGTTGTCCAATTGTTCATTTGAATTTAAGCCCGTCAAATGTATTGCTGGACCACAACTACATAC
Chiyacao    GTGCAGGTTGTCCAATTGTTCATTTGAATTTAAGCCCGTCAAATGTATTGCTGGACCACAACTACATAC
Hongmangmai GTGCAGGTTGTCCAATTGTTCATTTGAATTTAAGCCCGTCAAATGTATTGCTGGACCACAACTACATAC
            12,560  12,570  12,580  12,590  12,600  12,610  12,620
Hulutou     CACGCATCACAGGGTTCGATTTTTCGAAGCTCATTGGTGAAAAGAACACCAAATCAGTGGTACTTAAGC
Baihulu     CACGCATCACAGGGTTCGATTTTTCGAAGCTCATTGGTGAAAAGAACACCAAATCAGTGGTACTTAAGC
Chiyacao    CACGCATCACAGGGTTCGATTTTTCGAAGCTCATTGGTGAAAAGAACACCAAATCAGTGGTACTTAAGC
Hongmangmai CACGCATCACAGGGTTCGATTTTTCGAAGCTCATTGGTGAAAAGAACACCAAATCAGTGGTACTTAAGC
            12,630  12,640  12,650  12,660  12,670  12,680  12,690
Hulutou     TGAATGGACCCATGTATGTATGCTCTCAGTGCTACTGGTTTAAAATTAGCTCTTGAATTTGCTCATGAT
Baihulu     TGAATGGACCCATGTATGTATGCTCTCAGTGCTACTGGTTTAAAATTAGCTCTTGAATTTGCTCATGAT
Chiyacao    TGAATGGACCCATGTATGTATGCTCTCAGTGCTACTGGTTTAAAATTAGCTCTTGAATTTGCTCATGAT
Hongmangmai TGAATGGACCCATGTATGTATGCTCTCAGTGCTACTGGTTTAAAATTAGCTCTTGAATTTGCTCATGAT
            12,700  12,710  12,720  12,730  12,740  12,750  12,760
Hulutou     ACATGTCTATGTTTTGGAAAAATGTCTTATGTTTTGTGGCAGATCGGTTTAGCTAGTCTTTTTTCTGTG
Baihulu     ACATGTCTATGTTTTGGAAAAATGTCTTATGTTTTGTGGCAGATCGGTTTAGCTAGTCTTTTTTCTGTG
Chiyacao    ACATGTCTATGTTTTGGAAAAATGTCTTATGTTTTGTGGCAGATCGGTTTAGCTAGTCTTTTTTCTGTG
Hongmangmai ACATGTCTATGTTTTGGAAAAATGTCTTATGTTTTGTGGCAGATCGGTTTAGCTAGTCTTTTTTCTGTG
            12,770  12,780  12,790  12,800  12,810  12,820  12,830
Hulutou     AGAAACGTTTAGCAAGTCTATTGACCGTAATATGCTTTAATTAAACTGATGTGTTCATATATTTTTGCA
Baihulu     AGAAACGTTTAGCAAGTCTATTGACCGTAATATGCTTTAATTAAACTGATGTGTTCATATATTTTTGCA
Chiyacao    AGAAACGTTTAGCAAGTCTATTGACCGTAATATGCTTTAATTAAACTGATGTGTTCATATATTTTTGCA
Hongmangmai AGAAACGTTTAGCAAGTCTATTGACCGTAATATGCTTTAATTAAACTGATGTGTTCATATATTTTTGCA
            12,840  12,850  12,860  12,870  12,880  12,890  12,900
Hulutou     GAGCGTACCTGCCACCGGATTTCTTCCATTCGAAGGGTACTGATCTTAAATATCTTGCTACGGTAGATA
Baihulu     GAGCGTACCTGCCACCGGATTTCTTCCATTCGAAGGGTACTGATCTTAAATATCTTGCTACGGTAGATA
Chiyacao    GAGCGTACCTGCCACCGGATTTCTTCCATTCGAAGGGTACTGATCTTAAATATCTTGCTACGGTAGATA
Hongmangmai GAGCGTACCTGCCACCGGATTTCTTCCATTCGAAGGGTACTGATCTTAAATATCTTGCTACGGTAGATA
            12,910  12,920  12,930  12,940  12,950  12,960  12,970
Hulutou     TATACAGCTTGGGTCTTATGATTTTAGAAATCGCAACACAACAAGAGATCAAAGGCATCCATGGAGTGC
Baihulu     TATACAGCTTGGGTCTTATGATTTTAGAAATCGCAACACAACAAGAGATCAAAGGCATCCATGGAGTGC
Chiyacao    TATACAGCTTGGGTCTTATGATTTTAGAAATCGCAACACAACAAGAGATCAAAGGCATCCATGGAGTGC
Hongmangmai TATACAGCTTGGGTCTTATGATTTTAGAAATCGCAACACAACAAGAGATCAAAGGCATCCATGGAGTGC
            12,980  12,990  13,000  13,010  13,020  13,030  13,040
Hulutou     TTATTAAGAGTGTAAGACAAATTTATTAACCCTCTGATCATGAGATAGCTTTTATTGCTTGACATAAAA
Baihulu     TTATTAAGAGTGTAAGACAAATTTATTAACCCTCTGATCATGAGATAGCTTTTATTGCTTGACATAAAA
Chiyacao    TTATTAAGAGTGTAAGACAAATTTATTAACCCTCTGATCATGAGATAGCTTTTATTGCTTGACATAAAA
Hongmangmai TTATTAAGAGTGTAAGACAAATTTATTAACCCTCTGATCATGAGATAGCTTTTATTGCTTGACATAAAA
            13,050  13,060  13,070  13,080  13,090  13,100  13,110
Hulutou     GAACAAAAAAATTCAACCTTTTAAAACTTTGATGATCAAATATACTCTACAGATAGAGGAAAACTGGAG
Baihulu     GAACAAAAAAATTCAACCTTTTAAAACTTTGATGATCAAATATACTCTACAGATAGAGGAAAACTGGAG
Chiyacao    GAACAAAAAAATTCAACCTTTTAAAACTTTGATGATCAAATATACTCTACAGATAGAGGAAAACTGGAG
Hongmangmai GAACAAAAAAATTCAACCTTTTAAAACTTTGATGATCAAATATACTCTACAGATAGAGGAAAACTGGAG
```

13,120 13,130 13,140 13,150 13,160 13,170

Hulutou GGAGGAGTCACAAATAACACGGCTGTATACCTCACTAGGGGCCGACGAGCTGCGGCAAGTAAAAATGTG
Baihulu GGAGGAGTCACAAATAACACGGCTGTATACCTCACTAGGGGCCGACGAGCTGCGGCAAGTAAAAATGTG
Chiyacao GGAGGAGTCACAAATAACACGGCTGTATACCTCACTAGGGGCCGACGAGCTGCGGCAAGTAAAAATGTG
Hongmangmai GGAGGAGTCACAAATAACACGGCTGTATACCTCACTAGGGGCCGACGAGCTGCGGCAAGTAAAAATGTG

13,180 13,190 13,200 13,210 13,220 13,230 13,240

Hulutou CATTGATATTGGCCTAGACTGTGTCAAGTCAAACCCTGAAAAGAGACCTACAGCTGGGGCCATCATGCT
Baihulu CATTGATATTGGCCTAGACTGTGTCAAGTCAAACCCTGAAAAGAGACCTACAGCTGGGGCCATCATGCT
Chiyacao CATTGATATTGGCCTAGACTGTGTCAAGTCAAACCCTGAAAAGAGACCTACAGCTGGGGCCATCATGCT
Hongmangmai CATTGATATTGGCCTAGACTGTGTCAAGTCAAACCCTGAAAAGAGACCTACAGCTGGGGCCATCATGCT

13,250 13,260 13,270 13,280 13,290 13,300 13,310

Hulutou CTGGCTTGACAAAGAGAGCAAACCGGTCCCAGTTTCAAGGGCAGGTGCAGGAGTGCTGCCAAGACCTCC
Baihulu CTGGCTTGACAAAGAGAGCAAACCGGTCCCAGTTTCAAGGGCAGGTGCAGGAGTGCTGCCAAGACCTCC
Chiyacao CTGGCTTGACAAAGAGAGCAAACCGGTCCCAGTTTCAAGGGCAGGTGCAGGAGTGCTGCCAAGACCTCC
Hongmangmai CTGGCTTGACAAAGAGAGCAAACCGGTCCCAGTTTCAAGGGCAGGTGCAGGAGTGCTGCCAAGACCTCC

13,320 13,330 13,340 13,350 13,360 13,370 13,380

Hulutou GGTCCCTACTAATATCAACCATGCAGGTCGCATCCAAGGTATTCCGATTGAGAAGTCTCAAGTGTTAAT
Baihulu GGTCCCTACTAATATCAACCATGCAGGTCGCATCCAAGGTATTCCGATTGAGAAGTCTCAAGTGTTAAT
Chiyacao GGTCCCTACTAATATCAACCATGCAGGTCGCATCCAAGGTATTCCGATTGAGAAGTCTCAAGTGTTAAT
Hongmangmai GGTCCCTACTAATATCAACCATGCAGGTCGCATCCAAGGTATTCCGATTGAGAAGTCTCAAGTGTTAAT

13,390 13,400 13,410 13,420 13,430 13,440 13,450

Hulutou TCCTTCGTTTGCATGCAATGAATATTCTTCGGTGAAAATACAAAATCTTCATCTACTTCTGTTTATGTA
Baihulu TCCTTCGTTTGCATGCAATGAATATTCTTCGGTGAAAATACAAAATCTTCATCTACTTCTGTTTATGTA
Chiyacao TCCTTCGTTTGCATGCAATGAATATTCTTCGGTGAAAATACAAAATCTTCATCTACTTCTGTTTATGTA
Hongmangmai TCCTTCGTTTGCATGCAATGAATATTCTTCGGTGAAAATACAAAATCTTCATCTACTTCTGTTTATGTA

13,460 13,470 13,480 13,490 13,500 13,510 13,520

Hulutou TTACTAAGCAGCCGCAAGCTATTCCCTACTTCTCAATTGTTAATTCCTTCATTAGCATGCAGTGAATAT
Baihulu TTACTAAGCAGCCGCAAGCTATTCCCTACTTCTCAATTGTTAATTCCTTCATTAGCATGCAGTGAATAT
Chiyacao TTACTAAGCAGCCGCAAGCTATTCCCTACTTCTCAATTGTTAATTCCTTCATTAGCATGCAGTGAATAT
Hongmangmai TTACTAAGCAGCCGCAAGCTATTCCCTACTTCTCAATTGTTAATTCCTTCATTAGCATGCAGTGAATAT

13,530 13,540 13,550 13,560 13,570 13,580 13,590

Hulutou TCTTTGGTGAAAATTGCATCTATGAAAGGAAATTAAAATACAGTGTCTTCATCTATTACTATTTCTATT
Baihulu TCTTTGGTGAAAATTGCATCTATGAAAGGAAATTAAAATACAGTGTCTTCATCTATTACTATTTCTATT
Chiyacao TCTTTGGTGAAAATTGCATCTATGAAAGGAAATTAAAATACAGTGTCTTCATCTATTACTATTTCTATT
Hongmangmai TCTTTGGTGAAAATTGCATCTATGAAAGGAAATTAAAATACAGTGTCTTCATCTATTACTATTTCTATT

13,600 13,610 13,620 13,630 13,640 13,650 13,660

Hulutou CATCCAAGCTGATCAGTAAAAGCATCCACATGTTATAAGGGCAATCTATATGTGGTATTTAAAATGTTA
Baihulu CATCCAAGCTGATCAGTAAAAGCATCCACATGTTATAAGGGCAATCTATATGTGGTATTTAAAATGTTA
Chiyacao CATCCAAGCTGATCAGTAAAAGCATCCACATGTTATAAGGGCAATCTATATGTGGTATTTAAAATGTTA
Hongmangmai CATCCAAGCTGATCAGTAAAAGCATCCACATGTTATAAGGGCAATCTATATGTGGTATTTAAAATGTTA

13,670 13,680 13,690 13,700 13,710 13,720 13,730

Hulutou TTTCTGTCGCTTTTCACATCGTATATGCAATCTAAGATAATGCTCTCCCTCTCACTGTAAATAAATATG
Baihulu TTTCTGTCGCTTTTCACATCGTATATGCAATCTAAGATAATGCTCTCCCTCTCACTGTAAATAAATATG
Chiyacao TTTCTGTCGCTTTTCACATCGTATATGCAATCTAAGATAATGCTCTCCCTCTCACTGTAAATAAATATG
Hongmangmai TTTCTGTCGCTTTTCACATCGTATATGCAATCTAAGATAATGCTCTCCCTCTCACTGTAAATAAATATG

13,740 13,750 13,760 13,770 13,780 13,790 13,800

Hulutou TTACCAGAAAAGGAGAAGGCGGGATTCCTGAAACGACACTTCGGATGGAAGAAGTAACCACCTGCGCTG
Baihulu TTACCAGAAAAGGAGAAGGCGGGATTCCTGAAACGACACTTCGGATGGAAGAAGTAACCACCTGCGCTG
Chiyacao TTACCAGAAAAGGAGAAGGCGGGATTCCTGAAACGACACTTCGGATGGAAGAAGTAACCACCTGCGCTG
Hongmangmai TTACCAGAAAAGGAGAAGGCGGGATTCCTGAAACGACACTTCGGATGGAAGAAGTAACCACCTGCGCTG

	13,810 13,820 13,830 13,840 13,850 13,860
Hulutou	GCTTGTATTCCTGTGAATATTATAGATGGTTCGACAAAGCTCGTGGTTAACCGAAGGAGGATATTTGAG
Baihulu	GCTTGTATTCCTGTGAATATTATAGATGGTTCGACAAAGCTCGTGGTTAACCGAAGGAGGATATTTGAG
Chiyacao	GCTTGTATTCCTGTGAATATTATAGATGGTTCGACAAAGCTCGTGGTTAACCGAAGGAGGATATTTGAG
Hongmangmai	GCTTGTATTCCTGTGAATATTATAGATGGTTCGACAAAGCTCGTGGTTAACCGAAGGAGGATATTTGAG
	13,870 13,880 13,890 13,900 13,910 13,920 13,930
Hulutou	AGCTTTTGTGTTAGAACATGGCCATCTTGTGCGTCCGCAGCAGCCGGGGTCATCAGGAGGGAGTGCTCA
Baihulu	AGCTTTTGTGTTAGAACATGGCCATCTTGTGCGTCCGCAGCAGCCGGGGTCATCAGGAGGGAGTGCTCA
Chiyacao	AGCTTTTGTGTTAGAACATGGCCATCTTGTGCGTCCGCAGCAGCCGGGGTCATCAGGAGGGAGTGCTCA
Hongmangmai	AGCTTTTGTGTTAGAACATGGCCATCTTGTGCGTCCGCAGCAGCCGGGGTCATCAGGAGGGAGTGCTCA
	13,940 13,950 13,960 13,970 13,980 13,990 14,000
Hulutou	CTGCATGGACCAGATCACGCTCTGTCAGCCATGGGAGTCGTGCGCACATAGTGCCACACATGTAACGTT
Baihulu	CTGCATGGACCAGATCACGCTCTGTCAGCCATGGGAGTCGTGCGCACATAGTGCCACACATGTAACGTT
Chiyacao	CTGCATGGACCAGATCACGCTCTGTCAGCCATGGGAGTCGTGCGCACATAGTGCCACACATGTAACGTT
Hongmangmai	CTGCATGGACCAGATCACGCTCTGTCAGCCATGGGAGTCGTGCGCACATAGTGCCACACATGTAACGTT
	14,010 14,020 14,030 14,040 14,050 14,060 14,070
Hulutou	TAGTTGTGTGATCTGAACTGTTATGTGTCGTGTAACCTACCTCATGTGTGTGTATGATAAAGAAAGAAC
Baihulu	TAGTTGTGTGATCTGAACTGTTATGTGTCGTGTAACCTACCTCATGTGTGTGTATGATAAAGAAAGAAC
Chiyacao	TAGTTGTGTGATCTGAACTGTTATGTGTCGTGTAACCTACCTCATGTGTGTGTATGATAAAGAAAGAAC
Hongmangmai	TAGTTGTGTGATCTGAACTGTTATGTGTCGTGTAACCTACCTCATGTGTGTGTATGATAAAGAAAGAAC
	14,080 14,090 14,100 14,110 14,120 14,130 14,140
Hulutou	CACATCAGTGGAAGAGTTAAACCAATCGTATGGTATGACAGGTGAGACGTCACATATGTGGAAGCAAAA
Baihulu	CACATCAGTGGAAGAGTTAAACCAATCGTATGGTATGACAGGTGAGACGTCACATATGTGGAAGCAAAA
Chiyacao	CACATCAGTGGAAGAGTTAAACCAATCGTATGGTATGACAGGTGAGACGTCACATATGTGGAAGCAAAA
Hongmangmai	CACATCAGTGGAAGAGTTAAACCAATCGTATGGTATGACAGGTGAGACGTCACATATGTGGAAGCAAAA
	14,150 14,160 14,170 14,180 14,190 14,200 14,210
Hulutou	AAGAGTTGGAGGTAAGGTGGTTTTCGTACATACTGGTTCTGATCCCTCTCATATTTTTGTATCCTTATT
Baihulu	AAGAGTTGGAGGTAAGGTGGTTTTCGTACATACTGGTTCTGATCCCTCTCATATTTTTGTATCCTTATT
Chiyacao	AAGAGTTGGAGGTAAGGTGGTTTTCGTACATACTGGTTCTGATCCCTCTCATATTTTTGTATCCTTATT
Hongmangmai	AAGAGTTGGAGGTAAGGTGGTTTTCGTACATACTGGTTCTGATCCCTCTCATATTTTTGTATCCTTATT
	14,220 14,230 14,240 14,250 14,260 14,270 14,280
Hulutou	ATCTACTCCCTCTGTCCCATAATGTAAGAGTGTTTTTGACATTAGTGTCAAAAACTGTGTCAAAAACGC
Baihulu	ATCTACTCCCTCTGTCCCATAATGTAAGAGTGTTTTTGACATTAGTGTCAAAAACTGTGTCAAAAACGC
Chiyacao	ATCTACTCCCTCTGTCCCATAATGTAAGAGTGTTTTTGACATTAGTGTCAAAAACTGTGTCAAAAACGC
Hongmangmai	ATCTACTCCCTCTGTCCCATAATGTAAGAGTGTTTTTGACATTAGTGTCAAAAACTGTGTCAAAAACGC
	14,290 14,300 14,310 14,320 14,330 14,340 14,350
Hulutou	TCTTACATTACTGGACGGAGGGAGCATGTAAGTTTCGTATTAATCGTACTATGCGCCATCTTGCTCTAT
Baihulu	TCTTACATTACTGGACGGAGGGAGCATGTAAGTTTCGTATTAATCGTACTATGCGCCATCTTGCTCTAT
Chiyacao	TCTTACATTACTGGACGGAGGGAGCATGTAAGTTTCGTATTAATCGTACTATGCGCCATCTTGCTCTAT
Hongmangmai	TCTTACATTACTGGACGGAGGGAGCATGTAAGTTTCGTATTAATCGTACTATGCGCCATCTTGCTCTAT
	14,360 14,370 14,380 14,390 14,400 14,410 14,420
Hulutou	GAATCCCTATGCGAGACTTCTTTCTTCTCACAATTGATTTATTATTTTATGGTAATCTTGTTTCACATT
Baihulu	GAATCCCTATGCGAGACTTCTTTCTTCTCACAATTGATTTATTATTTTATGGTAATCTTGTTTCACATT
Chiyacao	GAATCCCTATGCGAGACTTCTTTCTTCTCACAATTGATTTATTATTTTATGGTAATCTTGTTTCACATT
Hongmangmai	GAATCCCTATGCGAGACTTCTTTCTTCTCACAATTGATTTATTATTTTATGGTAATCTTGTTTCACATT
	14,430 14,440 14,450 14,460 14,470 14,480 14,490
Hulutou	TATTGACGTGATTTTATCACCATATCTTGGTGTTCTTTCCTTAAACACCCAAACATATCCGAAAATGGT
Baihulu	TATTGACGTGATTTTATCACCATATCTTGGTGTTCTTTCCTTAAACACCCAAACATATCCGAAAATGGT
Chiyacao	TATTGACGTGATTTTATCACCATATCTTGGTGTTCTTTCCTTAAACACCCAAACATATCCGAAAATGGT
Hongmangmai	TATTGACGTGATTTTATCACCATATCTTGGTGTTCTTTCCTTAAACACCCAAACATATCCGAAAATGGT

```
            14,500    14,510    14,520    14,530    14,540    14,550
Hulutou     TTCCCACGAAAACAGGCAAAAGAAAGGTGCACAAGCACGGTAAAATCCGGGAAAGCTTACTACGTAGGC
Baihulu     TTCCCACGAAAACAGGCAAAAGAAAGGTGCACAAGCACGGTAAAATCCGGGAAAGCTTACTACGTAGGC
Chiyacao    TTCCCACGAAAACAGGCAAAAGAAAGGTGCACAAGCACGGTAAAATCCGGGAAAGCTTACTACGTAGGC
Hongmangmai TTCCCACGAAAACAGGCAAAAGAAAGGTGCACAAGCACGGTAAAATCCGGGAAAGCTTACTACGTAGGC
            14,560  14,570    14,580    14,590    14,600    14,610    14,620
Hulutou     ACAAAACAAAGGGTAACAACATCATAAAATTTGGACTCATCGCAACTCACGAGGGGCTAAGTGCTAGCA
Baihulu     ACAAAACAAAGGGTAACAACATCATAAAATTTGGACTCATCGCAACTCACGAGGGGCTAAGTGCTAGCA
Chiyacao    ACAAAACAAAGGGTAACAACATCATAAAATTTGGACTCATCGCAACTCACGAGGGGCTAAGTGCTAGCA
Hongmangmai ACAAAACAAAGGGTAACAACATCATAAAATTTGGACTCATCGCAACTCACGAGGGGCTAAGTGCTAGCA
            14,630    14,640    14,650    14,660    14,670    14,680    14,690
Hulutou     TGCCTCGTGCACAAGATGGGGCTGCTTCTGGTCCACCTAGCTAGCTCCAACAATAGTAGCCTTGGCTCA
Baihulu     TGCCTCGTGCACAAGATGGGGCTGCTTCTGGTCCACCTAGCTAGCTCCAACAATAGTAGCCTTGGCTCA
Chiyacao    TGCCTCGTGCACAAGATGGGGCTGCTTCTGGTCCACCTAGCTAGCTCCAACAATAGTAGCCTTGGCTCA
Hongmangmai TGCCTCGTGCACAAGATGGGGCTGCTTCTGGTCCACCTAGCTAGCTCCAACAATAGTAGCCTTGGCTCA
            14,700    14,710    14,720    14,730    14,740    14,750    14,760
Hulutou     CATCTACACTACTATTAAAAAAACAAACATAAACTCCCTACAGCTACACCAACAACTGTACACGCGATT
Baihulu     CATCTACACTACTATTAAAAAAACAAACATAAACTCCCTACAGCTACACCAACAACTGTACACGCGATT
Chiyacao    CATCTACACTACTATTAAAAAAACAAACATAAACTCCCTACAGCTACACCAACAACTGTACACGCGATT
Hongmangmai CATCTACACTACTATTAAAAAAACAAACATAAACTCCCTACAGCTACACCAACAACTGTACACGCGATT
            14,770    14,780    14,790    14,800    14,810    14,820    14,830
Hulutou     ATCAAGAACGTCAGACCAATCTAATTAAATCAAATCCGAGAGTCTATATTATATCAATAGTCTGCCATA
Baihulu     ATCAAGAACGTCAGACCAATCTAATTAAATCAAATCCGAGAGTCTATATTATATCAATAGTCTGCCATA
Chiyacao    ATCAAGAACGTCAGACCAATCTAATTAAATCAAATCCGAGAGTCTATATTATATCAATAGTCTGCCATA
Hongmangmai ATCAAGAACGTCAGACCAATCTAATTAAATCAAATCCGAGAGTCTATATTATATCAATAGTCTGCCATA
            14,840    14,850    14,860    14,870    14,880    14,890    14,900
Hulutou     CAAGTTAACGGTCCAGATTAAATGTTCTGCCGGGGCAGACCACCTCGCGCACCTCTTTGTCCACAATTA
Baihulu     CAAGTTAACGGTCCAGATTAAATGTTCTGCCGGGGCAGACCACCTCGCGCACCTCTTTGTCCACAATTA
Chiyacao    CAAGTTAACGGTCCAGATTAAATGTTCTGCCGGGGCAGACCACCTCGCGCACCTCTTTGTCCACAATTA
Hongmangmai CAAGTTAACGGTCCAGATTAAATGTTCTGCCGGGGCAGACCACCTCGCGCACCTCTTTGTCCACAATTA
            14,910    14,920    14,930    14,940    14,950    14,960    14,970
Hulutou     ATGGGGATATATGGTAACCACCACGCGCACCTCTTGGTACTCAATTAGTGAGGATATTAATCCCGTAAT
Baihulu     ATGGGGATATATGGTAACCACCACGCGCACCTCTTGGTACTCAATTAGTGAGGATATTAATCCCGTAAT
Chiyacao    ATGGGGATATATGGTAACCACCACGCGCACCTCTTGGTACTCAATTAGTGAGGATATTAATCCCGTAAT
Hongmangmai ATGGGGATATATGGTAACCACCACGCGCACCTCTTGGTACTCAATTAGTGAGGATATTAATCCCGTAAT
            14,980    14,990    15,000    15,010    15,020    15,030    15,040
Hulutou     TTTCTCTCTGGCCTTTGCCTTAGTTGTTGTTAATAAGTGCCTTGCGGTTACTCCTCTGATGCCCCAAGG
Baihulu     TTTCTCTCTGGCCTTTGCCTTAGTTGTTGTTAATAAGTGCCTTGCGGTTACTCCTCTGATGCCCCAAGG
Chiyacao    TTTCTCTCTGGCCTTTGCCTTAGTTGTTGTTAATAAGTGCCTTGCGGTTACTCCTCTGATGCCCCAAGG
Hongmangmai TTTCTCTCTGGCCTTTGCCTTAGTTGTTGTTAATAAGTGCCTTGCGGTTACTCCTCTGATGCCCCAAGG
            15,050    15,060    15,070    15,080    15,090    15,100    15,110
Hulutou     ACCGCTGCCGCCTACTTCTCCTCCTCTGTTGCCGAGTCAATTGCAAGAAACCACCACATTTGCGGCTAG
Baihulu     ACCGCTGCCGCCTACTTCTCCTCCTCTGTTGCCGAGTCAATTGCAAGAAACCACCACATTTGCGGCTAG
Chiyacao    ACCGCTGCCGCCTACTTCTCCTCCTCTGTTGCCGAGTCAATTGCAAGAAACCACCACATTTGCGGCTAG
Hongmangmai ACCGCTGCCGCCTACTTCTCCTCCTCTGTTGCCGAGTCAATTGCAAGAAACCACCACATTTGCGGCTAG
            15,120    15,130    15,140    15,150    15,160    15,170    15,180
Hulutou     GTTTGCAGAAAACCACCAACTCGTTAATCCGTTGCAGAAAACACCGAAAAATTTGTTAAGTCATTGCAA
Baihulu     GTTTGCAGAAAACCACCAACTCGTTAATCCGTTGCAGAAAACACCGAAAAATTTGTTAAGTCATTGCAA
Chiyacao    GTTTGCAGAAAACCACCAACTCGTTAATCCGTTGCAGAAAACACCGAAAAATTTGTTAAGTCATTGCAA
Hongmangmai GTTTGCAGAAAACCACCAACTCGTTAATCCGTTGCAGAAAACACCGAAAAATTTGTTAAGTCATTGCAA
```

15,190 15,200 15,210 15,220 15,230 15,240

Hulutou AAAGCACTGATCGGATGATTTGGCCCGTTTAATCACTTTCTTACAAGTGGGGCCAGATTGTAAGGAATT
Baihulu AAAGCACTGATCGGATGATTTGGCCCGTTTAATCACTTTCTTACAAGTGGGGCCAGATTGTAAGGAATT
Chiyacao AAAGCACTGATCGGATGATTTGGCCCGTTTAATCACTTTCTTACAAGTGGGGCCAGATTGTAAGGAATT
Hongmangmai AAAGCACTGATCGGATGATTTGGCCCGTTTAATCACTTTCTTACAAGTGGGGCCAGATTGTAAGGAATT

15,250 15,260 15,270 15,280 15,290 15,300 15,310

Hulutou GACTTAGCAAAAAAATAACACATACACCCCTAGATCTAAAAAACAAAAGCAATCAGACCCTCCCCCGTG
Baihulu GACTTAGCAAAAAAATAACACATACACCCCTAGATCTAAAAAACAAAAGCAATCAGACCCTCCCCCGTG
Chiyacao GACTTAGCAAAAAAATAACACATACACCCCTAGATCTAAAAAACAAAAGCAATCAGACCCTCCCCCGTG
Hongmangmai GACTTAGCAAAAAAATAACACATACACCCCTAGATCTAAAAAACAAAAGCAATCAGACCCTCCCCCGTG

15,320 15,330 15,340 15,350 15,360 15,370 15,380

Hulutou GAACACTGACGCCTTGTACACTACGCGTCGCCGCGGCGCTGCTGCTATCCGCCGCCGCTATGCTCCGGG
Baihulu GAACACTGACGCCTTGTACACTACGCGTCGCCGCGGCGCTGCTGCTATCCGCCGCCGCTATGCTCCGGG
Chiyacao GAACACTGACGCCTTGTACACTACGCGTCGCCGCGGCGCTGCTGCTATCCGCCGCCGCTATGCTCCGGG
Hongmangmai GAACACTGACGCCTTGTACACTACGCGTCGCCGCGGCGCTGCTGCTATCCGCCGCCGCTATGCTCCGGG

15,390 15,400 15,410 15,420 15,430 15,440 15,450

Hulutou CCGGGAGGATGAGGGAGGAGGAGCCCAGCTGGTTCGGGCGCTTCGACGAGGACCTGCCGTCGCCGGACG
Baihulu CCGGGAGGATGAGGGAGGAGGAGCCCAGCTGGTTCGGGCGCTTCGACGAGGACCTGCCGTCGCCGGACG
Chiyacao CCGGGAGGATGAGGGAGGAGGAGCCCAGCTGGTTCGGGCGCTTCGACGAGGACCTGCCGTCGCCGGACG
Hongmangmai CCGGGAGGATGAGGGAGGAGGAGCCCAGCTGGTTCGGGCGCTTCGACGAGGACCTGCCGTCGCCGGACG

15,460 15,470 15,480 15,490 15,500 15,510 15,520

Hulutou AGCTCATTCCGCTCTCACACTGGCTCATCACCCGCGATCTCCCCGCCGCCTTCAACATCCCCACGCACG
Baihulu AGCTCATTCCGCTCTCACACTGGCTCATCACCCGCGATCTCCCCGCCGCCTTCAACATCCCCACGCACG
Chiyacao AGCTCATTCCGCTCTCACACTGGCTCATCACCCGCGATCTCCCCGCCGCCTTCAACATCCCCACGCACG
Hongmangmai AGCTCATTCCGCTCTCACACTGGCTCATCACCCGCGATCTCCCCGCCGCCTTCAACATCCCCACGCACG

15,530 15,540 15,550 15,560 15,570 15,580 15,590

Hulutou GGGCCGGCCGCTCAGCGATCTTGGCAAAGGAAAGAGGGAGCGAGGCGTCGCCGGGGAACACATCAATGA
Baihulu GGGCCGGCCGCTCAGCGATCTTGGCAAAGGAAAGAGGGAGCGAGGCGTCGCCGGGGAACACATCAATGA
Chiyacao GGGCCGGCCGCTCAGCGATCTTGGCAAAGGAAAGAGGGAGCGAGGCGTCGCCGGGGAACACATCAATGA
Hongmangmai GGGCCGGCCGCTCAGCGATCTTGGCAAAGGAAAGAGGGAGCGAGGCGTCGCCGGGGAACACATCAATGA

15,599

Hulutou AGGAC
Baihulu AGGAC
Chiyacao AGGAC
Hongmangmai AGGAC

b

```
            1        10        20        30        40        50        60
Hulutou     ATGGGCGGATACGAGTTCCAGAGGGCGGAGCTAGATGCACTGGAAGGCGTCGTACGCGATCCAACTGCG
Baihulu     ATGGGCGGATACGAGTTCCAGAGGGCGGAGCTAGATGCACTGGAAGGCGTCGTACGCGATCCAACTGCG
Chiyacao    ATGGGCGGATACGAGTTCCAGAGGGCGGAGCTAGATGCACTGGAAGGCGTCGTACGCGATCCAACTGCG
Hongmangmai ATGGGCGGATACGAGTTCCAGAGGGCGGAGCTAGATGCACTGGAAGGCGTCGTACGCGATCCAACTGCG
            70        80        90        100       110       120       130
Hulutou     GAGCCAATGAGTCTGACGTTGCCGCTTCTCAGGCACATAACAAATGATTTCTCCCCTGAATTTGAAATT
Baihulu     GAGCCAATGAGTCTGACGTTGCCGCTTCTCAGGCACATAACAAATGATTTCTCCCCTGAATTTGAAATT
Chiyacao    GAGCCAATGAGTCTGACGTTGCCGCTTCTCAGGCACATAACAAATGATTTCTCCCCTGAATTTGAAATT
Hongmangmai GAGCCAATGAGTCTGACGTTGCCGCTTCTCAGGCACATAACAAATGATTTCTCCCCTGAATTTGAAATT
             140       150       160       170       180       190       200
Hulutou     AGTAAAGATGATTCTGCAGTGGTTTACCTGGGGGTGCTTCCAAGTGGGTTCCGTGTTGCTGTCAAGAAG
Baihulu     AGTAAAGATGATTCTGCAGTGGTTTACCTGGGGGTGCTTCCAAGTGGGTTCCGTGTTGCTGTCAAGAAG
Chiyacao    AGTAAAGATGATTCTGCAGTGGTTTACCTGGGGGTGCTTCCAAGTGGGTTCCGTGTTGCTGTCAAGAAG
Hongmangmai AGTAAAGATGATTCTGCAGTGGTTTACCTGGGGGTGCTTCCAAGTGGGTTCCGTGTTGCTGTCAAGAAG
              210       220       230       240       250       260       270
Hulutou     TCTCACTTTCGTTTTTGCTTGGATGATGAAGATGCATTCACAAATGAAGTTTCTATTGCAATGAAGGCT
Baihulu     TCTCACTTTCGTTTTTGCTTGGATGATGAAGATGCATTCACAAATGAAGTTTCTATTGCAATGAAGGCT
Chiyacao    TCTCACTTTCGTTTTTGCTTGGATGATGAAGATGCATTCACAAATGAAGTTTCTATTGCAATGAAGGCT
Hongmangmai TCTCACTTTCGTTTTTGCTTGGATGATGAAGATGCATTCACAAATGAAGTTTCTATTGCAATGAAGGCT
               280       290       300       310       320       330       340
Hulutou     GCTCATAAGAACACAGTGCGAGTCATAGGCTACTGTCATCACACGCATGAGCAAATTGCCGAATACGAA
Baihulu     GCTCATAAGAACACAGTGCGAGTCATAGGCTACTGTCATCACACGCATGAGCAAATTGCCGAATACGAA
Chiyacao    GCTCATAAGAACACAGTGCGAGTCATAGGCTACTGTCATCACACGCATGAGCAAATTGCCGAATACGAA
Hongmangmai GCTCATAAGAACACAGTGCGAGTCATAGGCTACTGTCATCACACGCATGAGCAAATTGCCGAATACGAA
                350       360       370       380       390       400       410
Hulutou     GGAAAACAAGTTTTCGCAGAGGTCAGAGAAAGGTTGATCTGTACCGAGTATGTGCCTAACGGACCCCTT
Baihulu     GGAAAACAAGTTTTCGCAGAGGTCAGAGAAAGGTTGATCTGTACCGAGTATGTGCCTAACGGACCCCTT
Chiyacao    GGAAAACAAGTTTTCGCAGAGGTCAGAGAAAGGTTGATCTGTACCGAGTATGTGCCTAACGGACCCCTT
Hongmangmai GGAAAACAAGTTTTCGCAGAGGTCAGAGAAAGGTTGATCTGTACCGAGTATGTGCCTAACGGACCCCTT
                 420       430       440       450       460       470       480
Hulutou     AGTGGACATATCGAAGGTAAGATATGTGCGCAAATGGATGGATACGAGTTCCAGAGGGCAGAACTAGAT
Baihulu     AGTGGACATATCGAAGGTAAGATATGTGCGCAAATGGATGGATACGAGTTCCAGAGGGCAGAACTAGAT
Chiyacao    AGTGGACATATCGAAGGTAAGATATGTGCGCAAATGGATGGATACGAGTTCCAGAGGGCAGAACTAGAT
Hongmangmai AGTGGACATATCGAAGGTAAGATATGTGCGCAAATGGATGGATACGAGTTCCAGAGGGCAGAACTAGAT
                  490       500       510       520       530       540       550
Hulutou     GCACTAGAACGCGTCGTACGCGATACAAGTGCGGAGCCAATGAGTCTGACGTTGCCGCTTCTCAGGCAC
Baihulu     GCACTAGAACGCGTCGTACGCGATACAAGTGCGGAGCCAATGAGTCTGACGTTGCCGCTTCTCAGGCAC
Chiyacao    GCACTAGAACGCGTCGTACGCGATACAAGTGCGGAGCCAATGAGTCTGACGTTGCCGCTTCTCAGGCAC
Hongmangmai GCACTAGAACGCGTCGTACGCGATACAAGTGCGGAGCCAATGAGTCTGACGTTGCCGCTTCTCAGGCAC
                   560       570       580       590       600       610       620
Hulutou     ATAACAAATGATTTCTCCGATGAATCTCGAATTGGCCGAGGTGGATTCGCAGTGGTTTACCTGGGGGTG
Baihulu     ATAACAAATGATTTCTCCGATGAATCTCGAATTGGCCGAGGTGGATTCGCAGTGGTTTACCTGGGGGTG
Chiyacao    ATAACAAATGATTTCTCCGATGAATCTCGAATTGGCCGAGGTGGATTCGCAGTGGTTTACCTGGGGGTG
Hongmangmai ATAACAAATGATTTCTCCGATGAATCTCGAATTGGCCGAGGTGGATTCGCAGTGGTTTACCTGGGGGTG
                    630       640       650       660       670       680       690
Hulutou     CTTCCAAGTGGGTTACGTATTGCTGTTAAGAGGCTTAGCAATATTGCTTATATGAACGAAAGTGCATTT
Baihulu     CTTCCAAGTGGGTTACGTATTGCTGTTAAGAGGCTTAGCAATATTGCTTATATGAACGAAAGTGCATTT
Chiyacao    CTTCCAAGTGGGTTACGTATTGCTGTTAAGAGGCTTAGCAATATTGCTTATATGAACGAAAGTGCATTT
Hongmangmai CTTCCAAGTGGGTTACGTATTGCTGTTAAGAGGCTTAGCAATATTGCTTATATGAACGAAAGTGCATTT
```

```
                 700        710        720        730        740        750
Hulutou      CAAAATGAAGTGTTTATCACAATGAAGGCCACTCACAAGAACACAGTGCGATTCATGGGCTACTGTAGT
Baihulu      CAAAATGAAGTGTTTATCACAATGAAGGCCACTCACAAGAACACAGTGCGATTCATGGGCTACTGTAGT
Chiyacao     CAAAATGAAGTGTTTATCACAATGAAGGCCACTCACAAGAACACAGTGCGATTCATGGGCTACTGTAGT
Hongmangmai  CAAAATGAAGTGTTTATCACAATGAAGGCCACTCACAAGAACACAGTGCGATTCATGGGCTACTGTAGT
             760        770        780        790        800        810        820
Hulutou      CAAATACAAGGTAAACTCATCGAACACGACGGGCAACATGTTTTCGCACAGCTCGAGGAAAGGTTGATC
Baihulu      CAAATACAAGGTAAACTCATCGAACACGACGGGCAACATGTTTTCGCACAGCTCGAGGAAAGGTTGATC
Chiyacao     CAAATACAAGGTAAACTCATCGAACACGACGGGCAACATGTTTTCGCACAGCTCGAGGAAAGGTTGATC
Hongmangmai  CAAATACAAGGTAAACTCATCGAACACGACGGGCAACATGTTTTCGCACAGCTCGAGGAAAGGTTGATC
              830        840        850        860        870        880        890
Hulutou      TGTGTGGAATATGCGCCTAAAGGAACCCTTGATGCACATATCGGTGACTATGGTGAACTTGACTGGAAC
Baihulu      TGTGTGGAATATGCGCCTAAAGGAACCCTTGATGCACATATCGGTGACTATGGTGAACTTGACTGGAAC
Chiyacao     TGTGTGGAATATGCGCCTAAAGGAACCCTTGATGCACATATCGGTGACTATGGTGAACTTGACTGGAAC
Hongmangmai  TGTGTGGAATATGCGCCTAAAGGAACCCTTGATGCACATATCGGTGACTATGGTGAACTTGACTGGAAC
               900        910        920        930        940        950        960
Hulutou      CAGCGTTATCAAATTCTAAAAGGAATTTGTCAAGGTTTGCATCATCTCCATGACGAAATGCACGTTTTT
Baihulu      CAGCGTTATCAAATTCTAAAAGGAATTTGTCAAGGTTTGCATCATCTCCATGACGAAATGCACGTTTTT
Chiyacao     CAGCGTTATCAAATTCTAAAAGGAATTTGTCAAGGTTTGCATCATCTCCATGACGAAATGCACGTTTTT
Hongmangmai  CAGCGTTATCAAATTCTAAAAGGAATTTGTCAAGGTTTGCATCATCTCCATGACGAAATGCACGTTTTT
                970        980        990      1,000      1,010      1,020      1,030
Hulutou      CATGGAGATATCAAACCAGCCAATATATTAATAGGGGATAACCTTGTGCCTAAAATCTATGACTTCGGT
Baihulu      CATGGAGATATCAAACCAGCCAATATATTAATAGGGGATAACCTTGTGCCTAAAATCTATGACTTCGGT
Chiyacao     CATGGAGATATCAAACCAGCCAATATATTAATAGGGGATAACCTTGTGCCTAAAATCTATGACTTCGGT
Hongmangmai  CATGGAGATATCAAACCAGCCAATATATTAATAGGGGATAACCTTGTGCCTAAAATCTATGACTTCGGT
               1,040      1,050      1,060      1,070      1,080      1,090      1,100
Hulutou      CTCTCCCAGATGTTTGAAGAAGAAGAAACGGAACGTATTGTTGAAAATATCGCCGGAACATTCGGATAT
Baihulu      CTCTCCCAGATGTTTGAAGAAGAAGAAACGGAACGTATTGTTGAAAATATCGCCGGAACATTCGGATAT
Chiyacao     CTCTCCCAGATGTTTGAAGAAGAAGAAACGGAACGTATTGTTGAAAATATCGCCGGAACATTCGGATAT
Hongmangmai  CTCTCCCAGATGTTTGAAGAAGAAGAAACGGAACGTATTGTTGAAAATATCGCCGGAACATTCGGATAT
                1,110      1,120      1,130      1,140      1,150      1,160      1,170
Hulutou      ATGGCACCGGAGTTTTGTACTAATAATATGGTGTCATTTAAGGCTGAGATATACAGTTTGGGCGTTGTG
Baihulu      ATGGCACCGGAGTTTTGTACTAATAATATGGTGTCATTTAAGGCTGAGATATACAGTTTGGGCGTTGTG
Chiyacao     ATGGCACCGGAGTTTTGTACTAATAATATGGTGTCATTTAAGGCTGAGATATACAGTTTGGGCGTTGTG
Hongmangmai  ATGGCACCGGAGTTTTGTACTAATAATATGGTGTCATTTAAGGCTGAGATATACAGTTTGGGCGTTGTG
                 1,180      1,190      1,200      1,210      1,220      1,230      1,240
Hulutou      ATCGGGGAGTTATTGATCGGGAAGTGGTTTGATGAGGATGTGAGAAAACTATTTGTACAGCAACTTAAG
Baihulu      ATCGGGGAGTTATTGATCGGGAAGTGGTTTGATGAGGATGTGAGAAAACTATTTGTACAGCAACTTAAG
Chiyacao     ATCGGGGAGTTATTGATCGGGAAGTGGTTTGATGAGGATGTGAGAAAACTATTTGTACAGCAACTTAAG
Hongmangmai  ATCGGGGAGTTATTGATCGGGAAGTGGTTTGATGAGGATGTGAGAAAACTATTTGTACAGCAACTTAAG
                  1,250      1,260      1,270      1,280      1,290      1,300      1,310
Hulutou      GGTTTGAGAAAAACATTGGTAAAAGAAGGAGCGTTTTCATCATGGGAAAACAAATACCACCAAGTTAGA
Baihulu      GGTTTGAGAAAAACATTGGTAAAAGAAGGAGCGTTTTCATCATGGGAAAACAAATACCACCAAGTTAGA
Chiyacao     GGTTTGAGAAAAACATTGGTAAAAGAAGGAGCGTTTTCATCATGGGAAAACAAATACCACCAAGTTAGA
Hongmangmai  GGTTTGAGAAAAACATTGGTAAAAGAAGGAGCGTTTTCATCATGGGAAAACAAATACCACCAAGTTAGA
                   1,320      1,330      1,340      1,350      1,360      1,370      1,380
Hulutou      ACATGTATGGAGATTGGGCAGGACTGCATAGACCCCAACCCACATAAAAGGCCCACTTTGTTGGAGATT
Baihulu      ACATGTATGGAGATTGGGCAGGACTGCATAGACCCCAACCCACATAAAAGGCCCACTTTGTTGGAGATT
Chiyacao     ACATGTATGGAGATTGGGCAGGACTGCATAGACCCCAACCCACATAAAAGGCCCACTTTGTTGGAGATT
Hongmangmai  ACATGTATGGAGATTGGGCAGGACTGCATAGACCCCAACCCACATAAAAGGCCCACTTTGTTGGAGATT
```

```
            1,390     1,400     1,410     1,420     1,430     1,440
Hulutou     ATCCAGCGGCTTAATGAAGCGGAAGATATGAACTATTCTGCAGCATCACTTTGGCAGTCAGGAGACGAG
Baihulu     ATCCAGCGGCTTAATGAAGCGGAAGATATGAACTATTCTGCAGCATCACTTTGGCAGTCAGGAGACGAG
Chiyacao    ATCCAGCGGCTTAATGAAGCGGAAGATATGAACTATTCTGCAGCATCACTTTGGCAGTCAGGAGACGAG
Hongmangmai ATCCAGCGGCTTAATGAAGCGGAAGATATGAACTATTCTGCAGCATCACTTTGGCAGTCAGGAGACGAG
            1,450     1,460     1,470     1,480     1,490     1,500     1,510
Hulutou     GAATCCGATTTATCGGATACAGAAGCTTTGGAGACAGAGACAACATCCGAGTTTCTTCCAAGTGACGAA
Baihulu     GAATCCGATTTATCGGATACAGAAGCTTTGGAGACAGAGACAACATCCGAGTTTCTTCCAAGTGACGAA
Chiyacao    GAATCCGATTTATCGGATACAGAAGCTTTGGAGACAGAGACAACATCCGAGTTTCTTCCAAGTGACGAA
Hongmangmai GAATCCGATTTATCGGATACAGAAGCTTTGGAGACAGAGACAACATCCGAGTTTCTTCCAAGTGACGAA
            1,520     1,530     1,540     1,550     1,560     1,570     1,580
Hulutou     GAACCCGCCTCTGTGGGCAAGACCGGAGAAACAAGCACACAGGAGCCTGATAAACCGGACCTAATAAGT
Baihulu     GAACCCGCCTCTGTGGGCAAGACCGGAGAAACAAGCACACAGGAGCCTGATAAACCGGACCTAATAAGT
Chiyacao    GAACCCGCCTCTGTGGGCAAGACCGGAGAAACAAGCACACAGGAGCCTGATAAACCGGACCTAATAAGT
Hongmangmai GAACCCGCCTCTGTGGGCAAGACCGGAGAAACAAGCACACAGGAGCCTGATAAACCGGACCTAATAAGT
            1,590     1,600     1,610     1,620     1,630     1,640     1,650
Hulutou     AAGTTGCCAGCATCGGTGGACCTGTCTGACCTAAAAGTCCTGGAGAAAATCACAGATGATTTTTCACAC
Baihulu     AAGTTGCCAGCATCGGTGGACCTGTCTGACCTAAAAGTCCTGGAGAAAATCACAGATGATTTTTCACAC
Chiyacao    AAGTTGCCAGCATCGGTGGACCTGTCTGACCTAAAAGTCCTGGAGAAAATCACAGATGATTTTTCACAC
Hongmangmai AAGTTGCCAGCATCGGTGGACCTGTCTGACCTAAAAGTCCTGGAGAAAATCACAGATGATTTTTCACAC
            1,660     1,670     1,680     1,690     1,700     1,710     1,720
Hulutou     GAAAGAATAGTTGGGAAGGACGGTACATTCAAAGGTTGTCATAAGGCATTTGTTTATAAGGGTGACATT
Baihulu     GAAAGAATAGTTGGGAAGGACGGTACATTCAAAGGTTGTCATAAGGCATTTGTTTATAAGGGTGACATT
Chiyacao    GAAAGAATAGTTGGGAAGGACGGTACATTCAAAGGTTGTCATAAGGCATTTGTTTATAAGGGTGACATT
Hongmangmai GAAAGAATAGTTGGGAAGGACGGTACATTCAAAGGTTGTCATAAGGCATTTGTTTATAAGGGTGACATT
            1,730     1,740     1,750     1,760     1,770     1,780     1,790
Hulutou     CCACTTAGAGAAATGATAGCCGTGAAGAGGTTAATTGGAGTGGAGATTCCATTTGAAAAGTTTAAGAGG
Baihulu     CCACTTAGAGAAATGATAGCCGTGAAGAGGTTAATTGGAGTGGAGATTCCATTTGAAAAGTTTAAGAGG
Chiyacao    CCACTTAGAGAAATGATAGCCGTGAAGAGGTTAATTGGAGTGGAGATTCCATTTGAAAAGTTTAAGAGG
Hongmangmai CCACTTAGAGAAATGATAGCCGTGAAGAGGTTAATTGGAGTGGAGATTCCATTTGAAAAGTTTAAGAGG
            1,800     1,810     1,820     1,830     1,840     1,850     1,860
Hulutou     GAAGCAGAACAGTTCATTAGTCTCGATCATAAGAATATAGTAAAGGTTGCCAGCTACTGCCACGACCAG
Baihulu     GAAGCAGAACAGTTCATTAGTCTCGATCATAAGAATATAGTAAAGGTTGCCAGCTACTGCCACGACCAG
Chiyacao    GAAGCAGAACAGTTCATTAGTCTCGATCATAAGAATATAGTAAAGGTTGCCAGCTACTGCCACGACCAG
Hongmangmai GAAGCAGAACAGTTCATTAGTCTCGATCATAAGAATATAGTAAAGGTTGCCAGCTACTGCCACGACCAG
            1,870     1,880     1,890     1,900     1,910     1,920     1,930
Hulutou     TCTAGAGGACATAGACTGGTACAGTTCAAAGGAAAACCGCTACCACAACTCTTTAACGGTCCCGAACAA
Baihulu     TCTAGAGGACATAGACTGGTACAGTTCAAAGGAAAACCGCTACCACAACTCTTTAACGGTCCCGAACAA
Chiyacao    TCTAGAGGACATAGACTGGTACAGTTCAAAGGAAAACCGCTACCACAACTCTTTAACGGTCCCGAACAA
Hongmangmai TCTAGAGGACATAGACTGGTACAGTTCAAAGGAAAACCGCTACCACAACTCTTTAACGGTCCCGAACAA
            1,940     1,950     1,960     1,970     1,980     1,990     2,000
Hulutou     CTGCTCTGCTATGAATATATGCACAACGGAAGCCTTCGCGACTATCTTATGGGTCAAGGATCTCGTGTA
Baihulu     CTGCTCTGCTATGAATATATGCACAACGGAAGCCTTCGCGACTATCTTATGGGTCAAGGATCTCGTGTA
Chiyacao    CTGCTCTGCTATGAATATATGCACAACGGAAGCCTTCGCGACTATCTTATGGGTCAAGGATCTCGTGTA
Hongmangmai CTGCTCTGCTATGAATATATGCACAACGGAAGCCTTCGCGACTATCTTATGGGTCAAGGATCTCGTGTA
            2,010     2,020     2,030     2,040     2,050     2,060     2,070
Hulutou     ATTGATTGGCAAATGCGCTACAAATTGATCAAAGGGACTTGCGCAGGCTTACATTACCTTCACAAGGGC
Baihulu     ATTGATTGGCAAATGCGCTACAAATTGATCAAAGGGACTTGCGCAGGCTTACATTACCTTCACAAGGGC
Chiyacao    ATTGATTGGCAAATGCGCTACAAATTGATCAAAGGGACTTGCGCAGGCTTACATTACCTTCACAAGGGC
Hongmangmai ATTGATTGGCAAATGCGCTACAAATTGATCAAAGGGACTTGCGCAGGCTTACATTACCTTCACAAGGGC
```

```
             2,080    2,090    2,100    2,110    2,120    2,130
Hulutou      CGTGCAGGTTGTCCAATTGTTCATTTGAATTTAAGCCCGTCAAATGTATTGCTGGACCACAACTACATA
Baihulu      CGTGCAGGTTGTCCAATTGTTCATTTGAATTTAAGCCCGTCAAATGTATTGCTGGACCACAACTACATA
Chiyacao     CGTGCAGGTTGTCCAATTGTTCATTTGAATTTAAGCCCGTCAAATGTATTGCTGGACCACAACTACATA
Hongmangmai  CGTGCAGGTTGTCCAATTGTTCATTTGAATTTAAGCCCGTCAAATGTATTGCTGGACCACAACTACATA
             2,140    2,150    2,160    2,170    2,180    2,190    2,200
Hulutou      CCACGCATCACAGGGTTCGATTTTTCGAAGCTCATTGGTGAAAAGAACACCAAATCAGTGGTACTTAAG
Baihulu      CCACGCATCACAGGGTTCGATTTTTCGAAGCTCATTGGTGAAAAGAACACCAAATCAGTGGTACTTAAG
Chiyacao     CCACGCATCACAGGGTTCGATTTTTCGAAGCTCATTGGTGAAAAGAACACCAAATCAGTGGTACTTAAG
Hongmangmai  CCACGCATCACAGGGTTCGATTTTTCGAAGCTCATTGGTGAAAAGAACACCAAATCAGTGGTACTTAAG
             2,210    2,220    2,230    2,240    2,250    2,260    2,270
Hulutou      CTGAATGGACCCATAGCGTACCTGCCACCGGATTTCTTCCATTCGAAGGGTACTGATCTTAAATATCTT
Baihulu      CTGAATGGACCCATAGCGTACCTGCCACCGGATTTCTTCCATTCGAAGGGTACTGATCTTAAATATCTT
Chiyacao     CTGAATGGACCCATAGCGTACCTGCCACCGGATTTCTTCCATTCGAAGGGTACTGATCTTAAATATCTT
Hongmangmai  CTGAATGGACCCATAGCGTACCTGCCACCGGATTTCTTCCATTCGAAGGGTACTGATCTTAAATATCTT
             2,280    2,290    2,300    2,310    2,320    2,330    2,340
Hulutou      GCTACGGTAGATATATACAGCTTGGGTCTTATGATTTTAGAAATCGCAACACAACAAGAGATCAAAGGC
Baihulu      GCTACGGTAGATATATACAGCTTGGGTCTTATGATTTTAGAAATCGCAACACAACAAGAGATCAAAGGC
Chiyacao     GCTACGGTAGATATATACAGCTTGGGTCTTATGATTTTAGAAATCGCAACACAACAAGAGATCAAAGGC
Hongmangmai  GCTACGGTAGATATATACAGCTTGGGTCTTATGATTTTAGAAATCGCAACACAACAAGAGATCAAAGGC
             2,350    2,360    2,370    2,380    2,390    2,400    2,410
Hulutou      ATCCATGGAGTGCTTATTAAGAGTATAGAGGAAAACTGGAGGGAGGAGTCACAAATAACACGGCTGTAT
Baihulu      ATCCATGGAGTGCTTATTAAGAGTATAGAGGAAAACTGGAGGGAGGAGTCACAAATAACACGGCTGTAT
Chiyacao     ATCCATGGAGTGCTTATTAAGAGTATAGAGGAAAACTGGAGGGAGGAGTCACAAATAACACGGCTGTAT
Hongmangmai  ATCCATGGAGTGCTTATTAAGAGTATAGAGGAAAACTGGAGGGAGGAGTCACAAATAACACGGCTGTAT
             2,420    2,430    2,440    2,450    2,460    2,470    2,480
Hulutou      ACCTCACTAGGGGCCGACGAGCTGCGGCAAGTAAAAATGTGCATTGATATTGGCCTAGACTGTGTCAAG
Baihulu      ACCTCACTAGGGGCCGACGAGCTGCGGCAAGTAAAAATGTGCATTGATATTGGCCTAGACTGTGTCAAG
Chiyacao     ACCTCACTAGGGGCCGACGAGCTGCGGCAAGTAAAAATGTGCATTGATATTGGCCTAGACTGTGTCAAG
Hongmangmai  ACCTCACTAGGGGCCGACGAGCTGCGGCAAGTAAAAATGTGCATTGATATTGGCCTAGACTGTGTCAAG
             2,490    2,500    2,510    2,520    2,530    2,540    2,550
Hulutou      TCAAACCCTGAAAAGAGACCTACAGCTGGGGCCATCATGCTCTGGCTTGACAAAGAGAGCAAACCGGTC
Baihulu      TCAAACCCTGAAAAGAGACCTACAGCTGGGGCCATCATGCTCTGGCTTGACAAAGAGAGCAAACCGGTC
Chiyacao     TCAAACCCTGAAAAGAGACCTACAGCTGGGGCCATCATGCTCTGGCTTGACAAAGAGAGCAAACCGGTC
Hongmangmai  TCAAACCCTGAAAAGAGACCTACAGCTGGGGCCATCATGCTCTGGCTTGACAAAGAGAGCAAACCGGTC
             2,560    2,570    2,580    2,590    2,600    2,610    2,620
Hulutou      CCAGTTTCAAGGGCAGGTGCAGGAGTGCTGCCAAGACCTCCGGTCCCTACTAATATCAACCATGCAGGT
Baihulu      CCAGTTTCAAGGGCAGGTGCAGGAGTGCTGCCAAGACCTCCGGTCCCTACTAATATCAACCATGCAGGT
Chiyacao     CCAGTTTCAAGGGCAGGTGCAGGAGTGCTGCCAAGACCTCCGGTCCCTACTAATATCAACCATGCAGGT
Hongmangmai  CCAGTTTCAAGGGCAGGTGCAGGAGTGCTGCCAAGACCTCCGGTCCCTACTAATATCAACCATGCAGGT
             2,630    2,640    2,650    2,660    2,670    2,682
Hulutou      CGCATCCAAGAAAAGGAGAAGGCGGGATTCCTGAAACGACACTTCGGATGGAAGAAGTAA
Baihulu      CGCATCCAAGAAAAGGAGAAGGCGGGATTCCTGAAACGACACTTCGGATGGAAGAAGTAA
Chiyacao     CGCATCCAAGAAAAGGAGAAGGCGGGATTCCTGAAACGACACTTCGGATGGAAGAAGTAA
Hongmangmai  CGCATCCAAGAAAAGGAGAAGGCGGGATTCCTGAAACGACACTTCGGATGGAAGAAGTAA
```

Supplementary Data 2 Genomic DNA and CDS alignments of *WTK3* gene in Chinese wheat landraces CYC, BHL, HLT, and HMM

a Genomic DNA alignments of WTK3 gene in Chinese wheat landraces CYC, BHL, HLT, and HMM. The sequence includes a 3,378 bp presumed native promoter, the 10,410 bp entire gene body including exons and introns, and a 1,811 bp terminator region of the WTK3 gene. **b** CDS alignments of the WTK3 gene in Chinese wheat landraces CYC, BHL, HLT, and HMM.
