# Supplementary material for: A rare gain of function mutation in a wheat tandem kinase confers resistance to powdery mildew
Source: Nat Commun. 2020 Feb 3;11:680. doi: 10.1038/s41467-020-14294-0 (PMC6997164; doi:10.1038/s41467-020-14294-0)
Supplement: Supplementary file 7 — Supplementary Data 3 [file 41467_2020_14294_MOESM7_ESM.pdf]

	1 10 20 30 40 50 60
Hulutou	ATGGGCGGATACGAGTTCCAGAGGGCGGAGCTAGATGCACTGGAAGGCGTCGTACGCGATCCAACTGC
Baihulu	ATGGGCGGATACGAGTTCCAGAGGGCGGAGCTAGATGCACTGGAAGGCGTCGTACGCGATCCAACTGC
Chiyacao	ATGGGCGGATACGAGTTCCAGAGGGCGGAGCTAGATGCACTGGAAGGCGTCGTACGCGATCCAACTGC
Hongmangmai	ATGGGCGGATACGAGTTCCAGAGGGCGGAGCTAGATGCACTGGAAGGCGTCGTACGCGATCCAACTGC
Hongmai	ATGGGCGGATACGAGTTCCAGAGGGCGGAGCTAGATGCACTGGAAGGCGTCGTACGCGATCCAACTGC
Wangshuibai	ATGGGCGGATACGAGTTCCAGAGGGCGGAGCTAGATGCACTGGAAGGCGTCGTACGCGATCCAACTGC
Shi 4185	ATGGGCGGATACGAGTTCCAGAGGGCGGAGCTAGATGCACTGGAAGGCGTCGTACGCGATCCAACTGC
Mazhamai	ATGGGCGGATACGAGTTCCAGAGGGCGGAGCTAGATGCACTGGAAGGCGTCGTACGCGATCCAACTGC
Xiaofoshou	ATGGGCGGATACGAGTTCCAGAGGGCGGAGCTAGATGCACTGGAAGGCGTCGTACGCGATCCAACTGC
Aikang 58	ATGGGCGGATACGAGTTCCAGAGGGCGGAGCTAGATGCACTGGAAGGCGTCGTACGCGATCCAACTGC
AL8/78	ATGGGCGGATACGAGTTCCAGAGGGCGGAGCTAGATGCACTGGAAGGCG-------------------
PI 431602	ATGGGCGGATACGAGTTCCAGAGGGCGGAGCTAGATGCACTGGAAGGCG-------------------
PI 486274	ATGGGCGGATACGAGTTCCAGAGGGCGGAGCTAGATGCACTGGAAGGCG-------------------
RM 000182	ATGGGCGGACACGAGTTCCACAGGGCGGAGCTAGATGCACTGGAAGGCGTCGTACGCGATCCAACTGC
PI 511381	ATGGGCGGACACGAGTTCCACAGGGCGGAGCTAGATGCACTGGAAGGCGTCGTACGCGATCCAACTGC
PI 486271	ATGGGCGGACACGAGTTCCACAGGGCGGAGCTAGATGCACTGGAAGGCGTCGTACGCGATCCAACTGC
PI 511367	ATGGGCGGACACGAGTTCCACAGGGCGGAGCTAGATGCACTGGAAGGCGTCGTACGCGATCCAACTGC
Chinese Spring	ATGGGCGGATACGAGTTCCAGAGGGCGGAGCTAGATGCACTGGAAGGCGTCGTACGCGATCCAACTGC
Hanzhongbai	ATGGGCGGATACGAGTTCCAGAGGGCGGAGCTAGATGCACTGGAAGGCGTCGTACGCGATCCAACTGC
Hongtoumai	ATGGGCGGATACGAGTTCCAGAGGGCGGAGCTAGATGCACTGGAAGGCGTCGTACGCGATCCAACTGC
Hongyouzi	ATGGGCGGATACGAGTTCCAGAGGGCGGAGCTAGATGCACTGGAAGGCGTCGTACGCGATCCAACTGC
Zijiehong	ATGGGCGGATACGAGTTCCAGAGGGCGGAGCTAGATGCACTGGAAGGCGTCGTACGCGATCCAACTGC
Baihuamai	ATGGGCGGATACGAGTTCCAGAGGGCGGAGCTAGATGCACTGGAAGGCGTCGTACGCGATCCAACTGC
Huixianhong	ATGGGCGGATACGAGTTCCAGAGGGCGGAGCTAGATGCACTGGAAGGCGTCGTACGCGATCCAACTGC
Baimangmai	ATGGGCGGATACGAGTTCCAGAGGGCGGAGCTAGATGCACTGGAAGGCGTCGTACGCGATCCAACTGC

	70 80 90 100 110 120 130
Hulutou	GGAGCCAATGAGTCTGACGTTGCCGCTTCTCAGGCACATAACAAATGATTTCTCCCCTGAATTTGAAA
Baihulu	GGAGCCAATGAGTCTGACGTTGCCGCTTCTCAGGCACATAACAAATGATTTCTCCCCTGAATTTGAAA
Chiyacao	GGAGCCAATGAGTCTGACGTTGCCGCTTCTCAGGCACATAACAAATGATTTCTCCCCTGAATTTGAAA
Hongmangmai	GGAGCCAATGAGTCTGACGTTGCCGCTTCTCAGGCACATAACAAATGATTTCTCCCCTGAATTTGAAA
Hongmai	GGAGCCAATGAGTCTGACGTTGCCGCTTCTCAGGCACATAACAAATGATTTCTCCCCTGAATTTGAAA
Wangshuibai	GGAGCCAATGAGTCTGACGTTGCCGCTTCTCAGGCACATAACAAATGATTTCTCCCCTGAATTTGAAA
Shi 4185	GGAGCCAATGAGTCTGACGTTGCCGCTTCTCAGGCACATAACAAATGATTTCTCCCCTGAATTTGAAA
Mazhamai	GGAGCCAATGAGTCTGACGTTGCCGCTTCTCAGGCACATAACAAATGATTTCTCCCCTGAATTTGAAA
Xiaofoshou	GGAGCCAATGAGTCTGACGTTGCCGCTTCTCAGGCACATAACAAATGATTTCTCCCCTGAATTTGAAA
Aikang 58	GGAGCCAATGAGTCTGACGTTGCCGCTTCTCAGGCACATAACAAATGATTTCTCCCCTGAATTTGAAA
AL8/78	--
PI 431602	--
PI 486274	--
RM 000182	GGAGCCAATGAGTCTGACGTTGCCGCTTCTCAGGCACATAACAAATGATTTCTCCCCTGAATTTGAAA
PI 511381	GGAGCCAATGAGTCTGACGTTGCCGCTTCTCAGGCACATAACAAATGATTTCTCCCCTGAATTTGAAA
PI 486271	GGAGCCAATGAGTCTGACGTTGCCGCTTCTCAGGCACATAACAAATGATTTCTCCCCTGAATTTGAAA
PI 511367	GGAGCCAATGAGTCTGACGTTGCCGCTTCTCAGGCACATAACAAATGATTTCTCCCCTGAATTTGAAA
Chinese Spring	GGAGCCAATGAGTCTGACGTTGCCGCTTCTCAGGCACATAACAAATGATTTCTCCCCTGAATTTGAAA
Hanzhongbai	GGAGCCAATGAGTCTGACGTTGCCGCTTCTCAGGCACATAACAAATGATTTCTCCCCTGAATTTGAAA
Hongtoumai	GGAGCCAATGAGTCTGACGTTGCCGCTTCTCAGGCACATAACAAATGATTTCTCCCCTGAATTTGAAA
Hongyouzi	GGAGCCAATGAGTCTGACGTTGCCGCTTCTCAGGCACATAACAAATGATTTCTCCCCTGAATTTGAAA
Zijiehong	GGAGCCAATGAGTCTGACGTTGCCGCTTCTCAGGCACATAACAAATGATTTCTCCCCTGAATTTGAAA
Baihuamai	GGAGCCAATGAGTCTGACGTTGCCGCTTCTCAGGCACATAACAAATGATTTCTCCCCTGAATTTGAAA
Huixianhong	GGAGCCAATGAGTCTGACGTTGCCGCTTCTCAGGCACATAACAAATGATTTCTCCCCTGAATTTGAAA
Baimangmai	GGAGCCAATGAGTCTGACGTTGCCGCTTCTCAGGCACATAACAAATGATTTCTCCCCTGAATTTGAAA

```
                140       150       160       170       180       190       200
Hulutou         TTAGTAAAGATGATTCTGCAGTGGTTTACCTGGTACGACTAAACTCATATCTCGTTTATTATTAATAT
Baihulu         TTAGTAAAGATGATTCTGCAGTGGTTTACCTGGTACGACTAAACTCATATCTCGTTTATTATTAATAT
Chiyacao        TTAGTAAAGATGATTCTGCAGTGGTTTACCTGGTACGACTAAACTCATATCTCGTTTATTATTAATAT
Hongmangmai     TTAGTAAAGATGATTCTGCAGTGGTTTACCTGGTACGACTAAACTCATATCTCGTTTATTATTAATAT
Hongmai         TTAGTAAAGATGATTCTGCAGTGGTTTACCTGGTACGACTAAACTCATATCTCGTTTATTATTAATAT
Wangshuibai     TTAGTAAAGATGATTCTGCAGTGGTTTACCTGGTACGACTAAACTCATATCTCGTTTATTATTAATAT
Shi 4185        TTAGTAAAGATGATTCTGCAGTGGTTTACCTGGTACGACTAAACTCATATCTCGTTTATTATTAATAT
Mazhamai        TTAGTAAAGATGATTCTGCAGTGGTTTACCTGGTACGACTAAACTCATATCTCGTTTATTATTAATAT
Xiaofoshou      TTAGTAAAGATGATTCTGCAGTGGTTTACCTGGTACGACTAAACTCATATCTCGTTTATTATTAATAT
Aikang 58       TTAGTAAAGATGATTCTGCAGTGGTTTACCTGGTACGACTAAACTCATATCTCGTTTATTATTAATAT
AL8/78          --------------------------------------------------------------------
PI 431602       --------------------------------------------------------------------
PI 486274       --------------------------------------------------------------------
RM 000182       TTAGTAAAGATGATTCTGCAGTGGTTTACCTGGTACGACTAAACTCATATCTCGTTTATTATTAATAT
PI 511381       TTAGTAAAGATGATTCTGCAGTGGTTTACCTGGTACGACTAAACTCATATCTCGTTTATTATTAATAT
PI 486271       TTAGTAAAGATGATTCTGCAGTGGTTTACCTGGTACGACTAAACTCATATCTCGTTTATTATTAATAT
PI 511367       TTAGTAAAGATGATTCTGCAGTGGTTTACCTGGTACGACTAAACTCATATCTCGTTTATTATTAATAT
Chinese Spring  TTAGTAAAGATGATTCTGCAGTGGTTTACCTGGTACGACTAAACTCATATCTCGTTTATTATTAATAT
Hanzhongbai     TTAGTAAAGATGATTCTGCAGTGGTTTACCTGGTACGACTAAACTCATATCTCGTTTATTATTAATAT
Hongtoumai      TTAGTAAAGATGATTCTGCAGTGGTTTACCTGGTACGACTAAACTCATATCTCGTTTATTATTAATAT
Hongyouzi       TTAGTAAAGATGATTCTGCAGTGGTTTACCTGGTACGACTAAACTCATATCTCGTTTATTATTAATAT
Zijiehong       TTAGTAAAGATGATTCTGCAGTGGTTTACCTGGTACGACTAAACTCATATCTCGTTTATTATTAATAT
Baihuamai       TTAGTAAAGATGATTCTGCAGTGGTTTACCTGGTACGACTAAACTCATATCTCGTTTATTATTAATAT
Huixianhong     TTAGTAAAGATGATTCTGCAGTGGTTTACCTGGTACGACTAAACTCATATCTCGTTTATTATTAATAT
Baimangmai      TTAGTAAAGATGATTCTGCAGTGGTTTACCTGGTACGACTAAACTCATATCTCGTTTATTATTAATAT

                  210       220       230       240       250       260       270
Hulutou         AAGCAAATCCTATCTCGTTAATTAGTACTCTGCTTATGTGGTCGAATATATATGTAGGGGGTGCTTCC
Baihulu         AAGCAAATCCTATCTCGTTAATTAGTACTCTGCTTATGTGGTCGAATATATATGTAGGGGGTGCTTCC
Chiyacao        AAGCAAATCCTATCTCGTTAATTAGTACTCTGCTTATGTGGTCGAATATATATGTAGGGGGTGCTTCC
Hongmangmai     AAGCAAATCCTATCTCGTTAATTAGTACTCTGCTTATGTGGTCGAATATATATGTAGGGGGTGCTTCC
Hongmai         AAGCAAATCCTATCTCGTTAATTAGTACTCTGCTTATGTGGTCGAATATATATGTAGGGGGTGCTTCC
Wangshuibai     AAGCAAATCCTATCTCGTTAATTAGTACTCTGCTTATGTGGTCGAATATATATGTAGGGGGTGCTTCC
Shi 4185        AAGCAAATCCTATCTCGTTAATTAGTACTCTGCTTATGTGGTCGAATATATATGTAGGGGGTGCTTCC
Mazhamai        AAGCAAATCCTATCTCGTTAATTAGTACTCTGCTTATGTGGTCGAATATATATGTAGGGGGTGCTTCC
Xiaofoshou      AAGCAAATCCTATCTCGTTAATTAGTACTCTGCTTATGTGGTCGAATATATATGTAGGGGGTGCTTCC
Aikang 58       AAGCAAATCCTATCTCGTTAATTAGTACTCTGCTTATGTGGTCGAATATATATGTAGGGGGTGCTTCC
AL8/78          --------------------------------------------------------------------
PI 431602       --------------------------------------------------------------------
PI 486274       --------------------------------------------------------------------
RM 000182       AAGCAAATCCTATCTCGTTAATTAGTACTCTGCTTATGTGGTCGAATATATATGTAGGGGGTGCTTCC
PI 511381       AAGCAAATCCTATCTCGTTAATTAGTACTCTGCTTATGTGGTCGAATATATATGTAGGGGGTGCTTCC
PI 486271       AAGCAAATCCTATCTCGTTAATTAGTACTCTGCTTATGTGGTCGAATATATATGTAGGGGGTGCTTCC
PI 511367       AAGCAAATCCTATCTCGTTAATTAGTACTCTGCTTATGTGGTCGAATATATATGTAGGGGGTGCTTCC
Chinese Spring  AAGCAAATCCTATCTCGTTAATTAGTACTCTGCTTATGTGGTCGAATATATATGTAGGGGGTGCTTCC
Hanzhongbai     AAGCAAATCCTATCTCGTTAATTAGTACTCTGCTTATGTGGTCGAATATATATGTAGGGGGTGCTTCC
Hongtoumai      AAGCAAATCCTATCTCGTTAATTAGTACTCTGCTTATGTGGTCGAATATATATGTAGGGGGTGCTTCC
Hongyouzi       AAGCAAATCCTATCTCGTTAATTAGTACTCTGCTTATGTGGTCGAATATATATGTAGGGGGTGCTTCC
Zijiehong       AAGCAAATCCTATCTCGTTAATTAGTACTCTGCTTATGTGGTCGAATATATATGTAGGGGGTGCTTCC
Baihuamai       AAGCAAATCCTATCTCGTTAATTAGTACTCTGCTTATGTGGTCGAATATATATGTAGGGGGTGCTTCC
Huixianhong     AAGCAAATCCTATCTCGTTAATTAGTACTCTGCTTATGTGGTCGAATATATATGTAGGGGGTGCTTCC
Baimangmai      AAGCAAATCCTATCTCGTTAATTAGTACTCTGCTTATGTGGTCGAATATATATGTAGGGGGTGCTTCC
```

```
                          280       290       300       310       320       330       340
Hulutou          AAGTGGGTTCCGTGTTGCTGTCAAGAAGTCTCACTTTCGTTTTTGCTTGGATGATGAAGATGCATTCA
Baihulu          AAGTGGGTTCCGTGTTGCTGTCAAGAAGTCTCACTTTCGTTTTTGCTTGGATGATGAAGATGCATTCA
Chiyacao         AAGTGGGTTCCGTGTTGCTGTCAAGAAGTCTCACTTTCGTTTTTGCTTGGATGATGAAGATGCATTCA
Hongmangmai      AAGTGGGTTCCGTGTTGCTGTCAAGAAGTCTCACTTTCGTTTTTGCTTGGATGATGAAGATGCATTCA
Hongmai          AAGTGGGTTCCGTGTTGCTGTCAAGAAGTCTCACTTTCGTTTTTGCTTGGATGATGAAGATGCATTCA
Wangshuibai      AAGTGGGTTCCGTGTTGCTGTCAAGAAGTCTCACTTTCGTTTTTGCTTGGATGATGAAGATGCATTCA
Shi 4185         AAGTGGGTTCCGTGTTGCTGTCAAGAAGTCTCACTTTCGTTTTTGCTTGGATGATGAAGATGCATTCA
Mazhamai         AAGTGGGTTCCGTGTTGCTGTCAAGAAGTCTCACTTTCGTTTTTGCTTGGATGATGAAGATGCATTCA
Xiaofoshou       AAGTGGGTTCCGTGTTGCTGTCAAGAAGTCTCACTTTCGTTTTTGCTTGGATGATGAAGATGCATTCA
Aikang 58        AAGTGGGTTCCGTGTTGCTGTCAAGAAGTCTCACTTTCGTTTTTGCTTGGATGATGAAGATGCATTCA
AL8/78           --------------------------------------------------------------------
PI 431602        --------------------------------------------------------------------
PI 486274        --------------------------------------------------------------------
RM 000182        AAGTGGGTTCCGTGTTGCTGTCAAGAAGCTTCACAATCGTCATTGGTTAGAAGATGAAGATGCATTTA
PI 511381        AAGTGGGTTCCGTGTTGCTGTCAAGAAGCTTCACAATCGTCATTGGTTAGAAGATGAAGATGCATTTA
PI 486271        AAGTGGGTTCCGTGTTGCTGTCAAGAAGCTTCACAATCGTCATTGGTTAGAAGATGAAGATGCATTTA
PI 511367        AAGTGGGTTCCGTGTTGCTGTCAAGAAGCTTCACAATCGTCATTGGTTAGAAGATGAAGATGCATTTA
Chinese Spring   AAGTGGGTTCCGTGTTGCTGTCAAGAAGCTTCACAATCGTCATTGGTTAGAAGATGAAGATGCATTTA
Hanzhongbai      AAGTGGGTTCCGTGTTGCTGTCAAGAAGCTTCACAATCGTCATTGGTTAGAAGATGAAGATGCATTTA
Hongtoumai       AAGTGGGTTCCGTGTTGCTGTCAAGAAGCTTCACAATCGTCATTGGTTAGAAGATGAAGATGCATTTA
Hongyouzi        AAGTGGGTTCCGTGTTGCTGTCAAGAAGCTTCACAATCGTCATTGGTTAGAAGATGAAGATGCATTTA
Zijiehong        AAGTGGGTTCCGTGTTGCTGTCAAGAAGCTTCACAATCGTCATTGGTTAGAAGATGAAGATGCATTTA
Baihuamai        AAGTGGGTTCCGTGTTGCTGTCAAGAAGCTTCACAATCGTCATTGGTTAGAAGATGAAGATGCATTTA
Huixianhong      AAGTGGGTTCCGTGTTGCTGTCAAGAAGCTTCACAATCGTCATTGGTTAGAAGATGAAGATGCATTTA
Baimangmai       AAGTGGGTTCCGTGTTGCTGTCAAGAAGCTTCACAATCGTCATTGGTTAGAAGATGAAGATGCATTTA

                            350       360       370       380       390       400
Hulutou          CAAATGAAGTTTCTATTGCAATGAAGGCTGCTCATAAGAACACAGTGCGAGTCATAGGCTACTGTCAT
Baihulu          CAAATGAAGTTTCTATTGCAATGAAGGCTGCTCATAAGAACACAGTGCGAGTCATAGGCTACTGTCAT
Chiyacao         CAAATGAAGTTTCTATTGCAATGAAGGCTGCTCATAAGAACACAGTGCGAGTCATAGGCTACTGTCAT
Hongmangmai      CAAATGAAGTTTCTATTGCAATGAAGGCTGCTCATAAGAACACAGTGCGAGTCATAGGCTACTGTCAT
Hongmai          CAAATGAAGTTTCTATTGCAATGAAGGCTGCTCATAAGAACACAGTGCGAGTCATAGGCTACTGTCAT
Wangshuibai      CAAATGAAGTTTCTATTGCAATGAAGGCTGCTCATAAGAACACAGTGCGAGTCATAGGCTACTGTCAT
Shi 4185         CAAATGAAGTTTCTATTGCAATGAAGGCTGCTCATAAGAACACAGTGCGAGTCATAGGCTACTGTCAT
Mazhamai         CAAATGAAGTTTCTATTGCAATGAAGGCTGCTCATAAGAACACAGTGCGAGTCATAGGCTACTGTCAT
Xiaofoshou       CAAATGAAGTTTCTATTGCAATGAAGGCTGCTCATAAGAACACAGTGCGAGTCATAGGCTACTGTCAT
Aikang 58        CAAATGAAGTTTCTATTGCAATGAAGGCTGCTCATAAGAACACAGTGCGAGTCATAGGCTACTGTCAT
AL8/78           --------------------------------------------------------------------
PI 431602        --------------------------------------------------------------------
PI 486274        --------------------------------------------------------------------
RM 000182        TAAATGAAGTTTCTATCGCAATGAAGGCTGCTAAGAAGAACACACTGCGAGTTATAGGCTACTGTCAG
PI 511381        TAAATGAAGTTTCTATCGCAATGAAGGCTGCTAAGAAGAACACACTGCGAGTTATAGGCTACTGTCAG
PI 486271        TAAATGAAGTTTCTATCGCAATGAAGGCTGCTAAGAAGAACACACTGCGAGTTATAGGCTACTGTCAG
PI 511367        TAAATGAAGTTTCTATCGCAATGAAGGCTGCTAAGAAGAACACACTGCGAGTTATAGGCTACTGTCAG
Chinese Spring   TAAATGAAGTTTCTATCGCAATGAAGGCTGCTAAGAAGAACACAGTGCGAGTTATAGGCTACTGTCAG
Hanzhongbai      TAAATGAAGTTTCTATCGCAATGAAGGCTGCTAAGAAGAACACAGTGCGAGTTATAGGCTACTGTCAG
Hongtoumai       TAAATGAAGTTTCTATCGCAATGAAGGCTGCTAAGAAGAACACAGTGCGAGTTATAGGCTACTGTCAG
Hongyouzi        TAAATGAAGTTTCTATCGCAATGAAGGCTGCTAAGAAGAACACAGTGCGAGTTATAGGCTACTGTCAG
Zijiehong        TAAATGAAGTTTCTATCGCAATGAAGGCTGCTAAGAAGAACACAGTGCGAGTTATAGGCTACTGTCAG
Baihuamai        TAAATGAAGTTTCTATCGCAATGAAGGCTGCTAAGAAGAACACAGTGCGAGTTATAGGCTACTGTCAG
Huixianhong      TAAATGAAGTTTCTATCGCAATGAAGGCTGCTAAGAAGAACACAGTGCGAGTTATAGGCTACTGTCAG
Baimangmai       TAAATGAAGTTTCTATCGCAATGAAGGCTGCTAAGAAGAACACAGTGCGAGTTATAGGCTACTGTCAG
```

```
                 410       420       430       440       450       460       470
Hulutou          CACACGCATGAGCAAATTGCCGAATACGAAGGAAAACAAGTTTTCGCAGAGGTCAGAGAAAGGTTGAT
Baihulu          CACACGCATGAGCAAATTGCCGAATACGAAGGAAAACAAGTTTTCGCAGAGGTCAGAGAAAGGTTGAT
Chiyacao         CACACGCATGAGCAAATTGCCGAATACGAAGGAAAACAAGTTTTCGCAGAGGTCAGAGAAAGGTTGAT
Hongmangmai      CACACGCATGAGCAAATTGCCGAATACGAAGGAAAACAAGTTTTCGCAGAGGTCAGAGAAAGGTTGAT
Hongmai          CACACGCATGAGCAAATTGCCGAATACGAAGGAAAACAAGTTTTCGCAGAGGTCAGAGAAAGGTTGAT
Wangshuibai      CACACGCATGAGCAAATTGCCGAATACGAAGGAAAACAAGTTTTCGCAGAGGTCAGAGAAAGGTTGAT
Shi 4185         CACACGCATGAGCAAATTGCCGAATACGAAGGAAAACAAGTTTTCGCAGAGGTCAGAGAAAGGTTGAT
Mazhamai         CACACGCATGAGCAAATTGCCGAATACGAAGGAAAACAAGTTTTCGCAGAGGTCAGAGAAAGGTTGAT
Xiaofoshou       CACACGCATGAGCAAATTGCCGAATACGAAGGAAAACAAGTTTTCGCAGAGGTCAGAGAAAGGTTGAT
Aikang 58        CACACGCATGAGCAAATTGCCGAATACGAAGGAAAACAAGTTTTCGCAGAGGTCAGAGAAAGGTTGAT
AL8/78           --------------------------------------------------------------------
PI 431602        --------------------------------------------------------------------
PI 486274        --------------------------------------------------------------------
RM 000182        CACACGCAAGAGCAAATTGCCGAATACGAAGGAAAACAAGTTTTCGCAGGGGTCAGAGAAAGGTTGAT
PI 511381        CACACGCAAGAGCAAATTGCCGAATACGAAGGAAAACAAGTTTTCGCAGGGGTCAGAGAAAGGTTGAT
PI 486271        CACACGCAAGAGCAAATTGCCGAATACGAAGGAAAACAAGTTTTCGCAGGGGTCAGAGAAAGGTTGAT
PI 511367        CACACGCAAGAGCAAATTGCCGAATACGAAGGAAAACAAGTTTTCGCAGGGGTCAGAGAAAGGTTGAT
Chinese Spring   CACACGCAAGAGCAAATTGCCGAATACGAAGGAAAACAAGTTTTCGCCGAGCTAACAGAAAGGTTGAT
Hanzhongbai      CACACGCAAGAGCAAATTGCCGAATACGAAGGAAAACAAGTTTTCGCCGAGCTAACAGAAAGGTTGAT
Hongtoumai       CACACGCAAGAGCAAATTGCCGAATACGAAGGAAAACAAGTTTTCGCCGAGCTAACAGAAAGGTTGAT
Hongyouzi        CACACGCAAGAGCAAATTGCCGAATACGAAGGAAAACAAGTTTTCGCCGAGCTAACAGAAAGGTTGAT
Zijiehong        CACACGCAAGAGCAAATTGCCGAATACGAAGGAAAACAAGTTTTCGCCGAGCTAACAGAAAGGTTGAT
Baihuamai        CACACGCAAGAGCAAATTGCCGAATACGAAGGAAAACAAGTTTTCGCCGAGCTAACAGAAAGGTTGAT
Huixianhong      CACACGCAAGAGCAAATTGCCGAATACGAAGGAAAACAAGTTTTCGCCGAGCTAACAGAAAGGTTGAT
Baimangmai       CACACGCAAGAGCAAATTGCCGAATACGAAGGAAAACAAGTTTTCGCCGAGCTAACAGAAAGGTTGAT
                    480       490       500       510       520       530       540
Hulutou          CTGTACCGAGTATGTGCCTAACGGACCCCTTAGTGGACATATCGAAGGTAAGATATGTGCGCAAATGG
Baihulu          CTGTACCGAGTATGTGCCTAACGGACCCCTTAGTGGACATATCGAAGGTAAGATATGTGCGCAAATGG
Chiyacao         CTGTACCGAGTATGTGCCTAACGGACCCCTTAGTGGACATATCGAAGGTAAGATATGTGCGCAAATGG
Hongmangmai      CTGTACCGAGTATGTGCCTAACGGACCCCTTAGTGGACATATCGAAGGTAAGATATGTGCGCAAATGG
Hongmai          CTGTACCGAGTATGTGCCTAACGGACCCCTTAGTGGACATATCGAAGGTAAGATATGTGCGCAAATGG
Wangshuibai      CTGTACCGAGTATGTGCCTAACGGACCCCTTAGTGGACATATCGAAGGTAAGATATGTGCGCAAATGG
Shi 4185         CTGTACCGAGTATGTGCCTAACGGACCCCTTAGTGGACATATCGAAGGTAAGATATGTGCGCAAATGG
Mazhamai         CTGTACCGAGTATGTGCCTAACGGACCCCTTAGTGGACATATCGAAGGTAAGATATGTGCGCAAATGG
Xiaofoshou       CTGTACCGAGTATGTGCCTAACGGACCCCTTAGTGGACATATCGAAGGTAAGATATGTGCGCAAATGG
Aikang 58        CTGTACCGAGTATGTGCCTAACGGACCCCTTAGTGGACATATCGAAGGTAAGATATGTGCGCAAATGG
AL8/78           --------------------------------------------------------------------
PI 431602        --------------------------------------------------------------------
PI 486274        --------------------------------------------------------------------
RM 000182        CTGTACCGAGTATGTGCCTAACGGACCCCTTAGTGGACATATCGAAGGTAAGATATGTGCGCAAATGG
PI 511381        CTGTACCGAGTATGTGCCTAACGGACCCCTTAGTGGACATATCGAAGGTAAGATATGTGCGCAAATGG
PI 486271        CTGTACCGAGTATGTGCCTAACGGACCCCTTAGTGGACATATCGAAGGTAAGATATGTGCGCAAATGG
PI 511367        CTGTACCGAGTATGTGCCTAACGGACCCCTTAGTGGACATATCGAAGGTAAGATATGTGCGCAAATGG
Chinese Spring   CTGTACCGAGTATGTGCCTAACGGACCCCTTAGTGGACATATCGAAGGTAAGATATGTGCGCAAATGG
Hanzhongbai      CTGTACCGAGTATGTGCCTAACGGACCCCTTAGTGGACATATCGAAGGTAAGATATGTGCGCAAATGG
Hongtoumai       CTGTACCGAGTATGTGCCTAACGGACCCCTTAGTGGACATATCGAAGGTAAGATATGTGCGCAAATGG
Hongyouzi        CTGTACCGAGTATGTGCCTAACGGACCCCTTAGTGGACATATCGAAGGTAAGATATGTGCGCAAATGG
Zijiehong        CTGTACCGAGTATGTGCCTAACGGACCCCTTAGTGGACATATCGAAGGTAAGATATGTGCGCAAATGG
Baihuamai        CTGTACCGAGTATGTGCCTAACGGACCCCTTAGTGGACATATCGAAGGTAAGATATGTGCGCAAATGG
Huixianhong      CTGTACCGAGTATGTGCCTAACGGACCCCTTAGTGGACATATCGAAGGTAAGATATGTGCGCAAATGG
Baimangmai       CTGTACCGAGTATGTGCCTAACGGACCCCTTAGTGGACATATCGAAGGTAAGATATGTGCGCAAATGG
```

```
                                550       560       570       580       590       600       610
Hulutou          ATGGATACGAGTTCCAGAGGGCAGAACTAGATGCACTAGAACGCGTCGTACGCGATACAAGTGCGGAG
Baihulu          ATGGATACGAGTTCCAGAGGGCAGAACTAGATGCACTAGAACGCGTCGTACGCGATACAAGTGCGGAG
Chiyacao         ATGGATACGAGTTCCAGAGGGCAGAACTAGATGCACTAGAACGCGTCGTACGCGATACAAGTGCGGAG
Hongmangmai      ATGGATACGAGTTCCAGAGGGCAGAACTAGATGCACTAGAACGCGTCGTACGCGATACAAGTGCGGAG
Hongmai          ATGGATACGAGTTCCAGAGGGCAGAACTAGATGCACTAGAACGCGTCGTACGCGATACAAGTGCGGAG
Wangshuibai      ATGGATACGAGTTCCAGAGGGCAGAACTAGATGCACTAGAACGCGTCGTACGCGATACAAGTGCGGAG
Shi 4185         ATGGATACGAGTTCCAGAGGGCAGAACTAGATGCACTAGAACGCGTCGTACGCGATACAAGTGCGGAG
Mazhamai         ATGGATACGAGTTCCAGAGGGCAGAACTAGATGCACTAGAACGCGTCGTACGCGATACAAGTGCGGAG
Xiaofoshou       ATGGATACGAGTTCCAGAGGGCAGAACTAGATGCACTAGAACGCGTCGTACGCGATACAAGTGCGGAG
Aikang 58        ATGGATACGAGTTCCAGAGGGCAGAACTAGATGCACTAGAACGCGTCGTACGCGATACAAGTGCGGAG
AL8/78           ---------------------------------------------TCGTACGCGATCCAACTGCGGAG
PI 431602        ---------------------------------------------TCGTACGCGATCCAACTGCGGAG
PI 486274        ---------------------------------------------TCGTACGCGATCCAACTGCGGAG
RM 000182        ATGGATACGAGTTCCAGAGGGCAGAACTAGATGCACTAGAACGCGTCGTACGCGATACAAGTGCGGAG
PI 511381        ATGGATACGAGTTCCAGAGGGCAGAACTAGATGCACTAGAACGCGTCGTACGCGATACAAGTGCGGAG
PI 486271        ATGGATACGAGTTCCAGAGGGCAGAACTAGATGCACTAGAACGCGTCGTACGCGATACAAGTGCGGAG
PI 511367        ATGGATACGAGTTCCAGAGGGCAGAACTAGATGCACTAGAACGCGTCGTACGCGATACAAGTGCGGAG
Chinese Spring   ATGGATACGAGTTCCAGAGGGCAGAACTAGATGCACTAGAACGCGTCGTACGCGATACAAGTGCGGAG
Hanzhongbai      ATGGATACGAGTTCCAGAGGGCAGAACTAGATGCACTAGAACGCGTCGTACGCGATACAAGTGCGGAG
Hongtoumai       ATGGATACGAGTTCCAGAGGGCAGAACTAGATGCACTAGAACGCGTCGTACGCGATACAAGTGCGGAG
Hongyouzi        ATGGATACGAGTTCCAGAGGGCAGAACTAGATGCACTAGAACGCGTCGTACGCGATACAAGTGCGGAG
Zijiehong        ATGGATACGAGTTCCAGAGGGCAGAACTAGATGCACTAGAACGCGTCGTACGCGATACAAGTGCGGAG
Baihuamai        ATGGATACGAGTTCCAGAGGGCAGAACTAGATGCACTAGAACGCGTCGTACGCGATACAAGTGCGGAG
Huixianhong      ATGGATACGAGTTCCAGAGGGCAGAACTAGATGCACTAGAACGCGTCGTACGCGATACAAGTGCGGAG
Baimangmai       ATGGATACGAGTTCCAGAGGGCAGAACTAGATGCACTAGAACGCGTCGTACGCGATACAAGTGCGGAG

                      620       630       640       650       660       670       680
Hulutou          CCAATGAGTCTGACGTTGCCGCTTCTCAGGCACATAACAAATGATTTCTCCGATGAATCTCGAATTGG
Baihulu          CCAATGAGTCTGACGTTGCCGCTTCTCAGGCACATAACAAATGATTTCTCCGATGAATCTCGAATTGG
Chiyacao         CCAATGAGTCTGACGTTGCCGCTTCTCAGGCACATAACAAATGATTTCTCCGATGAATCTCGAATTGG
Hongmangmai      CCAATGAGTCTGACGTTGCCGCTTCTCAGGCACATAACAAATGATTTCTCCGATGAATCTCGAATTGG
Hongmai          CCAATGAGTCTGACGTTGCCGCTTCTCAGGCACATAACAAATGATTTCTCCGATGAATCTCGAATTGG
Wangshuibai      CCAATGAGTCTGACGTTGCCGCTTCTCAGGCACATAACAAATGATTTCTCCGATGAATCTCGAATTGG
Shi 4185         CCAATGAGTCTGACGTTGCCGCTTCTCAGGCACATAACAAATGATTTCTCCGATGAATCTCGAATTGG
Mazhamai         CCAATGAGTCTGACGTTGCCGCTTCTCAGGCACATAACAAATGATTTCTCCGATGAATCTCGAATTGG
Xiaofoshou       CCAATGAGTCTGACGTTGCCGCTTCTCAGGCACATAACAAATGATTTCTCCGATGAATCTCGAATTGG
Aikang 58        CCAATGAGTCTGACGTTGCCGCTTCTCAGGCACATAACAAATGATTTCTCCGATGAATCTCGAATTGG
AL8/78           CCAATGAGTCTGACGTTGCCGCTTCTCAGGCACATAACAAATGATTTCTCCCATGAATCTCGAATTGG
PI 431602        CCAATGAGTCTGACGTTGCCGCTTCTCAGGCACATAACAAATGATTTCTCCCATGAATCTCGAATTGG
PI 486274        CCAATGAGTCTGACGTTGCCGCTTCTCAGGCACATAACAAATGATTTCTCCCATGAATCTCGAATTGG
RM 000182        CCAATGAGTCTGACGTTGCCGCTTCTCAGGCACATAACAAATGATTTCTCCGATGAATCTCGAATTGG
PI 511381        CCAATGAGTCTGACGTTGCCGCTTCTCAGGCACATAACAAATGATTTCTCCGATGAATCTCGAATTGG
PI 486271        CCAATGAGTCTGACGTTGCCGCTTCTCAGGCACATAACAAATGATTTCTCCGATGAATCTCGAATTGG
PI 511367        CCAATGAGTCTGACGTTGCCGCTTCTCAGGCACATAACAAATGATTTCTCCGATGAATCTCGAATTGG
Chinese Spring   CCAATGAGTCTGACGTTGCCGCTTCTCAGGCACATAACAAATGATTTCTCCGATGAATCTCGAATTGG
Hanzhongbai      CCAATGAGTCTGACGTTGCCGCTTCTCAGGCACATAACAAATGATTTCTCCGATGAATCTCGAATTGG
Hongtoumai       CCAATGAGTCTGACGTTGCCGCTTCTCAGGCACATAACAAATGATTTCTCCGATGAATCTCGAATTGG
Hongyouzi        CCAATGAGTCTGACGTTGCCGCTTCTCAGGCACATAACAAATGATTTCTCCGATGAATCTCGAATTGG
Zijiehong        CCAATGAGTCTGACGTTGCCGCTTCTCAGGCACATAACAAATGATTTCTCCGATGAATCTCGAATTGG
Baihuamai        CCAATGAGTCTGACGTTGCCGCTTCTCAGGCACATAACAAATGATTTCTCCGATGAATCTCGAATTGG
Huixianhong      CCAATGAGTCTGACGTTGCCGCTTCTCAGGCACATAACAAATGATTTCTCCGATGAATCTCGAATTGG
Baimangmai       CCAATGAGTCTGACGTTGCCGCTTCTCAGGCACATAACAAATGATTTCTCCGATGAATCTCGAATTGG
```

	690 700 710 720 730 740
Hulutou	CCGAGGTGGATTCGCAGTGGTTTACCTGGTATGATTAAACTCATACAGTATCTCCTTAGATATTAAGC
Baihulu	CCGAGGTGGATTCGCAGTGGTTTACCTGGTATGATTAAACTCATACAGTATCTCCTTAGATATTAAGC
Chiyacao	CCGAGGTGGATTCGCAGTGGTTTACCTGGTATGATTAAACTCATACAGTATCTCCTTAGATATTAAGC
Hongmangmai	CCGAGGTGGATTCGCAGTGGTTTACCTGGTATGATTAAACTCATACAGTATCTCCTTAGATATTAAGC
Hongmai	CCGAGGTGGATTCGCAGTGGTTTACCTGGTATGATTAAACTCATACAGTATCTCCTTAGATATTAAGC
Wangshuibai	CCGAGGTGGATTCGCAGTGGTTTACCTGGTATGATTAAACTCATACAGTATCTCCTTAGATATTAAGC
Shi 4185	CCGAGGTGGATTCGCAGTGGTTTACCTGGTATGATTAAACTCATACAGTATCTCCTTAGATATTAAGC
Mazhamai	CCGAGGTGGATTCGCAGTGGTTTACCTGGTATGATTAAACTCATACAGTATCTCCTTAGATATTAAGC
Xiaofoshou	CCGAGGTGGATTCGCAGTGGTTTACCTGGTATGATTAAACTCATACAGTATCTCCTTAGATATTAAGC
Aikang 58	CCGAGGTGGATTCGCAGTGGTTTACCTGGTATGATTAAACTCATACAGTATCTCCTTAGATATTAAGC
AL8/78	CCGAGGTGGATTCGCAGTGGTTTACCTGGTATGATTAAACTCATACAGTATCTCCTTAGATATTAAGC
PI 431602	CCGAGGTGGATTCGCAGTGGTTTACCTGGTATGATTAAACTCATACAGTATCTCCTTAGATATTAAGC
PI 486274	CCGAGGTGGATTCGCAGTGGTTTACCTGGTATGATTAAACTCATACAGTATCTCCTTAGATATTAAGC
RM 000182	CCGAGGTGGATTCGCAGTGGTTTACCTGGTATGATTAAACTCATACAGTATCTCCTTAGATATTAAGC
PI 511381	CCGAGGTGGATTCGCAGTGGTTTACCTGGTATGATTAAACTCATACAGTATCTCCTTAGATATTAAGC
PI 486271	CCGAGGTGGATTCGCAGTGGTTTACCTGGTATGATTAAACTCATACAGTATCTCCTTAGATATTAAGC
PI 511367	CCGAGGTGGATTCGCAGTGGTTTACCTGGTATGATTAAACTCATACAGTATCTCCTTAGATATTAAGC
Chinese Spring	CCGAGGTGGATTCGCAGTGGTTTACCTGGTATGATTAAACTCATACAGTATCTCCTTAGATATTAAGC
Hanzhongbai	CCGAGGTGGATTCGCAGTGGTTTACCTGGTATGATTAAACTCATACAGTATCTCCTTAGATATTAAGC
Hongtoumai	CCGAGGTGGATTCGCAGTGGTTTACCTGGTATGATTAAACTCATACAGTATCTCCTTAGATATTAAGC
Hongyouzi	CCGAGGTGGATTCGCAGTGGTTTACCTGGTATGATTAAACTCATACAGTATCTCCTTAGATATTAAGC
Zijiehong	CCGAGGTGGATTCGCAGTGGTTTACCTGGTATGATTAAACTCATACAGTATCTCCTTAGATATTAAGC
Baihuamai	CCGAGGTGGATTCGCAGTGGTTTACCTGGTATGATTAAACTCATACAGTATCTCCTTAGATATTAAGC
Huixianhong	CCGAGGTGGATTCGCAGTGGTTTACCTGGTATGATTAAACTCATACAGTATCTCCTTAGATATTAAGC
Baimangmai	CCGAGGTGGATTCGCAGTGGTTTACCTGGTATGATTAAACTCATACAGTATCTCCTTAGATATTAAGC

	750 760 770 780 790 800 810
Hulutou	AATTCCTATCTTGTCAATTACTAACTTCTTATTGTGGTCAAATATACAGGGGGTGCTTCCAAGTGGGT
Baihulu	AATTCCTATCTTGTCAATTACTAACTTCTTATTGTGGTCAAATATACAGGGGGTGCTTCCAAGTGGGT
Chiyacao	AATTCCTATCTTGTCAATTACTAACTTCTTATTGTGGTCAAATATACAGGGGGTGCTTCCAAGTGGGT
Hongmangmai	AATTCCTATCTTGTCAATTACTAACTTCTTATTGTGGTCAAATATACAGGGGGTGCTTCCAAGTGGGT
Hongmai	AATTCCTATCTTGTCAATTACTAACTTCTTATTGTGGTCAAATATACAGGGGGTGCTTCCAAGTGGGT
Wangshuibai	AATTCCTATCTTGTCAATTACTAACTTCTTATTGTGGTCAAATATACAGGGGGTGCTTCCAAGTGGGT
Shi 4185	AATTCCTATCTTGTCAATTACTAACTTCTTATTGTGGTCAAATATACAGGGGGTGCTTCCAAGTGGGT
Mazhamai	AATTCCTATCTTGTCAATTACTAACTTCTTATTGTGGTCAAATATACAGGGGGTGCTTCCAAGTGGGT
Xiaofoshou	AATTCCTATCTTGTCAATTACTAACTTCTTATTGTGGTCAAATATACAGGGGGTGCTTCCAAGTGGGT
Aikang 58	AATTCCTATCTTGTCAATTACTAACTTCTTATTGTGGTCAAATATACAGGGGGTGCTTCCAAGTGGGT
AL8/78	AATTCCTATCTTGTCAATTACTAACTTCTTATTGTGGTCAAATATACAGGGGGTGCTTCCAAGTGGGT
PI 431602	AATTCCTATCTTGTCAATTACTAACTTCTTATTGTGGTCAAATATACAGGGGGTGCTTCCAAGTGGGT
PI 486274	AATTCCTATCTTGTCAATTACTAACTTCTTATTGTGGTCAAATATACAGGGGGTGCTTCCAAGTGGGT
RM 000182	AATTCCTATCTTGTCAATTACTAACTTCTTATTGTGGTCAAATATACAGGGGGTGCTTCCAAGTGGGT
PI 511381	AATTCCTATCTTGTCAATTACTAACTTCTTATTGTGGTCAAATATACAGGGGGTGCTTCCAAGTGGGT
PI 486271	AATTCCTATCTTGTCAATTACTAACTTCTTATTGTGGTCAAATATACAGGGGGTGCTTCCAAGTGGGT
PI 511367	AATTCCTATCTTGTCAATTACTAACTTCTTATTGTGGTCAAATATACAGGGGGTGCTTCCAAGTGGGT
Chinese Spring	AATTCCTATCTTGTCAATTACTAACTTCTTATTGTGGTCAAATATACAGGGGGTGCTTCCAAGTGGGT
Hanzhongbai	AATTCCTATCTTGTCAATTACTAACTTCTTATTGTGGTCAAATATACAGGGGGTGCTTCCAAGTGGGT
Hongtoumai	AATTCCTATCTTGTCAATTACTAACTTCTTATTGTGGTCAAATATACAGGGGGTGCTTCCAAGTGGGT
Hongyouzi	AATTCCTATCTTGTCAATTACTAACTTCTTATTGTGGTCAAATATACAGGGGGTGCTTCCAAGTGGGT
Zijiehong	AATTCCTATCTTGTCAATTACTAACTTCTTATTGTGGTCAAATATACAGGGGGTGCTTCCAAGTGGGT
Baihuamai	AATTCCTATCTTGTCAATTACTAACTTCTTATTGTGGTCAAATATACAGGGGGTGCTTCCAAGTGGGT
Huixianhong	AATTCCTATCTTGTCAATTACTAACTTCTTATTGTGGTCAAATATACAGGGGGTGCTTCCAAGTGGGT
Baimangmai	AATTCCTATCTTGTCAATTACTAACTTCTTATTGTGGTCAAATATACAGGGGGTGCTTCCAAGTGGGT

	820 830 840 850 860 870 880
Hulutou	TACGTATTGCTGTTAAGAGGCTTAGCAATATTGCTTATATGAACGAAAGTGCATTTCAAAATGAAGTG
Baihulu	TACGTATTGCTGTTAAGAGGCTTAGCAATATTGCTTATATGAACGAAAGTGCATTTCAAAATGAAGTG
Chiyacao	TACGTATTGCTGTTAAGAGGCTTAGCAATATTGCTTATATGAACGAAAGTGCATTTCAAAATGAAGTG
Hongmangmai	TACGTATTGCTGTTAAGAGGCTTAGCAATATTGCTTATATGAACGAAAGTGCATTTCAAAATGAAGTG
Hongmai	TACGTATTGCTGTTAAGAGGCTTAGCAATATTGCTTATATGAACGAAAGTGCATTTCAAAATGAAGTG
Wangshuibai	TACGTATTGCTGTTAAGAGGCTTAGCAATATTGCTTATATGAACGAAAGTGCATTTCAAAATGAAGTG
Shi 4185	TACGTATTGCTGTTAAGAGGCTTAGCAATATTGCTTATATGAACGAAAGTGCATTTCAAAATGAAGTG
Mazhamai	TACGTATTGCTGTTAAGAGGCTTAGCAATATTGCTTATATGAACGAAAGTGCATTTCAAAATGAAGTG
Xiaofoshou	TACGTATTGCTGTTAAGAGGCTTAGCAATATTGCTTATATGAACGAAAGTGCATTTCAAAATGAAGTG
Aikang 58	TACGTATTGCTGTTAAGAGGCTTAGCAATATTGCTTATATGAACGAAAGTGCATTTCAAAATGAAGTG
AL8/78	TACGTATTGCTGTTAAGAGGCTTAGCAATATTGCTTATATGAATGAAAGTGCATTTCAAAATGAAGTG
PI 431602	TACGTATTGCTGTTAAGAGGCTTAGCAATATTGCTTATATGAATGAAAGTGCATTTCAAAATGAAGTG
PI 486274	TACGTATTGCTGTTAAGAGGCTTAGCAATATTGCTTATATGAATGAAAGTGCATTTCAAAATGAAGTG
RM 000182	TACGTATTGCTGTTAAGAGGCTTAGCAATATTGCTTATATGAACGAAAGTGCATTTCAAAATGAAGTG
PI 511381	TACGTATTGCTGTTAAGAGGCTTAGCAATATTGCTTATATGAACGAAAGTGCATTTCAAAATGAAGTG
PI 486271	TACGTATTGCTGTTAAGAGGCTTAGCAATATTGCTTATATGAACGAAAGTGCATTTCAAAATGAAGTG
PI 511367	TACGTATTGCTGTTAAGAGGCTTAGCAATATTGCTTATATGAACGAAAGTGCATTTCAAAATGAAGTG
Chinese Spring	TACGTATTGCTGTTAAGAGGCTTAGCAATATTGCTTATATGAATGAAAGTGCATTTCAAAATGAAGTG
Hanzhongbai	TACGTATTGCTGTTAAGAGGCTTAGCAATATTGCTTATATGAATGAAAGTGCATTTCAAAATGAAGTG
Hongtoumai	TACGTATTGCTGTTAAGAGGCTTAGCAATATTGCTTATATGAATGAAAGTGCATTTCAAAATGAAGTG
Hongyouzi	TACGTATTGCTGTTAAGAGGCTTAGCAATATTGCTTATATGAATGAAAGTGCATTTCAAAATGAAGTG
Zijiehong	TACGTATTGCTGTTAAGAGGCTTAGCAATATTGCTTATATGAATGAAAGTGCATTTCAAAATGAAGTG
Baihuamai	TACGTATTGCTGTTAAGAGGCTTAGCAATATTGCTTATATGAATGAAAGTGCATTTCAAAATGAAGTG
Huixianhong	TACGTATTGCTGTTAAGAGGCTTAGCAATATTGCTTATATGAATGAAAGTGCATTTCAAAATGAAGTG
Baimangmai	TACGTATTGCTGTTAAGAGGCTTAGCAATATTGCTTATATGAATGAAAGTGCATTTCAAAATGAAGTG

	890 900 910 920 930 940 950
Hulutou	TTTATCACAATGAAGGCCACTCACAAGAACACAGTGCGATTCATGGGCTACTGTAGTCAAATACAAGG
Baihulu	TTTATCACAATGAAGGCCACTCACAAGAACACAGTGCGATTCATGGGCTACTGTAGTCAAATACAAGG
Chiyacao	TTTATCACAATGAAGGCCACTCACAAGAACACAGTGCGATTCATGGGCTACTGTAGTCAAATACAAGG
Hongmangmai	TTTATCACAATGAAGGCCACTCACAAGAACACAGTGCGATTCATGGGCTACTGTAGTCAAATACAAGG
Hongmai	TTTATCACAATGAAGGCCACTCACAAGAACACAGTGCGATTCATGGGCTACTGTAGTCAAATACAAGG
Wangshuibai	TTTATCACAATGAAGGCCACTCACAAGAACACAGTGCGATTCATGGGCTACTGTAGTCAAATACAAGG
Shi 4185	TTTATCACAATGAAGGCCACTCACAAGAACACAGTGCGATTCATGGGCTACTGTAGTCAAATACAAGG
Mazhamai	TTTATCACAATGAAGGCCACTCACAAGAACACAGTGCGATTCATGGGCTACTGTAGTCAAATACAAGG
Xiaofoshou	TTTATCACAATGAAGGCCACTCACAAGAACACAGTGCGATTCATGGGCTACTGTAGTCAAATACAAGG
Aikang 58	TTTATCACAATGAAGGCCACTCACAAGAACACAGTGCGATTCATGGGCTACTGTAGTCAAATACAAGG
AL8/78	TTTATCACAATGAAGGCCACTCACAAGAACACAGTGCGATTCATGGGCTACTGTAGTCAAATACAACG
PI 431602	TTTATCACAATGAAGGCCACTCACAAGAACACAGTGCGATTCATGGGCTACTGTAGTCAAATACAACG
PI 486274	TTTATCACAATGAAGGCCACTCACAAGAACACAGTGCGATTCATGGGCTACTGTAGTCAAATACAACG
RM 000182	TTTATCACAATGAAGGCCACTCACAAGAACACAGTGCGATTCATGGGCTACTGTAGTCAAATACAAGG
PI 511381	TTTATCACAATGAAGGCCACTCACAAGAACACAGTGCGATTCATGGGCTACTGTAGTCAAATACAAGG
PI 486271	TTTATCACAATGAAGGCCACTCACAAGAACACAGTGCGATTCATGGGCTACTGTAGTCAAATACAAGG
PI 511367	TTTATCACAATGAAGGCCACTCACAAGAACACAGTGCGATTCATGGGCTACTGTAGTCAAATACAAGG
Chinese Spring	TTTATCACAATGAAGGCCACTCACAAGAACACAGTGCGATTCATGGGCTACTGTAGTCAAATACAAGG
Hanzhongbai	TTTATCACAATGAAGGCCACTCACAAGAACACAGTGCGATTCATGGGCTACTGTAGTCAAATACAAGG
Hongtoumai	TTTATCACAATGAAGGCCACTCACAAGAACACAGTGCGATTCATGGGCTACTGTAGTCAAATACAAGG
Hongyouzi	TTTATCACAATGAAGGCCACTCACAAGAACACAGTGCGATTCATGGGCTACTGTAGTCAAATACAAGG
Zijiehong	TTTATCACAATGAAGGCCACTCACAAGAACACAGTGCGATTCATGGGCTACTGTAGTCAAATACAAGG
Baihuamai	TTTATCACAATGAAGGCCACTCACAAGAACACAGTGCGATTCATGGGCTACTGTAGTCAAATACAAGG
Huixianhong	TTTATCACAATGAAGGCCACTCACAAGAACACAGTGCGATTCATGGGCTACTGTAGTCAAATACAAGG
Baimangmai	TTTATCACAATGAAGGCCACTCACAAGAACACAGTGCGATTCATGGGCTACTGTAGTCAAATACAAGG

	960 · 970 · 980 · 990 · 1,000 · 1,010 · 1,020
Hulutou	TAAACTCATCGAACACGACGGGCAACATGTTTTCGCACAGCTCGAGGAAAGGTTGATCTGTGTGGAAT
Baihulu	TAAACTCATCGAACACGACGGGCAACATGTTTTCGCACAGCTCGAGGAAAGGTTGATCTGTGTGGAAT
Chiyacao	TAAACTCATCGAACACGACGGGCAACATGTTTTCGCACAGCTCGAGGAAAGGTTGATCTGTGTGGAAT
Hongmangmai	TAAACTCATCGAACACGACGGGCAACATGTTTTCGCACAGCTCGAGGAAAGGTTGATCTGTGTGGAAT
Hongmai	TAAACTCATCGAACACGACGGGCAACATGTTTTCGCACAGCTCGAGGAAAGGTTGATCTGTGTGGAAT
Wangshuibai	TAAACTCATCGAACACGACGGGCAACATGTTTTCGCACAGCTCGAGGAAAGGTTGATCTGTGTGGAAT
Shi 4185	TAAACTCATCGAACACGACGGGCAACATGTTTTCGCACAGCTCGAGGAAAGGTTGATCTGTGTGGAAT
Mazhamai	TAAACTCATCGAACACGACGGGCAACATGTTTTCGCACAGCTCGAGGAAAGGTTGATCTGTGTGGAAT
Xiaofoshou	TAAACTCATCGAACACGACGGGCAACATGTTTTCGCACAGCTCGAGGAAAGGTTGATCTGTGTGGAAT
Aikang 58	TAAACTCATCG------ACGGGCAACATGTTTTCGCACAGCTCGAGGAAAGGTTGATCTGTGTGGAAT
AL8/78	CCAATTCATCAAACACCACGGGCGACATGTTTACGCACAGCTCGTGAAAAGGTTGATCTTTGTGGAAT
PI 431602	CCAATTCATCAAACACCACGGGCGACATGTTTACGCACAGCTCGTGAAAAGGTTGATCTTTGTGGAAT
PI 486274	CCAATTCATCAAACACCACGGGCGACATGTTTACGCACAGCTCGTGAAAAGGTTGATCTTTGTGGAAT
RM 000182	TAAACTCATCGAACACAACGGGCAACATGTTTTCGCACAGCTCGTGGAAAGGTTGATCTGTGTGGAAT
PI 511381	TAAACTCATCGAACACAACGGGCAACATGTTTTCGCACAGCTCGTGGAAAGGTTGATCTGTGTGGAAT
PI 486271	TAAACTCATCGAACACAACGGGCAACATGTTTTCGCACAGCTCGTGGAAAGGTTGATCTGTGTGGAAT
PI 511367	TAAACTCATCGAACACAACGGGCAACATGTTTTCGCACAGCTCGTGGAAAGGTTGATCTGTGTGGAAT
Chinese Spring	TAAACTCATCGAACACAACGGGCAACATGTTTTCGCACAGCTCGAGGAAAGGTTGATCTGTGTGGAAT
Hanzhongbai	TAAACTCATCGAACACAACGGGCAACATGTTTTCGCACAGCTCGAGGAAAGGTTGATCTGTGTGGAAT
Hongtoumai	TAAACTCATCGAACACAACGGGCAACATGTTTTCGCACAGCTCGAGGAAAGGTTGATCTGTGTGGAAT
Hongyouzi	TAAACTCATCGAACACAACGGGCAACATGTTTTCGCACAGCTCGAGGAAAGGTTGATCTGTGTGGAAT
Zijiehong	TAAACTCATCGAACACAACGGGCAACATGTTTTCGCACAGCTCGAGGAAAGGTTGATCTGTGTGGAAT
Baihuamai	TAAACTCATCGAACACAACGGGCAACATGTTTTCGCACAGCTCGAGGAAAGGTTGATCTGTGTGGAAT
Huixianhong	TAAACTCATCGAACACAACGGGCAACATGTTTTCGCACAGCTCGAGGAAAGGTTGATCTGTGTGGAAT
Baimangmai	TAAACTCATCGAACACAACGGGCAACATGTTTTCGCACAGCTCGAGGAAAGGTTGATCTGTGTGGAAT

	1,030 · 1,040 · 1,050 · 1,060 · 1,070 · 1,080
Hulutou	ATGCGCCTAAAGGAACCCTTGATGCACATATCGGTAAGATCGATAACACGGCATGCCCGCCCTAGCAA
Baihulu	ATGCGCCTAAAGGAACCCTTGATGCACATATCGGTAAGATCGATAACACGGCATGCCCGCCCTAGCAA
Chiyacao	ATGCGCCTAAAGGAACCCTTGATGCACATATCGGTAAGATCGATAACACGGCATGCCCGCCCTAGCAA
Hongmangmai	ATGCGCCTAAAGGAACCCTTGATGCACATATCGGTAAGATCGATAACACGGCATGCCCGCCCTAGCAA
Hongmai	ATGCGCCTAAAGGAACCCTTGATGCACATATCGGTAAGATCGATAACACGGCATGCCCGCCCTAGCAA
Wangshuibai	ATGCGCCTAAAGGAACCCTTGATGCACATATCGGTAAGATCGATAACACGGCATGCCCGCCCTAGCAA
Shi 4185	ATGCGCCTAAAGGAACCCTTGATGCACATATCGGTAAGATCGATAACACGGCATGCCCGCCCTAGCAA
Mazhamai	ATGCGCCTAAAGGAACCCTTGATGCACATATCGGTAAGATCGATAACACGGCATGCCCGCCCTAGCAA
Xiaofoshou	ATGCGCCTAAAGGAACCCTTGATGCACATATCGGTAAGATCGATAACACGGCATGCCCGCCCTAGCAA
Aikang 58	ATGCGCCTAAAGGAACCCTTGATGCACATATCGGTAAGATCGATAACACGGCATGCCCGCCCTAGCAA
AL8/78	ATGCGCCTAAAGGAACCCTTGATGCACATATCGGTAAGATCGATAACACGGCATGCCCGCCCTAGCAA
PI 431602	ATGCGCCTAAAGGAACCCTTGATGCACATATCGGTAAGATCGATAACACGGCATGCCCGCCCTAGCAA
PI 486274	ATGCGCCTAAAGGAACCCTTGATGCACATATCGGTAAGATCGATAACACGGCATGCCCGCCCTAGCAA
RM 000182	ATGCGCCTAAAGGAACCCTTGATGCACATATCGGTAAGATCGATAACACGGCATGCCCGCCCTAGCAA
PI 511381	ATGCGCCTAAAGGAACCCTTGATGCACATATCGGTAAGATCGATAACACGGCATGCCCGCCCTAGCAA
PI 486271	ATGCGCCTAAAGGAACCCTTGATGCACATATCGGTAAGATCGATAACACGGCATGCCCGCCCTAGCAA
PI 511367	ATGCGCCTAAAGGAACCCTTGATGCACATATCGGTAAGATCGATAACACGGCATGCCCGCCCTAGCAA
Chinese Spring	ATGCGCCTAAAGGAACCCTTGATGCACATATCGGTAAGATCGATAACACGGCATGCCCGCCCTAGCAA
Hanzhongbai	ATGCGCCTAAAGGAACCCTTGATGCACATATCGGTAAGATCGATAACACGGCATGCCCGCCCTAGCAA
Hongtoumai	ATGCGCCTAAAGGAACCCTTGATGCACATATCGGTAAGATCGATAACACGGCATGCCCGCCCTAGCAA
Hongyouzi	ATGCGCCTAAAGGAACCCTTGATGCACATATCGGTAAGATCGATAACACGGCATGCCCGCCCTAGCAA
Zijiehong	ATGCGCCTAAAGGAACCCTTGATGCACATATCGGTAAGATCGATAACACGGCATGCCCGCCCTAGCAA
Baihuamai	ATGCGCCTAAAGGAACCCTTGATGCACATATCGGTAAGATCGATAACACGGCATGCCCGCCCTAGCAA
Huixianhong	ATGCGCCTAAAGGAACCCTTGATGCACATATCGGTAAGATCGATAACACGGCATGCCCGCCCTAGCAA
Baimangmai	ATGCGCCTAAAGGAACCCTTGATGCACATATCGGTAAGATCGATAACACGGCATGCCCGCCCTAGCAA

	1,090 1,100 1,110 1,120 1,130 1,140 1,150
Hulutou	TAATTCCCTCTCTTTATTTTGGTGTTTCTGTTTGTGTGGCTGTTTTTTACTTTGTGCTCACAGAACAA
Baihulu	TAATTCCCTCTCTTTATTTTGGTGTTTCTGTTTGTGTGGCTGTTTTTTACTTTGTGCTCACAGAACAA
Chiyacao	TAATTCCCTCTCTTTATTTTGGTGTTTCTGTTTGTGTGGCTGTTTTTTACTTTGTGCTCACAGAACAA
Hongmangmai	TAATTCCCTCTCTTTATTTTGGTGTTTCTGTTTGTGTGGCTGTTTTTTACTTTGTGCTCACAGAACAA
Hongmai	TAATTCCCTCTCTTTATTTTGGTGTTTCTGTTTGTGTGGCTGTTTTTTACTTTGTGCTCACAGAACAA
Wangshuibai	TAATTCCCTCTCTTTATTTTGGTGTTTCTGTTTGTGTGGCTGTTTTTTACTTTGTGCTCACAGAACAA
Shi 4185	TAATTCCCTCTCTTTATTTTGGTGTTTCTGTTTGTGTGGCTGTTTTTTACTTTGTGCTCACAGAACAA
Mazhamai	TAATTCCCTCTCTTTATTTTGGTGTTTCTGTTTGTGTGGCTGTTTTTTACTTTGTGCTCACAGAACAA
Xiaofoshou	TAATTCCCTCTCTTTATTTTGGTGTTTCTGTTTGTGTGGCTGTTTTTTACTTTGTGCTCACAGAACAA
Aikang 58	TAATTCCCTCTCTTTATTTTGGTGTTTCTGTTTGTGTGGCTGTTTTTTACTTTGTGCTCACAGAACAA
AL8/78	TAATTCCCTCTCTTTATTTTGGTGTTTCTGTTTGTGTGGCTGTTTTTTACTTTGTGCTCACAGAACAA
PI 431602	TAATTCCCTCTCTTTATTTTGGTGTTTCTGTTTGTGTGGCTGTTTTTTACTTTGTGCTCACAGAACAA
PI 486274	TAATTCCCTCTCTTTATTTTGGTGTTTCTGTTTGTGTGGCTGTTTTTTACTTTGTGCTCACAGAACAA
RM 000182	TAATTCCCTCTCTTTATTTTGGTGTTTCTGTTTGTGTGGCTGTTTTTTACTTTGTGCTCACAGAACAA
PI 511381	TAATTCCCTCTCTTTATTTTGGTGTTTCTGTTTGTGTGGCTGTTTTTTACTTTGTGCTCACAGAACAA
PI 486271	TAATTCCCTCTCTTTATTTTGGTGTTTCTGTTTGTGTGGCTGTTTTTTACTTTGTGCTCACAGAACAA
PI 511367	TAATTCCCTCTCTTTATTTTGGTGTTTCTGTTTGTGTGGCTGTTTTTTACTTTGTGCTCACAGAACAA
Chinese Spring	TAATTCCCTCTCTTTATTTTGGTGTTTCTGTTTGTGTGGCTGTTTTTTACTTTGTGCTCACAGAACAA
Hanzhongbai	TAATTCCCTCTCTTTATTTTGGTGTTTCTGTTTGTGTGGCTGTTTTTTACTTTGTGCTCACAGAACAA
Hongtoumai	TAATTCCCTCTCTTTATTTTGGTGTTTCTGTTTGTGTGGCTGTTTTTTACTTTGTGCTCACAGAACAA
Hongyouzi	TAATTCCCTCTCTTTATTTTGGTGTTTCTGTTTGTGTGGCTGTTTTTTACTTTGTGCTCACAGAACAA
Zijiehong	TAATTCCCTCTCTTTATTTTGGTGTTTCTGTTTGTGTGGCTGTTTTTTACTTTGTGCTCACAGAACAA
Baihuamai	TAATTCCCTCTCTTTATTTTGGTGTTTCTGTTTGTGTGGCTGTTTTTTACTTTGTGCTCACAGAACAA
Huixianhong	TAATTCCCTCTCTTTATTTTGGTGTTTCTGTTTGTGTGGCTGTTTTTTACTTTGTGCTCACAGAACAA
Baimangmai	TAATTCCCTCTCTTTATTTTGGTGTTTCTGTTTGTGTGGCTGTTTTTTACTTTGTGCTCACAGAACAA

	1,160 1,170 1,180 1,190 1,200 1,210 1,220
Hulutou	AGAATGAATACCAGTGGAACAAAAAAGTAGACTATAATTTTAGCGCAACTGAAACCAACATGACCAGT
Baihulu	AGAATGAATACCAGTGGAACAAAAAAGTAGACTATAATTTTAGCGCAACTGAAACCAACATGACCAGT
Chiyacao	AGAATGAATACCAGTGGAACAAAAAAGTAGACTATAATTTTAGCGCAACTGAAACCAACATGACCAGT
Hongmangmai	AGAATGAATACCAGTGGAACAAAAAAGTAGACTATAATTTTAGCGCAACTGAAACCAACATGACCAGT
Hongmai	AGAATGAATACCAGTGGAACAAAAAAGTAGACTATAATTTTAGCGCAACTGAAACCAACATGACCAGT
Wangshuibai	AGAATGAATACCAGTGGAACAAAAAAGTAGACTATAATTTTAGCGCAACTGAAACCAACATGACCAGT
Shi 4185	AGAATGAATACCAGTGGAACAAAAAAGTAGACTATAATTTTAGCGCAACTGAAACCAACATGACCAGT
Mazhamai	AGAATGAATACCAGTGGAACAAAAAAGTAGACTATAATTTTAGCGCAACTGAAACCAACATGACCAGT
Xiaofoshou	AGAATGAATACCAGTGGAACAAAAAAGTAGACTATAATTTTAGCGCAACTGAAACCAACATGACCAGT
Aikang 58	AGAATGAATACCAGTGGAACAAAAAAGTAGACTATAATTTTAGCGCAACTGAAACCAACATGACCAGT
AL8/78	AGAATGAATACCAGTGGAACAAAAAAGTAGACTATAATTTTAGCGCAACTGAAACCAACATGACCAGT
PI 431602	AGAATGAATACCAGTGGAACAAAAAAGTAGACTATAATTTTAGCGCAACTGAAACCAACATGACCAGT
PI 486274	AGAATGAATACCAGTGGAACAAAAAAGTAGACTATAATTTTAGCGCAACTGAAACCAACATGACCAGT
RM 000182	AGAATGAATACCAGTGGAACAAAAAAGTAGACTATAATTTTAGCGCAACTGAAACCAACATGACCAGT
PI 511381	AGAATGAATACCAGTGGAACAAAAAAGTAGACTATAATTTTAGCGCAACTGAAACCAACATGACCAGT
PI 486271	AGAATGAATACCAGTGGAACAAAAAAGTAGACTATAATTTTAGCGCAACTGAAACCAACATGACCAGT
PI 511367	AGAATGAATACCAGTGGAACAAAAAAGTAGACTATAATTTTAGCGCAACTGAAACCAACATGACCAGT
Chinese Spring	AGAATGAATACCAGTGGAACAAAAAAGTAGACTATAATTTTAGCGCAACTGAAACCAACATGACCAGT
Hanzhongbai	AGAATGAATACCAGTGGAACAAAAAAGTAGACTATAATTTTAGCGCAACTGAAACCAACATGACCAGT
Hongtoumai	AGAATGAATACCAGTGGAACAAAAAAGTAGACTATAATTTTAGCGCAACTGAAACCAACATGACCAGT
Hongyouzi	AGAATGAATACCAGTGGAACAAAAAAGTAGACTATAATTTTAGCGCAACTGAAACCAACATGACCAGT
Zijiehong	AGAATGAATACCAGTGGAACAAAAAAGTAGACTATAATTTTAGCGCAACTGAAACCAACATGACCAGT
Baihuamai	AGAATGAATACCAGTGGAACAAAAAAGTAGACTATAATTTTAGCGCAACTGAAACCAACATGACCAGT
Huixianhong	AGAATGAATACCAGTGGAACAAAAAAGTAGACTATAATTTTAGCGCAACTGAAACCAACATGACCAGT
Baimangmai	AGAATGAATACCAGTGGAACAAAAAAGTAGACTATAATTTTAGCGCAACTGAAACCAACATGACCAGT

```
                 1,230     1,240     1,250     1,260     1,270     1,280     1,290
Hulutou          CGTAGAACACAAATTTAGACAAAGTGGACCAAATCATAAGAAATATTAAAACATTAA-TTTTTTAGAA
Baihulu          CGTAGAACACAAATTTAGACAAAGTGGACCAAATCATAAGAAATATTAAAACATTAA-TTTTTTAGAA
Chiyacao         CGTAGAACACAAATTTAGACAAAGTGGACCAAATCATAAGAAATATTAAAACATTAA-TTTTTTAGAA
Hongmangmai      CGTAGAACACAAATTTAGACAAAGTGGACCAAATCATAAGAAATATTAAAACATTAA-TTTTTTAGAA
Hongmai          CGTAGAACACAAATTTAGACAAAGTGGACCAAATCATAAGAAATATTAAAACATTAA-TTTTTTAAAA
Wangshuibai      CGTAGAACACAAATTTAGACAAAGTGGACCAAATCATAAGAAATATTAAAACATTAA-TTTTTTAGAA
Shi 4185         CGTAGAACACAAATTTAGACAAAGTGGACCAAATCATAAGAAATATTAAAACATTAA-TTTTTTAGAA
Mazhamai         CGTAGAACACAAATTTAGACAAAGTGGACCAAATCATAAGAAATATTAAAACATTAA-TTTTTTAGAA
Xiaofoshou       CGTAGAACACAAATTTAGACAAAGTGGACCAAATCATAAGAAATATTAAAACATTAA-TTTTTTAGAA
Aikang 58        CGTAGAACACAAATTTAGACAAAGTGGACCAAATCATAAGAAATATTAAAACATTAA-TTTTTTAGAA
AL8/78           CGTAGAACACAAATTTAGACAAAGTGGACCAAATCATAAGAAATATTAAAACATTAATTTTTTTAGAA
PI 431602        CGTAGAACACAAATTTAGACAAAGTGGACCAAATCATAAGAAATATTAAAACATTAATTTTTTTAGAA
PI 486274        CGTAGAACACAAATTTAGACAAAGTGGACCAAATCATAAGAAATATTAAAACATTAATTTTTTTAGAA
RM 000182        CATAGAACACAAATTTAGACAAAGTGGACCAAATCATAAGAAATATTAAAACATTAATTTTTTTAGAA
PI 511381        CATAGAACACAAATTTAGACAAAGTGGACCAAATCATAAGAAATATTAAAACATTAATTTTTTTAGAA
PI 486271        CATAGAACACAAATTTAGACAAAGTGGACCAAATCATAAGAAATATTAAAACATTAATTTTTTTAGAA
PI 511367        CATAGAACACAAATTTAGACAAAGTGGACCAAATCATAAGAAATATTAAAACATTAATTTTTTTAGAA
Chinese Spring   CGTAGAACACAAATTTAGACAAAGTGGACCAAATCATAAGAAATATTAGAACATTAATTTTTTTAGAA
Hanzhongbai      CGTAGAACACAAATTTAGACAAAGTGGACCAAATCATAAGAAATATTAGAACATTAATTTTTTTAGAA
Hongtoumai       CGTAGAACACAAATTTAGACAAAGTGGACCAAATCATAAGAAATATTAGAACATTAATTTTTTTAGAA
Hongyouzi        CGTAGAACACAAATTTAGACAAAGTGGACCAAATCATAAGAAATATTAGAACATTAATTTTTTTAGAA
Zijiehong        CGTAGAACACAAATTTAGACAAAGTGGACCAAATCATAAGAAATATTAGAACATTAATTTTTTTAGAA
Baihuamai        CGTAGAACACAAATTTAGACAAAGTGGACCAAATCATAAGAAATATTAGAACATTAATTTTTTTAGAA
Huixianhong      CGTAGAACACAAATTTAGACAAAGTGGACCAAATCATAAGAAATATTAGAACATTAATTTTTTTAGAA
Baimangmai       CGTAGAACACAAATTTAGACAAAGTGGACCAAATCATAAGAAATATTAGAACATTAATTTTTTTAGAA

                   1,300     1,310     1,320     1,330     1,340     1,350     1,360
Hulutou          CATAAGGCACAAAATTATAGTGCTCGTGAATCAAAACTTGGCTACAAGTGGAACACCTATTGTAATAA
Baihulu          CATAAGGCACAAAATTATAGTGCTCGTGAATCAAAACTTGGCTACAAGTGGAACACCTATTGTAATAA
Chiyacao         CATAAGGCACAAAATTATAGTGCTCGTGAATCAAAACTTGGCTACAAGTGGAACACCTATTGTAATAA
Hongmangmai      CATAAGGCACAAAATTATAGTGCTCGTGAATCAAAACTTGGCTACAAGTGGAACACCTATTGTAATAA
Hongmai          CATAAGGCACAAAATTATAGTGCTCGTGAATCAAAACTTGGCTACAAGTGGAACACCTATTGTAATAA
Wangshuibai      CATAAGGCACAAAATTATAGTGCTCGTGAATCAAAACTTGGCTACAAGTGGAACACCTATTGTAATAA
Shi 4185         CATAAGGCACAAAATTATAGTGCTCGTGAATCAAAACTTGGCTACAAGTGGAACACCTATTGTAATAA
Mazhamai         CATAAGGCACAAAATTATAGTGCTCGTGAATCAAAACTTGGCTACAAGTGGAACACCTATTGTAATAA
Xiaofoshou       CATAAGGCACAAAATTATAGTGCTCGTGAATCAAAACTTGGCTACAAGTGGAACACCTATTGTAATAA
Aikang 58        CATAAGGCACAAAATTATAGTGCTCGTGAATCAAAACTTGGCTACAAGTGGAACACCTATTGTAATAA
AL8/78           CATAAGGCACAAAATTATAGTGCTCGTGAATCAAAACTTGGCTACAAGTGGAACAAATATTGTGACAA
PI 431602        CATAAGGCACAAAATTATAGTGCTCGTGAATCAAAACTTGGCTACAAGTGGAACAAATATTGTGACAA
PI 486274        CATAAGGCACAAAATTATAGTGCTCGTGAATCAAAACTTGGCTACAAGTGGAACAAATATTGTGACAA
RM 000182        CATAAGGCACAAAATTATAGTGCTCATGAATCAAAACTTGGCTACAAGTGGAACAAATATTGTAACAA
PI 511381        CATAAGGCACAAAATTATAGTGCTCATGAATCAAAACTTGGCTACAAGTGGAACAAATATTGTAACAA
PI 486271        CATAAGGCACAAAATTATAGTGCTCATGAATCAAAACTTGGCTACAAGTGGAACAAATATTGTAACAA
PI 511367        CATAAGGCACAAAATTATAGTGCTCATGAATCAAAACTTGGCTACAAGTGGAACAAATATTGTAACAA
Chinese Spring   CATAAGGCACAAAATTATAGTGCTCGTGAATCAAAACTTGGCTACAAGTGGAACAAATATTGTAACAA
Hanzhongbai      CATAAGGCACAAAATTATAGTGCTCGTGAATCAAAACTTGGCTACAAGTGGAACAAATATTGTAACAA
Hongtoumai       CATAAGGCACAAAATTATAGTGCTCGTGAATCAAAACTTGGCTACAAGTGGAACAAATATTGTAACAA
Hongyouzi        CATAAGGCACAAAATTATAGTGCTCGTGAATCAAAACTTGGCTACAAGTGGAACAAATATTGTAACAA
Zijiehong        CATAAGGCACAAAATTATAGTGCTCGTGAATCAAAACTTGGCTACAAGTGGAACAAATATTGTAACAA
Baihuamai        CATAAGGCACAAAATTATAGTGCTCGTGAATCAAAACTTGGCTACAAGTGGAACAAATATTGTAACAA
Huixianhong      CATAAGGCACAAAATTATAGTGCTCGTGAATCAAAACTTGGCTACAAGTGGAACAAATATTGTAACAA
Baimangmai       CATAAGGCACAAAATTATAGTGCTCGTGAATCAAAACTTGGCTACAAGTGGAACAAATATTGTAACAA
```

```
                      1,370     1,380     1,390     1,400     1,410     1,420
Hulutou          ATGCAAGAAAACTATTAGAGGCTTGTGCAATAAAATTTCGGTGCCCATGGAACAAATCAAAATTCTAG
Baihulu          ATGCAAGAAAACTATTAGAGGCTTGTGCAATAAAATTTCGGTGCCCATGGAACAAATCAAAATTCTAG
Chiyacao         ATGCAAGAAAACTATTAGAGGCTTGTGCAATAAAATTTCGGTGCCCATGGAACAAATCAAAATTCTAG
Hongmangmai      ATGCAAGAAAACTATTAGAGGCTTGTGCAATAAAATTTCGGTGCCCATGGAACAAATCAAAATTCTAG
Hongmai          ATGCAAGAAAACTATTAGAGGCTTGTGCAATAAAATTTCGGTGCCCATGGAACAAATCAAAATTCTAG
Wangshuibai      ATGCAAGAAAACTATTAGAGGCTTGTGCAATAAAATTTCGGTGCCCATGGAACAAATCAAAATTCTAG
Shi 4185         ATGCAAGAAAACTATTAGAGGCTTGTGCAATAAAATTTCGGTGCCCATGGAACAAATCAAAATTCTAG
Mazhamai         ATGCAAGAAAACTATTAGAGGCTTGTGCAAAAAAATTTCGGTGCCCATGGAACAAATCAAAATTCTAG
Xiaofoshou       ATGCAAGAAAACTATTAGAGGCTTGTGCAAAAAAATTTCGGTGCCCATGGAACAAATCAAAATTCTAG
Aikang 58        ATGCAAGAAAACTATTAGAGGCTTGTGCAATAAAATTTCGGTGCCCATGGAACAAATCAAAATTCTAG
AL8/78           ATACAAGAAAACTATTAGAGGCTTGTGCAACAAAATTTCGGTGCCCATGGAACAAATCAAAATTCTAG
PI 431602        ATACAAGAAAACTATTAGAGGCTTGTGCAACAAAATTTCGGTGCCCATGGAACAAATCAAAATTCTAG
PI 486274        ATACAAGAAAACTATTAGAGGCTTGTGCAACAAAATTTCGGTGCCCATGGAACAAATCAAAATTCTAG
RM 000182        ATACAAGAAAACTATTAGAGGCTTGTGCAACAAAATTTCGGTGCCCATGGAACAAATCAAAACTCTAG
PI 511381        ATACAAGAAAACTATTAGAGGCTTGTGCAACAAAATTTCGGTGCCCATGGAACAAATCAAAACTCTAG
PI 486271        ATACAAGAAAACTATTAGAGGCTTGTGCAACAAAATTTCGGTGCCCATGGAACAAATCAAAACTCTAG
PI 511367        ATACAAGAAAACTATTAGAGGCTTGTGCAACAAAATTTCGGTGCCCATGGAACAAATCAAAACTCTAG
Chinese Spring   ATACAAGAAAACTATTAGAGGCTTGTGCAACAAAATTTCGGTGCCCATGGAACAAATCAAAATTCTAG
Hanzhongbai      ATACAAGAAAACTATTAGAGGCTTGTGCAACAAAATTTCGGTGCCCATGGAACAAATCAAAATTCTAG
Hongtoumai       ATACAAGAAAACTATTAGAGGCTTGTGCAACAAAATTTCGGTGCCCATGGAACAAATCAAAATTCTAG
Hongyouzi        ATACAAGAAAACTATTAGAGGCTTGTGCAACAAAATTTCGGTGCCCATGGAACAAATCAAAATTCTAG
Zijiehong        ATACAAGAAAACTATTAGAGGCTTGTGCAACAAAATTTCGGTGCCCATGGAACAAATCAAAATTCTAG
Baihuamai        ATACAAGAAAACTATTAGAGGCTTGTGCAACAAAATTTCGGTGCCCATGGAACAAATCAAAATTCTAG
Huixianhong      ATACAAGAAAACTATTAGAGGCTTGTGCAACAAAATTTCGGTGCCCATGGAACAAATCAAAATTCTAG
Baimangmai       ATACAAGAAAACTATTAGAGGCTTGTGCAACAAAATTTCGGTGCCCATGGAACAAATCAAAATTCTAG
```

```
                 1,430     1,440     1,450     1,460     1,470     1,480     1,490
Hulutou          TGGAACAAAAGTTTTAACTAAGT-GAATGAGCAAAGTATATCACTTGCAGGAACAAATTGAAACTACT
Baihulu          TGGAACAAAAGTTTTAACTAAGT-GAATGAGCAAAGTATATCACTTGCAGGAACAAATTGAAACTACT
Chiyacao         TGGAACAAAAGTTTTAACTAAGT-GAATGAGCAAAGTATATCACTTGCAGGAACAAATTGAAACTACT
Hongmangmai      TGGAACAAAAGTTTTAACTAAGT-GAATGAGCAAAGTATATCACTTGCAGGAACAAATTGAAACTACT
Hongmai          TGGAACAAAAGTTTTAACTAAGT-GAATGAGCAAAGTATATCACTTGCAGGAACAAATTGAAACTACT
Wangshuibai      TGGAACAAAAGTTTTAACTAAGT-GAATGAGCAAAGTATATCACTTGCAGGAACAAATTGAAACTACT
Shi 4185         TGGAACAAAAGTTTTAACTAAGT-GAATGAGCAAAGTATATCACTTGCAGGAACAAATTGAAACTACT
Mazhamai         TGGAACAAAAGTTTTAACTAAGT-GAATGAGCAAAGTATATCACTTGCAGGAACAAATTGAAACTACT
Xiaofoshou       TGGAACAAAAGTTTTAACTAAGT-GAATGAGCAAAGTATATCACTTGCAGGAACAAATTGAAACTACT
Aikang 58        TGGAACAAAAGTTTTAACTAAGT-GAATGAGCAAAGTATATCACTTGCAGGAACAAATTGAAACTACT
AL8/78           TGGAACAAAAGTTTTAACTAACTAGAATGAGCAAAGTATATCACTTGCAGGAACAAATTGAAACTACT
PI 431602        TGGAACAAAAGTTTTAACTAACTAGAATGAGCAAAGTATATCACTTGCAGGAACAAATTGAAACTACT
PI 486274        TGGAACAAAAGTTTTAACTAACTAGAATGAGCAAAGTATATCACTTGCAGGAACAAATTGAAACTACT
RM 000182        TGGAACAAAAAATTTAACTAACTAGAATGAGCAAAGTATATCACTTGCAGGAACAAATTGAAACTACT
PI 511381        TGGAACAAAAAATTTAACTAACTAGAATGAGCAAAGTATATCACTTGCAGGAACAAATTGAAACTACT
PI 486271        TGGAACAAAAAATTTAACTAACTAGAATGAGCAAAGTATATCACTTGCAGGAACAAATTGAAACTACT
PI 511367        TGGAACAAAAAATTTAACTAACTAGAATGAGCAAAGTATATCACTTGCAGGAACAAATTGAAACTACT
Chinese Spring   TGGAACAAAAGTTTTAACTAACTAGAATGAGCAAAGTATATCACTTGCAGGAAAAAATTGAAACTACT
Hanzhongbai      TGGAACAAAAGTTTTAACTAACTAGAATGAGCAAAGTATATCACTTGCAGGAAAAAATTGAAACTACT
Hongtoumai       TGGAACAAAAGTTTTAACTAACTAGAATGAGCAAAGTATATCACTTGCAGGAAAAAATTGAAACTACT
Hongyouzi        TGGAACAAAAGTTTTAACTAACTAGAATGAGCAAAGTATATCACTTGCAGGAAAAAATTGAAACTACT
Zijiehong        TGGAACAAAAGTTTTAACTAACTAGAATGAGCAAAGTATATCACTTGCAGGAAAAAATTGAAACTACT
Baihuamai        TGGAACAAAAGTTTTAACTAACTAGAATGAGCAAAGTATATCACTTGCAGGAAAAAATTGAAACTACT
Huixianhong      TGGAACAAAAGTTTTAACTAACTAGAATGAGCAAAGTATATCACTTGCAGGAAAAAATTGAAACTACT
Baimangmai       TGGAACAAAAGTTTTAACTAACTAGAATGAGCAAAGTATATCACTTGCAGGAAAAAATTGAAACTACT
```

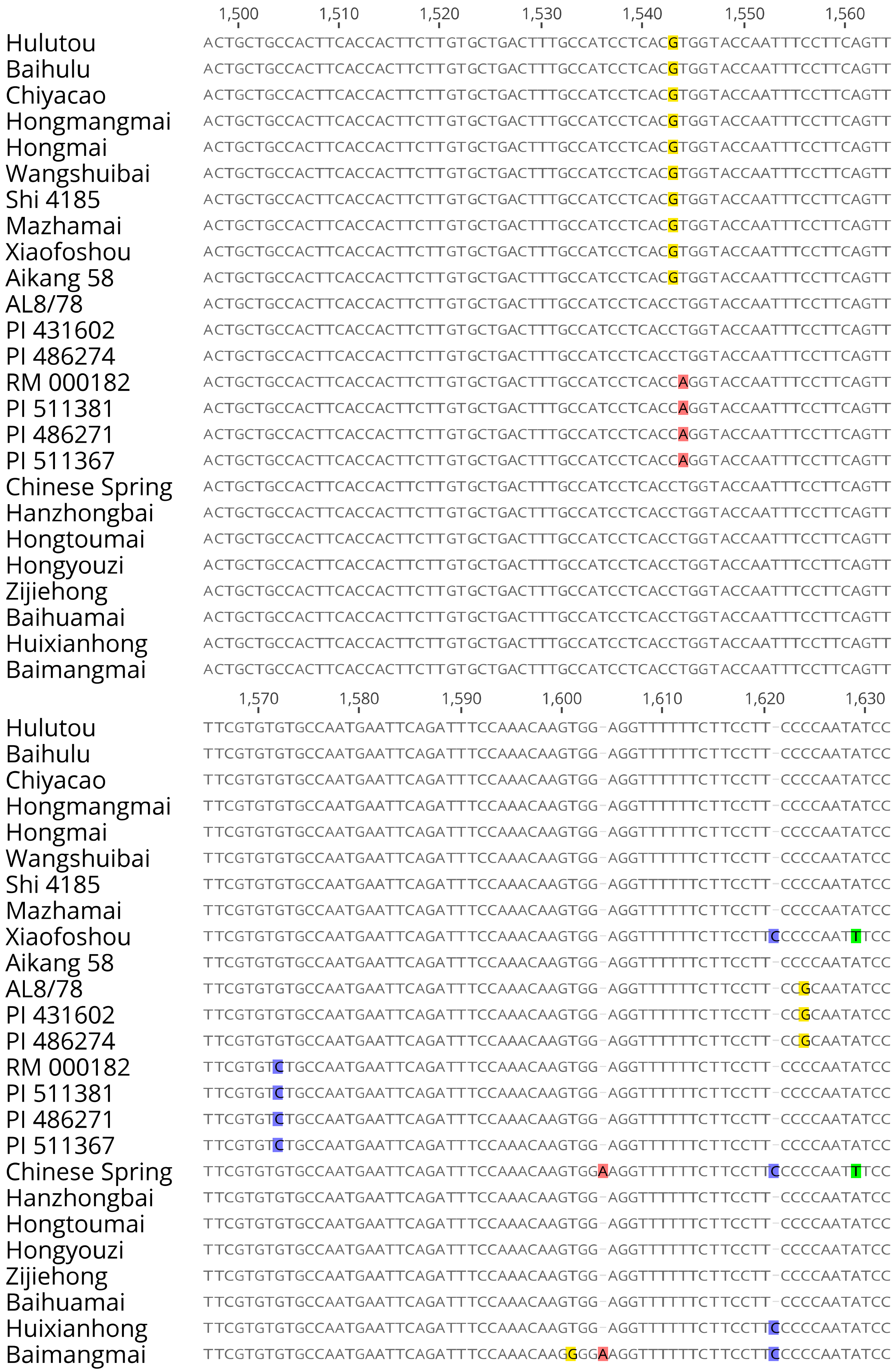

1,500 1,510 1,520 1,530 1,540 1,550 1,560
Hulutou ACTGCTGCCACTTCACCACTTCTTGTGCTGACTTTGCCATCCTCACGTGGTACCAATTTCCTTCAGTT
Baihulu ACTGCTGCCACTTCACCACTTCTTGTGCTGACTTTGCCATCCTCACGTGGTACCAATTTCCTTCAGTT
Chiyacao ACTGCTGCCACTTCACCACTTCTTGTGCTGACTTTGCCATCCTCACGTGGTACCAATTTCCTTCAGTT
Hongmangmai ACTGCTGCCACTTCACCACTTCTTGTGCTGACTTTGCCATCCTCACGTGGTACCAATTTCCTTCAGTT
Hongmai ACTGCTGCCACTTCACCACTTCTTGTGCTGACTTTGCCATCCTCACGTGGTACCAATTTCCTTCAGTT
Wangshuibai ACTGCTGCCACTTCACCACTTCTTGTGCTGACTTTGCCATCCTCACGTGGTACCAATTTCCTTCAGTT
Shi 4185 ACTGCTGCCACTTCACCACTTCTTGTGCTGACTTTGCCATCCTCACGTGGTACCAATTTCCTTCAGTT
Mazhamai ACTGCTGCCACTTCACCACTTCTTGTGCTGACTTTGCCATCCTCACGTGGTACCAATTTCCTTCAGTT
Xiaofoshou ACTGCTGCCACTTCACCACTTCTTGTGCTGACTTTGCCATCCTCACGTGGTACCAATTTCCTTCAGTT
Aikang 58 ACTGCTGCCACTTCACCACTTCTTGTGCTGACTTTGCCATCCTCACGTGGTACCAATTTCCTTCAGTT
AL8/78 ACTGCTGCCACTTCACCACTTCTTGTGCTGACTTTGCCATCCTCACCTGGTACCAATTTCCTTCAGTT
PI 431602 ACTGCTGCCACTTCACCACTTCTTGTGCTGACTTTGCCATCCTCACCTGGTACCAATTTCCTTCAGTT
PI 486274 ACTGCTGCCACTTCACCACTTCTTGTGCTGACTTTGCCATCCTCACCTGGTACCAATTTCCTTCAGTT
RM 000182 ACTGCTGCCACTTCACCACTTCTTGTGCTGACTTTGCCATCCTCACCAGGTACCAATTTCCTTCAGTT
PI 511381 ACTGCTGCCACTTCACCACTTCTTGTGCTGACTTTGCCATCCTCACCAGGTACCAATTTCCTTCAGTT
PI 486271 ACTGCTGCCACTTCACCACTTCTTGTGCTGACTTTGCCATCCTCACCAGGTACCAATTTCCTTCAGTT
PI 511367 ACTGCTGCCACTTCACCACTTCTTGTGCTGACTTTGCCATCCTCACCAGGTACCAATTTCCTTCAGTT
Chinese Spring ACTGCTGCCACTTCACCACTTCTTGTGCTGACTTTGCCATCCTCACCTGGTACCAATTTCCTTCAGTT
Hanzhongbai ACTGCTGCCACTTCACCACTTCTTGTGCTGACTTTGCCATCCTCACCTGGTACCAATTTCCTTCAGTT
Hongtoumai ACTGCTGCCACTTCACCACTTCTTGTGCTGACTTTGCCATCCTCACCTGGTACCAATTTCCTTCAGTT
Hongyouzi ACTGCTGCCACTTCACCACTTCTTGTGCTGACTTTGCCATCCTCACCTGGTACCAATTTCCTTCAGTT
Zijiehong ACTGCTGCCACTTCACCACTTCTTGTGCTGACTTTGCCATCCTCACCTGGTACCAATTTCCTTCAGTT
Baihuamai ACTGCTGCCACTTCACCACTTCTTGTGCTGACTTTGCCATCCTCACCTGGTACCAATTTCCTTCAGTT
Huixianhong ACTGCTGCCACTTCACCACTTCTTGTGCTGACTTTGCCATCCTCACCTGGTACCAATTTCCTTCAGTT
Baimangmai ACTGCTGCCACTTCACCACTTCTTGTGCTGACTTTGCCATCCTCACCTGGTACCAATTTCCTTCAGTT
1,570 1,580 1,590 1,600 1,610 1,620 1,630
Hulutou TTCGTGTGTGCCAATGAATTCAGATTTCCAAACAAGTGG-AGGTTTTTTCTTCCTT-CCCCAATATCC
Baihulu TTCGTGTGTGCCAATGAATTCAGATTTCCAAACAAGTGG-AGGTTTTTTCTTCCTT-CCCCAATATCC
Chiyacao TTCGTGTGTGCCAATGAATTCAGATTTCCAAACAAGTGG-AGGTTTTTTCTTCCTT-CCCCAATATCC
Hongmangmai TTCGTGTGTGCCAATGAATTCAGATTTCCAAACAAGTGG-AGGTTTTTTCTTCCTT-CCCCAATATCC
Hongmai TTCGTGTGTGCCAATGAATTCAGATTTCCAAACAAGTGG-AGGTTTTTTCTTCCTT-CCCCAATATCC
Wangshuibai TTCGTGTGTGCCAATGAATTCAGATTTCCAAACAAGTGG-AGGTTTTTTCTTCCTT-CCCCAATATCC
Shi 4185 TTCGTGTGTGCCAATGAATTCAGATTTCCAAACAAGTGG-AGGTTTTTTCTTCCTT-CCCCAATATCC
Mazhamai TTCGTGTGTGCCAATGAATTCAGATTTCCAAACAAGTGG-AGGTTTTTTCTTCCTT-CCCCAATATCC
Xiaofoshou TTCGTGTGTGCCAATGAATTCAGATTTCCAAACAAGTGG-AGGTTTTTTCTTCCTTCCCCCAATTTCC
Aikang 58 TTCGTGTGTGCCAATGAATTCAGATTTCCAAACAAGTGG-AGGTTTTTTCTTCCTT-CCCCAATATCC
AL8/78 TTCGTGTGTGCCAATGAATTCAGATTTCCAAACAAGTGG-AGGTTTTTTCTTCCTT-CCGCAATATCC
PI 431602 TTCGTGTGTGCCAATGAATTCAGATTTCCAAACAAGTGG-AGGTTTTTTCTTCCTT-CCGCAATATCC
PI 486274 TTCGTGTGTGCCAATGAATTCAGATTTCCAAACAAGTGG-AGGTTTTTTCTTCCTT-CCGCAATATCC
RM 000182 TTCGTGTCTGCCAATGAATTCAGATTTCCAAACAAGTGG-AGGTTTTTTCTTCCTT-CCCCAATATCC
PI 511381 TTCGTGTCTGCCAATGAATTCAGATTTCCAAACAAGTGG-AGGTTTTTTCTTCCTT-CCCCAATATCC
PI 486271 TTCGTGTCTGCCAATGAATTCAGATTTCCAAACAAGTGG-AGGTTTTTTCTTCCTT-CCCCAATATCC
PI 511367 TTCGTGTCTGCCAATGAATTCAGATTTCCAAACAAGTGG-AGGTTTTTTCTTCCTT-CCCCAATATCC
Chinese Spring TTCGTGTGTGCCAATGAATTCAGATTTCCAAACAAGTGGAAGGTTTTTTCTTCCTTCCCCCAATTTCC
Hanzhongbai TTCGTGTGTGCCAATGAATTCAGATTTCCAAACAAGTGG-AGGTTTTTTCTTCCTT-CCCCAATATCC
Hongtoumai TTCGTGTGTGCCAATGAATTCAGATTTCCAAACAAGTGG-AGGTTTTTTCTTCCTT-CCCCAATATCC
Hongyouzi TTCGTGTGTGCCAATGAATTCAGATTTCCAAACAAGTGG-AGGTTTTTTCTTCCTT-CCCCAATATCC
Zijiehong TTCGTGTGTGCCAATGAATTCAGATTTCCAAACAAGTGG-AGGTTTTTTCTTCCTT-CCCCAATATCC
Baihuamai TTCGTGTGTGCCAATGAATTCAGATTTCCAAACAAGTGG-AGGTTTTTTCTTCCTT-CCCCAATATCC
Huixianhong TTCGTGTGTGCCAATGAATTCAGATTTCCAAACAAGTGG-AGGTTTTTTCTTCCTTCCCCCAATATCC
Baimangmai TTCGTGTGTGCCAATGAATTCAGATTTCCAAACAAGGGGAAGGTTTTTTCTTCCTTCCCCCAATATCC

	1,640 – 1,700
Hulutou	CTCGTTCTACCCAACTA--TATAATGGGGCAGAGAAGGATAAGCAAAGTAGGCGGTAGAACAAAAAA-
Baihulu	CTCGTTCTACCCAACTA--TATAATGGGGCAGAGAAGGATAAGCAAAGTAGGCGGTAGAACAAAAAA-
Chiyacao	CTCGTTCTACCCAACTA--TATAATGGGGCAGAGAAGGATAAGCAAAGTAGGCGGTAGAACAAAAAA-
Hongmangmai	CTCGTTCTACCCAACTA--TATAATGGGGCAGAGAAGGATAAGCAAAGTAGGCGGTAGAACAAAAAA-
Hongmai	CTCGTTCTACCCAACTA--TATAATGGGGCAGAGAAGGATAAGCAAAGTAGGCGGTAGAACAAAAAA-
Wangshuibai	CTCGTTCTACCCAACTA--TATAATGGGGCAGAGAAGGATAAGCAAAGTAGGCGGTAGAACAAAAAA-
Shi 4185	CTCGTTCTACCCAACTA--TATAATGGGGCAGAGAAGGATAAGCAAAGTAGGCGGTAGAACAAAAAA-
Mazhamai	CTCGTTCTACCCAACTA--TATAATGGGGCAGAGAAGGATAAGCAAAGTAGGCGGTAGAACAAAAAA-
Xiaofoshou	CTCGTTCTACCCAACTA--TATAATGGGGCAGAGAAGGATAAGCAAAGTAGGCGGTAGAACAAAAAAT
Aikang 58	CTCGTTCTACCCAACTA--TATAATGGGGCAGAGAAGGATAAGCAAAGTAGGCGGTAGAACAAAAAA-
AL8/78	CTCGTTCTACCCAACTATCTATAATGGGGCAGAGAAGGATAAGCAAAGTAGGCGGTAGAACAAAAAA-
PI 431602	CTCGTTCTACCCAACTATCTATAATGGGGCAGAGAAGGATAAGCAAAGTAGGCGGTAGAACAAAAAA-
PI 486274	CTCGTTCTACCCAACTATCTATAATGGGGCAGAGAAGGATAAGCAAAGTAGGCGGTAGAACAAAAAA-
RM 000182	CTCGTTCTACCCAACTATCTATAATGGGGCAGAGAAGGATAAGCAAAGTAGGCGGTAGAACAAAAAA-
PI 511381	CTCGTTCTACCCAACTATCTATAATGGGGCAGAGAAGGATAAGCAAAGTAGGCGGTAGAACAAAAAA-
PI 486271	CTCGTTCTACCCAACTATCTATAATGGGGCAGAGAAGGATAAGCAAAGTAGGCGGTAGAACAAAAAA-
PI 511367	CTCGTTCTACCCAACTATCTATAATGGGGCAGAGAAGGATAAGCAAAGTAGGCGGTAGAACAAAAAA-
Chinese Spring	CTCGTTCTACCCAACTATCTATAAT-GGGCAGAGAAGGATAAGCAAAGTAGGCGGTAGAACAAAAAAT
Hanzhongbai	CTCGTTCTACCCAACTATCTATAATGGGGCAGAGAAGGATAAGCAAAGTAGGCGGTAGAACAAAAAA-
Hongtoumai	CTCGTTCTACCCAACTATCTATAATGGGGCAGAGAAGGATAAGCAAAGTAGGCGGTAGAACAAAAAA-
Hongyouzi	CTCGTTCTACCCAACTATCTATAATGGGGCAGAGAAGGATAAGCAAAGTAGGCGGTAGAACAAAAAA-
Zijiehong	CTCGTTCTACCCAACTATCTATAATGGGGCAGAGAAGGATAAGCAAAGTAGGCGGTAGAACAAAAAA-
Baihuamai	CTCGTTCTACCCAACTATCTATAATGGGGCAGAGAAGGATAAGCAAAGTAGGCGGTAGAACAAAAAA-
Huixianhong	CTCGTTCTACCCAACTATCTATAATGGGGCAGAGAAGGATAAGCAAAGTAGGCGGTAGAACAAAAAA-
Baimangmai	CTCGTTCTACCCAACTATCTATAATGGGGCAGAGAAGGATAAGCAAAGTAGGCGGTAGAACAAAAAAA

	1,710 – 1,760
Hulutou	TTAATGTAATTGGGACAAAATTTAATCAGTAAATGCTCACTAGTCTGAGGATAAACGAAAGCATGAAA
Baihulu	TTAATGTAATTGGGACAAAATTTAATCAGTAAATGCTCACTAGTCTGAGGATAAACGAAAGCATGAAA
Chiyacao	TTAATGTAATTGGGACAAAATTTAATCAGTAAATGCTCACTAGTCTGAGGATAAACGAAAGCATGAAA
Hongmangmai	TTAATGTAATTGGGACAAAATTTAATCAGTAAATGCTCACTAGTCTGAGGATAAACGAAAGCATGAAA
Hongmai	TTAATGTAATTGGGACAAAATTTAATCAGTAAATGCTCACTAGTCTGAGGATAAACGAAAGCATGAAA
Wangshuibai	TTAATGTAATTGGGACAAAATTTAATCAGTAAATGCTCACTAGTCTGAGGATAAACGAAAGCATGAAA
Shi 4185	TTAATGTAATTGGGACAAAATTTAATCAGTAAATGCTCACTAGTCTGAGGATAAACGAAAGCATGAAA
Mazhamai	TTAATGTAATTGGGACAAAATTTAATCAGTAAATGCTCACTAGTCTGAGGATAAACGAAAGCATGAAA
Xiaofoshou	TTAATGTAATTGGGACAAAATTTAATCAGTAAATGCTCACTAGTCTGAGGATAAACGAAAGCATGAAA
Aikang 58	TTAATGTAATTGGGACAAAATTTAATCAGTAAATGCTCACTAGTCTGAGGATAAACGAAAGCATGAAA
AL8/78	TTAATGTAATTGGGACAAAATTTAATCAGTAAATGCTCACTAGTCTGAGGATAAACGAAAGCATGAAA
PI 431602	TTAATGTAATTGGGACAAAATTTAATCAGTAAATGCTCACTAGTCTGAGGATAAACGAAAGCATGAAA
PI 486274	TTAATGTAATTGGGACAAAATTTAATCAGTAAATGCTCACTAGTCTGAGGATAAACGAAAGCATGAAA
RM 000182	TTAATGTAATTGGGACAAAATTTAATCAGTAAATGCTCACTAGTCTGAGGATAAACGAAAGCATGAAA
PI 511381	TTAATGTAATTGGGACAAAATTTAATCAGTAAATGCTCACTAGTCTGAGGATAAACGAAAGCATGAAA
PI 486271	TTAATGTAATTGGGACAAAATTTAATCAGTAAATGCTCACTAGTCTGAGGATAAACGAAAGCATGAAA
PI 511367	TTAATGTAATTGGGACAAAATTTAATCAGTAAATGCTCACTAGTCTGAGGATAAACGAAAGCATGAAA
Chinese Spring	TTAATGTAATTGGGACAAAATTTAATCAGTAAATGCTCACTAGTCTGAGGATAAACGAAAGCATGAAA
Hanzhongbai	TTAATGTAATTGGGACAAAATTTAATCAGTAAATGCTCACTAGTCTGAGGATAAACGAAAGCATGAAA
Hongtoumai	TTAATGTAATTGGGACAAAATTTAATCAGTAAATGCTCACTAGTCTGAGGATAAACGAAAGCATGAAA
Hongyouzi	TTAATGTAATTGGGACAAAATTTAATCAGTAAATGCTCACTAGTCTGAGGATAAACGAAAGCATGAAA
Zijiehong	TTAATGTAATTGGGACAAAATTTAATCAGTAAATGCTCACTAGTCTGAGGATAAACGAAAGCATGAAA
Baihuamai	TTAATGTAATTGGGACAAAATTTAATCAGTAAATGCTCACTAGTCTGAGGATAAACGAAAGCATGAAA
Huixianhong	TTAATGTAATTGGGACAAAATTTAATCAGTAAATGCTCACTAGTCTGAGGATAAACGAAAGCATGAAA
Baimangmai	TTAATGTAATTGGGACAAAATTTAATCAGTAAATGCTCACTAGTCTGAGGATAAACGAAAGCATGAAA

	1,770 1,780 1,790 1,800 1,810 1,820 1,830
Hulutou	CAAGGAAAAGATGAGGATGAGCAACTATTGGAATAAAAGCAAAATTAAACTAGCAGAGCAAAGTAAAG
Baihulu	CAAGGAAAAGATGAGGATGAGCAACTATTGGAATAAAAGCAAAATTAAACTAGCAGAGCAAAGTAAAG
Chiyacao	CAAGGAAAAGATGAGGATGAGCAACTATTGGAATAAAAGCAAAATTAAACTAGCAGAGCAAAGTAAAG
Hongmangmai	CAAGGAAAAGATGAGGATGAGCAACTATTGGAATAAAAGCAAAATTAAACTAGCAGAGCAAAGTAAAG
Hongmai	CAAGGAAAAGATGAGGATGAGCAACTATTGGAATAAAAGCAAAATTAAACTAGCAGAGCAAAGTAAAG
Wangshuibai	CAAGGAAAAGATGAGGATGAGCAACTATTGGAATAAAAGCAAAATTAAACTAGCAGAGCAAAGTAAAG
Shi 4185	CAAGGAAAAGATGAGGATGAGCAACTATTGGAATAAAAGCAAAATTAAACTAGCAGAGCAAAGTAAAG
Mazhamai	CAAGGAAAAGATGAGGATGAGCAACTATTGGAATAAAAGCAAAATTAAACTAGCAGAGCAAAGTAAAG
Xiaofoshou	CAAGGAAAAGATGAGGATGAGCAACTATTGGAATAAAAGCAAAATTAAACTAGCAGAGCAAAGTAAAG
Aikang 58	CAAGGAAAAGATGAGGATGAGCAACTATTGGAATAAAAGCAAAATTAAACTAGCAGAGCAAAGTAAAG
AL8/78	CAAGGAAAAGACGAGGATGAGCAACTATTGGAATAAAAGCAAAATTAAACTAGCAGAGCAAAGTAAAG
PI 431602	CAAGGAAAAGACGAGGATGAGCAACTATTGGAATAAAAGCAAAATTAAACTAGCAGAGCAAAGTAAAG
PI 486274	CAAGGAAAAGACGAGGATGAGCAACTATTGGAATAAAAGCAAAATTAAACTAGCAGAGCAAAGTAAAG
RM 000182	CAAGGAAAAGACGAGGGTGAGCAACTATTGGAATAAAAGAAAAATTAAACTAGCAGAGCAAAGTAAAG
PI 511381	CAAGGAAAAGACGAGGGTGAGCAACTATTGGAATAAAAGAAAAATTAAACTAGCAGAGCAAAGTAAAG
PI 486271	CAAGGAAAAGACGAGGGTGAGCAACTATTGGAATAAAAGAAAAATTAAACTAGCAGAGCAAAGTAAAG
PI 511367	CAAGGAAAAGACGAGGGTGAGCAACTATTGGAATAAAAGAAAAATTAAACTAGCAGAGCAAAGTAAAG
Chinese Spring	CAAGGAAAAGACGAGGGTGAGCAACTATTGGAATAAAAGAAAAATTAAACTAGCAGAGCAAAGTAAAG
Hanzhongbai	CAAGGAAAAGACGAGGGTGAGCAACTATTGGAATAAAAGAAAAATTAAACTAGCAGAGCAAAGTAAAG
Hongtoumai	CAAGGAAAAGACGAGGGTGAGCAACTATTGGAATAAAAGAAAAATTAAACTAGCAGAGCAAAGTAAAG
Hongyouzi	CAAGGAAAAGACGAGGGTGAGCAACTATTGGAATAAAAGAAAAATTAAACTAGCAGAGCAAAGTAAAG
Zijiehong	CAAGGAAAAGACGAGGGTGAGCAACTATTGGAATAAAAGAAAAATTAAACTAGCAGAGCAAAGTAAAG
Baihuamai	CAAGGAAAAGACGAGGGTGAGCAACTATTGGAATAAAAGAAAAATTAAACTAGCAGAGCAAAGTAAAG
Huixianhong	CAAGGAAAAGACGAGGGTGAGCAACTATTGGAATAAAAGAAAAATTAAACTAGCAGAGCAAAGTAAAG
Baimangmai	CAAGGAAAAGACGAGGGTGAGCAACTATTGGAATAAAAGAAAAATTAAACTAGCAGAGCAAAGTAAAG

	1,840 1,850 1,860 1,870 1,880 1,890 1,900
Hulutou	TTGCAACCAAATCATCAGCGAGCACCACATCAGCGCGCTAGTCTCCCCTCCCGGTGGTAACCGCTATT
Baihulu	TTGCAACCAAATCATCAGCGAGCACCACATCAGCGCGCTAGTCTCCCCTCCCGGTGGTAACCGCTATT
Chiyacao	TTGCAACCAAATCATCAGCGAGCACCACATCAGCGCGCTAGTCTCCCCTCCCGGTGGTAACCGCTATT
Hongmangmai	TTGCAACCAAATCATCAGCGAGCACCACATCAGCGCGCTAGTCTCCCCTCCCGGTGGTAACCGCTATT
Hongmai	TTGCAACCAAATCATCAGCGAGCACCACATCAGCGCGCTAGTCTCCCCTCCCGGTGGTAACCGCTATT
Wangshuibai	TTGCAACCAAATCATCAGCGAGCACCACATCAGCGCGCTAGTCTCCCCTCCCGGTGGTAACCGCTATT
Shi 4185	TTGCAACCAAATCATCAGCGAGCACCACATCAGCGCGCTAGTCTCCCCTCCCGGTGGTAACCGCTATT
Mazhamai	TTGCAACCAAATCATCAGCGAGCACCACATCAGCGCGCTAGTCTCCCCTCCCGGTGGTAACCGCTATT
Xiaofoshou	TTGCAACCAAATCATCAGCGAGCACCACATCAGCGCGCTAGTCTCCCCTCCCGGTGGTAACCGCTATT
Aikang 58	TTGCAACCAAATCATCAGCGAGCACCACATCAGCGCGCTAGTCTCCCCTCCCGGTGGTAACCGCTATT
AL8/78	TTGCAACCAAATCATCAGCGAGCACCACATCAGCGCGCTAGTCTCCCCTCCCGGCGGTAACCGCTATT
PI 431602	TTGCAACCAAATCATCAGCGAGCACCACATCAGCGCGCTAGTCTCCCCTCCCGGCGGTAACCGCTATT
PI 486274	TTGCAACCAAATCATCAGCGAGCACCACATCAGCGCGCTAGTCTCCCCTCCCGGCGGTAACCGCTATT
RM 000182	TTGCAACCAAATCATCAGCGAGCACCACATCAGCGCGCTAGTCTCCCCTCCCGGCGGTAACCGCTATT
PI 511381	TTGCAACCAAATCATCAGCGAGCACCACATCAGCGCGCTAGTCTCCCCTCCCGGCGGTAACCGCTATT
PI 486271	TTGCAACCAAATCATCAGCGAGCACCACATCAGCGCGCTAGTCTCCCCTCCCGGCGGTAACCGCTATT
PI 511367	TTGCAACCAAATCATCAGCGAGCACCACATCAGCGCGCTAGTCTCCCCTCCCGGCGGTAACCGCTATT
Chinese Spring	TTGCAACCAAATCATCAGCGAGCACCACATCAGCGCGCTAGTCTCCCCTCCCGGCGGTAACCGCTATT
Hanzhongbai	TTGCAACCAAATCATCAGCGAGCACCACATCAGCGCGCTAGTCTCCCCTCCCGGCGGTAACCGCTATT
Hongtoumai	TTGCAACCAAATCATCAGCGAGCACCACATCAGCGCGCTAGTCTCCCCTCCCGGCGGTAACCGCTATT
Hongyouzi	TTGCAACCAAATCATCAGCGAGCACCACATCAGCGCGCTAGTCTCCCCTCCCGGCGGTAACCGCTATT
Zijiehong	TTGCAACCAAATCATCAGCGAGCACCACATCAGCGCGCTAGTCTCCCCTCCCGGCGGTAACCGCTATT
Baihuamai	TTGCAACCAAATCATCAGCGAGCACCACATCAGCGCGCTAGTCTCCCCTCCCGGCGGTAACCGCTATT
Huixianhong	TTGCAACCAAATCATCAGCGAGCACCACATCAGCGCGCTAGTCTCCCCTCCCGGCGGTAACCGCTATT
Baimangmai	TTGCAACCAAATCATCAGCGAGCACCACATCAGCGCGCTAGTCTCCCCTCCCGGCGGTAACCGCTATT

	1,910 · 1,920 · 1,930 · 1,940 · 1,950 · 1,960 · 1,970
Hulutou	GGTGGCTCACCGGCGTCTAAGGCCCCTAGGCGGATGAAGACTAGATGGCTTTCCTTGCCGATCTACAT
Baihulu	GGTGGCTCACCGGCGTCTAAGGCCCCTAGGCGGATGAAGACTAGATGGCTTTCCTTGCCGATCTACAT
Chiyacao	GGTGGCTCACCGGCGTCTAAGGCCCCTAGGCGGATGAAGACTAGATGGCTTTCCTTGCCGATCTACAT
Hongmangmai	GGTGGCTCACCGGCGTCTAAGGCCCCTAGGCGGATGAAGACTAGATGGCTTTCCTTGCCGATCTACAT
Hongmai	GGTGGCTCACCGGCGTCTAAGGCCCCTAGGCGGATGAAGACTAGATGGCTTTCCTTGCCGATCTACAT
Wangshuibai	GGTGGCTCACCGGCGTCTAAGGCCCCTAGGCGGATGAAGACTAGATGGCTTTCCTTGCCGATCTACAT
Shi 4185	GGTGGCTCACCGGCGTCTAAGGCCCCTAGGCGGATGAAGACTAGATGGCTTTCCTTGCCGATCTACAT
Mazhamai	GGTGGCTCACCGGCGTCTAAGGCCCCTAGGCGGATGAAGACTAGATGGCTTTCCTTGCCGATCTACAT
Xiaofoshou	GGTGGCTCACCGGCGTCTAAGGCCCCTAGGCGGATGAAGACTAGATGGCTTTCCTTGCCGATCTACAT
Aikang 58	GGTGGCTCACCGGCGTCTAAGGCCCCTAGGCGGATGAAGACTAGATGGCTTTCCTTGCCGATCTACAT
AL8/78	GGTGGCTCACCGGCGTCTAAGGCCCCTAGGCGGATGAAGACTAGATGGCTTTCCTTGCCGATCTACAT
PI 431602	GGTGGCTCACCGGCGTCTAAGGCCCCTAGGCGGATGAAGACTAGATGGCTTTCCTTGCCGATCTACAT
PI 486274	GGTGGCTCACCGGCGTCTAAGGCCCCTAGGCGGATGAAGACTAGATGGCTTTCCTTGCCGATCTACAT
RM 000182	GGTGGCTCACCGGCGTCTAAGGCCCCTAGGCGGATGAAGACTAGATGGCTTTCCTTGCCGATCTACAT
PI 511381	GGTGGCTCACCGGCGTCTAAGGCCCCTAGGCGGATGAAGACTAGATGGCTTTCCTTGCCGATCTACAT
PI 486271	GGTGGCTCACCGGCGTCTAAGGCCCCTAGGCGGATGAAGACTAGATGGCTTTCCTTGCCGATCTACAT
PI 511367	GGTGGCTCACCGGCGTCTAAGGCCCCTAGGCGGATGAAGACTAGATGGCTTTCCTTGCCGATCTACAT
Chinese Spring	GGTGGCTCACCGGCGTCTAAGGCCCCTAGGCGGATGAAGACTAGATGGCTTTCCTTGCCGATCTACAT
Hanzhongbai	GGTGGCTCACCGGCGTCTAAGGCCCCTAGGCGGATGAAGACTAGATGGCTTTCCTTGCCGATCTACAT
Hongtoumai	GGTGGCTCACCGGCGTCTAAGGCCCCTAGGCGGATGAAGACTAGATGGCTTTCCTTGCCGATCTACAT
Hongyouzi	GGTGGCTCACCGGCGTCTAAGGCCCCTAGGCGGATGAAGACTAGATGGCTTTCCTTGCCGATCTACAT
Zijiehong	GGTGGCTCACCGGCGTCTAAGGCCCCTAGGCGGATGAAGACTAGATGGCTTTCCTTGCCGATCTACAT
Baihuamai	GGTGGCTCACCGGCGTCTAAGGCCCCTAGGCGGATGAAGACTAGATGGCTTTCCTTGCCGATCTACAT
Huixianhong	GGTGGCTCACCGGCGTCTAAGGCCCCTAGGCGGATGAAGACTAGATGGCTTTCCTTGCCGATCTACAT
Baimangmai	GGTGGCTCACCGGCGTCTAAGGCCCCTAGGCGGATGAAGACTAGATGGCTTTCCTTGCCGATCTACAT

	1,980 · 1,990 · 2,000 · 2,010 · 2,020 · 2,030 · 2,040
Hulutou	GACATCTGGCCCTTGGCTTATTGGTGGCGATTTCAACATGATCGTCTCTGAGGCGGAC-AAGAATAAC
Baihulu	GACATCTGGCCCTTGGCTTATTGGTGGCGATTTCAACATGATCGTCTCTGAGGCGGAC-AAGAATAAC
Chiyacao	GACATCTGGCCCTTGGCTTATTGGTGGCGATTTCAACATGATCGTCTCTGAGGCGGAC-AAGAATAAC
Hongmangmai	GACATCTGGCCCTTGGCTTATTGGTGGCGATTTCAACATGATCGTCTCTGAGGCGGAC-AAGAATAAC
Hongmai	GACATCTGGCCCTTGGCTTATTGGTGGCGATTTCAACATGATCGTCTCTGAGGCGGAC-AAGAATAAC
Wangshuibai	GACATCTGGCCCTTGGCTTATTGGTGGCGATTTCAACATGATCGTCTCTGAGGCGGAC-AAGAATAAC
Shi 4185	GACATCTGGCCCTTGGCTTATTGGTGGCGATTTCAACATGATCGTCTCTGAGGCGGAC-AAGAATAAC
Mazhamai	GACATCTGGCCCTTGGCTTATTGGTGGCGATTTCAACATGATCGTCTCTGAGGCGGAC-AAGAATAAC
Xiaofoshou	GACATCTGGCCCTTGGCTTATTGGTGGCGATTTCAACATGATCGTCTCTGAGGCGGAC-AAGAATAAC
Aikang 58	GACATCTGGCCCTTGGCTTATTGGTGGCGATTTCAACATGATCGTCTCTGAGGCGGAC-AAGAATAAC
AL8/78	GACGTCTGGCCCTTGGCTTATTGGTGGCGATTTCAACATGATTGTCTCTGAGGCGGAC-AAGAATAAC
PI 431602	GACGTCTGGCCCTTGGCTTATTGGTGGCGATTTCAACATGATTGTCTCTGAGGCGGACTAAGAATAAC
PI 486274	GACGTCTGGCCCTTGGCTTATTGGTGGCGATTTCAACATGATTGTCTCTGAGGCGGAC-AAGAATAAC
RM 000182	GACGTCTGGCCCTTGGCTTATTGGTGCCGATTTCAACATGATCGTCTCTGAGGCAGAC-AAGAATAAC
PI 511381	GACGTCTGGCCCTTGGCTTATTGGTGCCGATTTCAACATGATCGTCTCTGAGGCAGAC-AAGAATAAC
PI 486271	GACGTCTGGCCCTTGGCTTATTGGTGCCGATTTCAACATGATCGTCTCTGAGGCAGAC-AAGAATAAC
PI 511367	GACGTCTGGCCCTTGGCTTATTGGTGCCGATTTCAACATGATCGTCTCTGAGGCAGAC-AAGAATAAC
Chinese Spring	GACGTCTGGCCCTTGGCTTATTGGTGCCGATTTCAACATGATCGTCTCTGAGGCGGAC-AAGAATAAC
Hanzhongbai	GACGTCTGGCCCTTGGCTTATTGGTGCCGATTTCAACATGATCGTCTCTGAGGCGGAC-AAGAATAAC
Hongtoumai	GACGTCTGGCCCTTGGCTTATTGGTGCCGATTTCAACATGATCGTCTCTGAGGCGGAC-AAGAATAAC
Hongyouzi	GACGTCTGGCCCTTGGCTTATTGGTGCCGATTTCAACATGATCGTCTCTGAGGCGGAC-AAGAATAAC
Zijiehong	GACGTCTGGCCCTTGGCTTATTGGTGCCGATTTCAACATGATCGTCTCTGAGGCGGAC-AAGAATAAC
Baihuamai	GACGTCTGGCCCTTGGCTTATTGGTGCCGATTTCAACATGATCGTCTCTGAGGCGGAC-AAGAATAAC
Huixianhong	GACGTCTGGCCCTTGGCTTATTGGTGCCGATTTCAACATGATCGTCTCTGAGGCGGAC-AAGAATAAC
Baimangmai	GACGTCTGGCCCTTGGCTTATTGGTGCCGATTTCAACATGATCGTCTCTGAGGCGGAC-AAGAATAAC

```
                   2,050     2,060     2,070     2,080     2,090     2,100
Hulutou          CCGCACGTGAACCGGCGGATC-ATGCATAGCTTTCGATGCTTCCTTGTCGACGAGGAACTTCATGACA
Baihulu          CCGCACGTGAACCGGCGGATC-ATGCATAGCTTTCGATGCTTCCTTGTCGACGAGGAACTTCATGACA
Chiyacao         CCGCACGTGAACCGGCGGATC-ATGCATAGCTTTCGATGCTTCCTTGTCGACGAGGAACTTCATGACA
Hongmangmai      CCGCACGTGAACCGGCGGATC-ATGCATAGCTTTCGATGCTTCCTTGTCGACGAGGAACTTCATGACA
Hongmai          CCGCACGTGAACCGGCGGATC-ATGCATAGCTTTCGATGCTTCCTTGTCGACGAGGAACTTCATGACA
Wangshuibai      CCGCACGTGAACCGGCGGATC-ATGCATAGCTTTCGATGCTTCCTTGTCGACGAGGAACTTCATGACA
Shi 4185         CCGCACGTGAACCGGCGGATC-ATGCATAGCTTTCGATGCTTCCTTGTCGACGAGGAACTTCATGACA
Mazhamai         CCGCACGTGAACCGGCGGATC-ATGCATAGCTTTCGATGCTTCCTTGTCGACGAGGAACTTCATGACA
Xiaofoshou       CCGCACGTGAACCGGCGGATC-ATGCATAGCTTTCGATGCTTCCTTGTCGACGAGGAACTTCATGACA
Aikang 58        CCGCACGTGAACCGGCGGATC-ATGCATAGCTTTCGATGCTTCCTTGTCGACGAGGAACTTCATGACA
AL8/78           CCGCACGTGAACCGGCGGATC-ATGCATCGCTTTCGATGCTTCCTTGTCGACGAGGAACTTCATGACA
PI 431602        CCGCACGTGAACCGGCGGATCAATGCATCGCTTTCGATGCTTCCTTGTCGACGAGGAACTTCATGACA
PI 486274        CCGCACGTGAACCGGCGGATC-ATGCATCGCTTTCGATGCTTCCTTGTCGACGAGGAACTTCATGACA
RM 000182        CCGCACGTGAACCGGCGGATC-ATGCATCGCTTTCGATGCTTCCTTGTCGACGAGGAACTTCATGACA
PI 511381        CCGCACGTGAACCGGCGGATC-ATGCATCGCTTTCGATGCTTCCTTGTCGACGAGGAACTTCATGACA
PI 486271        CCGCACGTGAACCGGCGGATC-ATGCATCGCTTTCGATGCTTCCTTGTCGACGAGGAACTTCATGACA
PI 511367        CCGCACGTGAACCGGCGGATC-ATGCATCGCTTTCGATGCTTCCTTGTCGACGAGGAACTTCATGACA
Chinese Spring   CCGCACGTGAACCGGCGGATC-ATGCATCGCTTTCGATGCTTCCTTGTCGACGAGGAACTTCATGACA
Hanzhongbai      CCGCACGTGAACCGGCGGATC-ATGCATCGCTTTCGATGCTTCCTTGTCGACGAGGAACTTCATGACA
Hongtoumai       CCGCACGTGAACCGGCGGATC-ATGCATCGCTTTCGATGCTTCCTTGTCGACGAGGAACTTCATGACA
Hongyouzi        CCGCACGTGAACCGGCGGATC-ATGCATCGCTTTCGATGCTTCCTTGTCGACGAGGAACTTCATGACA
Zijiehong        CCGCACGTGAACCGGCGGATC-ATGCATCGCTTTCGATGCTTCCTTGTCGACGAGGAACTTCATGACA
Baihuamai        CCGCACGTGAACCGGCGGATC-ATGCATCGCTTTCGATGCTTCCTTGTCGACGAGGAACTTCATGACA
Huixianhong      CCGCACGTGAACCGGCGGATC-ATGCATCGCTTTCGATGCTTCCTTGTCGACGAGGAACTTCATGACA
Baimangmai       CCGCACGTGAACCGGCGGATC-ATGCATCGCTTTCGATGCTTCCTTGTCGACGAGGAACTTCATGACA

                 2,110     2,120     2,130     2,140     2,150     2,160     2,170
Hulutou          TTTGTATGCATGGTAGGTGCTACACATGGTCCAGCGAGCGCAAGA-TTCCTACTCTTGTCAAGAACAA
Baihulu          TTTGTATGCATGGTAGGTGCTACACATGGTCCAGCGAGCGCAAGA-TTCCTACTCTTGTCAAGAACAA
Chiyacao         TTTGTATGCATGGTAGGTGCTACACATGGTCCAGCGAGCGCAAGA-TTCCTACTCTTGTCAAGAACAA
Hongmangmai      TTTGTATGCATGGTAGGTGCTACACATGGTCCAGCGAGCGCAAGA-TTCCTACTCTTGTCAAGAACAA
Hongmai          TTTGTATGCATGGTAGGTGCTACACATGGTCCAGCGAGCGCAAGA-TTCCTACTCTTGTCAAGAACAA
Wangshuibai      TTTGTATGCATGGTAGGTGCTACACATGGTCCAGCGAGCGCAAGA-TTCCTACTCTTGTCAAGAACAA
Shi 4185         TTTGTATGCATGGTAGGTGCTACACATGGTCCAGCGAGCGCAAGA-TTCCTACTCTTGTCAAGAACAA
Mazhamai         TTTGTATGCATGGTAGGTGCTACACATGGTCCAGCGAGCGCAAGA-TTCCTACTCTTGTCAAGAACAA
Xiaofoshou       TTTGTATGCATGGTAGGTGCTACACATGGTCCAGCGAGCGCAAGA-TTCCTACTCTTGTCAAGAACAA
Aikang 58        TTTGTATGCATGGTAGGTGCTACACATGGTCCAGCGAGCGCAAGA-TTCCTACTCTTGTCAAGAACAA
AL8/78           TTTGTATGCATGGTAGGCGCTACACATGGTCCAGCGAGCGCAAGA-TTCCTACTCTTGTCAAGAACAA
PI 431602        TTTGTATGCATGGTAGGCGCTACACATGGTCCAGCGAGCGCAAGA-TTCCTACTCTTGTCAAGAACAA
PI 486274        TTTGTATGCATGGTAGGCGCTACACATGGTCCAGCGAGCGCAAGA-TTCCTACTCTTGTCAAGAACAA
RM 000182        TTTGTATGCATGGTAGGCGCTACACATGGTCCAGCGAGCGCAAGATTTCCTACTCTTGTCAAGAACAA
PI 511381        TTTGTATGCATGGTAGGCGCTACACATGGTCCAGCGAGCGCAAGATTTCCTACTCTTGTCAAGAACAA
PI 486271        TTTGTATGCATGGTAGGCGCTACACATGGTCCAGCGAGCGCAAGATTTCCTACTCTTGTCAAGAACAA
PI 511367        TTTGTATGCATGGTAGGCGCTACACATGGTCCAGCGAGCGCAAGATTTCCTACTCTTGTCAAGAACAA
Chinese Spring   TTTGTATGCATGGTAGGCGCT--ACATGGTCCAGCGAGCGCAAGA-TTCCTACTCTTGTCAAGAACAA
Hanzhongbai      TTTGTATGCATGGTAGGCGCT--ACATGGTCCAGCGAGCGCAAGA-TTCCTACTCTTGTCAAGAACAA
Hongtoumai       TTTGTATGCATGGTAGGCGCT--ACATGGTCCAGCGAGCGCAAGA-TTCCTACTCTTGTCAAGAACAA
Hongyouzi        TTTGTATGCATGGTAGGCGCT--ACATGGTCCAGCGAGCGCAAGA-TTCCTACTCTTGTCAAGAACAA
Zijiehong        TTTGTATGCATGGTAGGCGCT--ACATGGTCCAGCGAGCGCAAGA-TTCCTACTCTTGTCAAGAACAA
Baihuamai        TTTGTATGCATGGTAGGCGCT--ACATGGTCCAGCGAGCGCAAGA-TTCCTACTCTTGTCAAGAACAA
Huixianhong      TTTGTATGCATGGTAGGCGCT--ACATGGTCCAGCGAGCGCAAGA-TTCCTACTCTTGTCAAGAACAA
Baimangmai       TTTGTATGCATGGTAGGCGCT--ACATGGTCCAGCGAGCGCAAGA-TTCCTACTCTTGTCAAGAACAA
```

	2,180 · 2,190 · 2,200 · 2,210 · 2,220 · 2,230 · 2,240
Hulutou	CCGCATCGTCTCCTCCTCCTCGAAGGATGCGCATCCCCACTGCCTGCTGTAGTGCCTCTATTCAGCTG
Baihulu	CCGCATCGTCTCCTCCTCCTCGAAGGATGCGCATCCCCACTGCCTGCTGTAGTGCCTCTATTCAGCTG
Chiyacao	CCGCATCGTCTCCTCCTCCTCGAAGGATGCGCATCCCCACTGCCTGCTGTAGTGCCTCTATTCAGCTG
Hongmangmai	CCGCATCGTCTCCTCCTCCTCGAAGGATGCGCATCCCCACTGCCTGCTGTAGTGCCTCTATTCAGCTG
Hongmai	CCGCATCGTCTCCTCCTCCTCGAAGGATGCGCATCCCCACTGCCTGCTGTAGTGCCTCTATTCAGCTG
Wangshuibai	CCGCATCGTCTCCTCCTCCTCGAAGGATGCGCATCCCCACTGCCTGCTGTAGTGCCTCTATTCAGCTG
Shi 4185	CCGCATCGTCTCCTCCTCCTCGAAGGATGCGCATCCCCACTGCCTGCTGTAGTGCCTCTATTCAGCTG
Mazhamai	CCGCATCGTCTCCTCCTCCTCGAAGGATGCGCATCCCCACTGCCTGCTGTAGTGCCTCTATTCAGCTG
Xiaofoshou	CCGCATCGTCTCCTCCTCCTCGAAGGATGCGCATCCCCACTGCCTGCTGTAGTGCCTCTATTCAGCTG
Aikang 58	CCGCATCGTCTCCTCCTCCTCGAAGGATGCGCATCCCCACTGCCTGCTGTAGTGCCTCTATTCAGCTG
AL8/78	CCGCATCGTCTCCTCCTCCTCGAAGGATGCGCATCCCCACTGCCTGCTGTAGTGCCTCTATTCAGCTG
PI 431602	CCGCATCGTCTCCTCCTCCTCGAAGGATGCGCATCCCCACTGCCTGCTGTAGTGCCTCTATTCAGCTG
PI 486274	CCGCATCGTCTCCTCCTCCTCGAAGGATGCGCATCCCCACTGCCTGCTGTAGTGCCTCTATTCAGCTG
RM 000182	CCGCATCGTCTCCTCCTCCTCGAAGGATGCGCATCCCCACTGCCTGCTGTAGTGCCTCTATTCAGCTG
PI 511381	CCGCATCGTCTCCTCCTCCTCGAAGGATGCGCATCCCCACTGCCTGCTGTAGTGCCTCTATTCAGCTG
PI 486271	CCGCATCGTCTCCTCCTCCTCGAAGGATGCGCATCCCCACTGCCTGCTGTAGTGCCTCTATTCAGCTG
PI 511367	CCGCATCGTCTCCTCCTCCTCGAAGGATGCGCATCCCCACTGCCTGCTGTAGTGCCTCTATTCAGCTG
Chinese Spring	CCGCATCGTCTCCTCCTCCTCGAAGGATGCACATCCCCACTGCCTGTTGTAGTGCCTCTATTCAGCTG
Hanzhongbai	CCGCATCGTCTCCTCCTCCTCGAAGGATGCACATCCCCACTGCCTGTTGTAGTGCCTCTATTCAGCTG
Hongtoumai	CCGCATCGTCTCCTCCTCCTCGAAGGATGCACATCCCCACTGCCTGTTGTAGTGCCTCTATTCAGCTG
Hongyouzi	CCGCATCGTCTCCTCCTCCTCGAAGGATGCACATCCCCACTGCCTGTTGTAGTGCCTCTATTCAGCTG
Zijiehong	CCGCATCGTCTCCTCCTCCTCGAAGGATGCACATCCCCACTGCCTGTTGTAGTGCCTCTATTCAGCTG
Baihuamai	CCGCATCGTCTCCTCCTCCTCGAAGGATGCACATCCCCACTGCCTGTTGTAGTGCCTCTATTCAGCTG
Huixianhong	CCGCATCGTCTCCTCCTCCTCGAAGGATGCACATCCCCACTGCCTGTTGTAGTGCCTCTATTCAGCTG
Baimangmai	CCGCATCGTCTCCTCCTCCTCGAAGGATGCACATCCCCACTGCCTGTTGTAGTGCCTCTATTCAGCTG

	2,250 · 2,260 · 2,270 · 2,280 · 2,290 · 2,300 · 2,310
Hulutou	CTTCAGAGCACTGGCCGCTCCTTCTTGATTGTGTCCCGTGTACCCATGGGATCAAGCGGTTCCACTTC
Baihulu	CTTCAGAGCACTGGCCGCTCCTTCTTGATTGTGTCCCGTGTACCCATGGGATCAAGCGGTTCCACTTC
Chiyacao	CTTCAGAGCACTGGCCGCTCCTTCTTGATTGTGTCCCGTGTACCCATGGGATCAAGCGGTTCCACTTC
Hongmangmai	CTTCAGAGCACTGGCCGCTCCTTCTTGATTGTGTCCCGTGTACCCATGGGATCAAGCGGTTCCACTTC
Hongmai	CTTCAGAGCACTGGCCGCTCCTTCTTGATTGTGTCCCGTGTACCCATGGGATCAAGCGGTTCCACTTC
Wangshuibai	CTTCAGAGCACTGGCCGCTCCTTCTTGATTGTGTCCCGTGTACCCATGGGATCAAGCGGTTCCACTTC
Shi 4185	CTTCAGAGCACTGGCCGCTCCTTCTTGATTGTGTCCCGTGTACCCATGGGATCAAGCGGTTCCACTTC
Mazhamai	CTTCAGAGCACTGGCCGCTCCTTCTTGATTGTGTCCCGTGTACCCATGGGATCAAGCGGTTCCACTTC
Xiaofoshou	CTTCAGAGCACTGGCCGCTCCTTCTTGATTGTGTCCCGTGTACCCATGGGATCAAGCGGTTCCACTTC
Aikang 58	CTTCAGAGCACTGGCCGCTCCTTCTTGATTGTGTCCCGTGTACCCATGGGATCAAGCGGTTCCACTTC
AL8/78	CTTCAGAGCACTGGCCGCTCCTTCTTGATTGTGTCCCGTGTACCCATGGGATCAAGCGGTTCCACTTC
PI 431602	CTTCAGAGCACTGGCCGCTCCTTCTTGATTGTGTCCCGTGTACCCATGGGATCAAGCGGTTCCACTTC
PI 486274	CTTCAGAGCACTGGCCGCTCCTTCTTGATTGTGTCCCGTGTACCCATGGGATCAAGCGGTTCCACTTC
RM 000182	CTTCAGAGCACTGGCCGCTCCTTCTTGATTGTGTCCCGTGTACCCATGGGATCAAGCGGTTCCACTTC
PI 511381	CTTCAGAGCACTGGCCGCTCCTTCTTGATTGTGTCCCGTGTACCCATGGGATCAAGCGGTTCCACTTC
PI 486271	CTTCAGAGCACTGGCCGCTCCTTCTTGATTGTGTCCCGTGTACCCATGGGATCAAGCGGTTCCACTTC
PI 511367	CTTCAGAGCACTGGCCGCTCCTTCTTGATTGTGTCCCGTGTACCCATGGGATCAAGCGGTTCCACTTC
Chinese Spring	CTTCAGAGCACTGGCCGCTCCTTCTTGATTGTGTCCCGTGTACCCATGGGATCAAGCGGTTCCACTTC
Hanzhongbai	CTTCAGAGCACTGGCCGCTCCTTCTTGATTGTGTCCCGTGTACCCATGGGATCAAGCGGTTCCACTTC
Hongtoumai	CTTCAGAGCACTGGCCGCTCCTTCTTGATTGTGTCCCGTGTACCCATGGGATCAAGCGGTTCCACTTC
Hongyouzi	CTTCAGAGCACTGGCCGCTCCTTCTTGATTGTGTCCCGTGTACCCATGGGATCAAGCGGTTCCACTTC
Zijiehong	CTTCAGAGCACTGGCCGCTCCTTCTTGATTGTGTCCCGTGTACCCATGGGATCAAGCGGTTCCACTTC
Baihuamai	CTTCAGAGCACTGGCCGCTCCTTCTTGATTGTGTCCCGTGTACCCATGGGATCAAGCGGTTCCACTTC
Huixianhong	CTTCAGAGCACTGGCCGCTCCTTCTTGATTGTGTCCCGTGTACCCATGGGATCAAGCGGTTCCACTTC
Baimangmai	CTTCAGAGCACTGGCCGCTCCTTCTTGATTGTGTCCCGTGTACCCATGGGATCAAGCGGTTCCACTTC

```
                       2,320      2,330      2,340      2,350      2,360      2,370    2,380
Hulutou          GAGTGATTTTGGCCGAAGCTTGATGATTTCCAACTAGTCGCCTCTGAGGCTTGGAACTCCATTGAGCA
Baihulu          GAGTGATTTTGGCCGAAGCTTGATGATTTCCAACTAGTCGCCTCTGAGGCTTGGAACTCCATTGAGCA
Chiyacao         GAGTGATTTTGGCCGAAGCTTGATGATTTCCAACTAGTCGCCTCTGAGGCTTGGAACTCCATTGAGCA
Hongmangmai      GAGTGATTTTGGCCGAAGCTTGATGATTTCCAACTAGTCGCCTCTGAGGCTTGGAACTCCATTGAGCA
Hongmai          GAGTGATTTTGGCCGAAGCTTGATGATTTCCAACTAGTCGCCTCTGAGGCTTGGAACTCCATTGAGCA
Wangshuibai      GAGTGATTTTGGCCGAAGCTTGATGATTTCCAACTAGTCGCCTCTGAGGCTTGGAACTCCATTGAGCA
Shi 4185         GAGTGATTTTGGCCGAAGCTTGATGATTTCCAACTAGTCGCCTCTGAGGCTTGGAACTCCATTGAGCA
Mazhamai         GAGTGATTTTGGCCGAAGCTTGATGATTTCCAACTAGTCGCCTCTGAGGCTTGGAACTCCATTGAGCA
Xiaofoshou       GAGTGATTTTGGCCGAAGCTTGATGATTTCCAACTAGTCGCCTCTGAGGCTTGGAACTCCATTGAGCA
Aikang 58        GAGTGATTTTGGCCGAAGCTTGATGATTTCCAACTAGTCGCCTCTGAGGCTTGGAACTCCATTGAGCA
AL8/78           GAGTGATTTTGGCCGAAGCTTGATGATTTCCAACTAGTCGCCTCTGAGGCTTGGAACTCCATTGAGCA
PI 431602        GAGTGATTTTGGCCGAAGCTTGATGATTTCCAACTAGTCGCCTCTGAGGCTTGGAACTCCATTGAGCA
PI 486274        GAGTGATTTTGGCCGAAGCTTGATGATTTCCAACTAGTCGCCTCTGAGGCTTGGAACTCCATTGAGCA
RM 000182        GAGTGATTTTGGCTGAAGCTTGATGATTTCCAACTAGTCGCCTCTGAGGCTTGGAACTCCATTGAGCA
PI 511381        GAGTGATTTTGGCTGAAGCTTGATGATTTCCAACTAGTCGCCTCTGAGGCTTGGAACTCCATTGAGCA
PI 486271        GAGTGATTTTGGCTGAAGCTTGATGATTTCCAACTAGTCGCCTCTGAGGCTTGGAACTCCATTGAGCA
PI 511367        GAGTGATTTTGGCTGAAGCTTGATGATTTCCAACTAGTCGCCTCTGAGGCTTGGAACTCCATTGAGCA
Chinese Spring   GAGTGATTTTGGCAGAAGCTTGATGATTTCCAACTAGTCGCCTCTGAGGCTTGGAACTCCATTGAGCA
Hanzhongbai      GAGTGATTTTGGCAGAAGCTTGATGATTTCCAACTAGTCGCCTCTGAGGCTTGGAACTCCATTGAGCA
Hongtoumai       GAGTGATTTTGGCAGAAGCTTGATGATTTCCAACTAGTCGCCTCTGAGGCTTGGAACTCCATTGAGCA
Hongyouzi        GAGTGATTTTGGCAGAAGCTTGATGATTTCCAACTAGTCGCCTCTGAGGCTTGGAACTCCATTGAGCA
Zijiehong        GAGTGATTTTGGCAGAAGCTTGATGATTTCCAACTAGTCGCCTCTGAGGCTTGGAACTCCATTGAGCA
Baihuamai        GAGTGATTTTGGCAGAAGCTTGATGATTTCCAACTAGTCGCCTCTGAGGCTTGGAACTCCATTGAGCA
Huixianhong      GAGTGATTTTGGCAGAAGCTTGATGATTTCCAACTAGTCGCCTCTGAGGCTTGGAACTCCATTGAGCA
Baimangmai       GAGTGATTTTGGCAGAAGCTTGATGATTTCCAACTAGTCGCCTCTGAGGCTTGGAACTCCATTGAGCA
```

```
                         2,390      2,400      2,410      2,420      2,430      2,440
Hulutou          TGCCCCTGATGCATTTCGCCATATCGTCGTGCGCCTTAAGTCTACCACACGTCGTCTCCAGAGCTGGA
Baihulu          TGCCCCTGATGCATTTCGCCATATCGTCGTGCGCCTTAAGTCTACCACACGTCGTCTCCAGAGCTGGA
Chiyacao         TGCCCCTGATGCATTTCGCCATATCGTCGTGCGCCTTAAGTCTACCACACGTCGTCTCCAGAGCTGGA
Hongmangmai      TGCCCCTGATGCATTTCGCCATATCGTCGTGCGCCTTAAGTCTACCACACGTCGTCTCCAGAGCTGGA
Hongmai          TGCCCCTGATGCATTTCGCCATATCGTCGTGCGCCTTAAGTCTACCACACGTCGTCTCCAGAGCTGGA
Wangshuibai      TGCCCCTGATGCATTTCGCCATATCGTCGTGCGCCTTAAGTCTACCACACGTCGTCTCCAGAGCTGGA
Shi 4185         TGCCCCTGATGCATTTCGCCATATCGTCGTGCGCCTTAAGTCTACCACACGTCGTCTCCAGAGCTGGA
Mazhamai         TGCCCCTGATGCATTTCGCCATATCGTCGTGCGCCTTAAGTCTACCACACGTCGTCTCCAGAGCTGGA
Xiaofoshou       TGCCCCTGATGCATTTCGCCATATCGTCGTGCGCCTTAAGTCTACCACACGTCGTCTCCAGAGCTGGA
Aikang 58        TGCCCCTGATGCATTTCGCCATATCGTCGTGCGCCTTAAGTCTACCACACGTCGTCTCCAGAGCTGGA
AL8/78           TGCCCCTGATGCATTTCGCCATATCGTCGTGCGCCTTAAGTCTACCACACGTCGTCTCCAGAGCTGGA
PI 431602        TGCCCCTGATGCATTTCGCCATATCGTCGTGCGCCTTAAGTCTACCACACGTCGTCTCCAGAGCTGGA
PI 486274        TGCCCCTGATGCATTTCGCCATATCGTCGTGCGCCTTAAGTCTACCACACGTCGTCTCCAGAGCTGGA
RM 000182        TGCCCCTGATGCATTTCGCCATATCGTCGTGCGCCTTAAGTCTACCACACGTCGTCTCCAGAGCTGGA
PI 511381        TGCCCCTGATGCATTTCGCCATATCGTCGTGCGCCTTAAGTCTACCACACGTCGTCTCCAGAGCTGGA
PI 486271        TGCCCCTGATGCATTTCGCCATATCGTCGTGCGCCTTAAGTCTACCACACGTCGTCTCCAGAGCTGGA
PI 511367        TGCCCCTGATGCATTTCGCCATATCGTCGTGCGCCTTAAGTCTACCACACGTCGTCTCCAGAGCTGGA
Chinese Spring   TGCCCCTGATGCATTTCGCCATATCGTCGTGTGCCTTAAGTCTACCACACGTCGTCTCCAGAGCTGGA
Hanzhongbai      TGCCCCTGATGCATTTCGCCATATCGTCGTGTGCCTTAAGTCTACCACACGTCGTCTCCAGAGCTGGA
Hongtoumai       TGCCCCTGATGCATTTCGCCATATCGTCGTGTGCCTTAAGTCTACCACACGTCGTCTCCAGAGCTGGA
Hongyouzi        TGCCCCTGATGCATTTCGCCATATCGTCGTGTGCCTTAAGTCTACCACACGTCGTCTCCAGAGCTGGA
Zijiehong        TGCCCCTGATGCATTTCGCCATATCGTCGTGTGCCTTAAGTCTACCACACGTCGTCTCCAGAGCTGGA
Baihuamai        TGCCCCTGATGCATTTCGCCATATCGTCGTGTGCCTTAAGTCTACCACACGTCGTCTCCAGAGCTGGA
Huixianhong      TGCCCCTGATGCATTTCGCCATATCGTCGTGTGCCTTAAGTCTACCACACGTCGTCTCCAGAGCTGGA
Baimangmai       TGCCCCTGATGCATTTCGCCATATCGTCGTGTGCCTTAAGTCTACCACACGTCGTCTCCAGAGCTGGA
```

	2,450 · 2,460 · 2,470 · 2,480 · 2,490 · 2,500 · 2,510
Hulutou	GTGCCAAGTCTATTGGCCAAATCTCGGTGCAGCTTCAGATCGCGTGTGAGCTCATTGCTCATTTGGAC
Baihulu	GTGCCAAGTCTATTGGCCAAATCTCGGTGCAGCTTCAGATCGCGTGTGAGCTCATTGCTCATTTGGAC
Chiyacao	GTGCCAAGTCTATTGGCCAAATCTCGGTGCAGCTTCAGATCGCGTGTGAGCTCATTGCTCATTTGGAC
Hongmangmai	GTGCCAAGTCTATTGGCCAAATCTCGGTGCAGCTTCAGATCGCGTGTGAGCTCATTGCTCATTTGGAC
Hongmai	GTGCCAAGTCTATTGGCCAAATCTCGGTGCAGCTTCAGATCGCGTGTGAGCTCATTGCTCATTTGGAC
Wangshuibai	GTGCCAAGTCTATTGGCCAAATCTCGGTGCAGCTTCAGATCGCGTGTGAGCTCATTGCTCATTTGGAC
Shi 4185	GTGCCAAGTCTATTGGCCAAATCTCGGTGCAGCTTCAGATCGCGTGTGAGCTCATTGCTCATTTGGAC
Mazhamai	GTGCCAAGTCTATTGGCCAAATCTCGGTGCAGCTTCAGATCGCGTGTGAGCTCATTGCTCATTTGGAC
Xiaofoshou	GTGCCAAGTCTATTGGCCAAATCTCGGTGCAGCTTCAGATCGCGTGTGAGCTCATTGCTCATTTGGAC
Aikang 58	GTGCCAAGTCTATTGGCCAAATCTCGGTGCAGCTTCAGATCGCGTGTGAGCTCATTGCTCATTTGGAC
AL8/78	GTGCCAAGTCTATTGGCCAAATCTCGGTGCAGCTTCAGATCGCGTGTGAGCTCATTGCTCATTTGGAC
PI 431602	GTGCCAAGTCTATTGGCCAAATCTCGGTGCAGCTTCAGATCGCGTGTGAGCTCATTGCTCATTTGGAC
PI 486274	GTGCCAAGTCTATTGGCCAAATCTCGGTGCAGCTTCAGATCGCGTGTGAGCTCATTGCTCATTTGGAC
RM 000182	GTGCCAAGTCTATTGGCCAAATCTCGGTGCAGCTTCAGATCGCGTGTGAGCTCATTGCTCATTTGGAC
PI 511381	GTGCCAAGTCTATTGGCCAAATCTCGGTGCAGCTTCAGATCGCGTGTGAGCTCATTGCTCATTTGGAC
PI 486271	GTGCCAAGTCTATTGGCCAAATCTCGGTGCAGCTTCAGATCGCGTGTGAGCTCATTGCTCATTTGGAC
PI 511367	GTGCCAAGTCTATTGGCCAAATCTCGGTGCAGCTTCAGATCGCGTGTGAGCTCATTGCTCATTTGGAC
Chinese Spring	GTGCCAAGTCTATTGGCCAAATCTCGGTGCAGCTTCAGATCGCGTGTGAGCTCATTGCTCATTTGGAC
Hanzhongbai	GTGCCAAGTCTATTGGCCAAATCTCGGTGCAGCTTCAGATCGCGTGTGAGCTCATTGCTCATTTGGAC
Hongtoumai	GTGCCAAGTCTATTGGCCAAATCTCGGTGCAGCTTCAGATCGCGTGTGAGCTCATTGCTCATTTGGAC
Hongyouzi	GTGCCAAGTCTATTGGCCAAATCTCGGTGCAGCTTCAGATCGCGTGTGAGCTCATTGCTCATTTGGAC
Zijiehong	GTGCCAAGTCTATTGGCCAAATCTCGGTGCAGCTTCAGATCGCGTGTGAGCTCATTGCTCATTTGGAC
Baihuamai	GTGCCAAGTCTATTGGCCAAATCTCGGTGCAGCTTCAGATCGCGTGTGAGCTCATTGCTCATTTGGAC
Huixianhong	GTGCCAAGTCTATTGGCCAAATCTCGGTGCAGCTTCAGATCGCGTGTGAGCTCATTGCTCATTTGGAC
Baimangmai	GTGCCAAGTCTATTGGCCAAATCTCGGTGCAGCTTCAGATCGCGTGTGAGCTCATTGCTCATTTGGAC

	2,520 · 2,530 · 2,540 · 2,550 · 2,560 · 2,570 · 2,580
Hulutou	GCCGCTCAGCACTTTCGGCCGCTCTCGACCTCGGAGGCCTGGTTGCGACATAGGCTGAAGGCCGCCTA
Baihulu	GCCGCTCAGCACTTTCGGCCGCTCTCGACCTCGGAGGCCTGGTTGCGACATAGGCTGAAGGCCGCCTA
Chiyacao	GCCGCTCAGCACTTTCGGCCGCTCTCGACCTCGGAGGCCTGGTTGCGACATAGGCTGAAGGCCGCCTA
Hongmangmai	GCCGCTCAGCACTTTCGGCCGCTCTCGACCTCGGAGGCCTGGTTGCGACATAGGCTGAAGGCCGCCTA
Hongmai	GCCGCTCAGCACTTTCGGCCGCTCTCGACCTCGGAGGCCTGGTTGCGACATAGGCTGAAGGCCGCCTA
Wangshuibai	GCCGCTCAGCACTTTCGGCCGCTCTCGACCTCGGAGGCCTGGTTGCGACATAGGCTGAAGGCCGCCTA
Shi 4185	GCCGCTCAGCACTTTCGGCCGCTCTCGACCTCGGAGGCCTGGTTGCGACATAGGCTGAAGGCCGCCTA
Mazhamai	GCCGCTCAGCACTTTCGGCCGCTCTCGACCTCGGAGGCCTGGTTGCGACATAGGCTGAAGGCCGCCTA
Xiaofoshou	GCCGCTCAGCACTTTCGGCCGCTCTCGACCTCGGAGGCCTGGTTGCGACATAGGCTGAAGGCCGCCTA
Aikang 58	GCCGCTCAGCACTTTCGGCCGCTCTCGACCTCGGAGGCCTGGTTGCGACATAGGCTGAAGGCCGCCTA
AL8/78	GCCGCTCAGCACTTTCGGCCGCTCTCGACCTCGGAGGCCTAGTTGCGACATAGGCTGAAGGCCGCCTA
PI 431602	GCCGCTCAGCACTTTCGGCCGCTCTCGACCTCGGAGGCCTAGTTGCGACATAGGCTGAAGGCCGCCTA
PI 486274	GCCGCTCAGCACTTTCGGCCGCTCTCGACCTCGGAGGCCTAGTTGCGACATAGGCTGAAGGCCGCCTA
RM 000182	GCCGCTCAGCACTTTCGGCCGCTCTCGACCTCGGAGGCCTGGTTGCGACATAGGCTGAAGGCCGCCTA
PI 511381	GCCGCTCAGCACTTTCGGCCGCTCTCGACCTCGGAGGCCTGGTTGCGACATAGGCTGAAGGCCGCCTA
PI 486271	GCCGCTCAGCACTTTCGGCCGCTCTCGACCTCGGAGGCCTGGTTGCGACATAGGCTGAAGGCCGCCTA
PI 511367	GCCGCTCAGCACTTTCGGCCGCTCTCGACCTCGGAGGCCTGGTTGCGACATAGGCTGAAGGCCGCCTA
Chinese Spring	GCCGCTCAGCACTTTCTGCCGCTCTCGACCTCGGAGGCCTGGTTGCGACATAGGCTGAAGGCCGCCTA
Hanzhongbai	GCCGCTCAGCACTTTCTGCCGCTCTCGACCTCGGAGGCCTGGTTGCGACATAGGCTGAAGGCCGCCTA
Hongtoumai	GCCGCTCAGCACTTTCTGCCGCTCTCGACCTCGGAGGCCTGGTTGCGACATAGGCTGAAGGCCGCCTA
Hongyouzi	GCCGCTCAGCACTTTCTGCCGCTCTCGACCTCGGAGGCCTGGTTGCGACATAGGCTGAAGGCCGCCTA
Zijiehong	GCCGCTCAGCACTTTCTGCCGCTCTCGACCTCGGAGGCCTGGTTGCGACATAGGCTGAAGGCCGCCTA
Baihuamai	GCCGCTCAGCACTTTCTGCCGCTCTCGACCTCGGAGGCCTGGTTGCGACATAGGCTGAAGGCCGCCTA
Huixianhong	GCCGCTCAGCACTTTCTGCCGCTCTCGACCTCGGAGGCCTGGTTGCGACATAGGCTGAAGGCCGCCTA
Baimangmai	GCCGCTCAGCACTTTCTGCCGCTCTCGACCTCGGAGGCCTGGTTGCGACATAGGCTGAAGGCCGCCTA

	2,590 – 2,650
Hulutou	CCTTGGCCTTGCCTCGCTCGAGTGTTCCATCATTCGGCAACGCACGAGGTTTGCCTACGAGGTTTGCC
Baihulu	CCTTGGCCTTGCCTCGCTCGAGTGTTCCATCATTCGGCAACGCACGAGGTTTGCCTACGAGGTTTGCC
Chiyacao	CCTTGGCCTTGCCTCGCTCGAGTGTTCCATCATTCGGCAACGCACGAGGTTTGCCTACGAGGTTTGCC
Hongmangmai	CCTTGGCCTTGCCTCGCTCGAGTGTTCCATCATTCGGCAACGCACGAGGTTTGCCTACGAGGTTTGCC
Hongmai	CCTTGGCCTTGCCTCGCTCGAGTGTTCCATCATTCGGCAACGCACGAGGTTTGCCTACGAGGTTTGCC
Wangshuibai	CCTTGGCCTTGCCTCGCTCGAGTGTTCCATCATTCGGCAACGCACGAGGTTTGCCTACGAGGTTTGCC
Shi 4185	CCTTGGCCTTGCCTCGCTCGAGTGTTCCATCATTCGGCAACGCACGAGGTTTGCCTACGAGGTTTGCC
Mazhamai	CCTTGGCCTTGCCTCGCTCGAGTGTTCCATCATTCGGCAACGCACGAGGTTTGCCTACGAGGTTTGCC
Xiaofoshou	CCTTGGCCTTGCCTCGCTCGAGTGTTCCATCATTCGGCAACGCACGAGGTTTGCCTACGAGGTTTGCC
Aikang 58	CCTTGGCCTTGCCTCGCTCGAGTGTTCCATCATTCGGCAACGCACGAGGTTTGCCTACGAGGTTTGCC
AL8/78	CCTTGGCCTTGCCTCGCTCGAGTGTTCCATCATTCGGCAGCGC-------------ACGAGGTTTGCC
PI 431602	CCTTGGCCTTGCCTCGCTCGAGTGTTCCATCATTCGGCAGCGC-------------ACGAGGTTTGCC
PI 486274	CCTTGGCCTTGCCTCGCTCGAGTGTTCCATCATTCGGCAGCGC-------------ACGAGGTTTGCC
RM 000182	CCTTGGCCTTGCCTCGCTCGAGTGTTCCATCATTCGGCAGCGC-------------GCGAGGTTTGCC
PI 511381	CCTTGGCCTTGCCTCGCTCGAGTGTTCCATCATTCGGCAGCGC-------------GCGAGGTTTGCC
PI 486271	CCTTGGCCTTGCCTCGCTCGAGTGTTCCATCATTCGGCAGCGC-------------GCGAGGTTTGCC
PI 511367	CCTTGGCCTTGCCTCGCTCGAGTGTTCCATCATTCGGCAGCGC-------------GCGAGGTTTGCC
Chinese Spring	CCTTGGCCTTGCCTCGCTCGAGTGTTCCATCATTCGGCAGCGC-------------ACGAGGTTTGCC
Hanzhongbai	CCTTGGCCTTGCCTCGCTCGAGTGTTCCATCATTCGGCAGCGC-------------ACGAGGTTTGCC
Hongtoumai	CCTTGGCCTTGCCTCGCTCGAGTGTTCCATCATTCGGCAGCGC-------------ACGAGGTTTGCC
Hongyouzi	CCTTGGCCTTGCCTCGCTCGAGTGTTCCATCATTCGGCAGCGC-------------ACGAGGTTTGCC
Zijiehong	CCTTGGCCTTGCCTCGCTCGAGTGTTCCATCATTCGGCAGCGC-------------ACGAGGTTTGCC
Baihuamai	CCTTGGCCTTGCCTCGCTCGAGTGTTCCATCATTCGGCAGCGC-------------ACGAGGTTTGCC
Huixianhong	CCTTGGCCTTGCCTCGCTCGAGTGTTCCATCATTCGGCAGCGC-------------ACGAGGTTTGCC
Baimangmai	CCTTGGCCTTGCCTCGCTCGAGTGTTCCATCATTCGGCAGCGC-------------ACGAGGTTTGCC

	2,660 – 2,720
Hulutou	TGTCTCAGGGAGGCTGATGCTGGCCCTACCTTCTTCAACATGCATGCATGCCTCGTACCATAAGCAGA
Baihulu	TGTCTCAGGGAGGCTGATGCTGGCCCTACCTTCTTCAACATGCATGCATGCCTCGTACCATAAGCAGA
Chiyacao	TGTCTCAGGGAGGCTGATGCTGGCCCTACCTTCTTCAACATGCATGCATGCCTCGTACCATAAGCAGA
Hongmangmai	TGTCTCAGGGAGGCTGATGCTGGCCCTACCTTCTTCAACATGCATGCATGCCTCGTACCATAAGCAGA
Hongmai	TGTCTCAGGGAGGCTGATGCTGGCCCTACCTTCTTCAACATGCATGCATGCCTCGTACCATAAGCAGA
Wangshuibai	TGTCTCAGGGAGGCTGATGCTGGCCCTACCTTCTTCAACATGCATGCATGCCTCGTACCATAAGCAGA
Shi 4185	TGTCTCAGGGAGGCTGATGCTGGCCCTACCTTCTTCAACATGCATGCATGCCTCGTACCATAAGCAGA
Mazhamai	TGTCTCAGGGAGGCTGATGCTGGCCCTACCTTCTTCAACATGCATGCATGCCTCGTACCATAAGCAGA
Xiaofoshou	TGTCTCAGGGAGGCTGATGCTGGCCCTACCTTCTTCAACATGCATGCATGCCTCGTACCATAAGCAGA
Aikang 58	TGTCTCAGGGAGGCTGATGCTGGCCCTACCTTCTTCAACATGCATGCATGCCTCGTACCATAAGCAGA
AL8/78	TGTCTCAGGGAGGCTGATGTCGACCCTACCTTCTTCAACATGCATGCATGCCTCGTACCATAAGCAGA
PI 431602	TGTCTCAGGGAGGCTGATGTCGACCCTACCTTCTTCAACATGCATGCATGCCTCGTACCATAAGCAGA
PI 486274	TGTCTCAGGGAGGCTGATGTCGACCCTACCTTCTTCAACATGCATGCATGCCTCGTACCATAAGCAGA
RM 000182	TGTCTCAGGGAGGCTGATGCCGGCCCTACCTTCTTCAACATGCATGCATGCCTCGTACCATAAGCAGA
PI 511381	TGTCTCAGGGAGGCTGATGCCGGCCCTACCTTCTTCAACATGCATGCATGCCTCGTACCATAAGCAGA
PI 486271	TGTCTCAGGGAGGCTGATGCCGGCCCTACCTTCTTCAACATGCATGCATGCCTCGTACCATAAGCAGA
PI 511367	TGTCTCAGGGAGGCTGATGCCGGCCCTACCTTCTTCAACATGCATGCATGCCTCGTACCATAAGCAGA
Chinese Spring	TGTCTCAGGGAGGCTGATGCCGGCCCTACCTTCTTCAACATGCATGCATGCCTCGTACCATAAGCAGA
Hanzhongbai	TGTCTCAGGGAGGCTGATGCCGGCCCTACCTTCTTCAACATGCATGCATGCCTCGTACCATAAGCAGA
Hongtoumai	TGTCTCAGGGAGGCTGATGCCGGCCCTACCTTCTTCAACATGCATGCATGCCTCGTACCATAAGCAGA
Hongyouzi	TGTCTCAGGGAGGCTGATGCCGGCCCTACCTTCTTCAACATGCATGCATGCCTCGTACCATAAGCAGA
Zijiehong	TGTCTCAGGGAGGCTGATGCCGGCCCTACCTTCTTCAACATGCATGCATGCCTCGTACCATAAGCAGA
Baihuamai	TGTCTCAGGGAGGCTGATGCCGGCCCTACCTTCTTCAACATGCATGCATGCCTCGTACCATAAGCAGA
Huixianhong	TGTCTCAGGGAGGCTGATGCCGGCCCTACCTTCTTCAACATGCATGCATGCCTCGTACCATAAGCAGA
Baimangmai	TGTCTCAGGGAGGCTGATGCCGGCCCTACCTTCTTCAACATGCATGCATGCCTCGTACCATAAGCAGA

	2,730 – 2,780
Hulutou	AAAACCGCATCTTCGAGCTCAAGGTGGGTGATAGAGTTGTCTCCACGCCGGAGGCGTTTGCCACTGCG
Baihulu	AAAACCGCATCTTCGAGCTCAAGGTGGGTGATAGAGTTGTCTCCACGCCGGAGGCGTTTGCCACTGCG
Chiyacao	AAAACCGCATCTTCGAGCTCAAGGTGGGTGATAGAGTTGTCTCCACGCCGGAGGCGTTTGCCACTGCG
Hongmangmai	AAAACCGCATCTTCGAGCTCAAGGTGGGTGATAGAGTTGTCTCCACGCCGGAGGCGTTTGCCACTGCG
Hongmai	AAAACCGCATCTTCGAGCTCAAGGTGGGTGATAGAGTTGTCTCCACGCCGGAGGCGTTTGCCACTGCG
Wangshuibai	AAAACCGCATCTTCGAGCTCAAGGTGGGTGATAGAGTTGTCTCCACGCCGGAGGCGTTTGCCACTGCG
Shi 4185	AAAACCGCATCTTCGAGCTCAAGGTGGGTGATAGAGTTGTCTCCACGCCGGAGGCGTTTGCCACTGCG
Mazhamai	AAAACCGCATCTTCGAGCTCAAGGTGGGTGATAGAGTTGTCTCCACGCCGGAGGCGTTTGCCACTGCG
Xiaofoshou	AAAACCGCATCTTCGAGCTCAAGGTGGGTGATAGAGTTGTCTCCACGCCGGAGGCGTTTGCCACTGCG
Aikang 58	AAAACCGCATCTTCGAGCTCAAGGTGGGTGATAGAGTTGTCTCCACGCCGGAGGCGTTTGCCACTGCG
AL8/78	AAAACCACATCTTCGAGCTCAAGGTGGGTGATAGAGTTGTCTCCACGCCGGAGGCGTTTGCCACTGCG
PI 431602	AAAACCACATCTTCGAGCTCAAGGTGGGTGATAGAGTTGTCTCCACGCCGGAGGCGTTTGCCACTGCG
PI 486274	AAAACCACATCTTCGAGCTCAAGGTGGGTGATAGAGTTGTCTCCACGCCGGAGGCGTTTGCCACTGCG
RM 000182	AAAACCGCATCTTCGAGCTCAAGGTGGGTGATAGAGTTGTCTCCACGCCGGAGGCGCTTGCCACTGCG
PI 511381	AAAACCGCATCTTCGAGCTCAAGGTGGGTGATAGAGTTGTCTCCACGCCGGAGGCGCTTGCCACTGCG
PI 486271	AAAACCGCATCTTCGAGCTCAAGGTGGGTGATAGAGTTGTCTCCACGCCGGAGGCGCTTGCCACTGCG
PI 511367	AAAACCGCATCTTCGAGCTCAAGGTGGGTGATAGAGTTGTCTCCACGCCGGAGGCGCTTGCCACTGCG
Chinese Spring	AAAACCACATCTTCGAGCTCAAGGTGGGTGATAGAGTTGTCTCCACGCCGGAGGCGTTTGCCACTGCG
Hanzhongbai	AAAACCACATCTTCGAGCTCAAGGTGGGTGATAGAGTTGTCTCCACGCCGGAGGCGTTTGCCACTGCG
Hongtoumai	AAAACCACATCTTCGAGCTCAAGGTGGGTGATAGAGTTGTCTCCACGCCGGAGGCGTTTGCCACTGCG
Hongyouzi	AAAACCACATCTTCGAGCTCAAGGTGGGTGATAGAGTTGTCTCCACGCCGGAGGCGTTTGCCACTGCG
Zijiehong	AAAACCACATCTTCGAGCTCAAGGTGGGTGATAGAGTTGTCTCCACGCCGGAGGCGTTTGCCACTGCG
Baihuamai	AAAACCACATCTTCGAGCTCAAGGTGGGTGATAGAGTTGTCTCCACGCCGGAGGCGTTTGCCACTGCG
Huixianhong	AAAACCACATCTTCGAGCTCAAGGTGGGTGATAGAGTTGTCTCCACGCCGGAGGCGTTTGCCACTGCG
Baimangmai	AAAACCACATCTTCGAGCTCAAGGTGGGTGATAGAGTTGTCTCCACGCCGGAGGCGTTTGCCACTGCG

	2,790 – 2,850
Hulutou	GGATTTTGATCACTTCACCGGTGCCCTTGAGACCTCCGAGGACCGCGACTTCTCCATCTCCCTTCGGG
Baihulu	GGATTTTGATCACTTCACCGGTGCCCTTGAGACCTCCGAGGACCGCGACTTCTCCATCTCCCTTCGGG
Chiyacao	GGATTTTGATCACTTCACCGGTGCCCTTGAGACCTCCGAGGACCGCGACTTCTCCATCTCCCTTCGGG
Hongmangmai	GGATTTTGATCACTTCACCGGTGCCCTTGAGACCTCCGAGGACCGCGACTTCTCCATCTCCCTTCGGG
Hongmai	GGATTTTGATCACTTCACCGGTGCCCTTGAGACCTCCGAGGACCGCGACTTCTCCATCTCCCTTCGGG
Wangshuibai	GGATTTTGATCACTTCACCGGTGCCCTTGAGACCTCCGAGGACCGCGACTTCTCCATCTCCCTTCGGG
Shi 4185	GGATTTTGATCACTTCACCGGTGCCCTTGAGACCTCCGAGGACCGCGACTTCTCCATCTCCCTTCGGG
Mazhamai	GGATTTTGATCACTTCACCGGTGCCCTTGAGACCTCCGAGGACCGCGACTTCTCCATCTCCCTTCGGG
Xiaofoshou	GGATTTTGATCACTTCACCGGTGCCCTTGAGACCTCCGAGGACCGCGACTTCTCCATCTCCCTTCGGG
Aikang 58	GGATTTTGATCACTTCACCGGTGCCCTTGAGACCTCCGAGGACCGCGACTTCTCCATCTCCCTTCGGG
AL8/78	GGATTTTGATCACTTCACCGGTGCCCTTGAGACCTCCGAGGACCGCGACTTCTCCATCTCCCTTCGGG
PI 431602	GGATTTTGATCACTTCACCGGTGCCCTTGAGACCTCCGAGGACCGCGACTTCTCCATCTCCCTTCGGG
PI 486274	GGATTTTGATCACTTCACCGGTGCCCTTGAGACCTCCGAGGACCGCGACTTCTCCATCTCCCTTCGGG
RM 000182	GGATTTTGATCACTTCACCGGTGCCCTTGAGACCTCCGAGGACCGCGACTTCTCCATCTCCCTTCGGG
PI 511381	GGATTTTGATCACTTCACCGGTGCCCTTGAGACCTCCGAGGACCGCGACTTCTCCATCTCCCTTCGGG
PI 486271	GGATTTTGATCACTTCACCGGTGCCCTTGAGACCTCCGAGGACCGCGACTTCTCCATCTCCCTTCGGG
PI 511367	GGATTTTGATCACTTCACCGGTGCCCTTGAGACCTCCGAGGACCGCGACTTCTCCATCTCCCTTCGGG
Chinese Spring	GGATTTTGATCACTTCACCGGTGCCCTTGAGACCTCCGAGGACCGCGACTTCTCCATCTCCCTTCGGG
Hanzhongbai	GGATTTTGATCACTTCACCGGTGCCCTTGAGACCTCCGAGGACCGCGACTTCTCCATCTCCCTTCGGG
Hongtoumai	GGATTTTGATCACTTCACCGGTGCCCTTGAGACCTCCGAGGACCGCGACTTCTCCATCTCCCTTCGGG
Hongyouzi	GGATTTTGATCACTTCACCGGTGCCCTTGAGACCTCCGAGGACCGCGACTTCTCCATCTCCCTTCGGG
Zijiehong	GGATTTTGATCACTTCACCGGTGCCCTTGAGACCTCCGAGGACCGCGACTTCTCCATCTCCCTTCGGG
Baihuamai	GGATTTTGATCACTTCACCGGTGCCCTTGAGACCTCCGAGGACCGCGACTTCTCCATCTCCCTTCGGG
Huixianhong	GGATTTTGATCACTTCACCGGTGCCCTTGAGACCTCCGAGGACCGCGACTTCTCCATCTCCCTTCGGG
Baimangmai	GGATTTTGATCACTTCACCGGTGCCCTTGAGACCTCCGAGGACCGCGACTTCTCCATCTCCCTTCGGG

	2,860 · 2,870 · 2,880 · 2,890 · 2,900 · 2,910 · 2,920
Hulutou	ATCTGCATGCTGGTGCTTTCGATATCAAGGCCCTTGATGAGCCATTTTTTTAGGCCGAAATTTGGCGC
Baihulu	ATCTGCATGCTGGTGCTTTCGATATCAAGGCCCTTGATGAGCCATTTTTTTAGGCCGAAATTTGGCGC
Chiyacao	ATCTGCATGCTGGTGCTTTCGATATCAAGGCCCTTGATGAGCCATTTTTTTAGGCCGAAATTTGGCGC
Hongmangmai	ATCTGCATGCTGGTGCTTTCGATATCAAGGCCCTTGATGAGCCATTTTTTTAGGCCGAAATTTGGCGC
Hongmai	ATCTGCATGCTGGTGCTTTCGATATCAAGGCCCTTGATGAGCCATTTTTTTAGGCCGAAATTTGGCGC
Wangshuibai	ATCTGCATGCTGGTGCTTTCGATATCAAGGCCCTTGATGAGCCATTTTTTTAGGCCGAAATTTGGCGC
Shi 4185	ATCTGCATGCTGGTGCTTTCGATATCAAGGCCCTTGATGAGCCATTTTTTTAGGCCGAAATTTGGCGC
Mazhamai	ATCTGCATGCTGGTGCTTTCGATATCAAGGCCCTTGATGAGCCATTTTTTTAGGCCGAAATTTGGCGC
Xiaofoshou	ATCTGCATGCTGGTGCTTTCGATATCAAGGCCCTTGATGAGCCATTTTTTTAGGCCGAAATTTGGCGC
Aikang 58	ATCTGCATGCTGGTGCTTTCGATATCAAGGCCCTTGATGAGCCATTTTTTTAGGCCGAAATTTGGCGC
AL8/78	ATCTGCATGCTGGTGCTTTCGATATCAAGGCCCTTGATGAGCCAT-TTTTTAGGCCGAAATTTGGCGC
PI 431602	ATCTGCATGCTGGTGCTTTCGATATCAAGGCCCTTGATGAGCCAT-TTTTTAGGCCGAAATTTGGCGC
PI 486274	ATCTGCATGCTGGTGCTTTCGATATCAAGGCCCTTGATGAGCCAT-TTTTTAGGCCGAAATTTGGCGC
RM 000182	ATCTGCATGCTGGTGCTTTCGATATCAAGGCCCTTGATGAGCCATTTTTTTAGGCCGAAATTTGGCGC
PI 511381	ATCTGCATGCTGGTGCTTTCGATATCAAGGCCCTTGATGAGCCATTTTTTTAGGCCGAAATTTGGCGC
PI 486271	ATCTGCATGCTGGTGCTTTCGATATCAAGGCCCTTGATGAGCCATTTTTTTAGGCCGAAATTTGGCGC
PI 511367	ATCTGCATGCTGGTGCTTTCGATATCAAGGCCCTTGATGAGCCATTTTTTTAGGCCGAAATTTGGCGC
Chinese Spring	ATCTGCATGCTGGTGCTTTCGATATCAAGGCCCTTGATGAGCCATTTTTTTAGGCCGAAATTTGGCGC
Hanzhongbai	ATCTGCATGCTGGTGCTTTCGATATCAAGGCCCTTGATGAGCCATTTTTTTAGGCCGAAATTTGGCGC
Hongtoumai	ATCTGCATGCTGGTGCTTTCGATATCAAGGCCCTTGATGAGCCATTTTTTTAGGCCGAAATTTGGCGC
Hongyouzi	ATCTGCATGCTGGTGCTTTCGATATCAAGGCCCTTGATGAGCCATTTTTTTAGGCCGAAATTTGGCGC
Zijiehong	ATCTGCATGCTGGTGCTTTCGATATCAAGGCCCTTGATGAGCCATTTTTTTAGGCCGAAATTTGGCGC
Baihuamai	ATCTGCATGCTGGTGCTTTCGATATCAAGGCCCTTGATGAGCCATTTTTTTAGGCCGAAATTTGGCGC
Huixianhong	ATCTGCATGCTGGTGCTTTCGATATCAAGGCCCTTGATGAGCCATTTTTTTAGGCCGAAATTTGGCGC
Baimangmai	ATCTGCATGCTGGTGCTTTCGATATCAAGGCCCTTGATGAGCCATTTTTTTAGGCCGAAATTTGGCGC

	2,930 · 2,940 · 2,950 · 2,960 · 2,970 · 2,980 · 2,990
Hulutou	GTTGTCAAGGCTCTACCCTCCAGTAAG**A**CGCCGGGGCATCCCTCAATGCGTGAGGCTTTCAAAAACT
Baihulu	GTTGTCAAGGCTCTACCCTCCAGTAAG**A**CGCCGGGGCATCCCTCAATGCGTGAGGCTTTCAAAAACT
Chiyacao	GTTGTCAAGGCTCTACCCTCCAGTAAG**A**CGCCGGGGCATCCCTCAATGCGTGAGGCTTTCAAAAACT
Hongmangmai	GTTGTCAAGGCTCTACCCTCCAGTAAG**A**CGCCGGGGCATCCCTCAATGCGTGAGGCTTTCAAAAACT
Hongmai	GTTGTCAAGGCTCTACCCTCCAGTAAG**A**CGCCGGGGCATCCCTCAATGCGTGAGGCTTTCAAAAACT
Wangshuibai	GTTGTCAAGGCTCTACCCTCCAGTAAG**A**CGCCGGGGCATCCCTCAATGCGTGAGGCTTTCAAAAACT
Shi 4185	GTTGTCAAGGCTCTACCCTCCAGTAAG**A**CGCCGGGGCATCCCTCAATGCGTGAGGCTTTCAAAAACT
Mazhamai	GTTGTCAAGGCTCTACCCTCCAGTAAG**A**CGCCGGGGCATCCCTCAATGCGTGAGGCTTTCAAAAACT
Xiaofoshou	GTTGTCAAGGCTCTACCCTCCAGTAAG**A**CGCCGGGGCATCCCTCAATGCGTGAGGCTTTCAAAAACT
Aikang 58	GTTGTCAAGGCTCTACCCTCCAGTAAG**A**CGCCGGGGCATCCCTCAATGCGTGAGGCTTTCAAAAACT
AL8/78	GTTGTCAAGGCTCTACCCTCCAGTAAGGCGCCGGGGCATCCCTCAATGCGTGAGGCTTTCAAAAACT
PI 431602	GTTGTCAAGGCTCTACCCTCCAGTAAGGCGCCGGGGCATCCCTCAATGCGTGAGGCTTTCAAAAACT
PI 486274	GTTGTCAAGGCTCTACCCTCCAGTAAGGCGCCGGGGCATCCCTCAATGCGTGAGGCTTTCAAAAACT
RM 000182	GTTGTCAAGGCTCTACCCTCCAGTAAGGCGCCGGGGCATCCCTCA**G**TGCGTGAGGCTTTCAAAAACT
PI 511381	GTTGTCAAGGCTCTACCCTCCAGTAAGGCGCCGGGGCATCCCTCA**G**TGCGTGAGGCTTTCAAAAACT
PI 486271	GTTGTCAAGGCTCTACCCTCCAGTAAGGCGCCGGGGCATCCCTCA**G**TGCGTGAGGCTTTCAAAAACT
PI 511367	GTTGTCAAGGCTCTACCCTCCAGTAAGGCGCCGGGGCATCCCTCA**G**TGCGTGAGGCTTTCAAAAACT
Chinese Spring	GTTGTCAAGGCTCTACCCTCCAGTAAGGCGCCGGGGCATCCCTCAATGCGTGAGGCTTTCAAAAACT
Hanzhongbai	GTTGTCAAGGCTCTACCCTCCAGTAAGGCGCCGGGGCATCCCTCAATGCGTGAGGCTTTCAAAAACT
Hongtoumai	GTTGTCAAGGCTCTACCCTCCAGTAAGGCGCCGGGGCATCCCTCAATGCGTGAGGCTTTCAAAAACT
Hongyouzi	GTTGTCAAGGCTCTACCCTCCAGTAAGGCGCCGGGGCATCCCTCAATGCGTGAGGCTTTCAAAAACT
Zijiehong	GTTGTCAAGGCTCTACCCTCCAGTAAGGCGCCGGGGCATCCCTCAATGCGTGAGGCTTTCAAAAACT
Baihuamai	GTTGTCAAGGCTCTACCCTCCAGTAAGGCGCCGGGGCATCCCTCAATGCGTGAGGCTTTCAAAAACT
Huixianhong	GTTGTCAAGGCTCTACCCTCCAGTAAGGCGCCGGGGCATCCCTCAATGCGTGAGGCTTTCAAAAACT
Baimangmai	GTTGTCAAGGCTCTACCCTCCAGTAAGGCGCCGGGGCATCCCTCAATGCGTGAGGCTTTCAAAAACT

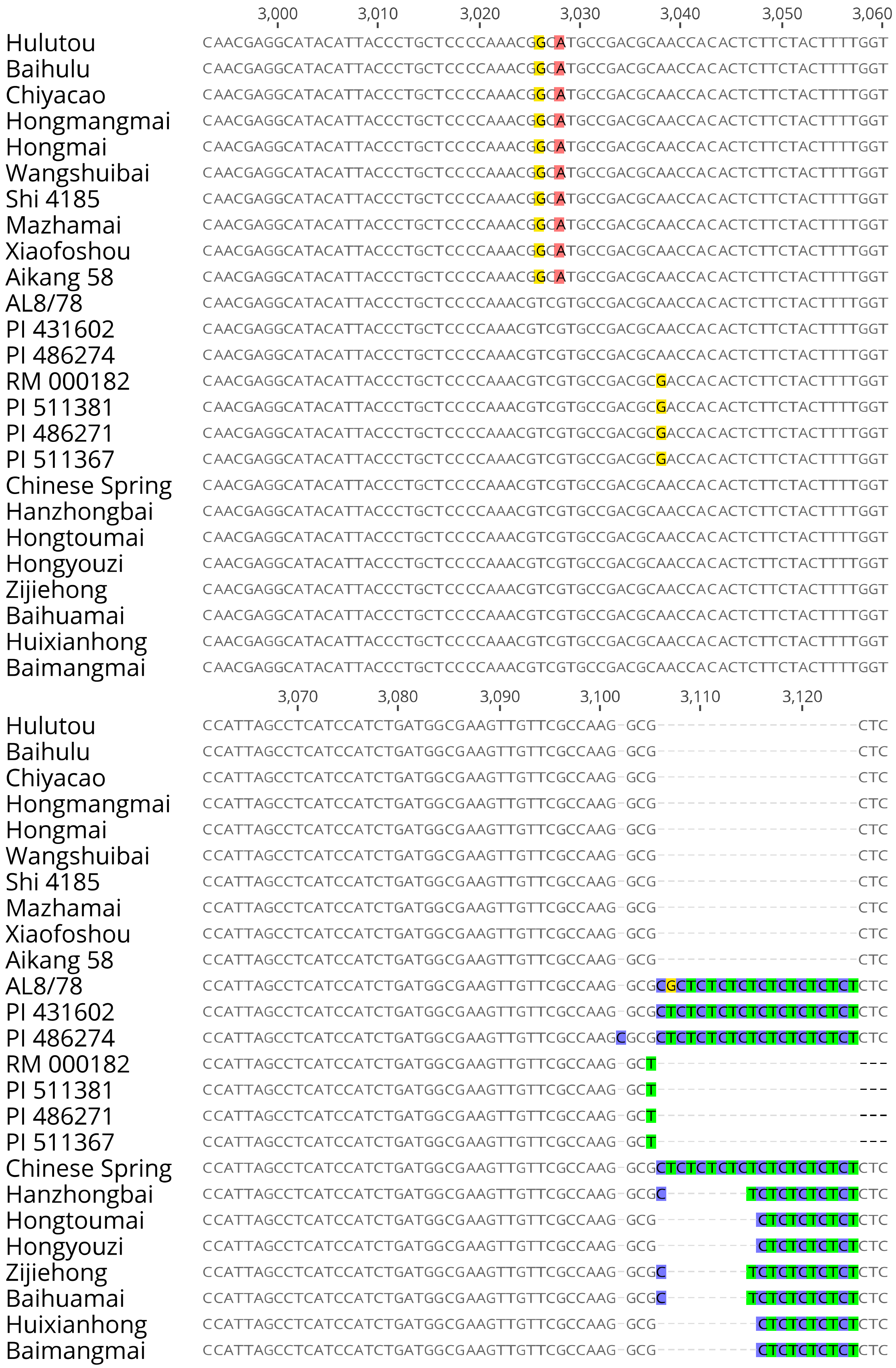

3,000 3,010 3,020 3,030 3,040 3,050 3,060
3,070 3,080 3,090 3,100 3,110 3,120
Hulutou
Baihulu
Chiyacao
Hongmangmai
Hongmai
Wangshuibai
Shi 4185
Mazhamai
Xiaofoshou
Aikang 58
AL8/78
PI 431602
PI 486274
RM 000182
PI 511381
PI 486271
PI 511367
Chinese Spring
Hanzhongbai
Hongtoumai
Hongyouzi
Zijiehong
Baihuamai
Huixianhong
Baimangmai

```
                   3,130         3,140         3,150          3,160          3,170          3,180          3,190
Hulutou            TCTCTCTCTCTCTCTCTCTCTCG--CTCTCTCTCTCTCTCTGCAAACGGCGTGTGTTCTGCACAATCT
Baihulu            TCTCTCTCTCTCTCTCTCTCTCG--CTCTCTCTCTCTCTCTGCAAACGGCGTGTGTTCTGCACAATCT
Chiyacao           TCTCTCTCTCTCTCTCTCTCTCG--CTCTCTCTCTCTCTCTGCAAACGGCGTGTGTTCTGCACAATCT
Hongmangmai        TCTCTCTCTCTCTCTCTCTCTCG--CTCTCTCTCTCTCTCTGCAAACGGCGTGTGTTCTGCACAATCT
Hongmai            TCTCTCTCTCTCTCTCTCTCTCGCTCTCTCTCTCTCTCTCTGCAAACGGCGTGTGTTCTGCACAATCT
Wangshuibai        TCTCTCTCTCTCTCTCTCTCTCG--CTCTCTCTCTCTCTCTGCAAACGGCGTGTGTTCTGCACAATCT
Shi 4185           TCTCTCTCTCTCTCTCTCTCTCG--CTCTCTCTCTCTCTCTGCAAACGGCGTGTGTTCTGCACAATCT
Mazhamai           TCTCTCTCTCTCTCTCTCTCTCG--CTCTCTCTCTCTCTCTGCAAACGGCGTGTGTTCTGCACAATCT
Xiaofoshou         TCTCTCTCTCTCTCTCTCTCTCG--CTCTCTCTCTCTCTCTGCAAACGGCGTGTGTTCTGCACAATCT
Aikang 58          TCTCTCTCTCTCTCTCTCTCTCG--CTCTCTCTCTCTCTCTGCAAACGGCGTGTGTTCTGCACAATCT
AL8/78             TCTCTCTCTCTCTCTCTCTCTCT--CTCTCTCTCTCTCTCTGCAAACGGCGTGTGTTCTGCACAATCT
PI 431602          TCTCTCTCTCTCTCTCTCTCTCT--CTCTCTCTCTCTCTCTGCAAACGGCGTGTGTTCTGCACAATCT
PI 486274          TCTCTCTCTCTCTCTCTCTCTCT--CTCTCTCTCTCTCTCTGCAAACGGCGTGTGTTCTGCACAATCT
RM 000182          ---------------------CT--CTCTCTCTCTCTCTCTACAAACGGCGTGTGTTCTGCACAATCT
PI 511381          ---------------------CT--CTCTCTCTCTCTCTCTACAAACGGCGTGTGTTCTGCACAATCT
PI 486271          ---------------------CT--CTCTCTCTCTCTCTCTACAAACGGCGTGTGTTCTGCACAATCT
PI 511367          ---------------------CT--CTCTCTCTCTCTCTCTACAAACGGCGTGTGTTCTGCACAATCT
Chinese Spring     TCTCTCTCTCTCTCTCTCTCTCT--CTCTCTCTCTCTCTCTGCAAACGGCGTGTGTTCTGCACAATCT
Hanzhongbai        TCTCTCTCTCTCTCTCTCTCTCT--CTCTCTCTCTCTCTCTGCAAACGGCGTGTGTTCTGCACAATCT
Hongtoumai         TCTCTCTCTCTCTCTCTCTCTCT--CTCTCTCTCTCTCTCTGCAAACGGCGTGTGTTCTGCACAATCT
Hongyouzi          TCTCTCTCTCTCTCTCTCTCTCT--CTCTCTCTCTCTCTCTGCAAACGGCGTGTGTTCTGCACAATCT
Zijiehong          TCTCTCTCTCTCTCTCTCTCTCT--CTCTCTCTCTCTCTCTGCAAACGGCGTGTGTTCTGCACAATCT
Baihuamai          TCTCTCTCTCTCTCTCTCTCTCT--CTCTCTCTCTCTCTCTGCAAACGGCGTGTGTTCTGCACAATCT
Huixianhong        TCTCTCTCTCTCTCTCTCTCTCT--CTCTCTCTCTCTCTCTGCAAACGGCGTGTGTTCTGCACAATCT
Baimangmai         TCTCTCTCTCTCTCTCTCTCTCT--CTCTCTCTCTCTCTCTGCAAACGGCGTGTGTTCTGCACAATCT

                      3,200         3,210         3,220          3,230          3,240          3,250          3,260
Hulutou            CAAGGTTCCTCGGGTGATGCTGAAGCTTGACATCGTTAGGGCCTTCGACTCGGTGTCTTAGCCCTTTC
Baihulu            CAAGGTTCCTCGGGTGATGCTGAAGCTTGACATCGTTAGGGCCTTCGACTCGGTGTCTTAGCCCTTTC
Chiyacao           CAAGGTTCCTCGGGTGATGCTGAAGCTTGACATCGTTAGGGCCTTCGACTCGGTGTCTTAGCCCTTTC
Hongmangmai        CAAGGTTCCTCGGGTGATGCTGAAGCTTGACATCGTTAGGGCCTTCGACTCGGTGTCTTAGCCCTTTC
Hongmai            CAAGGTTCCTCGGGTGATGCTGAAGCTTGACATCGTTAGGGCCTTCGACTCGGTGTCTTAGCCCTTTC
Wangshuibai        CAAGGTTCCTCGGGTGATGCTGAAGCTTGACATCGTTAGGGCCTTCGACTCGGTGTCTTAGCCCTTTC
Shi 4185           CAAGGTTCCTCGGGTGATGCTGAAGCTTGACATCGTTAGGGCCTTCGACTCGGTGTCTTAGCCCTTTC
Mazhamai           CAAGGTTCCTCGGGTGATGCTGAAGCTTGACATCGTTAGGGCCTTCGACTCGGTGTCTTAGCCCTTTC
Xiaofoshou         CAAGGTTCCTCGGGTGATGCTGAAGCTTGACATCGTTAGGGCCTTCGACTCGGTGTCTTAGCCCTTTC
Aikang 58          CAAGGTTCCTCGGGTGATGCTGAAGCTTGACATCGTTAGGGCCTTCGACTCGGTGTCTTAGCCCTTTC
AL8/78             CAAGGTTCCTCGGGTGATGCTGAAGCTTGACATCGTTAGGGCCTTCGACTCGGTGTCTTAGCCCTTTC
PI 431602          CAAGGTTCCTCGGGTGATGCTGAAGCTTGACATCGTTAGGGCCTTCGACTCGGTGTCTTAGCCCTTTC
PI 486274          CAAGGTTCCTCGGGTGATGCTGAAGCTTGACATCGTTAGGGCCTTCGACTCGGTGTCTTAGCCCTTTC
RM 000182          CAAGGTTCCTCGGGTGATGCTGAAGCTTGACATCGTTAGGGCCTTCGACTCGGTGTCTTAGCCCTTTC
PI 511381          CAAGGTTCCTCGGGTGATGCTGAAGCTTGACATCGTTAGGGCCTTCGACTCGGTGTCTTAGCCCTTTC
PI 486271          CAAGGTTCCTCGGGTGATGCTGAAGCTTGACATCGTTAGGGCCTTCGACTCGGTGTCTTAGCCCTTTC
PI 511367          CAAGGTTCCTCGGGTGATGCTGAAGCTTGACATCGTTAGGGCCTTCGACTCGGTGTCTTAGCCCTTTC
Chinese Spring     CAAGGTTCCTCGGGTGATGCTGAAGCTTGACATCGTTAGGGCCTTCGACTCGGTGTCTTAGCCCTTTC
Hanzhongbai        CAAGGTTCCTCGGGTGATGCTGAAGCTTGACATCGTTAGGGCCTTCGACTCGGTGTCTTAGCCCTTTC
Hongtoumai         CAAGGTTCCTCGGGTGATGCTGAAGCTTGACATCGTTAGGGCCTTCGACTCGGTGTCTTAGCCCTTTC
Hongyouzi          CAAGGTTCCTCGGGTGATGCTGAAGCTTGACATCGTTAGGGCCTTCGACTCGGTGTCTTAGCCCTTTC
Zijiehong          CAAGGTTCCTCGGGTGATGCTGAAGCTTGACATCGTTAGGGCCTTCGACTCGGTGTCTTAGCCCTTTC
Baihuamai          CAAGGTTCCTCGGGTGATGCTGAAGCTTGACATCGTTAGGGCCTTCGACTCGGTGTCTTAGCCCTTTC
Huixianhong        CAAGGTTCCTCGGGTGATGCTGAAGCTTGACATCGTTAGGGCCTTCGACTCGGTGTCTTAGCCCTTTC
Baimangmai         CAAGGTTCCTCGGGTGATGCTGAAGCTTGACATCGTTAGGGCCTTCGACTCGGTGTCTTAGCCCTTTC
```

	3,270 · 3,280 · 3,290 · 3,300 · 3,310 · 3,320 · 3,330
Hulutou	TTCTGGCCCGAATGTCCCTCTCGTCCGCAGAAAGGGCGTGTGATTCTTGGGCAATGTCAAGGCGAAGG
Baihulu	TTCTGGCCCGAATGTCCCTCTCGTCCGCAGAAAGGGCGTGTGATTCTTGGGCAATGTCAAGGCGAAGG
Chiyacao	TTCTGGCCCGAATGTCCCTCTCGTCCGCAGAAAGGGCGTGTGATTCTTGGGCAATGTCAAGGCGAAGG
Hongmangmai	TTCTGGCCCGAATGTCCCTCTCGTCCGCAGAAAGGGCGTGTGATTCTTGGGCAATGTCAAGGCGAAGG
Hongmai	TTCTGGCCCGAATGTCCCTCTCGTCCGCAGAAAGGGCGTGTGATTCTTGGGCAATGTCAAGGCGAAGG
Wangshuibai	TTCTGGCCCGAATGTCCCTCTCGTCCGCAGAAAGGGCGTGTGATTCTTGGGCAATGTCAAGGCGAAGG
Shi 4185	TTCTGGCCCGAATGTCCCTCTCGTCCGCAGAAAGGGCGTGTGATTCTTGGGCAATGTCAAGGCGAAGG
Mazhamai	TTCTGGCCCGAATGTCCCTCTCGTCCGCAGAAAGGGCGTGTGATTCTTGGGCAATGTCAAGGCGAAGG
Xiaofoshou	TTCTGGCCCGAATGTCCCTCTCGTCCGCAGAAAGGGCGTGTGATTCTTGGGCAATGTCAAGGCGAAGG
Aikang 58	TTCTGGCCCGAATGTCCCTCTCGTCCGCAGAAAGGGCGTGTGATTCTTGGGCAATGTCAAGGCGAAGG
AL8/78	TTCTGGCCCGAATGTCCCTCTCGTC**T**GCAGAAAGGGCGTGTGATTCTTGGGCAATGTCAAGGCGAAGG
PI 431602	TTCTGGCCCGAATGTCCCTCTCGTC**T**GCAGAAAGGGCGTGTGATTCTTGGGCAATGTCAAGGCGAAGG
PI 486274	TTCTGGCCCGAATGTCCCTCTCGTC**T**GCAGAAAGGGCGTGTGATTCTTGGGCAATGTCAAGGCGAAGG
RM 000182	TTCTGGCCCGAATGTCCCTCTCGTCCGCAGAAAGGGCGTG**C**GATTCTTGGGCAATGTCAAGGC**C**AAGG
PI 511381	TTCTGGCCCGAATGTCCCTCTCGTCCGCAGAAAGGGCGTG**C**GATTCTTGGGCAATGTCAAGGC**C**AAGG
PI 486271	TTCTGGCCCGAATGTCCCTCTCGTCCGCAGAAAGGGCGTG**C**GATTCTTGGGCAATGTCAAGGC**C**AAGG
PI 511367	TTCTGGCCCGAATGTCCCTCTCGTCCGCAGAAAGGGCGTG**C**GATTCTTGGGCAATGTCAAGGC**C**AAGG
Chinese Spring	TTCTGGCCCGAATGTCCCTCTCGTCCGCAGAAAGGGCGTGTGATTCTTGGGCAATGTCAAGGCGAAGG
Hanzhongbai	TTCTGGCCCGAATGTCCCTCTCGTCCGCAGAAAGGGCGTGTGATTCTTGGGCAATGTCAAGGCGAAGG
Hongtoumai	TTCTGGCCCGAATGTCCCTCTCGTCCGCAGAAAGGGCGTGTGATTCTTGGGCAATGTCAAGGCGAAGG
Hongyouzi	TTCTGGCCCGAATGTCCCTCTCGTCCGCAGAAAGGGCGTGTGATTCTTGGGCAATGTCAAGGCGAAGG
Zijiehong	TTCTGGCCCGAATGTCCCTCTCGTCCGCAGAAAGGGCGTGTGATTCTTGGGCAATGTCAAGGCGAAGG
Baihuamai	TTCTGGCCCGAATGTCCCTCTCGTCCGCAGAAAGGGCGTGTGATTCTTGGGCAATGTCAAGGCGAAGG
Huixianhong	TTCTGGCCCGAATGTCCCTCTCGTCCGCAGAAAGGGCGTGTGATTCTTGGGCAATGTCAAGGCGAAGG
Baimangmai	TTCTGGCCCGAATGTCCCTCTCGTCCGCAGAAAGGGCGTGTGATTCTTGGGCAATGTCAAGGCGAAGG

	3,340 · 3,350 · 3,360 · 3,370 · 3,380 · 3,390 · 3,400
Hulutou	ATAACGAGAAGGGCTGCATGAAGATGCAACTTTGCCTTGGAGAAGAGGCCCTTACTCCATCTCGCCAA
Baihulu	ATAACGAGAAGGGCTGCATGAAGATGCAACTTTGCCTTGGAGAAGAGGCCCTTACTCCATCTCGCCAA
Chiyacao	ATAACGAGAAGGGCTGCATGAAGATGCAACTTTGCCTTGGAGAAGAGGCCCTTACTCCATCTCGCCAA
Hongmangmai	ATAACGAGAAGGGCTGCATGAAGATGCAACTTTGCCTTGGAGAAGAGGCCCTTACTCCATCTCGCCAA
Hongmai	ATAACGAGAAGGGCTGCATGAAGATGCAACTTTGCCTTGGAGAAGAGGCCCTTACTCCATCTCGCCAA
Wangshuibai	ATAACGAGAAGGGCTGCATGAAGATGCAACTTTGCCTTGGAGAAGAGGCCCTTACTCCATCTCGCCAA
Shi 4185	ATAACGAGAAGGGCTGCATGAAGATGCAACTTTGCCTTGGAGAAGAGGCCCTTACTCCATCTCGCCAA
Mazhamai	ATAACGAGAAGGGCTGCATGAAGATGCAACTTTGCCTTGGAGAAGAGGCCCTTACTCCATCTCGCCAA
Xiaofoshou	ATAACGAGAAGGGCTGCATGAAGATGCAACTTTGCCTTGGAGAAGAGGCCCTTACTCCATCTCGCCAA
Aikang 58	ATAACGAGAAGGGCTGCATGAAGATGCAACTTTGCCTTGGAGAAGAGGCCCTTACTCCATCTCGCCAA
AL8/78	ATAACGAGAAGGGCTGCATGAAGATGCAACTTTGCCTTGGAGAAGAGGCCCTTACTCCATCTCGCCAA
PI 431602	ATAACGAGAAGGGCTGCATGAAGATGCAACTTTGCCTTGGAGAAGAGGCCCTTACTCCATCTCGCCAA
PI 486274	ATAACGAGAAGGGCTGCATGAAGATGCAACTTTGCCTTGGAGAAGAGGCCCTTACTCCATCTCGCCAA
RM 000182	ATAACGAGAAGGGCTGCATGAAGATGCAAC**C**TTGCCTTGGAGAAGAGG**A**CCTTACTCCATCTC**A**CCAA
PI 511381	ATAACGAGAAGGGCTGCATGAAGATGCAAC**C**TTGCCTTGGAGAAGAGG**A**CCTTACTCCATCTC**A**CCAA
PI 486271	ATAACGAGAAGGGCTGCATGAAGATGCAAC**C**TTGCCTTGGAGAAGAGG**A**CCTTACTCCATCTC**A**CCAA
PI 511367	ATAACGAGAAGGGCTGCATGAAGATGCAAC**C**TTGCCTTGGAGAAGAGG**A**CCTTACTCCATCTC**A**CCAA
Chinese Spring	ATAACGAGAAGGGCTGCATGAAGATGCAACTTTGCCTTGGAGAAGAGGCCCTTACTCCATCTCGCCAA
Hanzhongbai	ATAACGAGAAGGGCTGCATGAAGATGCAACTTTGCCTTGGAGAAGAGGCCCTTACTCCATCTCGCCAA
Hongtoumai	ATAACGAGAAGGGCTGCATGAAGATGCAACTTTGCCTTGGAGAAGAGGCCCTTACTCCATCTCGCCAA
Hongyouzi	ATAACGAGAAGGGCTGCATGAAGATGCAACTTTGCCTTGGAGAAGAGGCCCTTACTCCATCTCGCCAA
Zijiehong	ATAACGAGAAGGGCTGCATGAAGATGCAACTTTGCCTTGGAGAAGAGGCCCTTACTCCATCTCGCCAA
Baihuamai	ATAACGAGAAGGGCTGCATGAAGATGCAACTTTGCCTTGGAGAAGAGGCCCTTACTCCATCTCGCCAA
Huixianhong	ATAACGAGAAGGGCTGCATGAAGATGCAACTTTGCCTTGGAGAAGAGGCCCTTACTCCATCTCGCCAA
Baimangmai	ATAACGAGAAGGGCTGCATGAAGATGCAACTTTGCCTTGGAGAAGAGGCCCTTACTCCATCTCGCCAA

	3,410 · 3,420 · 3,430 · 3,440 · 3,450 · 3,460
Hulutou	GTGCCACCCCGTTTTCCTGAGCTTATGGAAAAGCCGCTGGCAAGGCTCCGTGTGCTCGCATGGCTCAT
Baihulu	GTGCCACCCCGTTTTCCTGAGCTTATGGAAAAGCCGCTGGCAAGGCTCCGTGTGCTCGCATGGCTCAT
Chiyacao	GTGCCACCCCGTTTTCCTGAGCTTATGGAAAAGCCGCTGGCAAGGCTCCGTGTGCTCGCATGGCTCAT
Hongmangmai	GTGCCACCCCGTTTTCCTGAGCTTATGGAAAAGCCGCTGGCAAGGCTCCGTGTGCTCGCATGGCTCAT
Hongmai	GTGCCACCCCGTTTTCCTGAGCTTATGGAAAAGCCGCTGGCAAGGCTCCGTGTGCTCGCATGGCTCAT
Wangshuibai	GTGCCACCCCGTTTTCCTGAGCTTATGGAAAAGCCGCTGGCAAGGCTCCGTGTGCTCGCATGGCTCAT
Shi 4185	GTGCCACCCCGTTTTCCTGAGCTTATGGAAAAGCCGCTGGCAAGGCTCCGTGTGCTCGCATGGCTCAT
Mazhamai	GTGCCACCCCGTTTTCCTGAGCTTATGGAAAAGCCGCTGGCAAGGCTCCGTGTGCTCGCATGGCTCAT
Xiaofoshou	GTGCCACCCCGTTTTCCTGAGCTTATGGAAAAGCCGCTGGCAAGGCTCCGTGTGCTCGCATGGCTCAT
Aikang 58	GTGCCACCCCGTTTTCCTGAGCTTATGGAAAAGCCGCTGGCAAGGCTCCGTGTGCTCGCATGGCTCAT
AL8/78	GTGCCACCCCGTTTTCCTGAGCTTATGGAAAAGCCGCTGGCAAGGCTCCGTGTGCTCGCATGGCTCAT
PI 431602	GTGCCACCCCGTTTTCCTGAGCTTATGGAAAAGCCGCTGGCAAGGCTCCGTGTGCTCGCATGGCTCAT
PI 486274	GTGCCACCCCGTTTTCCTGAGCTTATGGAAAAGCCGCTGGCAAGGCTCCGTGTGCTCGCATGGCTCAT
RM 000182	GTGCCACCCCGTTTTCTTGAGCTTATGGAAAAGCCGCTGGCAAGGCTCCGTGTGCTCGCATGGCTCAT
PI 511381	GTGCCACCCCGTTTTCTTGAGCTTATGGAAAAGCCGCTGGCAAGGCTCCGTGTGCTCGCATGGCTCAT
PI 486271	GTGCCACCCCGTTTTCTTGAGCTTATGGAAAAGCCGCTGGCAAGGCTCCGTGTGCTCGCATGGCTCAT
PI 511367	GTGCCACCCCGTTTTCTTGAGCTTATGGAAAAGCCGCTGGCAAGGCTCCGTGTGCTCGCATGGCTCAT
Chinese Spring	GTGCCACCCCGTTTTCCTGAGCTTATGGAAAAGCCGCTGGCAAGGCTCCGTGTGCTCGCATGGCTCAT
Hanzhongbai	GTGCCACCCCGTTTTCCTGAGCTTATGGAAAAGCCGCTGGCAAGGCTCCGTGTGCTCGCATGGCTCAT
Hongtoumai	GTGCCACCCCGTTTTCCTGAGCTTATGGAAAAGCCGCTGGCAAGGCTCCGTGTGCTCGCATGGCTCAT
Hongyouzi	GTGCCACCCCGTTTTCCTGAGCTTATGGAAAAGCCGCTGGCAAGGCTCCGTGTGCTCGCATGGCTCAT
Zijiehong	GTGCCACCCCGTTTTCCTGAGCTTATGGAAAAGCCGCTGGCAAGGCTCCGTGTGCTCGCATGGCTCAT
Baihuamai	GTGCCACCCCGTTTTCCTGAGCTTATGGAAAAGCCGCTGGCAAGGCTCCGTGTGCTCGCATGGCTCAT
Huixianhong	GTGCCACCCCGTTTTCCTGAGCTTATGGAAAAGCCGCTGGCAAGGCTCCGTGTGCTCGCATGGCTCAT
Baimangmai	GTGCCACCCCGTTTTCCTGAGCTTATGGAAAAGCCGCTGGCAAGGCTCCGTGTGCTCGCATGGCTCAT

	3,470 · 3,480 · 3,490 · 3,500 · 3,510 · 3,520 · 3,530
Hulutou	CCCAAGCCTTTGACACGATGTCAAAGAAGCCGGGGAGACAGGACAGAAAATTCTCAAATTTGAAACAT
Baihulu	CCCAAGCCTTTGACACGATGTCAAAGAAGCCGGGGAGACAGGACAGAAAATTCTCAAATTTGAAACAT
Chiyacao	CCCAAGCCTTTGACACGATGTCAAAGAAGCCGGGGAGACAGGACAGAAAATTCTCAAATTTGAAACAT
Hongmangmai	CCCAAGCCTTTGACACGATGTCAAAGAAGCCGGGGAGACAGGACAGAAAATTCTCAAATTTGAAACAT
Hongmai	CCCAAGCCTTTGACACGATGTCAAAGAAGCCGGGGAGACAGGACAGAAAATTCTCAAATTTGAAACAT
Wangshuibai	CCCAAGCCTTTGACACGATGTCAAAGAAGCCGGGGAGACAGGACAGAAAATTCTCAAATTTGAAACAT
Shi 4185	CCCAAGCCTTTGACACGATGTCAAAGAAGCCGGGGAGACAGGACAGAAAATTCTCAAATTTGAAACAT
Mazhamai	CCCAAGCCTTTGACACGATGTCAAAGAAGCCGGGGAGACAGGACAGAAAATTCTCAAATTTGAAACAT
Xiaofoshou	CCCAAGCCTTTGACACGATGTCAAAGAAGCCGGGGAGACAGGACAGAAAATTCTCAAATTTGAAACAT
Aikang 58	CCCAAGCCTTTGACACGATGTCAAAGAAGCCGGGGAGACAGGACAGAAAATTCTCAAATTTGAAACAT
AL8/78	CCCAAGCCTTTGACACGATGTCAAAGAAGCCGGGGAGACAGGACAGAAAATTCTCAAATTTGAAACAT
PI 431602	CCCAAGCCTTTGACACGATGTCAAAGAAGCCGGGGAGACAGGACAGAAAATTCTCAAATTTGAAACAT
PI 486274	CCCAAGCCTTTGACACGATGTCAAAGAAGCCGGGGAGACAGGACAGAAAATTCTCAAATTTGAAACAT
RM 000182	CCCAAGCCTTTGACATGATGTCAAAGAAGCCGGGGAGACAGGACAGAAAATTCTCAAATTTGAAACAT
PI 511381	CCCAAGCCTTTGACATGATGTCAAAGAAGCCGGGGAGACAGGACAGAAAATTCTCAAATTTGAAACAT
PI 486271	CCCAAGCCTTTGACATGATGTCAAAGAAGCCGGGGAGACAGGACAGAAAATTCTCAAATTTGAAACAT
PI 511367	CCCAAGCCTTTGACATGATGTCAAAGAAGCCGGGGAGACAGGACAGAAAATTCTCAAATTTGAAACAT
Chinese Spring	CCCAAGCCTTTGACACGATGTCAAAGAAGCCGGGGAGACAGGACAGAAAATTCTCAAATTTGAAACAT
Hanzhongbai	CCCAAGCCTTTGACACGATGTCAAAGAAGCCGGGGAGACAGGACAGAAAATTCTCAAATTTGAAACAT
Hongtoumai	CCCAAGCCTTTGACACGATGTCAAAGAAGCCGGGGAGACAGGACAGAAAATTCTCAAATTTGAAACAT
Hongyouzi	CCCAAGCCTTTGACACGATGTCAAAGAAGCCGGGGAGACAGGACAGAAAATTCTCAAATTTGAAACAT
Zijiehong	CCCAAGCCTTTGACACGATGTCAAAGAAGCCGGGGAGACAGGACAGAAAATTCTCAAATTTGAAACAT
Baihuamai	CCCAAGCCTTTGACACGATGTCAAAGAAGCCGGGGAGACAGGACAGAAAATTCTCAAATTTGAAACAT
Huixianhong	CCCAAGCCTTTGACACGATGTCAAAGAAGCCGGGGAGACAGGACAGAAAATTCTCAAATTTGAAACAT
Baimangmai	CCCAAGCCTTTGACACGATGTCAAAGAAGCCGGGGAGACAGGACAGAAAATTCTCAAATTTGAAACAT

	3,540 3,550 3,560 3,570 3,580 3,590 3,600
Hulutou	CTTGGTCGCTTGGGCCCCTTGTCATCAGCGAGGAGGAGAGGGCAGTGATCCGAAAGTGAATAGGAGAT
Baihulu	CTTGGTCGCTTGGGCCCCTTGTCATCAGCGAGGAGGAGAGGGCAGTGATCCGAAAGTGAATAGGAGAT
Chiyacao	CTTGGTCGCTTGGGCCCCTTGTCATCAGCGAGGAGGAGAGGGCAGTGATCCGAAAGTGAATAGGAGAT
Hongmangmai	CTTGGTCGCTTGGGCCCCTTGTCATCAGCGAGGAGGAGAGGGCAGTGATCCGAAAGTGAATAGGAGAT
Hongmai	CTTGGTCGCTTGGGCCCCTTGTCATCAGCGAGGAGGAGAGGGCAGTGATCCGAAAGTGAATAGGAGAT
Wangshuibai	CTTGGTCGCTTGGGCCCCTTGTCATCAGCGAGGAGGAGAGGGCAGTGATCCGAAAGTGAATAGGAGAT
Shi 4185	CTTGGTCGCTTGGGCCCCTTGTCATCAGCGAGGAGGAGAGGGCAGTGATCCGAAAGTGAATAGGAGAT
Mazhamai	CTTGGTCGCTTGGGCCCCTTGTCATCAGCGAGGAGGAGAGGGCAGTGATCCGAAAGTGAATAGGAGAT
Xiaofoshou	CTTGGTCGCTTGGGCCCCTTGTCATCAGCGAGGAGGAGAGGGCAGTGATCCGAAAGTGAATAGGAGAT
Aikang 58	CTTGGTCGCTTGGGCCCCTTGTCATCAGCGAGGAGGAGAGGGCAGTGATCCGAAAGTGAATAGGAGAT
AL8/78	CTTGGTCGCTTGGGCCCCTTGTCATCAGCGAGGAGGAGAGGGCAGTGATCCGAAAGTG-ATTGGAGAT
PI 431602	CTTGGTCGCTTGGGCCCCTTGTCATCAGCGAGGAGGAGAGGGCAGTGATCCGAAAGTG-ATTGGAGAT
PI 486274	CTTGGTCGCTTGGGCCCCTTGTCATCAGCGAGGAGGAGAGGGCAGTGATCCGAAAGTG-ATTGGAGAT
RM 000182	CTTGGTCGCTTGGGCCCCTTGTCATCAGCGAGGAGGAGAGGGCAGTGATCCGAAAGTTTATAGGAGAT
PI 511381	CTTGGTCGCTTGGGCCCCTTGTCATCAGCGAGGAGGAGAGGGCAGTGATCCGAAAGTTTATAGGAGAT
PI 486271	CTTGGTCGCTTGGGCCCCTTGTCATCAGCGAGGAGGAGAGGGCAGTGATCCGAAAGTTTATAGGAGAT
PI 511367	CTTGGTCGCTTGGGCCCCTTGTCATCAGCGAGGAGGAGAGGGCAGTGATCCGAAAGTTTATAGGAGAT
Chinese Spring	CTTGGTCGCTTGGGCCCCTTGTCATCAACGAGGAGGAGAGGGCAGTGATCCGAAAGTGAATAGGAGAT
Hanzhongbai	CTTGGTCGCTTGGGCCCCTTGTCATCAACGAGGAGGAGAGGGCAGTGATCCGAAAGTGAATAGGAGAT
Hongtoumai	CTTGGTCGCTTGGGCCCCTTGTCATCAACGAGGAGGAGAGGGCAGTGATCCGAAAGTGAATAGGAGAT
Hongyouzi	CTTGGTCGCTTGGGCCCCTTGTCATCAACGAGGAGGAGAGGGCAGTGATCCGAAAGTGAATAGGAGAT
Zijiehong	CTTGGTCGCTTGGGCCCCTTGTCATCAACGAGGAGGAGAGGGCAGTGATCCGAAAGTGAATAGGAGAT
Baihuamai	CTTGGTCGCTTGGGCCCCTTGTCATCAACGAGGAGGAGAGGGCAGTGATCCGAAAGTGAATAGGAGAT
Huixianhong	CTTGGTCGCTTGGGCCCCTTGTCATCAACGAGGAGGAGAGGGCAGTGATCCGAAAGTGAATAGGAGAT
Baimangmai	CTTGGTCGCTTGGGCCCCTTGTCATCAACGAGGAGGAGAGGGCAGTGATCCGAAAGTGAATAGGAGAT

	3,610 3,620 3,630 3,640 3,650 3,660 3,670
Hulutou	GGAGTGGAGCACATGGGAGTTAAAATGCATGTCCCAATCGGCATTTCGGAACATCAAGTCAAGCTTGC
Baihulu	GGAGTGGAGCACATGGGAGTTAAAATGCATGTCCCAATCGGCATTTCGGAACATCAAGTCAAGCTTGC
Chiyacao	GGAGTGGAGCACATGGGAGTTAAAATGCATGTCCCAATCGGCATTTCGGAACATCAAGTCAAGCTTGC
Hongmangmai	GGAGTGGAGCACATGGGAGTTAAAATGCATGTCCCAATCGGCATTTCGGAACATCAAGTCAAGCTTGC
Hongmai	GGAGTGGAGCACATGGGAGTTAAAATGCATGTCCCAATCGGCATTTCGGAACATCAAGTCAAGCTTGC
Wangshuibai	GGAGTGGAGCACATGGGAGTTAAAATGCATGTCCCAATCGGCATTTCGGAACATCAAGTCAAGCTTGC
Shi 4185	GGAGTGGAGCACATGGGAGTTAAAATGCATGTCCCAATCGGCATTTCGGAACATCAAGTCAAGCTTGC
Mazhamai	GGAGTGGAGCACATGGGAGTTAAAATGCATGTCCCAATCGGCATTTCGGAACATCAAGTCAAGCTTGC
Xiaofoshou	GGAGTGGAGCACATGGGAGTTAAAATGCATGTCCCAATCGGCATTTCGGAACATCAAGTCAAGCTTGC
Aikang 58	GGAGTGGAGCACATGGGAGTTAAAATGCATGTCCCAATCGGCATTTCGGAACATCAAGTCAAGCTTGC
AL8/78	GGAGTGGAGCACATGGGAGTTAAAATGCATGTCCCAATCGGCATTTCGGAACATCGAGTCAAGCTTGC
PI 431602	GGAGTGGAGCACATGGGAGTTAAAATGCATGTCCCAATCGGCATTTCGGAACATCGAGTCAAGCTTGC
PI 486274	GGAGTGGAGCACATGGGAGTTAAAATGCATGTCCCAATCGGCATTTCGGAACATCGAGTCAAGCTTGC
RM 000182	GGAGTGGAGCACATGGGAGTTAAAATGCATGTCCCAATCGGCATTTCGGAACATCGAGTCAAGCTTGC
PI 511381	GGAGTGGAGCACATGGGAGTTAAAATGCATGTCCCAATCGGCATTTCGGAACATCGAGTCAAGCTTGC
PI 486271	GGAGTGGAGCACATGGGAGTTAAAATGCATGTCCCAATCGGCATTTCGGAACATCGAGTCAAGCTTGC
PI 511367	GGAGTGGAGCACATGGGAGTTAAAATGCATGTCCCAATCGGCATTTCGGAACATCGAGTCAAGCTTGC
Chinese Spring	GGAGTGGAGCACATGGGAGTTAAAATGCATGTCCCAATCGGCATTTCGGAACATCGAGTCAAGCTTGC
Hanzhongbai	GGAGTGGAGCACATGGGAGTTAAAATGCATGTCCCAATCGGCATTTCGGAACATCGAGTCAAGCTTGC
Hongtoumai	GGAGTGGAGCACATGGGAGTTAAAATGCATGTCCCAATCGGCATTTCGGAACATCGAGTCAAGCTTGC
Hongyouzi	GGAGTGGAGCACATGGGAGTTAAAATGCATGTCCCAATCGGCATTTCGGAACATCGAGTCAAGCTTGC
Zijiehong	GGAGTGGAGCACATGGGAGTTAAAATGCATGTCCCAATCGGCATTTCGGAACATCGAGTCAAGCTTGC
Baihuamai	GGAGTGGAGCACATGGGAGTTAAAATGCATGTCCCAATCGGCATTTCGGAACATCGAGTCAAGCTTGC
Huixianhong	GGAGTGGAGCACATGGGAGTTAAAATGCATGTCCCAATCGGCATTTCGGAACATCGAGTCAAGCTTGC
Baimangmai	GGAGTGGAGCACATGGGAGTTAAAATGCATGTCCCAATCGGCATTTCGGAACATCGAGTCAAGCTTGC

	3,680 3,690 3,700 3,710 3,720 3,730 3,740
Hulutou	AAAGAGTTGGGTTTTGACGCTCGTTGCTCCAAGTGAATCGTATGTTTTGCATTACCATGCCTTCTATT
Baihulu	AAAGAGTTGGGTTTTGACGCTCGTTGCTCCAAGTGAATCGTATGTTTTGCATTACCATGCCTTCTATT
Chiyacao	AAAGAGTTGGGTTTTGACGCTCGTTGCTCCAAGTGAATCGTATGTTTTGCATTACCATGCCTTCTATT
Hongmangmai	AAAGAGTTGGGTTTTGACGCTCGTTGCTCCAAGTGAATCGTATGTTTTGCATTACCATGCCTTCTATT
Hongmai	AAAGAGTTGGGTTTTGACGCTCGTTGCTCCAAGTGAATCGTATGTTTTGCATTACCATGCCTTCTATT
Wangshuibai	AAAGAGTTGGGTTTTGACGCTCGTTGCTCCAAGTGAATCGTATGTTTTGCATTACCATGCCTTCTATT
Shi 4185	AAAGAGTTGGGTTTTGACGCTCGTTGCTCCAAGTGAATCGTATGTTTTGCATTACCATGCCTTCTATT
Mazhamai	AAAGAGTTGGGTTTTGACGCTCGTTGCTCCAAGTGAATCGTATGTTTTGCATTACCATGCCTTCTATT
Xiaofoshou	AAAGAGTTGGGTTTTGACGCTCGTTGCTCCAAGTGAATCGTATGTTTTGCATTACCATGCCTTCTATT
Aikang 58	AAAGAGTTGGGTTTTGACGCTCGTTGCTCCAAGTGAATCGTATGTTTTGCATTACCATGCCTTCTATT
AL8/78	AAAGAGTTGGGTTTTGACGCTCGTTGCTCCAAGTGAATCGTATGTTTTGCATTACCATGCCTTCTATT
PI 431602	AAAGAGTTGGGTTTTGACGCTCGTTGCTCCAAGTGAATCGTATGTTTTGCATTACCATGCCTTCTATT
PI 486274	AAAGAGTTGGGTTTTGACGCTCGTTGCTCCAAGTGAATCGTATGTTTTGCATTACCATGCCTTCTATT
RM 000182	AAAGAGTTGGGTTTTGACGCTCGTTGCTCCAAGTGAATCGTATGTTTTGCATTACCATGCCTTCTATT
PI 511381	AAAGAGTTGGGTTTTGACGCTCGTTGCTCCAAGTGAATCGTATGTTTTGCATTACCATGCCTTCTATT
PI 486271	AAAGAGTTGGGTTTTGACGCTCGTTGCTCCAAGTGAATCGTATGTTTTGCATTACCATGCCTTCTATT
PI 511367	AAAGAGTTGGGTTTTGACGCTCGTTGCTCCAAGTGAATCGTATGTTTTGCATTACCATGCCTTCTATT
Chinese Spring	AAAGAGTTGGGTTTTGACGCTCGTTGCTCCAAGTGAATCGTATGTTTTGCATTACCATGCCTTCTATT
Hanzhongbai	AAAGAGTTGGGTTTTGACGCTCGTTGCTCCAAGTGAATCGTATGTTTTGCATTACCATGCCTTCTATT
Hongtoumai	AAAGAGTTGGGTTTTGACGCTCGTTGCTCCAAGTGAATCGTATGTTTTGCATTACCATGCCTTCTATT
Hongyouzi	AAAGAGTTGGGTTTTGACGCTCGTTGCTCCAAGTGAATCGTATGTTTTGCATTACCATGCCTTCTATT
Zijiehong	AAAGAGTTGGGTTTTGACGCTCGTTGCTCCAAGTGAATCGTATGTTTTGCATTACCATGCCTTCTATT
Baihuamai	AAAGAGTTGGGTTTTGACGCTCGTTGCTCCAAGTGAATCGTATGTTTTGCATTACCATGCCTTCTATT
Huixianhong	AAAGAGTTGGGTTTTGACGCTCGTTGCTCCAAGTGAATCGTATGTTTTGCATTACCATGCCTTCTATT
Baimangmai	AAAGAGTTGGGTTTTGACGCTCGTTGCTCCAAGTGAATCGTATGTTTTGCATTACCATGCCTTCTATT

	3,750 3,760 3,770 3,780 3,790 3,800
Hulutou	TTTATCTCAAGCACGATAGATTTGGTTGAAGTCGCCCATGACAATCCA-TTTAGTGCCATTGTTGGGT
Baihulu	TTTATCTCAAGCACGATAGATTTGGTTGAAGTCGCCCATGACAATCCA-TTTAGTGCCATTGTTGGGT
Chiyacao	TTTATCTCAAGCACGATAGATTTGGTTGAAGTCGCCCATGACAATCCA-TTTAGTGCCATTGTTGGGT
Hongmangmai	TTTATCTCAAGCACGATAGATTTGGTTGAAGTCGCCCATGACAATCCA-TTTAGTGCCATTGTTGGGT
Hongmai	TTTATCTCAAGCACGATAGATTTGGTTGAAGTCGCCCATGACAATCCA-TTTAGTGCCATTGTTGGGT
Wangshuibai	TTTATCTCAAGCACGATAGATTTGGTTGAAGTCGCCCATGACAATCCA-TTTAGTGCCATTGTTGGGT
Shi 4185	TTTATCTCAAGCACGATAGATTTGGTTGAAGTCGCCCATGACAATCCA-TTTAGTGCCATTGTTGGGT
Mazhamai	TTTATCTCAAGCACGATAGATTTGGTTGAAGTCGCCCATGACAATCCA-TTTAGTGCCATTGTTGGGT
Xiaofoshou	TTTATCTCAAGCACGATAGATTTGGTTGAAGTCGCCCATGACAATCCA-TTTAGTGCCATTGTTGGGT
Aikang 58	TTTATCTCAAGCACGATAGATTTGGTTGAAGTCGCCCATGACAATCCA-TTTAGTGCCATTGTTGGGT
AL8/78	TTTATCTCAAGCACGATAGATTTGGTTGAAGTCGCCCATGACAATCCA-TTTAGTGCCATTGTTGGGT
PI 431602	TTTATCTCAAGCACGATAGATTTGGTTGAAGTCGCCCATGACAATCCA-TTTAGTGCCATTGTTGGGT
PI 486274	TTTATCTCAAGCACGATAGATTTGGTTGAAGTCGCCCATGACAATCCA-TTTAGTGCCATTGTTGGGT
RM 000182	TTTATCTCAAGCACGATAGATTTGGTTGAAGTCGCCCATGACAATCCA-TTTAGTGCCATTGTTGGGT
PI 511381	TTTATCTCAAGCACGATAGATTTGGTTGAAGTCGCCCATGACAATCCA-TTTAGTGCCATTGTTGGGT
PI 486271	TTTATCTCAAGCACGATAGATTTGGTTGAAGTCGCCCATGACAATCCA-TTTAGTGCCATTGTTGGGT
PI 511367	TTTATCTCAAGCACGATAGATTTGGTTGAAGTCGCCCATGACAATCCATTTTAGTGCCATTGTTGGGT
Chinese Spring	TTTATCTCAAGCACGATAGATTTGGTTGAAGTCGCCCATGACAATCCA-TTTAGTGCCATTGTTGGGT
Hanzhongbai	TTTATCTCAAGCACGATAGATTTGGTTGAAGTCGCCCATGACAATCCA-TTTAGTGCCATTGTTGGGT
Hongtoumai	TTTATCTCAAGCACGATAGATTTGGTTGAAGTCGCCCATGACAATCCA-TTTAGTGCCATTGTTGGGT
Hongyouzi	TTTATCTCAAGCACGATAGATTTGGTTGAAGTCGCCCATGACAATCCA-TTTAGTGCCATTGTTGGGT
Zijiehong	TTTATCTCAAGCACGATAGATTTGGTTGAAGTCGCCCATGACAATCCA-TTTAGTGCCATTGTTGGGT
Baihuamai	TTTATCTCAAGCACGATAGATTTGGTTGAAGTCGCCCATGACAATCCA-TTTAGTGCCATTGTTGGGT
Huixianhong	TTTATCTCAAGCACGATAGATTTGGTTGAAGTCGCCCATGACAATCCA-TCTAGTGCCATTGTTGGGT
Baimangmai	TTTATCTCAAGCACGATAGATTTGGTTGAAGTCGCCCATGACAATCCA-TTTAGTGCCATTGTTGGGT

```
                 3,810     3,820     3,830     3,840     3,850     3,860     3,870
Hulutou          TTTTGTGAGATTAGCTCGTT--AAAGAAGTCACCCTTTCTGTCGGAGGCCGT-GGGAACATAGACACT
Baihulu          TTTTGTGAGATTAGCTCGTT--AAAGAAGTCACCCTTTCTGTCGGAGGCCGT-GGGAACATAGACACT
Chiyacao         TTTTGTGAGATTAGCTCGTT--AAAGAAGTCACCCTTTCTGTCGGAGGCCGT-GGGAACATAGACACT
Hongmangmai      TTTTGTGAGATTAGCTCGTT--AAAGAAGTCACCCTTTCTGTCGGAGGCCGT-GGGAACATAGACACT
Hongmai          TTTTGTGAGATTAGCTCGTT--AAAGAAGTCACCCTTTCTGTCGGAGGCCGT-GGGAACATAGACACT
Wangshuibai      TTTTGTGAGATTAGCTCGTT--AAAGAAGTCACCCTTTCTGTCGGAGGCCGT-GGGAACATAGACACT
Shi 4185         TTTTGTGAGATTAGCTCGTT--AAAGAAGTCACCCTTTCTGTCGGAGGCCGT-GGGAACATAGACACT
Mazhamai         TTTTGTGAGATTAGCTCGTT--AAAGAAGTCACCCTTTCTGTCGGAGGCCGT-GGGAACATAGACACT
Xiaofoshou       TTTTGTGAGATTAGCTCGTT--AAAGAAGTCACCCTTTCTGTCGGAGGCCGT-GGGAACATAGACACT
Aikang 58        TTTTGTGAGATTAGCTCGTT--AAAGAAGTCACCCTTTCTGTCGGAGGCCGT-GGGAACATAGACACT
AL8/78           TTTTGTGAGATTAGCTCGTT--AAAGAAGTCACCCTTTCTGTCGGAGGCCGT-GGGAACATAGACACT
PI 431602        TTTTGTGAGATTAGCTCGTT--AAAGAAGTCACCCTTTCTGTCGGAGGCCGT-GGGAACATAGACACT
PI 486274        TTTTGTGAGATTAGCTCGTT--AAAGAAGTCACCCTTTCTGTCGGAGGCCGT-GGGAACATAGACACT
RM 000182        TTTTGTGAGATTAGCTCGTT--AAAGAAGTCACCCTTTCTGTCGGAGGCCGT-GGGAACATAGACACT
PI 511381        TTTTGTGAGATTAGCTCGTT--AAAGAAGTCACCCTTTCTGTCGGAGGCCGT-GGGAACATAGACACT
PI 486271        TTTTGTGAGATTAGCTCGTT--AAAGAAGTCACCCTTTCTGTCGGAGGCCGT-GGGAACATAGACACT
PI 511367        TTTTGTGAGATTAGCTCGTTAGAAGGAAGTCACCCTTTCTGTCGGAGGCCGTGGGGAACATAGACACT
Chinese Spring   TTTTGTGAGATTAGCTCGTT--AAAGAAGTCACCCTTTCTGTCGGAGGCCGT-GGGAACATAGACACT
Hanzhongbai      TTTTGTGAGATTAGCTCGTT--AAAGAAGTCACCCTTTCTGTCGGAGGCCGT-GGGAACATAGACACT
Hongtoumai       TTTTGTGAGATTAGCTCGTT--AAAGAAGTCACCCTTTCTGTCGGAGGCCGT-GGGAACATAGACACT
Hongyouzi        TTTTGTGAGATTAGCTCGTT--AAAGAAGTCACCCTTTCTGTCGGAGGCCGT-GGGAACATAGACACT
Zijiehong        TTTTGTGAGATTAGCTCGTT--AAAGAAGTCACCCTTTCTGTCGGAGGCCGT-GGGAACATAGACACT
Baihuamai        TTTTGTGAGATTAGCTCGTT--AAAGAAGTCACCCTTTCTGTCGGAGGCCGT-GGGAACATAGACACT
Huixianhong      TTTTGTGAGATTAGCTCGTT--AAAGAAGTCACCCTTTCTGTCGGAGGCCGT-GGGAACATAGACACT
Baimangmai       TTTTGTGAGATTAGCTCGTT--AAAGAAGTCACCCTTTCTGTCGGAGGCCGT-GGGAACATAGACACT
```

```
                   3,880     3,890     3,900     3,910     3,920     3,930     3,940
Hulutou          -AGTGATCATGAAGTAGGTATCGGTGTGAATCACATTGACCATTGCCGAGAGGCAGAACTCAGTAAAC
Baihulu          -AGTGATCATGAAGTAGGTATCGGTGTGAATCACATTGACCATTGCCGAGAGGCAGAACTCAGTAAAC
Chiyacao         -AGTGATCATGAAGTAGGTATCGGTGTGAATCACATTGACCATTGCCGAGAGGCAGAACTCAGTAAAC
Hongmangmai      -AGTGATCATGAAGTAGGTATCGGTGTGAATCACATTGACCATTGCCGAGAGGCAGAACTCAGTAAAC
Hongmai          -AGTGATCATGAAGTAGGTATCGGTGTGAATCACATTGACCATTGCCGAGAGGCAGAACTCAGTAAAC
Wangshuibai      -AGTGATCATGAAGTAGGTATCGGTGTGAATCACATTGACCATTGCCGAGAGGCAGAACTCAGTAAAC
Shi 4185         -AGTGATCATGAAGTAGGTATCGGTGTGAATCACATTGACCATTGCCGAGAGGCAGAACTCAGTAAAC
Mazhamai         -AGTGATCATGAAGTAGGTATCGGTGTGAATCACATTGACCATTGCCGAGAGGCAGAACTCAGTAAAC
Xiaofoshou       -AGTGATCATGAAGTAGGTATCGGTGTGAATCACATTGACCATTGCCGAGAGGCAGAACTCAGTAAAC
Aikang 58        -AGTGATCATGAAGTAGGTATCGGTGTGAATCACATTGACCATTGCCGAGAGGCAGAACTCAGTAAAC
AL8/78           -AGTGATCATGAAGTAGGTATCGGTGTGAATCACATTGACCATTGCCGAGAGGCAGAACTCAGTAAAG
PI 431602        -AGTGATCATGAAGTAGGTATCGGTGTGAATCACATTGACCATTGCCGAGAGGCAGAACTCAGTAAAG
PI 486274        -AGTGATCATGAAGTAGGTATCGGTGTGAATCACATTGACCATTGCCGAGAGGCAGAACTCAGTAAAG
RM 000182        -AGTGATCATGAAGTAGGTATCGGTGTGAATCACATTGACCATTGCCGAGAGGCAGAACTCAGTAAAG
PI 511381        -AGTGATCATGAAGTAGGTATCGGTGTGAATCACATTGACCATTGCCGAGAGGCAGAACTCAGTAAAG
PI 486271        -AGTGATCATGAAGTAGGTATCGGTGTGAATCACATTGACCATTGCCGAGAGGCAGAACTCAGTAAAG
PI 511367        AAGTGATCATGAAGTAGGTATCGGTGTGAATCACATTGACCATTGCCGAGAGGCAGAACTCAGTAAAG
Chinese Spring   -AGTGATCATGAAGTAGGTATCGGTGTGAATCACATTGACCATTGCCGAGAGGCAGAACTCAGTAAAG
Hanzhongbai      -AGTGATCATGAAGTAGGTATCGGTGTGAATCACATTGACCATTGCCGAGAGGCAGAACTCAGTAAAG
Hongtoumai       -AGTGATCATGAAGTAGGTATCGGTGTGAATCACATTGACCATTGCCGAGAGGCAGAACTCAGTAAAG
Hongyouzi        -AGTGATCATGAAGTAGGTATCGGTGTGAATCACATTGACCATTGCCGAGAGGCAGAACTCAGTAAAG
Zijiehong        -AGTGATCATGAAGTAGGTATCGGTGTGAATCACATTGACCATTGCCGAGAGGCAGAACTCAGTAAAG
Baihuamai        -AGTGATCATGAAGTAGGTATCGGTGTGAATCACATTGACCATTGCCGAGAGGCAGAACTCAGTAAAG
Huixianhong      -AGTGATCATGAAGTAGGTATCGGTGTGAATCACATTGACCATTGCCGAGAGGCAGAACTCAGTAAAG
Baimangmai       -AGTGATCATGAAGTAGGTATCGGTGTGAATCACATTGACCATTGCCGAGAGGCAGAACTCAGTAAAG
```

3,950 3,960 3,970 3,980 3,990 4,000 4,010

Hulutou	TTGACGTCTGAAATACGGACGACACTATCGTCCCAAAGCATGAGTAGGCCCCCTCTGGTTCCAATAGC
Baihulu	TTGACGTCTGAAATACGGACGACACTATCGTCCCAAAGCATGAGTAGGCCCCCTCTGGTTCCAATAGC
Chiyacao	TTGACGTCTGAAATACGGACGACACTATCGTCCCAAAGCATGAGTAGGCCCCCTCTGGTTCCAATAGC
Hongmangmai	TTGACGTCTGAAATACGGACGACACTATCGTCCCAAAGCATGAGTAGGCCCCCTCTGGTTCCAATAGC
Hongmai	TTGACGTCTGAAATACGGACGACACTATCGTCCCAAAGCATGAGTAGGCCCCCTCTGGTTCCAATAGC
Wangshuibai	TTGACGTCTGAAATACGGACGACACTATCGTCCCAAAGCATGAGTAGGCCCCCTCTGGTTCCAATAGC
Shi 4185	TTGACGTCTGAAATACGGACGACACTATCGTCCCAAAGCATGAGTAGGCCCCCTCTGGTTCCAATAGC
Mazhamai	TTGACGTCTGAAATACGGACGACACTATCGTCCCAAAGCATGAGTAGGCCCCCTCTGGTTCCAATAGC
Xiaofoshou	TTGACGTCTGAAATACGGACGACACTATCGTCCCAAAGCATGAGTAGGCCCCCTCTGGTTCCAATAGC
Aikang 58	TTGACGTCTGAAATACGGACGACACTATCGTCCCAAAGCATGAGTAGGCCCCCTCTGGTTCCAATAGC
AL8/78	TTGACGTCTGAAATACGGATGACACTATCGTCCCAAAGCATGAGTAGGCCCCCTTTGGTTCCAATAGC
PI 431602	TTGACGTCTGAAATACGGATGACACTATCGTCCCAAAGCATGAGTAGGCCCCCTTTGGTTCCAATAGC
PI 486274	TTGACGTCTGAAATACGGATGACACTATCGTCCCAAAGCATGAGTAGGCCCCCTTTGGTTCCAATAGC
RM 000182	TTGACGTCTGAAATACGGACGGCACTATCGTCCCAAAGCATGAGTAGGACCCCTCTGGTTCCAATAGC
PI 511381	TTGACGTCTGAAATACGGACGGCACTATCGTCCCAAAGCATGAGTAGGACCCCTCTGGTTCCAATAGC
PI 486271	TTGACGTCTGAAATACGGACGGCACTATCGTCCCAAAGCATGAGTAGGACCCCTCTGGTTCCAATAGC
PI 511367	TTGACGTCTGAAATACGGACGGCACTATCGTCCCAAAGCATGAGTAGGACCCCTCTGGTTCCAATAGC
Chinese Spring	TTGACGTCTGAAATACGGATGACACTATCGTCCCAAAGCATGAGTAGGCCCCCTTTGGTTCCAATAGC
Hanzhongbai	TTGACGTCTGAAATACGGATGACACTATCGTCCCAAAGCATGAGTAGGCCCCCTTTGGTTCCAATAGC
Hongtoumai	TTGACGTCTGAAATACGGATGACACTATCGTCCCAAAGCATGAGTAGGCCCCCTTTGGTTCCAATAGC
Hongyouzi	TTGACGTCTGAAATACGGATGACACTATCGTCCCAAAGCATGAGTAGGCCCCCTTTGGTTCCAATAGC
Zijiehong	TTGACGTCTGAAATACGGATGACACTATCGTCCCAAAGCATGAGTAGGCCCCCTTTGGTTCCAATAGC
Baihuamai	TTGACGTCTGAAATACGGATGACACTATCGTCCCAAAGCATGAGTAGGCCCCCTTTGGTTCCAATAGC
Huixianhong	TTGACGTCTGAAATACGGATGACACTATCGTCCCAAAGCATGAGTAGGCCCCCTTTGGTTCCAATAGC
Baimangmai	TTGACGTCTGAAATACGGATGACACTATCGTCCCAAAGCATGAGTAGGCCCCCTTTGGTTCCAATAGC

4,020 4,030 4,040 4,050 4,060 4,070 4,080

Hulutou	CGGACGCTGACAAAACTCGAGAGCCTATTGCCGCCGAGGTAGGAGGCGATGAATCGGTCCACTGATTC
Baihulu	CGGACGCTGACAAAACTCGAGAGCCTATTGCCGCCGAGGTAGGAGGCGATGAATCGGTCCACTGATTC
Chiyacao	CGGACGCTGACAAAACTCGAGAGCCTATTGCCGCCGAGGTAGGAGGCGATGAATCGGTCCACTGATTC
Hongmangmai	CGGACGCTGACAAAACTCGAGAGCCTATTGCCGCCGAGGTAGGAGGCGATGAATCGGTCCACTGATTC
Hongmai	CGGACGCTGACAAAACTCGAGAGCCTATTGCCGCCGAGGTAGGAGGCGATGAATCGGTCCACTGATTC
Wangshuibai	CGGACGCTGACAAAACTCGAGAGCCTATTGCCGCCGAGGTAGGAGGCGATGAATCGGTCCACTGATTC
Shi 4185	CGGACGCTGACAAAACTCGAGAGCCTATTGCCGCCGAGGTAGGAGGCGATGAATCGGTCCACTGATTC
Mazhamai	CGGACGCTGACAAAACTCGAGAGCCTATTGCCGCCGAGGTAGGAGGCGATGAATCGGTCCACTGATTC
Xiaofoshou	CGGACGCTGACAAAACTCGAGAGCCTATTGCCGCCGAGGTAGGAGGCGATGAATCGGTCCACTGATTC
Aikang 58	CGGACGCTGACAAAACTCGAGAGCCTATTGCCGCCGAGGTAGGAGGCGATGAATCGGTCCACTGATTC
AL8/78	CGGACGCTGACAAAACTCGAAAGCCTATTGCCGCCGAGGTAGGAGGCGATGAATCGGTCCACTGATTC
PI 431602	CATGC-CAAACAAAACTCGAAAGCCTATTGCCGCCGAGGTAGGAGGCGATGAATCGGTCCACTGCTTT
PI 486274	CATGC-CAAACAAAACTCGAAAGCCTATTTGCGCCGAGGTAGGAGGCGATGAATCGGTCCATTGCTTT
RM 000182	CGGACGCTGACAAAACTCGAAAGCCTATTGCCGCCGAGGTAGGAGGCGATGAATCGGTCCACTGATTC
PI 511381	CGGACGCTGACAAAACTCGAAAGCCTATTGCCGCCGAGGTAGGAGGCGATGAATCGGTCCACTGATTC
PI 486271	CGGACGCTGACAAAACTCGAAAGCCTATTGCCGCCGAGGTAGGAGGCGATGAATCGGTCCACTGATTC
PI 511367	CGGACGCTGACAAAACTCGAAAGCCTATTGCCGCCGAGGTAGGAGGCGATGAATCGGTCCACTGATTC
Chinese Spring	CGGACGCTGACAAAACTCGAAAGCCTATTGCCGCCGAGGTAGGAGGCGATGAATCGGTCCACTGATTC
Hanzhongbai	CGGACGCTGACAAAACTCGAAAGCCTATTGCCGCCGAGGTAGGAGGCGATGAATCGGTCCACTGATTC
Hongtoumai	CGGACGCTGACAAAACTCGAAAGCCTATTGCCGCCGAGGTAGGAGGCGATGAATCGGTCCACTGATTC
Hongyouzi	CGGACGCTGACAAAACTCGAAAGCCTATTGCCGCCGAGGTAGGAGGCGATGAATCGGTCCACTGATTC
Zijiehong	CGGACGCTGACAAAACTCGAAAGCCTATTGCCGCCGAGGTAGGAGGCGATGAATCGGTCCACTGATTC
Baihuamai	CGGACGCTGACAAAACTCGAAAGCCTATTGCCGCCGAGGTAGGAGGCGATGAATCGGTCCACTGATTC
Huixianhong	CGGACGCTGACAAAACTCGAAAGCCTATTGCCGCCGAGGTAGGAGGCGATGAATCGGTCCACTGATTC
Baimangmai	CGGACGCTGACAAAACTCGAAAGCCTATTGCCGCCGAGGTAGGAGGCGATGAATCGGTCCACTGATTC

```
                 4,090     4,100     4,110     4,120     4,130     4,140
Hulutou          CATCTTGGACTCCTGAAAGCAAACAATGTGGCAAGAGGAGGAGGCAATCGTCTCATGAACCGTCGCCC
Baihulu          CATCTTGGACTCCTGAAAGCAAACAATGTGGCAAGAGGAGGAGGCAATCGTCTCATGAACCGTCGCCC
Chiyacao         CATCTTGGACTCCTGAAAGCAAACAATGTGGCAAGAGGAGGAGGCAATCGTCTCATGAACCGTCGCCC
Hongmangmai      CATCTTGGACTCCTGAAAGCAAACAATGTGGCAAGAGGAGGAGGCAATCGTCTCATGAACCGTCGCCC
Hongmai          CATCTTGGACTCCTGAAAGCAAACAATGTGGCAAGAGGAGGAGGCAATCGTCTCATGAACCGTCGCCC
Wangshuibai      CATCTTGGACTCCTGAAAGCAAACAATGTGGCAAGAGGAGGAGGCAATCGTCTCATGAACCGTCGCCC
Shi 4185         CATCTTGGACTCCTGAAAGCAAACAATGTGGCAAGAGGAGGAGGCAATCGTCTCATGAACCGTCGCCC
Mazhamai         CATCTTGGACTCCTGAAAGCAAACAATGTGGCAAGAGGAGGAGGCAATCGTCTCATGAACCGTCGCCC
Xiaofoshou       CATCTTGGACTCCTGAAAGCAAACAATGTGGCAAGAGGAGGAGGCAATCGTCTCATGAACCGTCGCCC
Aikang 58        CATCTTGGACTCCTGAAAGCAAACAATGTGGCAAGAGGAGGAGGCAATCGTCTCATGAACCGTCGCCC
AL8/78           CATCTTGGACTCCTGAAAGCAAACAATGTGGCAAGAGGAGGAGGCAATCGTCTCATGAACCGTCGCCC
PI 431602        CTTAGAAAACTCCTGAAAGCAAACAATGTGGCAAGAGGAGGAGGCAATCGTATCAGGAAACGTCCCAC
PI 486274        CTTAGAAAACTCCTTCAAGCAAACACTGTGGCAAGAGGCGGAGGCAATAATATCAGGAAAGCTCCCAA
RM 000182        CATCTTGGG-------AAGCAAACAATGTGGCAAGAGGAGGAGGCAATCGTCTCATGAACCGTCGCCC
PI 511381        CATCTTGGG-------AAGCAAACAATGTGGCAAGAGGAGGAGGCAATCGTCTCATGAACCGTCGCCC
PI 486271        CATCTTGGG-------AAGCAAACAATGTGGCAAGAGGAGGAGGCAATCGTCTCATGAACCGTCGCCC
PI 511367        CATCTTGGG-------AAGCAAACAATGTGGCAAGAGGAGGAGGCAATCGTCTCATGAACCGTCGCCC
Chinese Spring   CATCTTGGACTCCTGAAAGCAAACAATGTGGCAAGAGGAGGAGGCAATCGTCTCATGAACCGTCGCCC
Hanzhongbai      CATCTTGGACTCCTGAAAGCAAACAATGTGGCAAGAGGAGGAGGCAATCGTCTCATGAACCGTCGCCC
Hongtoumai       CATCTTGGACTCCTGAAAGCAAACAATGTGGCAAGAGGAGGAGGCAATCGTCTCATGAACCGTCGCCC
Hongyouzi        CATCTTGGACTCCTGAAAGCAAACAATGTGGCAAGAGGAGGAGGCAATCGTCTCATGAACCGTCGCCC
Zijiehong        CATCTTGGACTCCTGAAAGCAAACAATGTGGCAAGAGGAGGAGGCAATCGTCTCATGAACCGTCGCCC
Baihuamai        CATCTTGGACTCCTGAAAGCAAACAATGTGGCAAGAGGAGGAGGCAATCGTCTCATGAACCGTCGCCC
Huixianhong      CATCTTGGACTCCTGAAAGCAAACAATGTGGCAAGAGGAGGAGGCAATCGTCTCATGAACCGTCGCCC
Baimangmai       CATCTTGGACTCCTGAAAGCAAACAATGTGGCAAGAGGAGGAGGCAATCGTCTCATGAACCGTCGCCC

                 4,150     4,160     4,170     4,180     4,190     4,200     4,210
Hulutou          GTCTACCTGGGTTGCCAAATTTTGATAATTGGTTTGATGCCAAAAATTGGCATGCCAAATTTTGGCAA
Baihulu          GTCTACCTGGGTTGCCAAATTTTGATAATTGGTTTGATGCCAAAAATTGGCATGCCAAATTTTGGCAA
Chiyacao         GTCTACCTGGGTTGCCAAATTTTGATAATTGGTTTGATGCCAAAAATTGGCATGCCAAATTTTGGCAA
Hongmangmai      GTCTACCTGGGTTGCCAAATTTTGATAATTGGTTTGATGCCAAAAATTGGCATGCCAAATTTTGGCAA
Hongmai          GTCTACCTGGGTTGCCAAATTTTGATAATTGGTTTGATGCCAAAAATTGGCATGCCAAATTTTGGCAA
Wangshuibai      GTCTACCTGGGTTGCCAAATTTTGATAATTGGTTTGATGCCAAAAATTGGCATGCCAAATTTTGGCAA
Shi 4185         GTCTACCTGGGTTGCCAAATTTTGATAATTGGTTTGATGCCAAAAATTGGCATGCCAAATTTTGGCAA
Mazhamai         GTCTACCTGGGTTGCCAAATTTTGATAATTGGTTTGATGCCAAAAATTGGCATGCCAAATTTTGGCAA
Xiaofoshou       GTCTACCTGGGTTGCCAAATTTTGATAATTGGTTTGATGCCAAAAATTGGCATGCCAAATTTTGGCAA
Aikang 58        GTCTACCTGGGTTGCCAAATTTTGATAATTGGTTTGATGCCAAAAATTGGCATGCCAAATT------A
AL8/78           GTCTACCTGGGTTGCCAAATTTTGATAATTGGTTTGATGCCAAAAATTGGCATGCCAAATTTTGGCAA
PI 431602        TTTTTCGTTGGTTGTCAAATTTTGAT------------------------------------------
PI 486274        TTTTTCGTGGATATCCGGCTTTTGAT------------------------------------------
RM 000182        GTCTATCTGGGTTGCCAAATTTTGATAATTGGTTTGATGCCAAAAATTGGCATGCCAAATTTTGGCAA
PI 511381        GTCTATCTGGGTTGCCAAATTTTGATAATTGGTTTGATGCCAAAAATTGGCATGCCAAATTTTGGCAA
PI 486271        GTCTATCTGGGTTGCCAAATTTTGATAATTGGTTTGATGCCAAAAATTGGCATGCCAAATTTTGGCAA
PI 511367        GTCTATCTGGGTTGCCAAATTTTGATAATTGGTTTGATGCCAAAAATTGGCATGCCAAATTTTGGCAA
Chinese Spring   GTCTACCTGGGTTGCCAAATTTTGATAATTGGTTTGATGCCAAAAATTGGCATGCCAAATTTTGGCAA
Hanzhongbai      GTCTACCTGGGTTGCCAAATTTTGATAATTGGTTTGATGCCAAAAATTGGCATGCCAAATTTTGGCAA
Hongtoumai       GTCTACCTGGGTTGCCAAATTTTGATAATTGGTTTGATGCCAAAAATTGGCATGCCAAATTTTGGCAA
Hongyouzi        GTCTACCTGGGTTGCCAAATTTTGATAATTGGTTTGATGCCAAAAATTGGCATGCCAAATTTTGGCAA
Zijiehong        GTCTACCTGGGTTGCCAAATTTTGATAATTGGTTTGATGCCAAAAATTGGCATGCCAAATTTTGGCAA
Baihuamai        GTCTACCTGGGTTGCCAAATTTTGATAATTGGTTTGATGCCAAAAATTGGCATGCCAAATTTTGGCAA
Huixianhong      GTCTACCTGGGTTGCCAAATTTTGATAATTGGTTTGATGCCAAAAATTGGCATGCCAAATTTTGGCAA
Baimangmai       GTCTACCTGGGTTGCCAAATTTTGATAATTGGTTTGATGCCAAAAATTGGCATGCCAAATTTTGGCAA
```

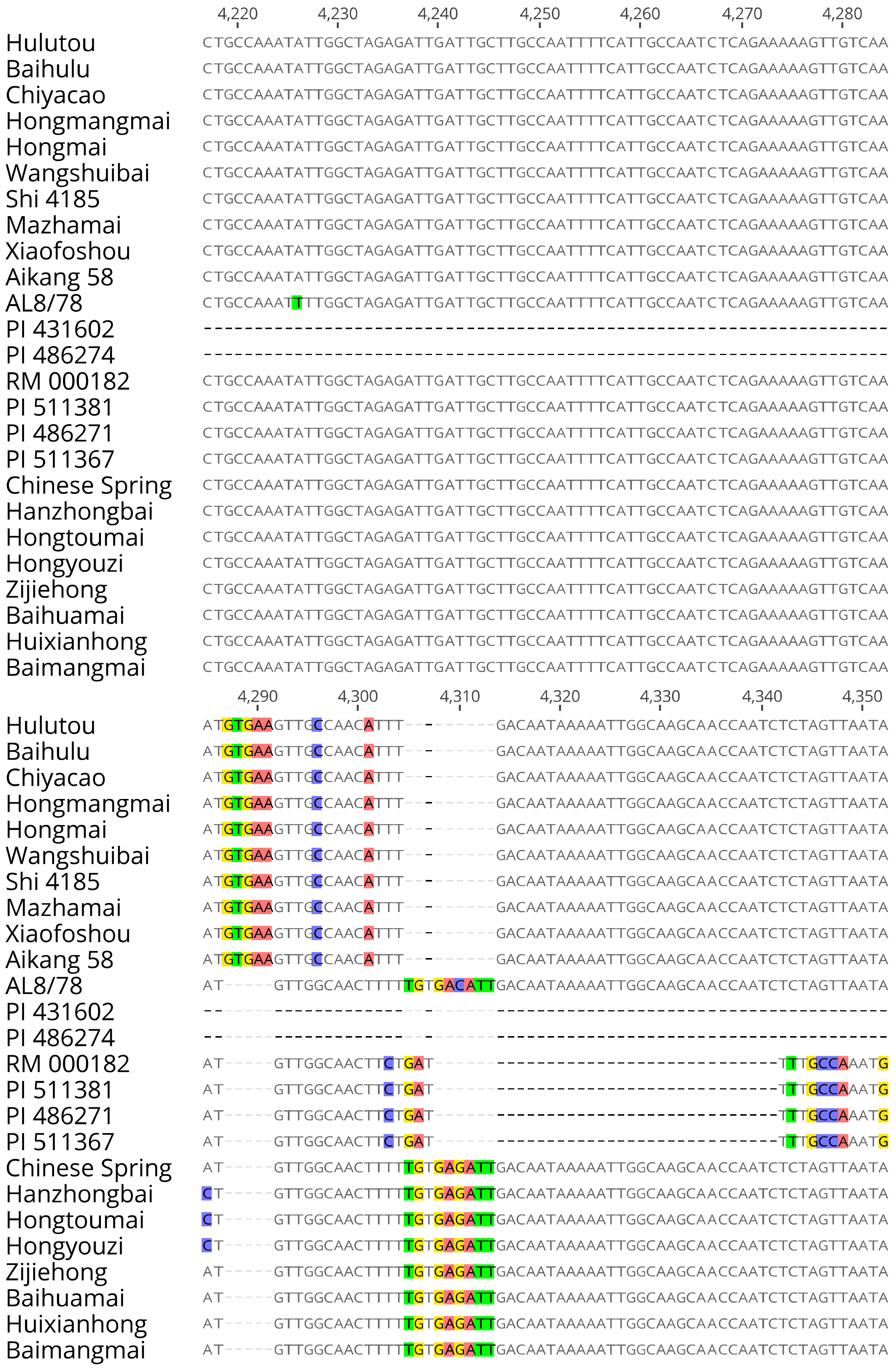

4,220
4,230
4,240
4,250
4,260
4,270
4,280
Hulutou CTGCCAAATATTGGCTAGAGATTGATTGCTTGCCAATTTTCATTGCCAATCTCAGAAAAAGTTGTCAA
Baihulu CTGCCAAATATTGGCTAGAGATTGATTGCTTGCCAATTTTCATTGCCAATCTCAGAAAAAGTTGTCAA
Chiyacao CTGCCAAATATTGGCTAGAGATTGATTGCTTGCCAATTTTCATTGCCAATCTCAGAAAAAGTTGTCAA
Hongmangmai CTGCCAAATATTGGCTAGAGATTGATTGCTTGCCAATTTTCATTGCCAATCTCAGAAAAAGTTGTCAA
Hongmai CTGCCAAATATTGGCTAGAGATTGATTGCTTGCCAATTTTCATTGCCAATCTCAGAAAAAGTTGTCAA
Wangshuibai CTGCCAAATATTGGCTAGAGATTGATTGCTTGCCAATTTTCATTGCCAATCTCAGAAAAAGTTGTCAA
Shi 4185 CTGCCAAATATTGGCTAGAGATTGATTGCTTGCCAATTTTCATTGCCAATCTCAGAAAAAGTTGTCAA
Mazhamai CTGCCAAATATTGGCTAGAGATTGATTGCTTGCCAATTTTCATTGCCAATCTCAGAAAAAGTTGTCAA
Xiaofoshou CTGCCAAATATTGGCTAGAGATTGATTGCTTGCCAATTTTCATTGCCAATCTCAGAAAAAGTTGTCAA
Aikang 58 CTGCCAAATATTGGCTAGAGATTGATTGCTTGCCAATTTTCATTGCCAATCTCAGAAAAAGTTGTCAA
AL8/78 CTGCCAAATTTTGGCTAGAGATTGATTGCTTGCCAATTTTCATTGCCAATCTCAGAAAAAGTTGTCAA
PI 431602 --
PI 486274 --
RM 000182 CTGCCAAATATTGGCTAGAGATTGATTGCTTGCCAATTTTCATTGCCAATCTCAGAAAAAGTTGTCAA
PI 511381 CTGCCAAATATTGGCTAGAGATTGATTGCTTGCCAATTTTCATTGCCAATCTCAGAAAAAGTTGTCAA
PI 486271 CTGCCAAATATTGGCTAGAGATTGATTGCTTGCCAATTTTCATTGCCAATCTCAGAAAAAGTTGTCAA
PI 511367 CTGCCAAATATTGGCTAGAGATTGATTGCTTGCCAATTTTCATTGCCAATCTCAGAAAAAGTTGTCAA
Chinese Spring CTGCCAAATATTGGCTAGAGATTGATTGCTTGCCAATTTTCATTGCCAATCTCAGAAAAAGTTGTCAA
Hanzhongbai CTGCCAAATATTGGCTAGAGATTGATTGCTTGCCAATTTTCATTGCCAATCTCAGAAAAAGTTGTCAA
Hongtoumai CTGCCAAATATTGGCTAGAGATTGATTGCTTGCCAATTTTCATTGCCAATCTCAGAAAAAGTTGTCAA
Hongyouzi CTGCCAAATATTGGCTAGAGATTGATTGCTTGCCAATTTTCATTGCCAATCTCAGAAAAAGTTGTCAA
Zijiehong CTGCCAAATATTGGCTAGAGATTGATTGCTTGCCAATTTTCATTGCCAATCTCAGAAAAAGTTGTCAA
Baihuamai CTGCCAAATATTGGCTAGAGATTGATTGCTTGCCAATTTTCATTGCCAATCTCAGAAAAAGTTGTCAA
Huixianhong CTGCCAAATATTGGCTAGAGATTGATTGCTTGCCAATTTTCATTGCCAATCTCAGAAAAAGTTGTCAA
Baimangmai CTGCCAAATATTGGCTAGAGATTGATTGCTTGCCAATTTTCATTGCCAATCTCAGAAAAAGTTGTCAA
4,290
4,300
4,310
4,320
4,330
4,340
4,350
Hulutou ATGTGAAGTTGCCAACATTT---------GACAATAAAAATTGGCAAGCAACCAATCTCTAGTTAATA
Baihulu ATGTGAAGTTGCCAACATTT---------GACAATAAAAATTGGCAAGCAACCAATCTCTAGTTAATA
Chiyacao ATGTGAAGTTGCCAACATTT---------GACAATAAAAATTGGCAAGCAACCAATCTCTAGTTAATA
Hongmangmai ATGTGAAGTTGCCAACATTT---------GACAATAAAAATTGGCAAGCAACCAATCTCTAGTTAATA
Hongmai ATGTGAAGTTGCCAACATTT---------GACAATAAAAATTGGCAAGCAACCAATCTCTAGTTAATA
Wangshuibai ATGTGAAGTTGCCAACATTT---------GACAATAAAAATTGGCAAGCAACCAATCTCTAGTTAATA
Shi 4185 ATGTGAAGTTGCCAACATTT---------GACAATAAAAATTGGCAAGCAACCAATCTCTAGTTAATA
Mazhamai ATGTGAAGTTGCCAACATTT---------GACAATAAAAATTGGCAAGCAACCAATCTCTAGTTAATA
Xiaofoshou ATGTGAAGTTGCCAACATTT---------GACAATAAAAATTGGCAAGCAACCAATCTCTAGTTAATA
Aikang 58 ATGTGAAGTTGCCAACATTT---------GACAATAAAAATTGGCAAGCAACCAATCTCTAGTTAATA
AL8/78 AT-----GTTGGCAACTTTTTGTGACATTGACAATAAAAATTGGCAAGCAACCAATCTCTAGTTAATA
PI 431602 --
PI 486274 --
RM 000182 AT-----GTTGGCAACTTCTGAT----------------------------------TTTGCCAAATG
PI 511381 AT-----GTTGGCAACTTCTGAT----------------------------------TTTGCCAAATG
PI 486271 AT-----GTTGGCAACTTCTGAT----------------------------------TTTGCCAAATG
PI 511367 AT-----GTTGGCAACTTCTGAT----------------------------------TTTGCCAAATG
Chinese Spring AT-----GTTGGCAACTTTTTGTGAGATTGACAATAAAAATTGGCAAGCAACCAATCTCTAGTTAATA
Hanzhongbai CT-----GTTGGCAACTTTTTGTGAGATTGACAATAAAAATTGGCAAGCAACCAATCTCTAGTTAATA
Hongtoumai CT-----GTTGGCAACTTTTTGTGAGATTGACAATAAAAATTGGCAAGCAACCAATCTCTAGTTAATA
Hongyouzi CT-----GTTGGCAACTTTTTGTGAGATTGACAATAAAAATTGGCAAGCAACCAATCTCTAGTTAATA
Zijiehong AT-----GTTGGCAACTTTTTGTGAGATTGACAATAAAAATTGGCAAGCAACCAATCTCTAGTTAATA
Baihuamai AT-----GTTGGCAACTTTTTGTGAGATTGACAATAAAAATTGGCAAGCAACCAATCTCTAGTTAATA
Huixianhong AT-----GTTGGCAACTTTTTGTGAGATTGACAATAAAAATTGGCAAGCAACCAATCTCTAGTTAATA
Baimangmai AT-----GTTGGCAACTTTTTGTGAGATTGACAATAAAAATTGGCAAGCAACCAATCTCTAGTTAATA

Accession	4,360 – 4,420
Hulutou	TTTGGCACTTGCCAAAATTTGGC-ATGCCAACTTTTAGCATCAAACCAATTATGCTCTTAACCCTTGC
Baihulu	TTTGGCACTTGCCAAAATTTGGC-ATGCCAACTTTTAGCATCAAACCAATTATGCTCTTAACCCTTGC
Chiyacao	TTTGGCACTTGCCAAAATTTGGC-ATGCCAACTTTTAGCATCAAACCAATTATGCTCTTAACCCTTGC
Hongmangmai	TTTGGCACTTGCCAAAATTTGGC-ATGCCAACTTTTAGCATCAAACCAATTATGCTCTTAACCCTTGC
Hongmai	TTTGGCACTTGCCAAAATTTGGC-ATGCCAACTTTTAGCATCAAACCAATTATGCTCTTAACCCTTGC
Wangshuibai	TTTGGCACTTGCCAAAATTTGGC-ATGCCAACTTTTAGCATCAAACCAATTATGCTCTTAACCCTTGC
Shi 4185	TTTGGCACTTGCCAAAATTTGGC-ATGCCAACTTTTAGCATCAAACCAATTATGCTCTTAACCCTTGC
Mazhamai	TTTGGCACTTGCCAAAATTTGGC-ATGCCAACTTTTAGCATCAAACCAATTATGCTCTTAACCCTTGC
Xiaofoshou	TTTGGCACTTGCCAAAATTTGGC-ATGCCAACTTTTAGCATCAAACCAATTATGCTCTTAACCCTTGC
Aikang 58	TTTGGCACTTGCCAAAATTTGGC-ATGCCAACTTTTAGCATCAAACCAATTATGCTCTTAACCCTTGC
AL8/78	TTTGGCACTTGCCAAAATTTGGC-ATGCCAACTTTTAGCATCAAACCAATTATGCTCTTAACCCTTGC
PI 431602	-------------AAAATGTGGG-ATGCCAACTTTTAGCATCAAACCAATTATGCTCTTAACCCTTGC
PI 486274	-------------AAAATTTGGG-ATGCCAACTTTTAGCATCAAACCAATTATGCTCTTAACCCTTGC
RM 000182	TTTGG--CTTGCCAAGTATTGGCAATGCCAAATGTTGGTAGCAAACCAATTATGCTCTTAACCCTTGC
PI 511381	TTTGG--CTTGCCAAGTATTGGCAATGCCAAATGTTGGTAGCAAACCAATTATGCTCTTAACCCTTGC
PI 486271	TTTGG--CTTGCCAAGTATTGGCAATGCCAAATGTTGGTAGCAAACCAATTATGCTCTTAACCCTTGC
PI 511367	TTTGG--CTTGCCAAGTATTGGCAATGCCAAATGTTGGTAGCAAACCAATTATGCTCTTAACCCTTGC
Chinese Spring	TTTGGCACTTGCCAAAATTTGGC-ATGCCAACTTTTAGCATCAAACCAATTATGCTCTTGACCCTTGC
Hanzhongbai	TTTGGCACTTGCCAAAATTTGGC-ATGCCAACTTTTAGCATCAAACCAATTATGCTCTTGACCCTTGC
Hongtoumai	TTTGGCACTTGCCAAAATTTGGC-ATGCCAACTTTTAGCATCAAACCAATTATGCTCTTGACCCTTGC
Hongyouzi	TTTGGCACTTGCCAAAATTTGGC-ATGCCAACTTTTAGCATCAAACCAATTATGCTCTTGACCCTTGC
Zijiehong	TTTGGCACTTGCCAAAATTTGGC-ATGCCAACTTTTAGCATCAAACCAATTATGCTCTTGACCCTTGC
Baihuamai	TTTGGCACTTGCCAAAATTTGGC-ATGCCAACTTTTAGCATCAAACCAATTATGCTCTTGACCCTTGC
Huixianhong	TTTGGCACTTGCCAAAATTTGGC-ATGCCAACTTTTAGCATCAAACCAATTATGCTCTTGACCCTTGC
Baimangmai	TTTGGCACTTGCCAAAATTTGGC-ATGCCAACTTTTTACATCAAACCAATTATGCTCTTGACCCTTGC

Accession	4,430 – 4,480
Hulutou	AGATTTCAACTCAAAAAAAATTATGTTTTGTTCTATCATCAGGAAACTAGGGGTCTCGTGGCACTCAC
Baihulu	AGATTTCAACTCAAAAAAAATTATGTTTTGTTCTATCATCAGGAAACTAGGGGTCTCGTGGCACTCAC
Chiyacao	AGATTTCAACTCAAAAAAAATTATGTTTTGTTCTATCATCAGGAAACTAGGGGTCTCGTGGCACTCAC
Hongmangmai	AGATTTCAACTCAAAAAAAATTATGTTTTGTTCTATCATCAGGAAACTAGGGGTCTCGTGGCACTCAC
Hongmai	AGATTTCAACTCAAAAAAAATTATGTTTTGTTCTATCATCAGGAAACTAGGGGTCTCGTGGCACTCAC
Wangshuibai	AGATTTCAACTCAAAAAAAATTATGTTTTGTTCTATCATCAGGAAACTAGGGGTCTCGTGGCACTCAC
Shi 4185	AGATTTCAACTCAAAAAAAATTATGTTTTGTTCTATCATCAGGAAACTAGGGGTCTCGTGGCACTCAC
Mazhamai	AGATTTCAACTCAAAAAAAATTATGTTTTGTTCTATCATCAGGAAACTAGGGGTCTCGTGGCACTCAC
Xiaofoshou	AGATTTCAACTCAAAAAAAATTATGTTTTGTTCTATCATCAGGAAACTAGGGGTCTCGTGGCACTCAC
Aikang 58	AGATTTCAACTCAAAAAAAATTATGTTTTGTTCTATCATCAGGAAACTAGGGGTCTCGTGGCACTCAC
AL8/78	AGATTTCAACTCAAAAAAATT-ATGTTTTGTTCTATCATCAGGAAACTAGGGGTCTCGTGGCACTCAC
PI 431602	AGATTTCAACTCAAAAAAATT-ATGTTTTGTTCTATCATCAGGAAACTAGGGGTCTCGTGGCACTCAC
PI 486274	AGATTTCAACTCAAAAAAATT-ATGTTTTGTTCTATCATCAGGAAACTAGGGGTCTCGTGGCACTCAC
RM 000182	AGATTTCAACTCAAAAAAAATTATGTTTTGTTCTATCATCAGGAAACTAGGGGTCTCGTGGCACTCAC
PI 511381	AGATTTCAACTCAAAAAAAATTATGTTTTGTTCTATCATCAGGAAACTAGGGGTCTCGTGGCACTCAC
PI 486271	AGATTTCAACTCAAAAAAAATTATGTTTTGTTCTATCATCAGGAAACTAGGGGTCTCGTGGCACTCAC
PI 511367	AGATTTCAACTCAAAAAAAATTATGTTTTGTTCTATCATCAGGAAACTAGGGGTCTCGTGGCACTCAC
Chinese Spring	AGATTTCAACTCAAAAAAAT---TGTTTTGTTCTATCATCAGGAAACTAGGGGTCTCGTGGCACTCAC
Hanzhongbai	AGATTTCAACTCAAAAAAAT---TGTTTTGTTCTATCATCAGGAAACTAGGGGTCTCGTGGCACTCAC
Hongtoumai	AGATTTCAACTCAAAAAAAT---TGTTTTGTTCTATCATCAGGAAACTAGGGGTCTCGTGGCACTCAC
Hongyouzi	AGATTTCAACTCAAAAAAAT---TGTTTTGTTCTATCATCAGGAAACTAGGGGTCTCGTGGCACTCAC
Zijiehong	AGATTTCAACTCAAAAAAAT---TGTTTTGTTCTATCATCAGGAAACTAGGGGTCTCGTGGCACTCAC
Baihuamai	AGATTTCAACTCAAAAAAAT---TGTTTTGTTCTATCATCAGGAAACTAGGGGTCTCGTGGCACTCAC
Huixianhong	AGATTTCAACTCAAAAAAAT---TGTTTTGTTCTATCATCAGGAAACTAGGGGTCTCGTGGCACTCAC
Baimangmai	AGATTTCAACTCAAAAAAAT---TGTTTTGTTCTATCATCAGGAAACTAGGGGTCTCGTGGCACTCAC

	4,490 · 4,500 · 4,510 · 4,520 · 4,530 · 4,540 · 4,550
Hulutou	AATTAGAGCCCGTCGGGGTTGCATGAAGTGTGCTTGCTGAACCAGCTCTTTGCACAACAAAGAGAGAA
Baihulu	AATTAGAGCCCGTCGGGGTTGCATGAAGTGTGCTTGCTGAACCAGCTCTTTGCACAACAAAGAGAGAA
Chiyacao	AATTAGAGCCCGTCGGGGTTGCATGAAGTGTGCTTGCTGAACCAGCTCTTTGCACAACAAAGAGAGAA
Hongmangmai	AATTAGAGCCCGTCGGGGTTGCATGAAGTGTGCTTGCTGAACCAGCTCTTTGCACAACAAAGAGAGAA
Hongmai	AATTAGAGCCCGTCGGGGTTGCATGAAGTGTGCTTGCTGAACCAGCTCTTTGCACAACAAAGAGAGAA
Wangshuibai	AATTAGAGCCCGTCGGGGTTGCATGAAGTGTGCTTGCTGAACCAGCTCTTTGCACAACAAAGAGAGAA
Shi 4185	AATTAGAGCCCGTCGGGGTTGCATGAAGTGTGCTTGCTGAACCAGCTCTTTGCACAACAAAGAGAGAA
Mazhamai	AATTAGAGCCCGTCGGGGTTGCATGAAGTGTGCTTGCTGAACCAGCTCTTTGCACAACAAAGAGAGAA
Xiaofoshou	AATTAGAGCCCGTCGGGGTTGCATGAAGTGTGCTTGCTGAACCAGCTCTTTGCACAACAAAGAGAGAA
Aikang 58	AATTAGAGCCCGTCGGGGTTGCATGAAGTGTGCTTGCTGAACCAGCTCTTTGCACAACAAAGAGAGAA
AL8/78	AATTAGAGCCCGTCGGGGTTGCATGAAGTGTGCTTGCTGAACCAGCTCTTTGCACAACAAAGAGAGAA
PI 431602	AATTAGAGCCCGTCGGGGTTGCATGAAGTGTGCTTGCTGAACCAGCTCTTTGCACAACAAAGAGAGAA
PI 486274	AATTAGAGCCCGTCGGGGTTGCATTAAGTGTGCTTGCTGAACCAGCTCTTTGCACAACAAAGAGAGAA
RM 000182	AATTAGAGCCCGTCGGGGTTGCATGAAGTGTGCTTGCTGAACCAGCTCTTTGCACAACAAAGAGAGAA
PI 511381	AATTAGAGCCCGTCGGGGTTGCATGAAGTGTGCTTGCTGAACCAGCTCTTTGCACAACAAAGAGAGAA
PI 486271	AATTAGAGCCCGTCGGGGTTGCATGAAGTGTGCTTGCTGAACCAGCTCTTTGCACAACAAAGAGAGAA
PI 511367	AATTAGAGCCCGTCGGGGTTGCATGAAGTGTGCTTGCTGAACCAGCTCTTTGCACAACAAAGAGAGAA
Chinese Spring	AATTAGAGCCCGTCGGGGTTGCATGAAGTGTGCTTGCTGAACCAGCTCTTTGCACAACAAAGAGAGAA
Hanzhongbai	AATTAGAGCCCGTCGGGGTTGCATGAAGTGTGCTTGCTGAACCAGCTCTTTGCACAACAAAGAGAGAA
Hongtoumai	AATTAGAGCCCGTCGGGGTTGCATGAAGTGTGCTTGCTGAACCAGCTCTTTGCACAACAAAGAGAGAA
Hongyouzi	AATTAGAGCCCGTCGGGGTTGCATGAAGTGTGCTTGCTGAACCAGCTCTTTGCACAACAAAGAGAGAA
Zijiehong	AATTAGAGCCCGTCGGGGTTGCATGAAGTGTGCTTGCTGAACCAGCTCTTTGCACAACAAAGAGAGAA
Baihuamai	AATTAGAGCCCGTCGGGGTTGCATGAAGTGTGCTTGCTGAACCAGCTCTTTGCACAACAAAGAGAGAA
Huixianhong	AATTAGAGCCCGTCGGGGTTGCATGAAGTGTGCTTGCTGAACCAGCTCTTTGCACAACAAAGAGAGAA
Baimangmai	AATTAGAGCCCGTCGGGGTTGCATGAAGTGTGCTTGCTGAACCAGCTCTTTGCACAACAAAGAGAGAA

	4,560 · 4,570 · 4,580 · 4,590 · 4,600 · 4,610 · 4,620
Hulutou	ATAATCTGGAAGCTGCGTGGAGTTTGAGGTCAGGGTGGCTGCTGTCTAGAGTAGAGGTTGCAACTTGC
Baihulu	ATAATCTGGAAGCTGCGTGGAGTTTGAGGTCAGGGTGGCTGCTGTCTAGAGTAGAGGTTGCAACTTGC
Chiyacao	ATAATCTGGAAGCTGCGTGGAGTTTGAGGTCAGGGTGGCTGCTGTCTAGAGTAGAGGTTGCAACTTGC
Hongmangmai	ATAATCTGGAAGCTGCGTGGAGTTTGAGGTCAGGGTGGCTGCTGTCTAGAGTAGAGGTTGCAACTTGC
Hongmai	ATAATCTGGAAGCTGCGTGGAGTTTGAGGTCAGGGTGGCTGCTGTCTAGAGTAGAGGTTGCAACTTGC
Wangshuibai	ATAATCTGGAAGCTGCGTGGAGTTTGAGGTCAGGGTGGCTGCTGTCTAGAGTAGAGGTTGCAACTTGC
Shi 4185	ATAATCTGGAAGCTGCGTGGAGTTTGAGGTCAGGGTGGCTGCTGTCTAGAGTAGAGGTTGCAACTTGC
Mazhamai	ATAATCTGGAAGCTGCGTGGAGTTTGAGGTCAGGGTGGCTGCTGTCTAGAGTAGAGGTTGCAACTTGC
Xiaofoshou	ATAATCTGGAAGCTGCGTGGAGTTTGAGGTCAGGGTGGCTGCTGTCTAGAGTAGAGGTTGCAACTTGC
Aikang 58	ATAATCTGGAAGCTGCGTGGAGTTTGAGGTCAGGGTGGCTGCTGTCTAGAGTAGAGGTTGCAACTTGC
AL8/78	ATAATCTGGAAGCTGCGTGGAGTTTGAGGTCAGGGTGGCTGCTGTCTAGAGTAGAGGTTGCAACTTGC
PI 431602	ATAATCTGGAAGCTGCGTGGAGTTTGAGGTCAGGGTGGCTGCTGTCTAGAGTAGAGGTTGCAACTTGC
PI 486274	ATAATCTGGAAGCTGCGTGGAGTTTGAGGTCAGGGTGGCTGCTGTCTAGAGTAGAGGTTGCAACTTGC
RM 000182	ATAATCTGGAAGCTGCGTGGAGTTTGAGGTCAGGGTGGCTGCTGTCTAGAGTAGAGGTTGCAACTTGC
PI 511381	ATAATCTGGAAGCTGCGTGGAGTTTGAGGTCAGGGTGGCTGCTGTCTAGAGTAGAGGTTGCAACTTGC
PI 486271	ATAATCTGGAAGCTGCGTGGAGTTTGAGGTCAGGGTGGCTGCTGTCTAGAGTAGAGGTTGCAACTTGC
PI 511367	ATAATCTGGAAGCTGCGTGGAGTTTGAGGTCAGGGTGGCTGCTGTCTAGAGTAGAGGTTGCAACTTGC
Chinese Spring	ATAATCTGGAAGCTGCGTGGAGTTTGAGGTCAGGGTGGCTGCTGTCTAGAGTAGAGGTTGCAACTTGC
Hanzhongbai	ATAATCTGGAAGCTGCGTGGAGTTTGAGGTCAGGGTGGCTGCTGTCTAGAGTAGAGGTTGCAACTTGC
Hongtoumai	ATAATCTGGAAGCTGCGTGGAGTTTGAGGTCAGGGTGGCTGCTGTCTAGAGTAGAGGTTGCAACTTGC
Hongyouzi	ATAATCTGGAAGCTGCGTGGAGTTTGAGGTCAGGGTGGCTGCTGTCTAGAGTAGAGGTTGCAACTTGC
Zijiehong	ATAATCTGGAAGCTGCGTGGAGTTTGAGGTCAGGGTGGCTGCTGTCTAGAGTAGAGGTTGCAACTTGC
Baihuamai	ATAATCTGGAAGCTGCGTGGAGTTTGAGGTCAGGGTGGCTGCTGTCTAGAGTAGAGGTTGCAACTTGC
Huixianhong	ATAATCTGGAAGCTGCGTGGAGTTTGAGGTCAGGGTGGCTGCTGTCTAGAGTAGAGGTTGCAACTTGC
Baimangmai	ATAATCTGGAAGCTGCGTGGAGTTTGAGGTCAGGGTGGCTGCTGTCTAGAGTAGAGGTTGCAACTTGC

```
                    4,630       4,640       4,650       4,660       4,670       4,680       4,690
Hulutou          AAGCGATCTTTGCACAACCATGATCTTCATTAAGCACTGGCGACGATGAACACCCAAGTTACAACCAA
Baihulu          AAGCGATCTTTGCACAACCATGATCTTCATTAAGCACTGGCGACGATGAACACCCAAGTTACAACCAA
Chiyacao         AAGCGATCTTTGCACAACCATGATCTTCATTAAGCACTGGCGACGATGAACACCCAAGTTACAACCAA
Hongmangmai      AAGCGATCTTTGCACAACCATGATCTTCATTAAGCACTGGCGACGATGAACACCCAAGTTACAACCAA
Hongmai          AAGCGATCTTTGCACAACCATGATCTTCATTAAGCACTGGCGACGATGAACACCCAAGTTACAACCAA
Wangshuibai      AAGCGATCTTTGCACAACCATGATCTTCATTAAGCACTGGCGACGATGAACACCCAAGTTACAACCAA
Shi 4185         AAGCGATCTTTGCACAACCATGATCTTCATTAAGCACTGGCGACGATGAACACCCAAGTTACAACCAA
Mazhamai         AAGCGATCTTTGCACAACCATGATCTTCATTAAGCACTGGCGACGATGAACACCCAAGTTACAACCAA
Xiaofoshou       AAGCGATCTTTGCACAACCATGATCTTCATTAAGCACTGGCGAGGATGAACACCCAAGTTACAACCAA
Aikang 58        AAGCGATCTTTGCACAACCATGATCTTCATTAAGCACTGGCGACGATGAACACCCAAGTTACAACCAA
AL8/78           AAGCGATCTTTGCACAACCATGATCTTCATTAAGCACTGGCGACGATGAACACCCAAGTTACAACCAA
PI 431602        AAGCGATCTTTGCACAACCATGATCTTCATTAAGCACTGGCGACGATGAACACCCAAGTTACAACCAA
PI 486274        AAGCGATCTTTGCACAACCATGATCTTCATTAAGCACTGGCGACGATGAACACCCAAGTTACAACCAA
RM 000182        AAGCGATCTTTGCACAACCATGATCTTCATTAAGCACTGGCGACGATGAACACCCAAGTTACAACCAA
PI 511381        AAGCGATCTTTGCACAACCATGATCTTCATTAAGCACTGGCGACGATGAACACCCAAGTTACAACCAA
PI 486271        AAGCGATCTTTGCACAACCATGATCTTCATTAAGCACTGGCGACGATGAACACCCAAGTTACAACCAA
PI 511367        AAGCGATCTTTGCACAACCATGATCTTCATTAAGCACTGGCGACGATGAACACCCAAGTTACAACCAA
Chinese Spring   AAGCGATCTTTGCACAACCATGATCTTCATTAAGCACTGGCGACGATGAACACCCAAGTTACAACCAA
Hanzhongbai      AAGCGATCTTTGCACAACCATGATCTTCATTAAGCACTGGCGACGATGAACACCCAAGTTACAACCAA
Hongtoumai       AAGCGATCTTTGCACAACCATGATCTTCATTAAGCACTGGCGACGATGAACACCCAAGTTACAACCAA
Hongyouzi        AAGCGATCTTTGCACAACCATGATCTTCATTAAGCACTGGCGACGATGAACACCCAAGTTACAACCAA
Zijiehong        AAGCGATCTTTGCACAACCATGATCTTCATTAAGCACTGGCGACGATGAACACCCAAGTTACAACCAA
Baihuamai        AAGCGATCTTTGCACAACCATGATCTTCATTAAGCACTGGCGACGATGAACACCCAAGTTACAACCAA
Huixianhong      AAGCGATCTTTGCACAACCATGATCTTCATTAAGCACTGGCGACGATGAACACCCAAGTTACAACCAA
Baimangmai       AAGCGATCTTTGCACAACCATGATCTTCATTAAGCACTGGCGACGATGAACACCCAAGTTACAACCAA
```

```
                      4,700       4,710       4,720       4,730       4,740       4,750     4,760
Hulutou          CAGGAACGTTACAGCAACTAGTTCCTCTTGGTTAAGGGATAACAGAGAGCTTTGCTAACATCAGGGCA
Baihulu          CAGGAACGTTACAGCAACTAGTTCCTCTTGGTTAAGGGATAACAGAGAGCTTTGCTAACATCAGGGCA
Chiyacao         CAGGAACGTTACAGCAACTAGTTCCTCTTGGTTAAGGGATAACAGAGAGCTTTGCTAACATCAGGGCA
Hongmangmai      CAGGAACGTTACAGCAACTAGTTCCTCTTGGTTAAGGGATAACAGAGAGCTTTGCTAACATCAGGGCA
Hongmai          CAGGAACGTTACAGCAACTAGTTCCTCTTGGTTAAGGGATAACAGAGAGCTTTGCTAACATCAGGGCA
Wangshuibai      CAGGAACGTTACAGCAACTAGTTCCTCTTGGTTAAGGGATAACAGAGAGCTTTGCTAACATCAGGGCA
Shi 4185         CAGGAACGTTACAGCAACTAGTTCCTCTTGGTTAAGGGATAACAGAGAGCTTTGCTAACATCAGGGCA
Mazhamai         CAGGAACGTTACAGCAACTAGTTCCTCTTGGTTAAGGGATAACAGAGAGCTTTGCTAACATCAGGGCA
Xiaofoshou       CAGGAACGTTACAGCAACTAGTTCCTCTTGGTTAAGGGATAACAGAGAGCTTTGCTAACATCAGGGCA
Aikang 58        CAGGAACGTTACAGCAACTAGTTCCTCTTGGTTAAGGGATAACAGAGAGCTTTGCTAACATCAGGGCA
AL8/78           CAGGAACGTTACAGCAACTAGTTCCTCTTGGTTAAGGGATAACAGAGAGCTTTGCTAACATCAGGGCA
PI 431602        CAGGAACGTTACAGCAACTAGTTCCTCTTGGTTAAGGGATAACAGAGAGCTTTGCTAACATCAGGGCA
PI 486274        CAGGAACGTTACAGCAACTAGTTCCTCTTGGTTAAGGGATAACAGAGAGCTTTGCTAACATCAGGGCA
RM 000182        CAGGAACGTTACAGCAACTAGTTCCTCTTGGTTAAGGGATAACAGAGAGCTTTGCTAACATCAGGGCA
PI 511381        CAGGAACGTTACAGCAACTAGTTCCTC----TTAAGGGATAACAGAGAGCTTTGCTAACATCAGGGCA
PI 486271        CAGGAACGTTACAGCAACTAGTTCCTC----TTAAGGGATAACAGAGAGCTTTGCTAACATCAGGGCA
PI 511367        CAGGAACGTTACAGCAACTAGTTCCTC----TTAAGGGATAACAGAGAGCTTTGCTAACATCAGGGCA
Chinese Spring   CAGGAACGTTACAGCAACTAGTTCCTCTTGGTTAAGGGATAACAGAGAGCTTTGCTAACATCAGGGCA
Hanzhongbai      CAGGAACGTTACAGCAACTAGTTCCTCTTGGTTAAGGGATAACAGAGAGCTTTGCTAACATCAGGGCA
Hongtoumai       CAGGAACGTTACAGCAACTAGTTCCTCTTGGTTAAGGGATAACAGAGAGCTTTGCTAACATCAGGGCA
Hongyouzi        CAGGAACGTTACAGCAACTAGTTCCTCTTGGTTAAGGGATAACAGAGAGCTTTGCTAACATCAGGGCA
Zijiehong        CAGGAACGTTACAGCAACTAGTTCCTCTTGGTTAAGGGATAACAGAGAGCTTTGCTAACATCAGGGCA
Baihuamai        CAGGAACGTTACAGCAACTAGTTCCTCTTGGTTAAGGGATAACAGAGAGCTTTGCTAACATCAGGGCA
Huixianhong      CAGGAACGTTACAGCAACTAGTTCCTCTTGGTTAAGGGATAACAGAGAGCTTTGCTAACATCAGGGCA
Baimangmai       CAGGAACGTTACAGCAACTAGTTCCTCTTGGTTAAGGGATAACAGAGAGCTTTGCTAACATCAGGGCA
```

	4,770 4,780 4,790 4,800 4,810 4,820
Hulutou	ACTAGTGCTTTGGCTCTGTGAAGTGGGGAGAAACAATGGAGGCACTTGCCCACCAATTGAGGGGGGAT
Baihulu	ACTAGTGCTTTGGCTCTGTGAAGTGGGGAGAAACAATGGAGGCACTTGCCCACCAATTGAGGGGGGAT
Chiyacao	ACTAGTGCTTTGGCTCTGTGAAGTGGGGAGAAACAATGGAGGCACTTGCCCACCAATTGAGGGGGGAT
Hongmangmai	ACTAGTGCTTTGGCTCTGTGAAGTGGGGAGAAACAATGGAGGCACTTGCCCACCAATTGAGGGGGGAT
Hongmai	ACTAGTGCTTTGGCTCTGTGAAGTGGGGAGAAACAATGGAGGCACTTGCCCACCAATTGAGGGGGGAT
Wangshuibai	ACTAGTGCTTTGGCTCTGTGAAGTGGGGAGAAACAATGGAGGCACTTGCCCACCAATTGAGGGGGGAT
Shi 4185	ACTAGTGCTTTGGCTCTGTGAAGTGGGGAGAAACAATGGAGGCACTTGCCCACCAATTGAGGGGGGAT
Mazhamai	ACTAGTGCTTTGGCTCTGTGAAGTGGGGAGAAACAATGGAGGCACTTGCCCACCAATTGAGGGGGGAT
Xiaofoshou	ACTAGTGCTTTGGCTCTGTGAAGTGGGGAGAAACAATGGAGGCACTTGCCCACCAATTGAGGGGGGAT
Aikang 58	ACTAGTGCTTTGGCTCTGTGAAGTGGGGAGAAACAATGGAGGCACTTGCCCACCAATTGAGGGGGGAT
AL8/78	ACTAGTGCTTTGGCTCTGTGAAGTGGGGAGAAACAATGGAGGCACTTGCCCACCAATTGAGGGGGGAT
PI 431602	ACTAGTGCTTTGGCTCTGTGAAGTGGGGAGAAACAATGGAGGCACTTGCCCACCAATTGAGGGGGGAT
PI 486274	ACTAGTGCTTTGGCTCTGTGAAGTGGGGAGAAACAATGGAGGCACTTGCCCACCAATTGAGGGGGGAT
RM 000182	ACTAGTGCTTTGGCTCTGTGAAGTGGGGAGAAACAATGGAGGCACTTGCCCACCAATTGAGGGGGGAT
PI 511381	ACTAGTGCTTTGGCTCTGTGAAGTGGGGAGAAACAATGGAGGCACTTGCCCACCAATTGAGGGGGGAT
PI 486271	ACTAGTGCTTTGGCTCTGTGAAGTGGGGAGAAACAATGGAGGCACTTGCCCACCAATTGAGGGGGGAT
PI 511367	ACTAGTGCTTTGGCTCTGTGAAGTGGGGAGAAACAATGGAGGCACTTGCCCACCAATTGAGGGGGGAT
Chinese Spring	ACTAGTGCTTTGGCTCTGTGAAGTGGGGAGAAACAATGGAGGCACTTGCCCACCAATTGAGGGGGGAT
Hanzhongbai	ACTAGTGCTTTGGCTCTGTGAAGTGGGGAGAAACAATGGAGGCACTTGCCCACCAATTGAGGGGGGAT
Hongtoumai	ACTAGTGCTTTGGCTCTGTGAAGTGGGGAGAAACAATGGAGGCACTTGCCCACCAATTGAGGGGGGAT
Hongyouzi	ACTAGTGCTTTGGCTCTGTGAAGTGGGGAGAAACAATGGAGGCACTTGCCCACCAATTGAGGGGGGAT
Zijiehong	ACTAGTGCTTTGGCTCTGTGAAGTGGGGAGAAACAATGGAGGCACTTGCCCACCAATTGAGGGGGGAT
Baihuamai	ACTAGTGCTTTGGCTCTGTGAAGTGGGGAGAAACAATGGAGGCACTTGCCCACCAATTGAGGGGGGAT
Huixianhong	ACTAGTGCTTTGGCTCTGTGAAGTGGGGAGAAACAATGGAGGCACTTGCCCACCAATTGAGGGGGGAT
Baimangmai	ACTAGTGCTTTGGCTCTGTGAAGTGGGGAGAAACAATGGAGGCACTTGCCCACCAATTGAGGGGGGAT

	4,830 4,840 4,850 4,860 4,870 4,880 4,890
Hulutou	TTGAGGCCGCCTGTGAGGAGCTGGAGGGGGGGTTTGGCAGGCCAGGGGAGGGGA-GGGGAGGTTGTCG
Baihulu	TTGAGGCCGCCTGTGAGGAGCTGGAGGGGGGGTTTGGCAGGCCAGGGGAGGGGA-GGGGAGGTTGTCG
Chiyacao	TTGAGGCCGCCTGTGAGGAGCTGGAGGGGGGGTTTGGCAGGCCAGGGGAGGGGA-GGGGAGGTTGTCG
Hongmangmai	TTGAGGCCGCCTGTGAGGAGCTGGAGGGGGGGTTTGGCAGGCCAGGGGAGGGGA-GGGGAGGTTGTCG
Hongmai	TTGAGGCCGCCTGTGAGGAGCTGGAGGGGGGGTTTGGCAGGCCAGGGGAGGGGA-GGGGAGGTTGTCG
Wangshuibai	TTGAGGCCGCCTGTGAGGAGCTGGAGGGGGGGTTTGGCAGGCCAGGGGAGGGGA-GGGGAGGTTGTCG
Shi 4185	TTGAGGCCGCCTGTGAGGAGCTGGAGGGGGGGTTTGGCAGGCCAGGGGAGGGGA-GGGGAGGTTGTCG
Mazhamai	TTGAGGCCGCCTGTGAGGAGCTGGAGGGGGGGTTTGGCAGGCCAGGGGAGGGGA-GGGGAGGTTGTCG
Xiaofoshou	TTGAGGCCGCCTGTGAGGAGCTGGAGGGGGGGTTTGGCAGGCCAGGGGAGGGGAGGGGGAGGTTGTCG
Aikang 58	TTGAGGCCGCCTGTGAGGAGCTGGAGGGGGGGTTTGGCAGGCCAGGGGAGGGGA-GGGGAGGTTGTCG
AL8/78	TTGAGGCCGCCTGTGAGGAGCTGGAGGGGGGGTTTGGCAGGCCAGGGGAGGGGA-GGGGAGGTTGTCG
PI 431602	TTGAGGCCGCCTGTGAGGAGCTGGAGGGGGGGTTTGGCAGGCCAGGGGAGGGGA-GGGGAGGTTGTCG
PI 486274	TTGAGGCCGCCTGTGAGGAGCTGGAGGGGGGGTTTGGCAGGCCAGGGGAGGGGA-GGGGAGGTTGTCG
RM 000182	TTGAGGCCGCCTGTGAGGAGCTGGAGGGGGGGTTTGGCAGGCCAGGGGAGGGGA-GGGGAGGTTGTCG
PI 511381	TTGAGGCCGCCTGTGAGGAGCTGGAGGGGGGGTTTGGCAGGCCAGGGGAGGGGA-GGGGAGGTTGTCG
PI 486271	TTGAGGCCGCCTGTGAGGAGCTGGAGGGGGGGTTTGGCAGGCCAGGGGAGGGGA-GGGGAGGTTGTCG
PI 511367	TTGAGGCCGCCTGTGAGGAGCTGGAGGGGGGGTTTGGCAGGCCAGGGGAGGGGA-GGGGAGGTTGTCG
Chinese Spring	TTGAGGCCGCCTGTGAGGAGCTGGAGGGGGGGTTTGGCAGGCCAGGGGAGGGGA-GGGGAGGTTGTCG
Hanzhongbai	TTGAGGCCGCCTGTGAGGAGCTGGAGGGGGGGTTTGGCAGGCCAGGGGAGGGGA-GGGGAGGTTGTCG
Hongtoumai	TTGAGGCCGCCTGTGAGGAGCTGGAGGGGGGGTTTGGCAGGCCAGGGGAGGGGA-GGGGAGGTTGTCG
Hongyouzi	TTGAGGCCGCCTGTGAGGAGCTGGAGGGGGGGTTTGGCAGGCCAGGGGAGGGGA-GGGGAGGTTGTCG
Zijiehong	TTGAGGCCGCCTGTGAGGAGCTGGAGGGGGGGTTTGGCAGGCCAGGGGAGGGGA-GGGGAGGTTGTCG
Baihuamai	TTGAGGCCGCCTGTGAGGAGCTGGAGGGGGGGTTTGGCAGGCCAGGGGAGGGGA-GGGGAGGTTGTCG
Huixianhong	TTGAGGCCGCCTGTGAGGAGCTGGAGGGGGGGTTTGGCAGGCCAGGGGAGGGGAGGGGGAGGTTGTCG
Baimangmai	TTGAGGCCGCCTGTGAGGAGCTGGAGGGGGGGTTTGGCAGGCCAGGGGAGGGGA-GGGGAGGTTGTCG

```
                 4,900     4,910     4,920     4,930     4,940     4,950     4,960
Hulutou          CTGCGTCCAGCCAGGCTCCAGGGGGCATCCTGTACTTCCTCTGCAAGGCGATGCGCGGGGGCGGCGTT
Baihulu          CTGCGTCCAGCCAGGCTCCAGGGGGCATCCTGTACTTCCTCTGCAAGGCGATGCGCGGGGGCGGCGTT
Chiyacao         CTGCGTCCAGCCAGGCTCCAGGGGGCATCCTGTACTTCCTCTGCAAGGCGATGCGCGGGGGCGGCGTT
Hongmangmai      CTGCGTCCAGCCAGGCTCCAGGGGGCATCCTGTACTTCCTCTGCAAGGCGATGCGCGGGGGCGGCGTT
Hongmai          CTGCGTCCAGCCAGGCTCCAGGGGGCATCCTGTACTTCCTCTGCAAGGCGATGCGCGGGGGCGGCGTT
Wangshuibai      CTGCGTCCAGCCAGGCTCCAGGGGGCATCCTGTACTTCCTCTGCAAGGCGATGCGCGGGGGCGGCGTT
Shi 4185         CTGCGTCCAGCCAGGCTCCAGGGGGCATCCTGTACTTCCTCTGCAAGGCGATGCGCGGGGGCGGCGTT
Mazhamai         CTGCGTCCAGCCAGGCTCCAGGGGGCATCCTGTACTTCCTCTGCAAGGCGATGCGCGGGGGCGGCGTT
Xiaofoshou       CTGCGTCCAGCCAGGCTCCAGGGGGCATCCTGTACTTCCTCTGCAAGGCGATGCGCGGGGGCGGCGTT
Aikang 58        CTGCGTCCAGCCAGGCTCCAGGGGGCATCCTGTACTTCCTCTGCAAGGCGATGCGCGGGGGCGGCGTT
AL8/78           CTGCGTCCAGCCAGGCTCCAGGGGGCATCCTGTACTTCCTCTGCAAGGCGATGCGCGGGGGCGGCGTT
PI 431602        CTGCGTCCAGCCAGGCTCCAGGGGGCATCCTGTACTTCCTCTGCAAGGCGATGCGCGGGGGCGGCGTT
PI 486274        CTGCGTCCAGCCAGGCTCCAGGGGGCATCCTGTACTTTCTCTGCAAGGCGATGCGCGGGGGCGGCGTT
RM 000182        CTGCGTCCAGCCAGGCTCCAGGGGGCATCCTGTACTTCCTCTGCAAGGCGATGCGCGGGGGCGGCGTT
PI 511381        CTGCGTCCAGCCAGGCTCCAGGGGGCATCCTGTACTTCCTCTGCAAGGCGATGCGCGGGGGCGGCGTT
PI 486271        CTGCGTCCAGCCAGGCTCCAGGGGGCATCCTGTACTTCCTCTGCAAGGCGATGCGCGGGGGCGGCGTT
PI 511367        CTGCGTCCAGCCAGGCTCCAGGGGGCATCCTGTACTTCCTCTGCAAGGCGATGCGCGGGGGCGGCGTT
Chinese Spring   CTGCGTCCAGCCAGGCTCCAGGGGGCATCCTGTACTTCCTCTGCAAGGCGATGCGCGGGGGCGGCGTT
Hanzhongbai      CTGCGTCCAGCCAGGCTCCAGGGGGCATCCTGTACTTCCTCTGCAAGGCGATGCGCGGGGGCGGCGTT
Hongtoumai       CTGCGTCCAGCCAGGCTCCAGGGGGCATCCTGTACTTCCTCTGCAAGGCGATGCGCGGGGGCGGCGTT
Hongyouzi        CTGCGTCCAGCCAGGCTCCAGGGGGCATCCTGTACTTCCTCTGCAAGGCGATGCGCGGGGGCGGCGTT
Zijiehong        CTGCGTCCAGCCAGGCTCCAGGGGGCATCCTGTACTTCCTCTGCAAGGCGATGCGCGGGGGCGGCGTT
Baihuamai        CTGCGTCCAGCCAGGCTCCAGGGGGCATCCTGTACTTCCTCTGCAAGGCGATGCGCGGGGGCGGCGTT
Huixianhong      CTGCGTCCAGCCAGGCTCCAGGGGGCATCCTGTACTTCCTCTGCAAGGCGATGCGCGGGGGCGGCGTT
Baimangmai       CTGCGTCCAGCCAGGCTCCAGGGGGCATCCTGTACTTCCTCTGCAAGGCGATGCGCGGGGGCGGCGTT
                     4,970     4,980     4,990     5,000     5,010     5,020     5,030
Hulutou          TGCAGCGGAAGACCCGGGGCGGGGTTGCCGCCCGGGCATTGATGGCGGCCTCCTCTTGAATGCGTTGC
Baihulu          TGCAGCGGAAGACCCGGGGCGGGGTTGCCGCCCGGGCATTGATGGCGGCCTCCTCTTGAATGCGTTGC
Chiyacao         TGCAGCGGAAGACCCGGGGCGGGGTTGCCGCCCGGGCATTGATGGCGGCCTCCTCTTGAATGCGTTGC
Hongmangmai      TGCAGCGGAAGACCCGGGGCGGGGTTGCCGCCCGGGCATTGATGGCGGCCTCCTCTTGAATGCGTTGC
Hongmai          TGCAGCGGAAGACCCGGGGCGGGGTTGCCGCCCGGGCATTGATGGCGGCCTCCTCTTGAATGCGTTGC
Wangshuibai      TGCAGCGGAAGACCCGGGGCGGGGTTGCCGCCCGGGCATTGATGGCGGCCTCCTCTTGAATGCGTTGC
Shi 4185         TGCAGCGGAAGACCCGGGGCGGGGTTGCCGCCCGGGCATTGATGGCGGCCTCCTCTTGAATGCGTTGC
Mazhamai         TGCAGCGGAAGACCCGGGGCGGGGTTGCCGCCCGGGCATTGATGGCGGCCTCCTCTTGAATGCGTTGC
Xiaofoshou       TGCAGCGGAAGACCCGGGGCGGGGTTGCCGCCCGGGCATTGATGGCGGCCTCCTCTTGAATGCGTTGC
Aikang 58        TGCAGCGGAAGACCCGGGGCGGGGTTGCCGCCCGGGCATTGATGGCGGCCTCCTCTTGAATGCGTTGC
AL8/78           TGCAGCGGAAGACCCGGGGCGGGGTTGCCGCCCGGGCATTGATGGCGGCCTCCTCTTGAATGCGTTGC
PI 431602        TGCAGCGGAAGACCCGGGGCGGGGTTGCCGCCCGGGCATTGATGGCGGCCTCCTCTTGAATGCGTTGC
PI 486274        TGCAGCGGAAGACCCGGGGCGGGGTTGCCGCCCGGGCATTGATGGCGGCCTCCTCTTGAATGCGTTGC
RM 000182        TGCAGCGGAAGACCCGGGGCGGGGTTGCCGCCCGGGCATTGATGGCGGCCTCCTCTTGAATGCGTTGC
PI 511381        TGCAGCGGAAGACCCGGGGCGGGGTTGCCGCCCGGGCATTGATGGCGGCCTCCTCTTGAATGCGTTGC
PI 486271        TGCAGCGGAAGACCCGGGGCGGGGTTGCCGCCCGGGCATTGATGGCGGCCTCCTCTTGAATGCGTTGC
PI 511367        TGCAGCGGAAGACCCGGGGCGGGGTTGCCGCCCGGGCATTGATGGCGGCCTCCTCTTGAATGCGTTGC
Chinese Spring   TGCAGCGGAAGACCCGGGGCGGGGTTGCCGCCCGGGCATTGATGGCGGCCTCCTCTTGAATGCGTTGC
Hanzhongbai      TGCAGCGGAAGACCCGGGGCGGGGTTGCCGCCCGGGCATTGATGGCGGCCTCCTCTTGAATGCGTTGC
Hongtoumai       TGCAGCGGAAGACCCGGGGCGGGGTTGCCGCCCGGGCATTGATGGCGGCCTCCTCTTGAATGCGTTGC
Hongyouzi        TGCAGCGGAAGACCCGGGGCGGGGTTGCCGCCCGGGCATTGATGGCGGCCTCCTCTTGAATGCGTTGC
Zijiehong        TGCAGCGGAAGACCCGGGGCGGGGTTGCCGCCCGGGCATTGATGGCGGCCTCCTCTTGAATGCGTTGC
Baihuamai        TGCAGCGGAAGACCCGGGGCGGGGTTGCCGCCCGGGCATTGATGGCGGCCTCCTCTTGAATGCGTTGC
Huixianhong      TGCAGCGGAAGACCCGGGGCGGGGTTGCCGCCCGGGCATTGATGGCGGCCTCCTCTTGAATGCGTTGC
Baimangmai       TGCAGCGGAAGACCCGGGGCGGGGTTGCCGCCCGGGCATTGATGGCGGCCTCCTCTTGAATGCGTTGC
```

	5,040 · 5,050 · 5,060 · 5,070 · 5,080 · 5,090 · 5,100
Hulutou	AGAGACGCGTCGGGGGCGACGATGATGGAAGCGACGCGCACGCCTCCCCCTCGAAGGACAGGCGGCGT
Baihulu	AGAGACGCGTCGGGGGCGACGATGATGGAAGCGACGCGCACGCCTCCCCCTCGAAGGACAGGCGGCGT
Chiyacao	AGAGACGCGTCGGGGGCGACGATGATGGAAGCGACGCGCACGCCTCCCCCTCGAAGGACAGGCGGCGT
Hongmangmai	AGAGACGCGTCGGGGGCGACGATGATGGAAGCGACGCGCACGCCTCCCCCTCGAAGGACAGGCGGCGT
Hongmai	AGAGACGCGTCGGGGGCGACGATGATGGAAGCGACGCGCACGCCTCCCCCTCGAAGGACAGGCGGCGT
Wangshuibai	AGAGACGCGTCGGGGGCGACGATGATGGAAGCGACGCGCACGCCTCCCCCTCGAAGGACAGGCGGCGT
Shi 4185	AGAGACGCGTCGGGGGCGACGATGATGGAAGCGACGCGCACGCCTCCCCCTCGAAGGACAGGCGGCGT
Mazhamai	AGAGACGCGTCGGGGGCGACGATGATGGAAGCGACGCGCACGCCTCCCCCTCGAAGGACAGGCGGCGT
Xiaofoshou	AGAGACGCGTCGGGGGCGACGATGATGGAAGCGACGCGCACGCCTCCCCCTCGAAGGACAGGCGGCGT
Aikang 58	AGAGACGCGTCGGGGGCGACGATGATGGAAGCGACGCGCACGCCTCCCCCTCGAAGGACAGGCGGCGT
AL8/78	AGAGACGCGTCGGGGGCGACGATGATGGAAGCGACGCGCACGCCTCCCCCTCGAAGGACAGGCGGCGT
PI 431602	AGAGACGCGTCGGGGGCGACGATGATGGAAGCGACGCGCACGCCTCCCCCTCGAAGGACAGGCGGCGT
PI 486274	AGAGACGCGTCGGGGGCGACGATGATGGAAGCGACGCGCACGCCTCCCCCTCGAAGGACAGGCGGCGT
RM 000182	AGAGACGCGTCGGGGGCGACGATGATGGAAGCGACGCGCACGCCTCCCCCTCGAAGGACAGGCGGCGT
PI 511381	AGAGACGCGTCGGGGGCGACGATGATGGAAGCGACGCGCACGCCTCCCCCTCGAAGGACAGGCGGCGT
PI 486271	AGAGACGCGTCGGGGGCGACGATGATGGAAGCGACGCGCACGCCTCCCCCTCGAAGGACAGGCGGCGT
PI 511367	AGAGACGCGTCGGGGGCGACGATGATGGAAGCGACGCGCACGCCTCCCCCTCGAAGGACAGGCGGCGT
Chinese Spring	AGAGACGCGTCGGGGGCGACGATGATGGAAGCGACGCGCACGCCTCCCCCTCGAAGGACAGGCGGCGT
Hanzhongbai	AGAGACGCGTCGGGGGCGACGATGATGGAAGCGACGCGCACGCCTCCCCCTCGAAGGACAGGCGGCGT
Hongtoumai	AGAGACGCGTCGGGGGCGACGATGATGGAAGCGACGCGCACGCCTCCCCCTCGAAGGACAGGCGGCGT
Hongyouzi	AGAGACGCGTCGGGGGCGACGATGATGGAAGCGACGCGCACGCCTCCCCCTCGAAGGACAGGCGGCGT
Zijiehong	AGAGACGCGTCGGGGGCGACGATGATGGAAGCGACGCGCACGCCTCCCCCTCGAAGGACAGGCGGCGT
Baihuamai	AGAGACGCGTCGGGGGCGACGATGATGGAAGCGACGCGCACGCCTCCCCCTCGAAGGACAGGCGGCGT
Huixianhong	AGAGACGCGTCGGGGGCGACGATGATGGAAGCGACGCGCACGCCTCCCCCTCGAAGGACAGGCGGCGT
Baimangmai	AGAGACGCGTCGGGGGCGACGATGATGGAAGCGACGCGCACGCCTCCCCCTCGAAGGACAGGCGGCGT

	5,110 · 5,120 · 5,130 · 5,140 · 5,150 · 5,160
Hulutou	CTCCAGAACCTCGTCAGCAAGCTCAGCTGCCTGGATCCGGAGACGTGCAGCGGCCGATCCAGACCGGG
Baihulu	CTCCAGAACCTCGTCAGCAAGCTCAGCTGCCTGGATCCGGAGACGTGCAGCGGCCGATCCAGACCGGG
Chiyacao	CTCCAGAACCTCGTCAGCAAGCTCAGCTGCCTGGATCCGGAGACGTGCAGCGGCCGATCCAGACCGGG
Hongmangmai	CTCCAGAACCTCGTCAGCAAGCTCAGCTGCCTGGATCCGGAGACGTGCAGCGGCCGATCCAGACCGGG
Hongmai	CTCCAGAACCTCGTCAGCAAGCTCAGCTGCCTGGATCCGGAGACGTGCAGCGGCCGATCCAGACCGGG
Wangshuibai	CTCCAGAACCTCGTCAGCAAGCTCAGCTGCCTGGATCCGGAGACGTGCAGCGGCCGATCCAGACCGGG
Shi 4185	CTCCAGAACCTCGTCAGCAAGCTCAGCTGCCTGGATCCGGAGACGTGCAGCGGCCGATCCAGACCGGG
Mazhamai	CTCCAGAACCTCGTCAGCAAGCTCAGCTGCCTGGATCCGGAGACGTGCAGCGGCCGATCCAGACCGGG
Xiaofoshou	CTCCAGAACCTCGTCAGCAAGCTCAGCTGCCTGGATCCGGAGACGTGCAGCGGCCGATCCAGACCGGG
Aikang 58	CTCCAGAACCTCGTCAGCAAGCTCAGCTGCCTGGATCCGGAGACGTGCAGCGGCCGATCCAGACCGGG
AL8/78	CTCCAGAACCTCGTCAG**T**AAG**T**TCAGCTGCCTGGATCCGGAGACGTGCAGCGGCCGATCCAGACCGGG
PI 431602	CTCCAGAACCTCGTCAG**T**AAG**T**TCAGCTGCCTGGATCCGGAGACGTGCAGCGGCCGATCCAGACCGGG
PI 486274	CTCCAGAACCTCGTCAG**T**AAG**T**TCAGCTGCCTGGATCCGGAGACGTGCAGCGGCCGATCCAGACCGGG
RM 000182	CTCCAGAACCTCGTCAGCAAGCTCAGCTGCCTGGATCCGGAGACGTGCAGCGGCCGATCCAGACCGGG
PI 511381	CTCCAGAACCTCGTCAGCAAGCTCAGCTGCCTGGATCCGGAGACGTGCAGCGGCCGATCCAGACCGGG
PI 486271	CTCCAGAACCTCGTCAGCAAGCTCAGCTGCCTGGATCCGGAGACGTGCAGCGGCCGATCCAGACCGGG
PI 511367	CTCCAGAACCTCGTCAGCAAGCTCAGCTGCCTGGATCCGGAGACGTGCAGCGGCCGATCCAGACCGGG
Chinese Spring	CTCCAGAACCTCGTCAGCAAGCTCAGCTGCCTGGATCCGGAGACGTGCAGCGGCCGATCCAGACCGGG
Hanzhongbai	CTCCAGAACCTCGTCAGCAAGCTCAGCTGCCTGGATCCGGAGACGTGCAGCGGCCGATCCAGACCGGG
Hongtoumai	CTCCAGAACCTCGTCAGCAAGCTCAGCTGCCTGGATCCGGAGACGTGCAGCGGCCGATCCAGACCGGG
Hongyouzi	CTCCAGAACCTCGTCAGCAAGCTCAGCTGCCTGGATCCGGAGACGTGCAGCGGCCGATCCAGACCGGG
Zijiehong	CTCCAGAACCTCGTCAGCAAGCTCAGCTGCCTGGATCCGGAGACGTGCAGCGGCCGATCCAGACCGGG
Baihuamai	CTCCAGAACCTCGTCAGCAAGCTCAGCTGCCTGGATCCGGAGACGTGCAGCGGCCGATCCAGACCGGG
Huixianhong	CTCCAGAACCTCGTCAGCAAGCTCAGCTGCCTGGATCCGGAGACGTGCAGCGGCCGATCCAGACCGGG
Baimangmai	CTCCAGAACCTCGTCAGCAAGCTCAGCTGCCTGGATCCGGAGACGTGCAGCGGCCGATCCAGACCGGG

Accession	5,170 – 5,230	5,240 – 5,300
Hulutou	CACCAGTGGCACGACAGTGGACACAGGGATAACAACCGGGGAAAAAAAATTCTCCGGCAAGCGGGGCG	GCGGCGGTGGCAGCTCATGTTGTCCGGCGAGCCCCGAATGGCACGCGTCGGGAGGGGCT----GGTGG
Baihulu	CACCAGTGGCACGACAGTGGACACAGGGATAACAACCGGGGAAAAAAAATTCTCCGGCAAGCGGGGCG	GCGGCGGTGGCAGCTCATGTTGTCCGGCGAGCCCCGAATGGCACGCGTCGGGAGGGGCT----GGTGG
Chiyacao	CACCAGTGGCACGACAGTGGACACAGGGATAACAACCGGGGAAAAAAAATTCTCCGGCAAGCGGGGCG	GCGGCGGTGGCAGCTCATGTTGTCCGGCGAGCCCCGAATGGCACGCGTCGGGAGGGGCT----GGTGG
Hongmangmai	CACCAGTGGCACGACAGTGGACACAGGGATAACAACCGGGGAAAAAAAATTCTCCGGCAAGCGGGGCG	GCGGCGGTGGCAGCTCATGTTGTCCGGCGAGCCCCGAATGGCACGCGTCGGGAGGGGCT----GGTGG
Hongmai	CACCAGTGGCACGACAGTGGACACAGGGATAACAACCGGGGAAAAAAAATTCTCCGGCAAGCGGGGCG	GCGGCGGTGGCAGCTCATGTTGTCCGGCGAGCCCCGAATGGCACGCGTCGGGAGGGGCT----GGTGG
Wangshuibai	CACCAGTGGCACGACAGTGGACACAGGGATAACAACCGGGGAAAAAAAATTCTCCGGCAAGCGGGGCG	GCGGCGGTGGCAGCTCATGTTGTCCGGCGAGCCCCGAATGGCACGCGTCGGGAGGGGCT----GGTGG
Shi 4185	CACCAGTGGCACGACAGTGGACACAGGGATAACAACCGGGGAAAAAAAATTCTCCGGCAAGCGGGGCG	GCGGCGGTGGCAGCTCATGTTGTCCGGCGAGCCCCGAATGGCACGCGTCGGGAGGGGCT----GGTGG
Mazhamai	CACCAGTGGCACGACAGTGGACACAGGGATAACAACCGGGGAAAAAAAATTCTCCGGCAAGCGGGGCG	GCGGCGGTGGCAGCTCATGTTGTCCGGCGAGCCCCGAATGGCACGCGTCGGGAGGGGCT----GGTGG
Xiaofoshou	CACCAGTGGCACGACAGTGGACACAGGGATAACAACCGGGGAAAAAAAATTCTCCGGCAAGCGGGGCG	GCGGCGGTGGCAGCTCATGTTGTCCGGCGAGCCCCGAATGGCACGCGTCGGGAGGGGCT----GGTGG
Aikang 58	CACCAGTGGCACGACAGTGGACACAGGGATAACAACCGGGGAAAAAAAATTCTCCGGCAAGCGGGGCG	GCGGCGGTGGCAGCTCATGTTGTCCGGCGAGCCCCGAATGGCACGCGTCGGGAGGGGCT----GGTGG
AL8/78	CACCAGTGGCACGACAGTGGACACAGGGATAACAACCGGGGAAAAAAAAATCTCCGGCAAGCGGGGCG	GCGGCGGTGGCAGCTCATGTTGTCCGGCGAGCCCCGAATGGCACGCGCCGGGAGGGGCTGGTGGGTGG
PI 431602	CACCAGTGGCACGACAGTGGACACAGGGATAACAACCGGGGAAAAAAAAATCTCCGGCAAGCGGGGCG	GCGGCGGTGGCAGCTCATGTTGTCCGGCGAGCCCCGAATGGCACGCGCCGGGAGGGGCTGGTGGGTGG
PI 486274	CACCAGTGGCACGACAGTGGACACAGGGATAACAACCGGGGAAAAAAAAATCTCCGGCAAGCGGGGCG	GCGGCGGTGGCAGCTCATGTTGTCCGGCGAGCCCCGAATGGCACGCGCCGGGAGGGGCTGGTGGGTGG
RM 000182	CACCAGTGGCACGACAGTGGACACAGGGATAACAACCGGG-AAAAAAAAATCTCCGGCAAGCGGGGCG	GCGGCGGTGGCAGCTCATGTTGTCCGGCGAGCCCCGAATGGCACGCGCCGGGAGGGGCTGGTGGGTGG
PI 511381	CACCAGTGGCACGACAGTGGACACAGGGATAACAACCGGG-AAAAAAAAATCTCCGGCAAGCGGGGCG	GCGGCGGTGGCAGCTCATGTTGTCCGGCGAGCCCCGAATGGCACGCGCCGGGAGGGGCTGGTGGGTGG
PI 486271	CACCAGTGGCACGACAGTGGACACAGGGATAACAACCGGG-AAAAAAAAATCTCCGGCAAGCGGGGCG	GCGGCGGTGGCAGCTCATGTTGTCCGGCGAGCCCCGAATGGCACGCGCCGGGAGGGGCTGGTGGGTGG
PI 511367	CACCAGTGGCACGACAGTGGACACAGGGATAACAACCGGG-AAAAAAAAATCTCCGGCAAGCGGGGCG	GCGGCGGTGGCAGCTCATGTTGTCCGGCGAGCCCCGAATGGCACGCGCCGGGAGGGGCTGGTGGGTGG
Chinese Spring	CACCAGTGGCACGACAGTGGACACAGGGATAACAACCGGGAAAAAAAAAATCTCCGGCAAGCGGGGCG	GCGGCGGTGGCAGCTCATGTTGTCCGGCGAGCCCCGAATGGCACGCGCCGGGTGGGGCTGGTGGGTGG
Hanzhongbai	CACCAGTGGCACGACAGTGGACACAGGGATAACAACCGGGAAAAAAAAAATCTCCGGCAAGCGGGGCG	GCGGCGGTGGCAGCTCATGTTGTCCGGCGAGCCCCGAATGGCACGCGCCGGGTGGGGCTGGTGGGTGG
Hongtoumai	CACCAGTGGCACGACAGTGGACACAGGGATAACAACCGGGAAAAAAAAAATCTCCGGCAAGCGGGGCG	GCGGCGGTGGCAGCTCATGTTGTCCGGCGAGCCCCGAATGGCACGCGCCGGGTGGGGCTGGTGGGTGG
Hongyouzi	CACCAGTGGCACGACAGTGGACACAGGGATAACAACCGGGAAAAAAAAAATCTCCGGCAAGCGGGGCG	GCGGCGGTGGCAGCTCATGTTGTCCGGCGAGCCCCGAATGGCACGCGCCGGGTGGGGCTGGTGGGTGG
Zijiehong	CACCAGTGGCACGACAGTGGACACAGGGATAACAACCGGGAAAAAAAAAATCTCCGGCAAGCGGGGCG	GCGGCGGTGGCAGCTCATGTTGTCCGGCGAGCCCCGAATGGCACGCGCCGGGTGGGGCTGGTGGGTGG
Baihuamai	CACCAGTGGCACGACAGTGGACACAGGGATAACAACCGGGAAAAAAAAAATCTCCGGCAAGCGGGGCG	GCGGCGGTGGCAGCTCATGTTGTCCGGCGAGCCCCGAATGGCACGCGCCGGGTGGGGCTGGTGGGTGG
Huixianhong	CACCAGTGGCACGACAGTGGACACAGGGATAACAACCGGGAAAAAAAAAATCTCCGGCAAGCGGGGCG	GCGGCGGTGGCAGCTCATGTTGTCCGGCGAGCCCCGAATGGCACGCGCCGGGTGGGGCTGGTGGGTGG
Baimangmai	CACCAGTGGCACGACAGTGGACACAGGGATAACAACCGGGAAAAAAAAAATCTCCGGCAAGCGGGGCG	GCGGCGGTGGCAGCTCATGTTGTCCGGCGAGCCCCGAATGGCACGCGCCGGGTGGGGCTGGTGGGTGG

```
                5,310     5,320     5,330     5,340     5,350     5,360     5,370
Hulutou         GTGGGTCGCCCACTCATGTTGTCCGGTGATTTTTCCAGCTGTGCCAGCTAAGTTTTTCA-----AGTG
Baihulu         GTGGGTCGCCCACTCATGTTGTCCGGTGATTTTTCCAGCTGTGCCAGCTAAGTTTTTCA-----AGTG
Chiyacao        GTGGGTCGCCCACTCATGTTGTCCGGTGATTTTTCCAGCTGTGCCAGCTAAGTTTTTCA-----AGTG
Hongmangmai     GTGGGTCGCCCACTCATGTTGTCCGGTGATTTTTCCAGCTGTGCCAGCTAAGTTTTTCA-----AGTG
Hongmai         GTGGGTCGCCCACTCATGTTGTCCGGTGATTTTTCCAGCTGTGCCAGCTAAGTTTTTCA-----AGTG
Wangshuibai     GTGGGTCGCCCACTCATGTTGTCCGGTGATTTTTCCAGCTGTGCCAGCTAAGTTTTTCA-----AGTG
Shi 4185        GTGGGTCGCCCACTCATGTTGTCCGGTGATTTTTCCAGCTGTGCCAGCTAAGTTTTTCA-----AGTG
Mazhamai        GTGGGTCGCCCACTCATGTTGTCCGGTGATTTTTCCAGCTGTGCCAGCTAAGTTTTTCA-----AGTG
Xiaofoshou      GTGGGTCGCCCACTCATGTTGTCCGGTGATTTTTCCAGCTGTGCCAGCTAAGTTTTTCA-----AGTG
Aikang 58       GTGGGTCGCCCACTCATGTTGTCCGGTGATTTTTCCAGCTGTGCCAGCTAAGTTTTTCA-----AGTG
AL8/78          GTGGGTCGCCCACTCATGTTGTCCGGTGATTTTTCCAGCTGTGCCAGCTAAGTTTTTCA-----AGTG
PI 431602       GTGGGTCGCCCACTCATGTTGTCCGGTGATTTTTCCAGCTGTGCCAGCTAAGTTTTTCA-----AGTG
PI 486274       GTGGGTCGCCCACTCATGTTGTCCGGTGATTTTTCCAGCTGTGCCAGCTAAGTTTTTCA-----AGTG
RM 000182       GTGGGTCGCCCACTCATCTTGTCCGGTGATTTTTCCAGCTGTGCCAGCTAAGTTTTTCAAGTGCAGTG
PI 511381       GTGGGTCGCCCACTCATCTTGTCCGGTGATTTTTCCAGCTGTGCCAGCTAAGTTTTTCAAGTGCAGTG
PI 486271       GTGGGTCGCCCACTCATCTTGTCCGGTGATTTTTCCAGCTGTGCCAGCTAAGTTTTTCAAGTGCAGTG
PI 511367       GTGGGTCGCCCACTCATCTTGTCCGGTGATTTTTCCAGCTGTGCCAGCTAAGTTTTTCAAGTGCAGTG
Chinese Spring  GTGGGTCGCCCACTCATCTTGTCCGGTGATTTTTCCAGCTGTGCCAGCTAAGTTTTTCAAGTGCAGTG
Hanzhongbai     GTGGGTCGCCCACTCATCTTGTCCGGTGATTTTTCCAGCTGTGCCAGCTAAGTTTTTCAAGTGCAGTG
Hongtoumai      GTGGGTCGCCCACTCATCTTGTCCGGTGATTTTTCCAGCTGTGCCAGCTAAGTTTTTCAAGTGCAGTG
Hongyouzi       GTGGGTCGCCCACTCATCTTGTCCGGTGATTTTTCCAGCTGTGCCAGCTAAGTTTTTCAAGTGCAGTG
Zijiehong       GTGGGTCGCCCACTCATCTTGTCCGGTGATTTTTCCAGCTGTGCCAGCTAAGTTTTTCAAGTGCAGTG
Baihuamai       GTGGGTCGCCCACTCATCTTGTCCGGTGATTTTTCCAGCTGTGCCAGCTAAGTTTTTCAAGTGCAGTG
Huixianhong     GTGGGTCGCCCACTCATCTTGTCCGGTGATTTTTCCAGCTGTGCCAGCTAAGTTTTTCAAGTGCAGTG
Baimangmai      GTGGGTCGCCCACTCATCTTGTCCGGTGATTTTTCCAGCTGTGCCAGCTAAGTTTTTCAAGTGCAGTG

                   5,380     5,390     5,400     5,410     5,420     5,430     5,440
Hulutou         TTTGCGATTTAGGAATTTTGAGAGGATACATGCTTCGCTGTTTGATCTATTTCTATCTATCTCATGTA
Baihulu         TTTGCGATTTAGGAATTTTGAGAGGATACATGCTTCGCTGTTTGATCTATTTCTATCTATCTCATGTA
Chiyacao        TTTGCGATTTAGGAATTTTGAGAGGATACATGCTTCGCTGTTTGATCTATTTCTATCTATCTCATGTA
Hongmangmai     TTTGCGATTTAGGAATTTTGAGAGGATACATGCTTCGCTGTTTGATCTATTTCTATCTATCTCATGTA
Hongmai         TTTGCGATTTAGGAATTTTGAGAGGATACATGCTTCGCTGTTTGATCTATTTCTATCTATCTCATGTA
Wangshuibai     TTTGCGATTTAGGAATTTTGAGAGGATACATGCTTCGCTGTTTGATCTATTTCTATCTATCTCATGTA
Shi 4185        TTTGCGATTTAGGAATTTTGAGAGGATACATGCTTCGCTGTTTGATCTATTTCTATCTATCTCATGTA
Mazhamai        TTTGCGATTTAGGAATTTTGAGAGGATACATGCTTCGCTGTTTGATCTATTTCTATCTATCTCATGTA
Xiaofoshou      TTTGCGATTTAGGAATTTTGAGAGGATACATGCTTCGCTGTTTGATCTATTTCTATCTATCTCATGTA
Aikang 58       TTTGCGATTTAGGAATTTTGAGAGGATACATGCTTCGCTGTTTGATCTATTTCTATCTATCTCATGTA
AL8/78          TTTGCGATTTAGGAATTTTGAGAGGATACATGCTTCGCTGTTTGATCTATTTCTATCTATCTCATGTA
PI 431602       TTTGCGATTTAGGAATTTTGAGAGGATACATGCTTCGCTGTTTGATCTATTTCTATCTATCTCATGTA
PI 486274       TTTGCGATTTAGGAATTTTGAGAGGATACATGCTTCGCTGTTTGATCTATTTCTATCTATCTCATGTA
RM 000182       TTTGCGATTTAGGAATTTTGAGAGGATACATGCTTCGCTGTTTTATCTATTTCTATCTATCTCATGTA
PI 511381       TTTGCGATTTAGGAATTTTGAGAGGATACATGCTTCGCTGTTTTATCTATTTCTATCTATCTCATGTA
PI 486271       TTTGCGATTTAGGAATTTTGAGAGGATACATGCTTCGCTGTTTTATCTATTTCTATCTATCTCATGTA
PI 511367       TTTGCGATTTAGGAATTTTGAGAGGATACATGCTTCGCTGTTTTATCTATTTCTATCTATCTCATGTA
Chinese Spring  TTTGCGATTTAGGAATTTTGAGAGGATACATGCTTCGCTGTTTTATCTATTTCTATCTATCTCATGTA
Hanzhongbai     TTTGCGATTTAGGAATTTTGAGAGGATACATGCTTCGCTGTTTTATCTATTTCTATCTATCTCATGTA
Hongtoumai      TTTGCGATTTAGGAATTTTGAGAGGATACATGCTTCGCTGTTTTATCTATTTCTATCTATCTCATGTA
Hongyouzi       TTTGCGATTTAGGAATTTTGAGAGGATACATGCTTCGCTGTTTTATCTATTTCTATCTATCTCATGTA
Zijiehong       TTTGCGATTTAGGAATTTTGAGAGGATACATGCTTCGCTGTTTTATCTATTTCTATCTATCTCATGTA
Baihuamai       TTTGCGATTTAGGAATTTTGAGAGGATACATGCTTCGCTGTTTTATCTATTTCTATCTATCTCATGTA
Huixianhong     TTTGCGATTTAGGAATTTTGAGAGGATACATGCTTCGCTGTTTTATCTATTTCTATCTATCTCATGTA
Baimangmai      TTTGCGATTTAGGAATTTTGAGAGGATACATGCTTCGCTGTTTTATCTATTTCTATCTATCTCATGTA
```

	5,450 · 5,460 · 5,470 · 5,480 · 5,490 · 5,500
Hulutou	AGCCTTTCGAGATGACAAGCAGATCAACTTCATGACATTATCAATACTTCTGCAGGTGACTATGGTGA
Baihulu	AGCCTTTCGAGATGACAAGCAGATCAACTTCATGACATTATCAATACTTCTGCAGGTGACTATGGTGA
Chiyacao	AGCCTTTCGAGATGACAAGCAGATCAACTTCATGACATTATCAATACTTCTGCAGGTGACTATGGTGA
Hongmangmai	AGCCTTTCGAGATGACAAGCAGATCAACTTCATGACATTATCAATACTTCTGCAGGTGACTATGGTGA
Hongmai	AGCCTTTCGAGATGACAAGCAGATCAACTTCATGACATTATCAATACTTCTGCAGGTGACTATGGTGA
Wangshuibai	AGCCTTTCGAGATGACAAGCAGATCAACTTCATGACATTATCAATACTTCTGCAGGTGACTATGGTGA
Shi 4185	AGCCTTTCGAGATGACAAGCAGATCAACTTCATGACATTATCAATACTTCTGCAGGTGACTATGGTGA
Mazhamai	AGCCTTTCGAGATGACAAGCAGATCAACTTCATGACATTATCAATACTTCTGCAGGTGACTATGGTGA
Xiaofoshou	AGCCTTTCGAGATGACAAGCAGATCAACTTCATGACATTATCAATACTTCTGCAGGTGACTATGGTGA
Aikang 58	AGCCTTTCGAGATGACAAGCAGATCAACTTCATGACATTATCAATACTTCTGCAGGTGACTATGGTGA
AL8/78	AGCCTTTCGAGATGACAAGCAGATCAACTTCATGACATTATCAATACTTCTGCAGGTGACTATGGTGA
PI 431602	AGCCTTTCGAGATGACAAGCAGATCAACTTCATGACATTATCAATACTTCTGCAGGTGACTATGGTGA
PI 486274	AGCCTTTCGAGATGACAAGCAGATCAACTTCATGACATTATCAATACTTCTGCAGGTGACTATGGTGA
RM 000182	AGCCTTTCG**G**GATGACAAGCAGATCAACTTCATGACATTATCAATACTTCTGCAGGTGACTATGGTGA
PI 511381	AGCCTTTCG**G**GATGACAAGCAGATCAACTTCATGACATTATCAATACTTCTGCAGGTGACTATGGTGA
PI 486271	AGCCTTTCG**G**GATGACAAGCAGATCAACTTCATGACATTATCAATACTTCTGCAGGTGACTATGGTGA
PI 511367	AGCCTTTCG**G**GATGACAAGCAGATCAACTTCATGACATTATCAATACTTCTGCAGGTGACTATGGTGA
Chinese Spring	AGCCTTTCG**G**GATGACAAGCAGATCAACTTCATGACATTATCAATACTTCTGCAGGTGACTATGGTGA
Hanzhongbai	AGCCTTTCG**G**GATGACAAGCAGATCAACTTCATGACATTATCAATACTTCTGCAGGTGACTATGGTGA
Hongtoumai	AGCCTTTCG**G**GATGACAAGCAGATCAACTTCATGACATTATCAATACTTCTGCAGGTGACTATGGTGA
Hongyouzi	AGCCTTTCG**G**GATGACAAGCAGATCAACTTCATGACATTATCAATACTTCTGCAGGTGACTATGGTGA
Zijiehong	AGCCTTTCG**G**GATGACAAGCAGATCAACTTCATGACATTATCAATACTTCTGCAGGTGACTATGGTGA
Baihuamai	AGCCTTTCG**G**GATGACAAGCAGATCAACTTCATGACATTATCAATACTTCTGCAGGTGACTATGGTGA
Huixianhong	AGCCTTTCG**G**GATGACAAGCAGATCAACTTCATGACATTATCAATACTTCTGCAGGTGACTATGGTGA
Baimangmai	AGCCTTTCG**G**GATGACAAGCAGATCAACTTCATGACATTATCAATACTTCTGCAGGTGACTATGGTGA

	5,510 · 5,520 · 5,530 · 5,540 · 5,550 · 5,560 · 5,570
Hulutou	ACTTGACTGGAACCAGCGTTATCAAATTCTAAAAGGAATTTGTCAAGGTTTGCATCATCTCCATGACG
Baihulu	ACTTGACTGGAACCAGCGTTATCAAATTCTAAAAGGAATTTGTCAAGGTTTGCATCATCTCCATGACG
Chiyacao	ACTTGACTGGAACCAGCGTTATCAAATTCTAAAAGGAATTTGTCAAGGTTTGCATCATCTCCATGACG
Hongmangmai	ACTTGACTGGAACCAGCGTTATCAAATTCTAAAAGGAATTTGTCAAGGTTTGCATCATCTCCATGACG
Hongmai	ACTTGACTGGAACCAGCGTTATCAAATTCTAAAAGGAATTTGTCAAGGTTTGCATCATCTCCATGACG
Wangshuibai	ACTTGACTGGAACCAGCGTTATCAAATTCTAAAAGGAATTTGTCAAGGTTTGCATCATCTCCATGACG
Shi 4185	ACTTGACTGGAACCAGCGTTATCAAATTCTAAAAGGAATTTGTCAAGGTTTGCATCATCTCCATGACG
Mazhamai	ACTTGACTGGAACCAGCGTTATCAAATTCTAAAAGGAATTTGTCAAGGTTTGCATCATCTCCATGACG
Xiaofoshou	ACTTGACTGGAACCAGCGTTATCAAATTCTAAAAGGAATTTGTCAAGGTTTGCATCATCTCCATGACG
Aikang 58	ACTTGACTGGAACCAGCGTTATCAAATTCTAAAAGGAATTTGTCAAGGTTTGCATCATCTCCATGACG
AL8/78	ACTTGACTGGAACCAGCGTTATCAAATTCTAAAAGGAATTTGTCAAGGTTTGCATCATCTCCATGACG
PI 431602	ACTTGACTGGAACCAGCGTTATCAAATTCTAAAAGGAATTTGTCAAGGTTTGCATCATCTCCATGACG
PI 486274	ACTTGACTGGAACCAGCGTTATCAAATTCTAAAAGGAATTTGTCAAGGTTTGCATCATCTCCATGACG
RM 000182	ACTTGACTGGAACCAGCGTTATCAAATTCTAAAAGGAATTTGTCA**G**GGTTTGCATCATCTCCATGACG
PI 511381	ACTTGACTGGAACCAGCGTTATCAAATTCTAAAAGGAATTTGTCA**G**GGTTTGCATCATCTCCATGACG
PI 486271	ACTTGACTGGAACCAGCGTTATCAAATTCTAAAAGGAATTTGTCA**G**GGTTTGCATCATCTCCATGACG
PI 511367	ACTTGACTGGAACCAGCGTTATCAAATTCTAAAAGGAATTTGTCA**G**GGTTTGCATCATCTCCATGACG
Chinese Spring	ACTTGACTGGAACCAGCGTTATCAAATTCTAAAAGGAATTTGTCA**G**GGTTTGCATCATCTCCATGACG
Hanzhongbai	ACTTGACTGGAACCAGCGTTATCAAATTCTAAAAGGAATTTGTCA**G**GGTTTGCATCATCTCCATGACG
Hongtoumai	ACTTGACTGGAACCAGCGTTATCAAATTCTAAAAGGAATTTGTCA**G**GGTTTGCATCATCTCCATGACG
Hongyouzi	ACTTGACTGGAACCAGCGTTATCAAATTCTAAAAGGAATTTGTCA**G**GGTTTGCATCATCTCCATGACG
Zijiehong	ACTTGACTGGAACCAGCGTTATCAAATTCTAAAAGGAATTTGTCA**G**GGTTTGCATCATCTCCATGACG
Baihuamai	ACTTGACTGGAACCAGCGTTATCAAATTCTAAAAGGAATTTGTCA**G**GGTTTGCATCATCTCCATGACG
Huixianhong	ACTTGACTGGAACCAGCGTTATCAAATTCTAAAAGGAATTTGTCA**G**GGTTTGCATCATCTCCATGACG
Baimangmai	ACTTGACTGGAACCAGCGTTATCAAATTCTAAAAGGAATTTGTCA**G**GGTTTGCATCATCTCCATGACG

```
                   5,580     5,590     5,600     5,610     5,620     5,630     5,640
Hulutou            AAATGCACGTTTTTCATGGAGATATCAAACCAGCCAATATATTAATAGGGGATAACCTTGTGCCTAAA
Baihulu            AAATGCACGTTTTTCATGGAGATATCAAACCAGCCAATATATTAATAGGGGATAACCTTGTGCCTAAA
Chiyacao           AAATGCACGTTTTTCATGGAGATATCAAACCAGCCAATATATTAATAGGGGATAACCTTGTGCCTAAA
Hongmangmai        AAATGCACGTTTTTCATGGAGATATCAAACCAGCCAATATATTAATAGGGGATAACCTTGTGCCTAAA
Hongmai            AAATGCACGTTTTTCATGGAGATATCAAACCAGCCAATATATTAATAGGGGATAACCTTGTGCCTAAA
Wangshuibai        AAATGCACGTTTTTCATGGAGATATCAAACCAGCCAATATATTAATAGGGGATAACCTTGTGCCTAAA
Shi 4185           AAATGCACGTTTTTCATGGAGATATCAAACCAGCCAATATATTAATAGGGGATAACCTTGTGCCTAAA
Mazhamai           AAATGCACGTTTTTCATGGAGATATCAAACCAGCCAATATATTAATAGGGGATAACCTTGTGCCTAAA
Xiaofoshou         AAATGCACGTTTTTCATGGAGATATCAAACCAGCCAATATATTAATAGGGGATAACCTTGTGCCTAAA
Aikang 58          AAATGCACGTTTTTCATGGAGATATCAAACCAGCCAATATATTAATAGGGGATAACCTTGTGCCTAAA
AL8/78             AAATGCACGTTTTTCATGGAGATATCAAACCAGCCAATATATTAATAGGGGATAACCTTGTGCCTAAA
PI 431602          AAATGCACGTTTTTCATGGAGATATCAAACCAGCCAATATATTAATAGGGGATAACCTTGTGCCTAAA
PI 486274          AAATGCACGTTTTTCATGGAGATATCAAACCAGCCAATATATTAATAGGGGATAACCTTGTGCCTAAA
RM 000182          AAATGCACGTTTTTCATGGAGATATCAAACCAGCCAATATATTAATAGGGGATAACCTTGTGCCTAAA
PI 511381          AAATGCACGTTTTTCATGGAGATATCAAACCAGCCAATATATTAATAGGGGATAACCTTGTGCCTAAA
PI 486271          AAATGCACGTTTTTCATGGAGATATCAAACCAGCCAATATATTAATAGGGGATAACCTTGTGCCTAAA
PI 511367          AAATGCACGTTTTTCATGGAGATATCAAACCAGCCAATATATTAATAGGGGATAACCTTGTGCCTAAA
Chinese Spring     AAATGCACGTTTTTCATGGAGATATCAAACCAGCCAATATATTAATAGGGGATAACCTTGTGCCTAAA
Hanzhongbai        AAATGCACGTTTTTCATGGAGATATCAAACCAGCCAATATATTAATAGGGGATAACCTTGTGCCTAAA
Hongtoumai         AAATGCACGTTTTTCATGGAGATATCAAACCAGCCAATATATTAATAGGGGATAACCTTGTGCCTAAA
Hongyouzi          AAATGCACGTTTTTCATGGAGATATCAAACCAGCCAATATATTAATAGGGGATAACCTTGTGCCTAAA
Zijiehong          AAATGCACGTTTTTCATGGAGATATCAAACCAGCCAATATATTAATAGGGGATAACCTTGTGCCTAAA
Baihuamai          AAATGCACGTTTTTCATGGAGATATCAAACCAGCCAATATATTAATAGGGGATAACCTTGTGCCTAAA
Huixianhong        AAATGCACGTTTTTCATGGAGATATCAAACCAGCCAATATATTAATAGGGGATAACCTTGTGCCTAAA
Baimangmai         AAATGCACGTTTTTCATGGAGATATCAAACCAGCCAATATATTAATAGGGGATAACCTTGTGCCTAAA

                     5,650     5,660     5,670     5,680     5,690     5,700     5,710
Hulutou            ATCTATGACTTCGGTCTCTCCCAG--ATGTTTGAAGAAGAAGAAACGGAACGTATTGTTGAAAATATC
Baihulu            ATCTATGACTTCGGTCTCTCCCAG--ATGTTTGAAGAAGAAGAAACGGAACGTATTGTTGAAAATATC
Chiyacao           ATCTATGACTTCGGTCTCTCCCAG--ATGTTTGAAGAAGAAGAAACGGAACGTATTGTTGAAAATATC
Hongmangmai        ATCTATGACTTCGGTCTCTCCCAG--ATGTTTGAAGAAGAAGAAACGGAACGTATTGTTGAAAATATC
Hongmai            ATCTATGACTTCGGTCTCTCCCAG--ATGTTTGAAGAAGAAGAAACGGAACGTATTGTTGAAAATATC
Wangshuibai        ATCTATGACTTCGGTCTCTCCCAG--ATGTTTGAAGAAGAAGAAACGGAACGTATTGTTGAAAATATC
Shi 4185           ATCTATGACTTCGGTCTCTCCCAG--ATGTTTGAAGAAGAAGAAACGGAACGTATTGTTGAAAATATC
Mazhamai           ATCTATGACTTCGGTCTCTCCCAG--ATGTTTGAAGAAGAAGAAACGGAACGTATTGTTGAAAATATC
Xiaofoshou         ATCTATGACTTCGGTCTCTCCCAG--ATGTTTGAAGAAGAAGAAACGGAACGTATTGTTGAAAATATC
Aikang 58          ATCTATGACTTCGGTCTCTCCCAG--ATGTTTGAAGAAGAAGAAACGGAACGTATTGTTGAAAATATC
AL8/78             ATCTATGACTTCGGTCTCTCCCAGATATGTTTGAAGAAGAAGAAACGGAACGTATTGTTGAAAATATC
PI 431602          ATCTATGACTTCGGTCTCTCCCAGATATGTTTGAAGAAGAAGAAACGGAACGTATTGTTGAAAATATC
PI 486274          ATCTATGACTTCGGTCTCTCCCAGATATGTTTGAAGAAGAAGAAACGGAACGTATTGTTGAAAATATC
RM 000182          ATCTATGACTTCGGTCTCTCCCAG--ATGTTTGAAGAAGAAGAAACGGAACGTATTGTTAAAAATATG
PI 511381          ATCTATGACTTCGGTCTCTCCCAG--ATGTTTGAAGAAGAAGAAACGGAACGTATTGTTAAAAATATG
PI 486271          ATCTATGACTTCGGTCTCTCCCAG--ATGTTTGAAGAAGAAGAAACGGAACGTATTGTTAAAAATATG
PI 511367          ATCTATGACTTCGGTCTCTCCCAG--ATGTTTGAAGAAGAAGAAACGGAACGTATTGTTAAAAATATG
Chinese Spring     ATCTATGACTTCGGTCTCTCCCAG--ATGTTTGAAGAAGAAGAAACGGAACGTATTGTTAAAAATATG
Hanzhongbai        ATCTATGACTTCGGTCTCTCCCAG--ATGTTTGAAGAAGAAGAAACGGAACGTATTGTTAAAAATATG
Hongtoumai         ATCTATGACTTCGGTCTCTCCCAG--ATGTTTGAAGAAGAAGAAACGGAACGTATTGTTAAAAATATG
Hongyouzi          ATCTATGACTTCGGTCTCTCCCAG--ATGTTTGAAGAAGAAGAAACGGAACGTATTGTTAAAAATATG
Zijiehong          ATCTATGACTTCGGTCTCTCCCAG--ATGTTTGAAGAAGAAGAAACGGAACGTATTGTTAAAAATATG
Baihuamai          ATCTATGACTTCGGTCTCTCCCAG--ATGTTTGAAGAAGAAGAAACGGAACGTATTGTTAAAAATATG
Huixianhong        ATCTATGACTTCGGTCTCTCCCAG--ATGTTTGAAGAAGAAGAAACGGAACGTATTGTTAAAAATATG
Baimangmai         ATCTATGACTTCGGTCTCTCCCAG--ATGTTTGAAGAAGAAGAAACGGAACGTATTGTTAAAAATATG
```

```
                          5,720         5,730         5,740         5,750         5,760         5,770         5,780
Hulutou          GCCGGAACATTGTAAGCTAACCCGTTCCTCTTTGTTATTTTGTTTTGTTTCTGCTGACACAGGTACTT
Baihulu          GCCGGAACATTGTAAGCTAACCCGTTCCTCTTTGTTATTTTGTTTTGTTTCTGCTGACACAGGTACTT
Chiyacao         GCCGGAACATTGTAAGCTAACCCGTTCCTCTTTGTTATTTTGTTTTGTTTCTGCTGACACAGGTACTT
Hongmangmai      GCCGGAACATTGTAAGCTAACCCGTTCCTCTTTGTTATTTTGTTTTGTTTCTGCTGACACAGGTACTT
Hongmai          GCCGGAACATTGTAAGCTAACCCGTTCCTCTTTGTTATTTTGTTTTGTTTCTGCTGACACAGGTACTT
Wangshuibai      GCCGGAACATTGTAAGCTAACCCGTTCCTCTTTGTTATTTTGTTTTGTTTCTGCTGACACAGGTACTT
Shi 4185         GCCGGAACATTGTAAGCTAACCCGTTCCTCTTTGTTATTTTGTTTTGTTTCTGCTGACACAGGTACTT
Mazhamai         GCCGGAACATTGTAAGCTAACCCGTTCCTCTTTGTTATTTTGTTTTGTTTCTGCTGACACAGGTACTT
Xiaofoshou       GCCGGAACATTGTAAGCTAACCCGTTCCTCTTTGTTATTTTGTTTTGTTTCTGCTGACACAGGTACTT
Aikang 58        GCCGGAACATTGTAAGCTAACCCGTTCCTCTTTGTTATTTTGTTTTGTTTCTGCTGACACAGGTACTT
AL8/78           GCCGGAACATTGTAAGCTAACCCGTTCCTCTTGGTTATTTTGTTTTGTTTCTGCTGACACAGGTACTT
PI 431602        GCCGGAACATTGTAAGCTAACCCGTTCCTCTTGGTTATTTTGTTTTGTTTCTGCTGACACAGGTACTT
PI 486274        GCCGGAACATTGTAAGCTAACCCGTTCCTCTTGGTTATTTTGTTTTGTTTCTGCTGACACAGGTACTT
RM 000182        GCCGGAACATTGTAAGCTAACCCGTTCCTCTTGGTTATTTTGTTTTGTTTCTGCTGACACAGGTACTT
PI 511381        GCCGGAACATTGTAAGCTAACCCGTTCCTCTTGGTTATTTTGTTTTGTTTCTGCTGACACAGGTACTT
PI 486271        GCCGGAACATTGTAAGCTAACCCGTTCCTCTTGGTTATTTTGTTTTGTTTCTGCTGACACAGGTACTT
PI 511367        GCCGGAACATTGTAAGCTAACCCGTTCCTCTTGGTTATTTTGTTTTGTTTCTGCTGACACAGGTACTT
Chinese Spring   GCCGGAACATTGTAAGCTAACCCGTTCCTCTTGGTTATTTTGTTTTGTTTCTGCTGACACAGGTACTT
Hanzhongbai      GCCGGAACATTGTAAGCTAACCCGTTCCTCTTGGTTATTTTGTTTTGTTTCTGCTGACACAGGTACTT
Hongtoumai       GCCGGAACATTGTAAGCTAACCCGTTCCTCTTGGTTATTTTGTTTTGTTTCTGCTGACACAGGTACTT
Hongyouzi        GCCGGAACATTGTAAGCTAACCCGTTCCTCTTGGTTATTTTGTTTTGTTTCTGCTGACACAGGTACTT
Zijiehong        GCCGGAACATTGTAAGCTAACCCGTTCCTCTTGGTTATTTTGTTTTGTTTCTGCTGACACAGGTACTT
Baihuamai        GCCGGAACATTGTAAGCTAACCCGTTCCTCTTGGTTATTTTGTTTTGTTTCTGCTGACACAGGTACTT
Huixianhong      GCCGGAACATTGTAAGCTAACCCGTTCCTCTTGGTTATTTTGTTTTGTTTCTGCTGACACAGGTACTT
Baimangmai       GCCGGAACATTGTAAGCTAACCCGTTCCTCTTGGTTATTTTGTTTTGTTTCTGCTGACACAGGTACTT

                            5,790         5,800         5,810         5,820         5,830         5,840
Hulutou          CGATAACCAAAATGCATTAGAGTTAGTCGGCCGATTGCCTCACATCTAACATATGTGCAGCGGATATA
Baihulu          CGATAACCAAAATGCATTAGAGTTAGTCGGCCGATTGCCTCACATCTAACATATGTGCAGCGGATATA
Chiyacao         CGATAACCAAAATGCATTAGAGTTAGTCGGCCGATTGCCTCACATCTAACATATGTGCAGCGGATATA
Hongmangmai      CGATAACCAAAATGCATTAGAGTTAGTCGGCCGATTGCCTCACATCTAACATATGTGCAGCGGATATA
Hongmai          CGATAACCAAAATGCATTAGAGTTAGTCGGCCGATTGCCTCACATCTAACATATGTGCAGCGGATATA
Wangshuibai      CGATAACCAAAATGCATTAGAGTTAGTCGGCCGATTGCCTCACATCTAACATATGTGCAGCGGATATA
Shi 4185         CGATAACCAAAATGCATTAGAGTTAGTCGGCCGATTGCCTCACATCTAACATATGTGCAGCGGATATA
Mazhamai         CGATAACCAAAATGCATTAGAGTTAGTCGGCCGATTGCCTCACATCTAACATATGTGCAGCGGATATA
Xiaofoshou       CGATAACCAAAATGCATTAGAGTTAGTCGGCCGATTGCCTCACATCTAACATATGTGCAGCGGATATA
Aikang 58        CGATAACCAAAATGCATTAGAGTTAGTCGGCCGATTGCCTCACATCTAACATATGTGCAGCGGATATA
AL8/78           CGATAACCAAAATGCATTAGAGTTAGTCGGCCGATTGCCTCACATCTAACATATGTGCAGCGGATATA
PI 431602        CGATAACCAAAATGCATTAGAGTTAGTCGGCCGATTGCCTCACATCTAACATATGTGCAGCGGATATA
PI 486274        CGATAACCAAAATGCATTAGAGTTAGTCGGCCGATTGCCTCACATCTAACATATGTGCAGCGGATATA
RM 000182        CGATAACCAAAATGCATTAGAGTTAGTCGGCCGATTGCCTCACATCTAACATATGTGCAGGTGTTATC
PI 511381        CGATAACCAAAATGCATTAGAGTTAGTCGGCCGATTGCCTCACATCTAACATATGTGCAGGTGTTATC
PI 486271        CGATAACCAAAATGCATTAGAGTTAGTCGGCCGATTGCCTCACATCTAACATATGTGCAGGTGTTATC
PI 511367        CGATAACCAAAATGCATTAGAGTTAGTCGGCCGATTGCCTCACATCTAACATATGTGCAGGTGTTATC
Chinese Spring   CGATAACCAAAATGCATTAGAGTTAGTCGGCCGATTGCCTCACATCTAACATATGTGCAGGTGTTATC
Hanzhongbai      CGATAACCAAAATGCATTAGAGTTAGTCGGCCGATTGCCTCACATCTAACATATGTGCAGGTGTTATC
Hongtoumai       CGATAACCAAAATGCATTAGAGTTAGTCGGCCGATTGCCTCACATCTAACATATGTGCAGGTGTTATC
Hongyouzi        CGATAACCAAAATGCATTAGAGTTAGTCGGCCGATTGCCTCACATCTAACATATGTGCAGGTGTTATC
Zijiehong        CGATAACCAAAATGCATTAGAGTTAGTCGGCCGATTGCCTCACATCTAACATATGTGCAGGTGTTATC
Baihuamai        CGATAACCAAAATGCATTAGAGTTAGTCGGCCGATTGCCTCACATCTAACATATGTGCAGGTGTTATC
Huixianhong      CGATAACCAAAATGCATTAGAGTTAGTCGGCCGATTGCCTCACATCTAACATATGTGCAGGTGTTATC
Baimangmai       CGATAACCAAAATGCATTAGAGTTAGTCGGCCGATTGCCTCACATCTAACATATGTGCAGGTGTTATC
```

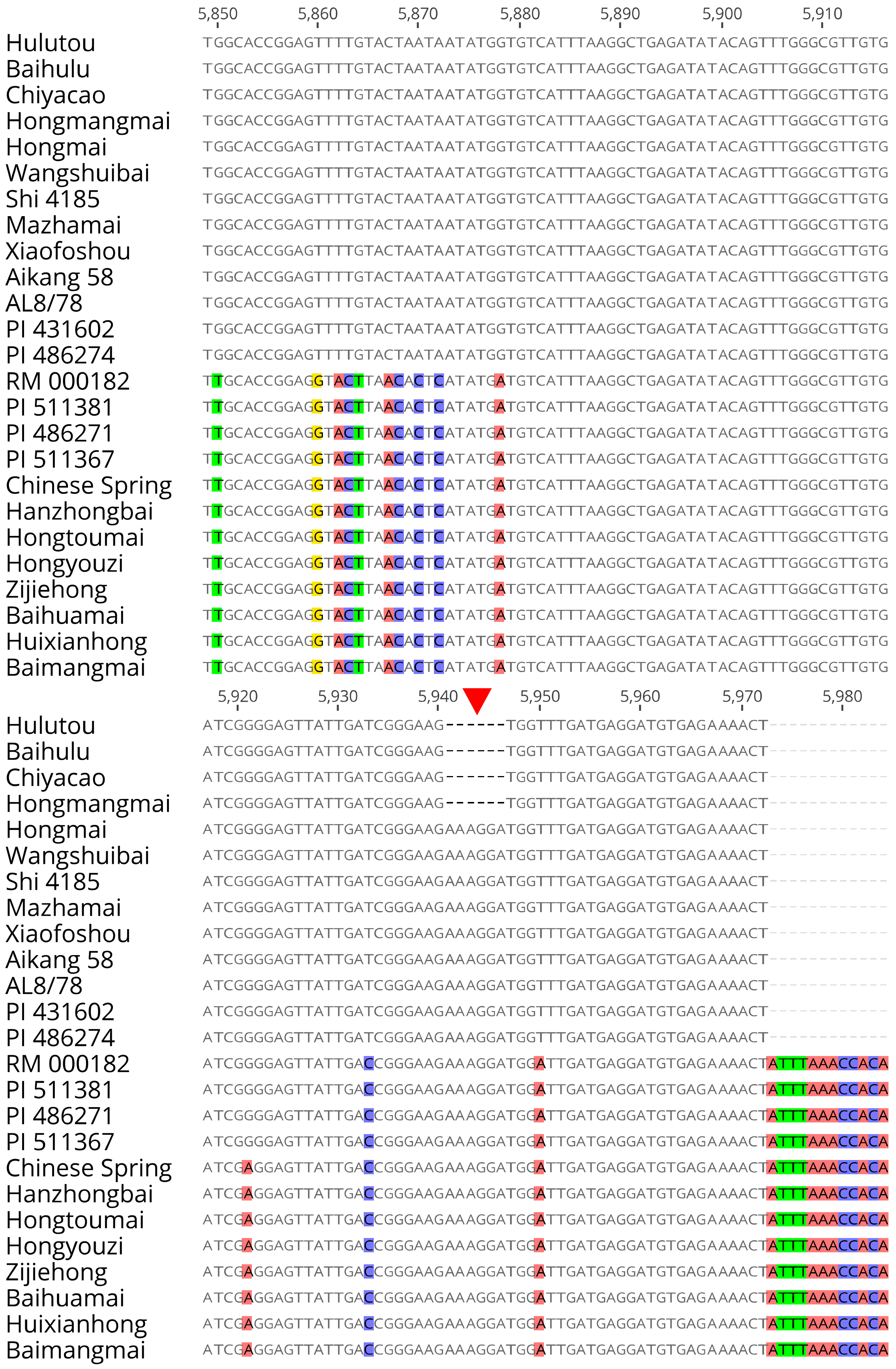
5,850 5,860 5,870 5,880 5,890 5,900 5,910
Hulutou TGGCACCGGAGTTTTGTACTAATAATATGGTGTCATTTAAGGCTGAGATATACAGTTTGGGCGTTGTG
Baihulu TGGCACCGGAGTTTTGTACTAATAATATGGTGTCATTTAAGGCTGAGATATACAGTTTGGGCGTTGTG
Chiyacao TGGCACCGGAGTTTTGTACTAATAATATGGTGTCATTTAAGGCTGAGATATACAGTTTGGGCGTTGTG
Hongmangmai TGGCACCGGAGTTTTGTACTAATAATATGGTGTCATTTAAGGCTGAGATATACAGTTTGGGCGTTGTG
Hongmai TGGCACCGGAGTTTTGTACTAATAATATGGTGTCATTTAAGGCTGAGATATACAGTTTGGGCGTTGTG
Wangshuibai TGGCACCGGAGTTTTGTACTAATAATATGGTGTCATTTAAGGCTGAGATATACAGTTTGGGCGTTGTG
Shi 4185 TGGCACCGGAGTTTTGTACTAATAATATGGTGTCATTTAAGGCTGAGATATACAGTTTGGGCGTTGTG
Mazhamai TGGCACCGGAGTTTTGTACTAATAATATGGTGTCATTTAAGGCTGAGATATACAGTTTGGGCGTTGTG
Xiaofoshou TGGCACCGGAGTTTTGTACTAATAATATGGTGTCATTTAAGGCTGAGATATACAGTTTGGGCGTTGTG
Aikang 58 TGGCACCGGAGTTTTGTACTAATAATATGGTGTCATTTAAGGCTGAGATATACAGTTTGGGCGTTGTG
AL8/78 TGGCACCGGAGTTTTGTACTAATAATATGGTGTCATTTAAGGCTGAGATATACAGTTTGGGCGTTGTG
PI 431602 TGGCACCGGAGTTTTGTACTAATAATATGGTGTCATTTAAGGCTGAGATATACAGTTTGGGCGTTGTG
PI 486274 TGGCACCGGAGTTTTGTACTAATAATATGGTGTCATTTAAGGCTGAGATATACAGTTTGGGCGTTGTG
RM 000182 TTGCACCGGAGGTACTTAACACTCATATGATGTCATTTAAGGCTGAGATATACAGTTTGGGCGTTGTG
PI 511381 TTGCACCGGAGGTACTTAACACTCATATGATGTCATTTAAGGCTGAGATATACAGTTTGGGCGTTGTG
PI 486271 TTGCACCGGAGGTACTTAACACTCATATGATGTCATTTAAGGCTGAGATATACAGTTTGGGCGTTGTG
PI 511367 TTGCACCGGAGGTACTTAACACTCATATGATGTCATTTAAGGCTGAGATATACAGTTTGGGCGTTGTG
Chinese Spring TTGCACCGGAGGTACTTAACACTCATATGATGTCATTTAAGGCTGAGATATACAGTTTGGGCGTTGTG
Hanzhongbai TTGCACCGGAGGTACTTAACACTCATATGATGTCATTTAAGGCTGAGATATACAGTTTGGGCGTTGTG
Hongtoumai TTGCACCGGAGGTACTTAACACTCATATGATGTCATTTAAGGCTGAGATATACAGTTTGGGCGTTGTG
Hongyouzi TTGCACCGGAGGTACTTAACACTCATATGATGTCATTTAAGGCTGAGATATACAGTTTGGGCGTTGTG
Zijiehong TTGCACCGGAGGTACTTAACACTCATATGATGTCATTTAAGGCTGAGATATACAGTTTGGGCGTTGTG
Baihuamai TTGCACCGGAGGTACTTAACACTCATATGATGTCATTTAAGGCTGAGATATACAGTTTGGGCGTTGTG
Huixianhong TTGCACCGGAGGTACTTAACACTCATATGATGTCATTTAAGGCTGAGATATACAGTTTGGGCGTTGTG
Baimangmai TTGCACCGGAGGTACTTAACACTCATATGATGTCATTTAAGGCTGAGATATACAGTTTGGGCGTTGTG
5,920 5,930 5,940 5,950 5,960 5,970 5,980
Hulutou ATCGGGGAGTTATTGATCGGGAAG------TGGTTTGATGAGGATGTGAGAAAACT------------
Baihulu ATCGGGGAGTTATTGATCGGGAAG------TGGTTTGATGAGGATGTGAGAAAACT------------
Chiyacao ATCGGGGAGTTATTGATCGGGAAG------TGGTTTGATGAGGATGTGAGAAAACT------------
Hongmangmai ATCGGGGAGTTATTGATCGGGAAG------TGGTTTGATGAGGATGTGAGAAAACT------------
Hongmai ATCGGGGAGTTATTGATCGGGAAGAAAGGATGGTTTGATGAGGATGTGAGAAAACT------------
Wangshuibai ATCGGGGAGTTATTGATCGGGAAGAAAGGATGGTTTGATGAGGATGTGAGAAAACT------------
Shi 4185 ATCGGGGAGTTATTGATCGGGAAGAAAGGATGGTTTGATGAGGATGTGAGAAAACT------------
Mazhamai ATCGGGGAGTTATTGATCGGGAAGAAAGGATGGTTTGATGAGGATGTGAGAAAACT------------
Xiaofoshou ATCGGGGAGTTATTGATCGGGAAGAAAGGATGGTTTGATGAGGATGTGAGAAAACT------------
Aikang 58 ATCGGGGAGTTATTGATCGGGAAGAAAGGATGGTTTGATGAGGATGTGAGAAAACT------------
AL8/78 ATCGGGGAGTTATTGATCGGGAAGAAAGGATGGTTTGATGAGGATGTGAGAAAACT------------
PI 431602 ATCGGGGAGTTATTGATCGGGAAGAAAGGATGGTTTGATGAGGATGTGAGAAAACT------------
PI 486274 ATCGGGGAGTTATTGATCGGGAAGAAAGGATGGTTTGATGAGGATGTGAGAAAACT------------
RM 000182 ATCGGGGAGTTATTGACCGGGAAGAAAGGATGGATTGATGAGGATGTGAGAAAACTATTTAAACCACA
PI 511381 ATCGGGGAGTTATTGACCGGGAAGAAAGGATGGATTGATGAGGATGTGAGAAAACTATTTAAACCACA
PI 486271 ATCGGGGAGTTATTGACCGGGAAGAAAGGATGGATTGATGAGGATGTGAGAAAACTATTTAAACCACA
PI 511367 ATCGGGGAGTTATTGACCGGGAAGAAAGGATGGATTGATGAGGATGTGAGAAAACTATTTAAACCACA
Chinese Spring ATCGAGGAGTTATTGACCGGGAAGAAAGGATGGATTGATGAGGATGTGAGAAAACTATTTAAACCACA
Hanzhongbai ATCGAGGAGTTATTGACCGGGAAGAAAGGATGGATTGATGAGGATGTGAGAAAACTATTTAAACCACA
Hongtoumai ATCGAGGAGTTATTGACCGGGAAGAAAGGATGGATTGATGAGGATGTGAGAAAACTATTTAAACCACA
Hongyouzi ATCGAGGAGTTATTGACCGGGAAGAAAGGATGGATTGATGAGGATGTGAGAAAACTATTTAAACCACA
Zijiehong ATCGAGGAGTTATTGACCGGGAAGAAAGGATGGATTGATGAGGATGTGAGAAAACTATTTAAACCACA
Baihuamai ATCGAGGAGTTATTGACCGGGAAGAAAGGATGGATTGATGAGGATGTGAGAAAACTATTTAAACCACA
Huixianhong ATCGAGGAGTTATTGACCGGGAAGAAAGGATGGATTGATGAGGATGTGAGAAAACTATTTAAACCACA
Baimangmai ATCGAGGAGTTATTGACCGGGAAGAAAGGATGGATTGATGAGGATGTGAGAAAACTATTTAAACCACA

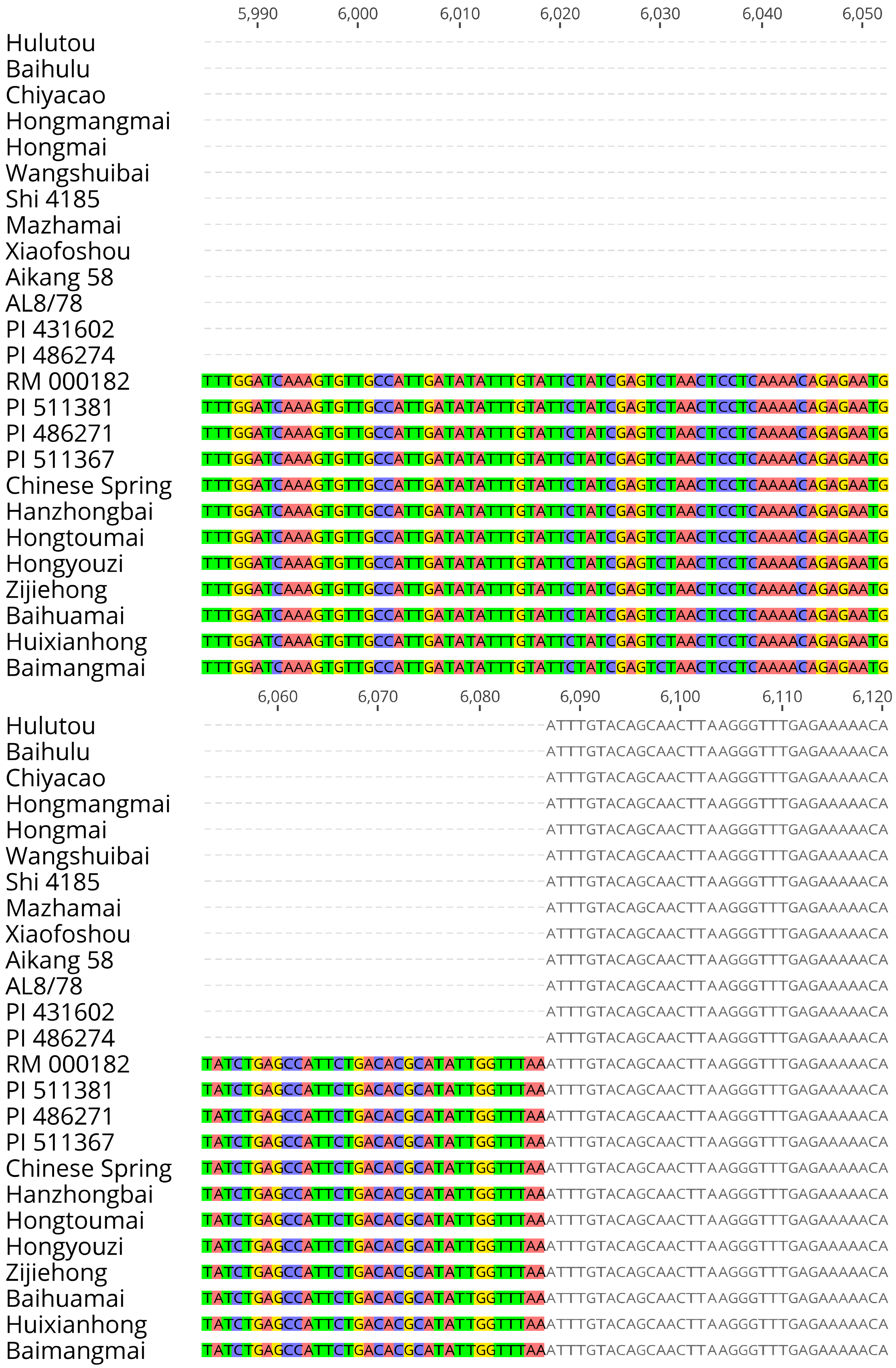

5,990 6,000 6,010 6,020 6,030 6,040 6,050
Hulutou
Baihulu
Chiyacao
Hongmangmai
Hongmai
Wangshuibai
Shi 4185
Mazhamai
Xiaofoshou
Aikang 58
AL8/78
PI 431602
PI 486274
RM 000182 TTTGGATCAAAGTGTTGCCATTGATATATTTGTATTCTATCGAGTCTAACTCCTCAAAACAGAGAATG
PI 511381 TTTGGATCAAAGTGTTGCCATTGATATATTTGTATTCTATCGAGTCTAACTCCTCAAAACAGAGAATG
PI 486271 TTTGGATCAAAGTGTTGCCATTGATATATTTGTATTCTATCGAGTCTAACTCCTCAAAACAGAGAATG
PI 511367 TTTGGATCAAAGTGTTGCCATTGATATATTTGTATTCTATCGAGTCTAACTCCTCAAAACAGAGAATG
Chinese Spring TTTGGATCAAAGTGTTGCCATTGATATATTTGTATTCTATCGAGTCTAACTCCTCAAAACAGAGAATG
Hanzhongbai TTTGGATCAAAGTGTTGCCATTGATATATTTGTATTCTATCGAGTCTAACTCCTCAAAACAGAGAATG
Hongtoumai TTTGGATCAAAGTGTTGCCATTGATATATTTGTATTCTATCGAGTCTAACTCCTCAAAACAGAGAATG
Hongyouzi TTTGGATCAAAGTGTTGCCATTGATATATTTGTATTCTATCGAGTCTAACTCCTCAAAACAGAGAATG
Zijiehong TTTGGATCAAAGTGTTGCCATTGATATATTTGTATTCTATCGAGTCTAACTCCTCAAAACAGAGAATG
Baihuamai TTTGGATCAAAGTGTTGCCATTGATATATTTGTATTCTATCGAGTCTAACTCCTCAAAACAGAGAATG
Huixianhong TTTGGATCAAAGTGTTGCCATTGATATATTTGTATTCTATCGAGTCTAACTCCTCAAAACAGAGAATG
Baimangmai TTTGGATCAAAGTGTTGCCATTGATATATTTGTATTCTATCGAGTCTAACTCCTCAAAACAGAGAATG
6,060 6,070 6,080 6,090 6,100 6,110 6,120
Hulutou ATTTGTACAGCAACTTAAGGGTTTGAGAAAAACA
Baihulu ATTTGTACAGCAACTTAAGGGTTTGAGAAAAACA
Chiyacao ATTTGTACAGCAACTTAAGGGTTTGAGAAAAACA
Hongmangmai ATTTGTACAGCAACTTAAGGGTTTGAGAAAAACA
Hongmai ATTTGTACAGCAACTTAAGGGTTTGAGAAAAACA
Wangshuibai ATTTGTACAGCAACTTAAGGGTTTGAGAAAAACA
Shi 4185 ATTTGTACAGCAACTTAAGGGTTTGAGAAAAACA
Mazhamai ATTTGTACAGCAACTTAAGGGTTTGAGAAAAACA
Xiaofoshou ATTTGTACAGCAACTTAAGGGTTTGAGAAAAACA
Aikang 58 ATTTGTACAGCAACTTAAGGGTTTGAGAAAAACA
AL8/78 ATTTGTACAGCAACTTAAGGGTTTGAGAAAAACA
PI 431602 ATTTGTACAGCAACTTAAGGGTTTGAGAAAAACA
PI 486274 ATTTGTACAGCAACTTAAGGGTTTGAGAAAAACA
RM 000182 TATCTGAGCCATTCTGACACGCATATTGGTTTAAATTTGTACAGCAACTTAAGGGTTTGAGAAAAACA
PI 511381 TATCTGAGCCATTCTGACACGCATATTGGTTTAAATTTGTACAGCAACTTAAGGGTTTGAGAAAAACA
PI 486271 TATCTGAGCCATTCTGACACGCATATTGGTTTAAATTTGTACAGCAACTTAAGGGTTTGAGAAAAACA
PI 511367 TATCTGAGCCATTCTGACACGCATATTGGTTTAAATTTGTACAGCAACTTAAGGGTTTGAGAAAAACA
Chinese Spring TATCTGAGCCATTCTGACACGCATATTGGTTTAAATTTGTACAGCAACTTAAGGGTTTGAGAAAAACA
Hanzhongbai TATCTGAGCCATTCTGACACGCATATTGGTTTAAATTTGTACAGCAACTTAAGGGTTTGAGAAAAACA
Hongtoumai TATCTGAGCCATTCTGACACGCATATTGGTTTAAATTTGTACAGCAACTTAAGGGTTTGAGAAAAACA
Hongyouzi TATCTGAGCCATTCTGACACGCATATTGGTTTAAATTTGTACAGCAACTTAAGGGTTTGAGAAAAACA
Zijiehong TATCTGAGCCATTCTGACACGCATATTGGTTTAAATTTGTACAGCAACTTAAGGGTTTGAGAAAAACA
Baihuamai TATCTGAGCCATTCTGACACGCATATTGGTTTAAATTTGTACAGCAACTTAAGGGTTTGAGAAAAACA
Huixianhong TATCTGAGCCATTCTGACACGCATATTGGTTTAAATTTGTACAGCAACTTAAGGGTTTGAGAAAAACA
Baimangmai TATCTGAGCCATTCTGACACGCATATTGGTTTAAATTTGTACAGCAACTTAAGGGTTTGAGAAAAACA

	6,130 6,140 6,150 6,160 6,170 6,180
Hulutou	TTGGTAAAAGAAGGAGCGTTTTCATCATGGGAAAACAAATACCACCAAGTTAGAACATGTATGGAGAT
Baihulu	TTGGTAAAAGAAGGAGCGTTTTCATCATGGGAAAACAAATACCACCAAGTTAGAACATGTATGGAGAT
Chiyacao	TTGGTAAAAGAAGGAGCGTTTTCATCATGGGAAAACAAATACCACCAAGTTAGAACATGTATGGAGAT
Hongmangmai	TTGGTAAAAGAAGGAGCGTTTTCATCATGGGAAAACAAATACCACCAAGTTAGAACATGTATGGAGAT
Hongmai	TTGGTAAAAGAAGGAGCGTTTTCATCATGGGAAAACAAATACCACCAAGTTAGAACATGTATGGAGAT
Wangshuibai	TTGGTAAAAGAAGGAGCGTTTTCATCATGGGAAAACAAATACCACCAAGTTAGAACATGTATGGAGAT
Shi 4185	TTGGTAAAAGAAGGAGCGTTTTCATCATGGGAAAACAAATACCACCAAGTTAGAACATGTATGGAGAT
Mazhamai	TTGGTAAAAGAAGGAGCGTTTTCATCATGGGAAAACAAATACCACCAAGTTAGAACATGTATGGAGAT
Xiaofoshou	TTGGTAAAAGAAGGAGCGTTTTCATCATGGGAAAACAAATACCACCAAGTTAGAACATGTATGGAGAT
Aikang 58	TTGGTAAAAGAAGGAGCGTTTTCATCATGGGAAAACAAATACCACCAAGTTAGAACATGTATGGAGAT
AL8/78	TTGGTAAAAGAAGGAGCGTTTTCATCATGGGAAAACAAATACCACCAAGTTAGAACATGTATGGAGAT
PI 431602	TTGGTAAAAGAAGGAGCGTTTTCATCATGGGAAAACAAATACCACCAAGTTAGAACATGTATGGAGAT
PI 486274	TTGGTAAAAGAAGGAGCGTTTTCATCATGGGAAAACAAATACCACCAAGTTAGAACATGTATGGAGAT
RM 000182	TTGGTAAAAGAAGGAGCGTTTTCATCATGGGAAAACAAATACCACCAAGTTAGAACATGTATGGAGAT
PI 511381	TTGGTAAAAGAAGGAGCGTTTTCATCATGGGAAAACAAATACCACCAAGTTAGAACATGTATGGAGAT
PI 486271	TTGGTAAAAGAAGGAGCGTTTTCATCATGGGAAAACAAATACCACCAAGTTAGAACATGTATGGAGAT
PI 511367	TTGGTAAAAGAAGGAGCGTTTTCATCATGGGAAAACAAATACCACCAAGTTAGAACATGTATGGAGAT
Chinese Spring	TTGGTAAAAGAAGGAGCGTTTTCATCATGGGAAAACAAATACCACCAAGTTAGAACATGTATGGAGAT
Hanzhongbai	TTGGTAAAAGAAGGAGCGTTTTCATCATGGGAAAACAAATACCACCAAGTTAGAACATGTATGGAGAT
Hongtoumai	TTGGTAAAAGAAGGAGCGTTTTCATCATGGGAAAACAAATACCACCAAGTTAGAACATGTATGGAGAT
Hongyouzi	TTGGTAAAAGAAGGAGCGTTTTCATCATGGGAAAACAAATACCACCAAGTTAGAACATGTATGGAGAT
Zijiehong	TTGGTAAAAGAAGGAGCGTTTTCATCATGGGAAAACAAATACCACCAAGTTAGAACATGTATGGAGAT
Baihuamai	TTGGTAAAAGAAGGAGCGTTTTCATCATGGGAAAACAAATACCACCAAGTTAGAACATGTATGGAGAT
Huixianhong	TTGGTAAAAGAAGGAGCGTTTTCATCATGGGAAAACAAATACCACCAAGTTAGAACATGTATGGAGAT
Baimangmai	TTGGTAAAAGAAGGAGCGTTTTCATCATGGGAAAACAAATACCACCAAGTTAGAACATGTATGGAGAT

	6,190 6,200 6,210 6,220 6,230 6,240 6,250
Hulutou	TGGGCAGGACTGCATAGACCCCAACCCACATAAAAGGCCCACTTTGTTGGAGATTATCCAGCGGCTTA
Baihulu	TGGGCAGGACTGCATAGACCCCAACCCACATAAAAGGCCCACTTTGTTGGAGATTATCCAGCGGCTTA
Chiyacao	TGGGCAGGACTGCATAGACCCCAACCCACATAAAAGGCCCACTTTGTTGGAGATTATCCAGCGGCTTA
Hongmangmai	TGGGCAGGACTGCATAGACCCCAACCCACATAAAAGGCCCACTTTGTTGGAGATTATCCAGCGGCTTA
Hongmai	TGGGCAGGACTGCATAGACCCCAACCCACATAAAAGGCCCACTTTGTTGGAGATTATCCAGCGGCTTA
Wangshuibai	TGGGCAGGACTGCATAGACCCCAACCCACATAAAAGGCCCACTTTGTTGGAGATTATCCAGCGGCTTA
Shi 4185	TGGGCAGGACTGCATAGACCCCAACCCACATAAAAGGCCCACTTTGTTGGAGATTATCCAGCGGCTTA
Mazhamai	TGGGCAGGACTGCATAGACCCCAACCCACATAAAAGGCCCACTTTGTTGGAGATTATCCAGCGGCTTA
Xiaofoshou	TGGGCAGGACTGCATAGACCCCAACCCACATAAAAGGCCCACTTTGTTGGAGATTATCCAGCGGCTTA
Aikang 58	TGGGCAGGACTGCATAGACCCCAACCCACATAAAAGGCCCACTTTGTTGGAGATTATCCAGCGGCTTA
AL8/78	TGGGCAGGACTGCATAGACCCCAACCCACATAAAAGGCCCACTTTGTTGGAGATTATCCAGCGGCTTA
PI 431602	TGGGCAGGACTGCATAGACCCCAACCCACATAAAAGGCCCACTTTGTTGGAGATTATCCAGCGGCTTA
PI 486274	TGGGCAGGACTGCATAGACCCCAACCCACATAAAAGGCCCACTTTGTTGGAGATTATCCAGCGGCTTA
RM 000182	TGGGCAGGACTGCATAGACCCCAACCCACATAAAAGGCCCACTTTGTT**T**GAGATTATCCAGCGGCTTA
PI 511381	TGGGCAGGACTGCATAGACCCCAACCCACATAAAAGGCCCACTTTGTT**T**GAGATTATCCAGCGGCTTA
PI 486271	TGGGCAGGACTGCATAGACCCCAACCCACATAAAAGGCCCACTTTGTT**T**GAGATTATCCAGCGGCTTA
PI 511367	TGGGCAGGACTGCATAGACCCCAACCCACATAAAAGGCCCACTTTGTT**T**GAGATTATCCAGCGGCTTA
Chinese Spring	TGGGCAGGACTGCATAGACCCCAACCCACATAAAAGGCCCACTTTGTT**T**GAGATTATCCAGCGGCTTA
Hanzhongbai	TGGGCAGGACTGCATAGACCCCAACCCACATAAAAGGCCCACTTTGTT**T**GAGATTATCCAGCGGCTTA
Hongtoumai	TGGGCAGGACTGCATAGACCCCAACCCACATAAAAGGCCCACTTTGTT**T**GAGATTATCCAGCGGCTTA
Hongyouzi	TGGGCAGGACTGCATAGACCCCAACCCACATAAAAGGCCCACTTTGTT**T**GAGATTATCCAGCGGCTTA
Zijiehong	TGGGCAGGACTGCATAGACCCCAACCCACATAAAAGGCCCACTTTGTT**T**GAGATTATCCAGCGGCTTA
Baihuamai	TGGGCAGGACTGCATAGACCCCAACCCACATAAAAGGCCCACTTTGTT**T**GAGATTATCCAGCGGCTTA
Huixianhong	TGGGCAGGACTGCATAGACCCCAACCCACATAAAAGGCCCACTTTGTT**T**GAGATTATCCAGCGGCTTA
Baimangmai	TGGGCAGGACTGCATAGACCCCAACCCACATAAAAGGCCCACTTTGTT**T**GAGATTATCCAGCGGCTTA

```
                 6,260     6,270     6,280     6,290     6,300     6,310     6,320
Hulutou          ATGAAGCGGAAGATATGAACTATTCTGCAGCATCACTTTGGCAGGTATGGAAAATCGAAAGCATAAGG
Baihulu          ATGAAGCGGAAGATATGAACTATTCTGCAGCATCACTTTGGCAGGTATGGAAAATCGAAAGCATAAGG
Chiyacao         ATGAAGCGGAAGATATGAACTATTCTGCAGCATCACTTTGGCAGGTATGGAAAATCGAAAGCATAAGG
Hongmangmai      ATGAAGCGGAAGATATGAACTATTCTGCAGCATCACTTTGGCAGGTATGGAAAATCGAAAGCATAAGG
Hongmai          ATGAAGCGGAAGATATGAACTATTCTGCAGCATCACTTTGGCAGGTATGGAAAATCGAAAGCATAAGG
Wangshuibai      ATGAAGCGGAAGATATGAACTATTCTGCAGCATCACTTTGGCAGGTATGGAAAATCGAAAGCATAAGG
Shi 4185         ATGAAGCGGAAGATATGAACTATTCTGCAGCATCACTTTGGCAGGTATGGAAAATCGAAAGCATAAGG
Mazhamai         ATGAAGCGGAAGATATGAACTATTCTGCAGCATCACTTTGGCAGGTATGGAAAATCGAAAGCATAAGG
Xiaofoshou       ATGAAGCGGAAGATATGAACTATTCTGCAGCATCACTTTGGCAGGTATGGAAAATCGAAAGCATAAGG
Aikang 58        ATGAAGCGGAAGATATGAACTATTCTGCAGCATCACTTTGGCAGGTATGGAAAATCGAAAGCATAAGG
AL8/78           ATGAAGCGGAAGATATGAACTATTCTGCAGCATCACTTTGGCAGGTATGGAAAATCGAAAGCATAAGA
PI 431602        ATGAAGCGGAAGATATGAACTATTCTGCAGCATCACTTTGGCAGGTATGGAAAATCGAAAGCATAAGA
PI 486274        ATGAAGCGGAAGATATGAACTATTCTGCAGCATCACTTTGGCAGGTATGGAAAATCGAAAGCATAAGA
RM 000182        ATGAAGCGGAAGATATGAACTATTCTGCAGCATCACTTTGGCAGGTATGGAAAATCGAAAGCATAAGA
PI 511381        ATGAAGCGGAAGATATGAACTATTCTGCAGCATCACTTTGGCAGGTATGGAAAATCGAAAGCATAAGA
PI 486271        ATGAAGCGGAAGATATGAACTATTCTGCAGCATCACTTTGGCAGGTATGGAAAATCGAAAGCATAAGA
PI 511367        ATGAAGCGGAAGATATGAACTATTCTGCAGCATCACTTTGGCAGGTATGGAAAATCGAAAGCATAAGA
Chinese Spring   ATGAAGCGGAAGATATGAACTATTCTGCAGCATCACTTTGGCAGGTATGGAAAATCGAAAGCATAAGA
Hanzhongbai      ATGAAGCGGAAGATATGAACTATTCTGCAGCATCACTTTGGCAGGTATGGAAAATCGAAAGCATAAGA
Hongtoumai       ATGAAGCGGAAGATATGAACTATTCTGCAGCATCACTTTGGCAGGTATGGAAAATCGAAAGCATAAGA
Hongyouzi        ATGAAGCGGAAGATATGAACTATTCTGCAGCATCACTTTGGCAGGTATGGAAAATCGAAAGCATAAGA
Zijiehong        ATGAAGCGGAAGATATGAACTATTCTGCAGCATCACTTTGGCAGGTATGGAAAATCGAAAGCATAAGA
Baihuamai        ATGAAGCGGAAGATATGAACTATTCTGCAGCATCACTTTGGCAGGTATGGAAAATCGAAAGCATAAGA
Huixianhong      ATGAAGCGGAAGATATGAACTATTCTGCAGCATCACTTTGGCAGGTATGGAAAATCGAAAGCATAAGA
Baimangmai       ATGAAGCGGAAGATATGAACTATTCTGCAGCATCACTTTGGCAGGTATGGAAAATCGAAAGCATAAGA

                   6,330     6,340     6,350     6,360     6,370     6,380     6,390
Hulutou          AAACAACTGTTCTTTATCTACCATTTCACTTTAGCTAACGTACTGTTTGAGAAGAATCAGTCTTAGCC
Baihulu          AAACAACTGTTCTTTATCTACCATTTCACTTTAGCTAACGTACTGTTTGAGAAGAATCAGTCTTAGCC
Chiyacao         AAACAACTGTTCTTTATCTACCATTTCACTTTAGCTAACGTACTGTTTGAGAAGAATCAGTCTTAGCC
Hongmangmai      AAACAACTGTTCTTTATCTACCATTTCACTTTAGCTAACGTACTGTTTGAGAAGAATCAGTCTTAGCC
Hongmai          AAACAACTGTTCTTTATCTACCATTTCACTTTAGCTAACGTACTGTTTGAGAAGAATCAGTCTTAGCC
Wangshuibai      AAACAACTGTTCTTTATCTACCATTTCACTTTAGCTAACGTACTGTTTGAGAAGAATCAGTCTTAGCC
Shi 4185         AAACAACTGTTCTTTATCTACCATTTCACTTTAGCTAACGTACTGTTTGAGAAGAATCAGTCTTAGCC
Mazhamai         AAACAACTGTTCTTTATCTACCATTTCACTTTAGCTAACGTACTGTTTGAGAAGAATCAGTCTTAGCC
Xiaofoshou       AAACAACTGTTCTTTATCTACCATTTCACTTTAGCTAACGTACTGTTTGAGAAGAATCAGTCTTAGCC
Aikang 58        AAACAACTGTTCTTTATCTACCATTTCACTTTAGCTAACGTACTGTTTGAGAAGAATCAGTCTTAGCC
AL8/78           AAACAACTGTTCTTTATCTACCATTTCACTTTAGCTAACGTACTGTTTGAGAAGAATCAGTCTTAGCC
PI 431602        AAACAACTGTTCTTTATCTACCATTTCACTTTAGCTAACGTACTGTTTGAGAAGAATCAGTCTTAGCC
PI 486274        AAACAACTGTTCTTTATCTACCATTTCACTTTAGCTAACGTACTGTTTGAGAAGAATCAGTCTTAGCC
RM 000182        AAACAACTGTTCTTTATCTACCATTTCACTTTAGCTAACGTACTGTTTGAGAAGAATCAGTCTTAGCC
PI 511381        AAACAACTGTTCTTTATCTACCATTTCACTTTAGCTAACGTACTGTTTGAGAAGAATCAGTCTTAGCC
PI 486271        AAACAACTGTTCTTTATCTACCATTTCACTTTAGCTAACGTACTGTTTGAGAAGAATCAGTCTTAGCC
PI 511367        AAACAACTGTTCTTTATCTACCATTTCACTTTAGCTAACGTACTGTTTGAGAAGAATCAGTCTTAGCC
Chinese Spring   AAACAACTGTTCTTTATCTACCATTTCACTTTAGCTAACGTACTGTTTGAGAAGAATCAGTCTTAGCC
Hanzhongbai      AAACAACTGTTCTTTATCTACCATTTCACTTTAGCTAACGTACTGTTTGAGAAGAATCAGTCTTAGCC
Hongtoumai       AAACAACTGTTCTTTATCTACCATTTCACTTTAGCTAACGTACTGTTTGAGAAGAATCAGTCTTAGCC
Hongyouzi        AAACAACTGTTCTTTATCTACCATTTCACTTTAGCTAACGTACTGTTTGAGAAGAATCAGTCTTAGCC
Zijiehong        AAACAACTGTTCTTTATCTACCATTTCACTTTAGCTAACGTACTGTTTGAGAAGAATCAGTCTTAGCC
Baihuamai        AAACAACTGTTCTTTATCTACCATTTCACTTTAGCTAACGTACTGTTTGAGAAGAATCAGTCTTAGCC
Huixianhong      AAACAACTGTTCTTTATCTACCATTTCACTTTAGCTAACGTACTGTTTGAGAAGAATCAGTCTTAGCC
Baimangmai       AAACAACTGTTCTTTATCTACCATTTCACTTTAGCTAACGTACTGTTTGAGAAGAATCAGTCTTAGCC
```

	6,400 6,410 6,420 6,430 6,440 6,450 6,460
Hulutou	AAGCTTACATCCTCACAGACACAGTACAGAAGTACATTTCTTTTCTTTAGCCATTACGATTAACATAA
Baihulu	AAGCTTACATCCTCACAGACACAGTACAGAAGTACATTTCTTTTCTTTAGCCATTACGATTAACATAA
Chiyacao	AAGCTTACATCCTCACAGACACAGTACAGAAGTACATTTCTTTTCTTTAGCCATTACGATTAACATAA
Hongmangmai	AAGCTTACATCCTCACAGACACAGTACAGAAGTACATTTCTTTTCTTTAGCCATTACGATTAACATAA
Hongmai	AAGCTTACATCCTCACAGACACAGTACAGAAGTACATTTCTTTTCTTTAGCCATTACGATTAACATAA
Wangshuibai	AAGCTTACATCCTCACAGACACAGTACAGAAGTACATTTCTTTTCTTTAGCCATTACGATTAACATAA
Shi 4185	AAGCTTACATCCTCACAGACACAGTACAGAAGTACATTTCTTTTCTTTAGCCATTACGATTAACATAA
Mazhamai	AAGCTTACATCCTCACAGACACAGTACAGAAGTACATTTCTTTTCTTTAGCCATTACGATTAACATAA
Xiaofoshou	AAGCTTACATCCTCACAGACACAGTACAGAAGTACATTTCTTTTCTTTAGCCATTACGATTAACATAA
Aikang 58	AAGCTTACATCCTCACAGACACAGTACAGAAGTACATTTCTTTTCTTTAGCCATTACGATTAACATAA
AL8/78	AAGCTTACATCCTCACAGACACAGTACAGAAGTACATTTGTTTTCTTTAGCCATTACGATTAACATAA
PI 431602	AAGCTTACATCCTCACAGACACAGTACAGAAGTACATTTGTTTTCTTTAGCCATTACGATTAACATAA
PI 486274	AAGCTTACATCCTCACAGACACAGTACAGAAGTACATTTGTTTTCTTTAGCCATTACGATTAACATAA
RM 000182	AAGCTTACATCCTCACAGACACAGTACAGAAGTACATTTCTTTTCTTTAGCCATTACGATTAACATAA
PI 511381	AAGCTTACATCCTCACAGACACAGTACAGAAGTACATTTCTTTTCTTTAGCCATTACGATTAACATAA
PI 486271	AAGCTTACATCCTCACAGACACAGTACAGAAGTACATTTCTTTTCTTTAGCCATTACGATTAACATAA
PI 511367	AAGCTTACATCCTCACAGACACAGTACAGAAGTACATTTCTTTTCTTTAGCCATTACGATTAACATAA
Chinese Spring	AAGCTTACATCCTCACAGACACAGTACAGAAGTACATTTCTTTTCTTTAGCCATTACGATTAACATAA
Hanzhongbai	AAGCTTACATCCTCACAGACACAGTACAGAAGTACATTTCTTTTCTTTAGCCATTACGATTAACATAA
Hongtoumai	AAGCTTACATCCTCACAGACACAGTACAGAAGTACATTTCTTTTCTTTAGCCATTACGATTAACATAA
Hongyouzi	AAGCTTACATCCTCACAGACACAGTACAGAAGTACATTTCTTTTCTTTAGCCATTACGATTAACATAA
Zijiehong	AAGCTTACATCCTCACAGACACAGTACAGAAGTACATTTCTTTTCTTTAGCCATTACGATTAACATAA
Baihuamai	AAGCTTACATCCTCACAGACACAGTACAGAAGTACATTTCTTTTCTTTAGCCATTACGATTAACATAA
Huixianhong	AAGCTTACATCCTCACAGACACAGTACAGAAGTACATTTCTTTTCTTTAGCCATTACGATTAACATAA
Baimangmai	AAGCTTACATCCTCACAGACACAGTACAGAAGTACATTTCTTTTCTTTAGCCATTACGATTAACATAA

	6,470 6,480 6,490 6,500 6,510 6,520
Hulutou	AGAGTTCACAAAGCCATCTCCAACGAAAGATATGTGCTAATTAAACTAAATTGGAAGAGAAAATACAT
Baihulu	AGAGTTCACAAAGCCATCTCCAACGAAAGATATGTGCTAATTAAACTAAATTGGAAGAGAAAATACAT
Chiyacao	AGAGTTCACAAAGCCATCTCCAACGAAAGATATGTGCTAATTAAACTAAATTGGAAGAGAAAATACAT
Hongmangmai	AGAGTTCACAAAGCCATCTCCAACGAAAGATATGTGCTAATTAAACTAAATTGGAAGAGAAAATACAT
Hongmai	AGAGTTCACAAAGCCATCTCCAACGAAAGATATGTGCTAATTAAACTAAATTGGAAGAGAAAATACAT
Wangshuibai	AGAGTTCACAAAGCCATCTCCAACGAAAGATATGTGCTAATTAAACTAAATTGGAAGAGAAAATACAT
Shi 4185	AGAGTTCACAAAGCCATCTCCAACGAAAGATATGTGCTAATTAAACTAAATTGGAAGAGAAAATACAT
Mazhamai	AGAGTTCACAAAGCCATCTCCAACGAAAGATATGTGCTAATTAAACTAAATTGGAAGAGAAAATACAT
Xiaofoshou	AGAGTTCACAAAGCCATCTCCAACGAAAGATATGTGCTAATTAAACTAAATTGGAAGAGAAAATACAT
Aikang 58	AGAGTTCACAAAGCCATCTCCAACGAAAGATATGTGCTAATTAAACTAAATTGGAAGAGAAAATACAT
AL8/78	AGAGTTCACAAAGCCATCTCCAACGAAAGATATGTGCTAATTAAACTAAATTGGAAGAGAAAATACAT
PI 431602	AGAGTTCACAAAGCCATCTCCAACGAAAGATATGTGCTAATTAAACTAAATTGGAAGAGAAAATACAT
PI 486274	AGAGTTCACAAAGCCATCTCCAACGAAAGATATGTGCTAATTAAACTAAATTGGAAGAGAAAATACAT
RM 000182	AGAGTTCACAAAGCCATCTCCAACGAAAGATATGTGCTAATTAAACTAAATTGGAAGAGAAAATACAT
PI 511381	AGAGTTCACAAAGCCATCTCCAACGAAAGATATGTGCTAATTAAACTAAATTGGAAGAGAAAATACAT
PI 486271	AGAGTTCACAAAGCCATCTCCAACGAAAGATATGTGCTAATTAAACTAAATTGGAAGAGAAAATACAT
PI 511367	AGAGTTCACAAAGCCATCTCCAACGAAAGATATGTGCTAATTAAACTAAATTGGAAGAGAAAATACAT
Chinese Spring	AGAGTTCACAAAGCCATCTCCAACGAAAGATATGTGCTAATTAAACTAAATTGGAAGAGAAAATACAT
Hanzhongbai	AGAGTTCACAAAGCCATCTCCAACGAAAGATATGTGCTAATTAAACTAAATTGGAAGAGAAAATACAT
Hongtoumai	AGAGTTCACAAAGCCATCTCCAACGAAAGATATGTGCTAATTAAACTAAATTGGAAGAGAAAATACAT
Hongyouzi	AGAGTTCACAAAGCCATCTCCAACGAAAGATATGTGCTAATTAAACTAAATTGGAAGAGAAAATACAT
Zijiehong	AGAGTTCACAAAGCCATCTCCAACGAAAGATATGTGCTAATTAAACTAAATTGGAAGAGAAAATACAT
Baihuamai	AGAGTTCACAAAGCCATCTCCAACGAAAGATATGTGCTAATTAAACTAAATTGGAAGAGAAAATACAT
Huixianhong	AGAGTTCACAAAGCCATCTCCAACGAAAGATATGTGCTAATTAAACTAAATTGGAAGAGAAAATACAT
Baimangmai	AGAGTTCACAAAGCCATCTCCAACGAAAGATATGTGCTAATTAAACTAAATTGGAAGAGAAAATACAT

	6,530 · 6,540 · 6,550 · 6,560 · 6,570 · 6,580 · 6,590
Hulutou	AACAAAGCGAAGACACCATGTAAATTAGAGAAGAAACAAACTC--TTGGTACTCCTTCAGCGAGGGAA
Baihulu	AACAAAGCGAAGACACCATGTAAATTAGAGAAGAAACAAACTC--TTGGTACTCCTTCAGCGAGGGAA
Chiyacao	AACAAAGCGAAGACACCATGTAAATTAGAGAAGAAACAAACTC--TTGGTACTCCTTCAGCGAGGGAA
Hongmangmai	AACAAAGCGAAGACACCATGTAAATTAGAGAAGAAACAAACTC--TTGGTACTCCTTCAGCGAGGGAA
Hongmai	AACAAAGCGAAGACACCATGTAAATTAGAGAAGAAACAAACTC--TTGGTACTCCTTCAGCGAGGGAA
Wangshuibai	AACAAAGCGAAGACACCATGTAAATTAGAGAAGAAACAAACTC--TTGGTACTCCTTCAGCGAGGGAA
Shi 4185	AACAAAGCGAAGACACCATGTAAATTAGAGAAGAAACAAACTC--TTGGTACTCCTTCAGCGAGGGAA
Mazhamai	AACAAAGCGAAGACACCATGTAAATTAGAGAAGAAACAAACTC--TTGGTACTCCTTCAGCGAGGGAA
Xiaofoshou	AACAAAGCGAAGACACCATGTAAATTAGAGAAGAAACAAACTC--TTGGTACTCCTTCAGCGAGGGAA
Aikang 58	AACAAAGCGAAGACACCATGTAAATTAGAGAAGAAACAAACTC--TTGGTACTCCTTCAGCGAGGGAA
AL8/78	AACAAAGCGAAGACACCATGTAAATTAGAGAAGAAACAAACTC--TTGGTACTCCTTCAGCGAGGGAA
PI 431602	AACAAAGCGAAGACACCATGTAAATTAGAGAAGAAACAAACTC--TTGGTACTCCTTCAGCGAGGGAA
PI 486274	AACAAAGCGAAGACACCATGTAAATTAGAGAAGAAACAAACTC--TTGGTACTCCTTCAGCGAGGGAA
RM 000182	AACAAAGCGAAGACACCATGTAAATTAGAGAAGAAACAAACTCTTTTGGTACTCCTTCAGCGAGGGAA
PI 511381	AACAAAGCGAAGACACCATGTAAATTAGAGAAGAAACAAACTCTTTTGGTACTCCTTCAGCGAGGGAA
PI 486271	AACAAAGCGAAGACACCATGTAAATTAGAGAAGAAACAAACTCTTTTGGTACTCCTTCAGCGAGGGAA
PI 511367	AACAAAGCGAAGACACCATGTAAATTAGAGAAGAAACAAACTCTTTTGGTACTCCTTCAGCGAGGGAA
Chinese Spring	AACAAAGCGAAGACACCATGTAAATTAGAGAAGAAACAAACTC--TTGGTACTCCTTCAGCGAGGGAA
Hanzhongbai	AACAAAGCGAAGACACCATGTAAATTAGAGAAGAAACAAACTC--TTGGTACTCCTTCAGCGAGGGAA
Hongtoumai	AACAAAGCGAAGACACCATGTAAATTAGAGAAGAAACAAACTC--TTGGTACTCCTTCAGCGAGGGAA
Hongyouzi	AACAAAGCGAAGACACCATGTAAATTAGAGAAGAAACAAACTC--TTGGTACTCCTTCAGCGAGGGAA
Zijiehong	AACAAAGCGAAGACACCATGTAAATTAGAGAAGAAACAAACTC--TTGGTACTCCTTCAGCGAGGGAA
Baihuamai	AACAAAGCGAAGACACCATGTAAATTAGAGAAGAAACAAACTC--TTGGTACTCCTTCAGCGAGGGAA
Huixianhong	AACAAAGCGAAGACACCATGTAAATTAGAGAAGAAACAAACTC--TTGGTACTCCTTCAGCGAGGGAA
Baimangmai	AACAAAGCGAAGACACCATGTAAATTAGAGAAGAAACAAACTC--TTGGTACTCCTTCAGCGAGGGAA

	6,600 · 6,610 · 6,620 · 6,630 · 6,640 · 6,650 · 6,660
Hulutou	GACACCTTGTCAACTTAGCAGAAACAAACTCTTGGTACTCCTTCAGCGAGGGAAGACACCTTGTCAAC
Baihulu	GACACCTTGTCAACTTAGCAGAAACAAACTCTTGGTACTCCTTCAGCGAGGGAAGACACCTTGTCAAC
Chiyacao	GACACCTTGTCAACTTAGCAGAAACAAACTCTTGGTACTCCTTCAGCGAGGGAAGACACCTTGTCAAC
Hongmangmai	GACACCTTGTCAACTTAGCAGAAACAAACTCTTGGTACTCCTTCAGCGAGGGAAGACACCTTGTCAAC
Hongmai	GACACCTTGTCAACTTAGCAGAAACAAACTCTTGGTACTCCTTCAGCGAGGGAAGACACCTTGTCAAC
Wangshuibai	GACACCTTGTCAACTTAGCAGAAACAAACTCTTGGTACTCCTTCAGCGAGGGAAGACACCTTGTCAAC
Shi 4185	GACACCTTGTCAACTTAGCAGAAACAAACTCTTGGTACTCCTTCAGCGAGGGAAGACACCTTGTCAAC
Mazhamai	GACACCTTGTCAACTTAGCAGAAACAAACTCTTGGTACTCCTTCAGCGAGGGAAGACACCTTGTCAAC
Xiaofoshou	GACACCTTGTCAACTTAGCAGAAACAAACTCTTGGTACTCCTTCAGCGAGGGAAGACACCTTGTCAAC
Aikang 58	GACACCTTGTCAACTTAGCAGAAACAAACTCTTGGTACTCCTTCAGCGAGGGAAGACACCTTGTCAAC
AL8/78	GACACCTTGTCAACTTAGCAGAAACAAACTCTTGGTACTCCTTCAGCGAGGGAAGACACCTTGTCAAC
PI 431602	GACACCTTGTCAACTTAGCAGAAACAAACTCTTGGTACTCCTTCAGCGAGGGAAGACACCTTGTCAAC
PI 486274	GACACCTTGTCAACTTAGCAGAAACAAACTCTTGGTACTCCTTCAGCGAGGGAAGACACCTTGTCAAC
RM 000182	GACACCTTGTCAACTTAGCAGAAAGAAACTCTTGGTACTCCTTGAGCGAGGGAAGACACCTTGTCAAC
PI 511381	GACACCTTGTCAACTTAGCAGAAAGAAACTCTTGGTACTCCTTGAGCGAGGGAAGACACCTTGTCAAC
PI 486271	GACACCTTGTCAACTTAGCAGAAAGAAACTCTTGGTACTCCTTGAGCGAGGGAAGACACCTTGTCAAC
PI 511367	GACACCTTGTCAACTTAGCAGAAAGAAACTCTTGGTACTCCTTGAGCGAGGGAAGACACCTTGTCAAC
Chinese Spring	GACACCTTGTCAACTTAGCAGAAACAAACTCTTGGTACTCCTTCAGCGAGGGAAGACACCTTGTCAAC
Hanzhongbai	GACACCTTGTCAACTTAGCAGAAACAAACTCTTGGTACTCCTTCAGCGAGGGAAGACACCTTGTCAAC
Hongtoumai	GACACCTTGTCAACTTAGCAGAAACAAACTCTTGGTACTCCTTCAGCGAGGGAAGACACCTTGTCAAC
Hongyouzi	GACACCTTGTCAACTTAGCAGAAACAAACTCTTGGTACTCCTTCAGCGAGGGAAGACACCTTGTCAAC
Zijiehong	GACACCTTGTCAACTTAGCAGAAACAAACTCTTGGTACTCCTTCAGCGAGGGAAGACACCTTGTCAAC
Baihuamai	GACACCTTGTCAACTTAGCAGAAACAAACTCTTGGTACTCCTTCAGCGAGGGAAGACACCTTGTCAAC
Huixianhong	GACACCTTGTCAACTTAGCAGAAACAAACTCTTGGTACTCCTTCAGCGAGGGAAGACACCTTGTCAAC
Baimangmai	GACACCTTGTCAACTTAGCAGAAACAAACTCTTGGTACTCCTTCAGCGAGGGAAGACACCTTGTCAAC

	6,670 6,680 6,690 6,700 6,710 6,720 6,730
Hulutou	TTAGCAGAAACCTTACGACTCTAGTCCTACATGACATGTCTAAGAGAATATTGATGTCATGCATTGCA
Baihulu	TTAGCAGAAACCTTACGACTCTAGTCCTACATGACATGTCTAAGAGAATATTGATGTCATGCATTGCA
Chiyacao	TTAGCAGAAACCTTACGACTCTAGTCCTACATGACATGTCTAAGAGAATATTGATGTCATGCATTGCA
Hongmangmai	TTAGCAGAAACCTTACGACTCTAGTCCTACATGACATGTCTAAGAGAATATTGATGTCATGCATTGCA
Hongmai	TTAGCAGAAACCTTACGACTCTAGTCCTACATGACATGTCTAAGAGAATATTGATGTCATGCATTGCA
Wangshuibai	TTAGCAGAAACCTTACGACTCTAGTCCTACATGACATGTCTAAGAGAATATTGATGTCATGCATTGCA
Shi 4185	TTAGCAGAAACCTTACGACTCTAGTCCTACATGACATGTCTAAGAGAATATTGATGTCATGCATTGCA
Mazhamai	TTAGCAGAAACCTTACGACTCTAGTCCTACATGACATGTCTAAGAGAATATTGATGTCATGCATTGCA
Xiaofoshou	TTAGCAGAAACCTTACGACTCTAGTCCTACATGACATGTCTAAGAGAATATTGATGTCATGCATTGCA
Aikang 58	TTAGCAGAAACCTTACGACTCTAGTCCTACATGACATGTCTAAGAGAATATTGATGTCATGCATTGCA
AL8/78	TTAGCAGAAACCTTACGACTCTAGTCCTACATGACATGTCTAAGAGAATATTGATATCACGCATTGCA
PI 431602	TTAGCAGAAACCTTACGACTCTAGTCCTACATGACATGTCTAAGAGAATATTGATATCACGCATTGCA
PI 486274	TTAGCAGAAACCTTACGACTCTAGTCCTACATGACATGTCTAAGAGAATATTGATATCACGCATTGCA
RM 000182	TTAGCAGAAACCTTACGACTCTAGTCCTACATGACATGTCTAAGAGAATATTGATATCACGCATTGCA
PI 511381	TTAGCAGAAACCTTACGACTCTAGTCCTACATGACATGTCTAAGAGAATATTGATATCACGCATTGCA
PI 486271	TTAGCAGAAACCTTACGACTCTAGTCCTACATGACATGTCTAAGAGAATATTGATATCACGCATTGCA
PI 511367	TTAGCAGAAACCTTACGACTCTAGTCCTACATGACATGTCTAAGAGAATATTGATATCACGCATTGCA
Chinese Spring	TTAGCAGAAACCTTACGACTCTAGTCCTACATGACATGTCTAAGAGAATATTGATATCACGCATTGCA
Hanzhongbai	TTAGCAGAAACCTTACGACTCTAGTCCTACATGACATGTCTAAGAGAATATTGATATCACGCATTGCA
Hongtoumai	TTAGCAGAAACCTTACGACTCTAGTCCTACATGACATGTCTAAGAGAATATTGATATCACGCATTGCA
Hongyouzi	TTAGCAGAAACCTTACGACTCTAGTCCTACATGACATGTCTAAGAGAATATTGATATCACGCATTGCA
Zijiehong	TTAGCAGAAACCTTACGACTCTAGTCCTACATGACATGTCTAAGAGAATATTGATATCACGCATTGCA
Baihuamai	TTAGCAGAAACCTTACGACTCTAGTCCTACATGACATGTCTAAGAGAATATTGATATCACGCATTGCA
Huixianhong	TTAGCAGAAACCTTACGACTCTAGTCCTACATGACATGTCTAAGAGAATATTGATATCACGCATTGCA
Baimangmai	TTAGCAGAAACCTTACGACTCTAGTCCTACATGACATGTCTAAGAGAATATTGATATCACGCATTGCA

	6,740 6,750 6,760 6,770 6,780 6,790 6,800
Hulutou	TAATGCTTACATGGTAAGTTATATATAAGTGCAGTCAGGAGACGAGGAATCCGATTTATCGGATACAG
Baihulu	TAATGCTTACATGGTAAGTTATATATAAGTGCAGTCAGGAGACGAGGAATCCGATTTATCGGATACAG
Chiyacao	TAATGCTTACATGGTAAGTTATATATAAGTGCAGTCAGGAGACGAGGAATCCGATTTATCGGATACAG
Hongmangmai	TAATGCTTACATGGTAAGTTATATATAAGTGCAGTCAGGAGACGAGGAATCCGATTTATCGGATACAG
Hongmai	TAATGCTTACATGGTAAGTTATATATAAGTGCAGTCAGGAGACGAGGAATCCGATTTATCGGATACAG
Wangshuibai	TAATGCTTACATGGTAAGTTATATATAAGTGCAGTCAGGAGACGAGGAATCCGATTTATCGGATACAG
Shi 4185	TAATGCTTACATGGTAAGTTATATATAAGTGCAGTCAGGAGACGAGGAATCCGATTTATCGGATACAG
Mazhamai	TAATGCTTACATGGTAAGTTATATATAAGTGCAGTCAGGAGACGAGGAATCCGATTTATCGGATACAG
Xiaofoshou	TAATGCTTACATGGTAAGTTATATATAAGTGCAGTCAGGAGACGAGGAATCCGATTTATCGGATACAG
Aikang 58	TAATGCTTACATGGTAAGTTATATATAAGTGCAGTCAGGAGACGAGGAATCCGATTTATCGGATACAG
AL8/78	TAATGCTTACATGGTAAGTTATATATAAGTGCAGTCAGGAGACGAGGAATCCGATTTATCGGATACAG
PI 431602	TAATGCTTACATGGTAAGTTATATATAAGTGCAGTCAGGAGACGAGGAATCCGATTTATCGGATACAG
PI 486274	TAATGCTTACATGGTAAGTTATATATAAGTGCAGTCAGGAGACGAGGAATCCGATTTATCGGATACAG
RM 000182	TAATGCTTACATGGTAAGTTATATATAAGTGCAGTCAGGAGACGAGGAATCCGATTTATCGGATACAG
PI 511381	TAATGCTTACATGGTAAGTTATATATAAGTGCAGTCAGGAGACGAGGAATCCGATTTATCGGATACAG
PI 486271	TAATGCTTACATGGTAAGTTATATATAAGTGCAGTCAGGAGACGAGGAATCCGATTTATCGGATACAG
PI 511367	TAATGCTTACATGGTAAGTTATATATAAGTGCAGTCAGGAGACGAGGAATCCGATTTATCGGATACAG
Chinese Spring	TAATGCTTACATGGTAAGTTATATATAAGTGCAGTCAGGAGACGAGGAATCCGATTTATCGGATACAG
Hanzhongbai	TAATGCTTACATGGTAAGTTATATATAAGTGCAGTCAGGAGACGAGGAATCCGATTTATCGGATACAG
Hongtoumai	TAATGCTTACATGGTAAGTTATATATAAGTGCAGTCAGGAGACGAGGAATCCGATTTATCGGATACAG
Hongyouzi	TAATGCTTACATGGTAAGTTATATATAAGTGCAGTCAGGAGACGAGGAATCCGATTTATCGGATACAG
Zijiehong	TAATGCTTACATGGTAAGTTATATATAAGTGCAGTCAGGAGACGAGGAATCCGATTTATCGGATACAG
Baihuamai	TAATGCTTACATGGTAAGTTATATATAAGTGCAGTCAGGAGACGAGGAATCCGATTTATCGGATACAG
Huixianhong	TAATGCTTACATGGTAAGTTATATATAAGTGCAGTCAGGAGACGAGGAATCCGATTTATCGGATACAG
Baimangmai	TAATGCTTACATGGTAAGTTATATATAAGTGCAGTCAGGAGACGAGGAATCCGATTTATCGGATACAG

6,810 6,820 6,830 6,840 6,850 6,860

Hulutou	AAGCTTTGGAGACAGAGACAACATCCGAGTTTCTTCCAAGTGACGAAGAACCCGCCTCTGTGGGCAAG
Baihulu	AAGCTTTGGAGACAGAGACAACATCCGAGTTTCTTCCAAGTGACGAAGAACCCGCCTCTGTGGGCAAG
Chiyacao	AAGCTTTGGAGACAGAGACAACATCCGAGTTTCTTCCAAGTGACGAAGAACCCGCCTCTGTGGGCAAG
Hongmangmai	AAGCTTTGGAGACAGAGACAACATCCGAGTTTCTTCCAAGTGACGAAGAACCCGCCTCTGTGGGCAAG
Hongmai	AAGCTTTGGAGACAGAGACAACATCCGAGTTTCTTCCAAGTGACGAAGAACCCGCCTCTGTGGGCAAG
Wangshuibai	AAGCTTTGGAGACAGAGACAACATCCGAGTTTCTTCCAAGTGACGAAGAACCCGCCTCTGTGGGCAAG
Shi 4185	AAGCTTTGGAGACAGAGACAACATCCGAGTTTCTTCCAAGTGACGAAGAACCCGCCTCTGTGGGCAAG
Mazhamai	AAGCTTTGGAGACAGAGACAACATCCGAGTTTCTTCCAAGTGACGAAGAACCCGCCTCTGTGGGCAAG
Xiaofoshou	AAGCTTTGGAGACAGAGACAACATCCGAGTTTCTTCCAAGTGACGAAGAACCCGCCTCTGTGGGCAAG
Aikang 58	AAGCTTTGGAGACAGAGACAACATCCGAGTTTCTTCCAAGTGACGAAGAACCCGCCTCTGTGGGCAAG
AL8/78	AAGCTTTGGAGACAGAGACAACATCCGAGTTTCTTCCAAGTGACGAGGAACCCGCCTCTGTGGGCAAG
PI 431602	AAGCTTTGGAGACAGAGACAACATCCGAGTTTCTTCCAAGTGACGAGGAACCCGCCTCTGTGGGCAAG
PI 486274	AAGCTTTGGAGACAGAGACAACATCCGAGTTTCTTCCAAGTGACGAGGAACCCGCCTCTGTGGGCAAG
RM 000182	AAGCTTTGGAGACAGAGACAACATCCGAGTTTCTTCCAAGTGACGAGGAACCCGCCTCTGTGGGCAAG
PI 511381	AAGCTTTGGAGACAGAGACAACATCCGAGTTTCTTCCAAGTGACGAGGAACCCGCCTCTGTGGGCAAG
PI 486271	AAGCTTTGGAGACAGAGACAACATCCGAGTTTCTTCCAAGTGACGAGGAACCCGCCTCTGTGGGCAAG
PI 511367	AAGCTTTGGAGACAGAGACAACATCCGAGTTTCTTCCAAGTGACGAGGAACCCGCCTCTGTGGGCAAG
Chinese Spring	AAGCTTTGGAGACAGAGACAACATCCGAGTTTCTTCCAAGTGACGAGGAACCCGCCTCTGTGGGCAAG
Hanzhongbai	AAGCTTTGGAGACAGAGACAACATCCGAGTTTCTTCCAAGTGACGAGGAACCCGCCTCTGTGGGCAAG
Hongtoumai	AAGCTTTGGAGACAGAGACAACATCCGAGTTTCTTCCAAGTGACGAGGAACCCGCCTCTGTGGGCAAG
Hongyouzi	AAGCTTTGGAGACAGAGACAACATCCGAGTTTCTTCCAAGTGACGAGGAACCCGCCTCTGTGGGCAAG
Zijiehong	AAGCTTTGGAGACAGAGACAACATCCGAGTTTCTTCCAAGTGACGAGGAACCCGCCTCTGTGGGCAAG
Baihuamai	AAGCTTTGGAGACAGAGACAACATCCGAGTTTCTTCCAAGTGACGAGGAACCCGCCTCTGTGGGCAAG
Huixianhong	AAGCTTTGGAGACAGAGACAACATCCGAGTTTCTTCCAAGTGACGAGGAACCCGCCTCTGTGGGCAAG
Baimangmai	AAGCTTTGGAGACAGAGACAACATCCGAGTTTCTTCCAAGTGACGAGGAACCCGCCTCTGTGGGCAAG

6,870 6,880 6,890 6,900 6,910 6,920 6,930

Hulutou	ACCGGAGAAACAAGCACACAGGAGCCTGATAAACCGGACCTAATAAGTAAGTTGCCAGCATCGGTGGA
Baihulu	ACCGGAGAAACAAGCACACAGGAGCCTGATAAACCGGACCTAATAAGTAAGTTGCCAGCATCGGTGGA
Chiyacao	ACCGGAGAAACAAGCACACAGGAGCCTGATAAACCGGACCTAATAAGTAAGTTGCCAGCATCGGTGGA
Hongmangmai	ACCGGAGAAACAAGCACACAGGAGCCTGATAAACCGGACCTAATAAGTAAGTTGCCAGCATCGGTGGA
Hongmai	ACCGGAGAAACAAGCACACAGGAGCCTGATAAACCGGACCTAATAAGTAAGTTGCCAGCATCGGTGGA
Wangshuibai	ACCGGAGAAACAAGCACACAGGAGCCTGATAAACCGGACCTAATAAGTAAGTTGCCAGCATCGGTGGA
Shi 4185	ACCGGAGAAACAAGCACACAGGAGCCTGATAAACCGGACCTAATAAGTAAGTTGCCAGCATCGGTGGA
Mazhamai	ACCGGAGAAACAAGCACACAGGAGCCTGATAAACCGGACCTAATAAGTAAGTTGCCAGCATCGGTGGA
Xiaofoshou	ACCGGAGAAACAAGCACACAGGAGCCTGATAAACCGGACCTAATAAGTAAGTTGCCAGCATCGGTGGA
Aikang 58	ACCGGAGAAACAAGCACACAGGAGCCTGATAAACCGGACCTAATAAGTAAGTTGCCAGCATCGGTGGA
AL8/78	ACCGGAGAAACAAGCACACAGGAGCCTGATAAACCGGACCTAGTAAGTAAGTTGCCAGCATCGGTGGA
PI 431602	ACCGGAGAAACAAGCACACAGGAGCCTGATAAACCGGACCTAGTAAGTAAGTTGCCAGCATCGGTGGA
PI 486274	ACCGGAGAAACAAGCACACAGGAGCCTGATAAACCGGACCTAGTAAGTAAGTTGCCAGCATCGGTGGA
RM 000182	ACCGGAGAAACAAGCACACAGGAGCCTGATAAACCGGACCTAGTAAGTAAGTTGCCAGCATCGGTGGA
PI 511381	ACCGGAGAAACAAGCACACAGGAGCCTGATAAACCGGACCTAGTAAGTAAGTTGCCAGCATCGGTGGA
PI 486271	ACCGGAGAAACAAGCACACAGGAGCCTGATAAACCGGACCTAGTAAGTAAGTTGCCAGCATCGGTGGA
PI 511367	ACCGGAGAAACAAGCACACAGGAGCCTGATAAACCGGACCTAGTAAGTAAGTTGCCAGCATCGGTGGA
Chinese Spring	ACCGGAGAAACAAGCACACAGGAGCCTGATAAACCGGACCTAGTAAGTAAGTTGCCAGCATCGGTGGA
Hanzhongbai	ACCGGAGAAACAAGCACACAGGAGCCTGATAAACCGGACCTAGTAAGTAAGTTGCCAGCATCGGTGGA
Hongtoumai	ACCGGAGAAACAAGCACACAGGAGCCTGATAAACCGGACCTAGTAAGTAAGTTGCCAGCATCGGTGGA
Hongyouzi	ACCGGAGAAACAAGCACACAGGAGCCTGATAAACCGGACCTAGTAAGTAAGTTGCCAGCATCGGTGGA
Zijiehong	ACCGGAGAAACAAGCACACAGGAGCCTGATAAACCGGACCTAGTAAGTAAGTTGCCAGCATCGGTGGA
Baihuamai	ACCGGAGAAACAAGCACACAGGAGCCTGATAAACCGGACCTAGTAAGTAAGTTGCCAGCATCGGTGGA
Huixianhong	ACCGGAGAAACAAGCACACAGGAGCCTGATAAACCGGACCTAGTAAGTAAGTTGCCAGCATCGGTGGA
Baimangmai	ACCGGAGAAACAAGCACACAGGAGCCTGATAAACCGGACCTAGTAAGTAAGTTGCCAGCATCGGTGGA

	6,940 · 6,950 · 6,960 · 6,970 · 6,980 · 6,990 · 7,000
Hulutou	CCTGTCTGACCTAAAAGTCCTGGAGAAAATCACAGATGATTTTTCACACGAAAGAATAGTT-GGGAAG
Baihulu	CCTGTCTGACCTAAAAGTCCTGGAGAAAATCACAGATGATTTTTCACACGAAAGAATAGTT-GGGAAG
Chiyacao	CCTGTCTGACCTAAAAGTCCTGGAGAAAATCACAGATGATTTTTCACACGAAAGAATAGTT-GGGAAG
Hongmangmai	CCTGTCTGACCTAAAAGTCCTGGAGAAAATCACAGATGATTTTTCACACGAAAGAATAGTT-GGGAAG
Hongmai	CCTGTCTGACCTAAAAGTCCTGGAGAAAATCACAGATGATTTTTCACACGAAAGAATAGTT-GGGAAG
Wangshuibai	CCTGTCTGACCTAAAAGTCCTGGAGAAAATCACAGATGATTTTTCACACGAAAGAATAGTT-GGGAAG
Shi 4185	CCTGTCTGACCTAAAAGTCCTGGAGAAAATCACAGATGATTTTTCACACGAAAGAATAGTT-GGGAAG
Mazhamai	CCTGTCTGACCTAAAAGTCCTGGAGAAAATCACAGATGATTTTTCACACGAAAGAATAGTT-GGGAAG
Xiaofoshou	CCTGTCTGACCTAAAAGTCCTGGAGAAAATCACAGATGATTTTTCACACGAAAGAATAGTT-GGGAAG
Aikang 58	CCTGTCTGACCTAAAAGTCCTGGAGAAAATCACAGATGATTTTTCACACGAAAGAATAGTT-GGGAAG
AL8/78	CCTGTCTGACCTAAAAGTCCTGGAGAAAATCACAGATGATTTCTCACACGAAAGAATAGTT-GGGAAG
PI 431602	CCTGTCTGACCTAAAAGTCCTGGAGAAAATCACAGATGATTTCTCACACGAAAGAATAGTT-GGGAAG
PI 486274	CCTGTCTGACCTAAAAGTCCTGGAGAAAATCACAGATGATTTCTCACACGAAAGAATAGTT-GGGAAG
RM 000182	CCTGTCTGACCTAAAAGTCCTGGAGAAAATCACAGATGATTTTTCACACGAAAGAATAGTT-GGGAAG
PI 511381	CCTGTCTGACCTAAAAGTCCTGGAGAAAATCACAGATGATTTTTCACACGAAAGAATAGTT-GGGAAG
PI 486271	CCTGTCTGACCTAAAAGTCCTGGAGAAAATCACAGATGATTTTTCACACGAAAGAATAGTT-GGGAAG
PI 511367	CCTGTCTGACCTAAAAGTCCTGGAGAAAATCACAGATGATTTTTCACACGAAAGAATAGTT-GGGAAG
Chinese Spring	CCTGTCTGACCTAAAAGTCCTGGAGAAAATCACAGATGATTTTTCACACGAAAGAATAGTT-GGGAAG
Hanzhongbai	CCTGTCTGACCTAAAAGTCCTGGAGAAAATCACAGATGATTTTTCACACGAAAGAATAGTT-GGGAAG
Hongtoumai	CCTGTCTGACCTAAAAGTCCTGGAGAAAATCACAGATGATTTTTCACACGAAAGAATAGTT-GGGAAG
Hongyouzi	CCTGTCTGACCTAAAAGTCCTGGAGAAAATCACAGATGATTTTTCACACGAAAGAATAGTT-GGGAAG
Zijiehong	CCTGTCTGACCTAAAAGTCCTGGAGAAAATCACAGATGATTTTTCACACGAAAGAATAGTT-GGGAAG
Baihuamai	CCTGTCTGACCTAAAAGTCCTGGAGAAAATCACAGATGATTTTTCACACGAAAGAATAGTT-GGGAAG
Huixianhong	CCTGTCTGACCTAAAAGTCCTGGAGAAAATCACAGATGATTTTTCACACGAAAGAATAGTT-GGGAAG
Baimangmai	CCTGTCTGACCTAAAAGTCCTGGAGAAAATCACAGATGATTTTTCACACGAAAGAATAGTT-GGGAAG

	7,010 · 7,020 · 7,030 · 7,040 · 7,050 · 7,060 · 7,070
Hulutou	GACGGTACA-TTCAAAGGTTGTCATAA-GGCATTTG-TTTATAAGGTACACA-TAGTTGTAAGTT-AC
Baihulu	GACGGTACA-TTCAAAGGTTGTCATAA-GGCATTTG-TTTATAAGGTACACA-TAGTTGTAAGTT-AC
Chiyacao	GACGGTACA-TTCAAAGGTTGTCATAA-GGCATTTG-TTTATAAGGTACACA-TAGTTGTAAGTT-AC
Hongmangmai	GACGGTACA-TTCAAAGGTTGTCATAA-GGCATTTG-TTTATAAGGTACACA-TAGTTGTAAGTT-AC
Hongmai	GACGGTACA-TTCAAAGGTTGTCATAA-GGCATTTG-TTTATAAGGTACACA-TAGTTGTAAGTT-AC
Wangshuibai	GACGGTACA-TTCAAAGGTTGTCATAA-GGCATTTG-TTTATAAGGTACACA-TAGTTGTAAGTT-AC
Shi 4185	GACGGTACA-TTCAAAGGTTGTCATAA-GGCATTTG-TTTATAAGGTACACA-TAGTTGTAAGTT-AC
Mazhamai	GACGGTACA-TTCAAAGGTTGTCATAA-GGCATTTG-TTTATAAGGTACACA-TAGTTGTAAGTT-AC
Xiaofoshou	GACGGTACA-TTCAAAGGTTGTCATAA-GGCATTTG-TTTATAAGGTACACA-TAGTTGTAAGTT-AC
Aikang 58	GACGGTACA-TTCAAAGGTTGTCATAA-GGCATTTG-TTTATAAGGTACACA-TAGTTGTAAGTT-AC
AL8/78	GACGGTACA-TTCAAAGGTTGTCATAA-GGCATTTG-TTTATAAGGTACACA-TAGTTGTAAGTT-AC
PI 431602	GACGGTACA-TTCAAAGGTTGTCATAA-GGCATTTG-TTTATAAGGTACACA-TAGTTGTAAGTT-AC
PI 486274	GACGGTACA-TTCAAAGGTTGTCATAA-GGCATTTG-TTTATAAGGTACACA-TAGTTGTAAGTT-AC
RM 000182	GACGGTACA-TTCAAAGGTTGTCATAA-GGCATTTG-TTTATAAGGTACACA-TAGTTGTAAGTT-AC
PI 511381	GACGGTACA-TTCAAAGGTTGTCATAA-GGCATTTG-TTTATAAGGTACACA-TAGTTGTAAGTT-AC
PI 486271	GACGGTACA-TTCAAAGGTTGTCATAA-GGCATTTG-TTTATAAGGTACACA-TAGTTGTAAGTT-AC
PI 511367	GACGGTACA-TTCAAAGGTTGTCATAA-GGCATTTG-TTTATAAGGTACACA-TAGTTGTAAGTT-AC
Chinese Spring	GACGGTACA-TTCAAAGGTTGTCATAA-GGCATTTG-TTTATAAGGTACACA-TAGTTGTAAGTT-AC
Hanzhongbai	GACGGTACA-TTCAAAGGTTGTCATAA-GGCATTTG-TTTATAAGGTACACA-TAGTTGTAAGTT-AC
Hongtoumai	GACGGTACA-TTCAAAGGTTGTCATAA-GGCATTTG-TTTATAAGGTACACA-TAGTTGTAAGTT-AC
Hongyouzi	GACGGTACA-TTCAAAGGTTGTCATAA-GGCATTTG-TTTATAAGGTACACA-TAGTTGTAAGTT-AC
Zijiehong	GACGGTACA-TTCAAAGGTTGTCATAA-GGCATTTG-TTTATAAGGTACACA-TAGTTGTAAGTT-AC
Baihuamai	GACGGTACA-TTCAAAGGTTGTCATAA-GGCATTTG-TTTATAAGGTACACA-TAGTTGTAAGTT-AC
Huixianhong	GACGGTACA-TTCAAAGGTTGTCATAA-GGCATTTG-TTTATAAGGTACACA-TAGTTGTAAGTT-AC
Baimangmai	GACGGTACA-TTCAAAGGTTGTCATAA-GGCATTTG-TTTATAAGGTACACA-TAGTTGTAAGTT-AC

	7,080 · 7,090 · 7,100 · 7,110 · 7,120 · 7,130 · 7,140
Hulutou	TATGCAGTGCTTTGCTTTGCTGAT-GTCCTTTGAGTTCAACATATTCTTCCATCTTAATTATACTCCT
Baihulu	TATGCAGTGCTTTGCTTTGCTGAT-GTCCTTTGAGTTCAACATATTCTTCCATCTTAATTATACTCCT
Chiyacao	TATGCAGTGCTTTGCTTTGCTGAT-GTCCTTTGAGTTCAACATATTCTTCCATCTTAATTATACTCCT
Hongmangmai	TATGCAGTGCTTTGCTTTGCTGAT-GTCCTTTGAGTTCAACATATTCTTCCATCTTAATTATACTCCT
Hongmai	TATGCAGTGCTTTGCTTTGCTGAT-GTCCTTTGAGTTCAACATATTCTTCCATCTTAATTATACTCCT
Wangshuibai	TATGCAGTGCTTTGCTTTGCTGAT-GTCCTTTGAGTTCAACATATTCTTCCATCTTAATTATACTCCT
Shi 4185	TATGCAGTGCTTTGCTTTGCTGAT-GTCCTTTGAGTTCAACATATTCTTCCATCTTAATTATACTCCT
Mazhamai	TATGCAGTGCTTTGCTTTGCTGAT-GTCCTTTGAGTTCAACATATTCTTCCATCTTAATTATACTCCT
Xiaofoshou	TATGCAGTGCTTTGCTTTGCTGAT-GTCCTTTGAGTTCAACATATTCTTCCATCTTAATTATACTCCT
Aikang 58	TATGCAGTGCTTTGCTTTGCTGAT-GTCCTTTGAGTTCAACATATTCTTCCATCTTAATTATACTCCT
AL8/78	TATGCAGTGCTTTGCTTTGCTGAT-GTCCTTTGAGTTCAACATATTCTTCCATCTTAATTATACTCCT
PI 431602	TATGCAGTGCTTTGCTTTGCTGAT-GTCCTTTGAGTTCAACATATTCTTCCATCTTAATTATACTCCT
PI 486274	TATGCAGTGCTTTGCTTTGCTGAT-GTCCTTTGAGTTCAACATATTCTTCCATCTTAATTATACTCCT
RM 000182	TATGCAGTGCTTTGCTTTGCTGAT-GTCCTTTGAGTTCAACATATTCTTCCATCTTAATTATACTCCT
PI 511381	TATGCAGTGCTTTGCTTTGCTGAT-GTCCTTTGAGTTCAACATATTCTTCCATCTTAATTATACTCCT
PI 486271	TATGCAGTGCTTTGCTTTGCTGAT-GTCCTTTGAGTTCAACATATTCTTCCATCTTAATTATACTCCT
PI 511367	TATGCAGTGCTTTGCTTTGCTGAT-GTCCTTTGAGTTCAACATATTCTTCCATCTTAATTATACTCCT
Chinese Spring	TATGCAGTGCTTTGCTTTGCTGAT-GTCCTTTGAGTTCAACATATTCTTCCATCTTAATTATACTCCT
Hanzhongbai	TATGCAGTGCTTTGCTTTGCTGAT-GTCCTTTGAGTTCAACATATTCTTCCATCTTAATTATACTCCT
Hongtoumai	TATGCAGTGCTTTGCTTTGCTGAT-GTCCTTTGAGTTCAACATATTCTTCCATCTTAATTATACTCCT
Hongyouzi	TATGCAGTGCTTTGCTTTGCTGAT-GTCCTTTGAGTTCAACATATTCTTCCATCTTAATTATACTCCT
Zijiehong	TATGCAGTGCTTTGCTTTGCTGAT-GTCCTTTGAGTTCAACATATTCTTCCATCTTAATTATACTCCT
Baihuamai	TATGCAGTGCTTTGCTTTGCTGAT-GTCCTTTGAGTTCAACATATTCTTCCATCTTAATTATACTCCT
Huixianhong	TATGCAGTGCTTTGCTTTGCTGAT-GTCCTTTGAGTTCAACATATTCTTCCATCTTAATTATACTCCT
Baimangmai	TATGCAGTGCTTTGCTTTGCTGAT-GTCCTTTGAGTTCAACATATTCTTCCATCTTAATTATACTCCT

	7,150 · 7,160 · 7,170 · 7,180 · 7,190 · 7,200
Hulutou	AACACACACACGCATACTAAATGTACATTTATTTGAACAGGGTGACATTCCACTTAGAGAAATGATAG
Baihulu	AACACACACACGCATACTAAATGTACATTTATTTGAACAGGGTGACATTCCACTTAGAGAAATGATAG
Chiyacao	AACACACACACGCATACTAAATGTACATTTATTTGAACAGGGTGACATTCCACTTAGAGAAATGATAG
Hongmangmai	AACACACACACGCATACTAAATGTACATTTATTTGAACAGGGTGACATTCCACTTAGAGAAATGATAG
Hongmai	AACACACACACGCATACTAAATGTACATTTATTTGAACAGGGTGACATTCCACTTAGAGAAATGATAG
Wangshuibai	AACACACACACGCATACTAAATGTACATTTATTTGAACAGGGTGACATTCCACTTAGAGAAATGATAG
Shi 4185	AACACACACACGCATACTAAATGTACATTTATTTGAACAGGGTGACATTCCACTTAGAGAAATGATAG
Mazhamai	AACACACACACGCATACTAAATGTACATTTATTTGAACAGGGTGACATTCCACTTAGAGAAATGATAG
Xiaofoshou	AACACACACACGCATACTAAATGTACATTTATTTGAACAGGGTGACATTCCACTTAGAGAAATGATAG
Aikang 58	AACACACACACGCATACTAAATGTACATTTATTTGAACAGGGTGACATTCCACTTAGAGAAATGATAG
AL8/78	AACACACACACGCATACTAAATGTACATTTATTTGAACAGGGTGACATTCCACGTAGAGAAATGATAG
PI 431602	AACACACACACGCATACTAAATGTACATTTATTTGAACAGGGTGACATTCCACGTAGAGAAATGATAG
PI 486274	AACACACACACGCATACTAAATGTACATTTATTTGAACAGGGTGACATTCCACGTAGAGAAATGATAG
RM 000182	AACACACACACGCATACTAAATGTACATTTATTTGAACAGGGTGACATTCCACTTAGAGAAATGATAG
PI 511381	AACACACACACGCATACTAAATGTACATTTATTTGAACAGGGTGACATTCCACTTAGAGAAATGATAG
PI 486271	AACACACACACGCATACTAAATGTACATTTATTTGAACAGGGTGACATTCCACTTAGAGAAATGATAG
PI 511367	AACACACACACGCATACTAAATGTACATTTATTTGAACAGGGTGACATTCCACTTAGAGAAATGATAG
Chinese Spring	AACACACACACGCATACTAAATGTACATTTATTTGAACAGGGTGACATTCCACGTAGAGAAATGATAG
Hanzhongbai	AACACACACACGCATACTAAATGTACATTTATTTGAACAGGGTGACATTCCACGTAGAGAAATGATAG
Hongtoumai	AACACACACACGCATACTAAATGTACATTTATTTGAACAGGGTGACATTCCACGTAGAGAAATGATAG
Hongyouzi	AACACACACACGCATACTAAATGTACATTTATTTGAACAGGGTGACATTCCACGTAGAGAAATGATAG
Zijiehong	AACACACACACGCATACTAAATGTACATTTATTTGAACAGGGTGACATTCCACGTAGAGAAATGATAG
Baihuamai	AACACACACACGCATACTAAATGTACATTTATTTGAACAGGGTGACATTCCACGTAGAGAAATGATAG
Huixianhong	AACACACACACGCATACTAAATGTACATTTATTTGAACAGGGTGACATTCCACGTAGAGAAATGATAG
Baimangmai	AACACACACACGCATACTAAATGTACATTTATTTGAACAGGGTGACATTCCACGTAGAGAAATGATAG

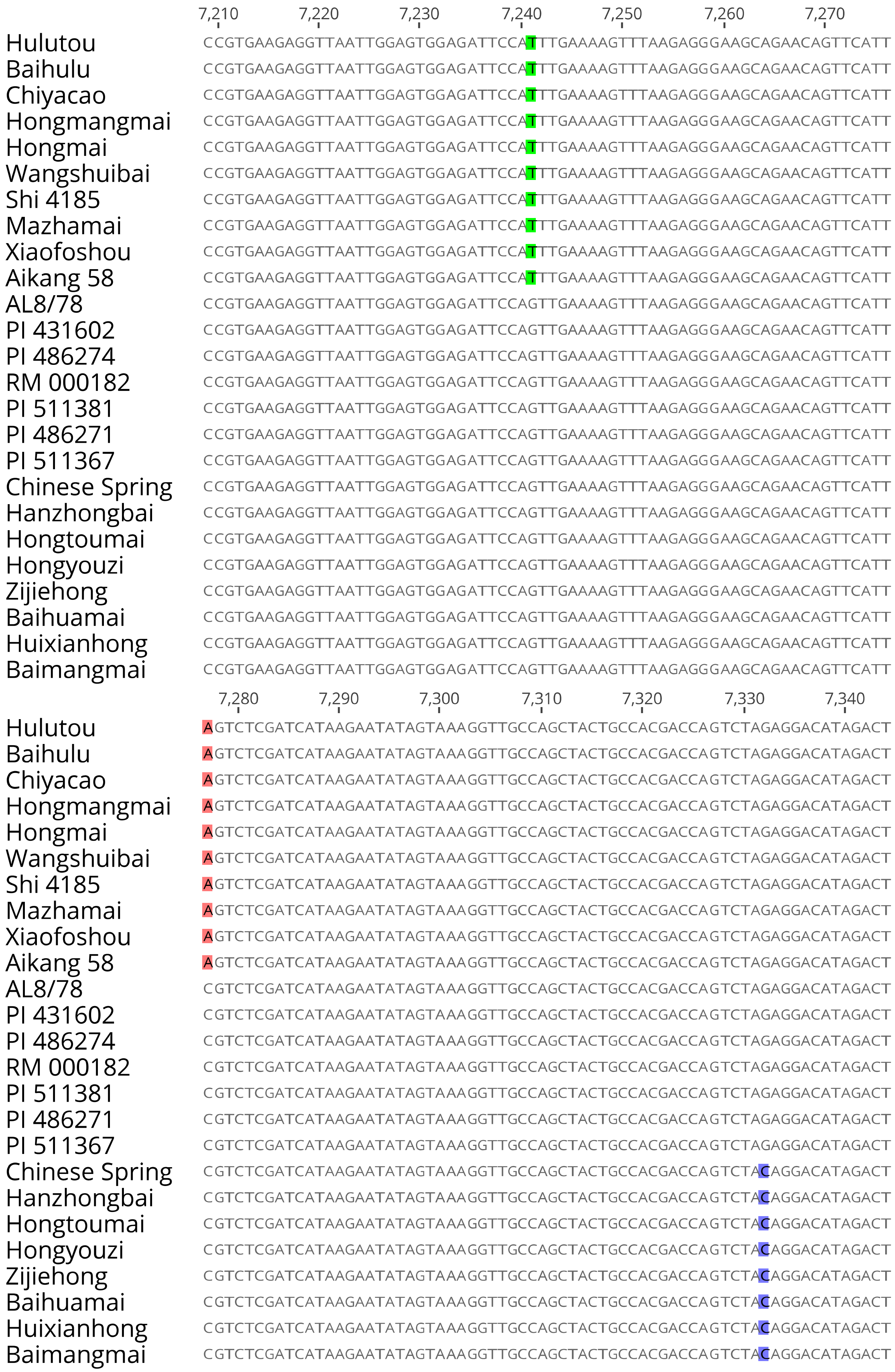
7,210 7,220 7,230 7,240 7,250 7,260 7,270
Hulutou CCGTGAAGAGGTTAATTGGAGTGGAGATTCCATTTGAAAAGTTTAAGAGGGAAGCAGAACAGTTCATT
Baihulu CCGTGAAGAGGTTAATTGGAGTGGAGATTCCATTTGAAAAGTTTAAGAGGGAAGCAGAACAGTTCATT
Chiyacao CCGTGAAGAGGTTAATTGGAGTGGAGATTCCATTTGAAAAGTTTAAGAGGGAAGCAGAACAGTTCATT
Hongmangmai CCGTGAAGAGGTTAATTGGAGTGGAGATTCCATTTGAAAAGTTTAAGAGGGAAGCAGAACAGTTCATT
Hongmai CCGTGAAGAGGTTAATTGGAGTGGAGATTCCATTTGAAAAGTTTAAGAGGGAAGCAGAACAGTTCATT
Wangshuibai CCGTGAAGAGGTTAATTGGAGTGGAGATTCCATTTGAAAAGTTTAAGAGGGAAGCAGAACAGTTCATT
Shi 4185 CCGTGAAGAGGTTAATTGGAGTGGAGATTCCATTTGAAAAGTTTAAGAGGGAAGCAGAACAGTTCATT
Mazhamai CCGTGAAGAGGTTAATTGGAGTGGAGATTCCATTTGAAAAGTTTAAGAGGGAAGCAGAACAGTTCATT
Xiaofoshou CCGTGAAGAGGTTAATTGGAGTGGAGATTCCATTTGAAAAGTTTAAGAGGGAAGCAGAACAGTTCATT
Aikang 58 CCGTGAAGAGGTTAATTGGAGTGGAGATTCCATTTGAAAAGTTTAAGAGGGAAGCAGAACAGTTCATT
AL8/78 CCGTGAAGAGGTTAATTGGAGTGGAGATTCCAGTTGAAAAGTTTAAGAGGGAAGCAGAACAGTTCATT
PI 431602 CCGTGAAGAGGTTAATTGGAGTGGAGATTCCAGTTGAAAAGTTTAAGAGGGAAGCAGAACAGTTCATT
PI 486274 CCGTGAAGAGGTTAATTGGAGTGGAGATTCCAGTTGAAAAGTTTAAGAGGGAAGCAGAACAGTTCATT
RM 000182 CCGTGAAGAGGTTAATTGGAGTGGAGATTCCAGTTGAAAAGTTTAAGAGGGAAGCAGAACAGTTCATT
PI 511381 CCGTGAAGAGGTTAATTGGAGTGGAGATTCCAGTTGAAAAGTTTAAGAGGGAAGCAGAACAGTTCATT
PI 486271 CCGTGAAGAGGTTAATTGGAGTGGAGATTCCAGTTGAAAAGTTTAAGAGGGAAGCAGAACAGTTCATT
PI 511367 CCGTGAAGAGGTTAATTGGAGTGGAGATTCCAGTTGAAAAGTTTAAGAGGGAAGCAGAACAGTTCATT
Chinese Spring CCGTGAAGAGGTTAATTGGAGTGGAGATTCCAGTTGAAAAGTTTAAGAGGGAAGCAGAACAGTTCATT
Hanzhongbai CCGTGAAGAGGTTAATTGGAGTGGAGATTCCAGTTGAAAAGTTTAAGAGGGAAGCAGAACAGTTCATT
Hongtoumai CCGTGAAGAGGTTAATTGGAGTGGAGATTCCAGTTGAAAAGTTTAAGAGGGAAGCAGAACAGTTCATT
Hongyouzi CCGTGAAGAGGTTAATTGGAGTGGAGATTCCAGTTGAAAAGTTTAAGAGGGAAGCAGAACAGTTCATT
Zijiehong CCGTGAAGAGGTTAATTGGAGTGGAGATTCCAGTTGAAAAGTTTAAGAGGGAAGCAGAACAGTTCATT
Baihuamai CCGTGAAGAGGTTAATTGGAGTGGAGATTCCAGTTGAAAAGTTTAAGAGGGAAGCAGAACAGTTCATT
Huixianhong CCGTGAAGAGGTTAATTGGAGTGGAGATTCCAGTTGAAAAGTTTAAGAGGGAAGCAGAACAGTTCATT
Baimangmai CCGTGAAGAGGTTAATTGGAGTGGAGATTCCAGTTGAAAAGTTTAAGAGGGAAGCAGAACAGTTCATT
7,280 7,290 7,300 7,310 7,320 7,330 7,340
Hulutou AGTCTCGATCATAAGAATATAGTAAAGGTTGCCAGCTACTGCCACGACCAGTCTAGAGGACATAGACT
Baihulu AGTCTCGATCATAAGAATATAGTAAAGGTTGCCAGCTACTGCCACGACCAGTCTAGAGGACATAGACT
Chiyacao AGTCTCGATCATAAGAATATAGTAAAGGTTGCCAGCTACTGCCACGACCAGTCTAGAGGACATAGACT
Hongmangmai AGTCTCGATCATAAGAATATAGTAAAGGTTGCCAGCTACTGCCACGACCAGTCTAGAGGACATAGACT
Hongmai AGTCTCGATCATAAGAATATAGTAAAGGTTGCCAGCTACTGCCACGACCAGTCTAGAGGACATAGACT
Wangshuibai AGTCTCGATCATAAGAATATAGTAAAGGTTGCCAGCTACTGCCACGACCAGTCTAGAGGACATAGACT
Shi 4185 AGTCTCGATCATAAGAATATAGTAAAGGTTGCCAGCTACTGCCACGACCAGTCTAGAGGACATAGACT
Mazhamai AGTCTCGATCATAAGAATATAGTAAAGGTTGCCAGCTACTGCCACGACCAGTCTAGAGGACATAGACT
Xiaofoshou AGTCTCGATCATAAGAATATAGTAAAGGTTGCCAGCTACTGCCACGACCAGTCTAGAGGACATAGACT
Aikang 58 AGTCTCGATCATAAGAATATAGTAAAGGTTGCCAGCTACTGCCACGACCAGTCTAGAGGACATAGACT
AL8/78 CGTCTCGATCATAAGAATATAGTAAAGGTTGCCAGCTACTGCCACGACCAGTCTAGAGGACATAGACT
PI 431602 CGTCTCGATCATAAGAATATAGTAAAGGTTGCCAGCTACTGCCACGACCAGTCTAGAGGACATAGACT
PI 486274 CGTCTCGATCATAAGAATATAGTAAAGGTTGCCAGCTACTGCCACGACCAGTCTAGAGGACATAGACT
RM 000182 CGTCTCGATCATAAGAATATAGTAAAGGTTGCCAGCTACTGCCACGACCAGTCTAGAGGACATAGACT
PI 511381 CGTCTCGATCATAAGAATATAGTAAAGGTTGCCAGCTACTGCCACGACCAGTCTAGAGGACATAGACT
PI 486271 CGTCTCGATCATAAGAATATAGTAAAGGTTGCCAGCTACTGCCACGACCAGTCTAGAGGACATAGACT
PI 511367 CGTCTCGATCATAAGAATATAGTAAAGGTTGCCAGCTACTGCCACGACCAGTCTAGAGGACATAGACT
Chinese Spring CGTCTCGATCATAAGAATATAGTAAAGGTTGCCAGCTACTGCCACGACCAGTCTACAGGACATAGACT
Hanzhongbai CGTCTCGATCATAAGAATATAGTAAAGGTTGCCAGCTACTGCCACGACCAGTCTACAGGACATAGACT
Hongtoumai CGTCTCGATCATAAGAATATAGTAAAGGTTGCCAGCTACTGCCACGACCAGTCTACAGGACATAGACT
Hongyouzi CGTCTCGATCATAAGAATATAGTAAAGGTTGCCAGCTACTGCCACGACCAGTCTACAGGACATAGACT
Zijiehong CGTCTCGATCATAAGAATATAGTAAAGGTTGCCAGCTACTGCCACGACCAGTCTACAGGACATAGACT
Baihuamai CGTCTCGATCATAAGAATATAGTAAAGGTTGCCAGCTACTGCCACGACCAGTCTACAGGACATAGACT
Huixianhong CGTCTCGATCATAAGAATATAGTAAAGGTTGCCAGCTACTGCCACGACCAGTCTACAGGACATAGACT
Baimangmai CGTCTCGATCATAAGAATATAGTAAAGGTTGCCAGCTACTGCCACGACCAGTCTACAGGACATAGACT

	7,350 7,360 7,370 7,380 7,390 7,400 7,410
Hulutou	GGTACAGTTCAAAGGAAAACCGCTACCACAACTCTTTAACGGTCCCGAACAACTGCTCTGCTATGAAT
Baihulu	GGTACAGTTCAAAGGAAAACCGCTACCACAACTCTTTAACGGTCCCGAACAACTGCTCTGCTATGAAT
Chiyacao	GGTACAGTTCAAAGGAAAACCGCTACCACAACTCTTTAACGGTCCCGAACAACTGCTCTGCTATGAAT
Hongmangmai	GGTACAGTTCAAAGGAAAACCGCTACCACAACTCTTTAACGGTCCCGAACAACTGCTCTGCTATGAAT
Hongmai	GGTACAGTTCAAAGGAAAACCGCTACCACAACTCTTTAACGGTCCCGAACAACTGCTCTGCTATGAAT
Wangshuibai	GGTACAGTTCAAAGGAAAACCGCTACCACAACTCTTTAACGGTCCCGAACAACTGCTCTGCTATGAAT
Shi 4185	GGTACAGTTCAAAGGAAAACCGCTACCACAACTCTTTAACGGTCCCGAACAACTGCTCTGCTATGAAT
Mazhamai	GGTACAGTTCAAAGGAAAACCGCTACCACAACTCTTTAACGGTCCCGAACAACTGCTCTGCTATGAAT
Xiaofoshou	GGTACAGTTCAAAGGAAAACCGCTACCACAACTCTTTAACGGTCCCGAACAACTGCTCTGCTATGAAT
Aikang 58	GGTACAGTTCAAAGGAAAACCGCTACCACAACTCTTTAACGGTCCCGAACAACTGCTCTGCTATGAAT
AL8/78	GGTACAGTTCAAAGGAAAACCGCTACCACAACTCTTTAACGGTCCCGAACAACTGCTCTGCTATGAAT
PI 431602	GGTACAGTTCAAAGGAAAACCGCTACCACAACTCTTTAACGGTCCCGAACAACTGCTCTGCTATGAAT
PI 486274	GGTACAGTTCAAAGGAAAACCGCTACCACAACTCTTTAACGGTCCCGAACAACTGCTCTGCTATGAAT
RM 000182	GGTACAGTTCAAAGGAAAACCGCTACCACAACTCTTTAACGGTCCCGAACAACTGCTCTGCTATGAAT
PI 511381	GGTACAGTTCAAAGGAAAACCGCTACCACAACTCTTTAACGGTCCCGAACAACTGCTCTGCTATGAAT
PI 486271	GGTACAGTTCAAAGGAAAACCGCTACCACAACTCTTTAACGGTCCCGAACAACTGCTCTGCTATGAAT
PI 511367	GGTACAGTTCAAAGGAAAACCGCTACCACAACTCTTTAACGGTCCCGAACAACTGCTCTGCTATGAAT
Chinese Spring	GGTACAGTTCAAAGGAAAACCGCTACCACAACTCTTTAACGGTCCCGAACAACTGCTCTGCTATGAAT
Hanzhongbai	GGTACAGTTCAAAGGAAAACCGCTACCACAACTCTTTAACGGTCCCGAACAACTGCTCTGCTATGAAT
Hongtoumai	GGTACAGTTCAAAGGAAAACCGCTACCACAACTCTTTAACGGTCCCGAACAACTGCTCTGCTATGAAT
Hongyouzi	GGTACAGTTCAAAGGAAAACCGCTACCACAACTCTTTAACGGTCCCGAACAACTGCTCTGCTATGAAT
Zijiehong	GGTACAGTTCAAAGGAAAACCGCTACCACAACTCTTTAACGGTCCCGAACAACTGCTCTGCTATGAAT
Baihuamai	GGTACAGTTCAAAGGAAAACCGCTACCACAACTCTTTAACGGTCCCGAACAACTGCTCTGCTATGAAT
Huixianhong	GGTACAGTTCAAAGGAAAACCGCTACCACAACTCTTTAACGGTCCCGAACAACTGCTCTGCTATGAAT
Baimangmai	GGTACAGTTCAAAGGAAAACCGCTACCACAACTCTTTAACGGTCCCGAACAACTGCTCTGCTATGAAT

	7,420 7,430 7,440 7,450 7,460 7,470 7,480
Hulutou	ATATGCACAACGGAAGCCTTCGCGACTATCTTATGGGTAATGATACCGATCACTAATCAACTACTGTT
Baihulu	ATATGCACAACGGAAGCCTTCGCGACTATCTTATGGGTAATGATACCGATCACTAATCAACTACTGTT
Chiyacao	ATATGCACAACGGAAGCCTTCGCGACTATCTTATGGGTAATGATACCGATCACTAATCAACTACTGTT
Hongmangmai	ATATGCACAACGGAAGCCTTCGCGACTATCTTATGGGTAATGATACCGATCACTAATCAACTACTGTT
Hongmai	ATATGCACAACGGAAGCCTTCGCGACTATCTTATGGGTAATGATACCGATCACTAATCAACTACTGTT
Wangshuibai	ATATGCACAACGGAAGCCTTCGCGACTATCTTATGGGTAATGATACCGATCACTAATCAACTACTGTT
Shi 4185	ATATGCACAACGGAAGCCTTCGCGACTATCTTATGGGTAATGATACCGATCACTAATCAACTACTGTT
Mazhamai	ATATGCACAACGGAAGCCTTCGCGACTATCTTATGGGTAATGATACCGATCACTAATCAACTACTGTT
Xiaofoshou	ATATGCACAACGGAAGCCTTCGCGACTATCTTATGGGTAATGATACCGATCACTAATCAACTACTGTT
Aikang 58	ATATGCACAACGGAAGCCTTCGCGACTATCTTATGGGTAATGATACCGATCACTAATCAACTACTGTT
AL8/78	ATATGCACAACGGAAGCCTTCGCGACTATCTTATGGGTAATGATACCGATCACTAATCAACTACTGTT
PI 431602	ATATGCACAACGGAAGCCTTCGCGACTATCTTATGGGTAATGATACCGATCACTAATCAACTACTGTT
PI 486274	ATATGCACAACGGAAGCCTTCGCGACTATCTTATGGGTAATGATACCGATCACTAATCAACTACTGTT
RM 000182	ATATGCACAACGGAAGCCTTCGCGACTATCTTATGGGTAATGATACCGATCACTAATCAACTACTGTT
PI 511381	ATATGCACAACGGAAGCCTTCGCGACTATCTTATGGGTAATGATACCGATCACTAATCAACTACTGTT
PI 486271	ATATGCACAACGGAAGCCTTCGCGACTATCTTATGGGTAATGATACCGATCACTAATCAACTACTGTT
PI 511367	ATATGCACAACGGAAGCCTTCGCGACTATCTTATGGGTAATGATACCGATCACTAATCAACTACTGTT
Chinese Spring	ATATGCACAACGGAAGCCTTCGCGACTATCTTATGGGTAATGATACCGATCACTAATCAACTACTGTT
Hanzhongbai	ATATGCACAACGGAAGCCTTCGCGACTATCTTATGGGTAATGATACCGATCACTAATCAACTACTGTT
Hongtoumai	ATATGCACAACGGAAGCCTTCGCGACTATCTTATGGGTAATGATACCGATCACTAATCAACTACTGTT
Hongyouzi	ATATGCACAACGGAAGCCTTCGCGACTATCTTATGGGTAATGATACCGATCACTAATCAACTACTGTT
Zijiehong	ATATGCACAACGGAAGCCTTCGCGACTATCTTATGGGTAATGATACCGATCACTAATCAACTACTGTT
Baihuamai	ATATGCACAACGGAAGCCTTCGCGACTATCTTATGGGTAATGATACCGATCACTAATCAACTACTGTT
Huixianhong	ATATGCACAACGGAAGCCTTCGCGACTATCTTATGGGTAATGATACCGATCACTAATCAACTACTGTT
Baimangmai	ATATGCACAACGGAAGCCTTCGCGACTATCTTATGGGTAATGATACCGATCACTAATCAACTACTGTT

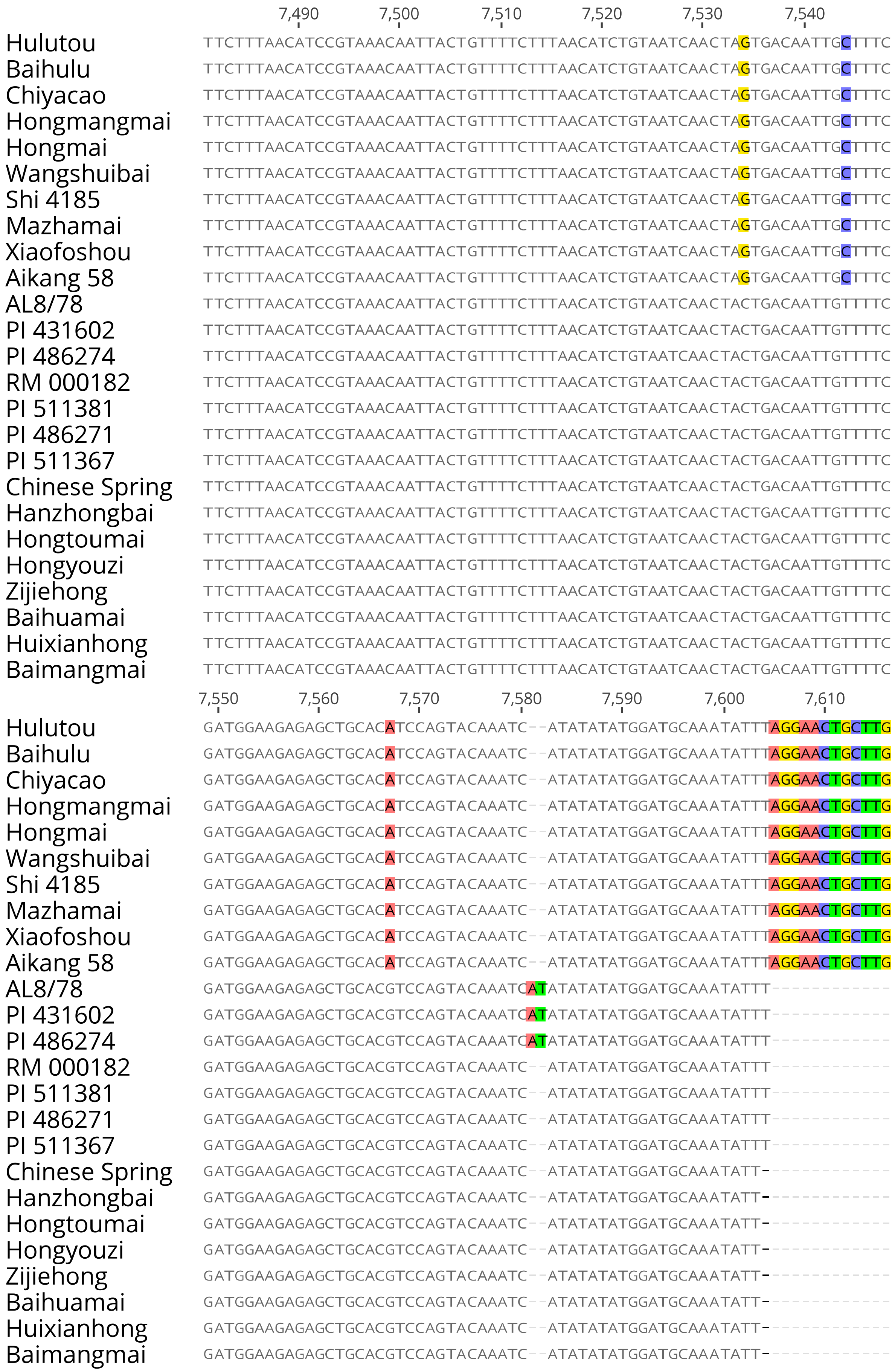

7,490 7,500 7,510 7,520 7,530 7,540
Hulutou TTCTTTAACATCCGTAAACAATTACTGTTTTCTTTAACATCTGTAATCAACTAGTGACAATTGCTTTC
Baihulu TTCTTTAACATCCGTAAACAATTACTGTTTTCTTTAACATCTGTAATCAACTAGTGACAATTGCTTTC
Chiyacao TTCTTTAACATCCGTAAACAATTACTGTTTTCTTTAACATCTGTAATCAACTAGTGACAATTGCTTTC
Hongmangmai TTCTTTAACATCCGTAAACAATTACTGTTTTCTTTAACATCTGTAATCAACTAGTGACAATTGCTTTC
Hongmai TTCTTTAACATCCGTAAACAATTACTGTTTTCTTTAACATCTGTAATCAACTAGTGACAATTGCTTTC
Wangshuibai TTCTTTAACATCCGTAAACAATTACTGTTTTCTTTAACATCTGTAATCAACTAGTGACAATTGCTTTC
Shi 4185 TTCTTTAACATCCGTAAACAATTACTGTTTTCTTTAACATCTGTAATCAACTAGTGACAATTGCTTTC
Mazhamai TTCTTTAACATCCGTAAACAATTACTGTTTTCTTTAACATCTGTAATCAACTAGTGACAATTGCTTTC
Xiaofoshou TTCTTTAACATCCGTAAACAATTACTGTTTTCTTTAACATCTGTAATCAACTAGTGACAATTGCTTTC
Aikang 58 TTCTTTAACATCCGTAAACAATTACTGTTTTCTTTAACATCTGTAATCAACTAGTGACAATTGCTTTC
AL8/78 TTCTTTAACATCCGTAAACAATTACTGTTTTCTTTAACATCTGTAATCAACTACTGACAATTGTTTTC
PI 431602 TTCTTTAACATCCGTAAACAATTACTGTTTTCTTTAACATCTGTAATCAACTACTGACAATTGTTTTC
PI 486274 TTCTTTAACATCCGTAAACAATTACTGTTTTCTTTAACATCTGTAATCAACTACTGACAATTGTTTTC
RM 000182 TTCTTTAACATCCGTAAACAATTACTGTTTTCTTTAACATCTGTAATCAACTACTGACAATTGTTTTC
PI 511381 TTCTTTAACATCCGTAAACAATTACTGTTTTCTTTAACATCTGTAATCAACTACTGACAATTGTTTTC
PI 486271 TTCTTTAACATCCGTAAACAATTACTGTTTTCTTTAACATCTGTAATCAACTACTGACAATTGTTTTC
PI 511367 TTCTTTAACATCCGTAAACAATTACTGTTTTCTTTAACATCTGTAATCAACTACTGACAATTGTTTTC
Chinese Spring TTCTTTAACATCCGTAAACAATTACTGTTTTCTTTAACATCTGTAATCAACTACTGACAATTGTTTTC
Hanzhongbai TTCTTTAACATCCGTAAACAATTACTGTTTTCTTTAACATCTGTAATCAACTACTGACAATTGTTTTC
Hongtoumai TTCTTTAACATCCGTAAACAATTACTGTTTTCTTTAACATCTGTAATCAACTACTGACAATTGTTTTC
Hongyouzi TTCTTTAACATCCGTAAACAATTACTGTTTTCTTTAACATCTGTAATCAACTACTGACAATTGTTTTC
Zijiehong TTCTTTAACATCCGTAAACAATTACTGTTTTCTTTAACATCTGTAATCAACTACTGACAATTGTTTTC
Baihuamai TTCTTTAACATCCGTAAACAATTACTGTTTTCTTTAACATCTGTAATCAACTACTGACAATTGTTTTC
Huixianhong TTCTTTAACATCCGTAAACAATTACTGTTTTCTTTAACATCTGTAATCAACTACTGACAATTGTTTTC
Baimangmai TTCTTTAACATCCGTAAACAATTACTGTTTTCTTTAACATCTGTAATCAACTACTGACAATTGTTTTC
7,550 7,560 7,570 7,580 7,590 7,600 7,610
Hulutou GATGGAAGAGAGCTGCACATCCAGTACAAATC--ATATATATGGATGCAAATATTTAGGAACTGCTTG
Baihulu GATGGAAGAGAGCTGCACATCCAGTACAAATC--ATATATATGGATGCAAATATTTAGGAACTGCTTG
Chiyacao GATGGAAGAGAGCTGCACATCCAGTACAAATC--ATATATATGGATGCAAATATTTAGGAACTGCTTG
Hongmangmai GATGGAAGAGAGCTGCACATCCAGTACAAATC--ATATATATGGATGCAAATATTTAGGAACTGCTTG
Hongmai GATGGAAGAGAGCTGCACATCCAGTACAAATC--ATATATATGGATGCAAATATTTAGGAACTGCTTG
Wangshuibai GATGGAAGAGAGCTGCACATCCAGTACAAATC--ATATATATGGATGCAAATATTTAGGAACTGCTTG
Shi 4185 GATGGAAGAGAGCTGCACATCCAGTACAAATC--ATATATATGGATGCAAATATTTAGGAACTGCTTG
Mazhamai GATGGAAGAGAGCTGCACATCCAGTACAAATC--ATATATATGGATGCAAATATTTAGGAACTGCTTG
Xiaofoshou GATGGAAGAGAGCTGCACATCCAGTACAAATC--ATATATATGGATGCAAATATTTAGGAACTGCTTG
Aikang 58 GATGGAAGAGAGCTGCACATCCAGTACAAATC--ATATATATGGATGCAAATATTTAGGAACTGCTTG
AL8/78 GATGGAAGAGAGCTGCACGTCCAGTACAAATCATATATATATGGATGCAAATATTT------------
PI 431602 GATGGAAGAGAGCTGCACGTCCAGTACAAATCATATATATATGGATGCAAATATTT------------
PI 486274 GATGGAAGAGAGCTGCACGTCCAGTACAAATCATATATATATGGATGCAAATATTT------------
RM 000182 GATGGAAGAGAGCTGCACGTCCAGTACAAATC--ATATATATGGATGCAAATATTT------------
PI 511381 GATGGAAGAGAGCTGCACGTCCAGTACAAATC--ATATATATGGATGCAAATATTT------------
PI 486271 GATGGAAGAGAGCTGCACGTCCAGTACAAATC--ATATATATGGATGCAAATATTT------------
PI 511367 GATGGAAGAGAGCTGCACGTCCAGTACAAATC--ATATATATGGATGCAAATATTT------------
Chinese Spring GATGGAAGAGAGCTGCACGTCCAGTACAAATC--ATATATATGGATGCAAATATT-------------
Hanzhongbai GATGGAAGAGAGCTGCACGTCCAGTACAAATC--ATATATATGGATGCAAATATT-------------
Hongtoumai GATGGAAGAGAGCTGCACGTCCAGTACAAATC--ATATATATGGATGCAAATATT-------------
Hongyouzi GATGGAAGAGAGCTGCACGTCCAGTACAAATC--ATATATATGGATGCAAATATT-------------
Zijiehong GATGGAAGAGAGCTGCACGTCCAGTACAAATC--ATATATATGGATGCAAATATT-------------
Baihuamai GATGGAAGAGAGCTGCACGTCCAGTACAAATC--ATATATATGGATGCAAATATT-------------
Huixianhong GATGGAAGAGAGCTGCACGTCCAGTACAAATC--ATATATATGGATGCAAATATT-------------
Baimangmai GATGGAAGAGAGCTGCACGTCCAGTACAAATC--ATATATATGGATGCAAATATT-------------

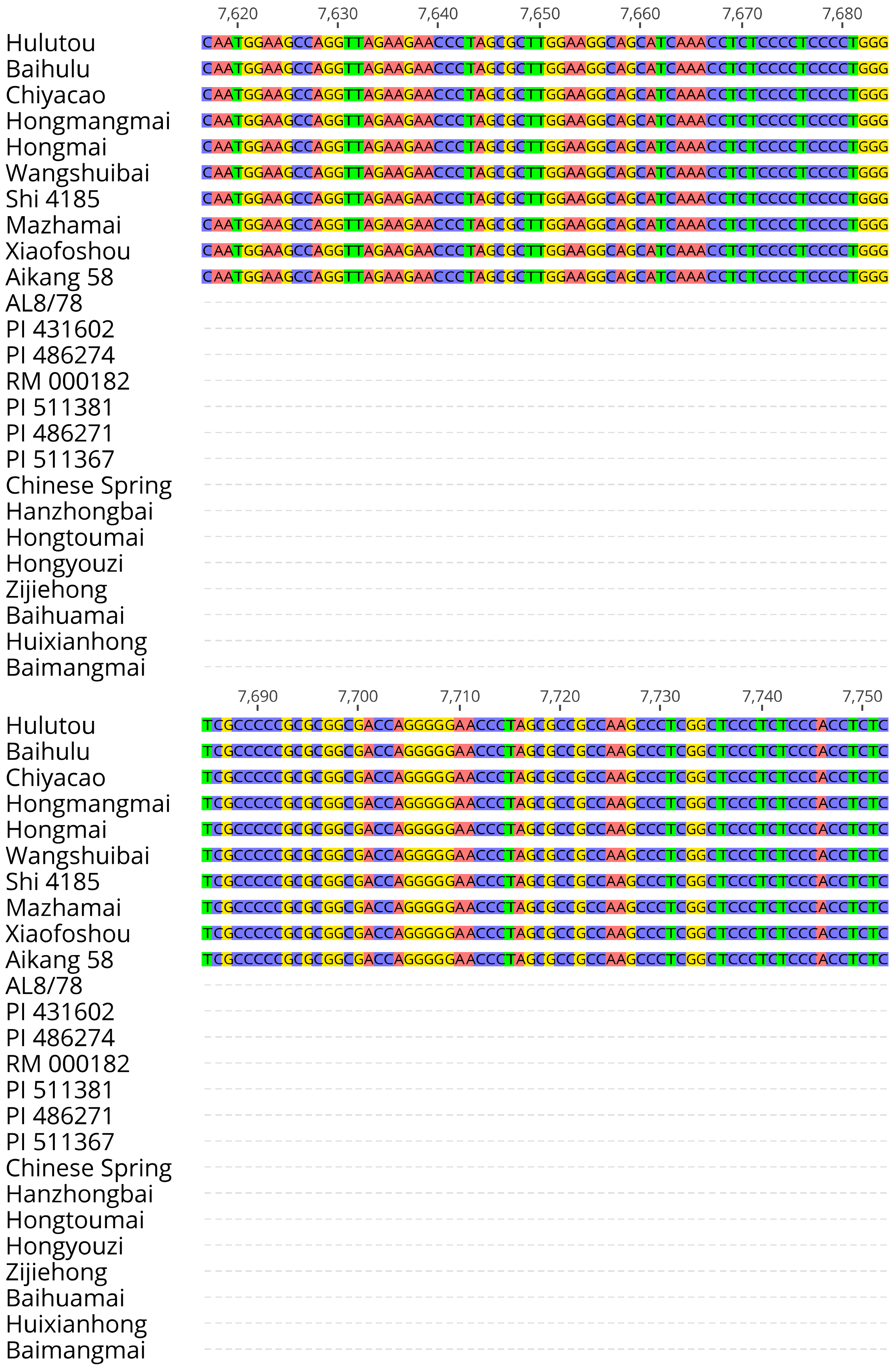
7,620 7,630 7,640 7,650 7,660 7,670 7,680
Hulutou CAATGGAAGCCAGGTTAGAAGAACCCTAGCGCTTGGAAGGCAGCATCAAACCTCTCCCCTCCCCTGGG
Baihulu CAATGGAAGCCAGGTTAGAAGAACCCTAGCGCTTGGAAGGCAGCATCAAACCTCTCCCCTCCCCTGGG
Chiyacao CAATGGAAGCCAGGTTAGAAGAACCCTAGCGCTTGGAAGGCAGCATCAAACCTCTCCCCTCCCCTGGG
Hongmangmai CAATGGAAGCCAGGTTAGAAGAACCCTAGCGCTTGGAAGGCAGCATCAAACCTCTCCCCTCCCCTGGG
Hongmai CAATGGAAGCCAGGTTAGAAGAACCCTAGCGCTTGGAAGGCAGCATCAAACCTCTCCCCTCCCCTGGG
Wangshuibai CAATGGAAGCCAGGTTAGAAGAACCCTAGCGCTTGGAAGGCAGCATCAAACCTCTCCCCTCCCCTGGG
Shi 4185 CAATGGAAGCCAGGTTAGAAGAACCCTAGCGCTTGGAAGGCAGCATCAAACCTCTCCCCTCCCCTGGG
Mazhamai CAATGGAAGCCAGGTTAGAAGAACCCTAGCGCTTGGAAGGCAGCATCAAACCTCTCCCCTCCCCTGGG
Xiaofoshou CAATGGAAGCCAGGTTAGAAGAACCCTAGCGCTTGGAAGGCAGCATCAAACCTCTCCCCTCCCCTGGG
Aikang 58 CAATGGAAGCCAGGTTAGAAGAACCCTAGCGCTTGGAAGGCAGCATCAAACCTCTCCCCTCCCCTGGG
AL8/78
PI 431602
PI 486274
RM 000182
PI 511381
PI 486271
PI 511367
Chinese Spring
Hanzhongbai
Hongtoumai
Hongyouzi
Zijiehong
Baihuamai
Huixianhong
Baimangmai
7,690 7,700 7,710 7,720 7,730 7,740 7,750
Hulutou TCGCCCCCGCGCGGCGACCAGGGGGAACCCTAGCGCCGCCAAGCCCTCGGCTCCCTCTCCCACCTCTC
Baihulu TCGCCCCCGCGCGGCGACCAGGGGGAACCCTAGCGCCGCCAAGCCCTCGGCTCCCTCTCCCACCTCTC
Chiyacao TCGCCCCCGCGCGGCGACCAGGGGGAACCCTAGCGCCGCCAAGCCCTCGGCTCCCTCTCCCACCTCTC
Hongmangmai TCGCCCCCGCGCGGCGACCAGGGGGAACCCTAGCGCCGCCAAGCCCTCGGCTCCCTCTCCCACCTCTC
Hongmai TCGCCCCCGCGCGGCGACCAGGGGGAACCCTAGCGCCGCCAAGCCCTCGGCTCCCTCTCCCACCTCTC
Wangshuibai TCGCCCCCGCGCGGCGACCAGGGGGAACCCTAGCGCCGCCAAGCCCTCGGCTCCCTCTCCCACCTCTC
Shi 4185 TCGCCCCCGCGCGGCGACCAGGGGGAACCCTAGCGCCGCCAAGCCCTCGGCTCCCTCTCCCACCTCTC
Mazhamai TCGCCCCCGCGCGGCGACCAGGGGGAACCCTAGCGCCGCCAAGCCCTCGGCTCCCTCTCCCACCTCTC
Xiaofoshou TCGCCCCCGCGCGGCGACCAGGGGGAACCCTAGCGCCGCCAAGCCCTCGGCTCCCTCTCCCACCTCTC
Aikang 58 TCGCCCCCGCGCGGCGACCAGGGGGAACCCTAGCGCCGCCAAGCCCTCGGCTCCCTCTCCCACCTCTC
AL8/78
PI 431602
PI 486274
RM 000182
PI 511381
PI 486271
PI 511367
Chinese Spring
Hanzhongbai
Hongtoumai
Hongyouzi
Zijiehong
Baihuamai
Huixianhong
Baimangmai

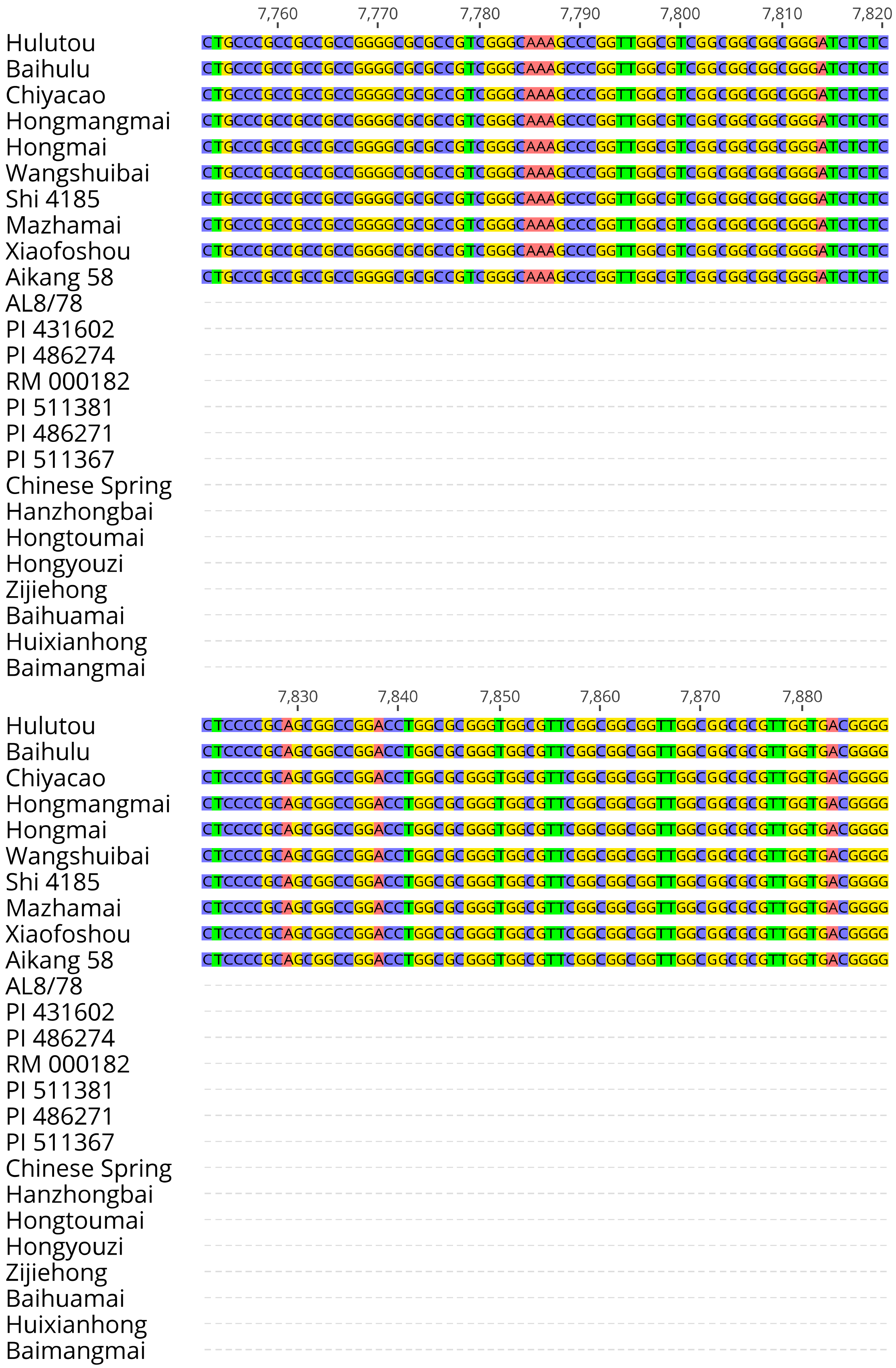
7,760 7,770 7,780 7,790 7,800 7,810 7,820
Hulutou CTGCCCGCCGCCGCCGGGGCGCGCCGTCGGGCAAAGCCCGGTTGGCGTCGGCGGCGGCGGGATCTCTC
Baihulu CTGCCCGCCGCCGCCGGGGCGCGCCGTCGGGCAAAGCCCGGTTGGCGTCGGCGGCGGCGGGATCTCTC
Chiyacao CTGCCCGCCGCCGCCGGGGCGCGCCGTCGGGCAAAGCCCGGTTGGCGTCGGCGGCGGCGGGATCTCTC
Hongmangmai CTGCCCGCCGCCGCCGGGGCGCGCCGTCGGGCAAAGCCCGGTTGGCGTCGGCGGCGGCGGGATCTCTC
Hongmai CTGCCCGCCGCCGCCGGGGCGCGCCGTCGGGCAAAGCCCGGTTGGCGTCGGCGGCGGCGGGATCTCTC
Wangshuibai CTGCCCGCCGCCGCCGGGGCGCGCCGTCGGGCAAAGCCCGGTTGGCGTCGGCGGCGGCGGGATCTCTC
Shi 4185 CTGCCCGCCGCCGCCGGGGCGCGCCGTCGGGCAAAGCCCGGTTGGCGTCGGCGGCGGCGGGATCTCTC
Mazhamai CTGCCCGCCGCCGCCGGGGCGCGCCGTCGGGCAAAGCCCGGTTGGCGTCGGCGGCGGCGGGATCTCTC
Xiaofoshou CTGCCCGCCGCCGCCGGGGCGCGCCGTCGGGCAAAGCCCGGTTGGCGTCGGCGGCGGCGGGATCTCTC
Aikang 58 CTGCCCGCCGCCGCCGGGGCGCGCCGTCGGGCAAAGCCCGGTTGGCGTCGGCGGCGGCGGGATCTCTC
AL8/78
PI 431602
PI 486274
RM 000182
PI 511381
PI 486271
PI 511367
Chinese Spring
Hanzhongbai
Hongtoumai
Hongyouzi
Zijiehong
Baihuamai
Huixianhong
Baimangmai
7,830 7,840 7,850 7,860 7,870 7,880
Hulutou CTCCCCGCAGCGGCCGGACCTGGCGCGGGTGGCGTTCGGCGGCGGTTGGCGGCGCGTTGGTGACGGGG
Baihulu CTCCCCGCAGCGGCCGGACCTGGCGCGGGTGGCGTTCGGCGGCGGTTGGCGGCGCGTTGGTGACGGGG
Chiyacao CTCCCCGCAGCGGCCGGACCTGGCGCGGGTGGCGTTCGGCGGCGGTTGGCGGCGCGTTGGTGACGGGG
Hongmangmai CTCCCCGCAGCGGCCGGACCTGGCGCGGGTGGCGTTCGGCGGCGGTTGGCGGCGCGTTGGTGACGGGG
Hongmai CTCCCCGCAGCGGCCGGACCTGGCGCGGGTGGCGTTCGGCGGCGGTTGGCGGCGCGTTGGTGACGGGG
Wangshuibai CTCCCCGCAGCGGCCGGACCTGGCGCGGGTGGCGTTCGGCGGCGGTTGGCGGCGCGTTGGTGACGGGG
Shi 4185 CTCCCCGCAGCGGCCGGACCTGGCGCGGGTGGCGTTCGGCGGCGGTTGGCGGCGCGTTGGTGACGGGG
Mazhamai CTCCCCGCAGCGGCCGGACCTGGCGCGGGTGGCGTTCGGCGGCGGTTGGCGGCGCGTTGGTGACGGGG
Xiaofoshou CTCCCCGCAGCGGCCGGACCTGGCGCGGGTGGCGTTCGGCGGCGGTTGGCGGCGCGTTGGTGACGGGG
Aikang 58 CTCCCCGCAGCGGCCGGACCTGGCGCGGGTGGCGTTCGGCGGCGGTTGGCGGCGCGTTGGTGACGGGG
AL8/78
PI 431602
PI 486274
RM 000182
PI 511381
PI 486271
PI 511367
Chinese Spring
Hanzhongbai
Hongtoumai
Hongyouzi
Zijiehong
Baihuamai
Huixianhong
Baimangmai

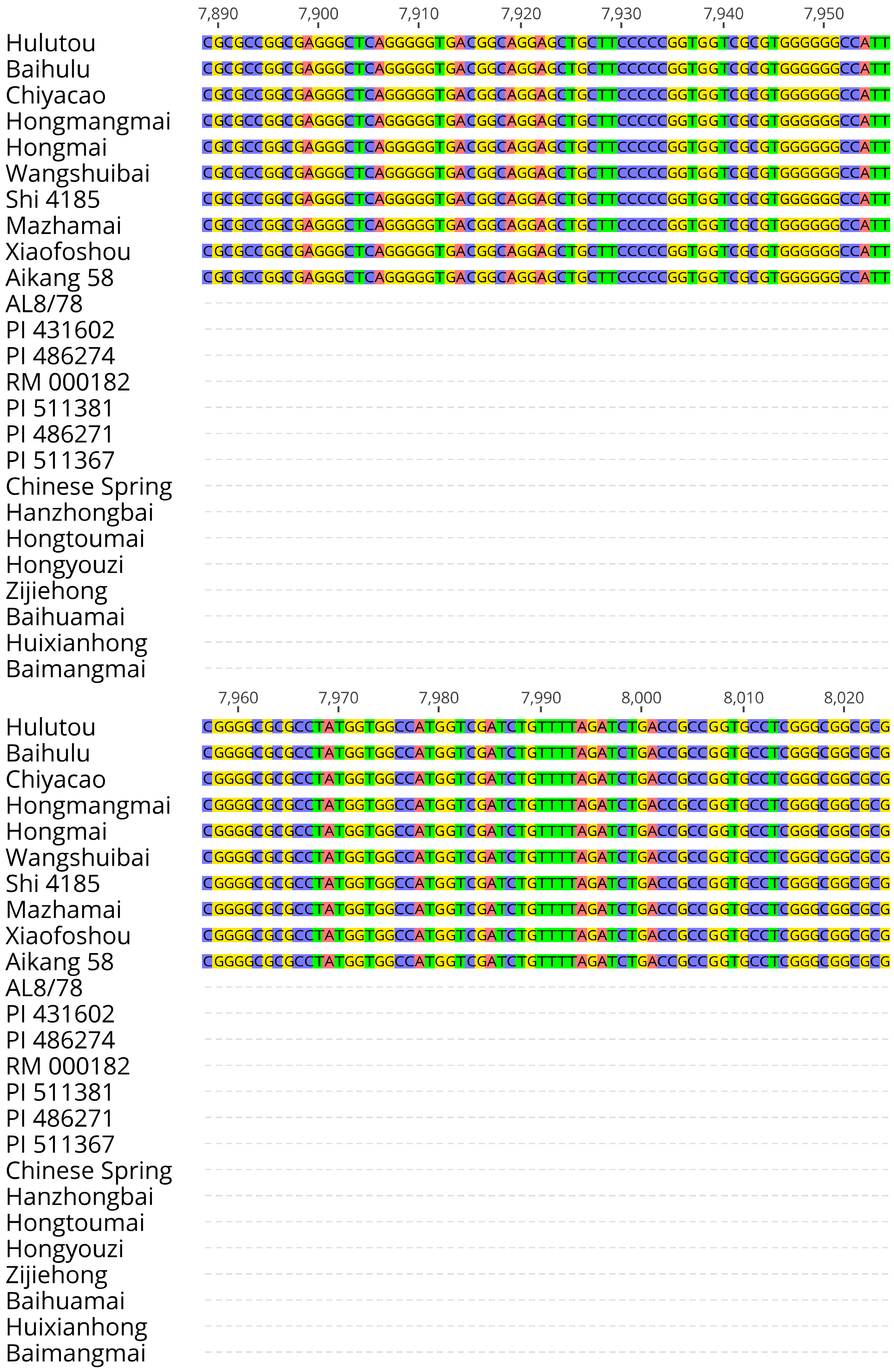
7,890 7,900 7,910 7,920 7,930 7,940 7,950
Hulutou CGCGCCGGCGAGGGCTCAGGGGGTGACGGCAGGAGCTGCTTCCCCCGGTGGTCGCGTGGGGGGCCATT
Baihulu CGCGCCGGCGAGGGCTCAGGGGGTGACGGCAGGAGCTGCTTCCCCCGGTGGTCGCGTGGGGGGCCATT
Chiyacao CGCGCCGGCGAGGGCTCAGGGGGTGACGGCAGGAGCTGCTTCCCCCGGTGGTCGCGTGGGGGGCCATT
Hongmangmai CGCGCCGGCGAGGGCTCAGGGGGTGACGGCAGGAGCTGCTTCCCCCGGTGGTCGCGTGGGGGGCCATT
Hongmai CGCGCCGGCGAGGGCTCAGGGGGTGACGGCAGGAGCTGCTTCCCCCGGTGGTCGCGTGGGGGGCCATT
Wangshuibai CGCGCCGGCGAGGGCTCAGGGGGTGACGGCAGGAGCTGCTTCCCCCGGTGGTCGCGTGGGGGGCCATT
Shi 4185 CGCGCCGGCGAGGGCTCAGGGGGTGACGGCAGGAGCTGCTTCCCCCGGTGGTCGCGTGGGGGGCCATT
Mazhamai CGCGCCGGCGAGGGCTCAGGGGGTGACGGCAGGAGCTGCTTCCCCCGGTGGTCGCGTGGGGGGCCATT
Xiaofoshou CGCGCCGGCGAGGGCTCAGGGGGTGACGGCAGGAGCTGCTTCCCCCGGTGGTCGCGTGGGGGGCCATT
Aikang 58 CGCGCCGGCGAGGGCTCAGGGGGTGACGGCAGGAGCTGCTTCCCCCGGTGGTCGCGTGGGGGGCCATT
AL8/78
PI 431602
PI 486274
RM 000182
PI 511381
PI 486271
PI 511367
Chinese Spring
Hanzhongbai
Hongtoumai
Hongyouzi
Zijiehong
Baihuamai
Huixianhong
Baimangmai
7,960 7,970 7,980 7,990 8,000 8,010 8,020
Hulutou CGGGGCGCGCCTATGGTGGCCATGGTCGATCTGTTTTAGATCTGACCGCCGGTGCCTCGGGCGGCGCG
Baihulu CGGGGCGCGCCTATGGTGGCCATGGTCGATCTGTTTTAGATCTGACCGCCGGTGCCTCGGGCGGCGCG
Chiyacao CGGGGCGCGCCTATGGTGGCCATGGTCGATCTGTTTTAGATCTGACCGCCGGTGCCTCGGGCGGCGCG
Hongmangmai CGGGGCGCGCCTATGGTGGCCATGGTCGATCTGTTTTAGATCTGACCGCCGGTGCCTCGGGCGGCGCG
Hongmai CGGGGCGCGCCTATGGTGGCCATGGTCGATCTGTTTTAGATCTGACCGCCGGTGCCTCGGGCGGCGCG
Wangshuibai CGGGGCGCGCCTATGGTGGCCATGGTCGATCTGTTTTAGATCTGACCGCCGGTGCCTCGGGCGGCGCG
Shi 4185 CGGGGCGCGCCTATGGTGGCCATGGTCGATCTGTTTTAGATCTGACCGCCGGTGCCTCGGGCGGCGCG
Mazhamai CGGGGCGCGCCTATGGTGGCCATGGTCGATCTGTTTTAGATCTGACCGCCGGTGCCTCGGGCGGCGCG
Xiaofoshou CGGGGCGCGCCTATGGTGGCCATGGTCGATCTGTTTTAGATCTGACCGCCGGTGCCTCGGGCGGCGCG
Aikang 58 CGGGGCGCGCCTATGGTGGCCATGGTCGATCTGTTTTAGATCTGACCGCCGGTGCCTCGGGCGGCGCG
AL8/78
PI 431602
PI 486274
RM 000182
PI 511381
PI 486271
PI 511367
Chinese Spring
Hanzhongbai
Hongtoumai
Hongyouzi
Zijiehong
Baihuamai
Huixianhong
Baimangmai

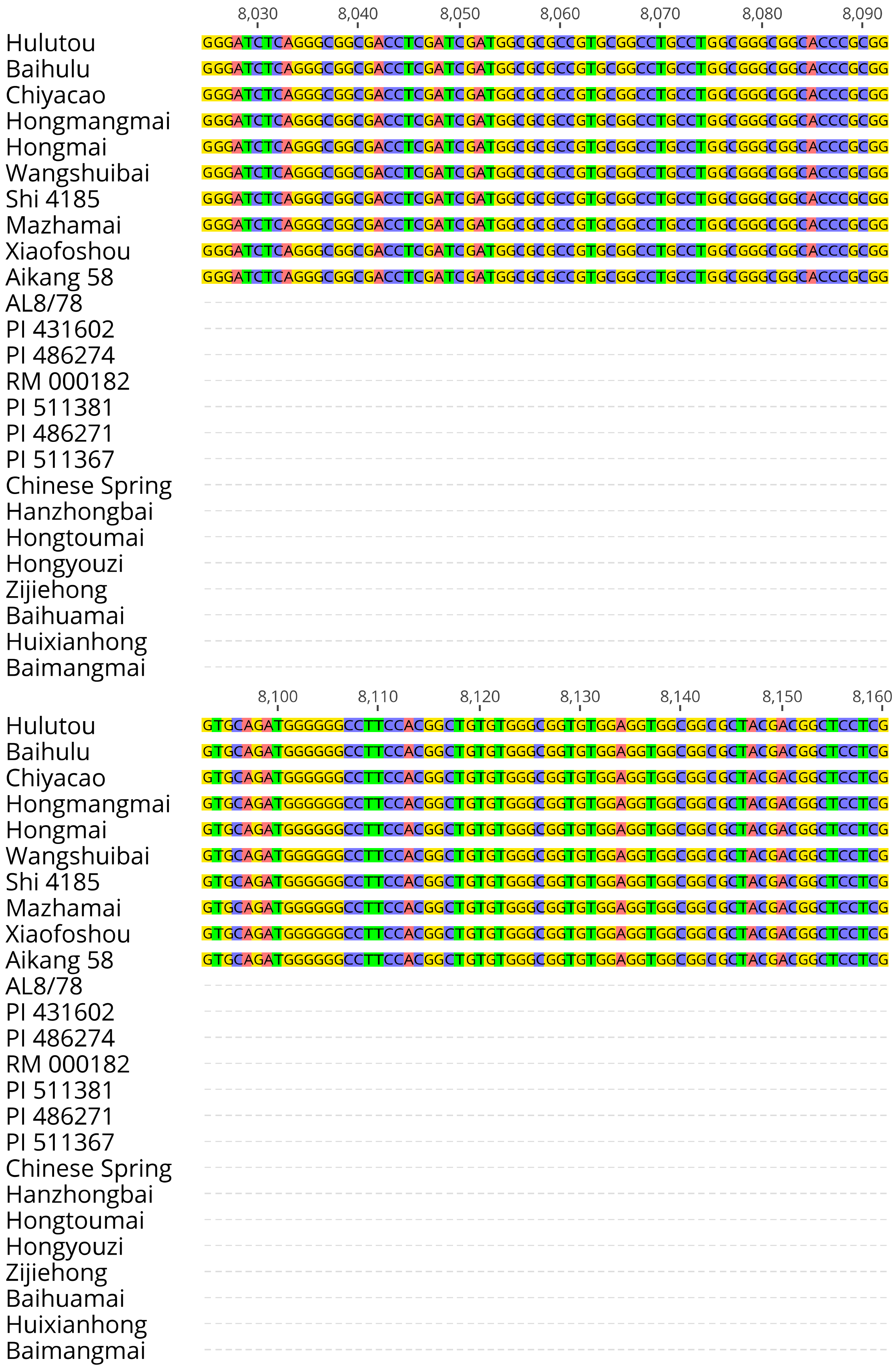

8,030 8,040 8,050 8,060 8,070 8,080 8,090
Hulutou GGGATCTCAGGGCGGCGACCTCGATCGATGGCGCGCCGTGCGGCCTGCCTGGCGGGCGGCACCCGCGG
Baihulu GGGATCTCAGGGCGGCGACCTCGATCGATGGCGCGCCGTGCGGCCTGCCTGGCGGGCGGCACCCGCGG
Chiyacao GGGATCTCAGGGCGGCGACCTCGATCGATGGCGCGCCGTGCGGCCTGCCTGGCGGGCGGCACCCGCGG
Hongmangmai GGGATCTCAGGGCGGCGACCTCGATCGATGGCGCGCCGTGCGGCCTGCCTGGCGGGCGGCACCCGCGG
Hongmai GGGATCTCAGGGCGGCGACCTCGATCGATGGCGCGCCGTGCGGCCTGCCTGGCGGGCGGCACCCGCGG
Wangshuibai GGGATCTCAGGGCGGCGACCTCGATCGATGGCGCGCCGTGCGGCCTGCCTGGCGGGCGGCACCCGCGG
Shi 4185 GGGATCTCAGGGCGGCGACCTCGATCGATGGCGCGCCGTGCGGCCTGCCTGGCGGGCGGCACCCGCGG
Mazhamai GGGATCTCAGGGCGGCGACCTCGATCGATGGCGCGCCGTGCGGCCTGCCTGGCGGGCGGCACCCGCGG
Xiaofoshou GGGATCTCAGGGCGGCGACCTCGATCGATGGCGCGCCGTGCGGCCTGCCTGGCGGGCGGCACCCGCGG
Aikang 58 GGGATCTCAGGGCGGCGACCTCGATCGATGGCGCGCCGTGCGGCCTGCCTGGCGGGCGGCACCCGCGG
AL8/78
PI 431602
PI 486274
RM 000182
PI 511381
PI 486271
PI 511367
Chinese Spring
Hanzhongbai
Hongtoumai
Hongyouzi
Zijiehong
Baihuamai
Huixianhong
Baimangmai
8,100 8,110 8,120 8,130 8,140 8,150 8,160
Hulutou GTGCAGATGGGGGGCCTTCCACGGCTGTGTGGGCGGTGTGGAGGTGGCGGCGCTACGACGGCTCCTCG
Baihulu GTGCAGATGGGGGGCCTTCCACGGCTGTGTGGGCGGTGTGGAGGTGGCGGCGCTACGACGGCTCCTCG
Chiyacao GTGCAGATGGGGGGCCTTCCACGGCTGTGTGGGCGGTGTGGAGGTGGCGGCGCTACGACGGCTCCTCG
Hongmangmai GTGCAGATGGGGGGCCTTCCACGGCTGTGTGGGCGGTGTGGAGGTGGCGGCGCTACGACGGCTCCTCG
Hongmai GTGCAGATGGGGGGCCTTCCACGGCTGTGTGGGCGGTGTGGAGGTGGCGGCGCTACGACGGCTCCTCG
Wangshuibai GTGCAGATGGGGGGCCTTCCACGGCTGTGTGGGCGGTGTGGAGGTGGCGGCGCTACGACGGCTCCTCG
Shi 4185 GTGCAGATGGGGGGCCTTCCACGGCTGTGTGGGCGGTGTGGAGGTGGCGGCGCTACGACGGCTCCTCG
Mazhamai GTGCAGATGGGGGGCCTTCCACGGCTGTGTGGGCGGTGTGGAGGTGGCGGCGCTACGACGGCTCCTCG
Xiaofoshou GTGCAGATGGGGGGCCTTCCACGGCTGTGTGGGCGGTGTGGAGGTGGCGGCGCTACGACGGCTCCTCG
Aikang 58 GTGCAGATGGGGGGCCTTCCACGGCTGTGTGGGCGGTGTGGAGGTGGCGGCGCTACGACGGCTCCTCG
AL8/78
PI 431602
PI 486274
RM 000182
PI 511381
PI 486271
PI 511367
Chinese Spring
Hanzhongbai
Hongtoumai
Hongyouzi
Zijiehong
Baihuamai
Huixianhong
Baimangmai

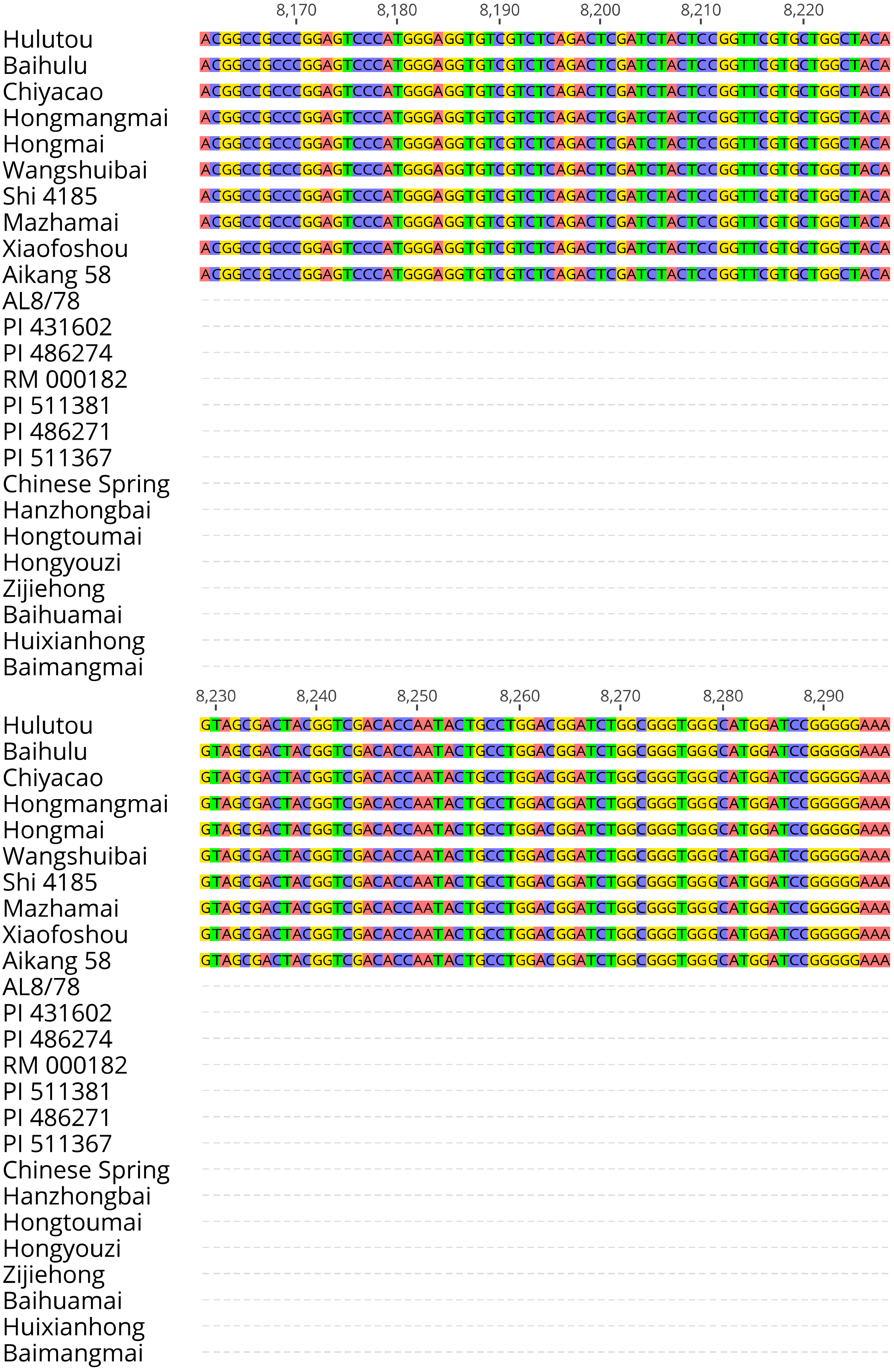
8,170 8,180 8,190 8,200 8,210 8,220
Hulutou ACGGCCGCCCGGAGTCCCATGGGAGGTGTCGTCTCAGACTCGATCTACTCCGGTTCGTGCTGGCTACA
Baihulu ACGGCCGCCCGGAGTCCCATGGGAGGTGTCGTCTCAGACTCGATCTACTCCGGTTCGTGCTGGCTACA
Chiyacao ACGGCCGCCCGGAGTCCCATGGGAGGTGTCGTCTCAGACTCGATCTACTCCGGTTCGTGCTGGCTACA
Hongmangmai ACGGCCGCCCGGAGTCCCATGGGAGGTGTCGTCTCAGACTCGATCTACTCCGGTTCGTGCTGGCTACA
Hongmai ACGGCCGCCCGGAGTCCCATGGGAGGTGTCGTCTCAGACTCGATCTACTCCGGTTCGTGCTGGCTACA
Wangshuibai ACGGCCGCCCGGAGTCCCATGGGAGGTGTCGTCTCAGACTCGATCTACTCCGGTTCGTGCTGGCTACA
Shi 4185 ACGGCCGCCCGGAGTCCCATGGGAGGTGTCGTCTCAGACTCGATCTACTCCGGTTCGTGCTGGCTACA
Mazhamai ACGGCCGCCCGGAGTCCCATGGGAGGTGTCGTCTCAGACTCGATCTACTCCGGTTCGTGCTGGCTACA
Xiaofoshou ACGGCCGCCCGGAGTCCCATGGGAGGTGTCGTCTCAGACTCGATCTACTCCGGTTCGTGCTGGCTACA
Aikang 58 ACGGCCGCCCGGAGTCCCATGGGAGGTGTCGTCTCAGACTCGATCTACTCCGGTTCGTGCTGGCTACA
AL8/78
PI 431602
PI 486274
RM 000182
PI 511381
PI 486271
PI 511367
Chinese Spring
Hanzhongbai
Hongtoumai
Hongyouzi
Zijiehong
Baihuamai
Huixianhong
Baimangmai
8,230 8,240 8,250 8,260 8,270 8,280 8,290
Hulutou GTAGCGACTACGGTCGACACCAATACTGCCTGGACGGATCTGGCGGGTGGGCATGGATCCGGGGGAAA
Baihulu GTAGCGACTACGGTCGACACCAATACTGCCTGGACGGATCTGGCGGGTGGGCATGGATCCGGGGGAAA
Chiyacao GTAGCGACTACGGTCGACACCAATACTGCCTGGACGGATCTGGCGGGTGGGCATGGATCCGGGGGAAA
Hongmangmai GTAGCGACTACGGTCGACACCAATACTGCCTGGACGGATCTGGCGGGTGGGCATGGATCCGGGGGAAA
Hongmai GTAGCGACTACGGTCGACACCAATACTGCCTGGACGGATCTGGCGGGTGGGCATGGATCCGGGGGAAA
Wangshuibai GTAGCGACTACGGTCGACACCAATACTGCCTGGACGGATCTGGCGGGTGGGCATGGATCCGGGGGAAA
Shi 4185 GTAGCGACTACGGTCGACACCAATACTGCCTGGACGGATCTGGCGGGTGGGCATGGATCCGGGGGAAA
Mazhamai GTAGCGACTACGGTCGACACCAATACTGCCTGGACGGATCTGGCGGGTGGGCATGGATCCGGGGGAAA
Xiaofoshou GTAGCGACTACGGTCGACACCAATACTGCCTGGACGGATCTGGCGGGTGGGCATGGATCCGGGGGAAA
Aikang 58 GTAGCGACTACGGTCGACACCAATACTGCCTGGACGGATCTGGCGGGTGGGCATGGATCCGGGGGAAA
AL8/78
PI 431602
PI 486274
RM 000182
PI 511381
PI 486271
PI 511367
Chinese Spring
Hanzhongbai
Hongtoumai
Hongyouzi
Zijiehong
Baihuamai
Huixianhong
Baimangmai

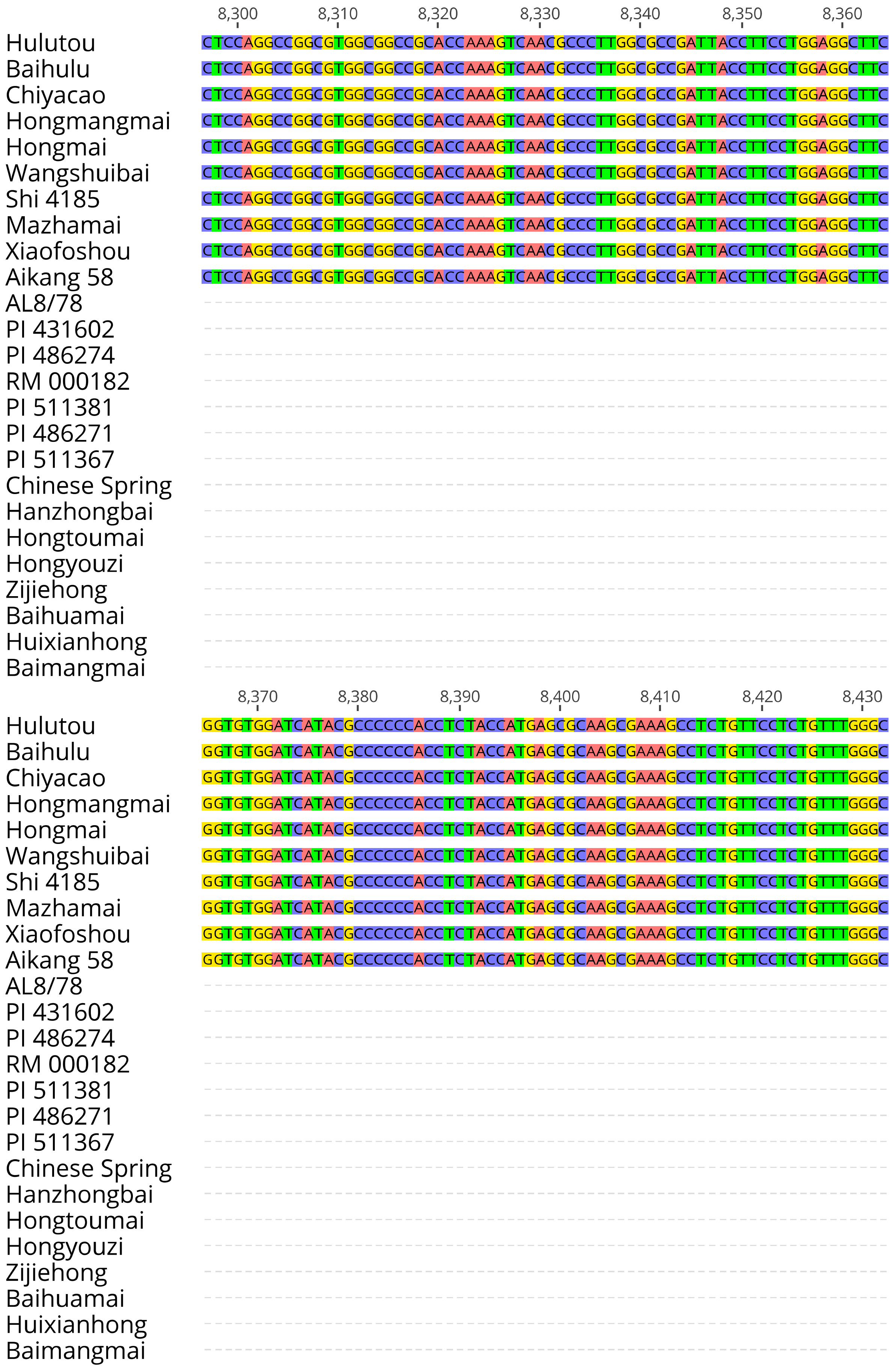
8,300 8,310 8,320 8,330 8,340 8,350 8,360
Hulutou CTCCAGGCCGGCGTGGCGGCCGCACCAAAGTCAACGCCCTTGGCGCCGATTACCTTCCTGGAGGCTTC
Baihulu CTCCAGGCCGGCGTGGCGGCCGCACCAAAGTCAACGCCCTTGGCGCCGATTACCTTCCTGGAGGCTTC
Chiyacao CTCCAGGCCGGCGTGGCGGCCGCACCAAAGTCAACGCCCTTGGCGCCGATTACCTTCCTGGAGGCTTC
Hongmangmai CTCCAGGCCGGCGTGGCGGCCGCACCAAAGTCAACGCCCTTGGCGCCGATTACCTTCCTGGAGGCTTC
Hongmai CTCCAGGCCGGCGTGGCGGCCGCACCAAAGTCAACGCCCTTGGCGCCGATTACCTTCCTGGAGGCTTC
Wangshuibai CTCCAGGCCGGCGTGGCGGCCGCACCAAAGTCAACGCCCTTGGCGCCGATTACCTTCCTGGAGGCTTC
Shi 4185 CTCCAGGCCGGCGTGGCGGCCGCACCAAAGTCAACGCCCTTGGCGCCGATTACCTTCCTGGAGGCTTC
Mazhamai CTCCAGGCCGGCGTGGCGGCCGCACCAAAGTCAACGCCCTTGGCGCCGATTACCTTCCTGGAGGCTTC
Xiaofoshou CTCCAGGCCGGCGTGGCGGCCGCACCAAAGTCAACGCCCTTGGCGCCGATTACCTTCCTGGAGGCTTC
Aikang 58 CTCCAGGCCGGCGTGGCGGCCGCACCAAAGTCAACGCCCTTGGCGCCGATTACCTTCCTGGAGGCTTC
AL8/78
PI 431602
PI 486274
RM 000182
PI 511381
PI 486271
PI 511367
Chinese Spring
Hanzhongbai
Hongtoumai
Hongyouzi
Zijiehong
Baihuamai
Huixianhong
Baimangmai
8,370 8,380 8,390 8,400 8,410 8,420 8,430
Hulutou GGTGTGGATCATACGCCCCCCACCTCTACCATGAGCGCAAGCGAAAGCCTCTGTTCCTCTGTTTGGGC
Baihulu GGTGTGGATCATACGCCCCCCACCTCTACCATGAGCGCAAGCGAAAGCCTCTGTTCCTCTGTTTGGGC
Chiyacao GGTGTGGATCATACGCCCCCCACCTCTACCATGAGCGCAAGCGAAAGCCTCTGTTCCTCTGTTTGGGC
Hongmangmai GGTGTGGATCATACGCCCCCCACCTCTACCATGAGCGCAAGCGAAAGCCTCTGTTCCTCTGTTTGGGC
Hongmai GGTGTGGATCATACGCCCCCCACCTCTACCATGAGCGCAAGCGAAAGCCTCTGTTCCTCTGTTTGGGC
Wangshuibai GGTGTGGATCATACGCCCCCCACCTCTACCATGAGCGCAAGCGAAAGCCTCTGTTCCTCTGTTTGGGC
Shi 4185 GGTGTGGATCATACGCCCCCCACCTCTACCATGAGCGCAAGCGAAAGCCTCTGTTCCTCTGTTTGGGC
Mazhamai GGTGTGGATCATACGCCCCCCACCTCTACCATGAGCGCAAGCGAAAGCCTCTGTTCCTCTGTTTGGGC
Xiaofoshou GGTGTGGATCATACGCCCCCCACCTCTACCATGAGCGCAAGCGAAAGCCTCTGTTCCTCTGTTTGGGC
Aikang 58 GGTGTGGATCATACGCCCCCCACCTCTACCATGAGCGCAAGCGAAAGCCTCTGTTCCTCTGTTTGGGC
AL8/78
PI 431602
PI 486274
RM 000182
PI 511381
PI 486271
PI 511367
Chinese Spring
Hanzhongbai
Hongtoumai
Hongyouzi
Zijiehong
Baihuamai
Huixianhong
Baimangmai

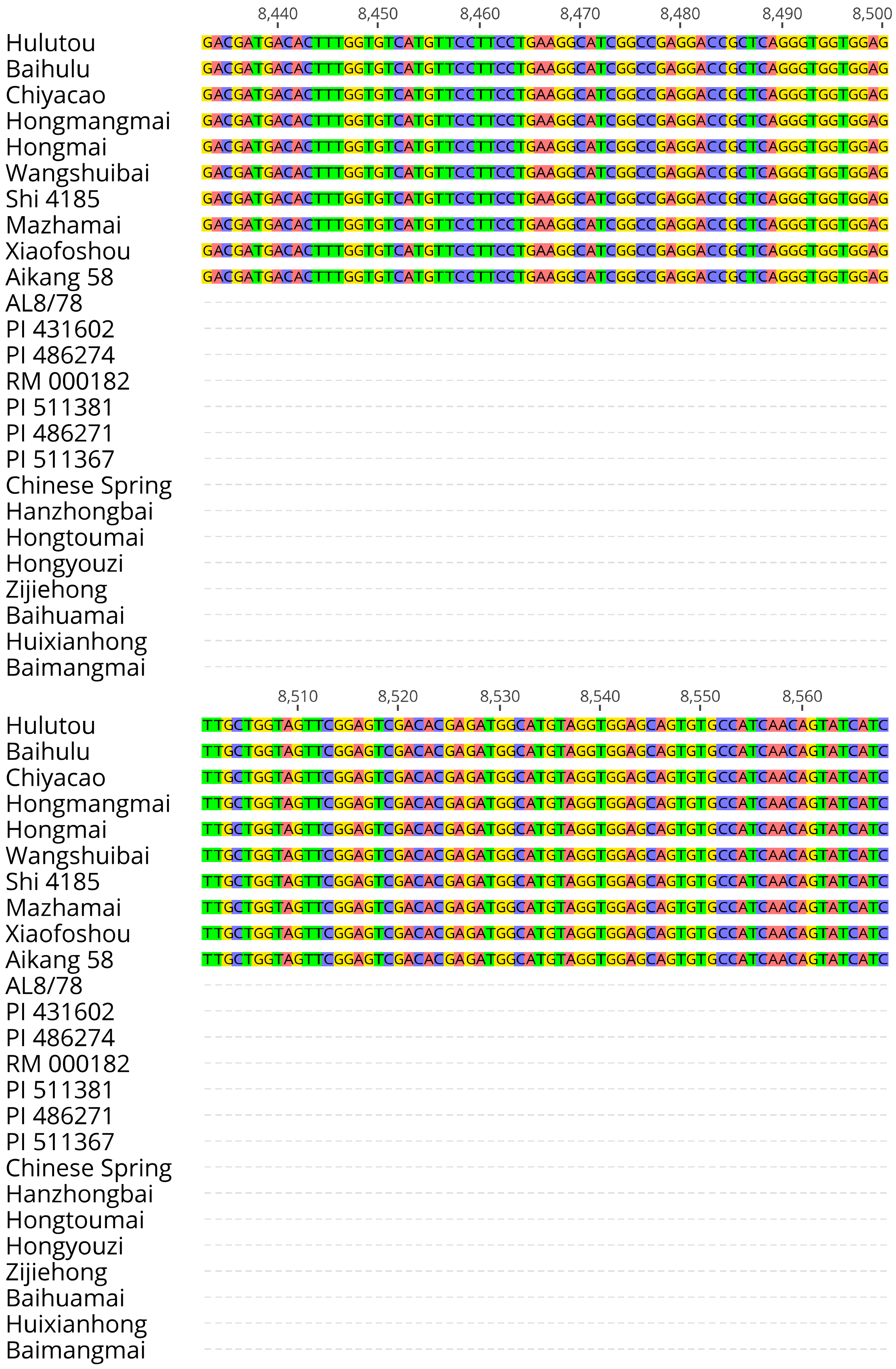

8,440 8,450 8,460 8,470 8,480 8,490 8,500
Hulutou GACGATGACACTTTGGTGTCATGTTCCTTCCTGAAGGCATCGGCCGAGGACCGCTCAGGGTGGTGGAG
Baihulu GACGATGACACTTTGGTGTCATGTTCCTTCCTGAAGGCATCGGCCGAGGACCGCTCAGGGTGGTGGAG
Chiyacao GACGATGACACTTTGGTGTCATGTTCCTTCCTGAAGGCATCGGCCGAGGACCGCTCAGGGTGGTGGAG
Hongmangmai GACGATGACACTTTGGTGTCATGTTCCTTCCTGAAGGCATCGGCCGAGGACCGCTCAGGGTGGTGGAG
Hongmai GACGATGACACTTTGGTGTCATGTTCCTTCCTGAAGGCATCGGCCGAGGACCGCTCAGGGTGGTGGAG
Wangshuibai GACGATGACACTTTGGTGTCATGTTCCTTCCTGAAGGCATCGGCCGAGGACCGCTCAGGGTGGTGGAG
Shi 4185 GACGATGACACTTTGGTGTCATGTTCCTTCCTGAAGGCATCGGCCGAGGACCGCTCAGGGTGGTGGAG
Mazhamai GACGATGACACTTTGGTGTCATGTTCCTTCCTGAAGGCATCGGCCGAGGACCGCTCAGGGTGGTGGAG
Xiaofoshou GACGATGACACTTTGGTGTCATGTTCCTTCCTGAAGGCATCGGCCGAGGACCGCTCAGGGTGGTGGAG
Aikang 58 GACGATGACACTTTGGTGTCATGTTCCTTCCTGAAGGCATCGGCCGAGGACCGCTCAGGGTGGTGGAG
AL8/78
PI 431602
PI 486274
RM 000182
PI 511381
PI 486271
PI 511367
Chinese Spring
Hanzhongbai
Hongtoumai
Hongyouzi
Zijiehong
Baihuamai
Huixianhong
Baimangmai
8,510 8,520 8,530 8,540 8,550 8,560
Hulutou TTGCTGGTAGTTCGGAGTCGACACGAGATGGCATGTAGGTGGAGCAGTGTGCCATCAACAGTATCATC
Baihulu TTGCTGGTAGTTCGGAGTCGACACGAGATGGCATGTAGGTGGAGCAGTGTGCCATCAACAGTATCATC
Chiyacao TTGCTGGTAGTTCGGAGTCGACACGAGATGGCATGTAGGTGGAGCAGTGTGCCATCAACAGTATCATC
Hongmangmai TTGCTGGTAGTTCGGAGTCGACACGAGATGGCATGTAGGTGGAGCAGTGTGCCATCAACAGTATCATC
Hongmai TTGCTGGTAGTTCGGAGTCGACACGAGATGGCATGTAGGTGGAGCAGTGTGCCATCAACAGTATCATC
Wangshuibai TTGCTGGTAGTTCGGAGTCGACACGAGATGGCATGTAGGTGGAGCAGTGTGCCATCAACAGTATCATC
Shi 4185 TTGCTGGTAGTTCGGAGTCGACACGAGATGGCATGTAGGTGGAGCAGTGTGCCATCAACAGTATCATC
Mazhamai TTGCTGGTAGTTCGGAGTCGACACGAGATGGCATGTAGGTGGAGCAGTGTGCCATCAACAGTATCATC
Xiaofoshou TTGCTGGTAGTTCGGAGTCGACACGAGATGGCATGTAGGTGGAGCAGTGTGCCATCAACAGTATCATC
Aikang 58 TTGCTGGTAGTTCGGAGTCGACACGAGATGGCATGTAGGTGGAGCAGTGTGCCATCAACAGTATCATC
AL8/78
PI 431602
PI 486274
RM 000182
PI 511381
PI 486271
PI 511367
Chinese Spring
Hanzhongbai
Hongtoumai
Hongyouzi
Zijiehong
Baihuamai
Huixianhong
Baimangmai

Accession	8,570 – 8,630
Hulutou	GACGGTGGGTCTCGGCGGCATGGCGCAGTGGAGTCTCGGCGTCCGATGCGCGGAGATGGACTCGCGCA
Baihulu	GACGGTGGGTCTCGGCGGCATGGCGCAGTGGAGTCTCGGCGTCCGATGCGCGGAGATGGACTCGCGCA
Chiyacao	GACGGTGGGTCTCGGCGGCATGGCGCAGTGGAGTCTCGGCGTCCGATGCGCGGAGATGGACTCGCGCA
Hongmangmai	GACGGTGGGTCTCGGCGGCATGGCGCAGTGGAGTCTCGGCGTCCGATGCGCGGAGATGGACTCGCGCA
Hongmai	GACGGTGGGTCTCGGCGGCATGGCGCAGTGGAGTCTCGGCGTCCGATGCGCGGAGATGGACTCGCGCA
Wangshuibai	GACGGTGGGTCTCGGCGGCATGGCGCAGTGGAGTCTCGGCGTCCGATGCGCGGAGATGGACTCGCGCA
Shi 4185	GACGGTGGGTCTCGGCGGCATGGCGCAGTGGAGTCTCGGCGTCCGATGCGCGGAGATGGACTCGCGCA
Mazhamai	GACGGTGGGTCTCGGCGGCATGGCGCAGTGGAGTCTCGGCGTCCGATGCGCGGAGATGGACTCGCGCA
Xiaofoshou	GACGGTGGGTCTCGGCGGCATGGCGCAGTGGAGTCTCGGCGTCCGATGCGCGGAGATGGACTCGCGCA
Aikang 58	GACGGTGGGTCTCGGCGGCATGGCGCAGTGGAGTCTCGGCGTCCGATGCGCGGAGATGGACTCGCGCA
AL8/78	
PI 431602	
PI 486274	
RM 000182	
PI 511381	
PI 486271	
PI 511367	
Chinese Spring	
Hanzhongbai	
Hongtoumai	
Hongyouzi	
Zijiehong	
Baihuamai	
Huixianhong	
Baimangmai	

Accession	8,640 – 8,700
Hulutou	GGAGGAGGTAGCTGTCTGGCGTCGTGGTGGCGTCGACGGTAGCTAGACCGTGCAAGGGAGATGCAACA
Baihulu	GGAGGAGGTAGCTGTCTGGCGTCGTGGTGGCGTCGACGGTAGCTAGACCGTGCAAGGGAGATGCAACA
Chiyacao	GGAGGAGGTAGCTGTCTGGCGTCGTGGTGGCGTCGACGGTAGCTAGACCGTGCAAGGGAGATGCAACA
Hongmangmai	GGAGGAGGTAGCTGTCTGGCGTCGTGGTGGCGTCGACGGTAGCTAGACCGTGCAAGGGAGATGCAACA
Hongmai	GGAGGAGGTAGCTGTCTGGCGTCGTGGTGGCGTCGACGGTAGCTAGACCGTGCAAGGGAGATGCAACA
Wangshuibai	GGAGGAGGTAGCTGTCTGGCGTCGTGGTGGCGTCGACGGTAGCTAGACCGTGCAAGGGAGATGCAACA
Shi 4185	GGAGGAGGTAGCTGTCTGGCGTCGTGGTGGCGTCGACGGTAGCTAGACCGTGCAAGGGAGATGCAACA
Mazhamai	GGAGGAGGTAGCTGTCTGGCGTCGTGGTGGCGTCGACGGTAGCTAGACCGTGCAAGGGAGATGCAACA
Xiaofoshou	GGAGGAGGTAGCTGTCTGGCGTCGTGGTGGCGTCGACGGTAGCTAGACCGTGCAAGGGAGATGCAACA
Aikang 58	GGAGGAGGTAGCTGTCTGGCGTCGTGGTGGCGTCGACGGTAGCTAGACCGTGCAAGGGAGATGCAACA
AL8/78	
PI 431602	
PI 486274	
RM 000182	
PI 511381	
PI 486271	
PI 511367	
Chinese Spring	
Hanzhongbai	
Hongtoumai	
Hongyouzi	
Zijiehong	
Baihuamai	
Huixianhong	
Baimangmai	

	8,710 – 8,770
Hulutou	GTACAACTCTGAAGATGGATTGGTGGCAGGTGGCTGCGGCAGCCTCATACCCGGCAGGCGTCCTGGTT
Baihulu	GTACAACTCTGAAGATGGATTGGTGGCAGGTGGCTGCGGCAGCCTCATACCCGGCAGGCGTCCTGGTT
Chiyacao	GTACAACTCTGAAGATGGATTGGTGGCAGGTGGCTGCGGCAGCCTCATACCCGGCAGGCGTCCTGGTT
Hongmangmai	GTACAACTCTGAAGATGGATTGGTGGCAGGTGGCTGCGGCAGCCTCATACCCGGCAGGCGTCCTGGTT
Hongmai	GTACAACTCTGAAGATGGATTGGTGGCAGGTGGCTGCGGCAGCCTCATACCCGGCAGGCGTCCTGGTT
Wangshuibai	GTACAACTCTGAAGATGGATTGGTGGCAGGTGGCTGCGGCAGCCTCATACCCGGCAGGCGTCCTGGTT
Shi 4185	GTACAACTCTGAAGATGGATTGGTGGCAGGTGGCTGCGGCAGCCTCATACCCGGCAGGCGTCCTGGTT
Mazhamai	GTACAACTCTGAAGATGGATTGGTGGCAGGTGGCTGCGGCAGCCTCATACCCGGCAGGCGTCCTGGTT
Xiaofoshou	GTACAACTCTGAAGATGGATTGGTGGCAGGTGGCTGCGGCAGCCTCATACCCGGCAGGCGTCCTGGTT
Aikang 58	GTACAACTCTGAAGATGGATTGGTGGCAGGTGGCTGCGGCAGCCTCATACCCGGCAGGCGTCCTGGTT
AL8/78	
PI 431602	
PI 486274	
RM 000182	
PI 511381	
PI 486271	
PI 511367	
Chinese Spring	
Hanzhongbai	
Hongtoumai	
Hongyouzi	
Zijiehong	
Baihuamai	
Huixianhong	
Baimangmai	

	8,780 – 8,840
Hulutou	GAGGAGTGCGCTGGACTGGTAGCAGGTGCCCCATACCCGGCAGGCGTCCTGGTTGAGGAGTGCGCTGG
Baihulu	GAGGAGTGCGCTGGACTGGTAGCAGGTGCCCCATACCCGGCAGGCGTCCTGGTTGAGGAGTGCGCTGG
Chiyacao	GAGGAGTGCGCTGGACTGGTAGCAGGTGCCCCATACCCGGCAGGCGTCCTGGTTGAGGAGTGCGCTGG
Hongmangmai	GAGGAGTGCGCTGGACTGGTAGCAGGTGCCCCATACCCGGCAGGCGTCCTGGTTGAGGAGTGCGCTGG
Hongmai	GAGGAGTGCGCTGGACTGGTAGCAGGTGCCCCATACCCGGCAGGCGTCCTGGTTGAGGAGTGCGCTGG
Wangshuibai	GAGGAGTGCGCTGGACTGGTAGCAGGTGCCCCATACCCGGCAGGCGTCCTGGTTGAGGAGTGCGCTGG
Shi 4185	GAGGAGTGCGCTGGACTGGTAGCAGGTGCCCCATACCCGGCAGGCGTCCTGGTTGAGGAGTGCGCTGG
Mazhamai	GAGGAGTGCGCTGGACTGGTAGCAGGTGCCCCATACCCGGCAGGCGTCCTGGTTGAGGAGTGCGCTGG
Xiaofoshou	GAGGAGTGCGCTGGACTGGTAGCAGGTGCCCCATACCCGGCAGGCGTCCTGGTTGAGGAGTGCGCTGG
Aikang 58	GAGGAGTGCGCTGGACTGGTAGCAGGTGCCCCATACCCGGCAGGCGTCCTGGTTGAGGAGTGCGCTGG
AL8/78	
PI 431602	
PI 486274	
RM 000182	
PI 511381	
PI 486271	
PI 511367	
Chinese Spring	
Hanzhongbai	
Hongtoumai	
Hongyouzi	
Zijiehong	
Baihuamai	
Huixianhong	
Baimangmai	

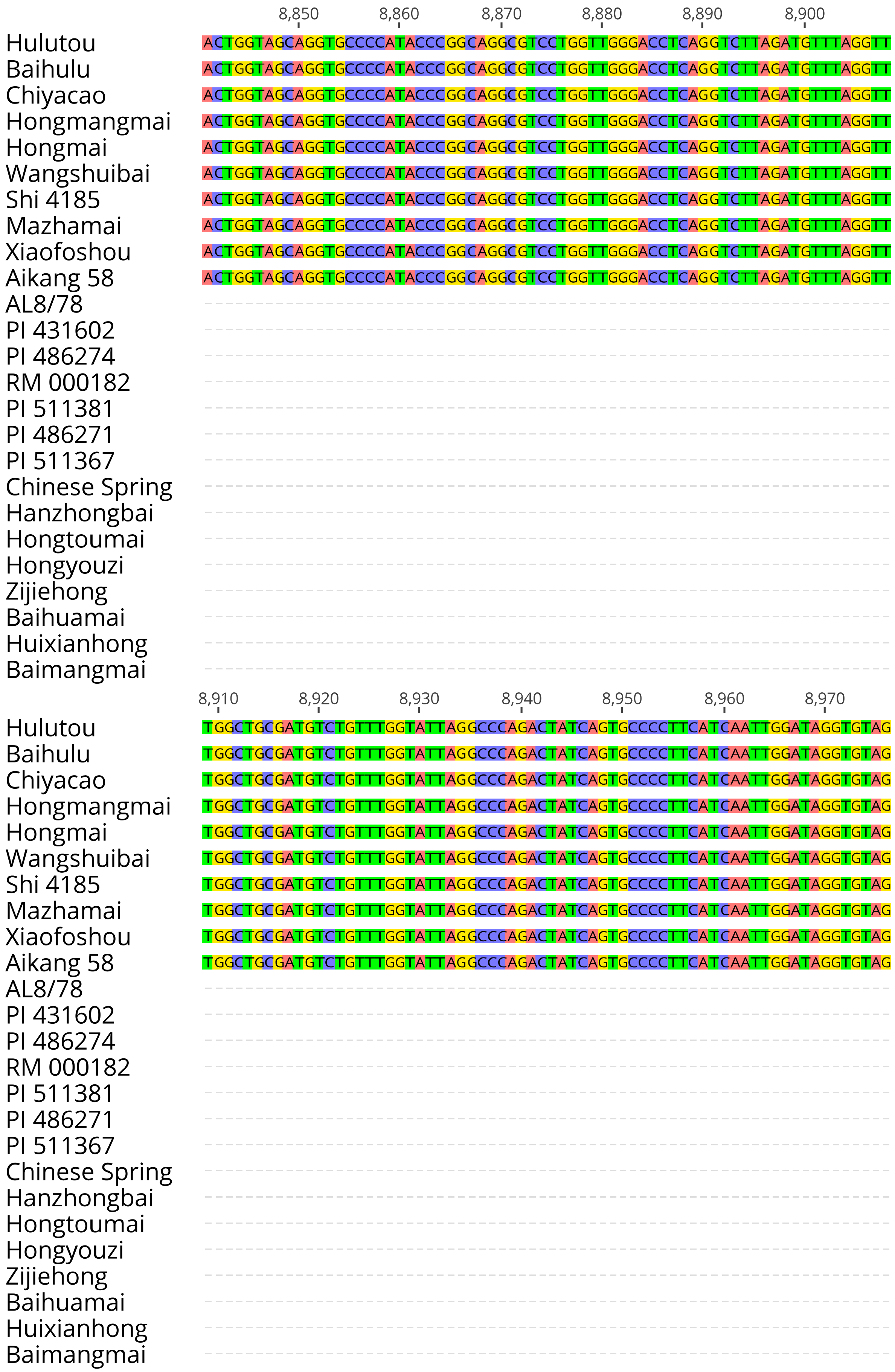

8,850
8,860
8,870
8,880
8,890
8,900
Hulutou ACTGGTAGCAGGTGCCCCATACCCGGCAGGCGTCCTGGTTGGGACCTCAGGTCTTAGATGTTTAGGTT
Baihulu ACTGGTAGCAGGTGCCCCATACCCGGCAGGCGTCCTGGTTGGGACCTCAGGTCTTAGATGTTTAGGTT
Chiyacao ACTGGTAGCAGGTGCCCCATACCCGGCAGGCGTCCTGGTTGGGACCTCAGGTCTTAGATGTTTAGGTT
Hongmangmai ACTGGTAGCAGGTGCCCCATACCCGGCAGGCGTCCTGGTTGGGACCTCAGGTCTTAGATGTTTAGGTT
Hongmai ACTGGTAGCAGGTGCCCCATACCCGGCAGGCGTCCTGGTTGGGACCTCAGGTCTTAGATGTTTAGGTT
Wangshuibai ACTGGTAGCAGGTGCCCCATACCCGGCAGGCGTCCTGGTTGGGACCTCAGGTCTTAGATGTTTAGGTT
Shi 4185 ACTGGTAGCAGGTGCCCCATACCCGGCAGGCGTCCTGGTTGGGACCTCAGGTCTTAGATGTTTAGGTT
Mazhamai ACTGGTAGCAGGTGCCCCATACCCGGCAGGCGTCCTGGTTGGGACCTCAGGTCTTAGATGTTTAGGTT
Xiaofoshou ACTGGTAGCAGGTGCCCCATACCCGGCAGGCGTCCTGGTTGGGACCTCAGGTCTTAGATGTTTAGGTT
Aikang 58 ACTGGTAGCAGGTGCCCCATACCCGGCAGGCGTCCTGGTTGGGACCTCAGGTCTTAGATGTTTAGGTT
AL8/78
PI 431602
PI 486274
RM 000182
PI 511381
PI 486271
PI 511367
Chinese Spring
Hanzhongbai
Hongtoumai
Hongyouzi
Zijiehong
Baihuamai
Huixianhong
Baimangmai
8,910
8,920
8,930
8,940
8,950
8,960
8,970
Hulutou TGGCTGCGATGTCTGTTTGGTATTAGGCCCAGACTATCAGTGCCCCTTCATCAATTGGATAGGTGTAG
Baihulu TGGCTGCGATGTCTGTTTGGTATTAGGCCCAGACTATCAGTGCCCCTTCATCAATTGGATAGGTGTAG
Chiyacao TGGCTGCGATGTCTGTTTGGTATTAGGCCCAGACTATCAGTGCCCCTTCATCAATTGGATAGGTGTAG
Hongmangmai TGGCTGCGATGTCTGTTTGGTATTAGGCCCAGACTATCAGTGCCCCTTCATCAATTGGATAGGTGTAG
Hongmai TGGCTGCGATGTCTGTTTGGTATTAGGCCCAGACTATCAGTGCCCCTTCATCAATTGGATAGGTGTAG
Wangshuibai TGGCTGCGATGTCTGTTTGGTATTAGGCCCAGACTATCAGTGCCCCTTCATCAATTGGATAGGTGTAG
Shi 4185 TGGCTGCGATGTCTGTTTGGTATTAGGCCCAGACTATCAGTGCCCCTTCATCAATTGGATAGGTGTAG
Mazhamai TGGCTGCGATGTCTGTTTGGTATTAGGCCCAGACTATCAGTGCCCCTTCATCAATTGGATAGGTGTAG
Xiaofoshou TGGCTGCGATGTCTGTTTGGTATTAGGCCCAGACTATCAGTGCCCCTTCATCAATTGGATAGGTGTAG
Aikang 58 TGGCTGCGATGTCTGTTTGGTATTAGGCCCAGACTATCAGTGCCCCTTCATCAATTGGATAGGTGTAG
AL8/78
PI 431602
PI 486274
RM 000182
PI 511381
PI 486271
PI 511367
Chinese Spring
Hanzhongbai
Hongtoumai
Hongyouzi
Zijiehong
Baihuamai
Huixianhong
Baimangmai

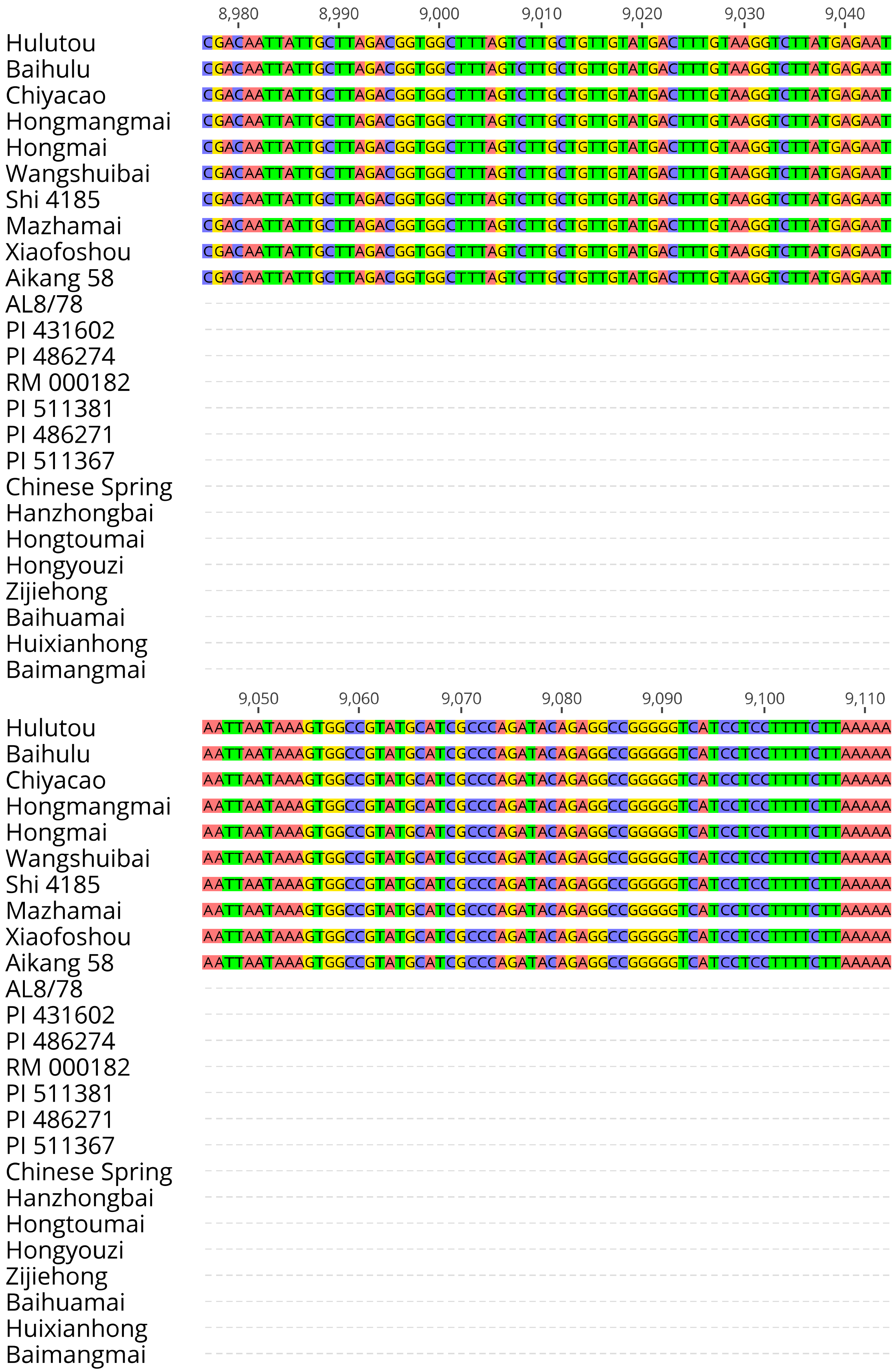
8,980
8,990
9,000
9,010
9,020
9,030
9,040
Hulutou CGACAATTATTGCTTAGACGGTGGCTTTAGTCTTGCTGTTGTATGACTTTGTAAGGTCTTATGAGAAT
Baihulu CGACAATTATTGCTTAGACGGTGGCTTTAGTCTTGCTGTTGTATGACTTTGTAAGGTCTTATGAGAAT
Chiyacao CGACAATTATTGCTTAGACGGTGGCTTTAGTCTTGCTGTTGTATGACTTTGTAAGGTCTTATGAGAAT
Hongmangmai CGACAATTATTGCTTAGACGGTGGCTTTAGTCTTGCTGTTGTATGACTTTGTAAGGTCTTATGAGAAT
Hongmai CGACAATTATTGCTTAGACGGTGGCTTTAGTCTTGCTGTTGTATGACTTTGTAAGGTCTTATGAGAAT
Wangshuibai CGACAATTATTGCTTAGACGGTGGCTTTAGTCTTGCTGTTGTATGACTTTGTAAGGTCTTATGAGAAT
Shi 4185 CGACAATTATTGCTTAGACGGTGGCTTTAGTCTTGCTGTTGTATGACTTTGTAAGGTCTTATGAGAAT
Mazhamai CGACAATTATTGCTTAGACGGTGGCTTTAGTCTTGCTGTTGTATGACTTTGTAAGGTCTTATGAGAAT
Xiaofoshou CGACAATTATTGCTTAGACGGTGGCTTTAGTCTTGCTGTTGTATGACTTTGTAAGGTCTTATGAGAAT
Aikang 58 CGACAATTATTGCTTAGACGGTGGCTTTAGTCTTGCTGTTGTATGACTTTGTAAGGTCTTATGAGAAT
AL8/78
PI 431602
PI 486274
RM 000182
PI 511381
PI 486271
PI 511367
Chinese Spring
Hanzhongbai
Hongtoumai
Hongyouzi
Zijiehong
Baihuamai
Huixianhong
Baimangmai
9,050
9,060
9,070
9,080
9,090
9,100
9,110
Hulutou AATTAATAAAGTGGCCGTATGCATCGCCCAGATACAGAGGCCGGGGGTCATCCTCCTTTTTCTTAAAAA
Baihulu AATTAATAAAGTGGCCGTATGCATCGCCCAGATACAGAGGCCGGGGGTCATCCTCCTTTTTCTTAAAAA
Chiyacao AATTAATAAAGTGGCCGTATGCATCGCCCAGATACAGAGGCCGGGGGTCATCCTCCTTTTTCTTAAAAA
Hongmangmai AATTAATAAAGTGGCCGTATGCATCGCCCAGATACAGAGGCCGGGGGTCATCCTCCTTTTTCTTAAAAA
Hongmai AATTAATAAAGTGGCCGTATGCATCGCCCAGATACAGAGGCCGGGGGTCATCCTCCTTTTTCTTAAAAA
Wangshuibai AATTAATAAAGTGGCCGTATGCATCGCCCAGATACAGAGGCCGGGGGTCATCCTCCTTTTTCTTAAAAA
Shi 4185 AATTAATAAAGTGGCCGTATGCATCGCCCAGATACAGAGGCCGGGGGTCATCCTCCTTTTTCTTAAAAA
Mazhamai AATTAATAAAGTGGCCGTATGCATCGCCCAGATACAGAGGCCGGGGGTCATCCTCCTTTTTCTTAAAAA
Xiaofoshou AATTAATAAAGTGGCCGTATGCATCGCCCAGATACAGAGGCCGGGGGTCATCCTCCTTTTTCTTAAAAA
Aikang 58 AATTAATAAAGTGGCCGTATGCATCGCCCAGATACAGAGGCCGGGGGTCATCCTCCTTTTTCTTAAAAA
AL8/78
PI 431602
PI 486274
RM 000182
PI 511381
PI 486271
PI 511367
Chinese Spring
Hanzhongbai
Hongtoumai
Hongyouzi
Zijiehong
Baihuamai
Huixianhong
Baimangmai

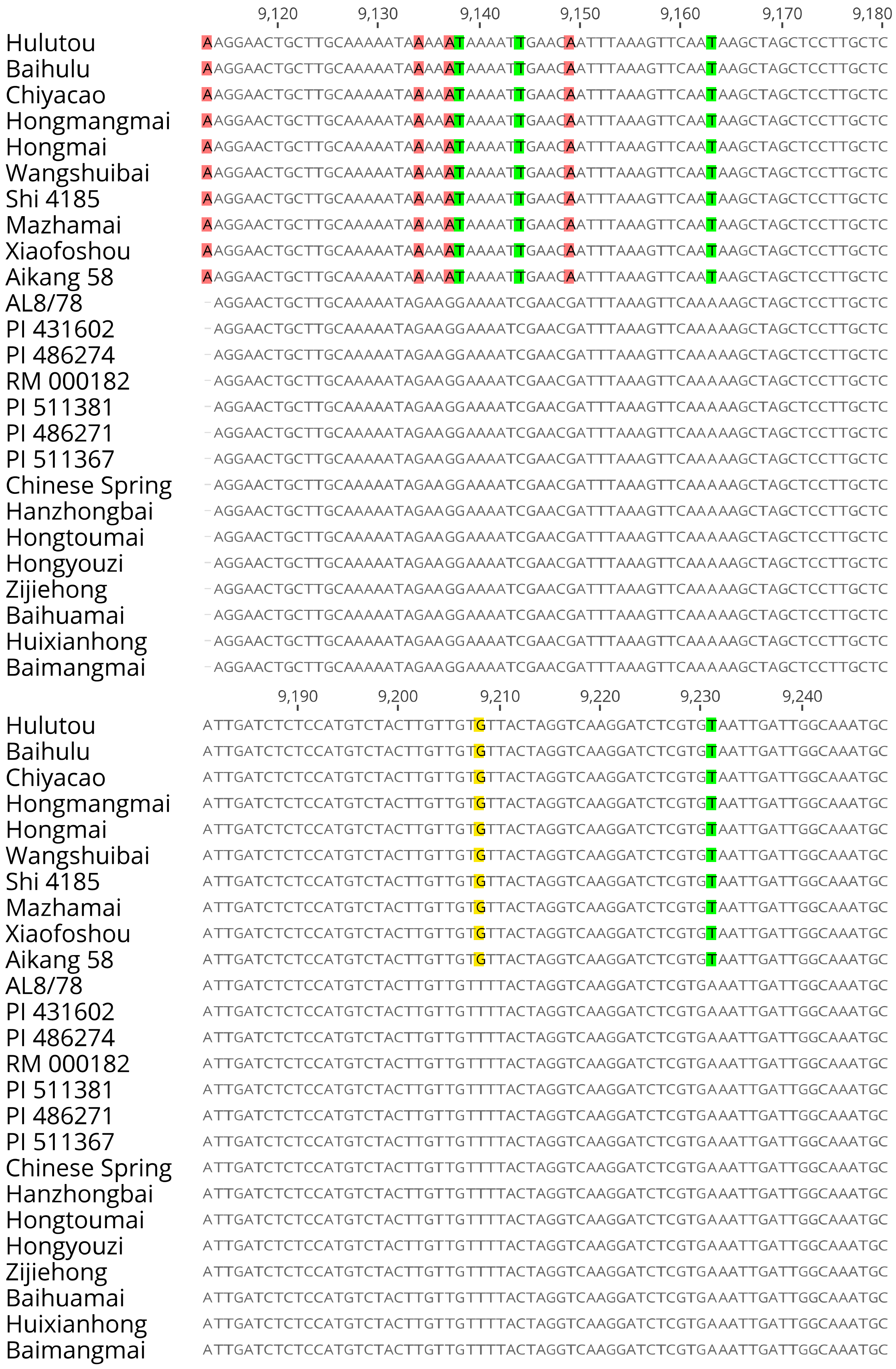

9,120
9,130
9,140
9,150
9,160
9,170
9,180
Hulutou AAGGAACTGCTTGCAAAAATAAAAATAAAATTGAACAATTTAAAGTTCAATAAGCTAGCTCCTTGCTC
Baihulu AAGGAACTGCTTGCAAAAATAAAAATAAAATTGAACAATTTAAAGTTCAATAAGCTAGCTCCTTGCTC
Chiyacao AAGGAACTGCTTGCAAAAATAAAAATAAAATTGAACAATTTAAAGTTCAATAAGCTAGCTCCTTGCTC
Hongmangmai AAGGAACTGCTTGCAAAAATAAAAATAAAATTGAACAATTTAAAGTTCAATAAGCTAGCTCCTTGCTC
Hongmai AAGGAACTGCTTGCAAAAATAAAAATAAAATTGAACAATTTAAAGTTCAATAAGCTAGCTCCTTGCTC
Wangshuibai AAGGAACTGCTTGCAAAAATAAAAATAAAATTGAACAATTTAAAGTTCAATAAGCTAGCTCCTTGCTC
Shi 4185 AAGGAACTGCTTGCAAAAATAAAAATAAAATTGAACAATTTAAAGTTCAATAAGCTAGCTCCTTGCTC
Mazhamai AAGGAACTGCTTGCAAAAATAAAAATAAAATTGAACAATTTAAAGTTCAATAAGCTAGCTCCTTGCTC
Xiaofoshou AAGGAACTGCTTGCAAAAATAAAAATAAAATTGAACAATTTAAAGTTCAATAAGCTAGCTCCTTGCTC
Aikang 58 AAGGAACTGCTTGCAAAAATAAAAATAAAATTGAACAATTTAAAGTTCAATAAGCTAGCTCCTTGCTC
AL8/78 -AGGAACTGCTTGCAAAAATAGAAGGAAAATCGAACGATTTAAAGTTCAAAAAGCTAGCTCCTTGCTC
PI 431602 -AGGAACTGCTTGCAAAAATAGAAGGAAAATCGAACGATTTAAAGTTCAAAAAGCTAGCTCCTTGCTC
PI 486274 -AGGAACTGCTTGCAAAAATAGAAGGAAAATCGAACGATTTAAAGTTCAAAAAGCTAGCTCCTTGCTC
RM 000182 -AGGAACTGCTTGCAAAAATAGAAGGAAAATCGAACGATTTAAAGTTCAAAAAGCTAGCTCCTTGCTC
PI 511381 -AGGAACTGCTTGCAAAAATAGAAGGAAAATCGAACGATTTAAAGTTCAAAAAGCTAGCTCCTTGCTC
PI 486271 -AGGAACTGCTTGCAAAAATAGAAGGAAAATCGAACGATTTAAAGTTCAAAAAGCTAGCTCCTTGCTC
PI 511367 -AGGAACTGCTTGCAAAAATAGAAGGAAAATCGAACGATTTAAAGTTCAAAAAGCTAGCTCCTTGCTC
Chinese Spring -AGGAACTGCTTGCAAAAATAGAAGGAAAATCGAACGATTTAAAGTTCAAAAAGCTAGCTCCTTGCTC
Hanzhongbai -AGGAACTGCTTGCAAAAATAGAAGGAAAATCGAACGATTTAAAGTTCAAAAAGCTAGCTCCTTGCTC
Hongtoumai -AGGAACTGCTTGCAAAAATAGAAGGAAAATCGAACGATTTAAAGTTCAAAAAGCTAGCTCCTTGCTC
Hongyouzi -AGGAACTGCTTGCAAAAATAGAAGGAAAATCGAACGATTTAAAGTTCAAAAAGCTAGCTCCTTGCTC
Zijiehong -AGGAACTGCTTGCAAAAATAGAAGGAAAATCGAACGATTTAAAGTTCAAAAAGCTAGCTCCTTGCTC
Baihuamai -AGGAACTGCTTGCAAAAATAGAAGGAAAATCGAACGATTTAAAGTTCAAAAAGCTAGCTCCTTGCTC
Huixianhong -AGGAACTGCTTGCAAAAATAGAAGGAAAATCGAACGATTTAAAGTTCAAAAAGCTAGCTCCTTGCTC
Baimangmai -AGGAACTGCTTGCAAAAATAGAAGGAAAATCGAACGATTTAAAGTTCAAAAAGCTAGCTCCTTGCTC
9,190
9,200
9,210
9,220
9,230
9,240
Hulutou ATTGATCTCTCCATGTCTACTTGTTGTGTTACTAGGTCAAGGATCTCGTGTAATTGATTGGCAAATGC
Baihulu ATTGATCTCTCCATGTCTACTTGTTGTGTTACTAGGTCAAGGATCTCGTGTAATTGATTGGCAAATGC
Chiyacao ATTGATCTCTCCATGTCTACTTGTTGTGTTACTAGGTCAAGGATCTCGTGTAATTGATTGGCAAATGC
Hongmangmai ATTGATCTCTCCATGTCTACTTGTTGTGTTACTAGGTCAAGGATCTCGTGTAATTGATTGGCAAATGC
Hongmai ATTGATCTCTCCATGTCTACTTGTTGTGTTACTAGGTCAAGGATCTCGTGTAATTGATTGGCAAATGC
Wangshuibai ATTGATCTCTCCATGTCTACTTGTTGTGTTACTAGGTCAAGGATCTCGTGTAATTGATTGGCAAATGC
Shi 4185 ATTGATCTCTCCATGTCTACTTGTTGTGTTACTAGGTCAAGGATCTCGTGTAATTGATTGGCAAATGC
Mazhamai ATTGATCTCTCCATGTCTACTTGTTGTGTTACTAGGTCAAGGATCTCGTGTAATTGATTGGCAAATGC
Xiaofoshou ATTGATCTCTCCATGTCTACTTGTTGTGTTACTAGGTCAAGGATCTCGTGTAATTGATTGGCAAATGC
Aikang 58 ATTGATCTCTCCATGTCTACTTGTTGTGTTACTAGGTCAAGGATCTCGTGTAATTGATTGGCAAATGC
AL8/78 ATTGATCTCTCCATGTCTACTTGTTGTTTTACTAGGTCAAGGATCTCGTGAAATTGATTGGCAAATGC
PI 431602 ATTGATCTCTCCATGTCTACTTGTTGTTTTACTAGGTCAAGGATCTCGTGAAATTGATTGGCAAATGC
PI 486274 ATTGATCTCTCCATGTCTACTTGTTGTTTTACTAGGTCAAGGATCTCGTGAAATTGATTGGCAAATGC
RM 000182 ATTGATCTCTCCATGTCTACTTGTTGTTTTACTAGGTCAAGGATCTCGTGAAATTGATTGGCAAATGC
PI 511381 ATTGATCTCTCCATGTCTACTTGTTGTTTTACTAGGTCAAGGATCTCGTGAAATTGATTGGCAAATGC
PI 486271 ATTGATCTCTCCATGTCTACTTGTTGTTTTACTAGGTCAAGGATCTCGTGAAATTGATTGGCAAATGC
PI 511367 ATTGATCTCTCCATGTCTACTTGTTGTTTTACTAGGTCAAGGATCTCGTGAAATTGATTGGCAAATGC
Chinese Spring ATTGATCTCTCCATGTCTACTTGTTGTTTTACTAGGTCAAGGATCTCGTGAAATTGATTGGCAAATGC
Hanzhongbai ATTGATCTCTCCATGTCTACTTGTTGTTTTACTAGGTCAAGGATCTCGTGAAATTGATTGGCAAATGC
Hongtoumai ATTGATCTCTCCATGTCTACTTGTTGTTTTACTAGGTCAAGGATCTCGTGAAATTGATTGGCAAATGC
Hongyouzi ATTGATCTCTCCATGTCTACTTGTTGTTTTACTAGGTCAAGGATCTCGTGAAATTGATTGGCAAATGC
Zijiehong ATTGATCTCTCCATGTCTACTTGTTGTTTTACTAGGTCAAGGATCTCGTGAAATTGATTGGCAAATGC
Baihuamai ATTGATCTCTCCATGTCTACTTGTTGTTTTACTAGGTCAAGGATCTCGTGAAATTGATTGGCAAATGC
Huixianhong ATTGATCTCTCCATGTCTACTTGTTGTTTTACTAGGTCAAGGATCTCGTGAAATTGATTGGCAAATGC
Baimangmai ATTGATCTCTCCATGTCTACTTGTTGTTTTACTAGGTCAAGGATCTCGTGAAATTGATTGGCAAATGC

```
                 9,250     9,260     9,270     9,280     9,290     9,300     9,310
Hulutou          GCTACAAATTGATCAAAGGGACTTGCGCAGGCTTACATTACCTTCACAAGGGCCGTGCAGGTTGTCCA
Baihulu          GCTACAAATTGATCAAAGGGACTTGCGCAGGCTTACATTACCTTCACAAGGGCCGTGCAGGTTGTCCA
Chiyacao         GCTACAAATTGATCAAAGGGACTTGCGCAGGCTTACATTACCTTCACAAGGGCCGTGCAGGTTGTCCA
Hongmangmai      GCTACAAATTGATCAAAGGGACTTGCGCAGGCTTACATTACCTTCACAAGGGCCGTGCAGGTTGTCCA
Hongmai          GCTACAAATTGATCAAAGGGACTTGCGCAGGCTTACATTACCTTCACAAGGGCCGTGCAGGTTGTCCA
Wangshuibai      GCTACAAATTGATCAAAGGGACTTGCGCAGGCTTACATTACCTTCACAAGGGCCGTGCAGGTTGTCCA
Shi 4185         GCTACAAATTGATCAAAGGGACTTGCGCAGGCTTACATTACCTTCACAAGGGCCGTGCAGGTTGTCCA
Mazhamai         GCTACAAATTGATCAAAGGGACTTGCGCAGGCTTACATTACCTTCACAAGGGCCGTGCAGGTTGTCCA
Xiaofoshou       GCTACAAATTGATCAAAGGGACTTGCGCAGGCTTACATTACCTTCACAAGGGCCGTGCAGGTTGTCCA
Aikang 58        GCTACAAATTGATCAAAGGGACTTGCGCAGGCTTACATTACCTTCACAAGGGCCGTGCAGGTTGTCCA
AL8/78           GTTACAAATTGATCAAAGGGACTTGCGCAGGCTTAGATTACCTTCACAAGGGCCGTGCAGGTTGTCCA
PI 431602        GTTACAAATTGATCAAAGGGACTTGCGCAGGCTTAGATTACCTTCACAAGGGCCGTGCAGGTTGTCCA
PI 486274        GTTACAAATTGATCAAAGGGACTTGCGCAGGCTTAGATTACCTTCACAAGGGCCGTGCAGGTTGTCCA
RM 000182        GTTACAAATTGATCAAAGGGACTTGCGCAGGCTTAGATTACCTTCACAAGGGCCGTGCAGGTTGTCCA
PI 511381        GTTACAAATTGATCAAAGGGACTTGCGCAGGCTTAGATTACCTTCACAAGGGCCGTGCAGGTTGTCCA
PI 486271        GTTACAAATTGATCAAAGGGACTTGCGCAGGCTTAGATTACCTTCACAAGGGCCGTGCAGGTTGTCCA
PI 511367        GTTACAAATTGATCAAAGGGACTTGCGCAGGCTTAGATTACCTTCACAAGGGCCGTGCAGGTTGTCCA
Chinese Spring   GTTACAAATTGATCAAAGGGACTTGCGCAGGCTTAGATTACCTTCACAAGGGCCGTGCAGGTTGTCCA
Hanzhongbai      GTTACAAATTGATCAAAGGGACTTGCGCAGGCTTAGATTACCTTCACAAGGGCCGTGCAGGTTGTCCA
Hongtoumai       GTTACAAATTGATCAAAGGGACTTGCGCAGGCTTAGATTACCTTCACAAGGGCCGTGCAGGTTGTCCA
Hongyouzi        GTTACAAATTGATCAAAGGGACTTGCGCAGGCTTAGATTACCTTCACAAGGGCCGTGCAGGTTGTCCA
Zijiehong        GTTACAAATTGATCAAAGGGACTTGCGCAGGCTTAGATTACCTTCACAAGGGCCGTGCAGGTTGTCCA
Baihuamai        GTTACAAATTGATCAAAGGGACTTGCGCAGGCTTAGATTACCTTCACAAGGGCCGTGCAGGTTGTCCA
Huixianhong      GTTACAAATTGATCAAAGGGACTTGCGCAGGCTTAGATTACCTTCACAAGGGCCGTGCAGGTTGTCCA
Baimangmai       GTTACAAATTGATCAAAGGGACTTGCGCAGGCTTAGATTACCTTCACAAGGGCCGTGCAGGTTGTCCA
```

```
                    9,320     9,330     9,340     9,350     9,360     9,370     9,380
Hulutou          ATTGTTCATTTGAATTTAAGCCCGTCAAATGTATTGCTGGACCACAAC-TACATACCACGCATCACAG
Baihulu          ATTGTTCATTTGAATTTAAGCCCGTCAAATGTATTGCTGGACCACAAC-TACATACCACGCATCACAG
Chiyacao         ATTGTTCATTTGAATTTAAGCCCGTCAAATGTATTGCTGGACCACAAC-TACATACCACGCATCACAG
Hongmangmai      ATTGTTCATTTGAATTTAAGCCCGTCAAATGTATTGCTGGACCACAAC-TACATACCACGCATCACAG
Hongmai          ATTGTTCATTTGAATTTAAGCCCGTCAAATGTATTGCTGGACCACAAC-TACATACCACGCATCACAG
Wangshuibai      ATTGTTCATTTGAATTTAAGCCCGTCAAATGTATTGCTGGACCACAAC-TACATACCACGCATCACAG
Shi 4185         ATTGTTCATTTGAATTTAAGCCCGTCAAATGTATTGCTGGACCACAAC-TACATACCACGCATCACAG
Mazhamai         ATTGTTCATTTGAATTTAAGCCCGTCAAATGTATTGCTGGACCACAAC-TACATACCACGCATCACAG
Xiaofoshou       ATTGTTCATTTGAATTTAAGCCCGTCAAATGTATTGCTGGACCACAAC-TACATACCACGCATCACAG
Aikang 58        ATTGTTCATTTGAATTTAAGCCCGTCAAATGTATTGCTGGACCACAAC-TACATACCACGCATCACAG
AL8/78           ATTGTTCATTTGAATTTAAGCCCGTCAAATGTATTGCTGGACCACAAC-TACATACCACGCATCACAG
PI 431602        ATTGTTCATTTGAATTTAAGCCCGTCAAATGTATTGCTGGACCACAAC-TACATACCACGCATCACAG
PI 486274        ATTGTTCATTTGAATTTAAGCCCGTCAAATGTATTGCTGGACCACAACTTACATACCACGCATCACAG
RM 000182        ATTGTTCATTTGAATTTAAGCCCGTCAAATGTATTGCTGGACCACAAC-TACATACCACGCATCACAG
PI 511381        ATTGTTCATTTGAATTTAAGCCCGTCAAATGTATTGCTGGACCACAAC-TACATACCACGCATCACAG
PI 486271        ATTGTTCATTTGAATTTAAGCCCGTCAAATGTATTGCTGGACCACAAC-TACATACCACGCATCACAG
PI 511367        ATTGTTCATTTGAATTTAAGCCCGTCAAATGTATTGCTGGACCACAAC-TACATACCACGCATCACAG
Chinese Spring   ATTGTTCATTTGAATTTAAGCCCGTCAAATGTATTGCTGGACCACAAC-TACATACCACGCATCACAG
Hanzhongbai      ATTGTTCATTTGAATTTAAGCCCGTCAAATGTATTGCTGGACCACAAC-TACATACCACGCATCACAG
Hongtoumai       ATTGTTCATTTGAATTTAAGCCCGTCAAATGTATTGCTGGACCACAAC-TACATACCACGCATCACAG
Hongyouzi        ATTGTTCATTTGAATTTAAGCCCGTCAAATGTATTGCTGGACCACAAC-TACATACCACGCATCACAG
Zijiehong        ATTGTTCATTTGAATTTAAGCCCGTCAAATGTATTGCTGGACCACAAC-TACATACCACGCATCACAG
Baihuamai        ATTGTTCATTTGAATTTAAGCCCGTCAAATGTATTGCTGGACCACAAC-TACATACCACGCATCACAG
Huixianhong      ATTGTTCATTTGAATTTAAGCCCGTCAAATGTATTGCTGGACCACAAC-TACATACCACGCATCACAG
Baimangmai       ATTGTTCATTTGAATTTAAGCCCGTCAAATGTATTGCTGGACCACAAC-TACATACCACGCATCACAG
```

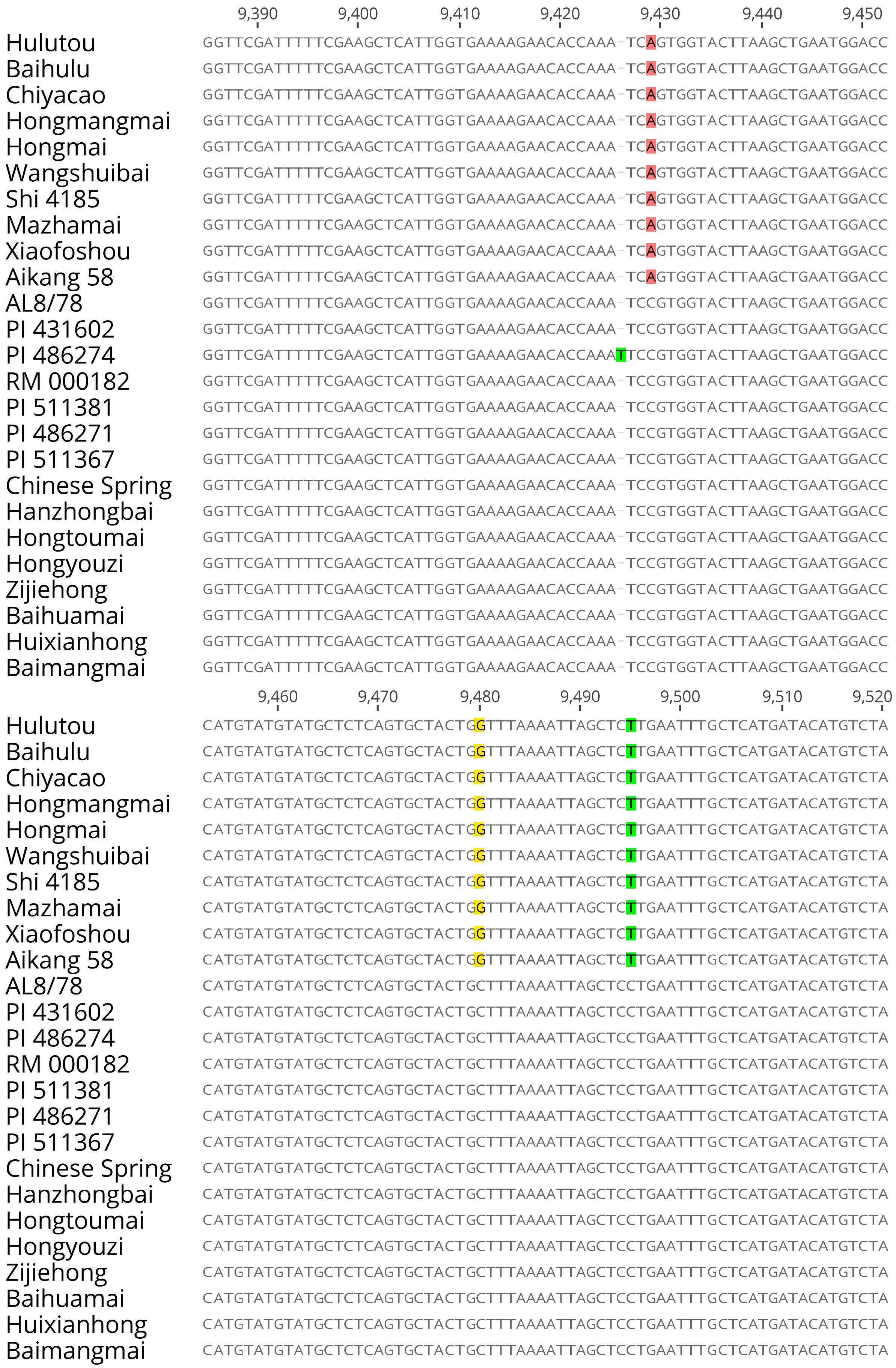
9,390 9,400 9,410 9,420 9,430 9,440 9,450
Hulutou GGTTCGATTTTTCGAAGCTCATTGGTGAAAAGAACACCAAA-TCAGTGGTACTTAAGCTGAATGGACC
Baihulu GGTTCGATTTTTCGAAGCTCATTGGTGAAAAGAACACCAAA-TCAGTGGTACTTAAGCTGAATGGACC
Chiyacao GGTTCGATTTTTCGAAGCTCATTGGTGAAAAGAACACCAAA-TCAGTGGTACTTAAGCTGAATGGACC
Hongmangmai GGTTCGATTTTTCGAAGCTCATTGGTGAAAAGAACACCAAA-TCAGTGGTACTTAAGCTGAATGGACC
Hongmai GGTTCGATTTTTCGAAGCTCATTGGTGAAAAGAACACCAAA-TCAGTGGTACTTAAGCTGAATGGACC
Wangshuibai GGTTCGATTTTTCGAAGCTCATTGGTGAAAAGAACACCAAA-TCAGTGGTACTTAAGCTGAATGGACC
Shi 4185 GGTTCGATTTTTCGAAGCTCATTGGTGAAAAGAACACCAAA-TCAGTGGTACTTAAGCTGAATGGACC
Mazhamai GGTTCGATTTTTCGAAGCTCATTGGTGAAAAGAACACCAAA-TCAGTGGTACTTAAGCTGAATGGACC
Xiaofoshou GGTTCGATTTTTCGAAGCTCATTGGTGAAAAGAACACCAAA-TCAGTGGTACTTAAGCTGAATGGACC
Aikang 58 GGTTCGATTTTTCGAAGCTCATTGGTGAAAAGAACACCAAA-TCAGTGGTACTTAAGCTGAATGGACC
AL8/78 GGTTCGATTTTTCGAAGCTCATTGGTGAAAAGAACACCAAA-TCCGTGGTACTTAAGCTGAATGGACC
PI 431602 GGTTCGATTTTTCGAAGCTCATTGGTGAAAAGAACACCAAA-TCCGTGGTACTTAAGCTGAATGGACC
PI 486274 GGTTCGATTTTTCGAAGCTCATTGGTGAAAAGAACACCAAATTCCGTGGTACTTAAGCTGAATGGACC
RM 000182 GGTTCGATTTTTCGAAGCTCATTGGTGAAAAGAACACCAAA-TCCGTGGTACTTAAGCTGAATGGACC
PI 511381 GGTTCGATTTTTCGAAGCTCATTGGTGAAAAGAACACCAAA-TCCGTGGTACTTAAGCTGAATGGACC
PI 486271 GGTTCGATTTTTCGAAGCTCATTGGTGAAAAGAACACCAAA-TCCGTGGTACTTAAGCTGAATGGACC
PI 511367 GGTTCGATTTTTCGAAGCTCATTGGTGAAAAGAACACCAAA-TCCGTGGTACTTAAGCTGAATGGACC
Chinese Spring GGTTCGATTTTTCGAAGCTCATTGGTGAAAAGAACACCAAA-TCCGTGGTACTTAAGCTGAATGGACC
Hanzhongbai GGTTCGATTTTTCGAAGCTCATTGGTGAAAAGAACACCAAA-TCCGTGGTACTTAAGCTGAATGGACC
Hongtoumai GGTTCGATTTTTCGAAGCTCATTGGTGAAAAGAACACCAAA-TCCGTGGTACTTAAGCTGAATGGACC
Hongyouzi GGTTCGATTTTTCGAAGCTCATTGGTGAAAAGAACACCAAA-TCCGTGGTACTTAAGCTGAATGGACC
Zijiehong GGTTCGATTTTTCGAAGCTCATTGGTGAAAAGAACACCAAA-TCCGTGGTACTTAAGCTGAATGGACC
Baihuamai GGTTCGATTTTTCGAAGCTCATTGGTGAAAAGAACACCAAA-TCCGTGGTACTTAAGCTGAATGGACC
Huixianhong GGTTCGATTTTTCGAAGCTCATTGGTGAAAAGAACACCAAA-TCCGTGGTACTTAAGCTGAATGGACC
Baimangmai GGTTCGATTTTTCGAAGCTCATTGGTGAAAAGAACACCAAA-TCCGTGGTACTTAAGCTGAATGGACC
9,460 9,470 9,480 9,490 9,500 9,510 9,520
Hulutou CATGTATGTATGCTCTCAGTGCTACTGGTTTAAAATTAGCTCTTGAATTTGCTCATGATACATGTCTA
Baihulu CATGTATGTATGCTCTCAGTGCTACTGGTTTAAAATTAGCTCTTGAATTTGCTCATGATACATGTCTA
Chiyacao CATGTATGTATGCTCTCAGTGCTACTGGTTTAAAATTAGCTCTTGAATTTGCTCATGATACATGTCTA
Hongmangmai CATGTATGTATGCTCTCAGTGCTACTGGTTTAAAATTAGCTCTTGAATTTGCTCATGATACATGTCTA
Hongmai CATGTATGTATGCTCTCAGTGCTACTGGTTTAAAATTAGCTCTTGAATTTGCTCATGATACATGTCTA
Wangshuibai CATGTATGTATGCTCTCAGTGCTACTGGTTTAAAATTAGCTCTTGAATTTGCTCATGATACATGTCTA
Shi 4185 CATGTATGTATGCTCTCAGTGCTACTGGTTTAAAATTAGCTCTTGAATTTGCTCATGATACATGTCTA
Mazhamai CATGTATGTATGCTCTCAGTGCTACTGGTTTAAAATTAGCTCTTGAATTTGCTCATGATACATGTCTA
Xiaofoshou CATGTATGTATGCTCTCAGTGCTACTGGTTTAAAATTAGCTCTTGAATTTGCTCATGATACATGTCTA
Aikang 58 CATGTATGTATGCTCTCAGTGCTACTGGTTTAAAATTAGCTCTTGAATTTGCTCATGATACATGTCTA
AL8/78 CATGTATGTATGCTCTCAGTGCTACTGCTTTAAAATTAGCTCCTGAATTTGCTCATGATACATGTCTA
PI 431602 CATGTATGTATGCTCTCAGTGCTACTGCTTTAAAATTAGCTCCTGAATTTGCTCATGATACATGTCTA
PI 486274 CATGTATGTATGCTCTCAGTGCTACTGCTTTAAAATTAGCTCCTGAATTTGCTCATGATACATGTCTA
RM 000182 CATGTATGTATGCTCTCAGTGCTACTGCTTTAAAATTAGCTCCTGAATTTGCTCATGATACATGTCTA
PI 511381 CATGTATGTATGCTCTCAGTGCTACTGCTTTAAAATTAGCTCCTGAATTTGCTCATGATACATGTCTA
PI 486271 CATGTATGTATGCTCTCAGTGCTACTGCTTTAAAATTAGCTCCTGAATTTGCTCATGATACATGTCTA
PI 511367 CATGTATGTATGCTCTCAGTGCTACTGCTTTAAAATTAGCTCCTGAATTTGCTCATGATACATGTCTA
Chinese Spring CATGTATGTATGCTCTCAGTGCTACTGCTTTAAAATTAGCTCCTGAATTTGCTCATGATACATGTCTA
Hanzhongbai CATGTATGTATGCTCTCAGTGCTACTGCTTTAAAATTAGCTCCTGAATTTGCTCATGATACATGTCTA
Hongtoumai CATGTATGTATGCTCTCAGTGCTACTGCTTTAAAATTAGCTCCTGAATTTGCTCATGATACATGTCTA
Hongyouzi CATGTATGTATGCTCTCAGTGCTACTGCTTTAAAATTAGCTCCTGAATTTGCTCATGATACATGTCTA
Zijiehong CATGTATGTATGCTCTCAGTGCTACTGCTTTAAAATTAGCTCCTGAATTTGCTCATGATACATGTCTA
Baihuamai CATGTATGTATGCTCTCAGTGCTACTGCTTTAAAATTAGCTCCTGAATTTGCTCATGATACATGTCTA
Huixianhong CATGTATGTATGCTCTCAGTGCTACTGCTTTAAAATTAGCTCCTGAATTTGCTCATGATACATGTCTA
Baimangmai CATGTATGTATGCTCTCAGTGCTACTGCTTTAAAATTAGCTCCTGAATTTGCTCATGATACATGTCTA

```
                         9,530       9,540       9,550       9,560       9,570       9,580
Hulutou          TG--------TTTTGGAAAAATGTCTTATGTTTTGTGGCAGATCGGTTTAGCTAGTCTTTTTTCTGTG
Baihulu          TG--------TTTTGGAAAAATGTCTTATGTTTTGTGGCAGATCGGTTTAGCTAGTCTTTTTTCTGTG
Chiyacao         TG--------TTTTGGAAAAATGTCTTATGTTTTGTGGCAGATCGGTTTAGCTAGTCTTTTTTCTGTG
Hongmangmai      TG--------TTTTGGAAAAATGTCTTATGTTTTGTGGCAGATCGGTTTAGCTAGTCTTTTTTCTGTG
Hongmai          TG--------TTTTGGAAAAATGTCTTATGTTTTGTGGCAGATCGGTTTAGCTAGTCTTTTTTCTGTG
Wangshuibai      TG--------TTTTGGAAAAATGTCTTATGTTTTGTGGCAGATCGGTTTAGCTAGTCTTTTTTCTGTG
Shi 4185         TG--------TTTTGGAAAAATGTCTTATGTTTTGTGGCAGATCGGTTTAGCTAGTCTTTTTTCTGTG
Mazhamai         TG--------TTTTGGAAAAATGTCTTATGTTTTGTGGCAGATCGGTTTAGCTAGTCTTTTTTCTGTG
Xiaofoshou       TG--------TTTTGGAAAAATGTCTTATGTTTTGTGGCAGATCGGTTTAGCTAGTCTTTTTTCTGTG
Aikang 58        TG--------TTTTGGAAAAATGTCTTATGTTTTGTGGCAGATCGGTTTAGCTAGTCTTTTTTCTGTG
AL8/78           TGTTTTATTTTTTTGGAAAAATGTCTTATGTTTTGTGGCTGCTCGGTTTAGCTAGTC-TTTTTCTGTG
PI 431602        TGTTTTATTTTTTTGGAAAAATGTCTTATGTTTTGTGGCTGCTCGGTTTAGCTAGTC-TTTTTCTGTG
PI 486274        TGTTTTATTTTTTTGGAAAAATGTCTTATGTTTTGTGGCTGCTCGGTTTAGCTAGTC-TTTTTCTGTG
RM 000182        TGTTTTATTTTTTTGGAAAAATGTCTTATGTTTTGTGGCTGCTCGGTTTAGCTAGTC-TTTTTCTGTG
PI 511381        TGTTTTATTTTTTTGGAAAAATGTCTTATGTTTTGTGGCTGCTCGGTTTAGCTAGTC-TTTTTCTGTG
PI 486271        TGTTTTATTTTTTTGGAAAAATGTCTTATGTTTTGTGGCTGCTCGGTTTAGCTAGTC-TTTTTCTGTG
PI 511367        TGTTTTATTTTTTTGGAAAAATGTCTTATGTTTTGTGGCTGCTCGGTTTAGCTAGTC-TTTTTCTGTG
Chinese Spring   TGTTTTATTTTTTTGGAAAAATGTCTTATGTTTTGTGGCTGCTCGGTTTAGCTAGTC-TTTTTCTGTG
Hanzhongbai      TGTTTTATTTTTTTGGAAAAATGTCTTATGTTTTGTGGCTGCTCGGTTTAGCTAGTC-TTTTTCTGTG
Hongtoumai       TGTTTTATTTTTTTGGAAAAATGTCTTATGTTTTGTGGCTGCTCGGTTTAGCTAGTC-TTTTTCTGTG
Hongyouzi        TGTTTTATTTTTTTGGAAAAATGTCTTATGTTTTGTGGCTGCTCGGTTTAGCTAGTC-TTTTTCTGTG
Zijiehong        TGTTTTATTTTTTTGGAAAAATGTCTTATGTTTTGTGGCTGCTCGGTTTAGCTAGTC-TTTTTCTGTG
Baihuamai        TGTTTTATTTTTTTGGAAAAATGTCTTATGTTTTGTGGCTGCTCGGTTTAGCTAGTC-TTTTTCTGTG
Huixianhong      TGTTTTATTTTTTTGGAAAAATGTCTTATGTTTTGTGGCTGCTCGGTTTAGCTAGTC-TTTTTCTGTG
Baimangmai       TGTTTTATTTTTTTGGAAAAATGTCTTATGTTTTGTGGCTGCTCGGTTTAGCTAGTC-TTTTTCTGTG

                 9,590       9,600       9,610       9,620       9,630       9,640       9,650
Hulutou          AGAAACGTTTAGCAAGTCTATTGACCGTAATATGCTTTAATTAAACTGATGTGTTCATATATTTTTGC
Baihulu          AGAAACGTTTAGCAAGTCTATTGACCGTAATATGCTTTAATTAAACTGATGTGTTCATATATTTTTGC
Chiyacao         AGAAACGTTTAGCAAGTCTATTGACCGTAATATGCTTTAATTAAACTGATGTGTTCATATATTTTTGC
Hongmangmai      AGAAACGTTTAGCAAGTCTATTGACCGTAATATGCTTTAATTAAACTGATGTGTTCATATATTTTTGC
Hongmai          AGAAACGTTTAGCAAGTCTATTGACCGTAATATGCTTTAATTAAACTGATGTGTTCATATATTTTTGC
Wangshuibai      AGAAACGTTTAGCAAGTCTATTGACCGTAATATGCTTTAATTAAACTGATGTGTTCATATATTTTTGC
Shi 4185         AGAAACGTTTAGCAAGTCTATTGACCGTAATATGCTTTAATTAAACTGATGTGTTCATATATTTTTGC
Mazhamai         AGAAACGTTTAGCAAGTCTATTGACCGTAATATGCTTTAATTAAACTGATGTGTTCATATATTTTTGC
Xiaofoshou       AGAAACGTTTAGCAAGTCTATTGACCGTAATATGCTTTAATTAAACTGATGTGTTCATATATTTTTGC
Aikang 58        AGAAACGTTTAGCAAGTCTATTGACCGTAATATGCTTTAATTAAACTGATGTGTTCATATATTTTTGC
AL8/78           AGAAACGTTTAGCTACTCTATTGATCGTAATATGCTTTAATTAAACTGATGTGTTCATATATTTTTGC
PI 431602        AGAAACGTTTAGCTACTCTATTGATCGTAATATGCTTTAATTAAACTGATGTGTTCATATATTTTTGC
PI 486274        AGAAACGTTTAGCTACTCTATTGATCGTAATATGCTTTAATTAAACTGATGTGTTCATATATTTTTGC
RM 000182        AGAAACGTTTAGCTACTCTATTGATCGTAATATGCTTTAATTAAACTGATGTGTTCATATATTTTTGC
PI 511381        AGAAACGTTTAGCTACTCTATTGATCGTAATATGCTTTAATTAAACTGATGTGTTCATATATTTTTGC
PI 486271        AGAAACGTTTAGCTACTCTATTGATCGTAATATGCTTTAATTAAACTGATGTGTTCATATATTTTTGC
PI 511367        AGAAACGTTTAGCTACTCTATTGATCGTAATATGCTTTAATTAAACTGATGTGTTCATATATTTTTGC
Chinese Spring   AGAAACGTTTAGCTACTCTATTGATCGTAATATGCTTTAATTAAACTGATGTGTTCATATATTTTTGC
Hanzhongbai      AGAAACGTTTAGCTACTCTATTGATCGTAATATGCTTTAATTAAACTGATGTGTTCATATATTTTTGC
Hongtoumai       AGAAACGTTTAGCTACTCTATTGATCGTAATATGCTTTAATTAAACTGATGTGTTCATATATTTTTGC
Hongyouzi        AGAAACGTTTAGCTACTCTATTGATCGTAATATGCTTTAATTAAACTGATGTGTTCATATATTTTTGC
Zijiehong        AGAAACGTTTAGCTACTCTATTGATCGTAATATGCTTTAATTAAACTGATGTGTTCATATATTTTTGC
Baihuamai        AGAAACGTTTAGCTACTCTATTGATCGTAATATGCTTTAATTAAACTGATGTGTTCATATATTTTTGC
Huixianhong      AGAAACGTTTAGCTACTCTATTGATCGTAATATGCTTTAATTAAACTGATGTGTTCATATATTTTTGC
Baimangmai       AGAAACGTTTAGCTACTCTATTGATCGTAATATGCTTTAATTAAACTGATGTGTTCATATATTTTTGC
```

```
                 9,660     9,670     9,680     9,690     9,700     9,710     9,720
Hulutou          AGAGCGTACCTGCCACCGGATTTCTTCCATTCGAAGGGTACTGATCTTAAATATCTTGCTACGGTAGA
Baihulu          AGAGCGTACCTGCCACCGGATTTCTTCCATTCGAAGGGTACTGATCTTAAATATCTTGCTACGGTAGA
Chiyacao         AGAGCGTACCTGCCACCGGATTTCTTCCATTCGAAGGGTACTGATCTTAAATATCTTGCTACGGTAGA
Hongmangmai      AGAGCGTACCTGCCACCGGATTTCTTCCATTCGAAGGGTACTGATCTTAAATATCTTGCTACGGTAGA
Hongmai          AGAGCGTACCTGCCACCGGATTTCTTCCATTCGAAGGGTACTGATCTTAAATATCTTGCTACGGTAGA
Wangshuibai      AGAGCGTACCTGCCACCGGATTTCTTCCATTCGAAGGGTACTGATCTTAAATATCTTGCTACGGTAGA
Shi 4185         AGAGCGTACCTGCCACCGGATTTCTTCCATTCGAAGGGTACTGATCTTAAATATCTTGCTACGGTAGA
Mazhamai         AGAGCGTACCTGCCACCGGATTTCTTCCATTCGAAGGGTACTGATCTTAAATATCTTGCTACGGTAGA
Xiaofoshou       AGAGCGTACCTGCCACCGGATTTCTTCCATTCGAAGGGTACTGATCTTAAATATCTTGCTACGGTAGA
Aikang 58        AGAGCGTACCTGCCACCGGATTTCTTCCATTCGAAGGGTACTGATCTTAAATATCTTGCTACGGTAGA
AL8/78           AGAGCGTACCTGCCACCGGATTTCTTCCATTCAAAGGGTACTGATCTTAAATATCTTGCTACGGTAGA
PI 431602        AGAGCGTACCTGCCACCGGATTTCTTCCATTCAAAGGGTACTGATCTTAAATATCTTGCTACGGTAGA
PI 486274        AGAGCGTACCTGCCACCGGATTTCTTCCATTCAAAGGGTACTGATCTTAAATATCTTGCTACGGTAGA
RM 000182        AGAGCGTACCTGCCACCGGATTTCTTCCATTCAAAGGGTACTGATCTTAAATATCTTGCTACGGTAGA
PI 511381        AGAGCGTACCTGCCACCGGATTTCTTCCATTCAAAGGGTACTGATCTTAAATATCTTGCTACGGTAGA
PI 486271        AGAGCGTACCTGCCACCGGATTTCTTCCATTCAAAGGGTACTGATCTTAAATATCTTGCTACGGTAGA
PI 511367        AGAGCGTACCTGCCACCGGATTTCTTCCATTCAAAGGGTACTGATCTTAAATATCTTGCTACGGTAGA
Chinese Spring   AGAGCGTACCTGCCACCGGATTTCTTCCATTCAAAGGGTACTGATCTTAAATATCTTGCTACGGTAGA
Hanzhongbai      AGAGCGTACCTGCCACCGGATTTCTTCCATTCAAAGGGTACTGATCTTAAATATCTTGCTACGGTAGA
Hongtoumai       AGAGCGTACCTGCCACCGGATTTCTTCCATTCAAAGGGTACTGATCTTAAATATCTTGCTACGGTAGA
Hongyouzi        AGAGCGTACCTGCCACCGGATTTCTTCCATTCAAAGGGTACTGATCTTAAATATCTTGCTACGGTAGA
Zijiehong        AGAGCGTACCTGCCACCGGATTTCTTCCATTCAAAGGGTACTGATCTTAAATATCTTGCTACGGTAGA
Baihuamai        AGAGCGTACCTGCCACCGGATTTCTTCCATTCAAAGGGTACTGATCTTAAATATCTTGCTACGGTAGA
Huixianhong      AGAGCGTACCTGCCACCGGATTTCTTCCATTCAAAGGGTACTGATCTTAAATATCTTGCTACGGTAGA
Baimangmai       AGAGCGTACCTGCCACCGGATTTCTTCCATTCAAAGGGTACTGATCTTAAATATCTTGCTACGGTAGA

                   9,730     9,740     9,750     9,760     9,770     9,780     9,790
Hulutou          TATATACAGCTTGGGTCTTATGATTTTAGAAATCGCAACACAACAAGAGATCAAAGGCATCCATGGAG
Baihulu          TATATACAGCTTGGGTCTTATGATTTTAGAAATCGCAACACAACAAGAGATCAAAGGCATCCATGGAG
Chiyacao         TATATACAGCTTGGGTCTTATGATTTTAGAAATCGCAACACAACAAGAGATCAAAGGCATCCATGGAG
Hongmangmai      TATATACAGCTTGGGTCTTATGATTTTAGAAATCGCAACACAACAAGAGATCAAAGGCATCCATGGAG
Hongmai          TATATACAGCTTGGGTCTTATGATTTTAGAAATCGCAACACAACAAGAGATCAAAGGCATCCATGGAG
Wangshuibai      TATATACAGCTTGGGTCTTATGATTTTAGAAATCGCAACACAACAAGAGATCAAAGGCATCCATGGAG
Shi 4185         TATATACAGCTTGGGTCTTATGATTTTAGAAATCGCAACACAACAAGAGATCAAAGGCATCCATGGAG
Mazhamai         TATATACAGCTTGGGTCTTATGATTTTAGAAATCGCAACACAACAAGAGATCAAAGGCATCCATGGAG
Xiaofoshou       TATATACAGCTTGGGTCTTATGATTTTAGAAATCGCAACACAACAAGAGATCAAAGGCATCCATGGAG
Aikang 58        TATATACAGCTTGGGTCTTATGATTTTAGAAATCGCAACACAACAAGAGATCAAAGGCATCCATGGAG
AL8/78           TATATACAGCTTGGGTCTTATGATTTTAGAAATCGCAACACAACAAGAGATCAAAGGCATCCATGGAG
PI 431602        TATATACAGCTTGGGTCTTATGATTTTAGAAATCGCAACACAACAAGAGATCAAAGGCATCCATGGAG
PI 486274        TATATACAGCTTGGGTCTTATGATTTTAGAAATCGCAACACAACAAGAGATCAAAGGCATCCATGGAG
RM 000182        TATATACAGCTTGGGTCTTATGATTTTAGAAATCGCAACACAACAAGAGATCAAAGGCATCCATGGAG
PI 511381        TATATACAGCTTGGGTCTTATGATTTTAGAAATCGCAACACAACAAGAGATCAAAGGCATCCATGGAG
PI 486271        TATATACAGCTTGGGTCTTATGATTTTAGAAATCGCAACACAACAAGAGATCAAAGGCATCCATGGAG
PI 511367        TATATACAGCTTGGGTCTTATGATTTTAGAAATCGCAACACAACAAGAGATCAAAGGCATCCATGGAG
Chinese Spring   TATATACAGCTTGGGTCTTATGATTTTAGAAATCGCAACACAACAAGAGATCAAAGGCATCCATGGAG
Hanzhongbai      TATATACAGCTTGGGTCTTATGATTTTAGAAATCGCAACACAACAAGAGATCAAAGGCATCCATGGAG
Hongtoumai       TATATACAGCTTGGGTCTTATGATTTTAGAAATCGCAACACAACAAGAGATCAAAGGCATCCATGGAG
Hongyouzi        TATATACAGCTTGGGTCTTATGATTTTAGAAATCGCAACACAACAAGAGATCAAAGGCATCCATGGAG
Zijiehong        TATATACAGCTTGGGTCTTATGATTTTAGAAATCGCAACACAACAAGAGATCAAAGGCATCCATGGAG
Baihuamai        TATATACAGCTTGGGTCTTATGATTTTAGAAATCGCAACACAACAAGAGATCAAAGGCATCCATGGAG
Huixianhong      TATATACAGCTTGGGTCTTATGATTTTAGAAATCGCAACACAACAAGAGATCAAAGGCATCCATGGAG
Baimangmai       TATATACAGCTTGGGTCTTATGATTTTAGAAATCGCAACACAACAAGAGATCAAAGGCATCCATGGAG
```

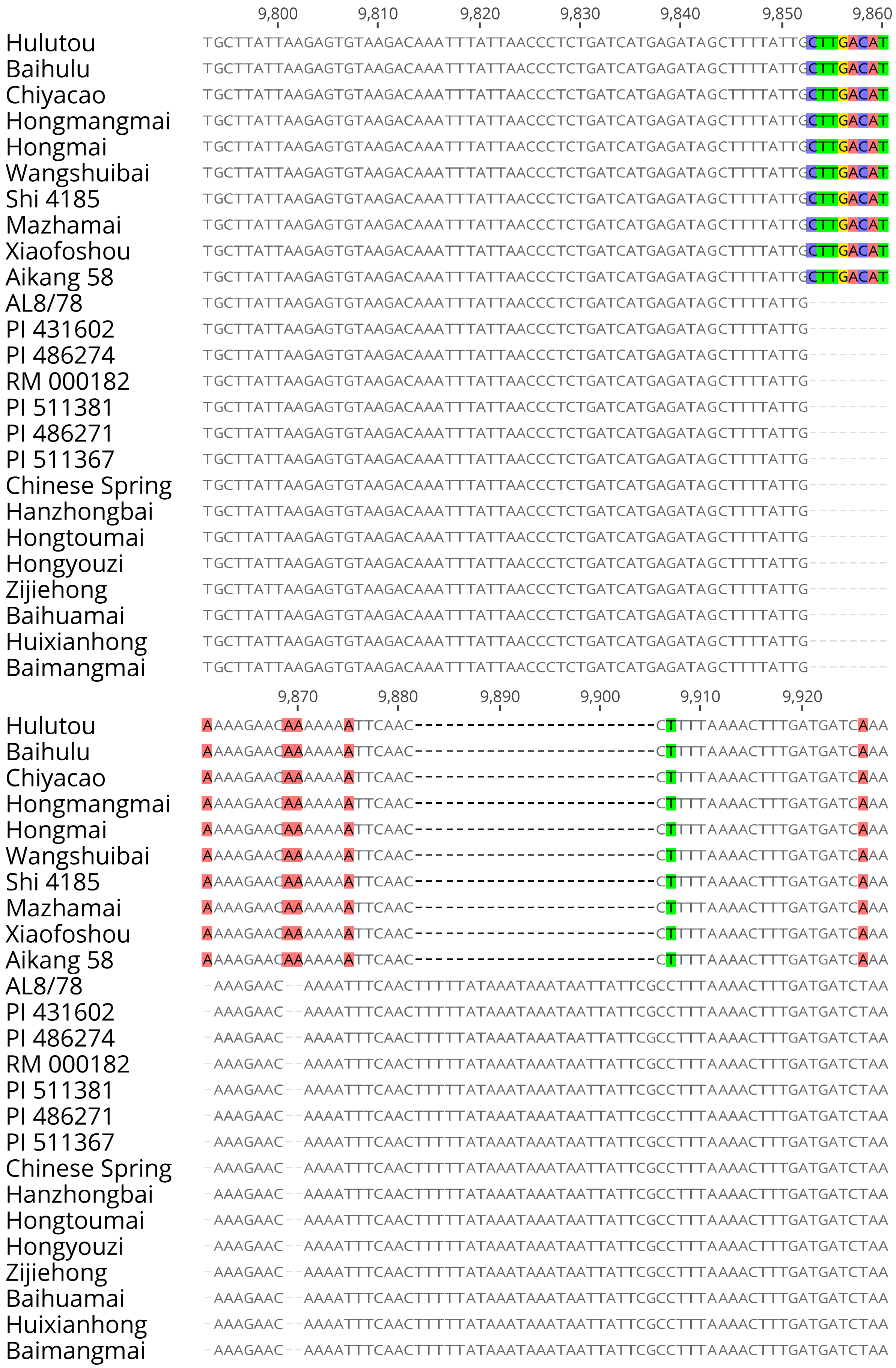

9,800 9,810 9,820 9,830 9,840 9,850 9,860
Hulutou TGCTTATTAAGAGTGTAAGACAAATTTATTAACCCTCTGATCATGAGATAGCTTTTATTGCTTGACAT
Baihulu TGCTTATTAAGAGTGTAAGACAAATTTATTAACCCTCTGATCATGAGATAGCTTTTATTGCTTGACAT
Chiyacao TGCTTATTAAGAGTGTAAGACAAATTTATTAACCCTCTGATCATGAGATAGCTTTTATTGCTTGACAT
Hongmangmai TGCTTATTAAGAGTGTAAGACAAATTTATTAACCCTCTGATCATGAGATAGCTTTTATTGCTTGACAT
Hongmai TGCTTATTAAGAGTGTAAGACAAATTTATTAACCCTCTGATCATGAGATAGCTTTTATTGCTTGACAT
Wangshuibai TGCTTATTAAGAGTGTAAGACAAATTTATTAACCCTCTGATCATGAGATAGCTTTTATTGCTTGACAT
Shi 4185 TGCTTATTAAGAGTGTAAGACAAATTTATTAACCCTCTGATCATGAGATAGCTTTTATTGCTTGACAT
Mazhamai TGCTTATTAAGAGTGTAAGACAAATTTATTAACCCTCTGATCATGAGATAGCTTTTATTGCTTGACAT
Xiaofoshou TGCTTATTAAGAGTGTAAGACAAATTTATTAACCCTCTGATCATGAGATAGCTTTTATTGCTTGACAT
Aikang 58 TGCTTATTAAGAGTGTAAGACAAATTTATTAACCCTCTGATCATGAGATAGCTTTTATTGCTTGACAT
AL8/78 TGCTTATTAAGAGTGTAAGACAAATTTATTAACCCTCTGATCATGAGATAGCTTTTATTG--------
PI 431602 TGCTTATTAAGAGTGTAAGACAAATTTATTAACCCTCTGATCATGAGATAGCTTTTATTG--------
PI 486274 TGCTTATTAAGAGTGTAAGACAAATTTATTAACCCTCTGATCATGAGATAGCTTTTATTG--------
RM 000182 TGCTTATTAAGAGTGTAAGACAAATTTATTAACCCTCTGATCATGAGATAGCTTTTATTG--------
PI 511381 TGCTTATTAAGAGTGTAAGACAAATTTATTAACCCTCTGATCATGAGATAGCTTTTATTG--------
PI 486271 TGCTTATTAAGAGTGTAAGACAAATTTATTAACCCTCTGATCATGAGATAGCTTTTATTG--------
PI 511367 TGCTTATTAAGAGTGTAAGACAAATTTATTAACCCTCTGATCATGAGATAGCTTTTATTG--------
Chinese Spring TGCTTATTAAGAGTGTAAGACAAATTTATTAACCCTCTGATCATGAGATAGCTTTTATTG--------
Hanzhongbai TGCTTATTAAGAGTGTAAGACAAATTTATTAACCCTCTGATCATGAGATAGCTTTTATTG--------
Hongtoumai TGCTTATTAAGAGTGTAAGACAAATTTATTAACCCTCTGATCATGAGATAGCTTTTATTG--------
Hongyouzi TGCTTATTAAGAGTGTAAGACAAATTTATTAACCCTCTGATCATGAGATAGCTTTTATTG--------
Zijiehong TGCTTATTAAGAGTGTAAGACAAATTTATTAACCCTCTGATCATGAGATAGCTTTTATTG--------
Baihuamai TGCTTATTAAGAGTGTAAGACAAATTTATTAACCCTCTGATCATGAGATAGCTTTTATTG--------
Huixianhong TGCTTATTAAGAGTGTAAGACAAATTTATTAACCCTCTGATCATGAGATAGCTTTTATTG--------
Baimangmai TGCTTATTAAGAGTGTAAGACAAATTTATTAACCCTCTGATCATGAGATAGCTTTTATTG--------
9,870 9,880 9,890 9,900 9,910 9,920
Hulutou AAAAGAACAAAAAAATTCAAC------------------------CTTTTAAAACTTTGATGATCAAA
Baihulu AAAAGAACAAAAAAATTCAAC------------------------CTTTTAAAACTTTGATGATCAAA
Chiyacao AAAAGAACAAAAAAATTCAAC------------------------CTTTTAAAACTTTGATGATCAAA
Hongmangmai AAAAGAACAAAAAAATTCAAC------------------------CTTTTAAAACTTTGATGATCAAA
Hongmai AAAAGAACAAAAAAATTCAAC------------------------CTTTTAAAACTTTGATGATCAAA
Wangshuibai AAAAGAACAAAAAAATTCAAC------------------------CTTTTAAAACTTTGATGATCAAA
Shi 4185 AAAAGAACAAAAAAATTCAAC------------------------CTTTTAAAACTTTGATGATCAAA
Mazhamai AAAAGAACAAAAAAATTCAAC------------------------CTTTTAAAACTTTGATGATCAAA
Xiaofoshou AAAAGAACAAAAAAATTCAAC------------------------CTTTTAAAACTTTGATGATCAAA
Aikang 58 AAAAGAACAAAAAAATTCAAC------------------------CTTTTAAAACTTTGATGATCAAA
AL8/78 -AAAGAAC--AAAATTTCAACTTTTTATAAATAAATAATTATTCGCCTTTAAAACTTTGATGATCTAA
PI 431602 -AAAGAAC--AAAATTTCAACTTTTTATAAATAAATAATTATTCGCCTTTAAAACTTTGATGATCTAA
PI 486274 -AAAGAAC--AAAATTTCAACTTTTTATAAATAAATAATTATTCGCCTTTAAAACTTTGATGATCTAA
RM 000182 -AAAGAAC--AAAATTTCAACTTTTTATAAATAAATAATTATTCGCCTTTAAAACTTTGATGATCTAA
PI 511381 -AAAGAAC--AAAATTTCAACTTTTTATAAATAAATAATTATTCGCCTTTAAAACTTTGATGATCTAA
PI 486271 -AAAGAAC--AAAATTTCAACTTTTTATAAATAAATAATTATTCGCCTTTAAAACTTTGATGATCTAA
PI 511367 -AAAGAAC--AAAATTTCAACTTTTTATAAATAAATAATTATTCGCCTTTAAAACTTTGATGATCTAA
Chinese Spring -AAAGAAC--AAAATTTCAACTTTTTATAAATAAATAATTATTCGCCTTTAAAACTTTGATGATCTAA
Hanzhongbai -AAAGAAC--AAAATTTCAACTTTTTATAAATAAATAATTATTCGCCTTTAAAACTTTGATGATCTAA
Hongtoumai -AAAGAAC--AAAATTTCAACTTTTTATAAATAAATAATTATTCGCCTTTAAAACTTTGATGATCTAA
Hongyouzi -AAAGAAC--AAAATTTCAACTTTTTATAAATAAATAATTATTCGCCTTTAAAACTTTGATGATCTAA
Zijiehong -AAAGAAC--AAAATTTCAACTTTTTATAAATAAATAATTATTCGCCTTTAAAACTTTGATGATCTAA
Baihuamai -AAAGAAC--AAAATTTCAACTTTTTATAAATAAATAATTATTCGCCTTTAAAACTTTGATGATCTAA
Huixianhong -AAAGAAC--AAAATTTCAACTTTTTATAAATAAATAATTATTCGCCTTTAAAACTTTGATGATCTAA
Baimangmai -AAAGAAC--AAAATTTCAACTTTTTATAAATAAATAATTATTCGCCTTTAAAACTTTGATGATCTAA

	9,930 · 9,940 · 9,950 · 9,960 · 9,970 · 9,980 · 9,990
Hulutou	TATACTCTACAGATAGAGGAAAACTGGAGGGA**G**GAGTCACAAATAACACGGCTGTATACCTCACTAGG
Baihulu	TATACTCTACAGATAGAGGAAAACTGGAGGGA**G**GAGTCACAAATAACACGGCTGTATACCTCACTAGG
Chiyacao	TATACTCTACAGATAGAGGAAAACTGGAGGGA**G**GAGTCACAAATAACACGGCTGTATACCTCACTAGG
Hongmangmai	TATACTCTACAGATAGAGGAAAACTGGAGGGA**G**GAGTCACAAATAACACGGCTGTATACCTCACTAGG
Hongmai	TATACTCTACAGATAGAGGAAAACTGGAGGGA**G**GAGTCACAAATAACACGGCTGTATACCTCACTAGG
Wangshuibai	TATACTCTACAGATAGAGGAAAACTGGAGGGA**G**GAGTCACAAATAACACGGCTGTATACCTCACTAGG
Shi 4185	TATACTCTACAGATAGAGGAAAACTGGAGGGA**G**GAGTCACAAATAACACGGCTGTATACCTCACTAGG
Mazhamai	TATACTCTACAGATAGAGGAAAACTGGAGGGA**G**GAGTCACAAATAACACGGCTGTATACCTCACTAGG
Xiaofoshou	TATACTCTACAGATAGAGGAAAACTGGAGGGA**G**GAGTCACAAATAACACGGCTGTATACCTCACTAGG
Aikang 58	TATACTCTACAGATAGAGGAAAACTGGAGGGA**G**GAGTCACAAATAACACGGCTGTATACCTCACTAGG
AL8/78	TATACTCTACAGATAGAGGAAAACTGGAGGGAAGAGTCACAAATAACACGGCTGTATACCTCACTAGG
PI 431602	TATACTCTACAGATAGAGGAAAACTGGAGGGAAGAGTCACAAATAACACGGCTGTATACCTCACTAGG
PI 486274	TATACTCTACAGATAGAGGAAAACTGGAGGGAAGAGTCACAAATAACACGGCTGTATACCTCACTAGG
RM 000182	TATACTCTACAGATAGAGGAAAACTGGAGGGAAGAGTCACAAATAACACGGCTGTATACCTCACTAGG
PI 511381	TATACTCTACAGATAGAGGAAAACTGGAGGGAAGAGTCACAAATAACACGGCTGTATACCTCACTAGG
PI 486271	TATACTCTACAGATAGAGGAAAACTGGAGGGAAGAGTCACAAATAACACGGCTGTATACCTCACTAGG
PI 511367	TATACTCTACAGATAGAGGAAAACTGGAGGGAAGAGTCACAAATAACACGGCTGTATACCTCACTAGG
Chinese Spring	TATACTCTACAGATAGAGGAAAACTGGAGGGAAGAGTCACAAATAACACGGCTGTATACCTCACTAGG
Hanzhongbai	TATACTCTACAGATAGAGGAAAACTGGAGGGAAGAGTCACAAATAACACGGCTGTATACCTCACTAGG
Hongtoumai	TATACTCTACAGATAGAGGAAAACTGGAGGGAAGAGTCACAAATAACACGGCTGTATACCTCACTAGG
Hongyouzi	TATACTCTACAGATAGAGGAAAACTGGAGGGAAGAGTCACAAATAACACGGCTGTATACCTCACTAGG
Zijiehong	TATACTCTACAGATAGAGGAAAACTGGAGGGAAGAGTCACAAATAACACGGCTGTATACCTCACTAGG
Baihuamai	TATACTCTACAGATAGAGGAAAACTGGAGGGAAGAGTCACAAATAACACGGCTGTATACCTCACTAGG
Huixianhong	TATACTCTACAGATAGAGGAAAACTGGAGGGAAGAGTCACAAATAACACGGCTGTATACCTCACTAGG
Baimangmai	TATACTCTACAGATAGAGGAAAACTGGAGGGAAGAGTCACAAATAACACGGCTGTATACCTCACTAGG

	10,000 · 10,010 · 10,020 · 10,030 · 10,040 · 10,050 · 10,060
Hulutou	GGCCGACGAGCTGCGGCAAGTAAAAATGTGCATTGATATTGGCCTAGACTGTGTCAAGTCAAACCCTG
Baihulu	GGCCGACGAGCTGCGGCAAGTAAAAATGTGCATTGATATTGGCCTAGACTGTGTCAAGTCAAACCCTG
Chiyacao	GGCCGACGAGCTGCGGCAAGTAAAAATGTGCATTGATATTGGCCTAGACTGTGTCAAGTCAAACCCTG
Hongmangmai	GGCCGACGAGCTGCGGCAAGTAAAAATGTGCATTGATATTGGCCTAGACTGTGTCAAGTCAAACCCTG
Hongmai	GGCCGACGAGCTGCGGCAAGTAAAAATGTGCATTGATATTGGCCTAGACTGTGTCAAGTCAAACCCTG
Wangshuibai	GGCCGACGAGCTGCGGCAAGTAAAAATGTGCATTGATATTGGCCTAGACTGTGTCAAGTCAAACCCTG
Shi 4185	GGCCGACGAGCTGCGGCAAGTAAAAATGTGCATTGATATTGGCCTAGACTGTGTCAAGTCAAACCCTG
Mazhamai	GGCCGACGAGCTGCGGCAAGTAAAAATGTGCATTGATATTGGCCTAGACTGTGTCAAGTCAAACCCTG
Xiaofoshou	GGCCGACGAGCTGCGGCAAGTAAAAATGTGCATTGATATTGGCCTAGACTGTGTCAAGTCAAACCCTG
Aikang 58	GGCCGACGAGCTGCGGCAAGTAAAAATGTGCATTGATATTGGCCTAGACTGTGTCAAGTCAAACCCTG
AL8/78	GGC**G**GACGAGCTGCGGCAAGTAAAAATGTGCATTGATATTGGCCTAGACTGTGTCAAGTCAAACCCTG
PI 431602	GGCCGACGAGCTGCGGCAAGTAAAAATGTGCATTGATATTGGCCTAGACTGTGTCAAGTCAAACCCTG
PI 486274	GGCCGACGAGCTGCGGCAAGTAAAAATGTGCATTGATATTGGCCTAGACTGTGTCAAGTCAAACCCTG
RM 000182	GGCCGACGAGCTGCGGCAAGTAAAAATGTGCATTGATATTGGCCTAGACTGTGTCAAGTCAAACCCTG
PI 511381	GGCCGACGAGCTGCGGCAAGTAAAAATGTGCATTGATATTGGCCTAGACTGTGTCAAGTCAAACCCTG
PI 486271	GGCCGACGAGCTGCGGCAAGTAAAAATGTGCATTGATATTGGCCTAGACTGTGTCAAGTCAAACCCTG
PI 511367	GGCCGACGAGCTGCGGCAAGTAAAAATGTGCATTGATATTGGCCTAGACTGTGTCAAGTCAAACCCTG
Chinese Spring	GGCCGACGAGCTGCGGCAAGTAAAAATGTGCATTGATATTGGCCTAGACTGTGTCAAGTCAAACCCTG
Hanzhongbai	GGCCGACGAGCTGCGGCAAGTAAAAATGTGCATTGATATTGGCCTAGACTGTGTCAAGTCAAACCCTG
Hongtoumai	GGCCGACGAGCTGCGGCAAGTAAAAATGTGCATTGATATTGGCCTAGACTGTGTCAAGTCAAACCCTG
Hongyouzi	GGCCGACGAGCTGCGGCAAGTAAAAATGTGCATTGATATTGGCCTAGACTGTGTCAAGTCAAACCCTG
Zijiehong	GGCCGACGAGCTGCGGCAAGTAAAAATGTGCATTGATATTGGCCTAGACTGTGTCAAGTCAAACCCTG
Baihuamai	GGCCGACGAGCTGCGGCAAGTAAAAATGTGCATTGATATTGGCCTAGACTGTGTCAAGTCAAACCCTG
Huixianhong	GGCCGACGAGCTGCGGCAAGTAAAAATGTGCATTGATATTGGCCTAGACTGTGTCAAGTCAAACCCTG
Baimangmai	GGCCGACGAGCTGCGGCAAGTAAAAATGTGCATTGATATTGGCCTAGACTGTGTCAAGTCAAACCCTG

```
                 10,070    10,080    10,090    10,100    10,110    10,120    10,130
Hulutou          AAAAGAGACCTACAGCTGGGGCCATCATGCTCTGGCTTGACAAAGAGAGCAAACCGGTCCCAGTTTCA
Baihulu          AAAAGAGACCTACAGCTGGGGCCATCATGCTCTGGCTTGACAAAGAGAGCAAACCGGTCCCAGTTTCA
Chiyacao         AAAAGAGACCTACAGCTGGGGCCATCATGCTCTGGCTTGACAAAGAGAGCAAACCGGTCCCAGTTTCA
Hongmangmai      AAAAGAGACCTACAGCTGGGGCCATCATGCTCTGGCTTGACAAAGAGAGCAAACCGGTCCCAGTTTCA
Hongmai          AAAAGAGACCTACAGCTGGGGCCATCATGCTCTGGCTTGACAAAGAGAGCAAACCGGTCCCAGTTTCA
Wangshuibai      AAAAGAGACCTACAGCTGGGGCCATCATGCTCTGGCTTGACAAAGAGAGCAAACCGGTCCCAGTTTCA
Shi 4185         AAAAGAGACCTACAGCTGGGGCCATCATGCTCTGGCTTGACAAAGAGAGCAAACCGGTCCCAGTTTCA
Mazhamai         AAAAGAGACCTACAGCTGGGGCCATCATGCTCTGGCTTGACAAAGAGAGCAAACCGGTCCCAGTTTCA
Xiaofoshou       AAAAGAGACCTACAGCTGGGGCCATCATGCTCTGGCTTGACAAAGAGAGCAAACCGGTCCCAGTTTCA
Aikang 58        AAAAGAGACCTACAGCTGGGGCCATCATGCTCTGGCTTGACAAAGAGAGCAAACCGGTCCCAGTTTCA
AL8/78           AAAAGAGACCTACAGCTGGGGCCATCATGGACTGGCTTAACAAAGAGAGCAAACCGGTCCCAGTTTCA
PI 431602        AAAAGAGACCTACAGCTGGGGCCATCATGGACTGGCTTAACAAAGAGAGCAAACCGGTCCCAGTTTCA
PI 486274        AAAAGAGACCTACAGCTGGGGCCATCATGGACTGGCTTAACAAAGAGAGCAAACCGGTCCCAGTTTCA
RM 000182        AAAAGAGACCTACAGCTGGGGCCATCATGCTCTGGCTTGACAAAGAGAGCAAACCGGTCCCAGTTTCA
PI 511381        AAAAGAGACCTACAGCTGGGGCCATCATGCTCTGGCTTGACAAAGAGAGCAAACCGGTCCCAGTTTCA
PI 486271        AAAAGAGACCTACAGCTGGGGCCATCATGCTCTGGCTTGACAAAGAGAGCAAACCGGTCCCAGTTTCA
PI 511367        AAAAGAGACCTACAGCTGGGGCCATCATGCTCTGGCTTGACAAAGAGAGCAAACCGGTCCCAGTTTCA
Chinese Spring   AAAAGAGACCTACAGCTGGGGCCATCATGCTCTGGCTTGACAAAGAGAGCAAACCGGTCCCAGTTTCA
Hanzhongbai      AAAAGAGACCTACAGCTGGGGCCATCATGCTCTGGCTTGACAAAGAGAGCAAACCGGTCCCAGTTTCA
Hongtoumai       AAAAGAGACCTACAGCTGGGGCCATCATGCTCTGGCTTGACAAAGAGAGCAAACCGGTCCCAGTTTCA
Hongyouzi        AAAAGAGACCTACAGCTGGGGCCATCATGCTCTGGCTTGACAAAGAGAGCAAACCGGTCCCAGTTTCA
Zijiehong        AAAAGAGACCTACAGCTGGGGCCATCATGCTCTGGCTTGACAAAGAGAGCAAACCGGTCCCAGTTTCA
Baihuamai        AAAAGAGACCTACAGCTGGGGCCATCATGCTCTGGCTTGACAAAGAGAGCAAACCGGTCCCAGTTTCA
Huixianhong      AAAAGAGACCTACAGCTGGGGCCATCATGCTCTGGCTTGACAAAGAGAGCAAACCGGTCCCAGTTTCA
Baimangmai       AAAAGAGACCTACAGCTGGGGCCATCATGCTCTGGCTTGACAAAGAGAGCAAACCGGTCCCAGTTTCA

                 10,140    10,150    10,160    10,170    10,180    10,190    10,200
Hulutou          AGGGCA-GGTGCAGGAGTGCTGCCAAGACCTCCGGTCCCTACTAATATCAACCATGCAGGTCGCATCC
Baihulu          AGGGCA-GGTGCAGGAGTGCTGCCAAGACCTCCGGTCCCTACTAATATCAACCATGCAGGTCGCATCC
Chiyacao         AGGGCA-GGTGCAGGAGTGCTGCCAAGACCTCCGGTCCCTACTAATATCAACCATGCAGGTCGCATCC
Hongmangmai      AGGGCA-GGTGCAGGAGTGCTGCCAAGACCTCCGGTCCCTACTAATATCAACCATGCAGGTCGCATCC
Hongmai          AGGGCA-GGTGCAGGAGTGCTGCCAAGACCTCCGGTCCCTACTAATATCAACCATGCAGGTCGCATCC
Wangshuibai      AGGGCA-GGTGCAGGAGTGCTGCCAAGACCTCCGGTCCCTACTAATATCAACCATGCAGGTCGCATCC
Shi 4185         AGGGCA-GGTGCAGGAGTGCTGCCAAGACCTCCGGTCCCTACTAATATCAACCATGCAGGTCGCATCC
Mazhamai         AGGGCA-GGTGCAGGAGTGCTGCCAAGACCTCCGGTCCCTACTAATATCAACCATGCAGGTCGCATCC
Xiaofoshou       AGGGCA-GGTGCAGGAGTGCTGCCAAGACCTCCGGTCCCTACTAATATCAACCATGCAGGTCGCATCC
Aikang 58        AGGGCA-GGTGCAGGAGTGCTGCCAAGACCTCCGGTCCCTACTAATATCAACCATGCAGGTCGCATCC
AL8/78           AGGGCAGGGTGCAGGAGTGCTGCCAAGACCTCCGGTCCCTACTAATATCAACCATGCAGGTCGCATCC
PI 431602        AGGGCA-GGTGCAGGAGTGCTGCCAAGACCTCCGGTCCCTACTAATATCAACCATGCAGGTCGCATCC
PI 486274        AGGGCA-GGTGCAGGAGTGCTGCCAAGACCTCCGGTCCCTACTAATATCAACCATGCAGGTCGCATCC
RM 000182        AGGGCA-GGTGCAGGAGTGCTGCCAAGACCTCCGGTCCCTACTAATATCAACCATGCAGGTCGCATCC
PI 511381        AGGGCA-GGTGCAGGAGTGCTGCCAAGACCTCCGGTCCCTACTAATATCAACCATGCAGGTCGCATCC
PI 486271        AGGGCA-GGTGCAGGAGTGCTGCCAAGACCTCCGGTCCCTACTAATATCAACCATGCAGGTCGCATCC
PI 511367        AGGGCA-GGTGCAGGAGTGCTGCCAAGACCTCCGGTCCCTACTAATATCAACCATGCAGGTCGCATCC
Chinese Spring   AGGGCA-GGTGCAGGAGTGCTGCCAAGACCTCCGGTCCCTACTAATATCAACCATGCAGGTCGCATCC
Hanzhongbai      AGGGCA-GGTGCAGGAGTGCTGCCAAGACCTCCGGTCCCTACTAATATCAACCATGCAGGTCGCATCC
Hongtoumai       AGGGCA-GGTGCAGGAGTGCTGCCAAGACCTCCGGTCCCTACTAATATCAACCATGCAGGTCGCATCC
Hongyouzi        AGGGCA-GGTGCAGGAGTGCTGCCAAGACCTCCGGTCCCTACTAATATCAACCATGCAGGTCGCATCC
Zijiehong        AGGGCA-GGTGCAGGAGTGCTGCCAAGACCTCCGGTCCCTACTAATATCAACCATGCAGGTCGCATCC
Baihuamai        AGGGCA-GGTGCAGGAGTGCTGCCAAGACCTCCGGTCCCTACTAATATCAACCATGCAGGTCGCATCC
Huixianhong      AGGGCA-GGTGCAGGAGTGCTGCCAAGACCTCCGGTCCCTACTAATATCAACCATGCAGGTCGCATCC
Baimangmai       AGGGCA-GGTGCAGGAGTGCTGCCAAGACCTCCGGTCCCTACTAATATCAACCATGCAGGTCGCATCC
```

```
                        10,210       10,220       10,230       10,240       10,250       10,260
Hulutou          AAGGTATTCCGATTGAGAAGTCTCAAGTGTTAATTCCTTCGTTTGCATGCAATGAATATTCTTCGGTG
Baihulu          AAGGTATTCCGATTGAGAAGTCTCAAGTGTTAATTCCTTCGTTTGCATGCAATGAATATTCTTCGGTG
Chiyacao         AAGGTATTCCGATTGAGAAGTCTCAAGTGTTAATTCCTTCGTTTGCATGCAATGAATATTCTTCGGTG
Hongmangmai      AAGGTATTCCGATTGAGAAGTCTCAAGTGTTAATTCCTTCGTTTGCATGCAATGAATATTCTTCGGTG
Hongmai          AAGGTATTCCGATTGAGAAGTCTCAAGTGTTAATTCCTTCGTTTGCATGCAATGAATATTCTTCGGTG
Wangshuibai      AAGGTATTCCGATTGAGAAGTCTCAAGTGTTAATTCCTTCGTTTGCATGCAATGAATATTCTTCGGTG
Shi 4185         AAGGTATTCCGATTGAGAAGTCTCAAGTGTTAATTCCTTCGTTTGCATGCAATGAATATTCTTCGGTG
Mazhamai         AAGGTATTCCGATTGAGAAGTCTCAAGTGTTAATTCCTTCGTTTGCATGCAATGAATATTCTTCGGTG
Xiaofoshou       AAGGTATTCCGATTGAGAAGTCTCAAGTGTTAATTCCTTCGTTTGCATGCAATGAATATTCTTCGGTG
Aikang 58        AAGGTATTCCGATTGAGAAGTCTCAAGTGTTAATTCCTTCGTTTGCATGCAATGAATATTCTTCGGTG
AL8/78           AAGGTATTCCGATTGAGAAGTCTCAAGTGTTAATTCCTTCGTTTGCATGCAATGAATATTCTTCGGTG
PI 431602        AAGGTATTCCGATTGAGAAGTCTCAAGTGTTAATTCCTTCGTTTGCATGCAATGAATATTCTTCGGTG
PI 486274        AAGGTATTCCGATTGAGAAGTCTCAAGTGTTAATTCCTTCGTTTGCATGCAATGAATATTCTTCGGTG
RM 000182        AAGGTATTCCGATTGAGAAGTCTCAAGTGTTAATTCCTTCGTTTGCATGCAATGAATATTCTTCGGTG
PI 511381        AAGGTATTCCGATTGAGAAGTCTCAAGTGTTAATTCCTTCGTTTGCATGCAATGAATATTCTTCGGTG
PI 486271        AAGGTATTCCGATTGAGAAGTCTCAAGTGTTAATTCCTTCGTTTGCATGCAATGAATATTCTTCGGTG
PI 511367        AAGGTATTCCGATTGAGAAGTCTCAAGTGTTAATTCCTTCGTTTGCATGCAATGAATATTCTTCGGTG
Chinese Spring   AAGGTATTCCGATTGAGAAGTCTCAAGTGTTAATTCCTTCGTTTGCATGCAATGAATATTCTTCGGTG
Hanzhongbai      AAGGTATTCCGATTGAGAAGTCTCAAGTGTTAATTCCTTCGTTTGCATGCAATGAATATTCTTCGGTG
Hongtoumai       AAGGTATTCCGATTGAGAAGTCTCAAGTGTTAATTCCTTCGTTTGCATGCAATGAATATTCTTCGGTG
Hongyouzi        AAGGTATTCCGATTGAGAAGTCTCAAGTGTTAATTCCTTCGTTTGCATGCAATGAATATTCTTCGGTG
Zijiehong        AAGGTATTCCGATTGAGAAGTCTCAAGTGTTAATTCCTTCGTTTGCATGCAATGAATATTCTTCGGTG
Baihuamai        AAGGTATTCCGATTGAGAAGTCTCAAGTGTTAATTCCTTCGTTTGCATGCAATGAATATTCTTCGGTG
Huixianhong      AAGGTATTCCGATTGAGAAGTCTCAAGTGTTAATTCCTTCGTTTGCATGCAATGAATATTCTTCGGTG
Baimangmai       AAGGTATTCCGATTGAGAAGTCTCAAGTGTTAATTCCTTCGTTTGCATGCAATGAATATTCTTCGGTG

                 10,270     10,280     10,290     10,300     10,310     10,320     10,330
Hulutou          AAAATACAAAATCTTCATCTACTTCTGTTTATGTATTACT--------------AAGCAGCCGCAAGC
Baihulu          AAAATACAAAATCTTCATCTACTTCTGTTTATGTATTACT--------------AAGCAGCCGCAAGC
Chiyacao         AAAATACAAAATCTTCATCTACTTCTGTTTATGTATTACT--------------AAGCAGCCGCAAGC
Hongmangmai      AAAATACAAAATCTTCATCTACTTCTGTTTATGTATTACT--------------AAGCAGCCGCAAGC
Hongmai          AAAATACAAAATCTTCATCTACTTCTGTTTATGTATTACT--------------AAGCAGCCGCAAGC
Wangshuibai      AAAATACAAAATCTTCATCTACTTCTGTTTATGTATTACT--------------AAGCAGCCGCAAGC
Shi 4185         AAAATACAAAATCTTCATCTACTTCTGTTTATGTATTACT--------------AAGCAGCCGCAAGC
Mazhamai         AAAATACAAAATCTTCATCTACTTCTGTTTATGTATTACT--------------AAGCAGCCGCAAGC
Xiaofoshou       AAAATACAAAATCTTCATCTACTTCTGTTTATGTATTACT--------------AAGCAGCCGCAAGC
Aikang 58        AAAATACAAAATCTTCATCTACTTCTGTTTATGTATTACT--------------AAGCAGCCGCAAGC
AL8/78           AAAATACAAAATCTTCATCTACTTCTGTTTATGTTCATCCAAGTTGATCAGTAAAAGCAGCCGCAAGC
PI 431602        AAAATACAAAATCTTCATCTACTTCTGTTTATGTTCATCCAAGTTGATCAGTAAAAGCAGCCGCAAGC
PI 486274        AAAATACAAAATCTTCATCTACTTCTGTTTATGTTCATCCAAGTTGATCAGTAAAAGCAGCCGCAAGC
RM 000182        AAAATACAAAATCTTCATCTACTTCTGTTTATGTTCATCCAAGTTGATCAGTAAAAGCAGCCGCAAGC
PI 511381        AAAATACAAAATCTTCATCTACTTCTGTTTATGTTCATCCAAGTTGATCAGTAAAAGCAGCCGCAAGC
PI 486271        AAAATACAAAATCTTCATCTACTTCTGTTTATGTTCATCCAAGTTGATCAGTAAAAGCAGCCGCAAGC
PI 511367        AAAATACAAAATCTTCATCTACTTCTGTTTATGTTCATCCAAGTTGATCAGTAAAAGCAGCCGCAAGC
Chinese Spring   AAAATACAAAATCTTCATCTACTTCTGTTTATGTTCATCCAAGTTGATCAGTAAAAGCAGCCGCAAGC
Hanzhongbai      AAAATACAAAATCTTCATCTACTTCTGTTTATGTTCATCCAAGTTGATCAGTAAAAGCAGCCGCAAGC
Hongtoumai       AAAATACAAAATCTTCATCTACTTCTGTTTATGTTCATCCAAGTTGATCAGTAAAAGCAGCCGCAAGC
Hongyouzi        AAAATACAAAATCTTCATCTACTTCTGTTTATGTTCATCCAAGTTGATCAGTAAAAGCAGCCGCAAGC
Zijiehong        AAAATACAAAATCTTCATCTACTTCTGTTTATGTTCATCCAAGTTGATCAGTAAAAGCAGCCGCAAGC
Baihuamai        AAAATACAAAATCTTCATCTACTTCTGTTTATGTTCATCCAAGTTGATCAGTAAAAGCAGCCGCAAGC
Huixianhong      AAAATACAAAATCTTCATCTACTTCTGTTTATGTTCATCCAAGTTGATCAGTAAAAGCAGCCGCAAGC
Baimangmai       AAAATACAAAATCTTCATCTACTTCTGTTTATGTTCATCCAAGTTGATCAGTAAAAGCAGCCGCAAGC
```

	10,340 · 10,350 · 10,360 · 10,370 · 10,380 · 10,390 · 10,400
Hulutou	TATTCCCTACTTCTCAATTGTTAATTCCTTCATTAGCATGCAGTGAATATTCTTTGGTGAAAATTGCA
Baihulu	TATTCCCTACTTCTCAATTGTTAATTCCTTCATTAGCATGCAGTGAATATTCTTTGGTGAAAATTGCA
Chiyacao	TATTCCCTACTTCTCAATTGTTAATTCCTTCATTAGCATGCAGTGAATATTCTTTGGTGAAAATTGCA
Hongmangmai	TATTCCCTACTTCTCAATTGTTAATTCCTTCATTAGCATGCAGTGAATATTCTTTGGTGAAAATTGCA
Hongmai	TATTCCCTACTTCTCAATTGTTAATTCCTTCATTAGCATGCAGTGAATATTCTTTGGTGAAAATTGCA
Wangshuibai	TATTCCCTACTTCTCAATTGTTAATTCCTTCATTAGCATGCAGTGAATATTCTTTGGTGAAAATTGCA
Shi 4185	TATTCCCTACTTCTCAATTGTTAATTCCTTCATTAGCATGCAGTGAATATTCTTTGGTGAAAATTGCA
Mazhamai	TATTCCCTACTTCTCAATTGTTAATTCCTTCATTAGCATGCAGTGAATATTCTTTGGTGAAAATTGCA
Xiaofoshou	TATTCCCTACTTCTCAATTGTTAATTCCTTCATTAGCATGCAGTGAATATTCTTTGGTGAAAATTGCA
Aikang 58	TATTCCCTACTTCTCAATTGTTAATTCCTTCATTAGCATGCAGTGAATATTCTTTGGTGAAAATTGCA
AL8/78	TATTCCCTACTTCTCAATTGTTAATTCCTTCATTAGCATGCAGTGAATATTCTTTGGTGAAAATTGCA
PI 431602	TATTCCCTACTTCTCAATTGTTAATTCCTTCATTAGCATGCAGTGAATATTCTTTGGTGAAAATTGCA
PI 486274	TATTCCCTACTTCTCAATTGTTAATTCCTTCATTAGCATGCAGTGAATATTCTTTGGTGAAAATTGCA
RM 000182	TATTCCCTACTTCTCAATTGTTAATTCCTTCATTAGCATGCAGTGAATATTCTTTGGTGAAAATTGCA
PI 511381	TATTCCCTACTTCTCAATTGTTAATTCCTTCATTAGCATGCAGTGAATATTCTTTGGTGAAAATTGCA
PI 486271	TATTCCCTACTTCTCAATTGTTAATTCCTTCATTAGCATGCAGTGAATATTCTTTGGTGAAAATTGCA
PI 511367	TATTCCCTACTTCTCAATTGTTAATTCCTTCATTAGCATGCAGTGAATATTCTTTGGTGAAAATTGCA
Chinese Spring	TATTCCCTACTTCTCAATTGTTAATTCCTTCATTAGCATGCAGTGAATATTCTTTGGTGAAAATTGCA
Hanzhongbai	TATTCCCTACTTCTCAATTGTTAATTCCTTCATTAGCATGCAGTGAATATTCTTTGGTGAAAATTGCA
Hongtoumai	TATTCCCTACTTCTCAATTGTTAATTCCTTCATTAGCATGCAGTGAATATTCTTTGGTGAAAATTGCA
Hongyouzi	TATTCCCTACTTCTCAATTGTTAATTCCTTCATTAGCATGCAGTGAATATTCTTTGGTGAAAATTGCA
Zijiehong	TATTCCCTACTTCTCAATTGTTAATTCCTTCATTAGCATGCAGTGAATATTCTTTGGTGAAAATTGCA
Baihuamai	TATTCCCTACTTCTCAATTGTTAATTCCTTCATTAGCATGCAGTGAATATTCTTTGGTGAAAATTGCA
Huixianhong	TATTCCCTACTTCTCAATTGTTAATTCCTTCATTAGCATGCAGTGAATATTCTTTGGTGAAAATTGCA
Baimangmai	TATTCCCTACTTCTCAATTGTTAATTCCTTCATTAGCATGCAGTGAATATTCTTTGGTGAAAATTGCA

	10,410 · 10,420 · 10,430 · 10,440 · 10,450 · 10,460 · 10,470
Hulutou	TCTATGAAAGGAAATTAAAATACAGTGTCTTCATCTATTACTATTTCTATTCATCCAAGCTGATCAGT
Baihulu	TCTATGAAAGGAAATTAAAATACAGTGTCTTCATCTATTACTATTTCTATTCATCCAAGCTGATCAGT
Chiyacao	TCTATGAAAGGAAATTAAAATACAGTGTCTTCATCTATTACTATTTCTATTCATCCAAGCTGATCAGT
Hongmangmai	TCTATGAAAGGAAATTAAAATACAGTGTCTTCATCTATTACTATTTCTATTCATCCAAGCTGATCAGT
Hongmai	TCTATGAAAGGAAATTAAAATACAGTGTCTTCATCTATTACTATTTCTATTCATCCAAGCTGATCAGT
Wangshuibai	TCTATGAAAGGAAATTAAAATACAGTGTCTTCATCTATTACTATTTCTATTCATCCAAGCTGATCAGT
Shi 4185	TCTATGAAAGGAAATTAAAATACAGTGTCTTCATCTATTACTATTTCTATTCATCCAAGCTGATCAGT
Mazhamai	TCTATGAAAGGAAATTAAAATACAGTGTCTTCATCTATTACTATTTCTATTCATCCAAGCTGATCAGT
Xiaofoshou	TCTATGAAAGGAAATTAAAATACAGTGTCTTCATCTATTACTATTTCTATTCATCCAAGCTGATCAGT
Aikang 58	TCTATGAAAGGAAATTAAAATACAGTGTCTTCATCTATTACTATTTCTATTCATCCAAGCTGATCAGT
AL8/78	TCTATGAAAGGAAATTAAAATACAGTGTCTTCATCTATTACTATTTCTATTCATCCAAGCTGATCAGT
PI 431602	TCTATGAAAGGAAATTAAAATACAGTGTCTTCATCTATTACTATTTCTATTCATCCAAGCTGATCAGT
PI 486274	TCTATGAAAGGAAATTAAAATACAGTGTCTTCATCTATTACTATTTCTATTCATCCAAGCTGATCAGT
RM 000182	TCTATGAAAGGAAATTAAAATACAGTGTCTTCATCTATTACTATTTCTATTCATCCAAGCTGATCAGT
PI 511381	TCTATGAAAGGAAATTAAAATACAGTGTCTTCATCTATTACTATTTCTATTCATCCAAGCTGATCAGT
PI 486271	TCTATGAAAGGAAATTAAAATACAGTGTCTTCATCTATTACTATTTCTATTCATCCAAGCTGATCAGT
PI 511367	TCTATGAAAGGAAATTAAAATACAGTGTCTTCATCTATTACTATTTCTATTCATCCAAGCTGATCAGT
Chinese Spring	TCTATGAAAGGAAATTAAAATACAGTGTCTTCATCTATTACTATTTCTATTCATCCAAGCTGATCAGT
Hanzhongbai	TCTATGAAAGGAAATTAAAATACAGTGTCTTCATCTATTACTATTTCTATTCATCCAAGCTGATCAGT
Hongtoumai	TCTATGAAAGGAAATTAAAATACAGTGTCTTCATCTATTACTATTTCTATTCATCCAAGCTGATCAGT
Hongyouzi	TCTATGAAAGGAAATTAAAATACAGTGTCTTCATCTATTACTATTTCTATTCATCCAAGCTGATCAGT
Zijiehong	TCTATGAAAGGAAATTAAAATACAGTGTCTTCATCTATTACTATTTCTATTCATCCAAGCTGATCAGT
Baihuamai	TCTATGAAAGGAAATTAAAATACAGTGTCTTCATCTATTACTATTTCTATTCATCCAAGCTGATCAGT
Huixianhong	TCTATGAAAGGAAATTAAAATACAGTGTCTTCATCTATTACTATTTCTATTCATCCAAGCTGATCAGT
Baimangmai	TCTATGAAAGGAAATTAAAATACAGTGTCTTCATCTATTACTATTTCTATTCATCCAAGCTGATCAGT

	10,480 – 10,540
Hulutou	AAAAGCATCCACATGTTATAAGGGCAATCTATATGTGGTATTTAAAATGTTATTTCTGTCGCTTTTCA
Baihulu	AAAAGCATCCACATGTTATAAGGGCAATCTATATGTGGTATTTAAAATGTTATTTCTGTCGCTTTTCA
Chiyacao	AAAAGCATCCACATGTTATAAGGGCAATCTATATGTGGTATTTAAAATGTTATTTCTGTCGCTTTTCA
Hongmangmai	AAAAGCATCCACATGTTATAAGGGCAATCTATATGTGGTATTTAAAATGTTATTTCTGTCGCTTTTCA
Hongmai	AAAAGCATCCACATGTTATAAGGGCAATCTATATGTGGTATTTAAAATGTTATTTCTGTCGCTTTTCA
Wangshuibai	AAAAGCATCCACATGTTATAAGGGCAATCTATATGTGGTATTTAAAATGTTATTTCTGTCGCTTTTCA
Shi 4185	AAAAGCATCCACATGTTATAAGGGCAATCTATATGTGGTATTTAAAATGTTATTTCTGTCGCTTTTCA
Mazhamai	AAAAGCATCCACATGTTATAAGGGCAATCTATATGTGGTATTTAAAATGTTATTTCTGTCGCTTTTCA
Xiaofoshou	AAAAGCATCCACATGTTATAAGGGCAATCTATATGTGGTATTTAAAATGTTATTTCTGTCGCTTTTCA
Aikang 58	AAAAGCATCCACATGTTATAAGGGCAATCTATATGTGGTATTTAAAATGTTATTTCTGTCGCTTTTCA
AL8/78	AAAAGCATCCACATGTTATAAGGGCAATCTATATGTGGTATTTAAAATGTTATTTCTGTCGCTTTTCA
PI 431602	AAAAGCATCCACATGTTATAAGGGCAATCTATATGTGGTATTTAAAATGTTATTTCTGTCGCTTTTCA
PI 486274	AAAAGCATCCACATGTTATAAGGGCAATCTATATGTGGTATTTAAAATGTTATTTCTGTCGCTTTTCA
RM 000182	AAAAGCATCCACATGTTATAAGGGCAATCTATATGTGGTATTTAAAATGTTATTTCTGTCGCTTTTCA
PI 511381	AAAAGCATCCACATGTTATAAGGGCAATCTATATGTGGTATTTAAAATGTTATTTCTGTCGCTTTTCA
PI 486271	AAAAGCATCCACATGTTATAAGGGCAATCTATATGTGGTATTTAAAATGTTATTTCTGTCGCTTTTCA
PI 511367	AAAAGCATCCACATGTTATAAGGGCAATCTATATGTGGTATTTAAAATGTTATTTCTGTCGCTTTTCA
Chinese Spring	AAAAGCATCCACATGTTATAAGGGCAATCTATATGTGGTATTTAAAATGTTATTTCTGTCGCTTTTCA
Hanzhongbai	AAAAGCATCCACATGTTATAAGGGCAATCTATATGTGGTATTTAAAATGTTATTTCTGTCGCTTTTCA
Hongtoumai	AAAAGCATCCACATGTTATAAGGGCAATCTATATGTGGTATTTAAAATGTTATTTCTGTCGCTTTTCA
Hongyouzi	AAAAGCATCCACATGTTATAAGGGCAATCTATATGTGGTATTTAAAATGTTATTTCTGTCGCTTTTCA
Zijiehong	AAAAGCATCCACATGTTATAAGGGCAATCTATATGTGGTATTTAAAATGTTATTTCTGTCGCTTTTCA
Baihuamai	AAAAGCATCCACATGTTATAAGGGCAATCTATATGTGGTATTTAAAATGTTATTTCTGTCGCTTTTCA
Huixianhong	AAAAGCATCCACATGTTATAAGGGCAATCTATATGTGGTATTTAAAATGTTATTTCTGTCGCTTTTCA
Baimangmai	AAAAGCATCCACATGTTATAAGGGCAATCTATATGTGGTATTTAAAATGTTATTTCTGTCGCTTTTCA

	10,550 – 10,600
Hulutou	CATCGTATATGCAATCTAAGATAATGCTCTCCCTCTCACTGTAAATAAATATGTTACCAGAAAAGGAG
Baihulu	CATCGTATATGCAATCTAAGATAATGCTCTCCCTCTCACTGTAAATAAATATGTTACCAGAAAAGGAG
Chiyacao	CATCGTATATGCAATCTAAGATAATGCTCTCCCTCTCACTGTAAATAAATATGTTACCAGAAAAGGAG
Hongmangmai	CATCGTATATGCAATCTAAGATAATGCTCTCCCTCTCACTGTAAATAAATATGTTACCAGAAAAGGAG
Hongmai	CATCGTATATGCAATCTAAGATAATGCTCTCCCTCTCACTGTAAATAAATATGTTACCAGAAAAGGAG
Wangshuibai	CATCGTATATGCAATCTAAGATAATGCTCTCCCTCTCACTGTAAATAAATATGTTACCAGAAAAGGAG
Shi 4185	CATCGTATATGCAATCTAAGATAATGCTCTCCCTCTCACTGTAAATAAATATGTTACCAGAAAAGGAG
Mazhamai	CATCGTATATGCAATCTAAGATAATGCTCTCCCTCTCACTGTAAATAAATATGTTACCAGAAAAGGAG
Xiaofoshou	CATCGTATATGCAATCTAAGATAATGCTCTCCCTCTCACTGTAAATAAATATGTTACCAGAAAAGGAG
Aikang 58	CATCGTATATGCAATCTAAGATAATGCTCTCCCTCTCACTGTAAATAAATATGTTACCAGAAAAGGAG
AL8/78	CATCGTATATGCAATCTAAGATAATGCTCTCCCTCTCACTGTAAATAAATATGTTACCAGAAAAGGAG
PI 431602	CATCGTATATGCAATCTAAGATAATGCTCTCCCTCTCACTGTAAATAAATATGTTACCAGAAAAGGAG
PI 486274	CATCGTATATGCAATCTAAGATAATGCTCTCCCTCTCACTGTAAATAAATATGTTACCAGAAAAGGAG
RM 000182	CAT**T**GTATATGCAATCTAAGATAATGCTCTCCCTCTCACTGTAAATAAATATGTTACCAGAAAAGGAG
PI 511381	CAT**T**GTATATGCAATCTAAGATAATGCTCTCCCTCTCACTGTAAATAAATATGTTACCAGAAAAGGAG
PI 486271	CAT**T**GTATATGCAATCTAAGATAATGCTCTCCCTCTCACTGTAAATAAATATGTTACCAGAAAAGGAG
PI 511367	CAT**T**GTATATGCAATCTAAGATAATGCTCTCCCTCTCACTGTAAATAAATATGTTACCAGAAAAGGAG
Chinese Spring	CATCGTATATGCAATCTAAGATAATGCTCTCCCTCTCACTGTAAATAAATATGTTACCAGAAAAGGAG
Hanzhongbai	CATCGTATATGCAATCTAAGATAATGCTCTCCCTCTCACTGTAAATAAATATGTTACCAGAAAAGGAG
Hongtoumai	CATCGTATATGCAATCTAAGATAATGCTCTCCCTCTCACTGTAAATAAATATGTTACCAGAAAAGGAG
Hongyouzi	CATCGTATATGCAATCTAAGATAATGCTCTCCCTCTCACTGTAAATAAATATGTTACCAGAAAAGGAG
Zijiehong	CATCGTATATGCAATCTAAGATAATGCTCTCCCTCTCACTGTAAATAAATATGTTACCAGAAAAGGAG
Baihuamai	CATCGTATATGCAATCTAAGATAATGCTCTCCCTCTCACTGTAAATAAATATGTTACCAGAAAAGGAG
Huixianhong	CATCGTATATGCAATCTAAGATAATGCTCTCCCTCTCACTGTAAATAAATATGTTACCAGAAAAGGAG
Baimangmai	CATCGTATATGCAATCTAAGATAATGCTCTCCCTCTCACTGTAAATAAATATGTTACCAGAAAAGGAG

10,610 10,620 10,630 10,640 10,650

Hulutou	AAGGCGGGATTCCTGAAACGACACTTCGGATGGAAGAAGTAA
Baihulu	AAGGCGGGATTCCTGAAACGACACTTCGGATGGAAGAAGTAA
Chiyacao	AAGGCGGGATTCCTGAAACGACACTTCGGATGGAAGAAGTAA
Hongmangmai	AAGGCGGGATTCCTGAAACGACACTTCGGATGGAAGAAGTAA
Hongmai	AAGGCGGGATTCCTGAAACGACACTTCGGATGGAAGAAGTAA
Wangshuibai	AAGGCGGGATTCCTGAAACGACACTTCGGATGGAAGAAGTAA
Shi 4185	AAGGCGGGATTCCTGAAACGACACTTCGGATGGAAGAAGTAA
Mazhamai	AAGGCGGGATTCCTGAAACGACACTTCGGATGGAAGAAGTAA
Xiaofoshou	AAGGCGGGATTCCTGAAACGACACTTCGGATGGAAGAAGTAA
Aikang 58	AAGGCGGGATTCCTGAAACGACACTTCGGATGGAAGAAGTAA
AL8/78	AAGGCGGGATTCCTGAAACGACACTTCGGATGGAAGAAGTAA
PI 431602	AAGGCGGGATTCCTGAAACGACACTTCGGATGGAAGAAGTAA
PI 486274	AAGGCGGGATTCCTGAAACGACACTTCGGATGGAAGAAGTAA
RM 000182	AAGGCGGGATTCCTGAAACGACACTTCGGATGGAAGAAGTAA
PI 511381	AAGGCGGGATTCCTGAAACGACACTTCGGATGGAAGAAGTAA
PI 486271	AAGGCGGGATTCCTGAAACGACACTTCGGATGGAAGAAGTAA
PI 511367	AAGGCGGGATTCCTGAAACGACACTTCGGATGGAAGAAGTAA
Chinese Spring	AAGGCGGGATTCCTGAAACGACACTTCGGATGGAAGAAGTAA
Hanzhongbai	AAGGCGGGATTCCTGAAACGACACTTCGGATGGAAGAAGTAA
Hongtoumai	AAGGCGGGATTCCTGAAACGACACTTCGGATGGAAGAAGTAA
Hongyouzi	AAGGCGGGATTCCTGAAACGACACTTCGGATGGAAGAAGTAA
Zijiehong	AAGGCGGGATTCCTGAAACGACACTTCGGATGGAAGAAGTAA
Baihuamai	AAGGCGGGATTCCTGAAACGACACTTCGGATGGAAGAAGTAA
Huixianhong	AAGGCGGGATTCCTGAAACGACACTTCGGATGGAAGAAGTAA
Baimangmai	AAGGCGGGATTCCTGAAACGACACTTCGGATGGAAGAAGTAA

Supplementary Data 3 Genomic DNA alignments of *WTK3* gene in 25 common wheat and *Ae. tauschii* accessions.
The SNPs and InDels were shown in different colors. Red triangle indicates the 6-bp deletion.
