# Supplementary material for: A rare gain of function mutation in a wheat tandem kinase confers resistance to powdery mildew
Source: Nat Commun. 2020 Feb 3;11:680. doi: 10.1038/s41467-020-14294-0 (PMC6997164; doi:10.1038/s41467-020-14294-0)
Supplement: Supplementary file 9 — Source Data [file 41467_2020_14294_MOESM9_ESM.pdf]

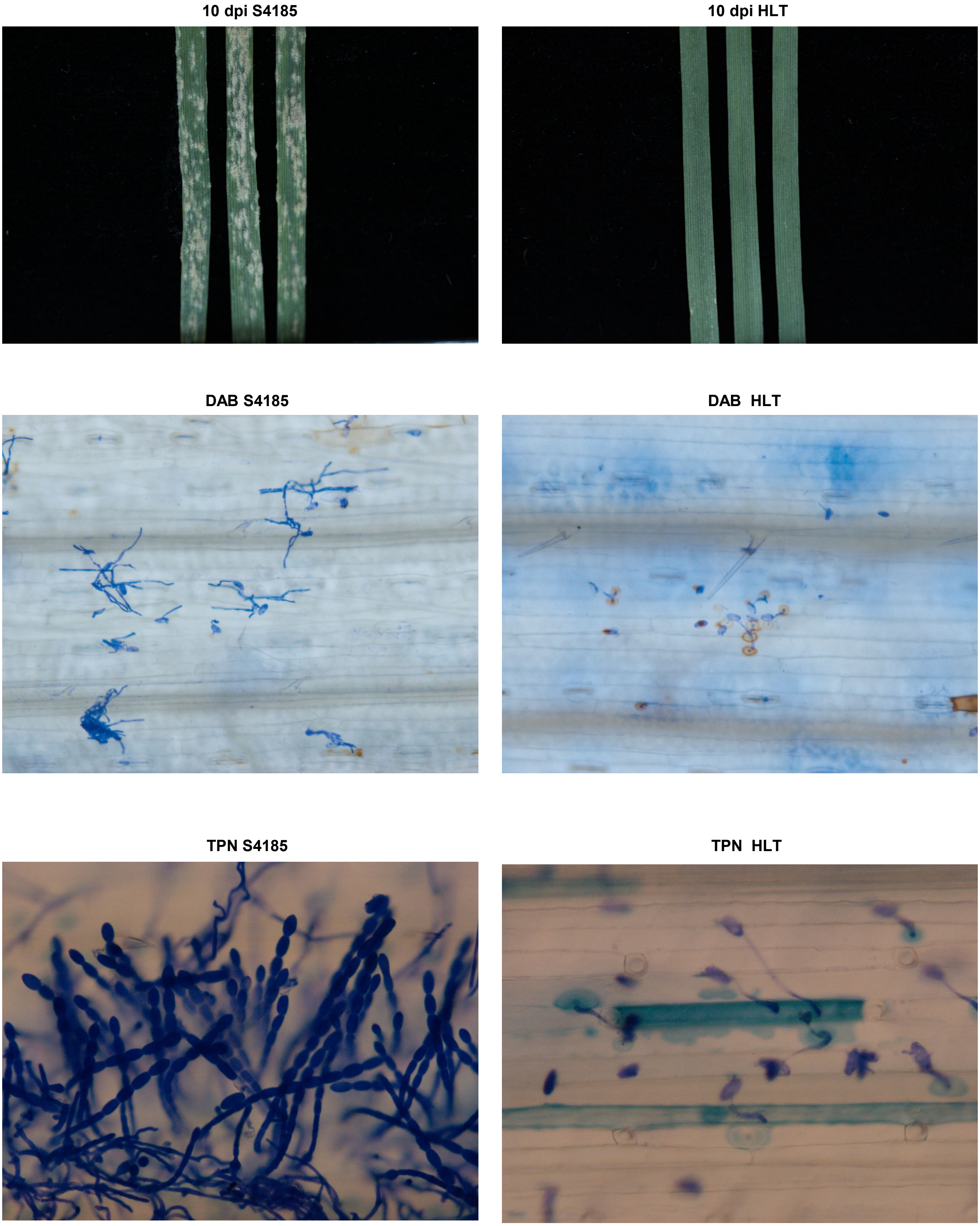

Fig. 1a Chinese wheat landrace HLT is highly resistant to powdery mildew. Two-week-old S4185 and HLT plants were inoculated with *Bgt* isolate E09. Representative leaves were photographed at 10 d post inoculation (dpi). Bar, 5 mm. DAB staining of leaves infected with *Bgt* isolate E09 at 2 dpi. Brown staining shows the accumulation of H_2O_2. Bar, 100 μm. Trypan blue staining of the leaves infected with *Bgt* isolate E09 at 1 dpi to visualize fungal structures and plant cell death. Bar, 100 μm.

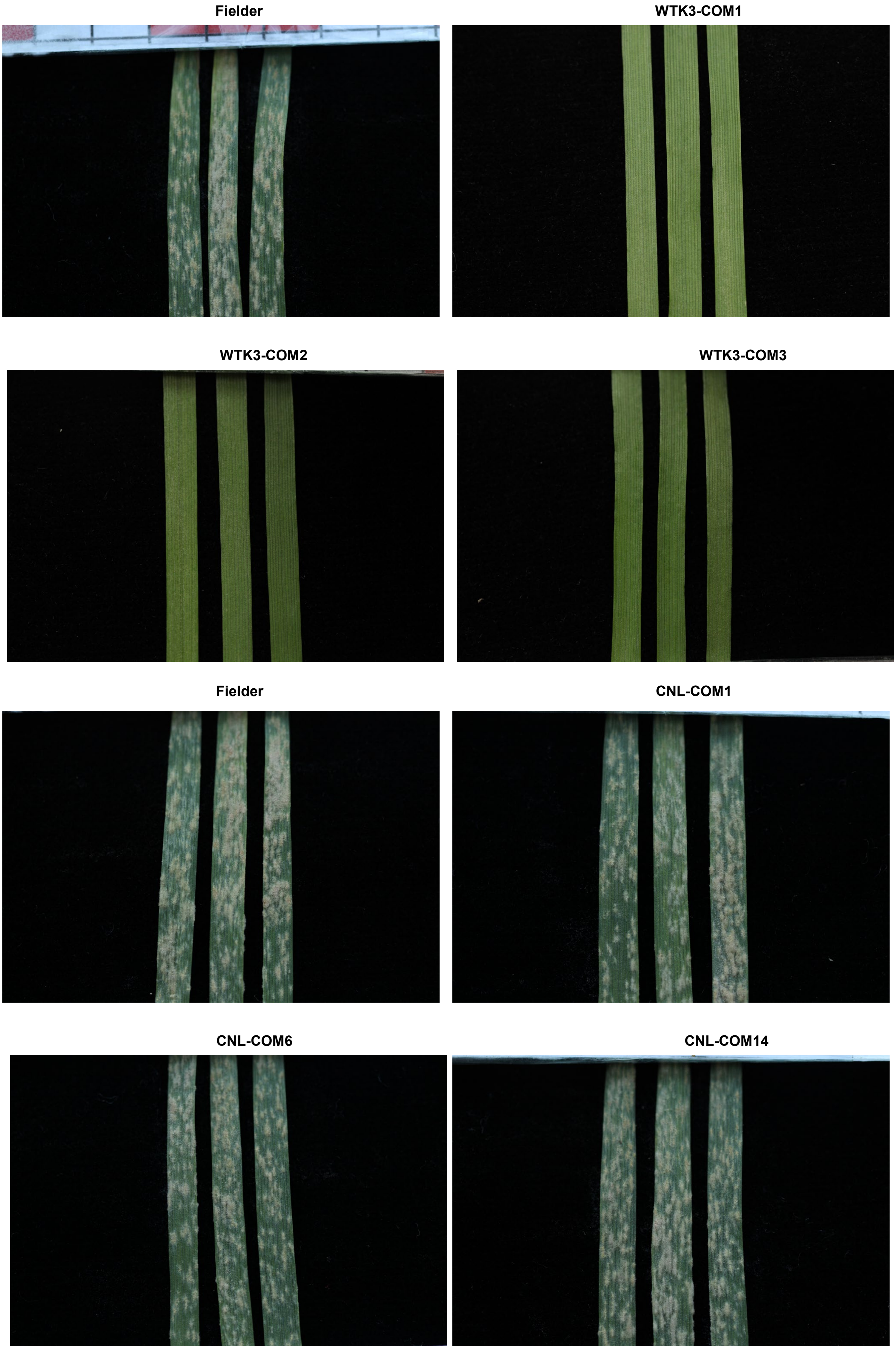
Fielder
WTK3-COM1
WTK3-COM2
WTK3-COM3
Fielder
CNL-COM1
CNL-COM6
CNL-COM14

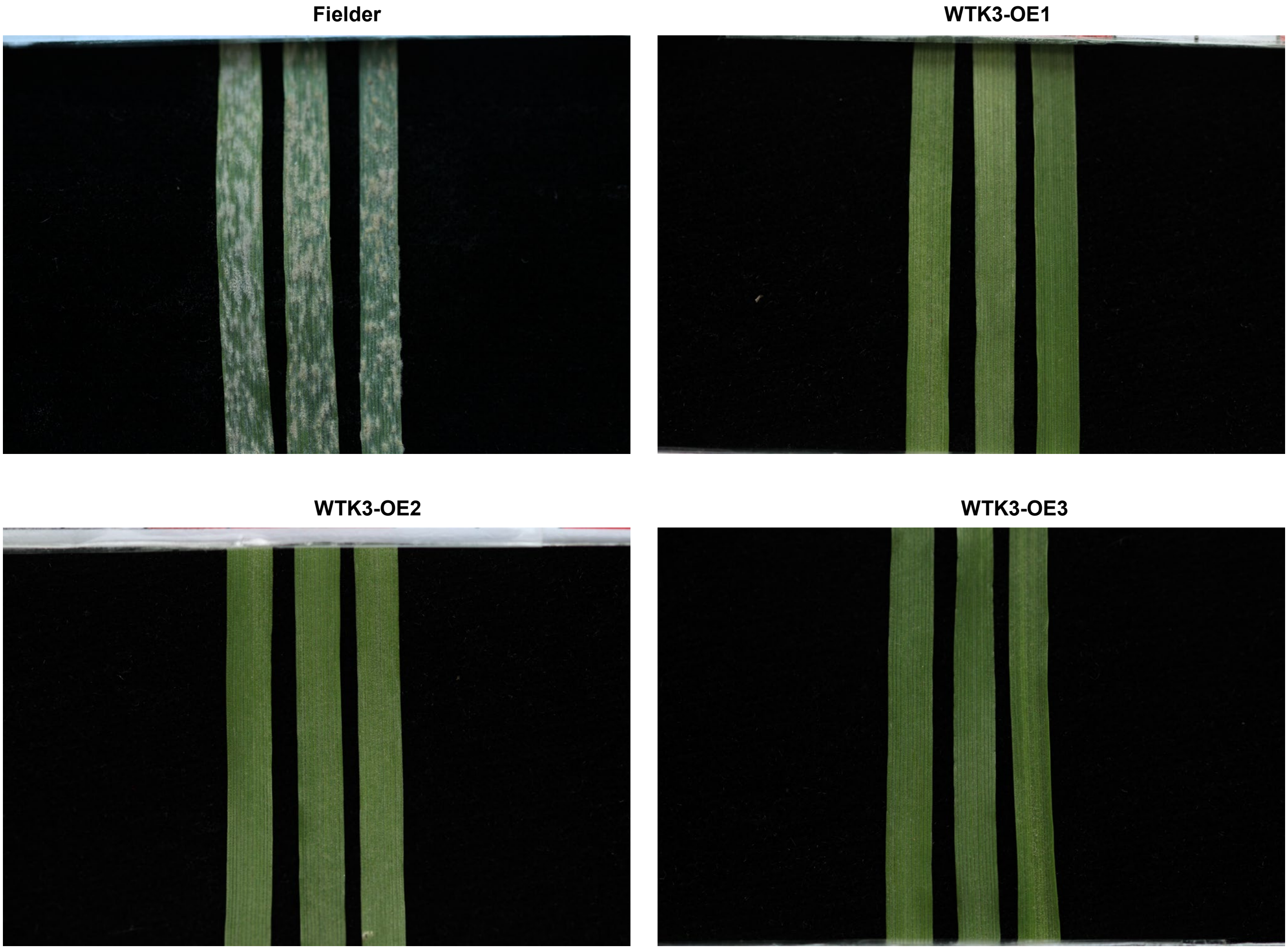

Fig. 2b Infection reactions of Fielder (Infection type IT 4), the T_1 transgenic plants of *ProWTK3*:*WTK3* (IT 0), *ProCNL*:*CNL* (IT 4) and *ProUbi*:*WTK3* (IT 0) to *Bgt* isolate E09, respectively. Three representative individuals of each transgenic line were photographed at 10 dpi; +/-: presence/absence of the transgene. **c** Powdery mildew resistance assessments of *WTK3* EMS mutants. Two-week-old HLT (IT 0) and 11 mutants (IT 3-4) were inoculated with *Bgt* isolate E09. Representative leaves were photographed at 10 dpi.

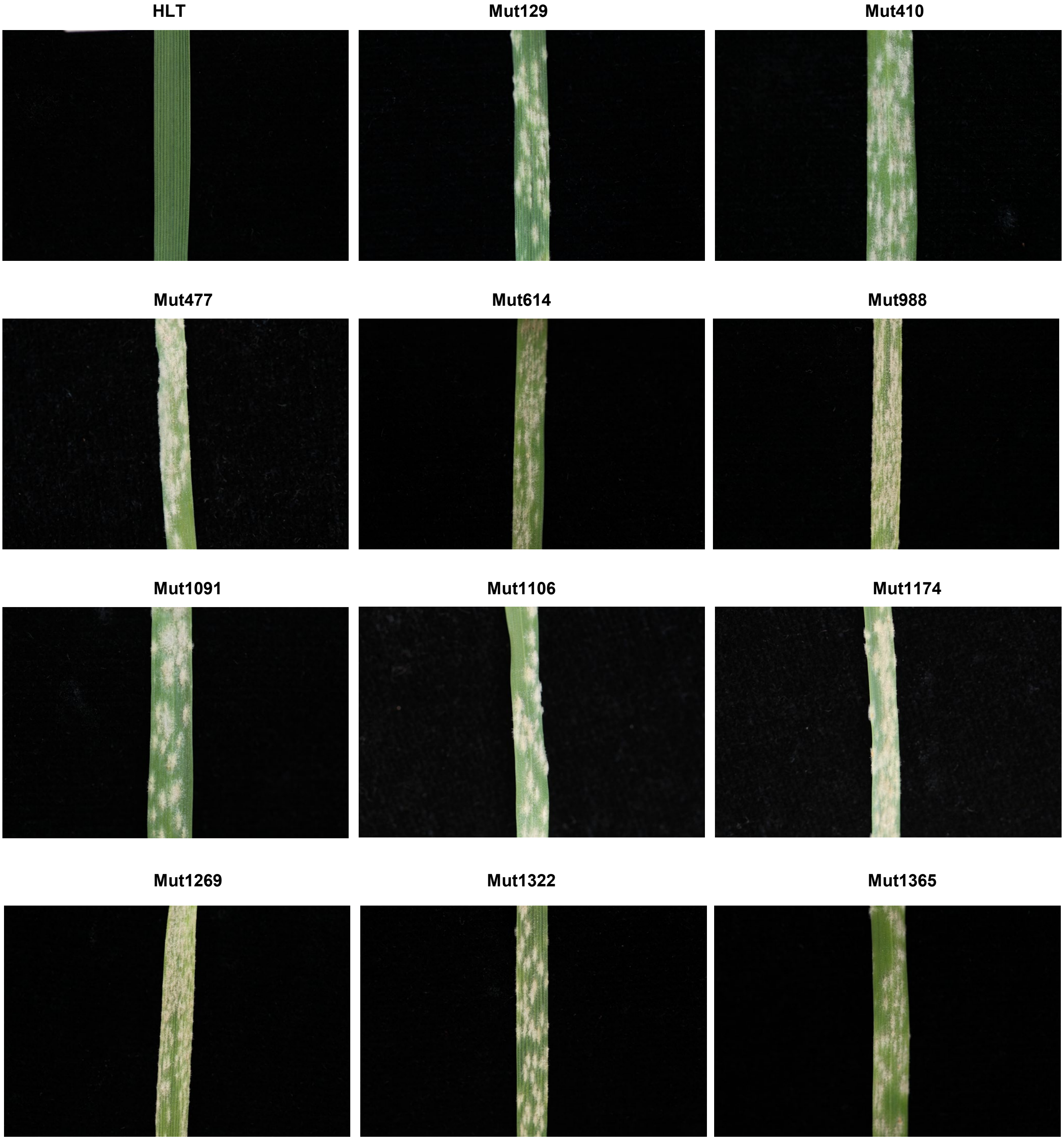

Fig. 2**c** Powdery mildew resistance assessments of *WTK3* EMS mutants. Two-week-old HLT (IT 0) and 11 mutants (IT 3-4) were inoculated with *Bgt* isolate E09. Representative leaves were photographed at 10 dpi.

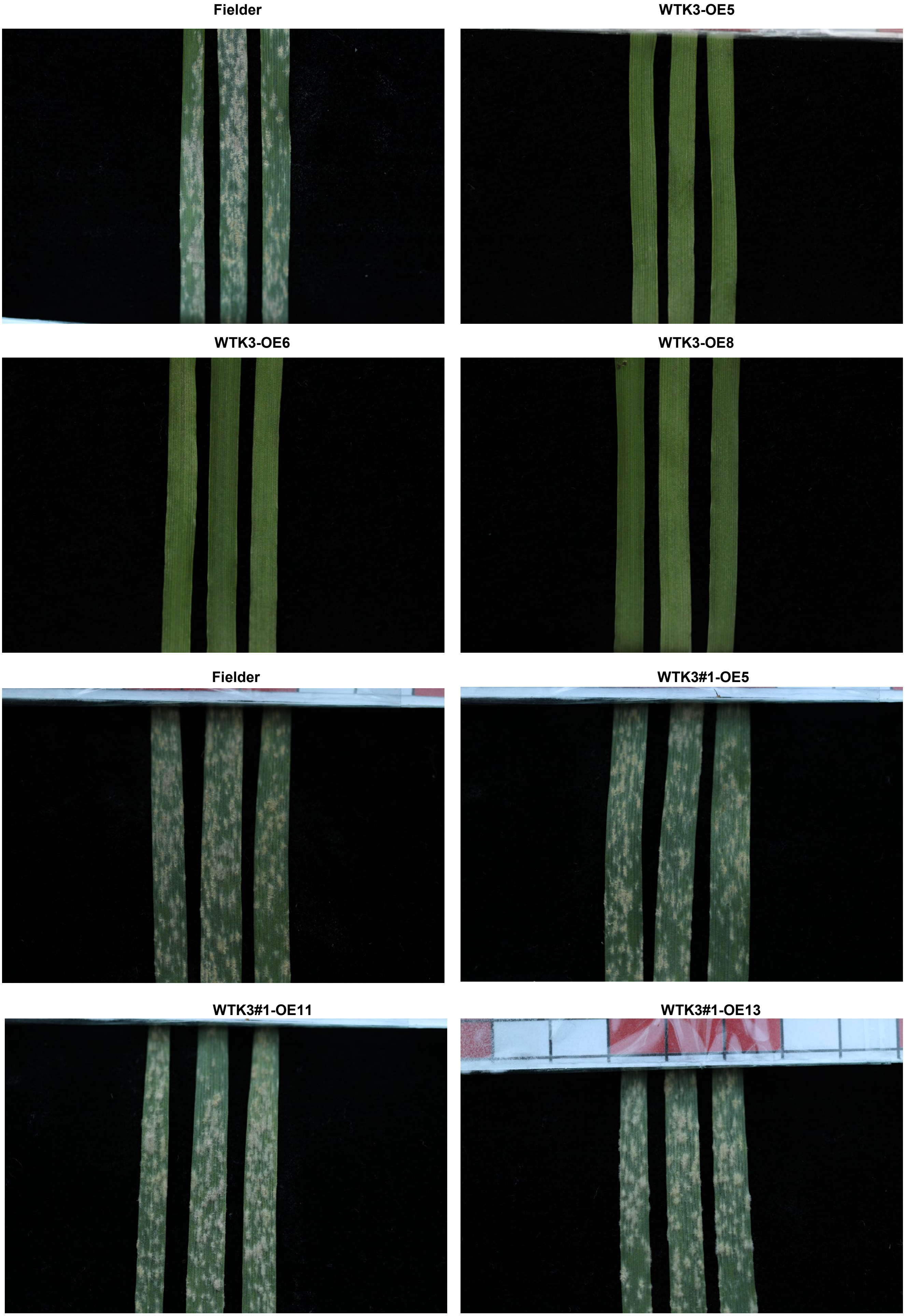

Fielder
WTK3-OE5
WTK3-OE6
WTK3-OE8
Fielder
WTK3#1-OE5
WTK3#1-OE11
WTK3#1-OE13

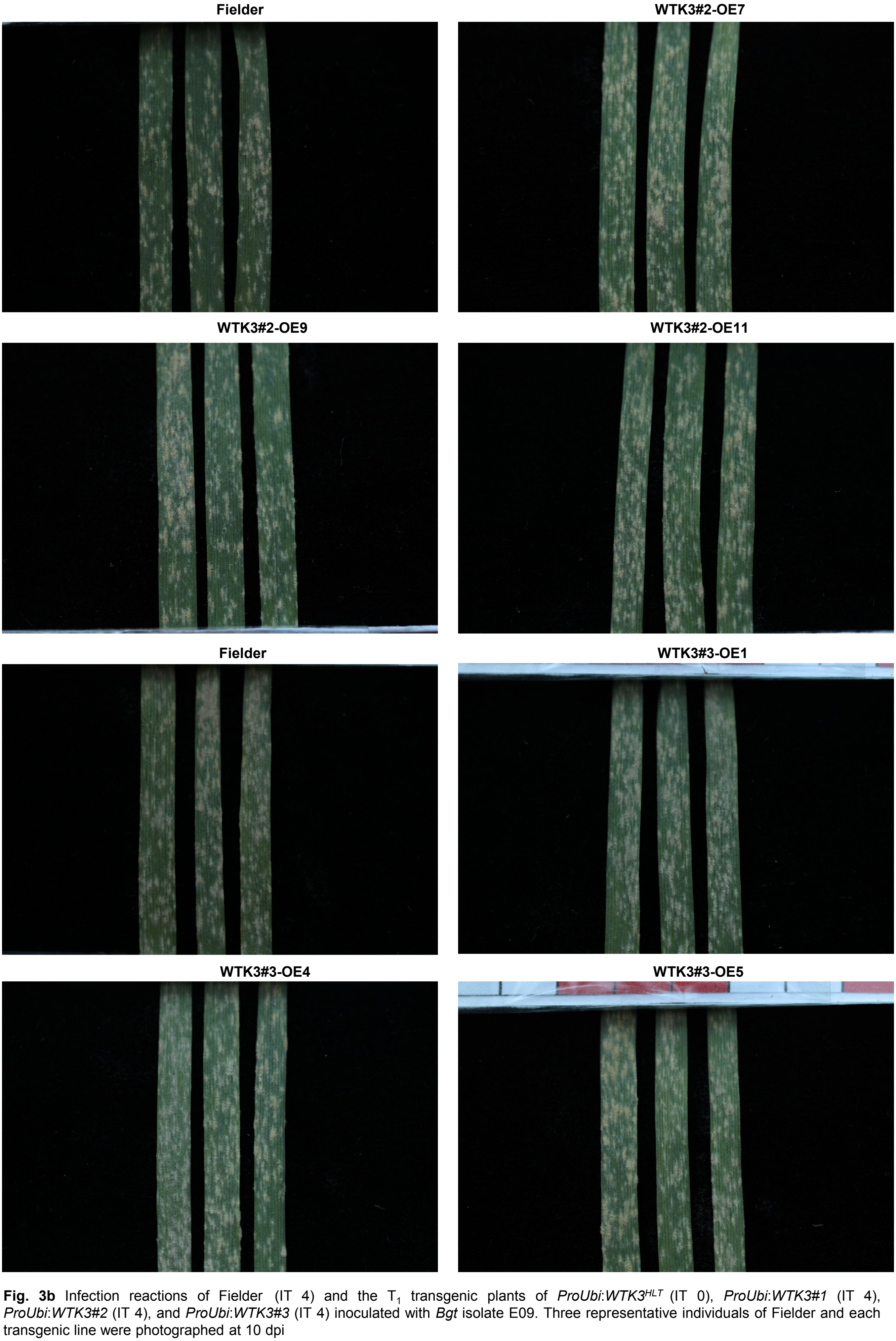

Fig. 3b Infection reactions of Fielder (IT 4) and the T_1 transgenic plants of *ProUbi*:*WTK3*^{HLT} (IT 0), *ProUbi*:*WTK3#1* (IT 4), *ProUbi*:*WTK3#2* (IT 4), and *ProUbi*:*WTK3#3* (IT 4) inoculated with *Bgt* isolate E09. Three representative individuals of Fielder and each transgenic line were photographed at 10 dpi

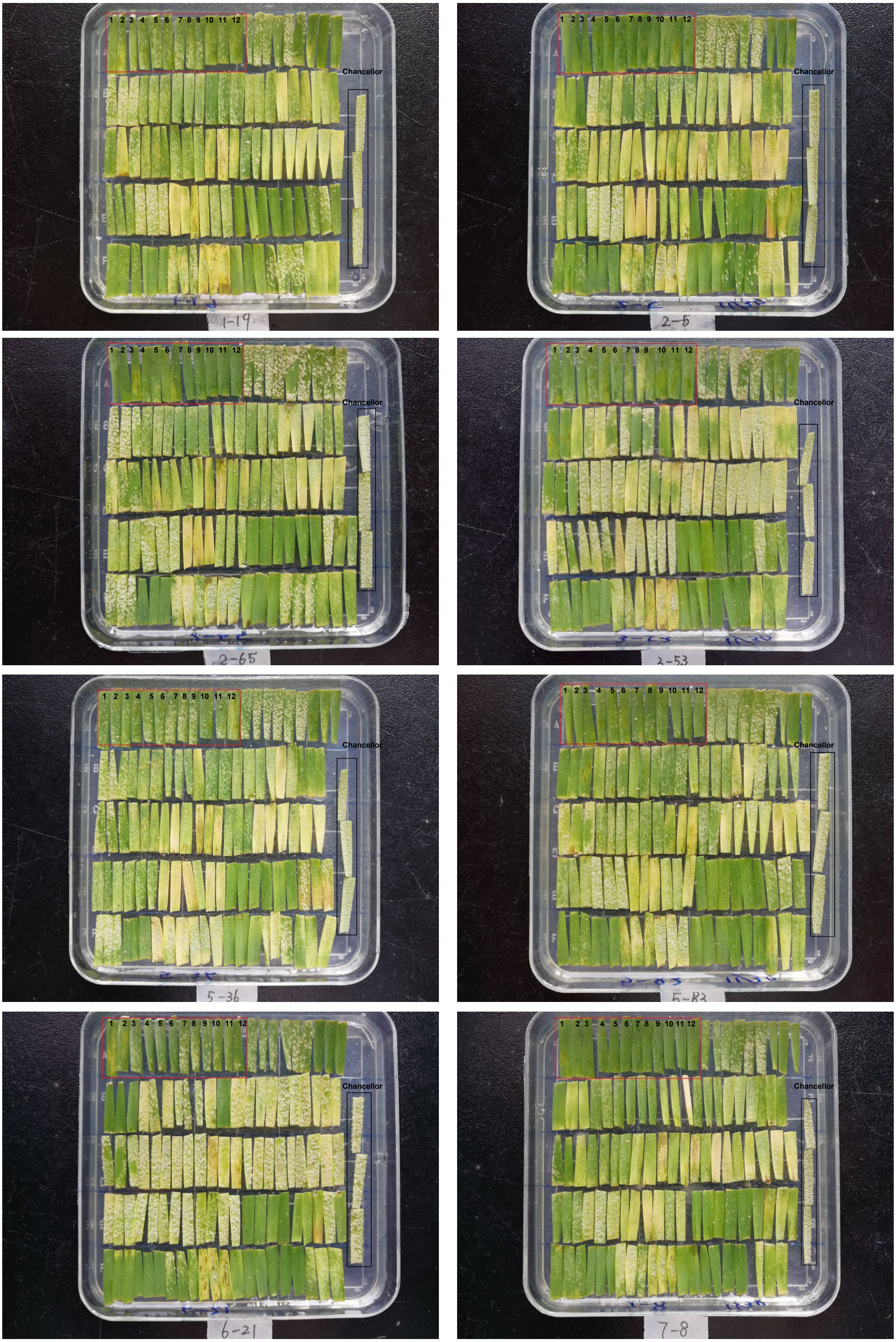

1 2 3 4 5 6 7 8 9 10 11 12
Chancellor
1-19
2-5
2-65
2-53
5-36
5-83
6-21
7-8

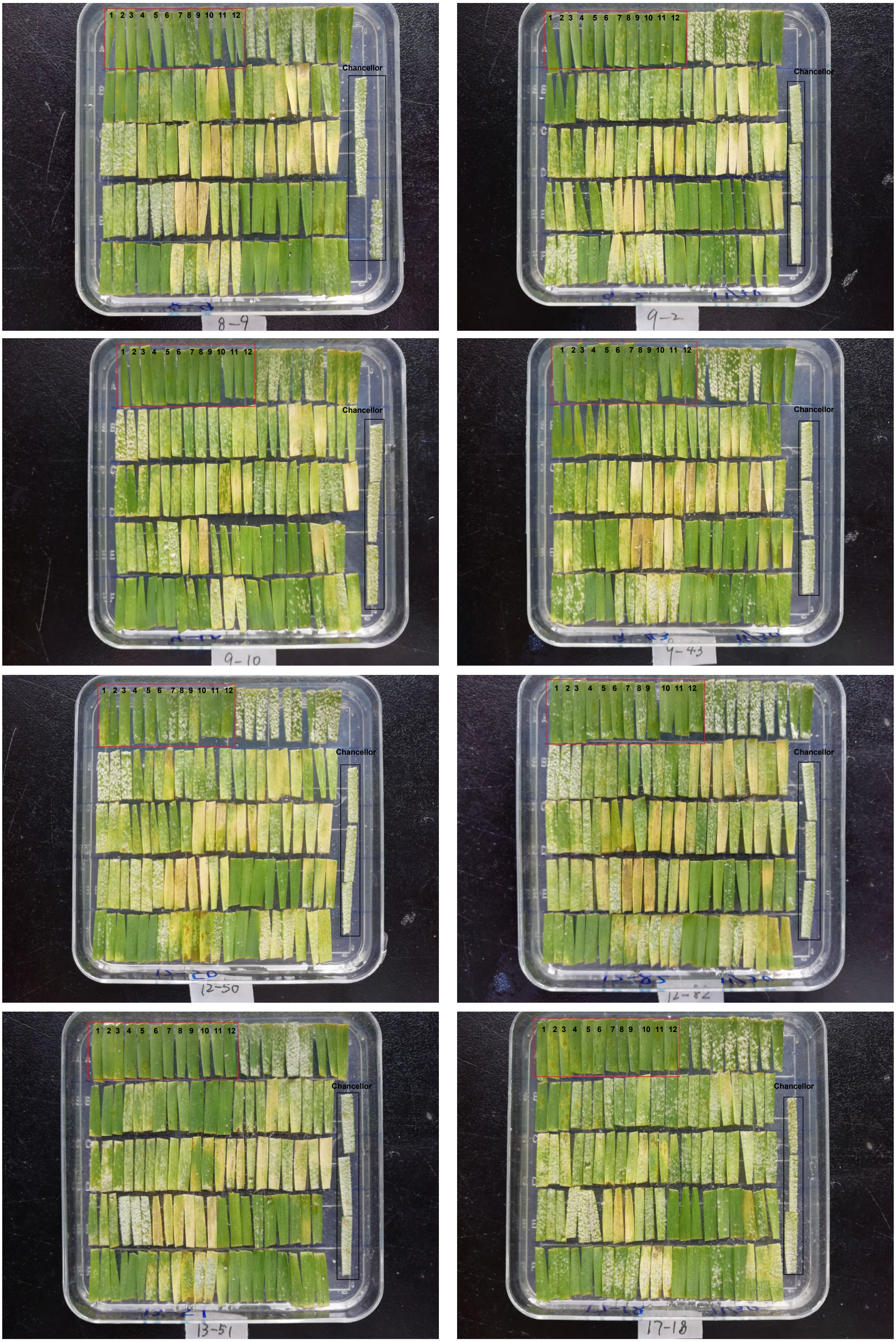
1 2 3 4 5 6 7 8 9 10 11 12
Chancellor
8-9
9-2
9-10
9-43
12-50
12-82
13-51
17-18

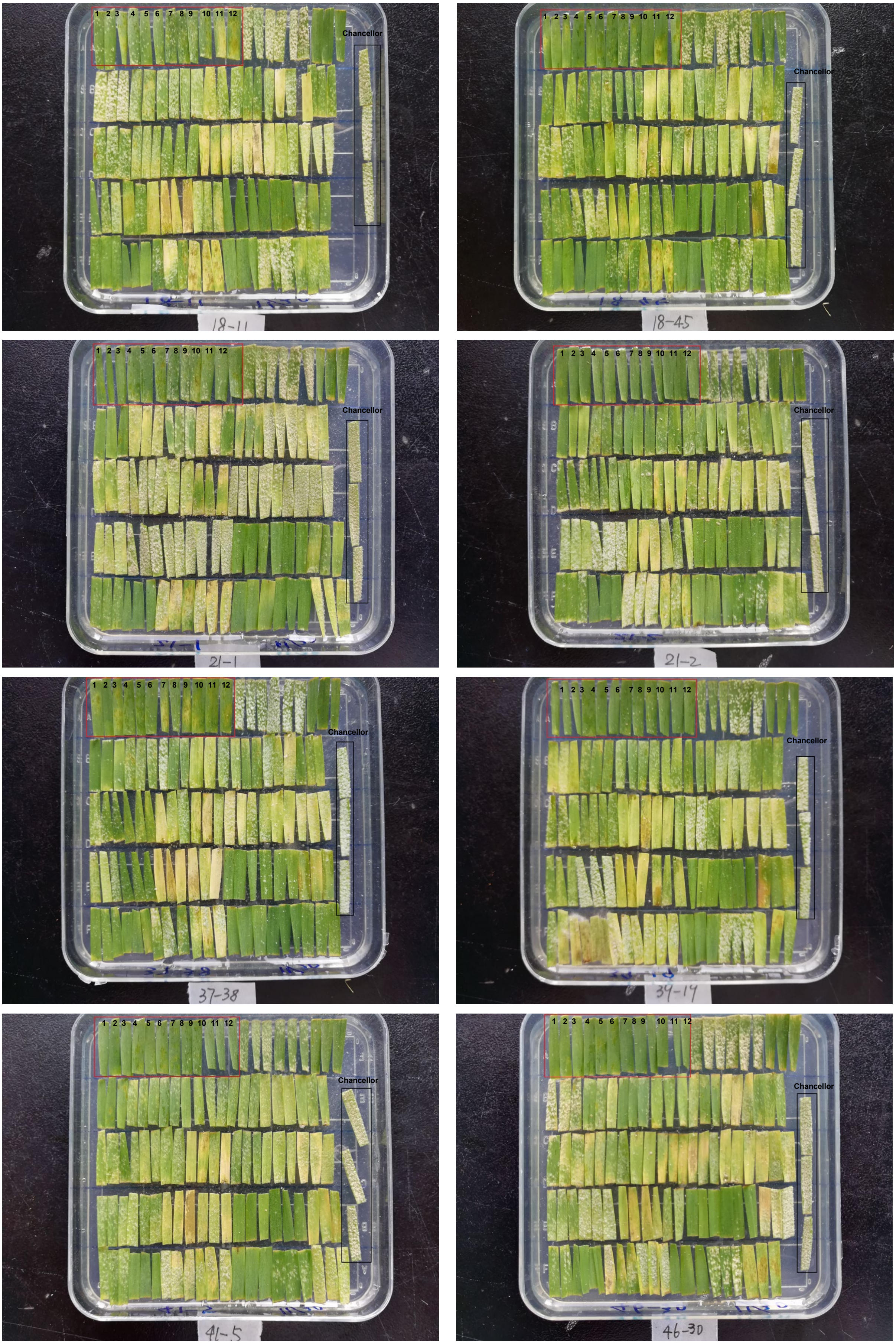
1 2 3 4 5 6 7 8 9 10 11 12
Chancellor
18-11
1 2 3 4 5 6 7 8 9 10 11 12
Chancellor
18-45
1 2 3 4 5 6 7 8 9 10 11 12
Chancellor
21-1
1 2 3 4 5 6 7 8 9 10 11 12
Chancellor
21-2
1 2 3 4 5 6 7 8 9 10 11 12
Chancellor
37-38
1 2 3 4 5 6 7 8 9 10 11 12
Chancellor
39-19
1 2 3 4 5 6 7 8 9 10 11 12
Chancellor
41-5
1 2 3 4 5 6 7 8 9 10 11 12
Chancellor
46-30

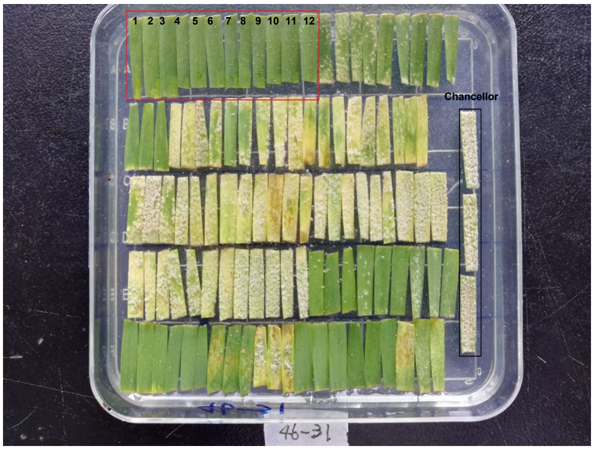
1 2 3 4 5 6 7 8 9 10 11 12
Chancellor
46-31

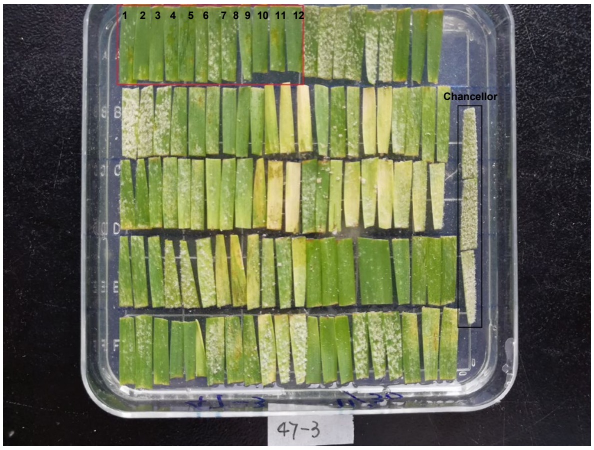
1 2 3 4 5 6 7 8 9 10 11 12
Chancellor
47-3

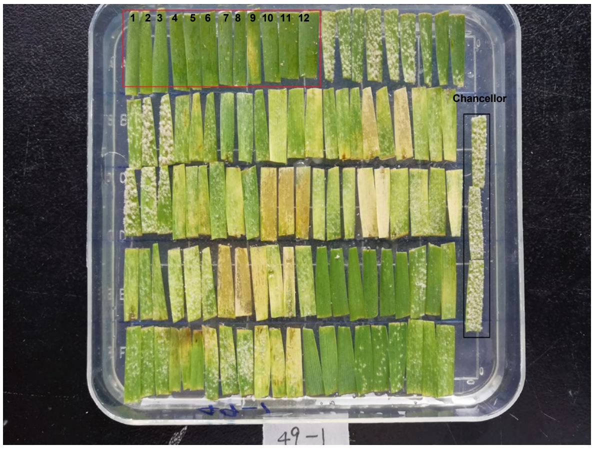
1 2 3 4 5 6 7 8 9 10 11 12
Chancellor
49-1

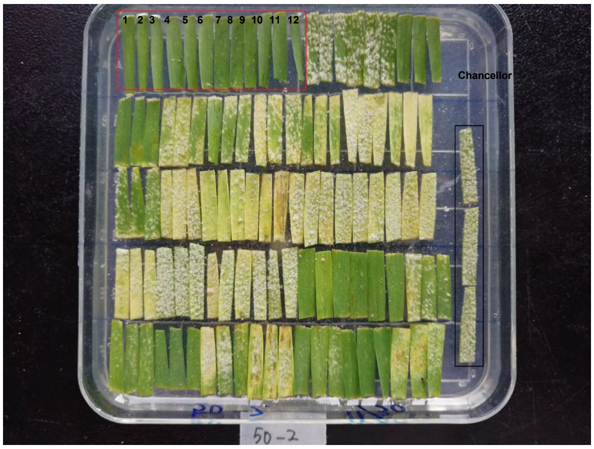
1 2 3 4 5 6 7 8 9 10 11 12
Chancellor
50-2

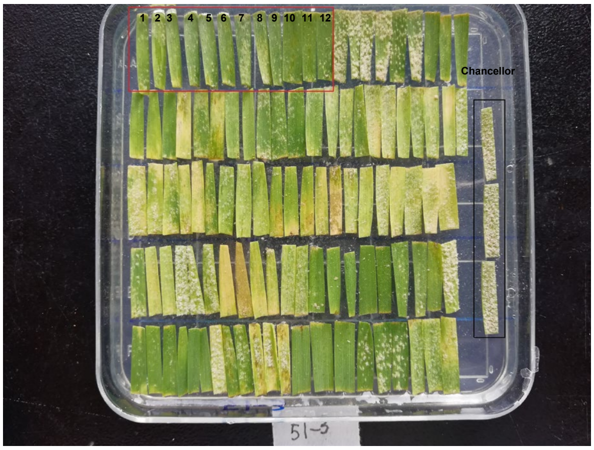
1 2 3 4 5 6 7 8 9 10 11 12
Chancellor
51-5

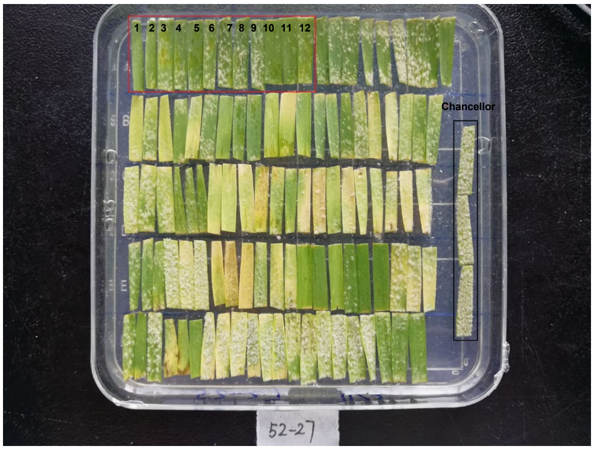
1 2 3 4 5 6 7 8 9 10 11 12
Chancellor
52-27

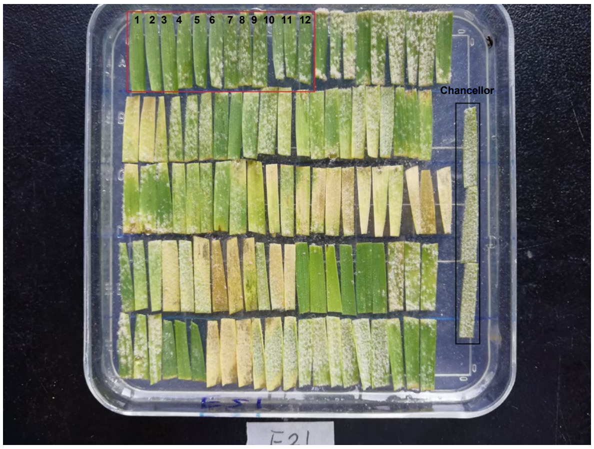
1 2 3 4 5 6 7 8 9 10 11 12
Chancellor

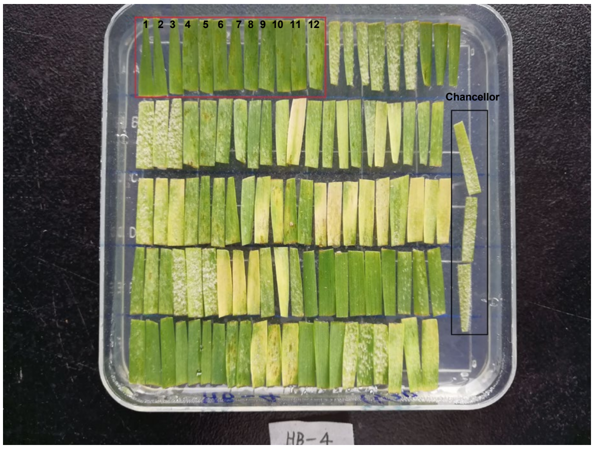
1 2 3 4 5 6 7 8 9 10 11 12
Chancellor
HB-4

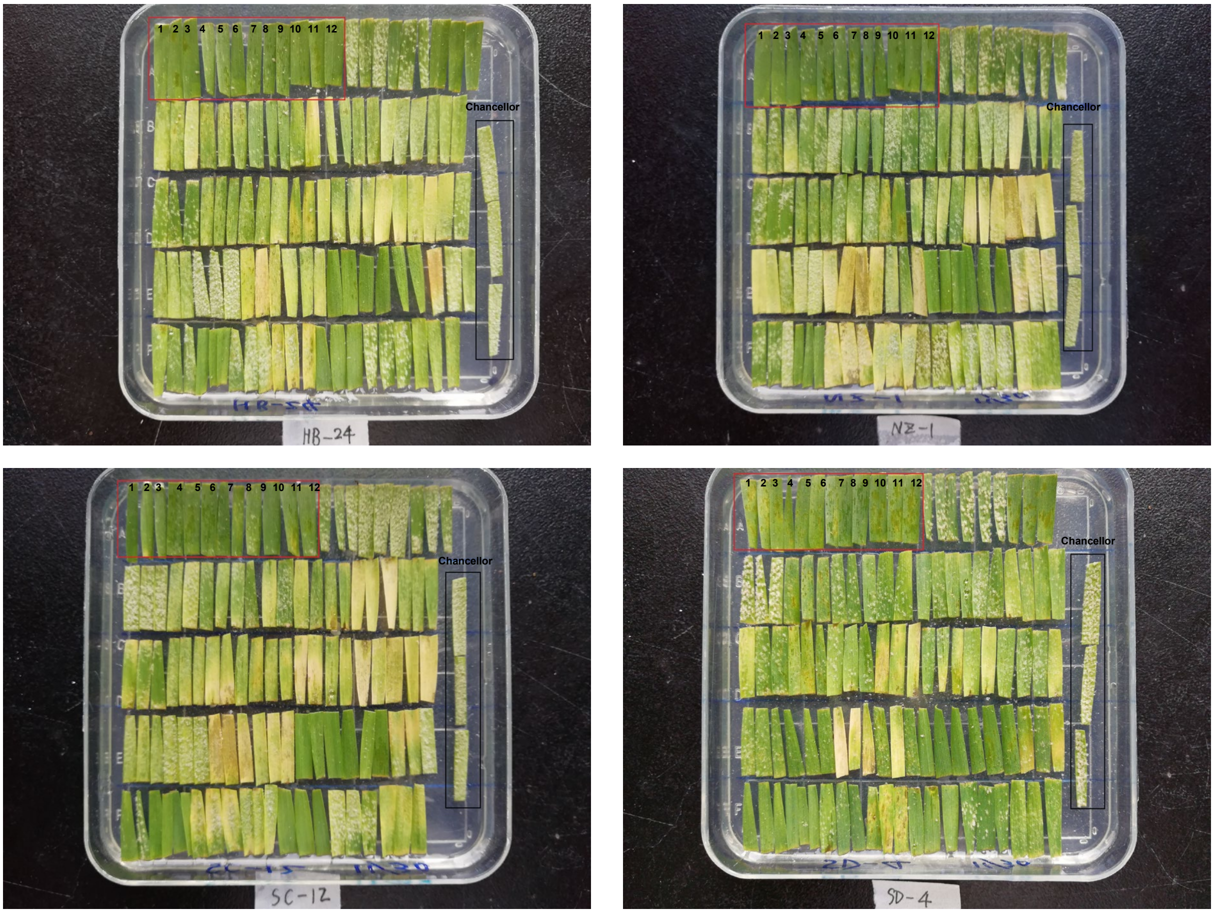

Supplementary Figure 1 Infection reactions of Chinese wheat landraces CYC, BHL, HLT and Hongmangmai (HMM) to 36 tested *Bgt* isolates. Common wheat cultivar Chancellor was used as the susceptible control. Two-week-old plants were inoculated with isolates, CYC(1-3, red rectangle), BHL(4-6, red rectangle), HLT(7-9, red rectangle), HMM(10-12, red rectangle), Chancellor (black rectangle). Representative leaves were photographed at 10 dpi.

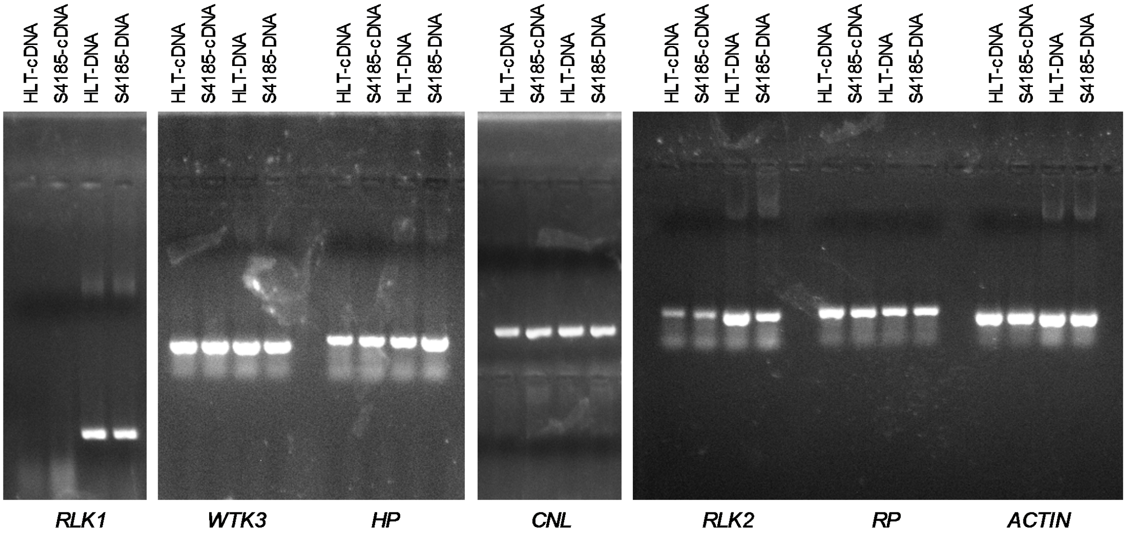

Supplementary Figure 2a Expression analysis of *MlHLT* candidate genes in HLT and S4185.

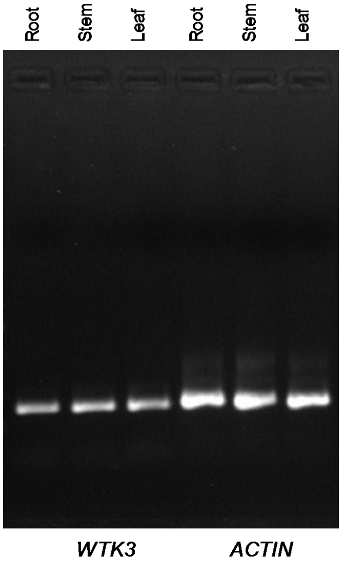

Supplementary Figure 2b *WTK3* expression survey in root, stem, and leaf of HLT by RT-PCR.

Supplementary Fig. 2c original data

WTK3 /ACTIN

hpi	Genotype	ΔCt	$2^{-\Delta\Delta Ct}$	Normalized to mean in control
0 h	HLT	4.66333333	0.03946361	0.986590196
0 h	HLT	5.00333333	0.03117788	0.779447013
0 h	HLT	4.83333333	0.03507694	0.876923475
0 h	S4185	4.31666667	0.05018268	1.254567003
0 h	S4185	4.98666667	0.03154015	0.788503751
0 h	S4185	4.76666667	0.03673587	0.918396802
4 h	HLT	4.21	0.05403358	1.350839424
4 h	HLT	4.61	0.04094979	1.023744847
4 h	HLT	4.53	0.04328467	1.082116772
4 h	S4185	4.58333333	0.04171375	1.042843636
4 h	S4185	4.11333333	0.0577781	1.444452594
4 h	S4185	4.11333333	0.0577781	1.444452594
12 h	HLT	3.29	0.10223776	2.555943933
12 h	HLT	3.17	0.11110534	2.777633379
12 h	HLT	3.09	0.11744034	2.936008591
12 h	S4185	3.20666667	0.10831713	2.707928268
12 h	S4185	3.20666667	0.10831713	2.707928268
12 h	S4185	3.30666667	0.10106346	2.526586413
24 h	HLT	2.55333333	0.17036096	4.259023991
24 h	HLT	2.33333333	0.19842513	4.960628287
24 h	HLT	2.51333333	0.17515046	4.378761453
24 h	S4185	2.38333333	0.19166604	4.791651077
24 h	S4185	2.63333333	0.16117129	4.029282214
24 h	S4185	2.50333333	0.17636873	4.409218147
36 h	HLT	2.6	0.16493849	4.123462221
36 h	HLT	2.82	0.14161049	3.540262142
36 h	HLT	2.75	0.14865089	3.716272234
36 h	S4185	3.15333333	0.11239632	2.809907924
36 h	S4185	2.94333333	0.13000749	3.250187293
36 h	S4185	3.06333333	0.11963129	2.99078221
48 h	HLT	4.69666667	0.03856226	0.964056445
48 h	HLT	4.47666667	0.04491476	1.122868921
48 h	HLT	4.43666667	0.04617748	1.154437063
48 h	S4185	4.07333333	0.05940247	1.485061684
48 h	S4185	4.38333333	0.04791651	1.197912769
48 h	S4185	4.27333333	0.05171285	1.292821285

Supplementary Fig. 2d original data

PR1/ACTIN

hpi	Genotype	ΔCt	$2^{-\Delta\Delta Ct}$	Normalized to mean in control
0 h	HLT	6.02	0.015409886	0.030819772
0 h	HLT	6.15	0.014082038	0.028164077
0 h	HLT	5.91	0.016630784	0.033261568
0 h	S4185	0.866666667	0.54841249	1.09682498
0 h	S4185	1.146666667	0.4516676	0.903335201
0 h	S4185	0.996666667	0.501156581	1.002313162
4 h	HLT	3.88	0.067920929	0.135841858
4 h	HLT	4.13	0.057114466	0.114228931
4 h	HLT	4.07	0.059539875	0.11907975
4 h	S4185	0.81	0.570381858	1.140763716
4 h	S4185	0.83	0.562529242	1.125058485
4 h	S4185	0.51	0.702222438	1.404444876
12 h	HLT	-0.19	1.140763716	2.281527432
12 h	HLT	-0.11	1.079228237	2.158456473
12 h	HLT	-0.41	1.328685814	2.657371628
12 h	S4185	-0.26333333	1.200248667	2.400497333
12 h	S4185	0.476666667	0.718636109	1.437272219
12 h	S4185	-0.09333333	1.066832243	2.133664486
24 h	HLT	-5.51	45.56960626	91.13921252
24 h	HLT	-5.15	35.50622311	71.01244621
24 h	HLT	-5.78	54.94818793	109.8963759
24 h	S4185	-4.96666667	31.26911899	62.53823798
24 h	S4185	-4.93666667	30.62560983	61.25121965
24 h	S4185	-5.11666667	34.69525985	69.3905197
36 h	HLT	-6.48	89.26359465	178.5271893
36 h	HLT	-6.17	72.00374302	144.007486
36 h	HLT	-6.56	94.35322991	188.7064598
36 h	S4185	-3.08	8.456144324	16.91228865
36 h	S4185	-3.05	8.282119391	16.56423878
36 h	S4185	-3.55	11.71268557	23.42537114
48 h	HLT	-4.09666667	17.10879998	34.21759995
48 h	HLT	-4.24666667	18.98340216	37.96680432
48 h	HLT	-4.03666667	16.41185794	32.82371589
48 h	S4185	-0.55	1.464085696	2.928171392
48 h	S4185	-0.88	1.840375301	3.680750602
48 h	S4185	-0.84	1.790050142	3.580100284

PR2/ACTIN

hpi	Genotype	ΔCt	$2^{-\Delta\Delta Ct}$	Normalized to mean in control
0 h	HLT	6.226666667	0.013353237	0.267064742
0 h	HLT	6.236666667	0.013261	0.265219991
0 h	HLT	6.026666667	0.015338841	0.30677683
0 h	S4185	4.28	0.051474439	1.029488772
0 h	S4185	4.65	0.03983002	0.796600392
0 h	S4185	4.13	0.057114466	1.142289313
4 h	HLT	3.953333333	0.064554732	1.291094644
4 h	HLT	4.233333333	0.053166698	1.063333951
4 h	HLT	4.253333333	0.052434736	1.048694718
4 h	S4185	2.97	0.127626516	2.552530314
4 h	S4185	2.94	0.13030822	2.606164402
4 h	S4185	2.67	0.157126672	3.142533436
12 h	HLT	1.316666667	0.401461441	8.029228819
12 h	HLT	2.406666667	0.188591078	3.771821567
12 h	HLT	1.656666667	0.317171123	6.343422469
12 h	S4185	1.01	0.496546248	9.930924954
12 h	S4185	1.74	0.299369676	5.987393523
12 h	S4185	1.49	0.356012549	7.120250978
24 h	HLT	-3.96333333	15.59847769	311.9695538
24 h	HLT	-3.29333333	9.80374754	196.0749508
24 h	HLT	-4.06333333	16.71803445	334.3606889
24 h	S4185	-2.53	5.775716782	115.5143356
24 h	S4185	-2.39	5.241573615	104.8314723
24 h	S4185	-2.28	4.856779538	97.13559075
36 h	HLT	-3.64666667	12.52437466	250.4874932
36 h	HLT	-3.68666667	12.87648276	257.5296552
36 h	HLT	-3.69666667	12.96604578	259.3209156
36 h	S4185	-0.01	1.00695555	20.139111
36 h	S4185	0.32	0.801069878	16.02139755
36 h	S4185	0.06	0.959264119	19.18528239
48 h	HLT	0.316666667	0.802922882	16.05845764
48 h	HLT	0.236666667	0.848703971	16.97407943
48 h	HLT	0.336666667	0.791868805	15.83737611
48 h	S4185	3.093333333	0.117169312	2.343386241
48 h	S4185	3.353333333	0.097846677	1.956933541
48 h	S4185	3.363333333	0.097170801	1.943416013

PR3/ACTIN

hpi	Genotype	ΔCt	$2^{-\Delta\Delta Ct}$	Normalized to mean in control
0 h	HLT	2.87	0.136786713	0.911911418
0 h	HLT	3.82	0.070805243	0.472034952
0 h	HLT	2.77	0.146604369	0.977362458
0 h	S4185	3.346666667	0.098299871	0.655332473
0 h	S4185	3.226666667	0.106825897	0.712172645
0 h	S4185	1.726666667	0.302149264	2.014328427
4 h	HLT	-0.10666667	1.076737568	7.178250455
4 h	HLT	0.113333333	0.92444966	6.162997735
4 h	HLT	-0.13666667	1.099362113	7.329080756
4 h	S4185	-0.06	1.042465761	6.949771739
4 h	S4185	-0.31	1.2397077	8.264718
4 h	S4185	-0.75	1.681792831	11.2119522
12 h	HLT	-1.39	2.620786808	17.47191205
12 h	HLT	-1.19	2.281527432	15.21018288
12 h	HLT	-1.43	2.694467154	17.96311436
12 h	S4185	-1.26	2.394957409	15.96638273
12 h	S4185	-0.92	1.892115293	12.61410196
12 h	S4185	-1.17	2.250116969	15.0007798
24 h	HLT	-4.58666667	24.02836646	160.1891097
24 h	HLT	-4.50666667	22.73221983	151.5481322
24 h	HLT	-4.92666667	30.41406329	202.7604219
24 h	S4185	-2.63666667	6.218931243	41.45954162
24 h	S4185	-2.67666667	6.393769198	42.62512799
24 h	S4185	-2.78666667	6.900336256	46.00224171
36 h	HLT	-4.37666667	20.77341741	138.4894494
36 h	HLT	-4.44666667	21.80620283	145.3746855
36 h	HLT	-4.47666667	22.26439751	148.4293167
36 h	S4185	-1.96333333	3.899619423	25.99746282
36 h	S4185	-2.15333333	4.448544343	29.65696229
36 h	S4185	-2.05333333	4.150638637	27.67092424
48 h	HLT	-0.86333333	1.819236788	12.12824525
48 h	HLT	-1.32333333	2.502436279	16.68290853
48 h	HLT	-1.73333333	3.324951585	22.1663439
48 h	S4185	0.663333333	0.631417726	4.209451504
48 h	S4185	0.583333333	0.667419927	4.449466181
48 h	S4185	0.623333333	0.649169294	4.327795294

PR4/ACTIN

hpi	Genotype	ΔCt	$2^{-\Delta\Delta Ct}$	Normalized to mean in control
0 h	HLT	-0.82	1.765405993	0.882702996
0 h	HLT	1.08	0.473028823	0.236514412
0 h	HLT	0.18	0.882702996	0.441351498
0 h	S4185	-1.21	2.313376368	1.156688184
0 h	S4185	-0.78	1.717130873	0.858565436
0 h	S4185	-1.27	2.411615655	1.205807828
4 h	HLT	-2.34	5.063026376	2.531513188
4 h	HLT	-1.07	2.099433367	1.049716684
4 h	HLT	-2.59	6.02098699	3.010493495
4 h	S4185	-2.21	4.626752736	2.313376368
4 h	S4185	-3.18	9.063071082	4.531535541
4 h	S4185	-2.17	4.500233939	2.250116969
12 h	HLT	-3.99	15.88947993	7.944739963
12 h	HLT	-2.68	6.408559021	3.20427951
12 h	HLT	-3.44	10.85283462	5.42641731
12 h	S4185	-1.02	2.02791896	1.01395948
12 h	S4185	-1.37	2.584705661	1.292352831
12 h	S4185	-1.77	3.410539567	1.705269784
24 h	HLT	-7.17	144.007486	72.00374302
24 h	HLT	-6.92	121.0953788	60.54768939
24 h	HLT	-7.22	149.0858991	74.54294953
24 h	S4185	-5.98	63.11889309	31.55944654
24 h	S4185	-5.79	55.3303828	27.6651914
24 h	S4185	-5.78	54.94818793	27.47409397
36 h	HLT	-7.73	212.3058037	106.1529019
36 h	HLT	-8.05	265.0278205	132.5139103
36 h	HLT	-7.9	238.8564458	119.4282229
36 h	S4185	-3.41	10.62948651	5.314745
36 h	S4185	-3.63	12.38051995	6.190259974
36 h	S4185	-2.56	5.897076869	2.948538435
48 h	HLT	-4.5	22.627417	11.3137085
48 h	HLT	-4.38	20.82146969	10.41073484
48 h	HLT	-4.58	23.91758798	11.95879399
48 h	S4185	-3.19	9.126109727	4.563054863
48 h	S4185	-2.43	5.388934307	2.694467154
48 h	S4185	-3.07	8.397733469	4.198866734

PR5/ACTIN

hpi	Genotype	ΔCt	$2^{-\Delta\Delta Ct}$	Normalized to mean in control
0 h	HLT	0.028164077	0.704101924	0.704101924
0 h	HLT	0.022405551	0.560138769	0.560138769
0 h	HLT	0.003721242	0.093031054	0.093031054
0 h	S4185	0.057911754	1.447793847	1.447793847
0 h	S4185	0.035158078	0.878951941	0.878951941
0 h	S4185	0.047038961	1.175974021	1.175974021
4 h	HLT	0.026278013	0.656950324	0.656950324
4 h	HLT	0.01845301	0.461325258	0.461325258
4 h	HLT	0.018971795	0.474294877	0.474294877
4 h	S4185	0.360982299	9.024557472	9.024557472
4 h	S4185	0.233258248	5.831456197	5.831456197
4 h	S4185	0.289172046	7.229301149	7.229301149
12 h	HLT	0.025382887	0.634572185	0.634572185
12 h	HLT	0.017824433	0.445610827	0.445610827
12 h	HLT	0.046070913	1.151772826	1.151772826
12 h	S4185	0.228457863	5.711446564	5.711446564
12 h	S4185	0.129408115	3.235202887	3.235202887
12 h	S4185	0.128514228	3.212855708	3.212855708
24 h	HLT	7.260153243	181.5038311	181.5038311
24 h	HLT	7.412704495	185.3176124	185.3176124
24 h	HLT	9.713559075	242.8389769	242.8389769
24 h	S4185	2	50	50
24 h	S4185	2.203810232	55.09525579	55.09525579
24 h	S4185	2.496661098	62.41652745	62.41652745
36 h	HLT	23.42537114	585.6342784	585.6342784
36 h	HLT	17.02992292	425.748073	425.748073
36 h	HLT	22.16175149	554.0437872	554.0437872
36 h	S4185	0.189464571	4.73661427	4.73661427
36 h	S4185	0.115023456	2.875586408	2.875586408
36 h	S4185	0.10153155	2.538288739	2.538288739
48 h	HLT	0.655196702	16.37991755	16.37991755
48 h	HLT	0.535886731	13.39716828	13.39716828
48 h	HLT	0.510506063	12.76265157	12.76265157
48 h	S4185	0.125869444	3.146736094	3.146736094
48 h	S4185	0.072795849	1.819896229	1.819896229
48 h	S4185	0.082469244	2.061731111	2.061731111

PR9/ACTIN

hpi	Genotype	ΔCt	$2^{-\Delta\Delta Ct}$	Normalized to mean in control
0 h	HLT	12.93	0.000128139	0.032034811
0 h	HLT	13.84	6.81938E-05	0.017048449
0 h	HLT	12.75	0.000145167	0.036291721
0 h	S4185	7.53	0.005410584	1.352645965
0 h	S4185	8.09	0.003670011	0.917502685
0 h	S4185	8.01	0.003879268	0.96981689
4 h	HLT	3.9	0.066985841	16.74646035
4 h	HLT	4	0.0625	15.625
4 h	HLT	3.99	0.062934722	15.73368047
4 h	S4185	1.22	0.429282718	107.3206796
4 h	S4185	1.17	0.444421341	111.1053351
4 h	S4185	1.12	0.460093825	115.0234563
12 h	HLT	3.1	0.116629124	29.15728099
12 h	HLT	3.28	0.102948877	25.73721929
12 h	HLT	3.3	0.10153155	25.38288739
12 h	S4185	0.81	0.570381858	142.5954645
12 h	S4185	2.16	0.223756268	55.93906693
12 h	S4185	2.23	0.213158723	53.28968074
24 h	HLT	-5.4	42.22425314	10556.06329
24 h	HLT	-5.44	43.41133848	10852.83462
24 h	HLT	-5.45	43.71328822	10928.32205
24 h	S4185	-3.58	11.95879399	2989.698497
24 h	S4185	-3.89	14.82540899	3706.352248
24 h	S4185	-3.7	12.99603834	3249.009585
36 h	HLT	-4.31	19.8353232	4958.8308
36 h	HLT	-4.33	20.11221399	5028.053498
36 h	HLT	-4.04	16.44982123	4112.455307
36 h	S4185	-1.68	3.20427951	801.0698776
36 h	S4185	-1.11	2.158456473	539.6141183
36 h	S4185	-1.23	2.345669898	586.4174746
48 h	HLT	0.93	0.524858342	131.2145855
48 h	HLT	1.72	0.303548721	75.88718027
48 h	HLT	1.14	0.453759578	113.4398944
48 h	S4185	3.81	0.071297732	17.82443306
48 h	S4185	2.64	0.160428237	40.1070593
48 h	S4185	4.75	0.037162722	9.290680586
